# Automated Left Ventricular Dimension Assessment Using Artificial Intelligence Developed and Validated by a UK-Wide Collaborative

**DOI:** 10.1161/CIRCIMAGING.120.011951

**Published:** 2021-05-17

**Authors:** James P. Howard, Catherine C. Stowell, Graham D. Cole, Kajaluxy Ananthan, Camelia D. Demetrescu, Keith Pearce, Ronak Rajani, Jobanpreet Sehmi, Kavitha Vimalesvaran, G. Sunthar Kanaganayagam, Eleanor McPhail, Arjun K. Ghosh, John B. Chambers, Amar P. Singh, Massoud Zolgharni, Bushra Rana, Darrel P. Francis, Matthew J. Shun-Shin

**Affiliations:** 1 Imperial College London (J.P.H., C.C.S., G.D.C., K.A., K.V., D.P.F., M.J.S.-S.).; 2 Hammersmith Hospital, London (J.P.H., C.C.S., D.P.F.).; 3 St Mary’s Hospital, London (M.J.S.-S.).; 4 Charing Cross Hospital, London (G.D.C., B.R.).; 5 Guy’s and St Thomas’ NHS Foundation Trust (C.D.D., R.R., J.B.C.).; 6 Manchester University Foundation Trust, Wythenshawe Hospital Manchester (K.P.).; 7 School of Biomedical Engineering and Imaging Sciences, King’s College London (R.R.).; 8 West Hertfordshire Hospitals NHS Trust (J.S.).; 9 Chelsea and Westminster and Imperial NHS Trust (G.S.K.).; 10 King’s College Hospital, London (E.M.).; 11 Barts Heart Centre, St Bartholomew’s Hospital, London (A.K.G.).; 12 London North West University Healthcare NHS Trust (A.P.S.).; 13 University of West London (M.Z.).

**Keywords:** consensus, echocardiography, hospital, left ventricle, machine learning

## Abstract

Artificial intelligence (AI) for echocardiography requires training and validation to standards expected of humans. We developed an online platform and established the Unity Collaborative to build a dataset of expertise from 17 hospitals for training, validation, and standardization of such techniques.

**Methods:**

The training dataset consisted of 2056 individual frames drawn at random from 1265 parasternal long-axis video-loops of patients undergoing clinical echocardiography in 2015 to 2016. Nine experts labeled these images using our online platform. From this, we trained a convolutional neural network to identify keypoints. Subsequently, 13 experts labeled a validation dataset of the end-systolic and end-diastolic frame from 100 new video-loops, twice each. The 26-opinion consensus was used as the reference standard. The primary outcome was precision SD, the SD of the differences between AI measurement and expert consensus.

**Results:**

In the validation dataset, the AI’s precision SD for left ventricular internal dimension was 3.5 mm. For context, precision SD of individual expert measurements against the expert consensus was 4.4 mm. Intraclass correlation coefficient between AI and expert consensus was 0.926 (95% CI, 0.904–0.944), compared with 0.817 (0.778–0.954) between individual experts and expert consensus. For interventricular septum thickness, precision SD was 1.8 mm for AI (intraclass correlation coefficient, 0.809; 0.729–0.967), versus 2.0 mm for individuals (intraclass correlation coefficient, 0.641; 0.568–0.716). For posterior wall thickness, precision SD was 1.4 mm for AI (intraclass correlation coefficient, 0.535 [95% CI, 0.379–0.661]), versus 2.2 mm for individuals (0.366 [0.288–0.462]). We present all images and annotations. This highlights challenging cases, including poor image quality and tapered ventricles.

**Conclusions:**

Experts at multiple institutions successfully cooperated to build a collaborative AI. This performed as well as individual experts. Future echocardiographic AI research should use a consensus of experts as a reference. Our collaborative welcomes new partners who share our commitment to publish all methods, code, annotations, and results openly.

## Abstract 2

Supplemental Digital Content is available in the text.

The advent of deep learning with neural networks has permitted computers to perform tasks in computer vision that could never have been realistically approached before. This is commonly termed artificial intelligence (AI).

AI has great potential to increase the time-efficiency for quantification when reporting echocardiograms.^1,2^ However, a key challenge to becoming part of the human-led team is that such automation needs to earn the trust of echocardiographers and cardiologists.

Human staff benefit from expert societies delivering education, training, and examinations to maintain high standards.^3^ This facilitates them to deliver measurements that reflect underlying anatomy and physiology and are consistent with historical clinical data. This allows physicians to trust the results for clinical decisions and to be able to interpret data, regardless of when and where it was acquired, in a broadly consistent manner.

AI must be trained and tested to at least the same level of rigor as human experts, for it to have any place in clinical environments.^4^ The approach for humans is to educate them with many examples and validate their performance against standards set by experts. AI should go through a process that is at least as demanding. To achieve this, we established the Unity Collaborative group of echocardiographers and cardiologists, which already includes 17 hospitals across the United Kingdom, to bring together expert representatives to provide this skilled training and validation of AI.

Here, we present the Unity Collaborative labeling system for developing open-access training and validation data for echocardiography and demonstrate its use for making guideline-standard parasternal long axis left ventricular (LV) measurements. The dataset for model development, trained AI, and associated code are made available at the project website (https://data.unityimaging.net).

## Methods

The dataset for model development, trained AI, and associated code are made freely available the project website (https://data.unityimaging.net). This research and release of associated dataset received a Favourable Opinion from the South Central—Oxford C Research Ethics Committee (Integrated Research Application System identifier 279328, 20/SC/0386).

### Image Annotation

**Figure 1.**
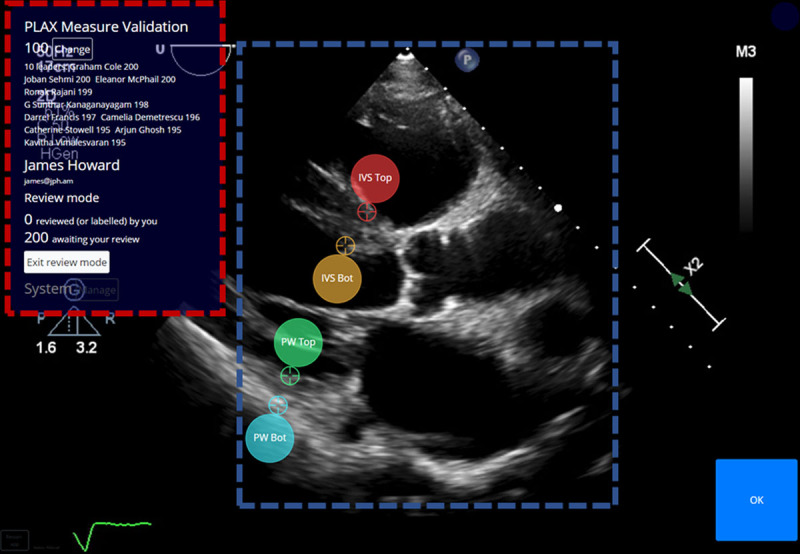
**The unity interface.** The unity interface (www.unityimaging.net) provides an easy-to-use web-based interface to annotate medical images. The system is divided into a labeled area (blue square) and an information area, showing that user’s statistics, compared with those of other users (red square). The 4 keypoints used in this study are highlighted as circles with their names and associated target icons for their exact location. Keypoints on echocardiograms can be labeled either using a touch screen interface or a mouse. The system also allows regions of interests and curves to be annotated (not shown).

We developed an online interface which could be accessed remotely by collaborators (https://data.unityimaging.net). It is a web-based, interactive, real-time platform for efficiently obtaining annotation of medical images (Figure 1). Within the platform, projects can be set up to collect keypoints (also known as landmarks) between which measurements are made.

Onto this platform, we stored 2 datasets of echocardiographic images showing the parasternal long-axis view. One dataset was for training and monitoring the progress of training. The other dataset was for validation: the neural network was not shown any of these images during the training phase.

Each AI and human measurement was calculated using the Euclidian distance between 2 identified keypoints: anterior to posterior septum for septal thickness; posterior septum to endocardial posterior wall for LV internal diameter, and endocardial to epicardial posterior wall for posterior wall thickness.

### Training and Progress-Monitoring Dataset

To train the neural network, we created a training (and progress-monitoring) dataset of echocardiographic images with expert-derived annotations of key points. The images were derived from echocardiograms collected between 2015 and 2016 from 7 laboratories.

The Unity Collaborative experts (see acknowledgements) shared the pooled task of annotating the images, using our online platform. Each image was labeled once, to mark the 4 keypoints required for measuring the LV internal diameter and wall thicknesses in the parasternal long-axis view. An example image with the 4 points identified is shown in Figure 1.

As successive trainings of the neural network were undertaken, we reviewed the outputs and results on the progress monitoring dataset. These results were used to further identify types of images (such as images with a small LV cavity) to target further labeling.

### Validation Dataset

The validation dataset was a fresh set of images, which the AI could never have encountered during training. The images were extracted from 100 consecutive echocardiograms performed over 3 days across the 7 echocardiography laboratories. From each study, the systolic and diastolic frames were extracted from the parasternal long-axis video, to form a dataset of 200 images.

Each image was labeled with the 4 keypoints twice by each of 13 experts, yielding 26 independent evaluations. From these, we derived high quality consensus reference measurements (see Statistical analysis). These experts were BSE-accredited echocardiographers and consultant cardiologists specializing in echocardiography. The images were presented in a random order, and each expert was blinded to any previous labeling by themselves or others. They were encouraged to label every image, unless image quality made it impossible.

### Training the Neural Network

We trained a neural network to annotate the 4 keypoints. For each point, the neural network was trained to produce a heatmap, which was an image with intensity 1 at the exact point of interest, and decayed away in all directions to 0, following a gaussian distribution with SD of 4 pixels.^5^ This approach made it easier for the network to learn, because if the network made an approximately correct proposal, it could not only be partially rewarded, but also be guided toward the correct answer, defined by the direction of steepest gradient up the heatmap. Previous applications of this network (eg, for human pose) have used gaussian distributions with an SD of 1 pixel,^6^ perhaps because it was possible for the human experts to identify those locations very precisely. In echocardiography, an individual expert making an individual assessment cannot consistently select the same pixel, and therefore a network has difficulty learning to match a patch with an SD of only 1 pixel. We found that with an SD of 4 pixels, training was robust.

We derived the coordinates of each predicted keypoint from the peak of the corresponding heatmap. From the 4 keypoints proposed by the network, we could calculate the 4 distances: left ventricular internal dimensions (systole and diastole) and the diastolic thicknesses of the anteroseptum and posterior wall.

The neural network architecture was HigherHRNet W-24,^6^ with an output layer for each of the 4 keypoints. Training images were augmented during each epoch with random affine transformations, random gamma changes, and random erasure of a section of this image. The network was trained for 300 epochs, with an initial learning rate of 0.001 using the RAdam optimizer^7^ and the mean squared error loss function. The learning rate was reduced by a factor of 5 every time the loss on the progress-monitoring dataset plateaued for 20 epochs. If an expert was unable to localize the keypoint on an image (eg, due to very poor image quality), the training process did not train on that key point of that image (by weighting the loss function to 0). The network was trained using four 24 GB Titan RTX graphical processing units (Nvidia Corporation, Santa Clara, California) with a batch size of 24 and an input image size of 608×608 pixels using the PyTorch framework version 1.4.0 and Python version 3.7. Training took ≈ 22 hours.

For inference, a center crop of 640×640 pixels (with zero padding if needed) was fed into the network. The resultant heatmaps were transformed into physical coordinates using the DICOM meta-data, which were extracted using the pydicom package.

During the network implementation and training process, 80% of the images in the training-and-progress-monitoring dataset were used for training the network, and 20% were kept aside for progress monitoring. We ensured that from each echocardiogram video, frames were used for training or for progress monitoring, but never both.

Finally, when the neural network had completed training, its performance was then assessed using the separate validation dataset of 200 images.

### Validation of Neural Network Against Consensus of Experts

The final validation process was necessarily more stringent than the training process. The reference standard against which the network was validated was, for each image, the consensus of the 26 measurements from 13 experts for each of the 4 keypoints on each of the 200 images.

### Statistical Analysis

The validation process recognized that expert opinions will vary for a single image. The reference standard for each measurement was defined for each measurement on each image as the median value of the 13 experts’ individual measurements.

For each measurement on each image, we calculated the signed deviation of the AI measurement with respect to the reference measurement, for example, +2 mm when the AI measured left ventricular internal diameter (LVID) as 41 mm and the consensus of experts was 39 mm. Across all images, we calculated the bias as the mean of these signed deviations, and the precision as their SD. We also calculated the 50th (ie, the median), 80th, 90th, and 95th quantiles of the absolute deviations. All of these measurements were also conducted for each individual expert (still using the consensus of experts as the reference).

This process was also carried out for each of the experts’ measurements treated as an individual: this provided context against which to judge the AI performance. We also calculated the associated intraclass correlation coefficients (ICCs) for these calculations.

The *F*-test was used for comparisons between standard deviations using R’s *var.test* function. Differences in absolute errors were assessed using a Wilcoxon signed-rank test, because of their inherent folded normal distribution. *P*<0.05 was used as the threshold for statistical significance.

Statistical analyses were performed using the R programming language version 3.6.2 using the tidyverse^8^ and irr^9^ packages.

## Results

### Dataset

The training and progress monitoring dataset comprised 2056 images. The validation dataset comprised 200 frames, which are paired end-systolic and end-diastolic frames from 100 separate echocardiography cases. Table 1 describes these videos. For the validation dataset, left ventricular internal diameter averaged 4.7 cm (SD 0.64 cm) in diastole and 3.77 (0.73 cm) in systole, and diastolic thicknesses of the interventricular septum were 1.17 cm (0.24 cm), and of the posterior wall 1.10 cm (0.16 cm).

**Table 1.**
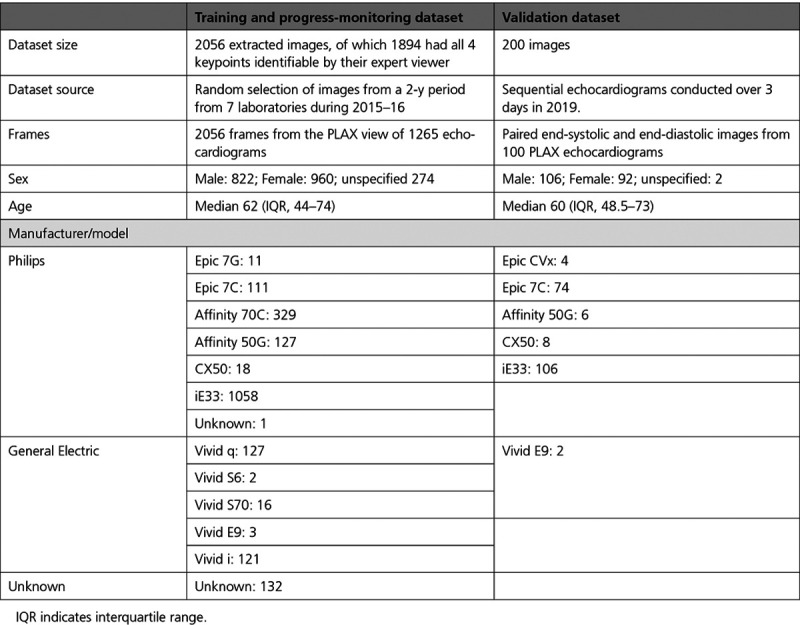
Image Sources

### Results of Training Phase: Precision With a Single Trainer As the Standard

**Figure 2.**
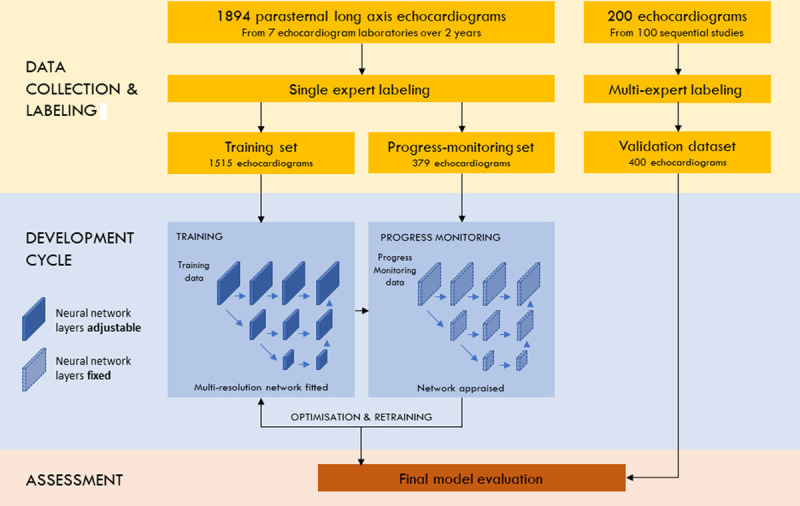
**System pipeline.** A neural network was trained on the training set of 1894 images. One thousand five hundred fifteen of these were used to directly train the network, while 379 were used for progress-monitoring. Finally, we assessed the performance of the network on a new dataset of 200 successive echocardiograms, labeled by 13 experts.

Our collaborative group reviewed the 2056 images in the training and progress-monitoring dataset, with each image annotated by 1 expert from a group of 9. In 1894 of these images, all 4 key points could be annotated. 80% of these annotated images were used directly to fit the network. The remaining 20% were kept aside solely for progress monitoring, which allowed precision of the network to be assessed on images it had not been fitted to (Figure 2). In line with recommendations,^10^ we have called this progress monitoring rather than the conventional AI term of validation, to avoid misunderstanding because to a clinical audience the term validation is generally reserved for a final assessment against a fresh dataset after a model or algorithm has been finalized.

For left ventricular internal dimension, at the end of training, the SD of the difference between the AI measurement and the single expert (precision SD) had fallen to 3.1 mm on the training dataset and 4.5 mm on the progress-monitoring dataset with minimal bias (−0.2 and −0.1 mm, respectively, Table 2).

**Table 2.**
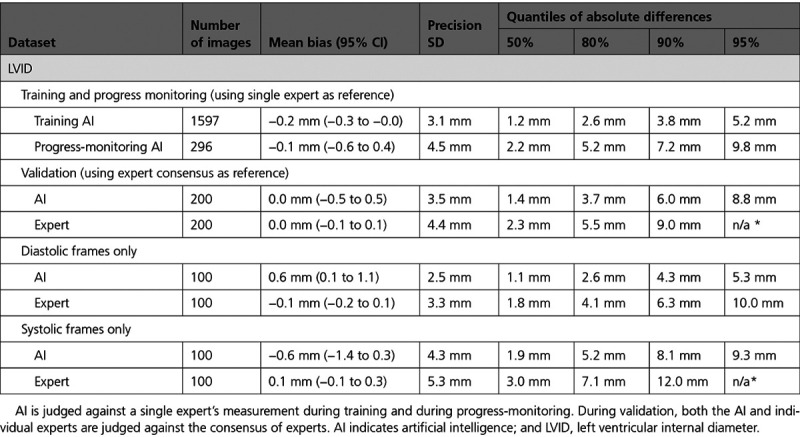
Performance in Measuring Left Ventricular Internal Dimension

For septal wall thickness, the precision SD had fallen to 2.5 mm for the training and 2.2 mm for the progress-monitoring dataset (Table 3). For the posterior wall, these values were 2.3 and 2.9 mmm, respectively (Table 3).

**Table 3.**
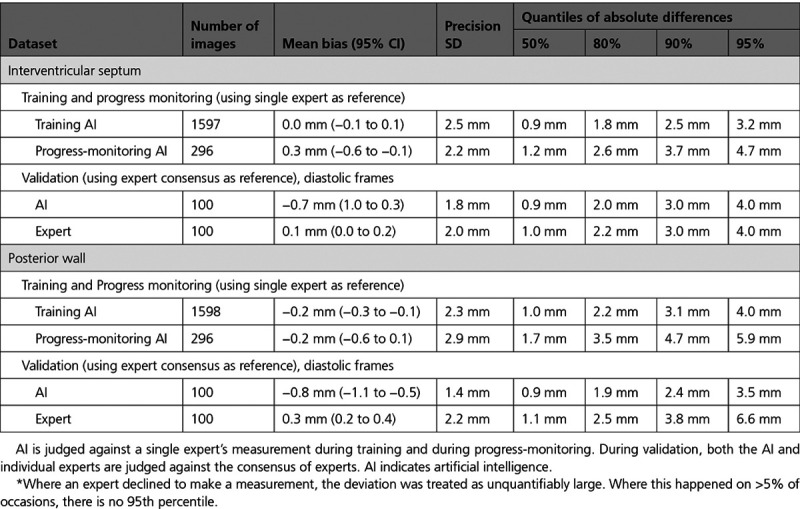
Performance in Measuring Interventricular Septum and Posterior Wall Dimensions

### Results of Validation Phase: Precision With the Consensus of Experts As the Standard

The 200 new images of the validation phase were each labeled by 13 experts (9 original + 4 new), twice in a random order. Each image therefore had 26 opinions. In this set, we defined the expert consensus reference standard for the correct dimension as the median value of the individual experts’ median opinion. We could therefore calculate the error in the dimension measured by the AI, as well as the errors in the dimensions reported by the individual expert opinions as compared with the expert consensus reference value.

The AI measured LV dimension with precision SD of 3.5 mm. Notably, this was smaller than that it delivered during progress monitoring of training (4.5 mm, *P*=0.0002). The corresponding ICC was 0.926 (95% CI, 0.904–0.944). Individual expert opinions matched the expert consensus with a precision SD of 4.4 mm, with an ICC of 0.817 (95% CI, 0.778–0.954).

The precision SDs were significantly smaller in diastole than in systole. For the AI, it was 2.5 mm in diastole versus 4.3 mm in systole (*P*<0.0001). For experts, it was 3.3 mm in diastole versus 5.3 mm in systole (*P*<0.0001).

For septum thickness, the AI delivered a precision SD of 1.8 mm (ICC, 0.809 [95% CI, 0.729–0.967]), and the individual experts 2.0 mm (ICC, 0.641 [95% CI, 0.568–0.716]). For posterior wall thickness, the AI had precision SD 1.4 mm (ICC, 0.535 [95% CI, 0.379–0.661]), and the individual experts 2.2 mm (ICC, 0.366 [95% CI, 0.288–0.462]).

### Visualizing AI Performance in the Context of Individual Expert Measurements

**Figure 3.**
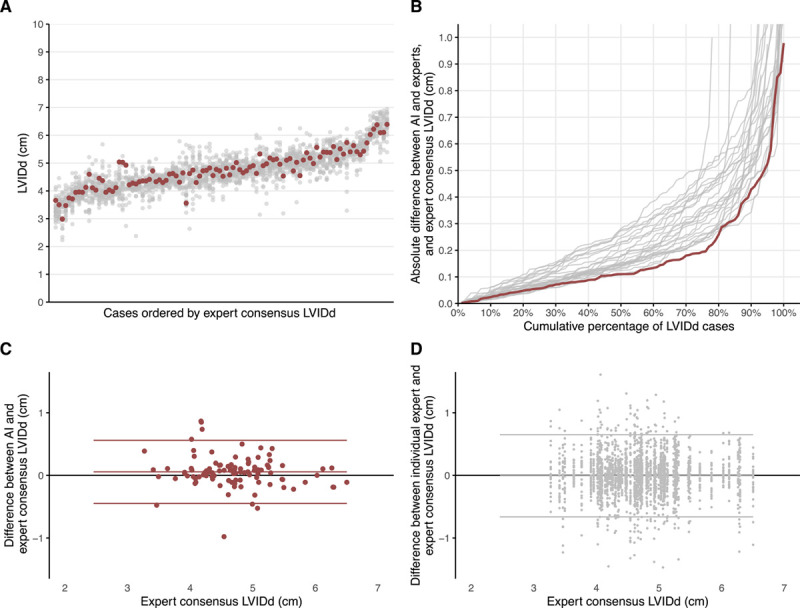
**Artificial intelligence (AI) performance in the context of individual expert measurements (diastolic left ventricular internal diameter [LVIDd]).** **A**, Shows measurements by the AI (red dots) in the context of the individual expert measurements (gray dots) for all 100 validation images, arranged in order of increasing ventricular dimension (defined by expert consensus). **B**, Shows the cumulative distribution of deviations from expert consensus, for the AI in red and the individual experts in gray. The lower panels show deviation from the expert consensus for AI (**C**), and the experts (**D**) with each panel showing the 95% limits of agreement (horizontal lines).

A simple visual summary of the AI measurements in the context of expert performance is given in Figure 3 for left ventricular internal dimension in diastole. Figure 3A shows that for each image, the AI generally reports a value near the middle of the spread of individual expert measurements. In Figure 3B, each expert viewing (2 viewings per expert) is represented by a separate gray curve, and the AI by a red curve. The curves show the distribution of magnitudes of deviation of the measurements from the consensus measurement. For example, for the expert represented by the lowest gray curve (ie, the expert whose measurements were generally closest to the expert consensus), the curve passes through the point (50%, 1.1 mm), which means that their median error was 0.11 cm. Similarly, it passes through (80%, 2.6 mm), which means that 80% of measurements were within 2.6 mm of expert consensus. For the LVID in diastole, the individual experts had a median error ranging from 1.1 to 2.8 mm, and the AI had a median error of 1.1 mm. Sizes of these errors are shown in Table 2. The lower panels show the deviation of the AI (Figure 3C) and single expert measurements (Figure 3D) against the expert consensus measurement as reference.

Corresponding displays for the wall thicknesses and left ventricular systolic dimension are given in Figures I through III in the Data Supplement.

### Accuracy of Dimensions Versus Keypoint Localization

The AI was better at matching the expert consensus of the dimension of the LV than it was at choosing key point locations that matched expert consensus, although it was specifically trained to succeed at the latter task rather than the former. For example, in the progress-monitoring dataset, the AI’s absolute error for the LVID dimension was smaller than both the absolute error in position of the septal endocardial point and the posterior wall endocardial point (median absolute error, 2.2 versus 3.1 and 5.8 mm, *P*=0.0006 and *P*<0.0001 respectively).

**Figure 4.**
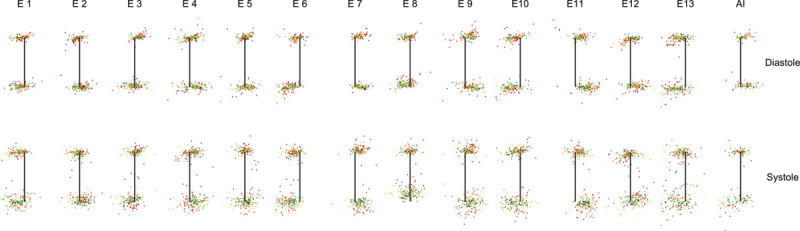
**Positions chosen by artificial intelligence (AI) and individual experts for the keypoints of LV dimension, plotted in relation to the expert consensus.** The top right panel shows, for each of the 100 diastolic images in the validation dataset, the LV dimension keypoint locations chosen by the AI (coloured dots), in relation to the expert consensus keypoint locations (black line), after reorienting and rescaling so that the expert consensus LV dimension line is vertical and length 1 unit. This shows the error in the AI’s placement of keypoints is largely longitudinal along the ventricle (horizontal on the plot). Dot colors range from green (cases with the smallest variation between experts) to red (largest variation). The remaining plots display the corresponding information for individual experts (E1 to E13) and for systole (lower row). Corresponding plots are shown in the Appendix for the septum (Figure IV in the Data Supplement) and posterior wall (Figure V in the Data Supplement).

This was because the neural network often made measurements at a different longitudinal position along the ventricle than the expert consensus but nevertheless correctly drew the dimension transversely across the ventricle and therefore obtained an acceptable measurement. The reason for this is best seen in the validation dataset, because there are multiple expert opinions. It emerges that, just like the AI, different experts also choose different keypoints for measuring LV dimension. Figure 4 displays this phenomenon in a standardized manner. For each image, we re-expressed the deviation of keypoint locations given by individual experts (E1 to E13) and the AI, relative to the size and orientation of the consensus measurement line for the LVID. This is equivalent to rotating and resizing the image so that the consensus measurement line for the LVID is vertical, and its length is 1 arbitrary unit. This allowed the error in the position of each point to be expressed as 2 components. One is in line with the direction of LVID measurement, that is, vertical on the rotated image, which we term transverse. The other is perpendicular to this direction, that is, horizontal on the rotated image, which we term longitudinal, expressed as a percentage of the 1 arbitrary unit.

The longitudinal variability was larger than the transverse for the AI (SD 15% versus 7%, *P*<0.0001). This was true both in diastole (13% versus 4%, *P*<0.0001) and in systole (14% versus 9%, *P*<0.0001). All the errors are shown in Figure 4. The corresponding plots for the septum and posterior wall are shown in Figures IV and V in the Data Supplement.

The errors in the posterior point were larger than those in the anterior point. This was true both for the AI (longitudinal 18% versus 10%, *P*<0.0001; transverse 7% versus 6%, *P*=0.0011) and the individual expert opinions (longitudinal 26% versus 20%, *P*<0.0001; transverse 12% versus 11%, *P*<0.0001).

## Discussion

This study has shown that imaging specialists representing a nation’s expertise can collaborate through a distributed online system to provide both training data and separate multiobserver validation dataset. A neural network can then be trained, and its performance judged using the multiple expert opinions in 2 ways: their consensus as the reference standard and their individual variation from consensus as the acceptable range in contemporary expert performance. AI performance for making guideline standard left ventricular measurements^11^ from the parasternal long-axis is good, on par with human experts, and is challenged by the cases that human experts find challenging.

### Capturing Multiple Mutually Blinded Expert Opinions

There are now many well-established neural network architectures for image processing.^12^ The bottleneck for an applicable echocardiographic AI is no longer the development of neural network architecture, nor the availability of vast image datasets,^13^ but rather expert annotations a clear provenance.

Many early AI tasks were classifying objects into simple categories, such as cats versus houses versus trees.^14^ The correct answer is generally unambiguous and obvious to any human. It is, therefore, reasonable and efficient to store a single correct answer for each image, and to aim for the AI to match that answer. The pioneering work in echocardiography AI^1,2^ also took this approach of defining the reference standard as a single opinion from a single expert. If the accuracy found in such a manner is imperfect, it is not possible to know whether this is (1) a failure of the AI, (2) a bias in the chosen expert, such as consistently over-estimating a cavity dimension, or (3) ambiguity within the image which allows an expert to give different opinions on separate viewings.

In echocardiography, experts can have different opinions on the ideal positions for keypoints. Collecting multiple, mutually blinded expert opinions gives 2 advantages. First, their consensus will be less noisy and therefore a better reference standard. Second, the variation between the opinions provides crucial context about the acceptable range of answers.

Consensus of experts has a pedigree as gold-standard in challenging tasks. Classifying retinal photographs was performed by up to 7 ophthalmologists in study of AI for assessing retinal images.^15^ The variation we observed between experts (eg, 5.3 mm for LVIDs) in our study suggests that such an approach is wise in cardiac imaging too.

Our network architecture was HigherHRNet, which maintains high-resolution representations through multiscale fusions across the convolutional layers.^6^ Previous work has used various other designs, typically adaptations of U-Net.^16^

An unusual feature of our study is that we mirrored the clinical approach^11^ of identifying keypoints as the primary network target, rather than segmenting areas and then defining keypoints based on those areas. This approach focuses the training process on aspects clinicians consider important for diagnostic measurements.

The other advantage of directly training a network to identify the keypoints is that it automatically chooses the longitudinal position along the length of the myocardium at which to measure the wall thickness and cavity dimension. The alternative, used by others, is to write an explicit algorithm to extract a dimension from a pair of outlines.^17^

Our study used an in-house designed web-based labeling platform to acquire expert opinions on the positioning of keypoints. Other workers have taken the approach of using the keypoints and tracings that were performed by the sonographer at the time of the scan.^1^ Although our method required considerably more effort, it ensured that each piece of training data was reviewed by another expert to ensure there were no accidental labeling errors. More importantly, for the validation set, it allowed us to capture the 26 mutually blinded opinions on each keypoint, so that the performance of the network could be judged in a representative context.

While other AI labeling platforms exist,^18,19^ unity offers certain unique advantages, including support for curve annotation (eg, of cardiac chamber walls) using splines, AI assisted-labeling techniques, and real-time project-specific leader boards to provide gamification and feedback to annotators. There were also certain ethical advantages by being self-hostable, reducing inter-institutional data transfer concerns early in development.

### A Focus on the Challenges

**Figure 5.**
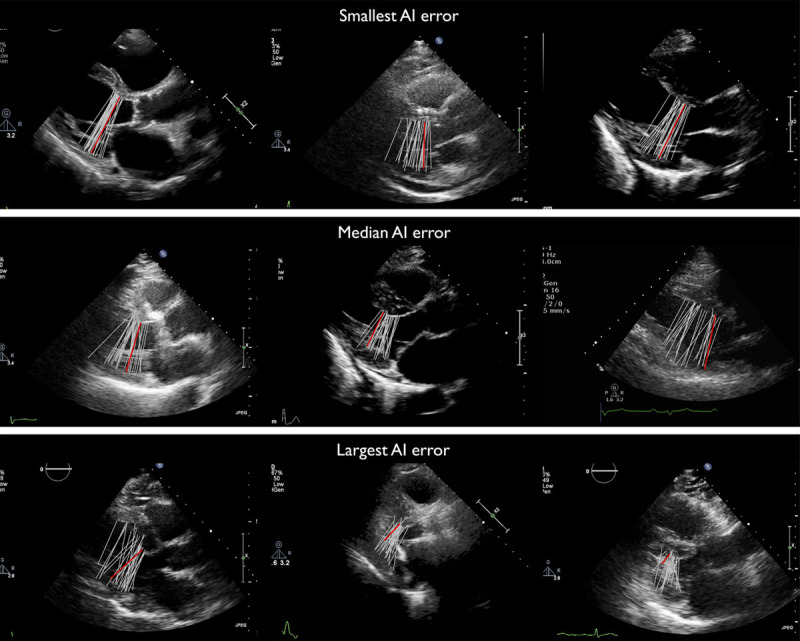
**Nine examples of artificial intelligence (AI) measurements of left ventricular (LV) dimension, drawn from 200 frames, showing the range of AI performance, with expert consensus as the reference standard.** Top: the 3 cases with the smallest AI error, Bottom: the 3 cases with the largest AI error. Middle: median cases when ranked by size of AI error, that is, showing typical performance. In each panel, AI measurements are in red, and 2×13=26 expert measurements in gray.

Some images remain difficult. Figure 5 shows the 3 worst cases of discrepancy between AI measurement and expert consensus, as well as the median case and the best cases. In each case, we show the individual expert measurements too, as context. In the Appendix, we show all measurements, ordered by the deviation of the AI measurement from the group consensus (left ventricular internal diameter in Figure VI in the Data Supplement, diastolic interventricular septal diameter in Figure VII in the Data Supplement, and diastolic posterior wall diameter in Figure VIII in the Data Supplement). Average errors in AI are now very low (our AI delivers a median error for diastolic LVID of 1.1 mm).

Future development would be most fruitfully focused on the types of cases that currently give the largest error. The largest errors occur when image quality is poor. As can be seen, these images are also challenging for experts.

A particular challenge for the AI was discriminating the posterior wall endocardium from the mitral valve apparatus (see bottom row, Figure 5). Because human experts also find this difficult, this aspect requires particular attention in future work.

More generally, the AI’s error in measuring LV dimension was larger in systole than diastole. This may be because the thickened posterior wall infringes even more closely on the mitral valve apparatus.

### Study Limitations

The main limitation of this study is that it used single image frames and not video loops. In clinical practice, clinicians sometimes scan back and forth a few frames, to allow themselves to better identify the structures.

In the validation dataset, the end-systolic and end-diastolic frames were preselected for showing to the AI and the experts. This was so that concordance between experts was not disadvantaged by the possibility that they may select different frames. In an ultimate clinical deployment, there are many possible methods of the system automatically selecting the end-diastolic and end-systolic frames. One possibility is for the AI to measure every frame and then use the maximum and minimum values appropriately.

Because we defined the correct answer as the expert consensus, which contained the opinion of all the human experts (but not the AI), the individual experts had a small advantage in that the expert consensus is slightly biased towards their opinion. However, the effect is small, because of the dilutional effect of the other 13 experts. Moreover, this slight disadvantage for the AI did not prevent it from performing satisfactorily.

There was significant variability in the keypoint locations selected by the experts (Figure 5). However, the majority of the variability occurred parallel to the long-axis of the left ventricle (Figure 4). Consequently, the variability between experts in the measured dimension was much less than the variability in keypoint location. The variability seen between experts in this study (LVID, precision SD 3.3 mm, median absolute deviation 1.8 mm) is comparable to that seen in historic^20^ (precision SD 2.1 mm) and contemporary^21^ (mean absolute deviation 2.5 mm) studies.

An AI system is not an accredited expert and is not a replacement for one. We envisaged that it could be used as a support tool in training programs or to assist offline quality control schemes, for example, feeding back to individuals who might be tending to over- or under-read. If applied in clinical practice, an AI might propose positions for key points, which are then confirmed or edited by staff. In research practice, it may have a role in reducing the cost of analyzing large numbers of images.

## Conclusions

The rate limiting step for creating AIs acceptable to clinicians is no longer the design of more complex neural networks but rather the acquisition of appropriately qualified expert opinions with which to train the network.

Validating an AI against a consensus of experts has 2 advantages. First, the consensus has smaller noise than a single expert’s opinion. Second, the variation between individual expert opinions provides a context to what level accuracy acceptable for an AI.

Using this approach to development and validation, the AI was able to make measurement with a precision SD for LVIDd of 2.5 mm, which is well within the range of acceptability for a human expert.

## Acknowledgments

In addition to authors of this article, the following other members of the Unity Collaborative contributed their expert annotations of key points for the Training and Progress-Monitoring dataset: Barts Health NHS Trust: Guy Lloyd, Sanjeev Bhattacharyya; Bedfordshire Hospitals NHS Foundation Trust: Maysaa Alzetani; Cambridge University Hospitals NHS Foundation Trust: Jason Tarkin, Sharon Wilson; Cardiff and Vale University Health Board: Navroz Masani; East Cheshire NHS Trust: Robin Edgell; NHS Greater Glasgow and Clyde: Colin Berry, Kenneth Mangion; Homerton University Hospital Foundation Trust: Camelia Demetrescu, Waleed Arshad; Imperial College Healthcare NHS Trust: Nina Bual; King’s College Hospital NHS Foundation Trust: Mark Monaghan; Liverpool University Hospitals NHS Foundation Trust: David Oxborough, Vishal Sharma; NHS Orkney: Kevin Fox; Oxford University Hospitals NHS Foundation Trust: Jim Newton; Sheffield Teaching Hospitals NHS Foundation Trust: Abdallah Al-Mohammad. We also acknowledge the National Institute for Health Research (NIHR) Biomedical Research Centre based at Imperial College Healthcare NHS Trust and Imperial College London, and thank them for their financial support (P81137). The views expressed are those of the author(s) and not necessarily those of the NHS, the NIHR or the Department of Health.

## Sources of Funding

Dr Howard is supported by the Wellcome Trust (212183/Z/18/Z).

## Disclosures

Dr Rajani received speaker fees from Siemens Healthcare and GE Medical and has provided consultancy to Medtronic and Edwards Lifesciences. The other authors report no conflicts.

## Supplemental Materials

Supplemental Figures I–VI

## Supplementary Material


