# Supplementary material for: Automated Left Ventricular Dimension Assessment Using Artificial Intelligence Developed and Validated by a UK-Wide Collaborative
Source: Circ Cardiovasc Imaging. 2021 May 17;14(5):e011951. doi: 10.1161/CIRCIMAGING.120.011951 (PMC8136463; doi:10.1161/CIRCIMAGING.120.011951)
Supplement: Supplementary file 1 [file hci-14-e011951-s001.pdf]

SUPPLEMENTAL MATERIAL

Supplemental Figures and Figure Legends

Supplemental Figure I

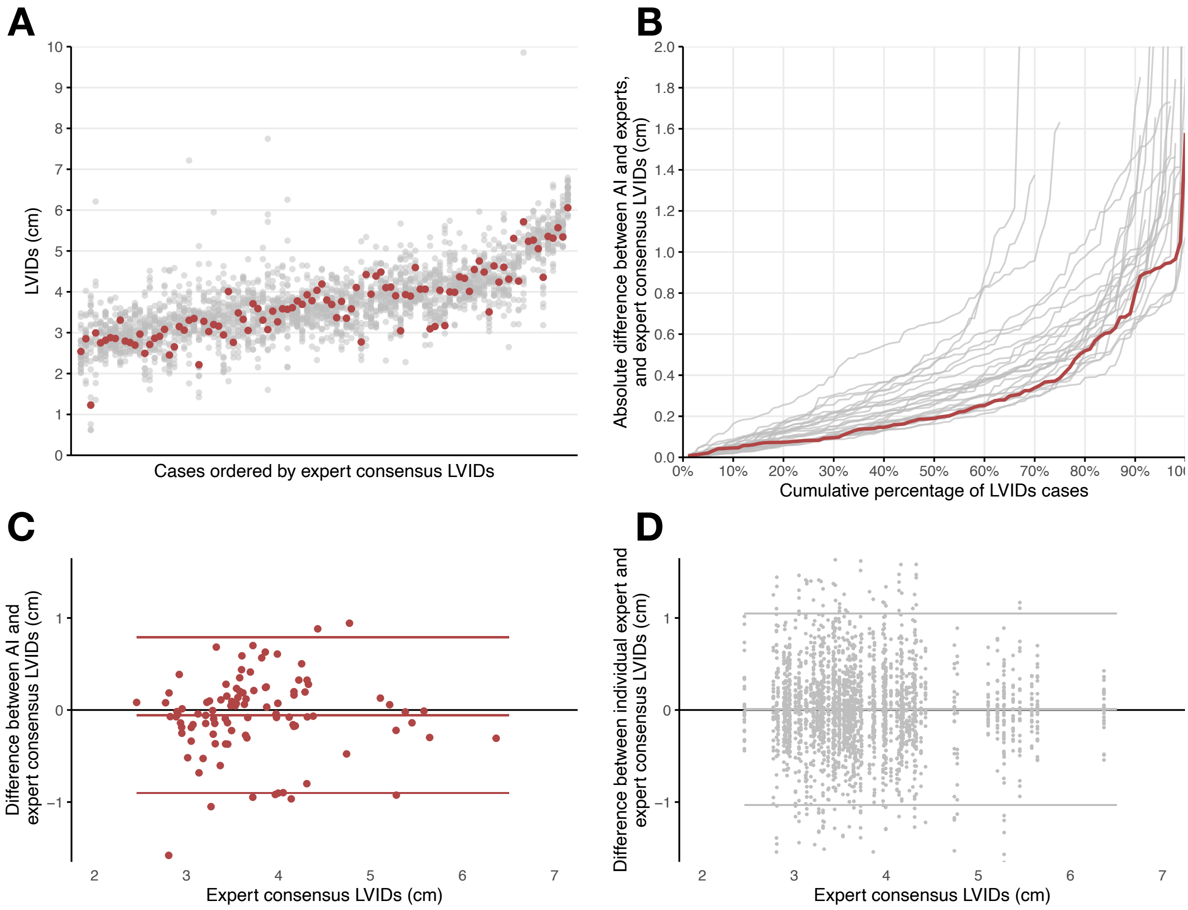

AI performance in the context of individual expert measurements (LVIDs)

Panel A shows measurements by the AI (red dots) in the context of the individual expert measurements (grey dots) for all 100 validation images, arranged in order of increasing ventricular dimension (defined by expert consensus). Panel B shows the cumulative distribution of deviations from expert consensus, for the AI in red and the individual experts in grey. The lower panels show deviation from the expert consensus for AI (Panel C), and the experts (Panel D) with each panel showing the 95% Limits of Agreement (horizontal lines).

Supplemental Figure II

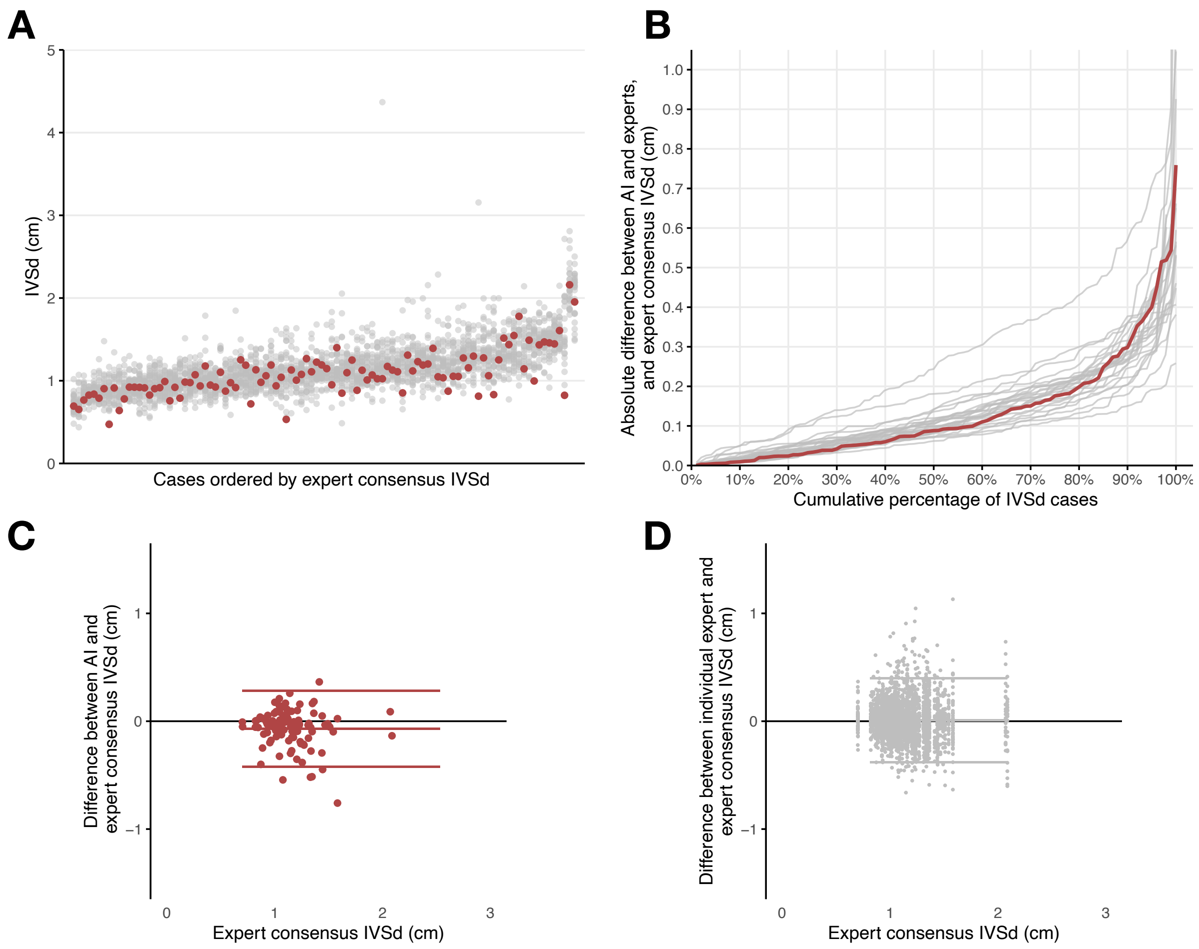

AI performance in the context of individual expert measurements (IVSd)

Panel A shows measurements by the AI (red dots) in the context of the individual expert measurements (grey dots) for all 100 validation images, arranged in order of increasing ventricular dimension (defined by expert consensus). Panel B shows the cumulative distribution of deviations from expert consensus, for the AI in red and the individual experts in grey. The lower panels show deviation from the expert consensus for AI (Panel C), and the experts (Panel D) with each panel showing the 95% Limits of Agreement (horizontal lines).

Supplemental Figure III

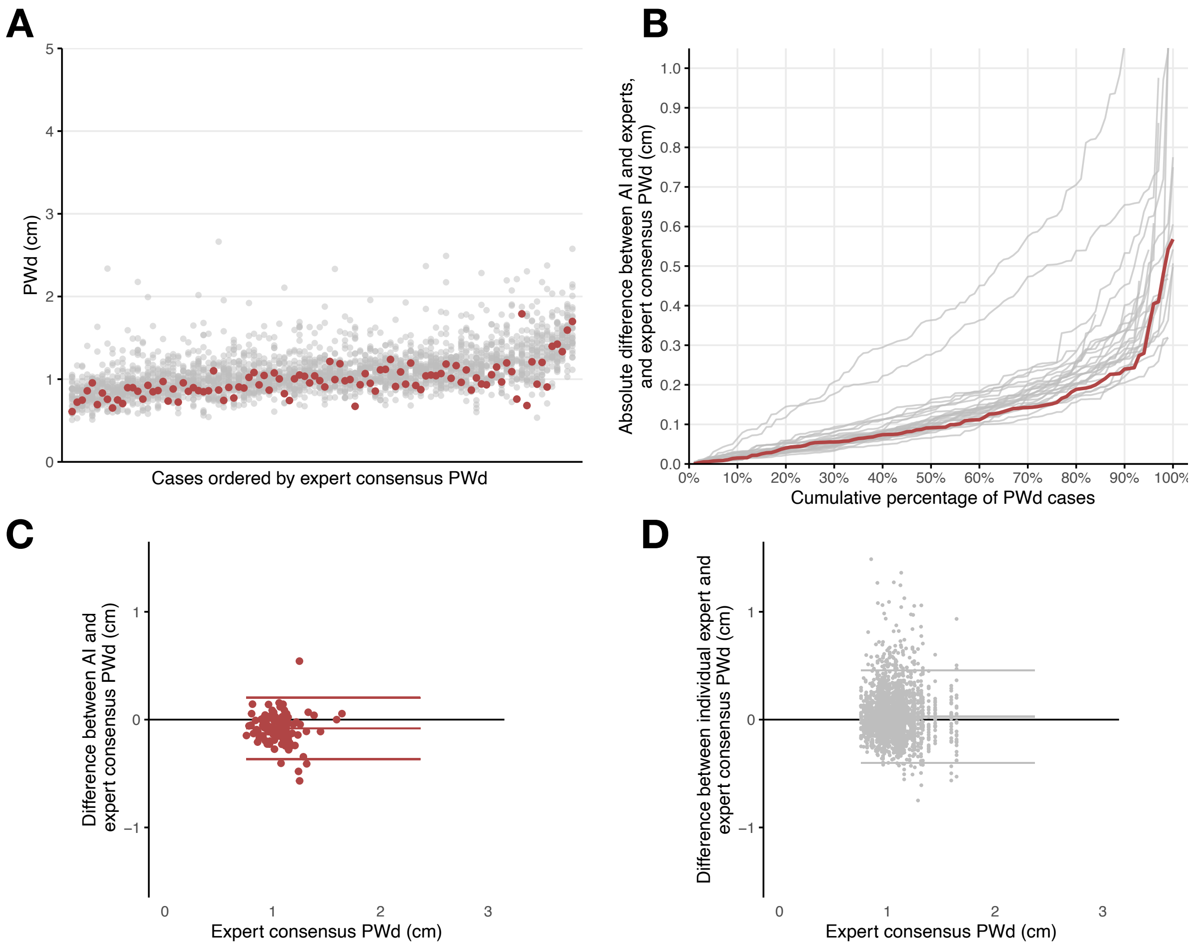

AI performance in the context of individual expert measurements (PWd)

Panel A shows measurements by the AI (red dots) in the context of the individual expert measurements (grey dots) for all 100 validation images, arranged in order of increasing ventricular dimension (defined by expert consensus). Panel B shows the cumulative distribution of deviations from expert consensus, for the AI in red and the individual experts in grey. The lower panels show deviation from the expert consensus for AI (Panel C), and the experts (Panel D) with each panel showing the 95% Limits of Agreement (horizontal lines).

Supplemental Figure IV

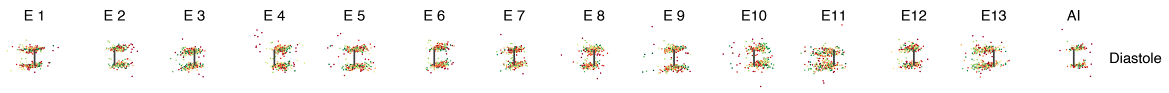

Positions chosen by AI and individual experts for the keypoints of IVSd, plotted in relation to the expert consensus

The top right panel shows, for each of the 100 diastolic images in the validation dataset, the IVSd keypoint locations chosen by the AI (coloured dots), in relation to the expert consensus keypoint locations (black line), after reorienting and rescaling so that the expert consensus IVSd line is vertical and length 1 unit. This shows the error in the AI's placement of keypoints is largely longitudinal along the ventricle (horizontal on the plot). Dot colours range from green (cases with the smallest variation between experts) to red (largest variation). The remaining plots display the corresponding information for individual experts (E1 to E13) and for systole (lower row).

Supplemental Figure V

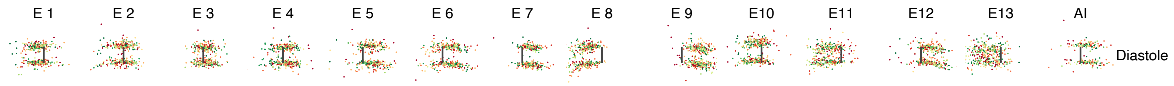

Positions chosen by AI and individual experts for the keypoints of PWd, plotted in relation to the expert consensus

The top right panel shows, for each of the 100 diastolic images in the validation dataset, the PWd keypoint locations chosen by the AI (coloured dots), in relation to the expert consensus keypoint locations (black line), after reorienting and rescaling so that the expert consensus PWd line is vertical and length 1 unit. This shows the error in the AI's placement of keypoints is largely longitudinal along the ventricle (horizontal on the plot). Dot colours range from green (cases with the smallest variation between experts) to red (largest variation). The remaining plots display the corresponding information for individual experts (E1 to E13) and for systole (lower row).

Supplemental Figure VI: Full disclosure LVID measurement dataset

The following 200 images show the AI (red) and expert (grey) left ventricular internal diameter (LVID) measurements on the end-diastolic and end-systolic frames of the 100 validation images. The images are ordered by the absolute difference between AI and expert consensus LVID measurement.

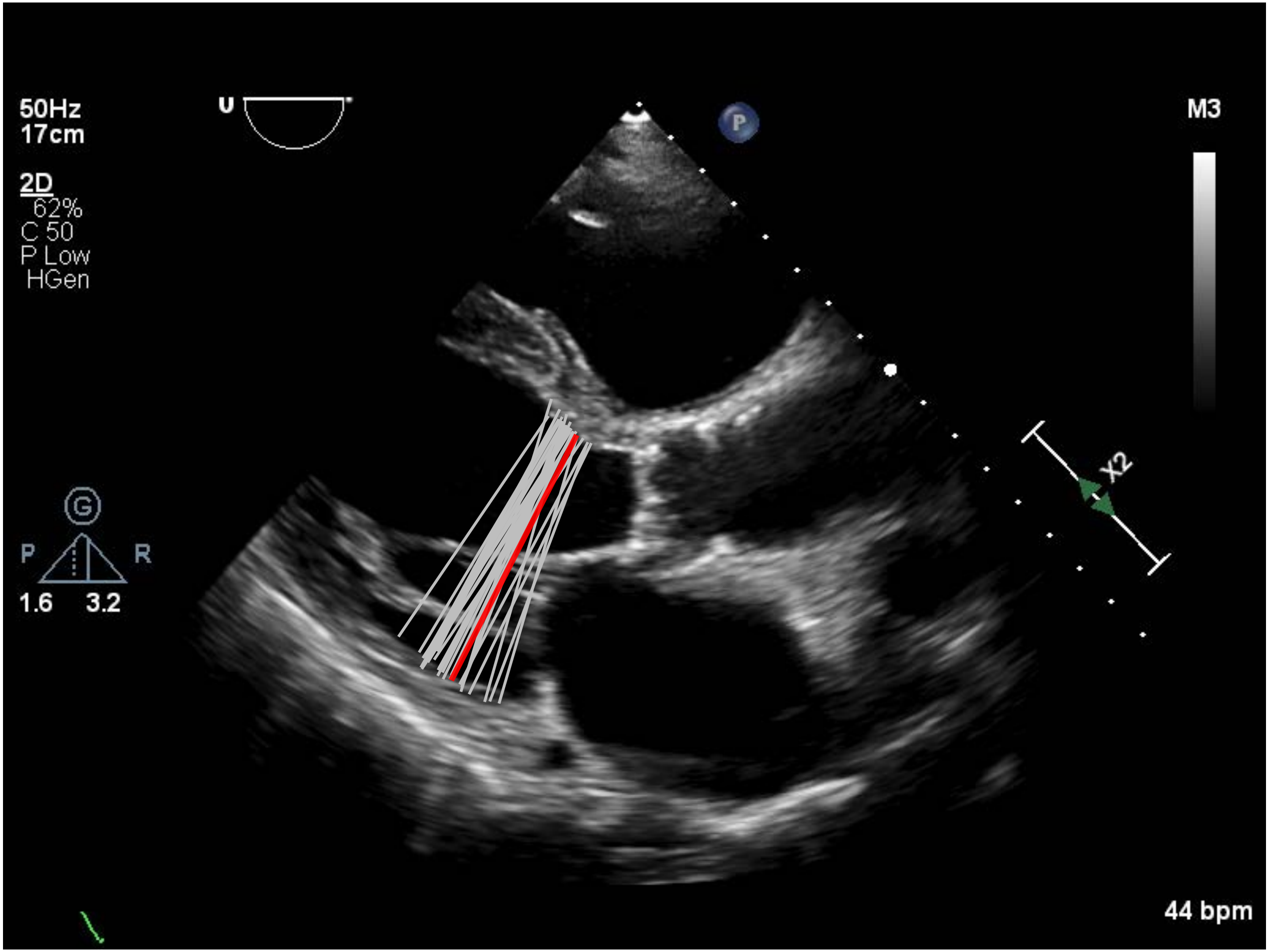

Pt 52 Diastole

Absolute difference between AI and expert consensus: 0.0085 mm

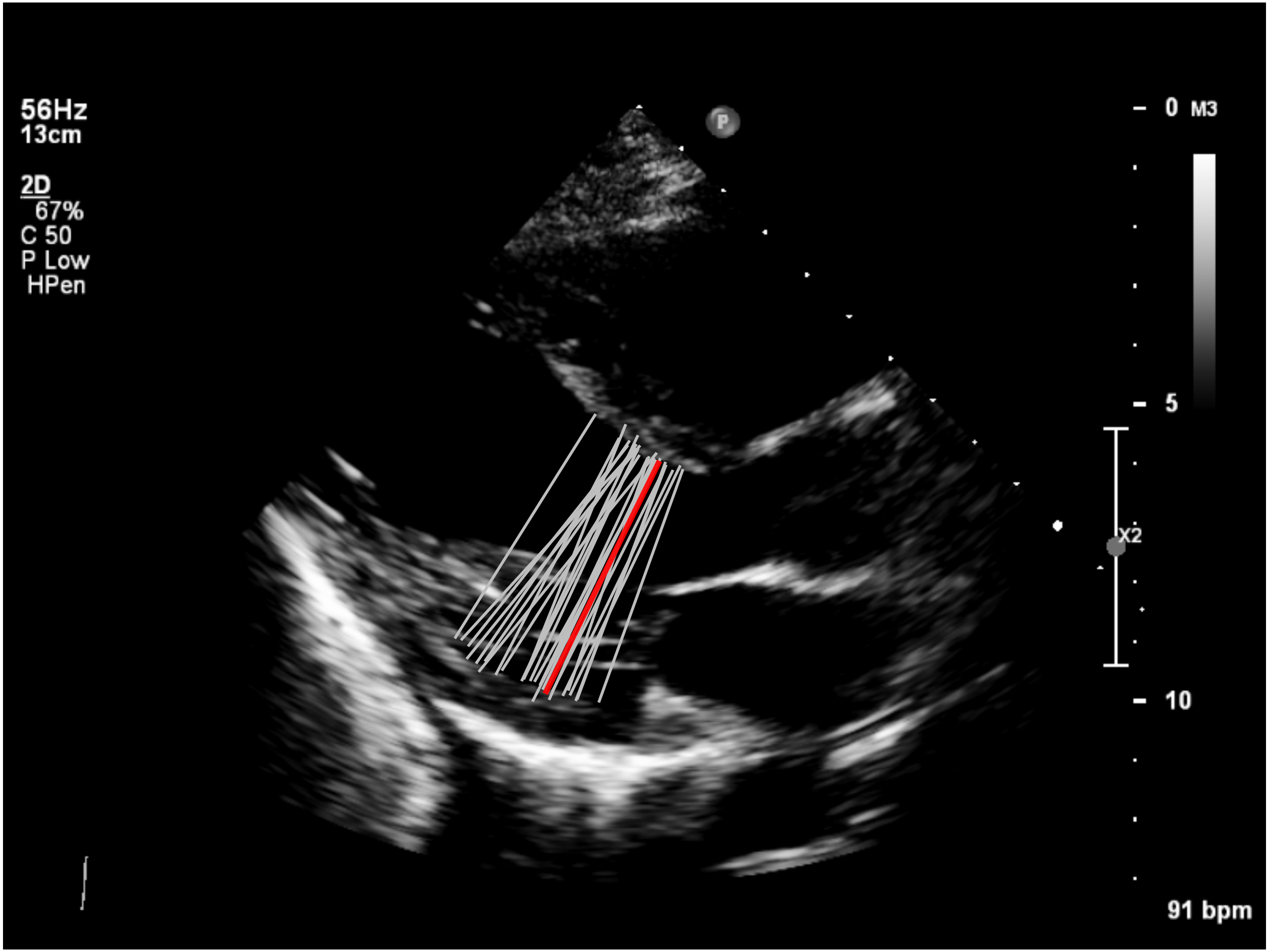

Pt 54 Diastole

Absolute difference between AI and expert consensus: 0.037 mm

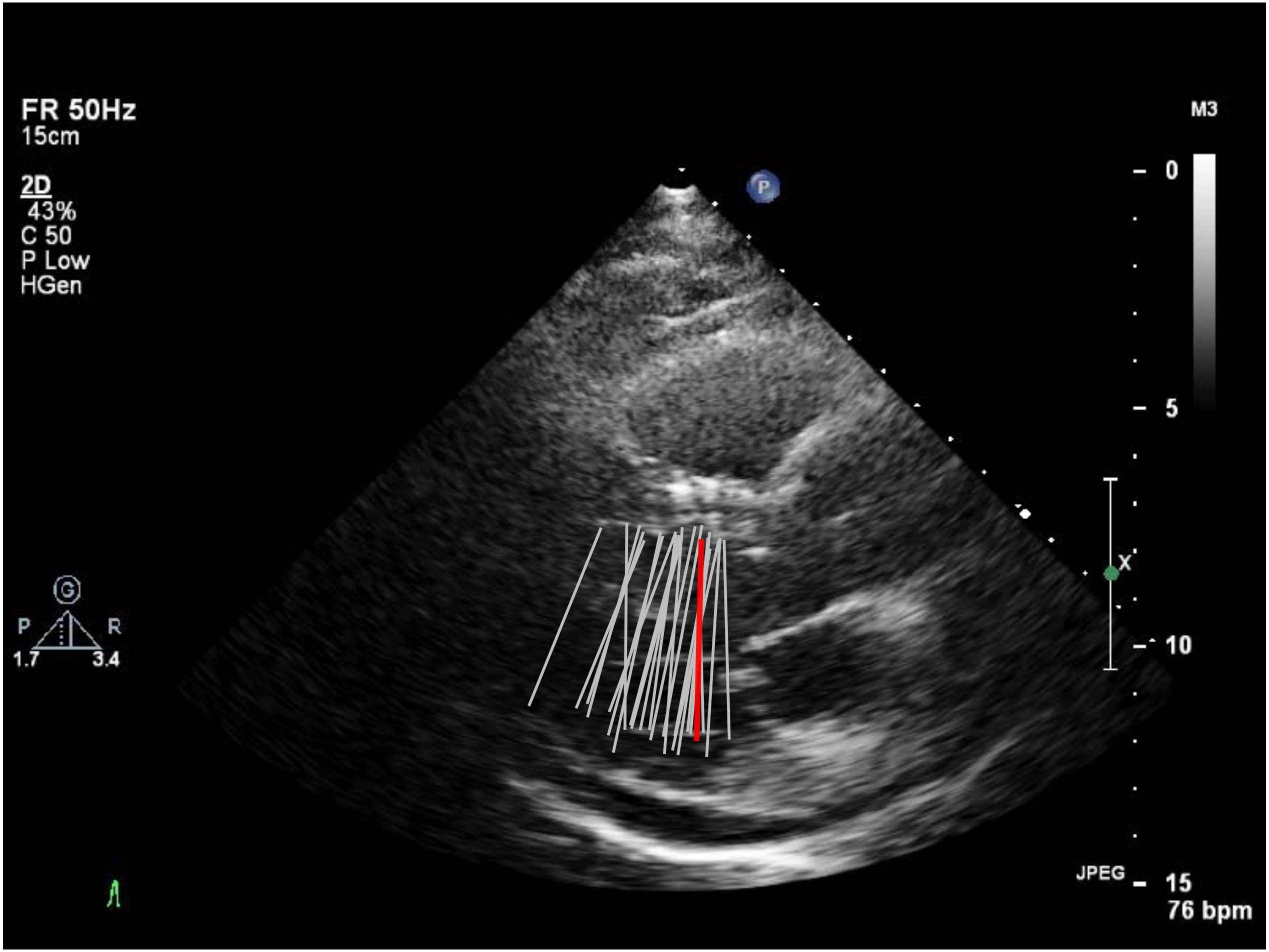

Pt 1 Diastole

Absolute difference between AI and expert consensus: 0.056 mm

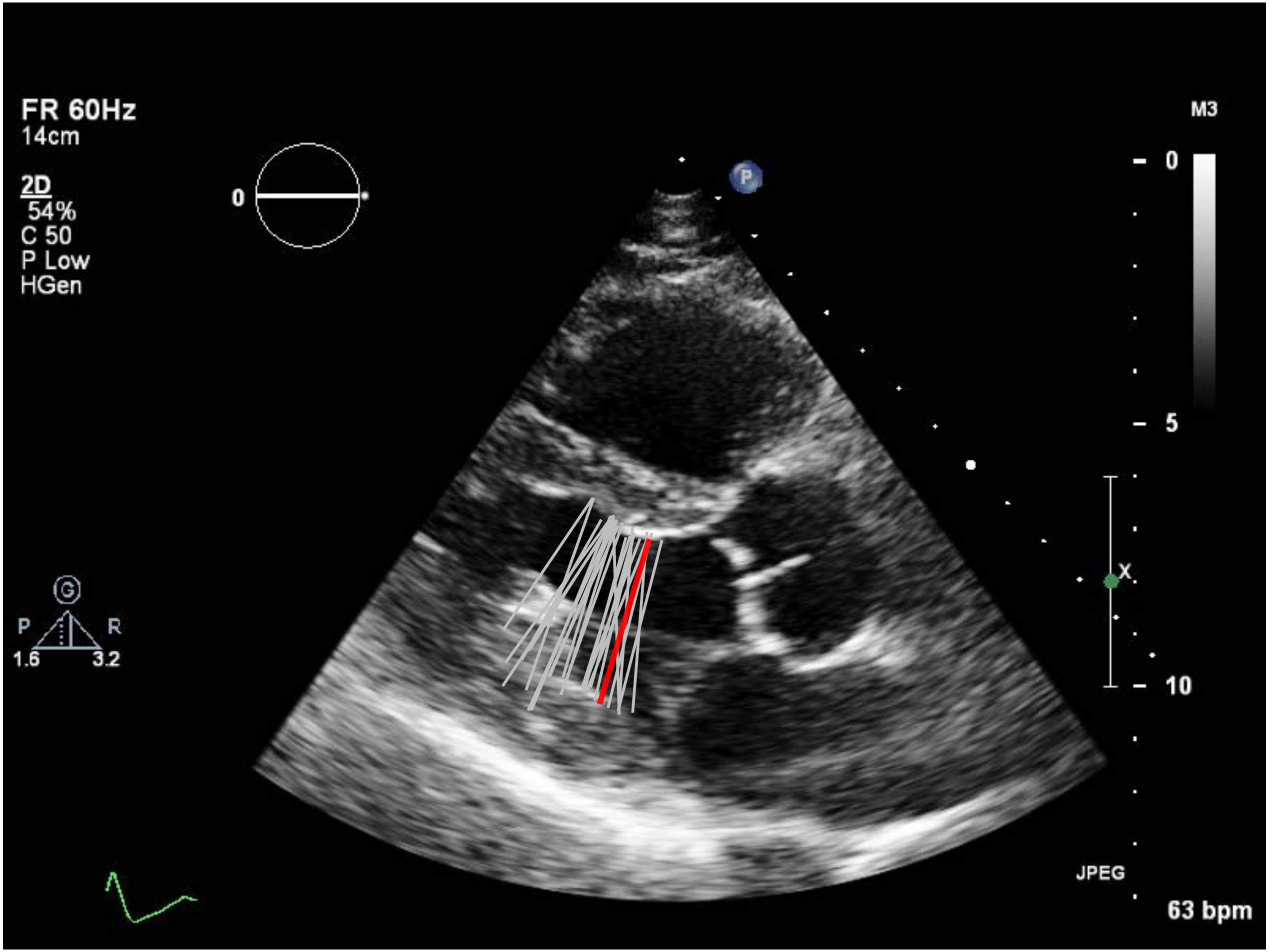

Pt 10 Systole

Absolute difference between AI and expert consensus: 0.057 mm

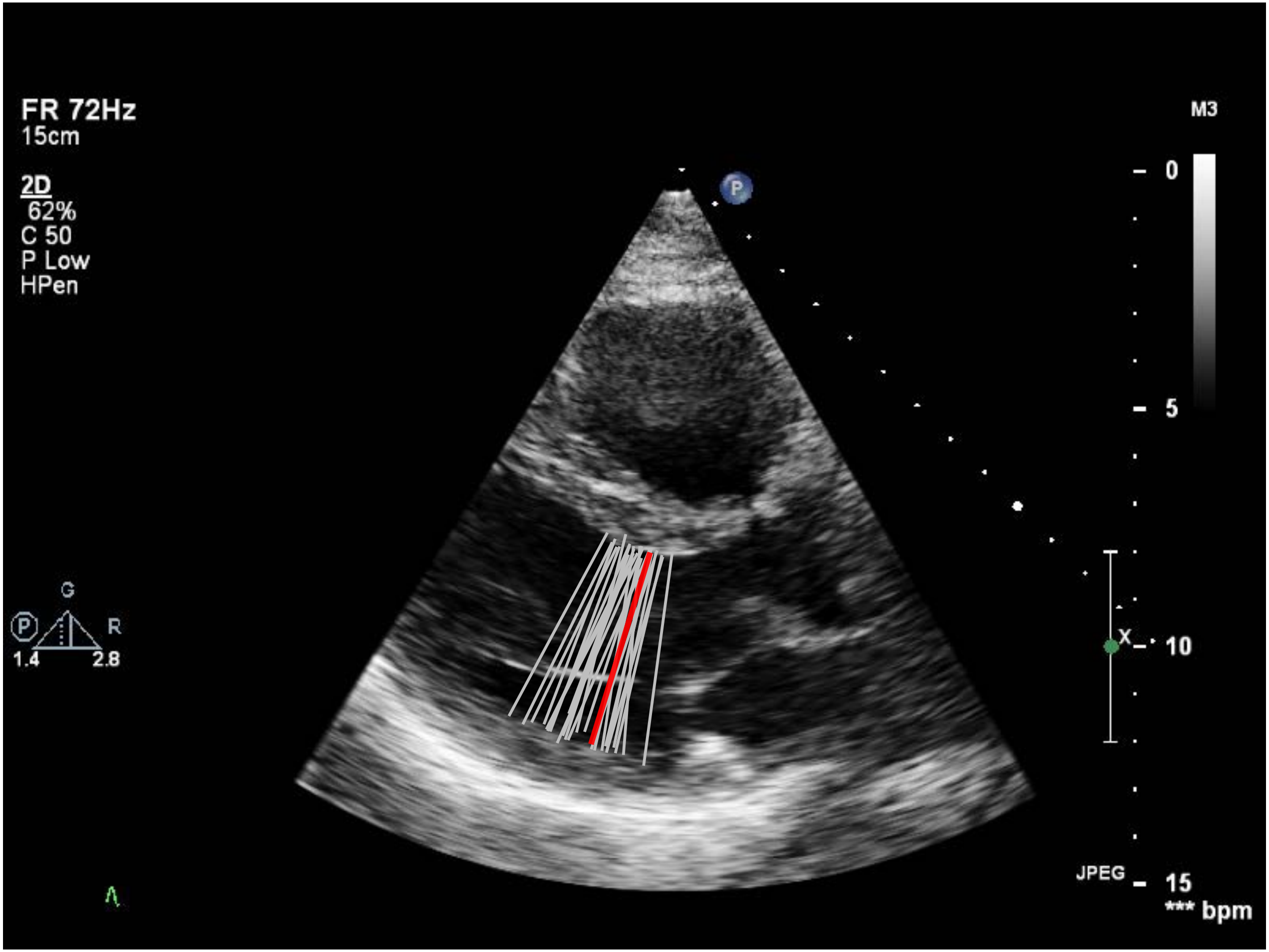

Pt 94 Diastole

Absolute difference between AI and expert consensus: 0.083 mm

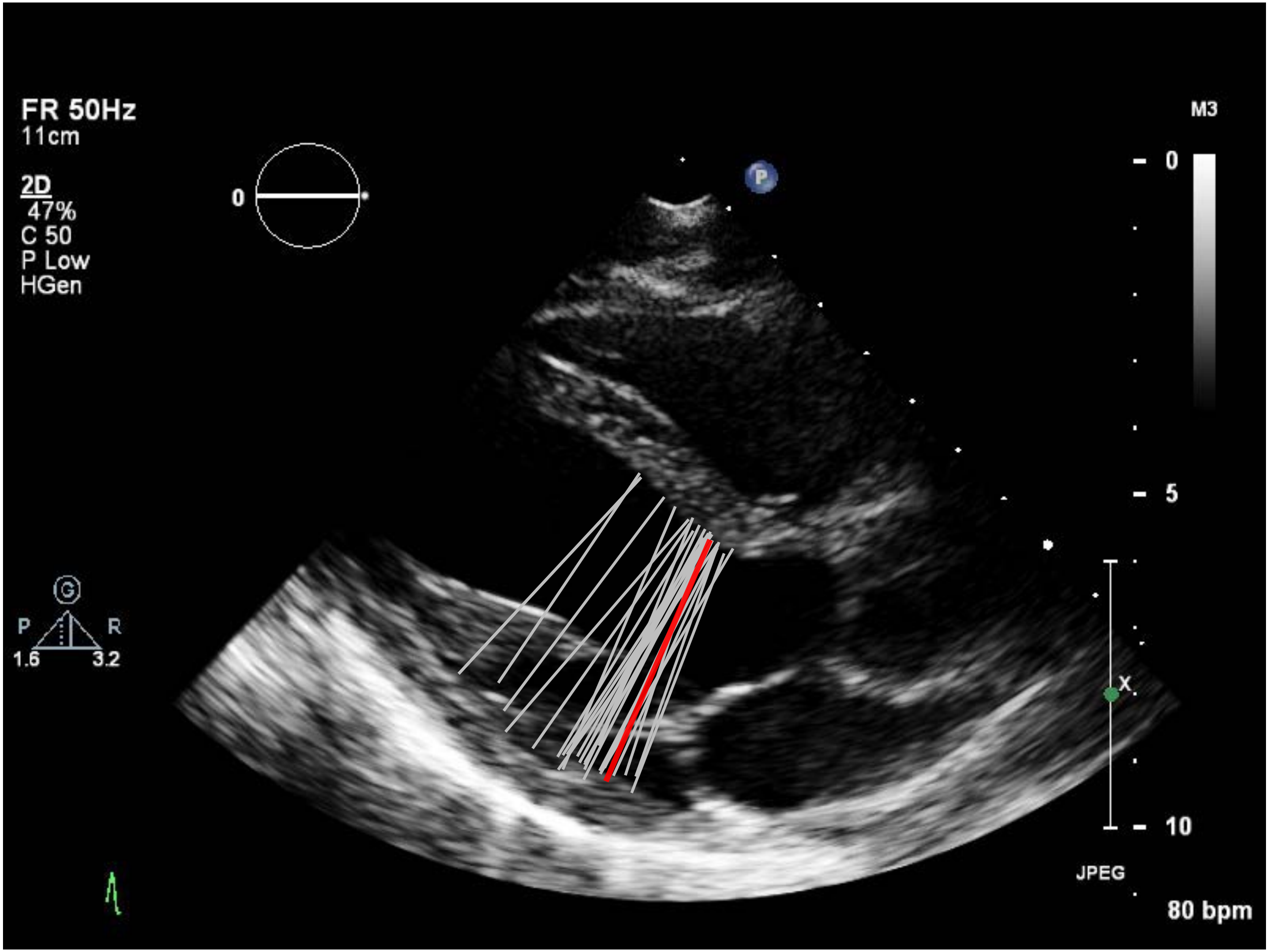

Pt 17 Diastole

Absolute difference between AI and expert consensus: 0.084 mm

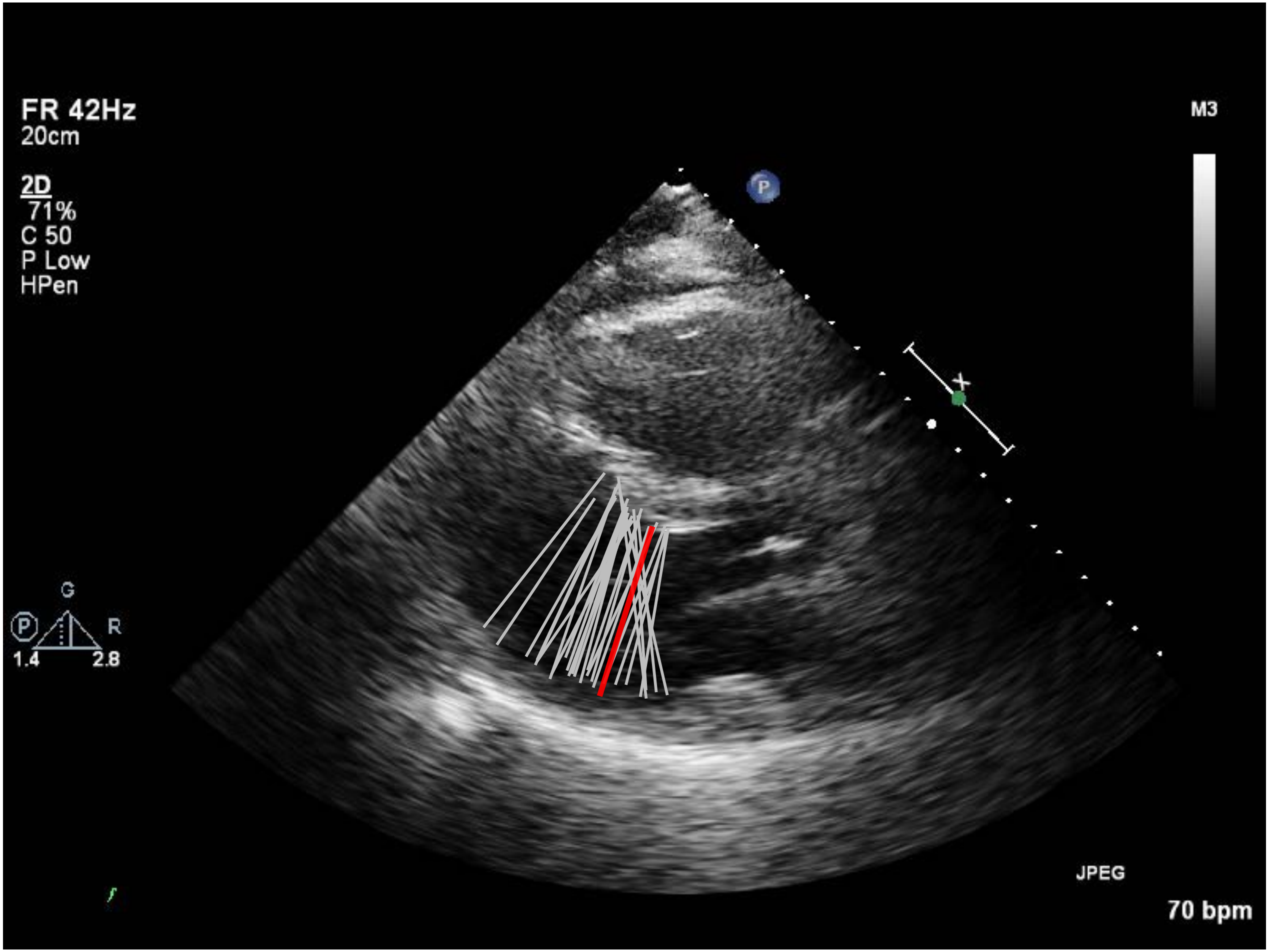

Pt 96 Diastole

Absolute difference between AI and expert consensus: 0.097 mm

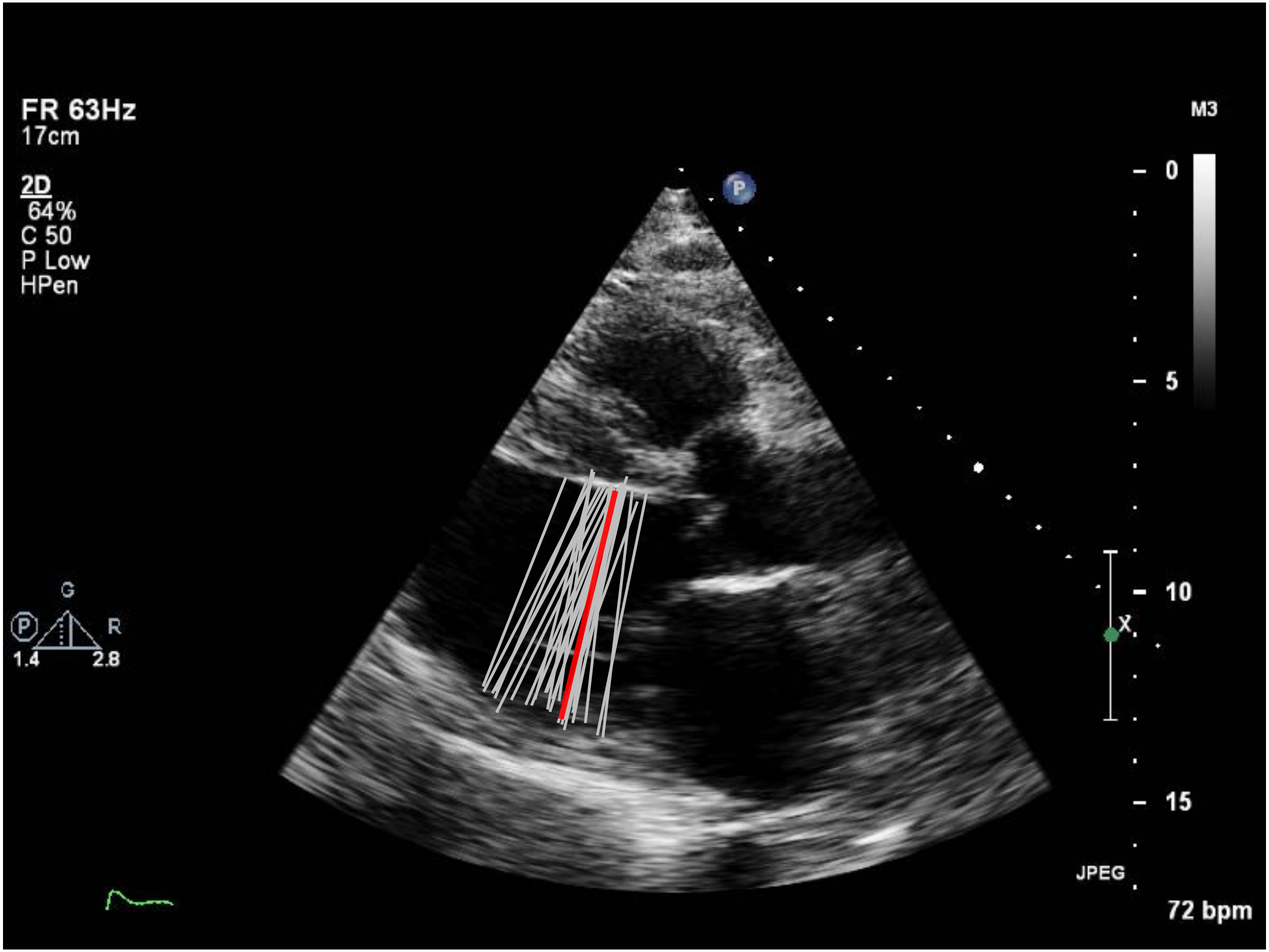

Pt 64 Systole

Absolute difference between AI and expert consensus: 0.11 mm

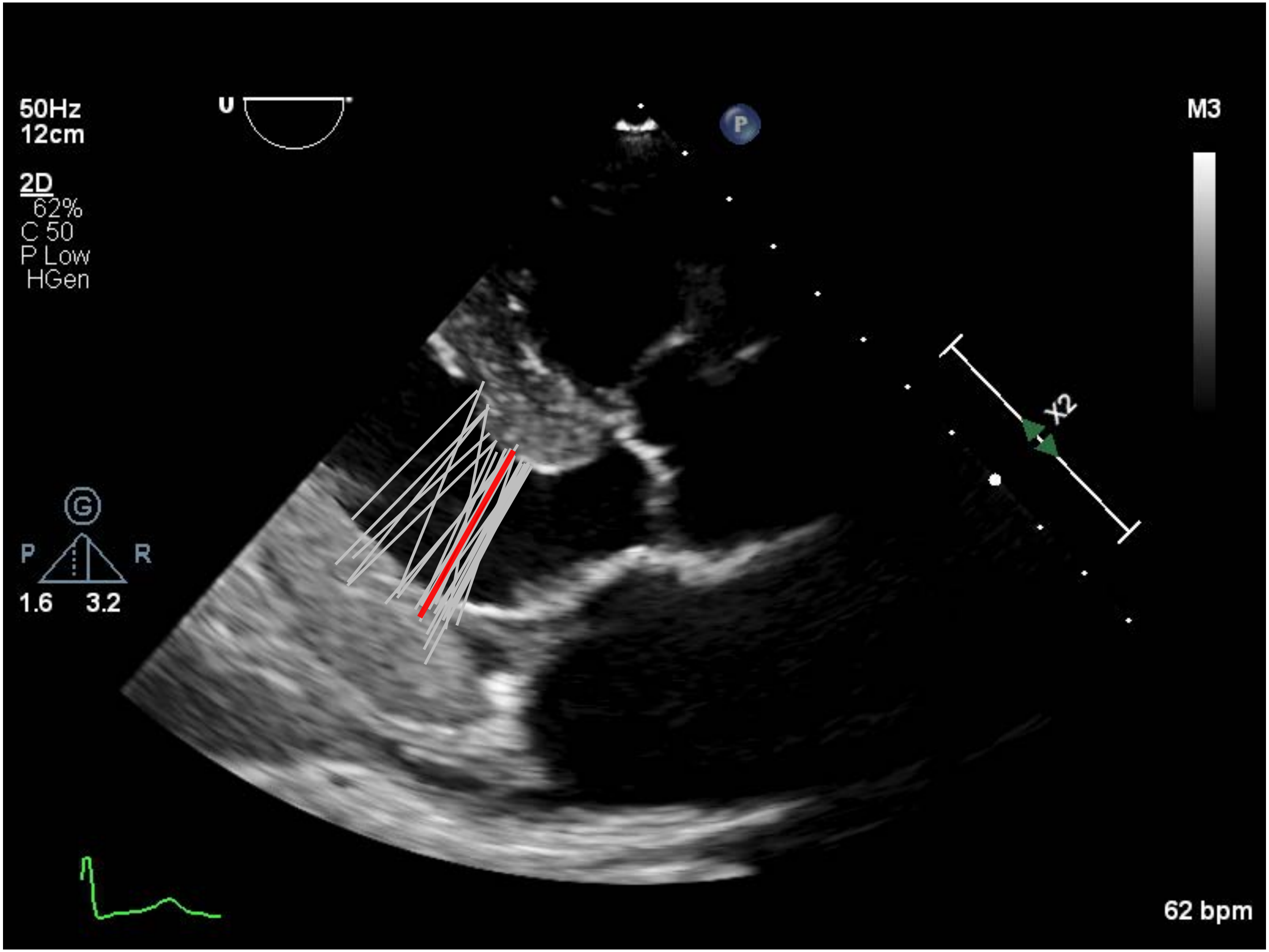

Pt 9 Systole

Absolute difference between AI and expert consensus: 0.13 mm

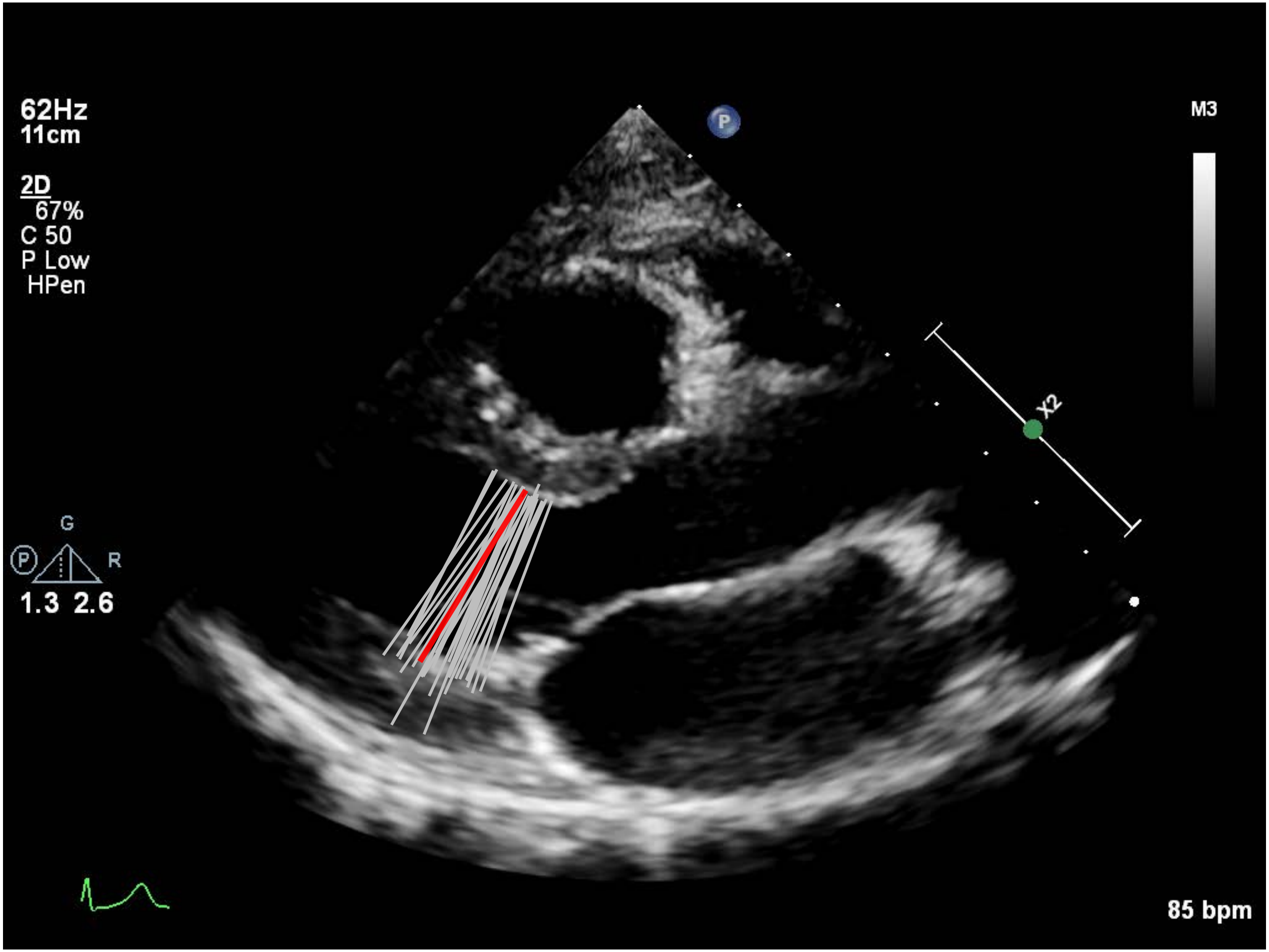

Pt 2 Systole
Absolute difference between AI and expert consensus: 0.17 mm

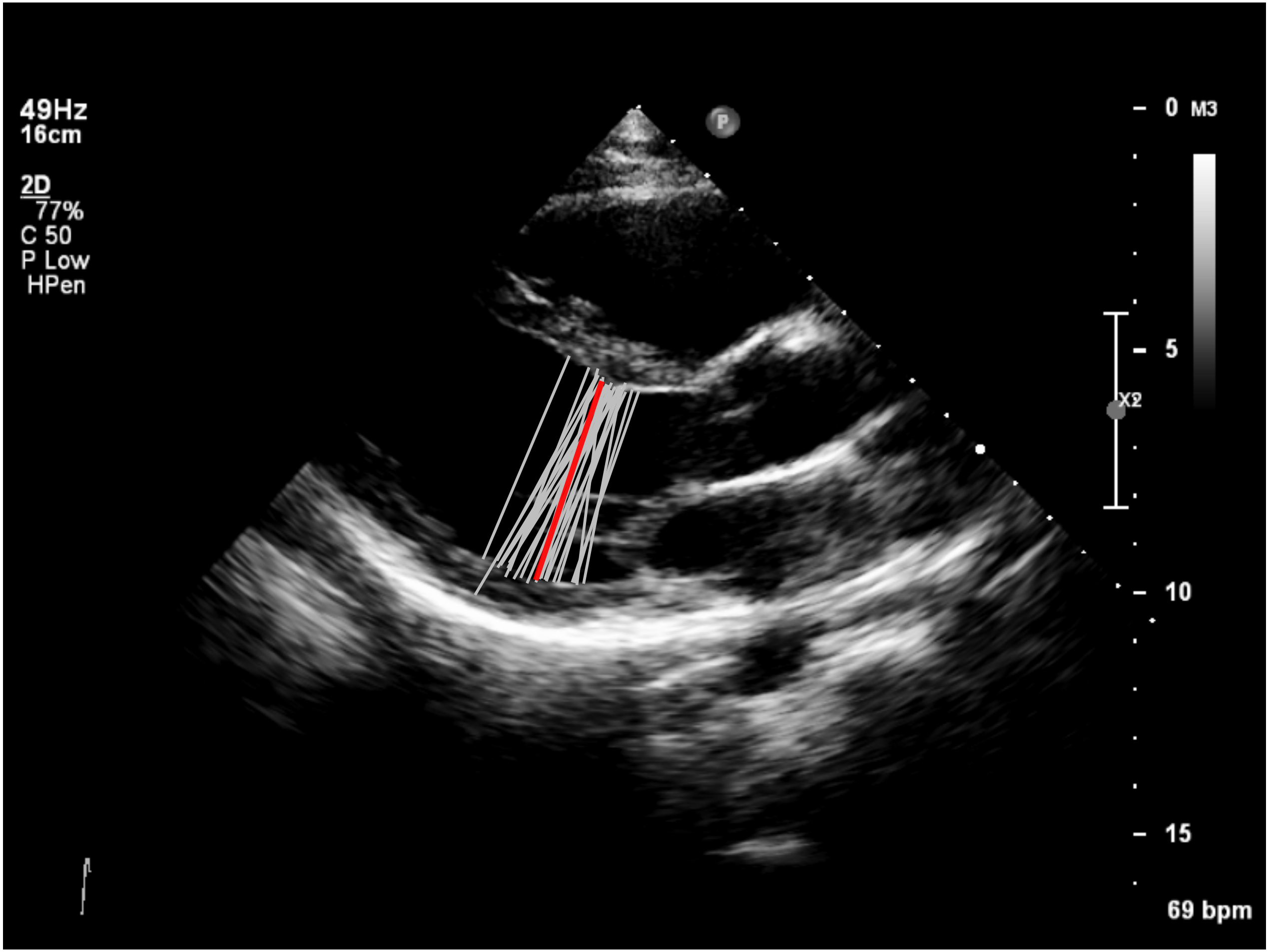

Pt 38 Diastole

Absolute difference between AI and expert consensus: 0.18 mm

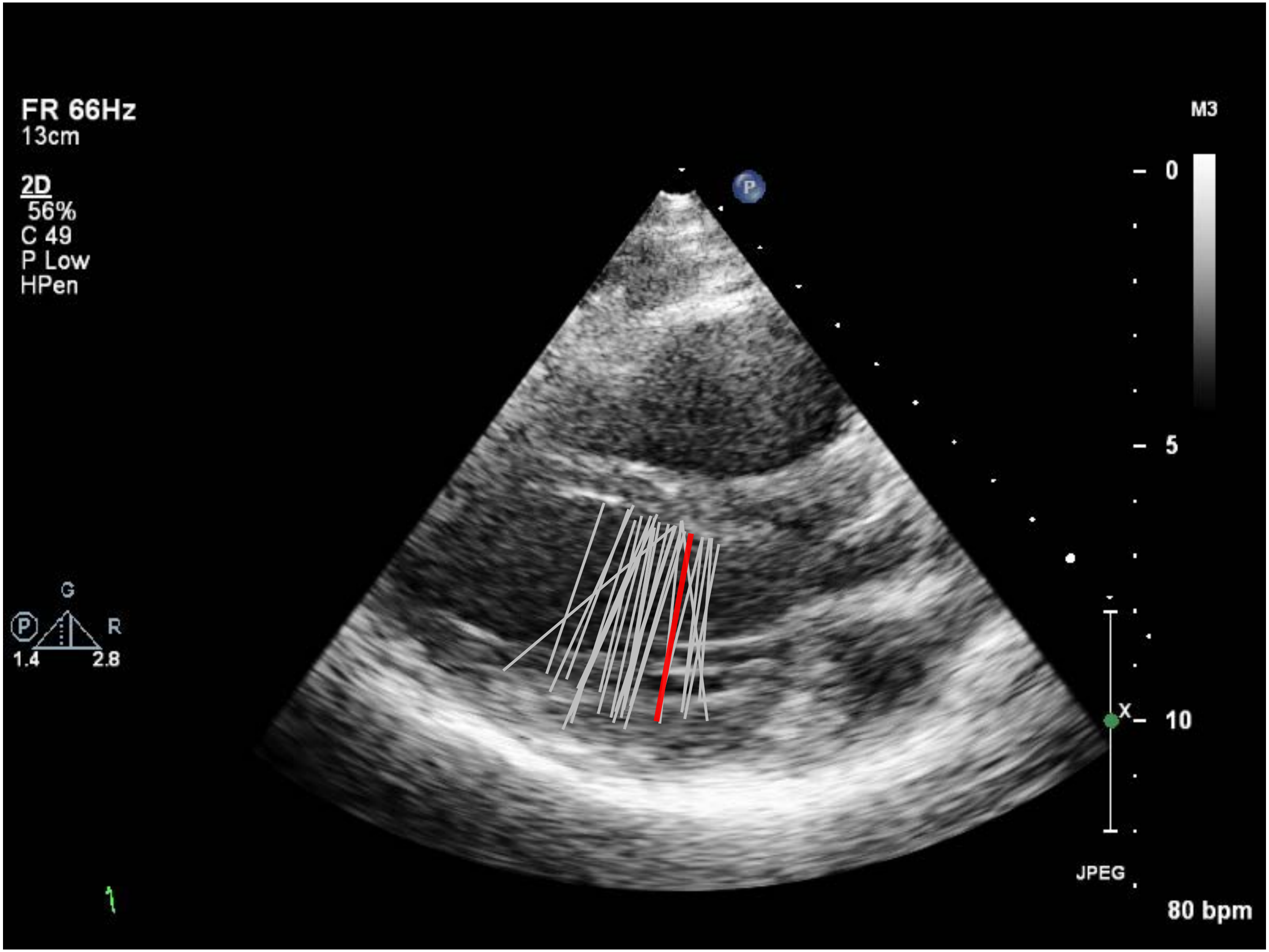

Pt 91 Diastole

Absolute difference between AI and expert consensus: 0.2 mm

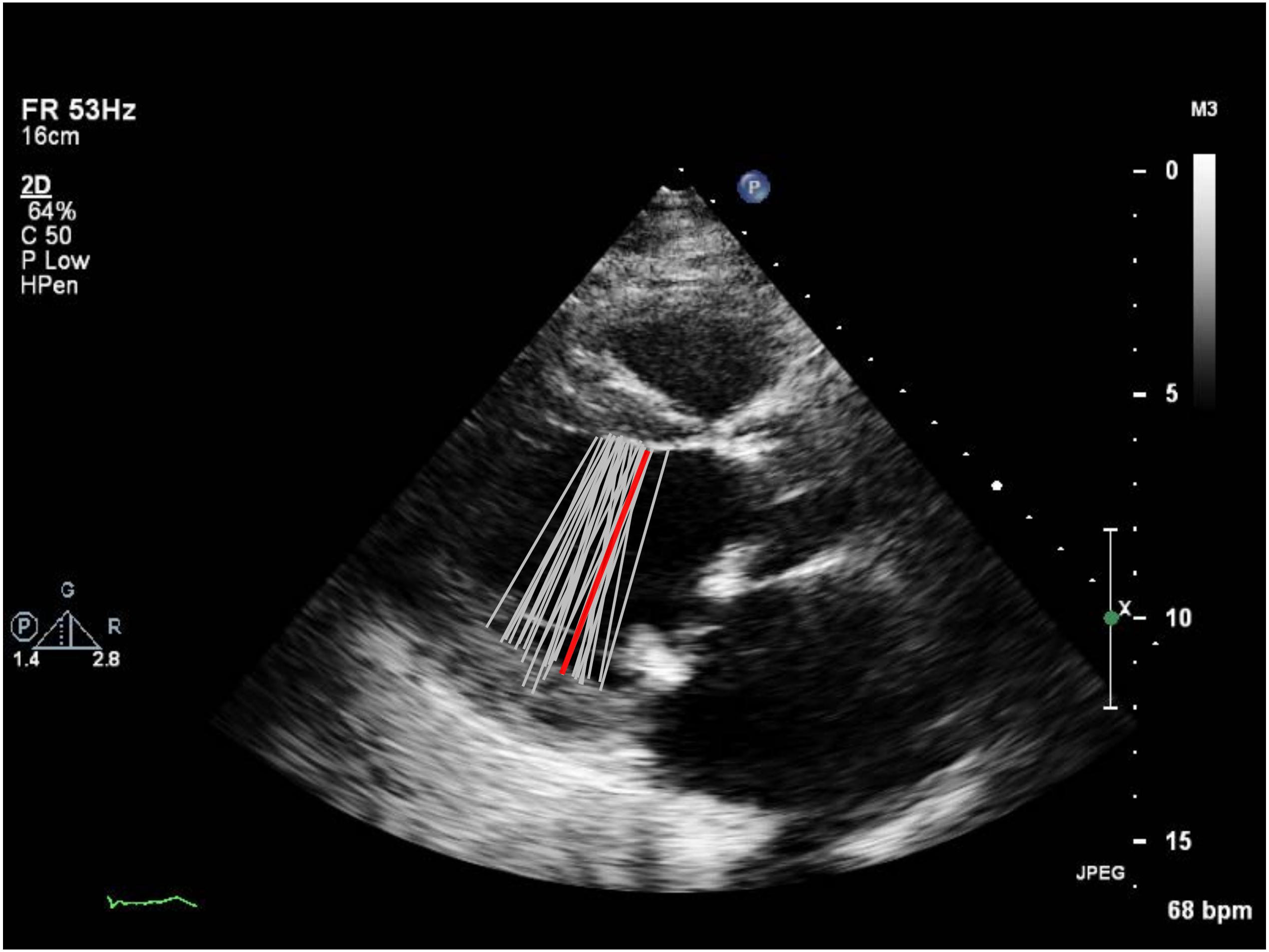

Pt 56 Systole

Absolute difference between AI and expert consensus: 0.21 mm

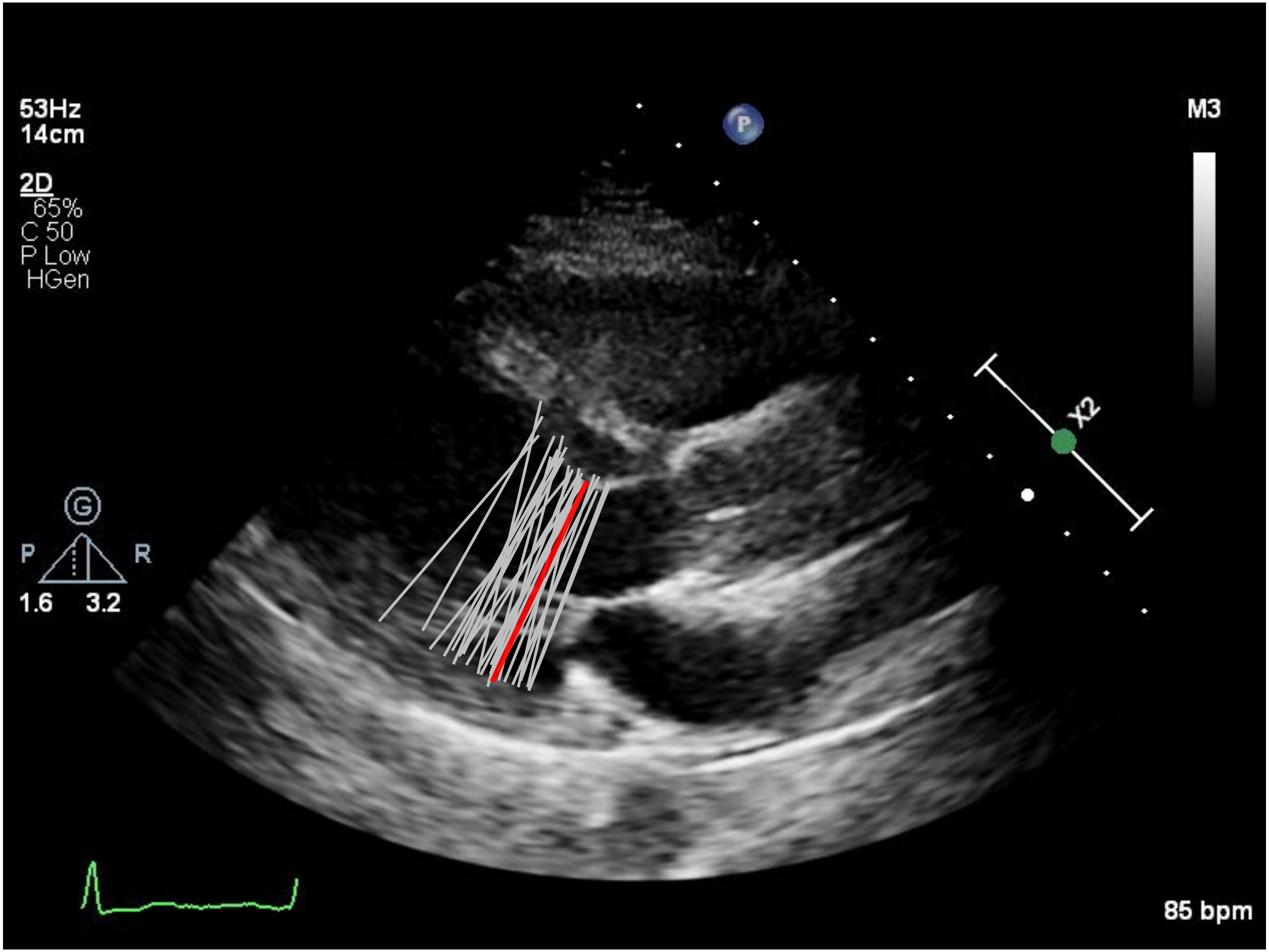

Pt 74 Diastole

Absolute difference between AI and expert consensus: 0.22 mm

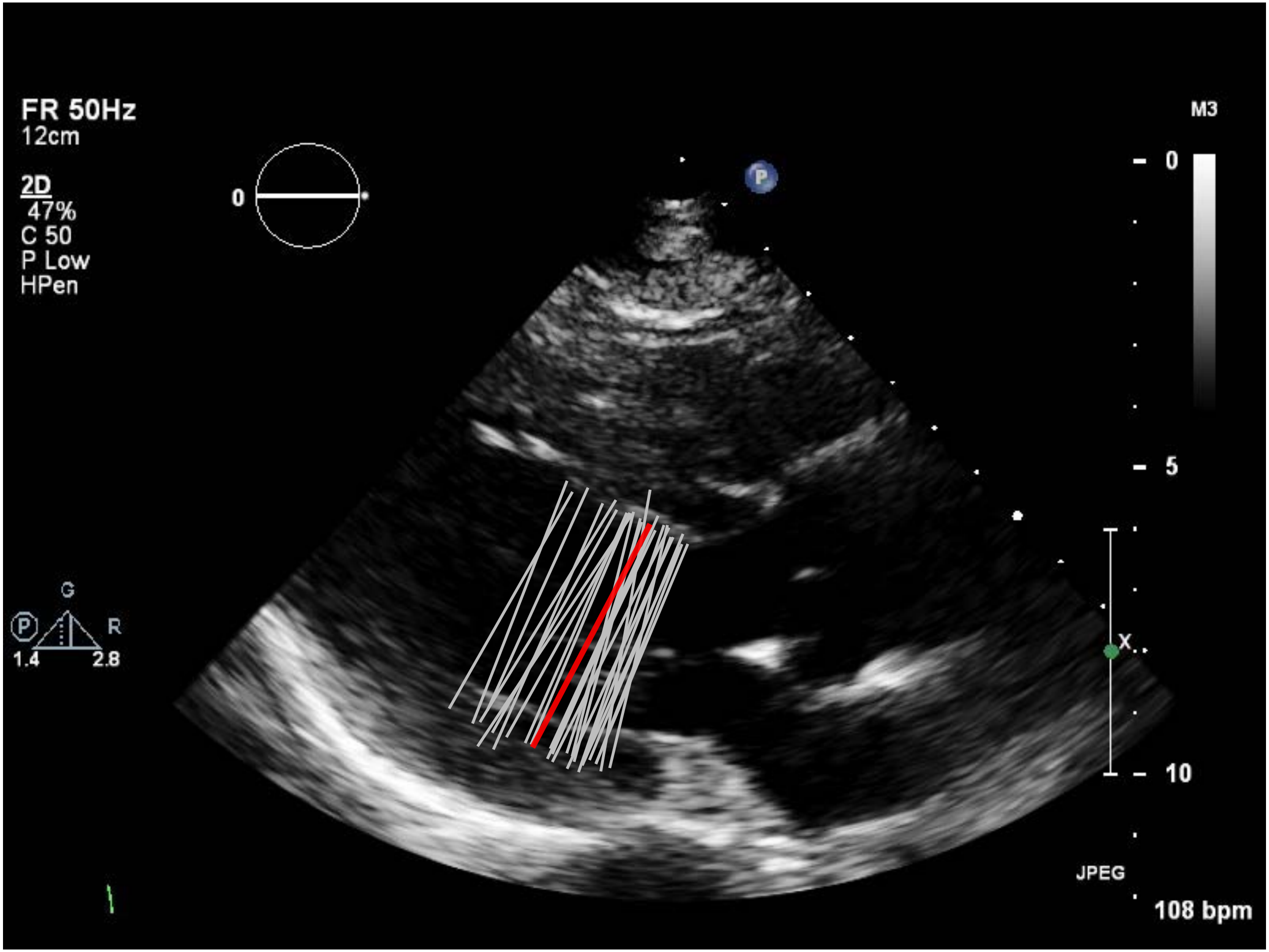

Pt 89 Diastole

Absolute difference between AI and expert consensus: 0.24 mm

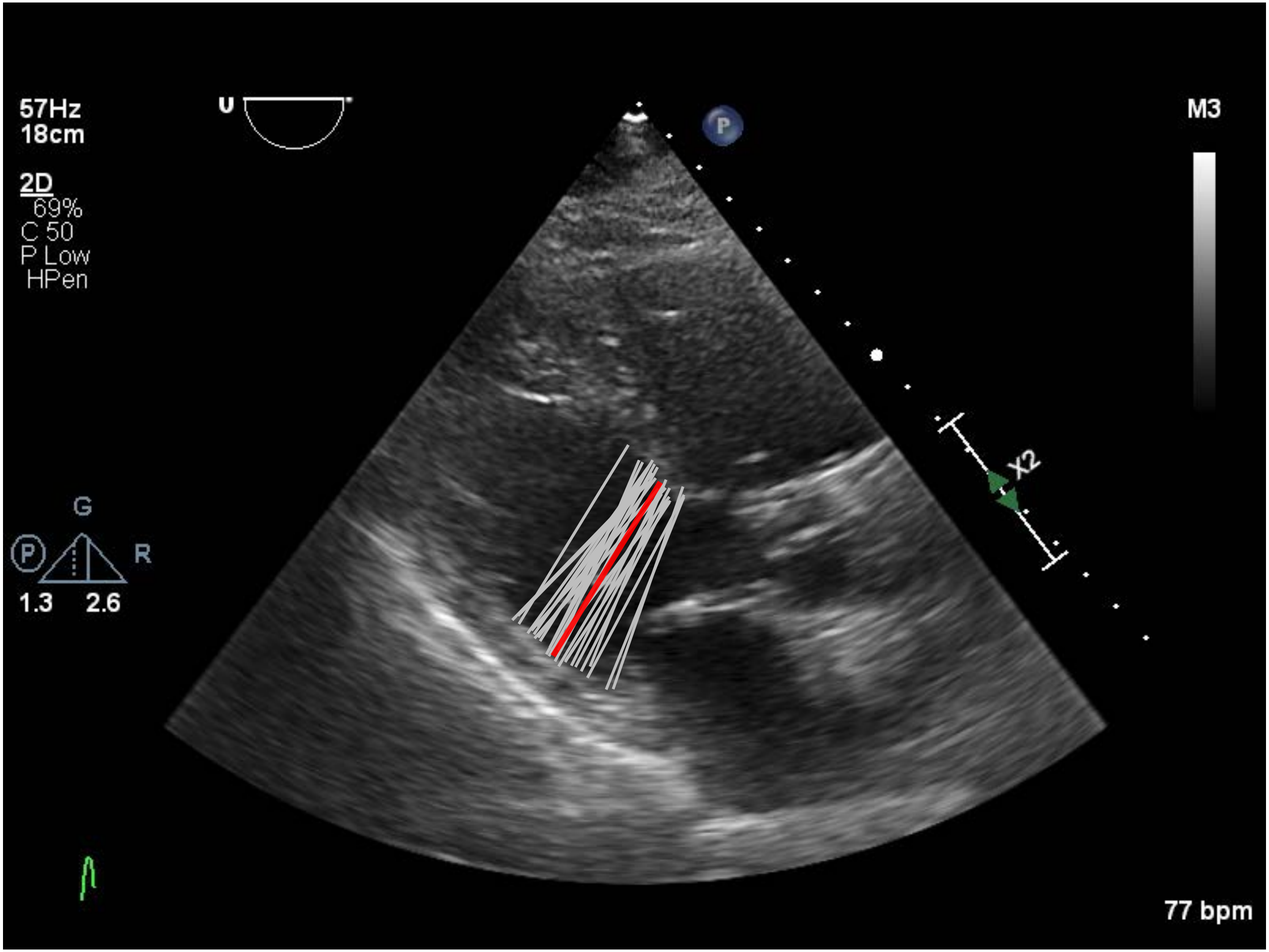

Pt 15 Diastole

Absolute difference between AI and expert consensus: 0.28 mm

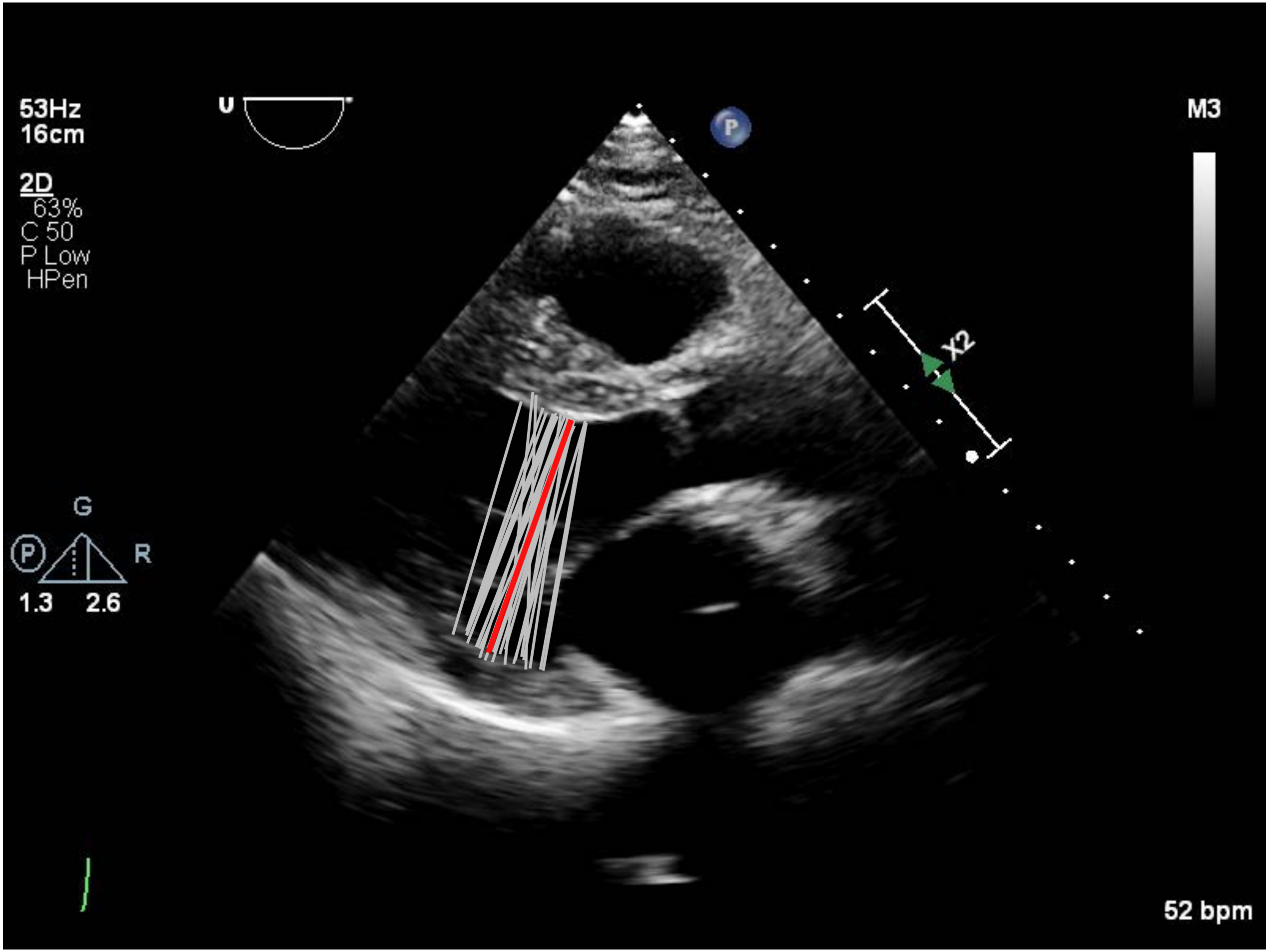

Pt 8 Diastole

Absolute difference between AI and expert consensus: 0.29 mm

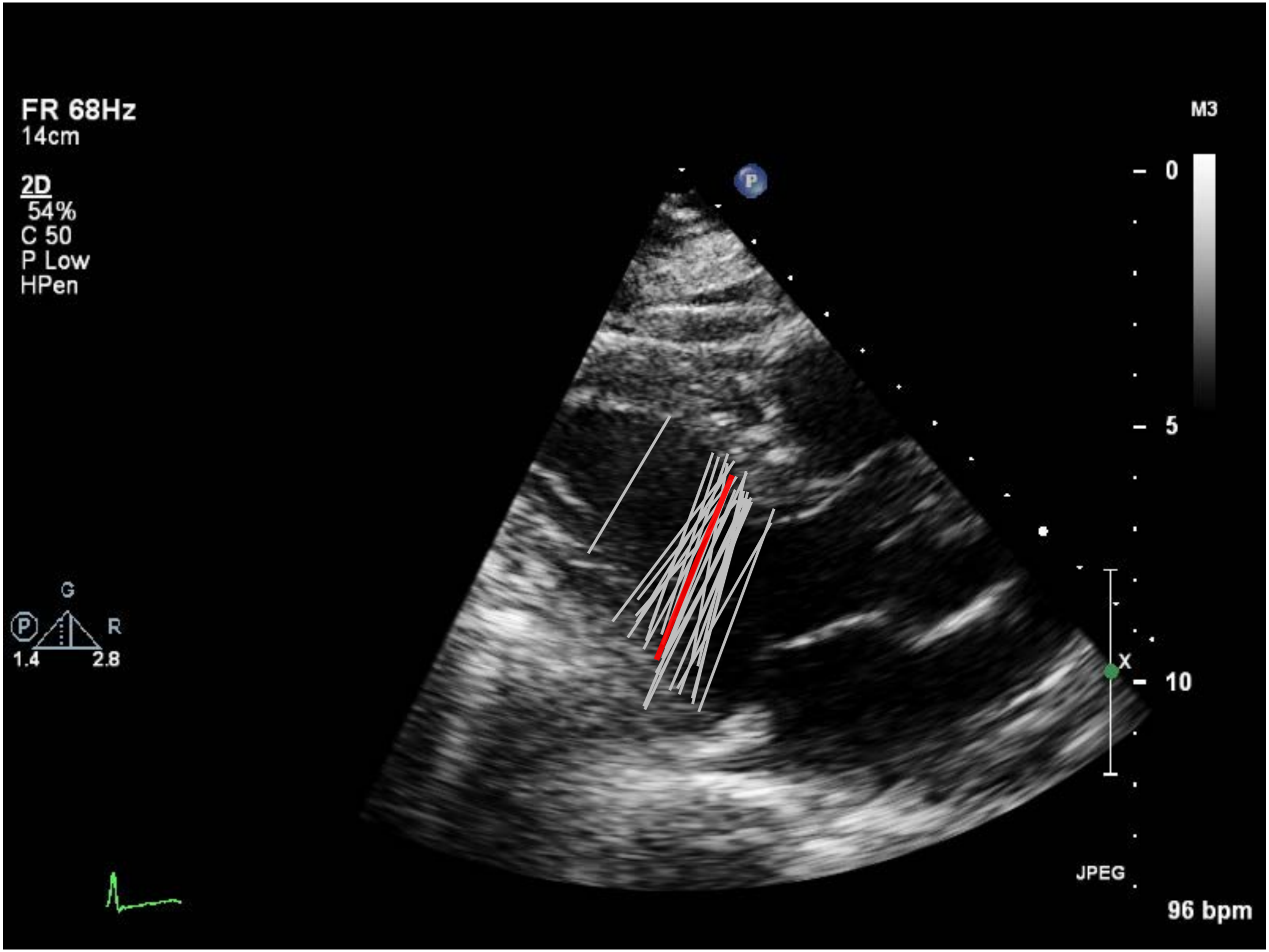

Pt 49 Systole

Absolute difference between AI and expert consensus: 0.32 mm

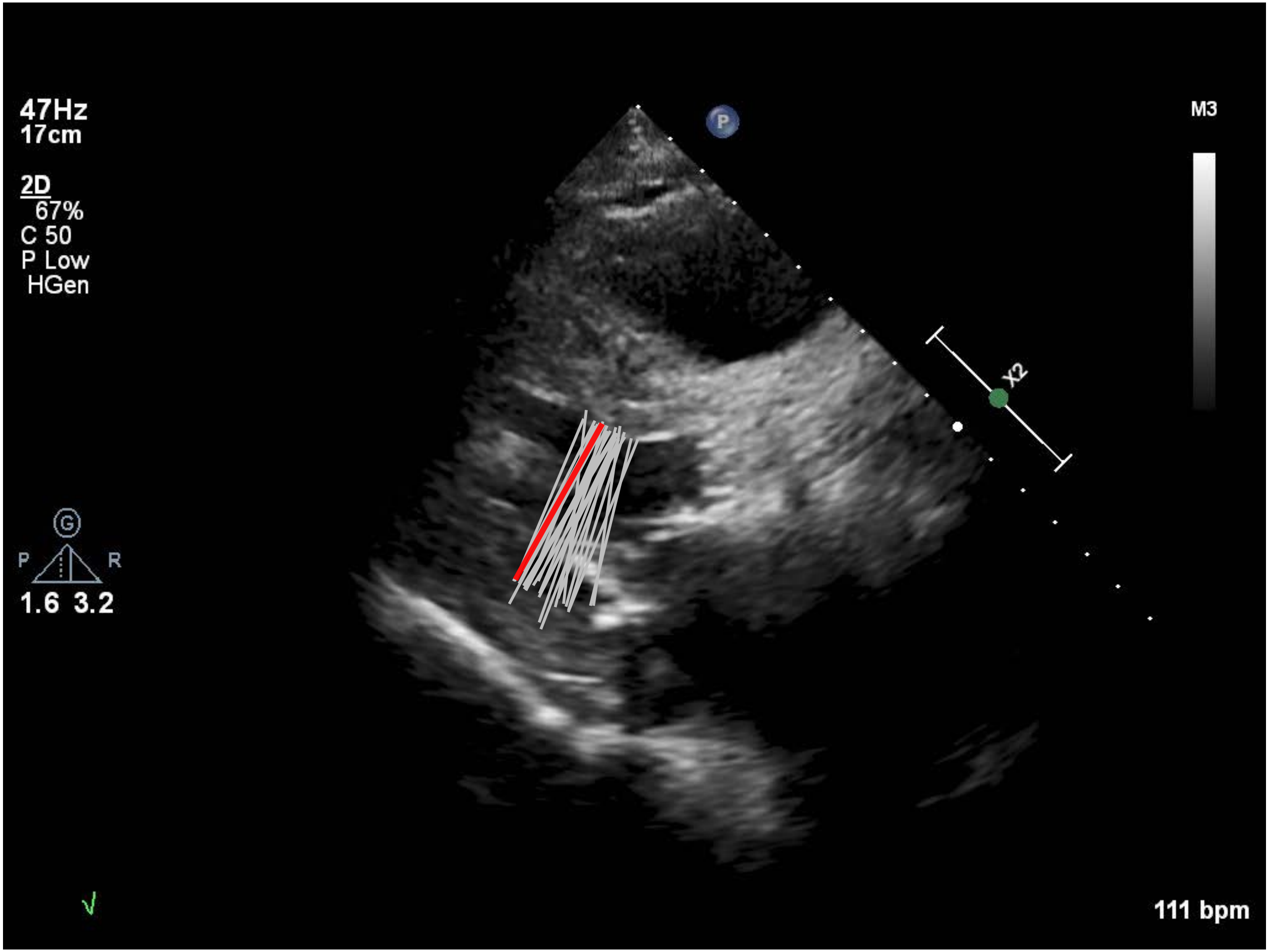

Pt 65 Diastole
Absolute difference between AI and expert consensus: 0.33 mm

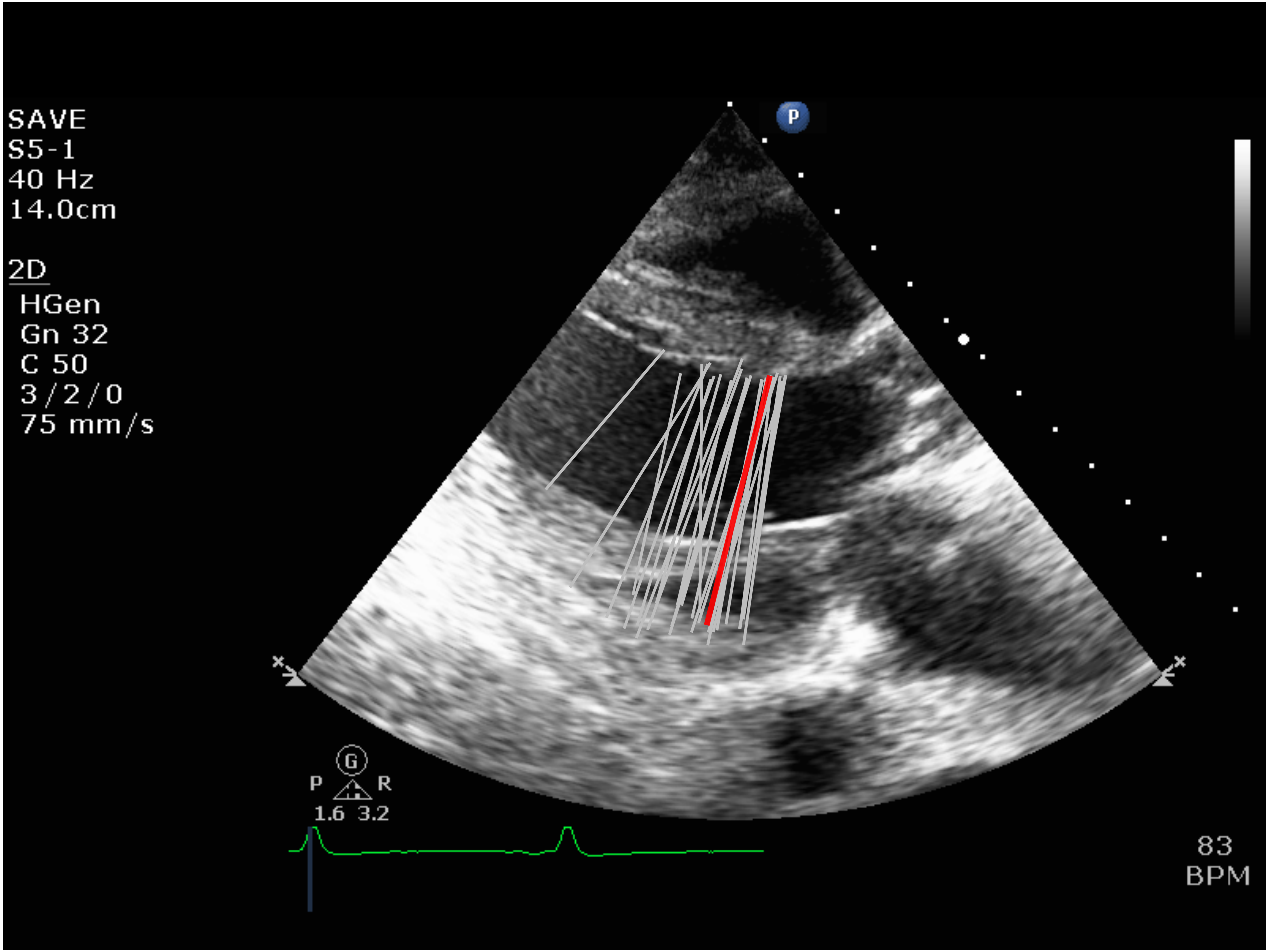

Pt 84 Diastole

Absolute difference between AI and expert consensus: 0.34 mm

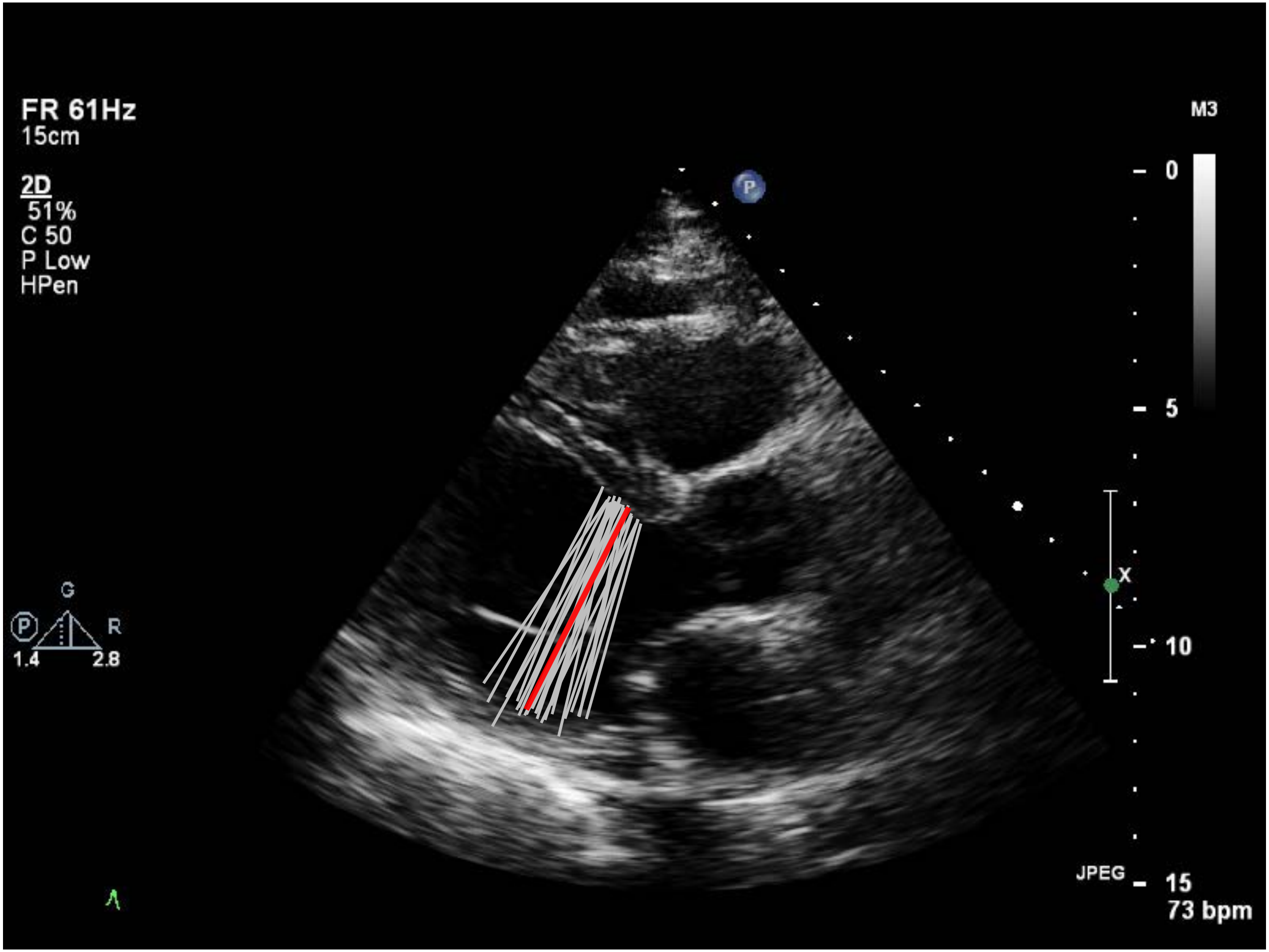

Pt 93 Diastole

Absolute difference between AI and expert consensus: 0.37 mm

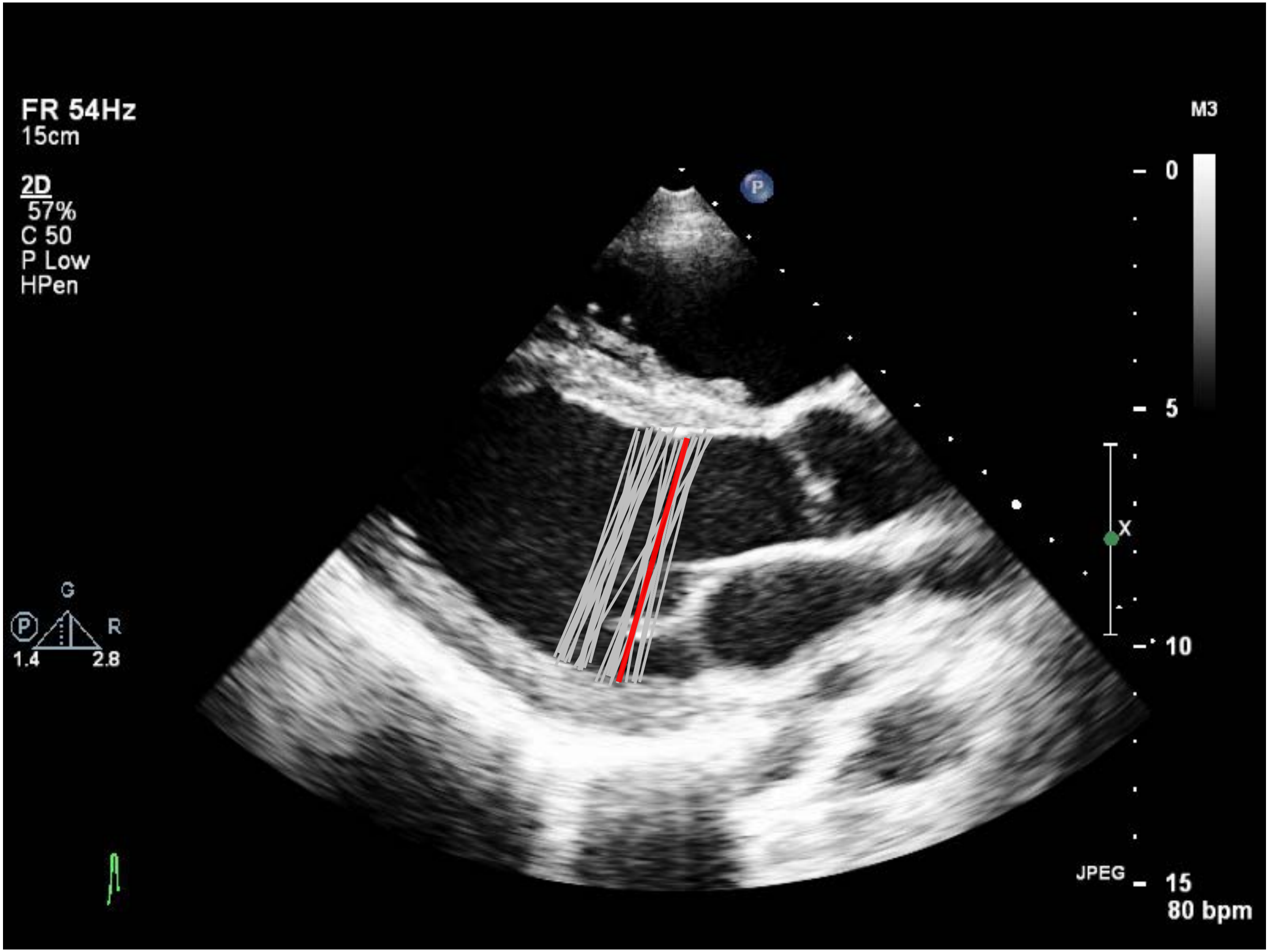

Pt 82 Diastole

Absolute difference between AI and expert consensus: 0.4 mm

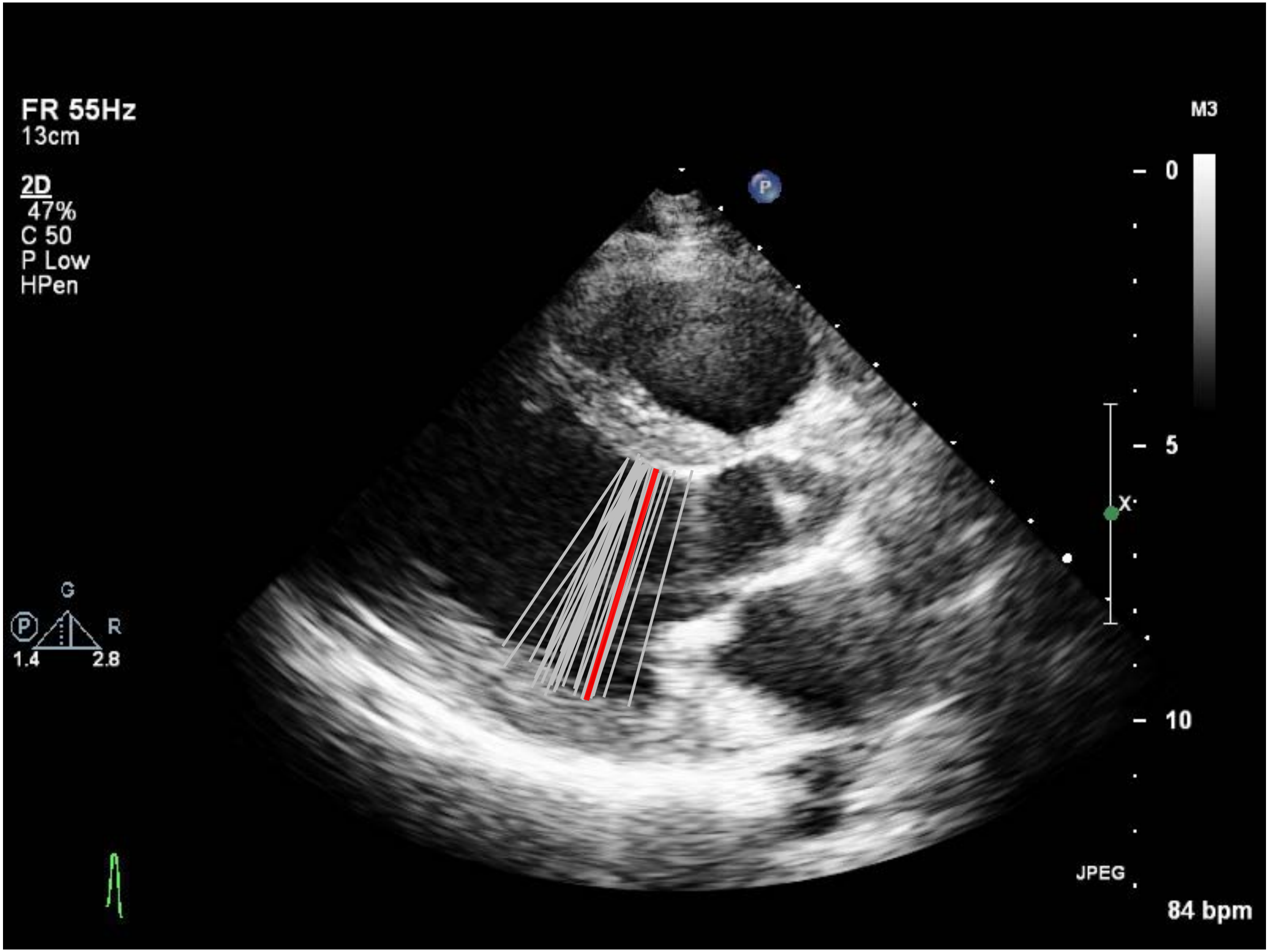

Pt 46 Diastole

Absolute difference between AI and expert consensus: 0.41 mm

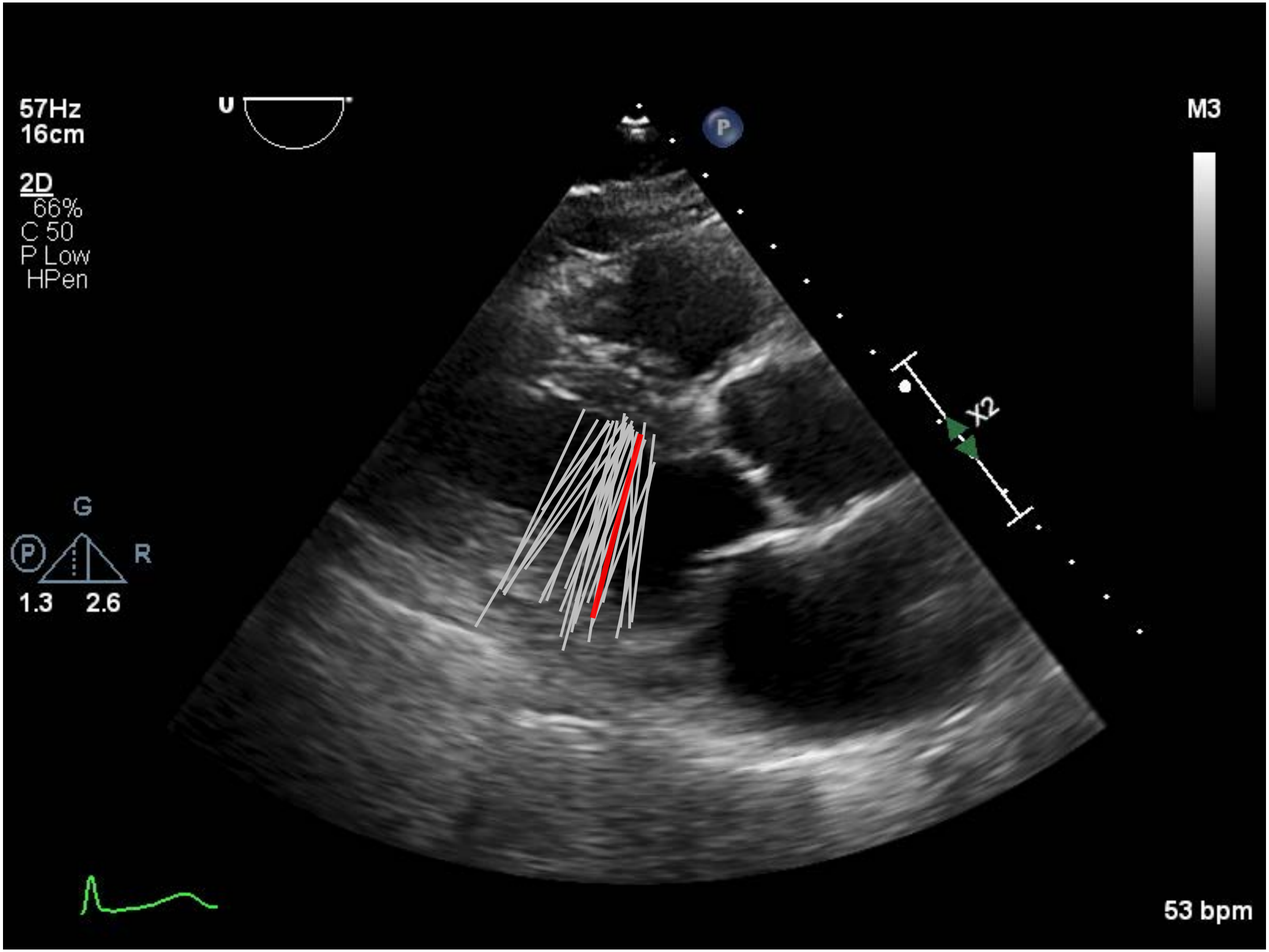

Pt 7 Systole

Absolute difference between AI and expert consensus: 0.42 mm

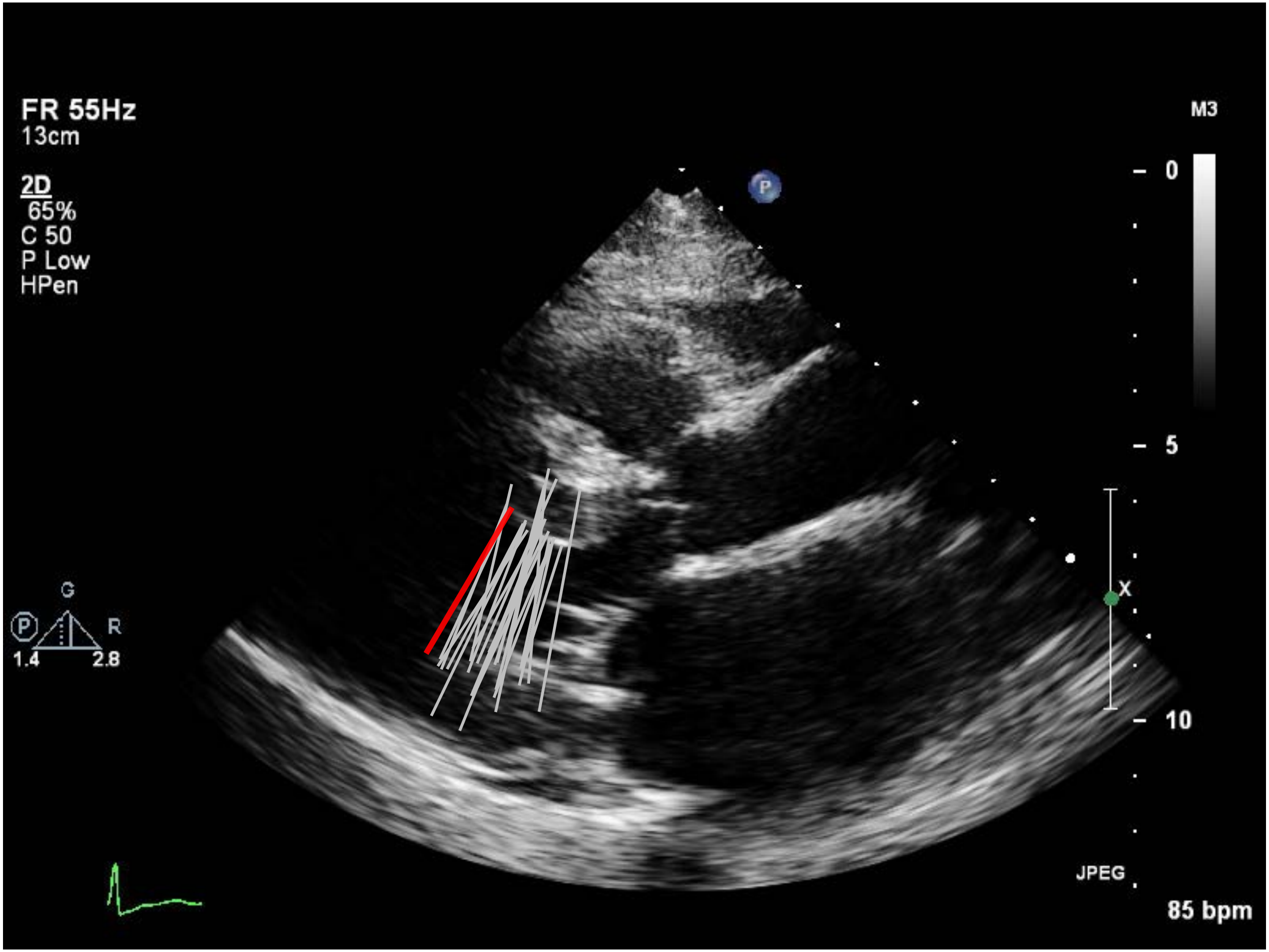

Pt 73 Systole

Absolute difference between AI and expert consensus: 0.43 mm

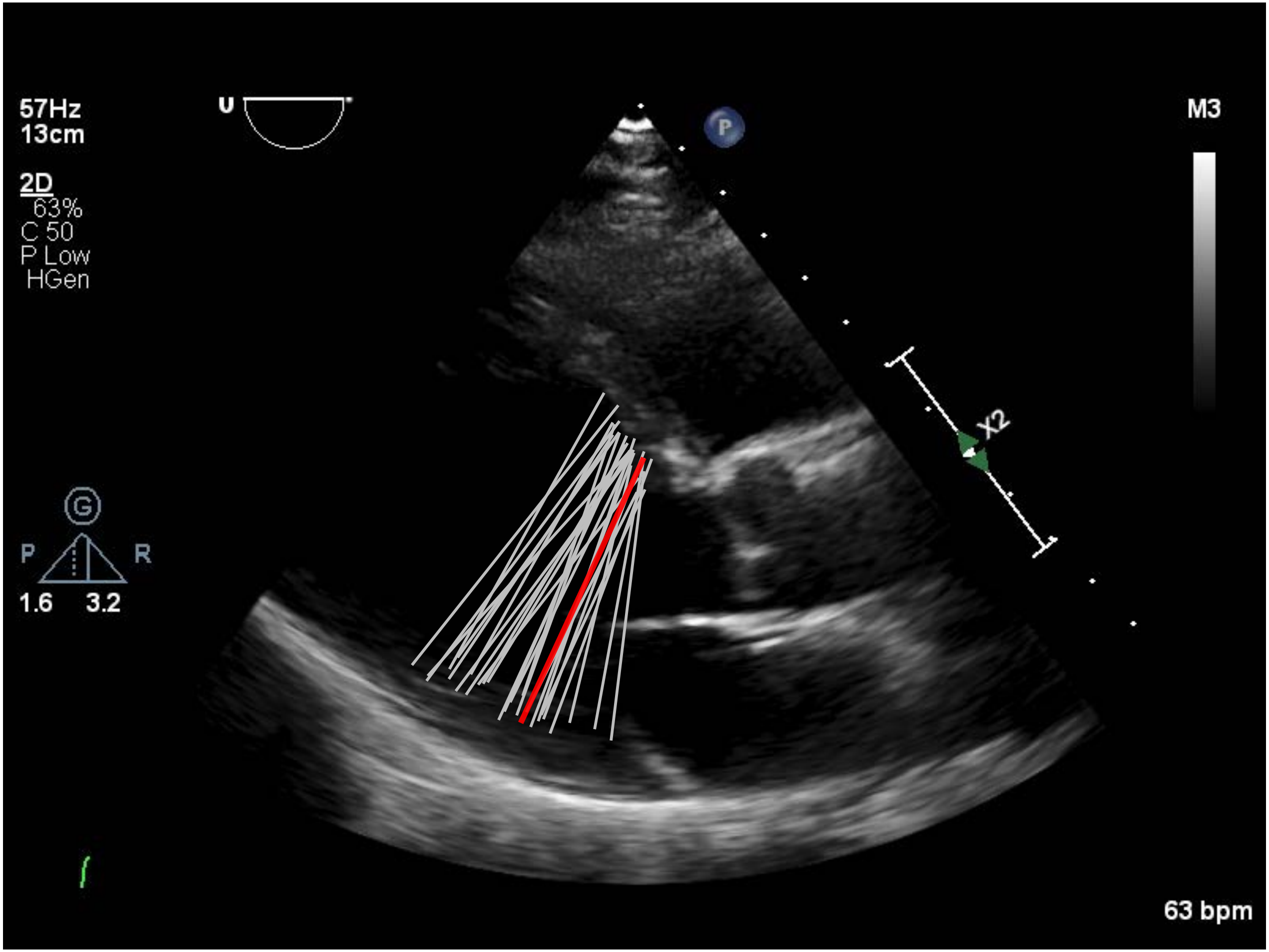

Pt 53 Diastole

Absolute difference between AI and expert consensus: 0.43 mm

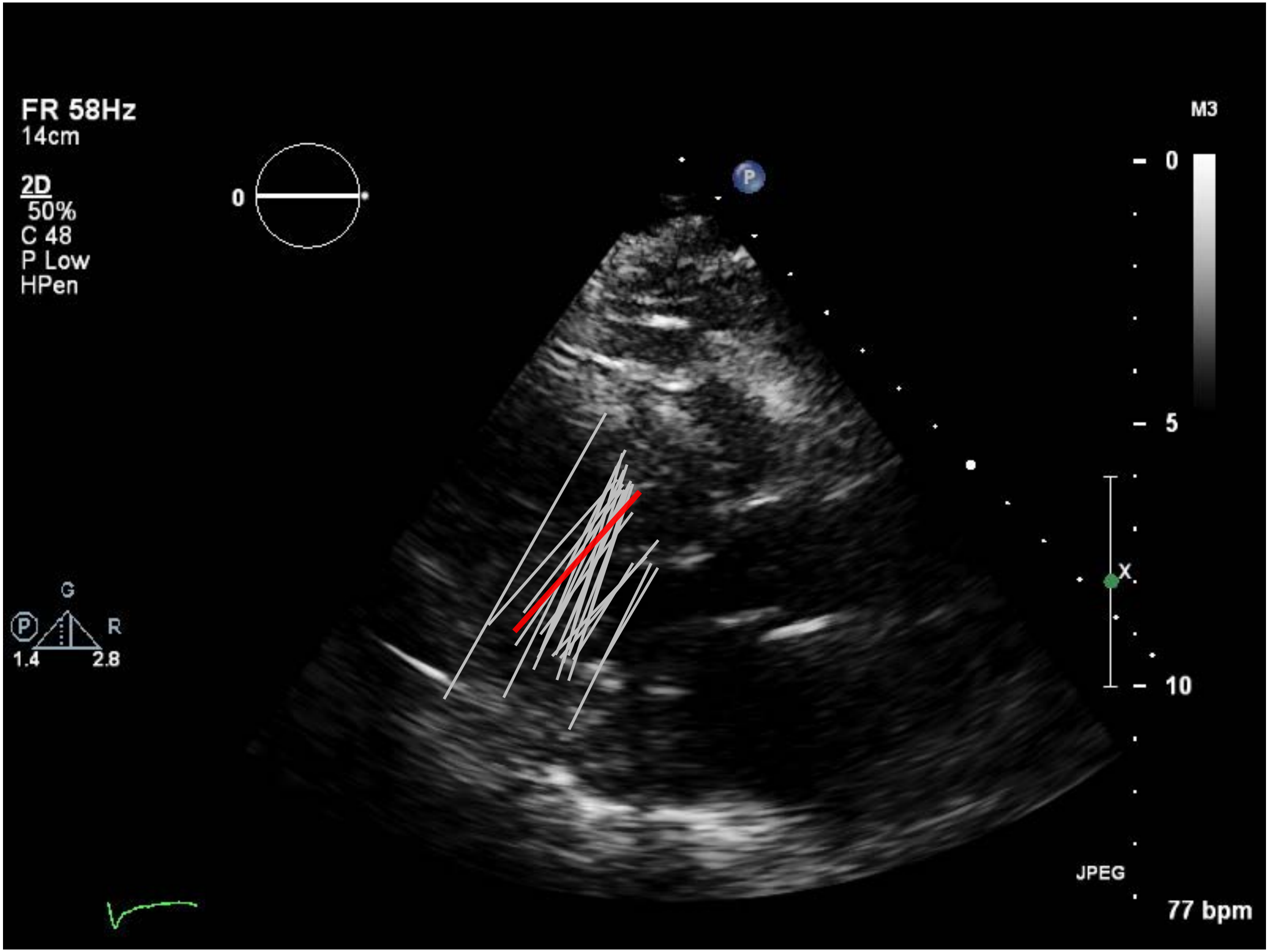

Pt 3 Systole

Absolute difference between AI and expert consensus: 0.45 mm

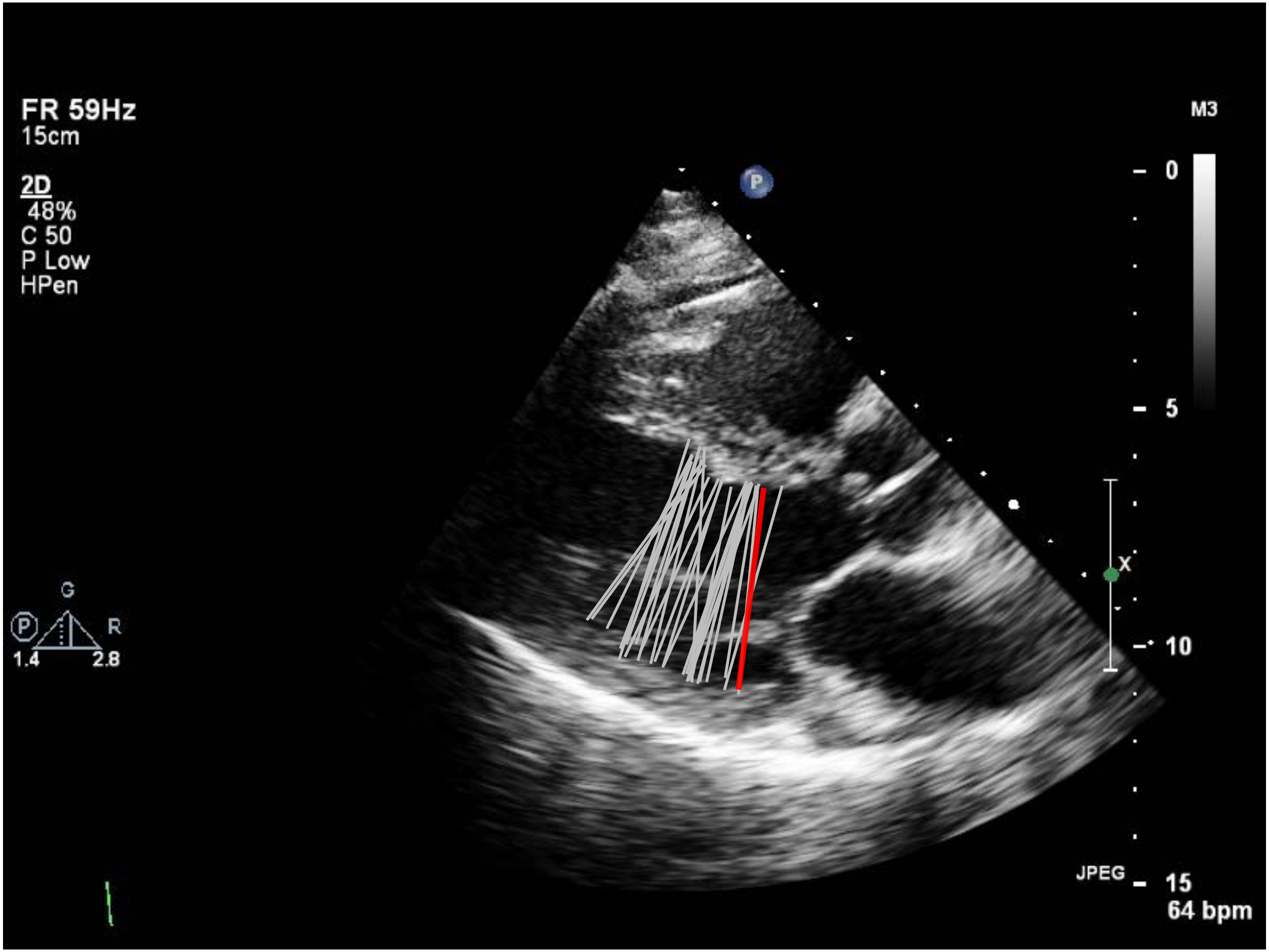

Pt 27 Diastole

Absolute difference between AI and expert consensus: 0.45 mm

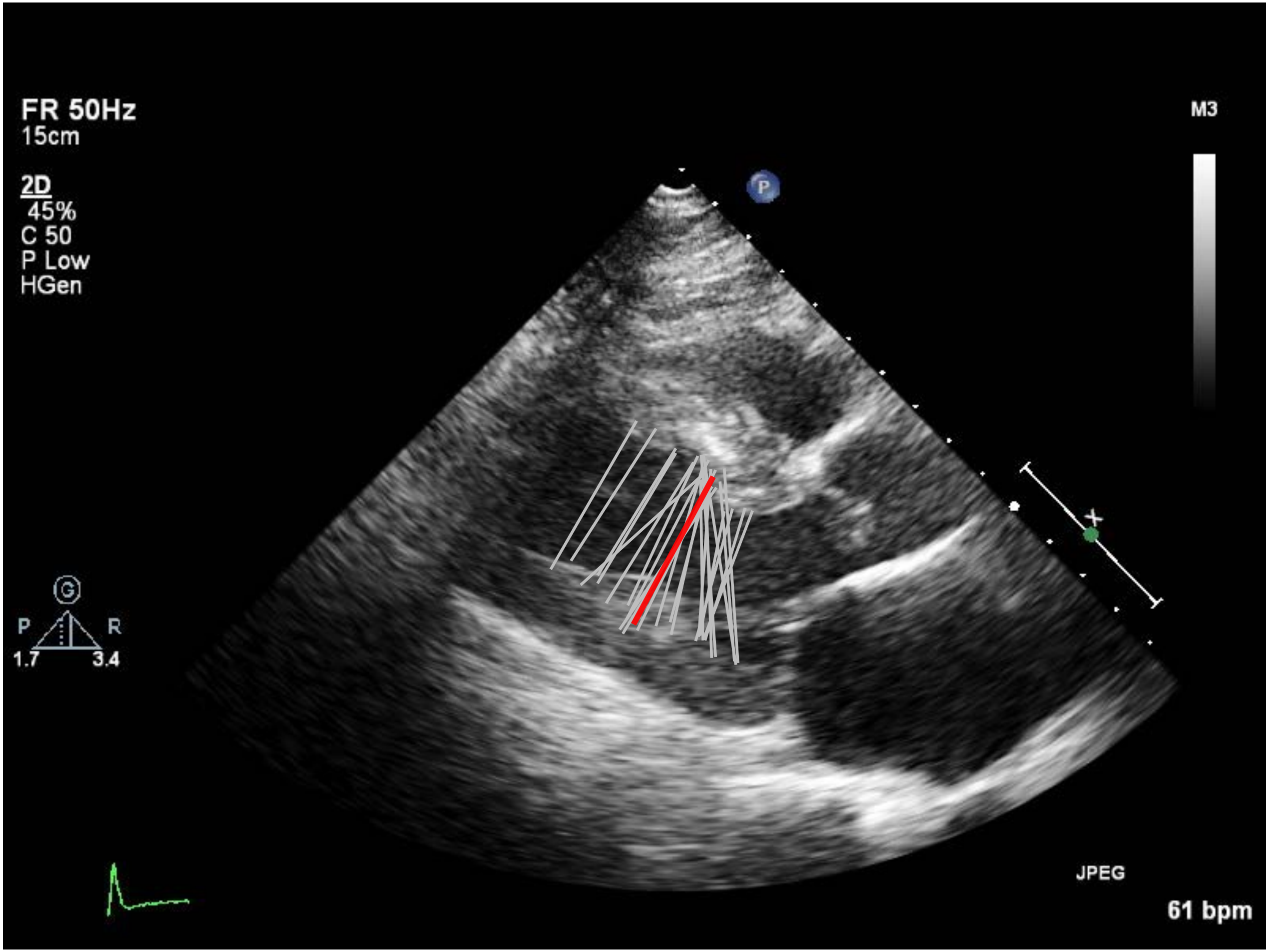

Pt 85 Systole

Absolute difference between AI and expert consensus: 0.46 mm

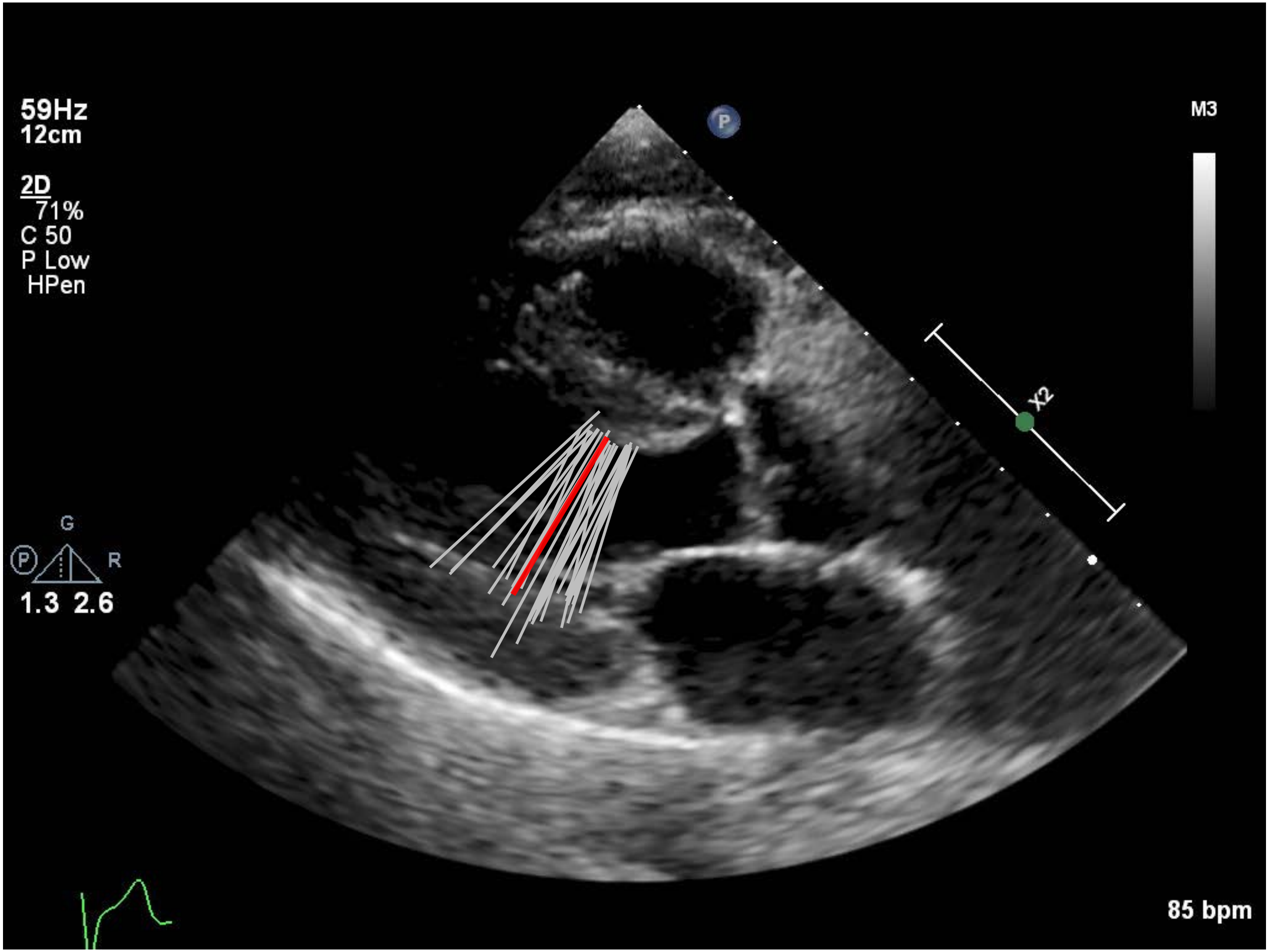

Pt 75 Systole
Absolute difference between AI and expert consensus: 0.46 mm

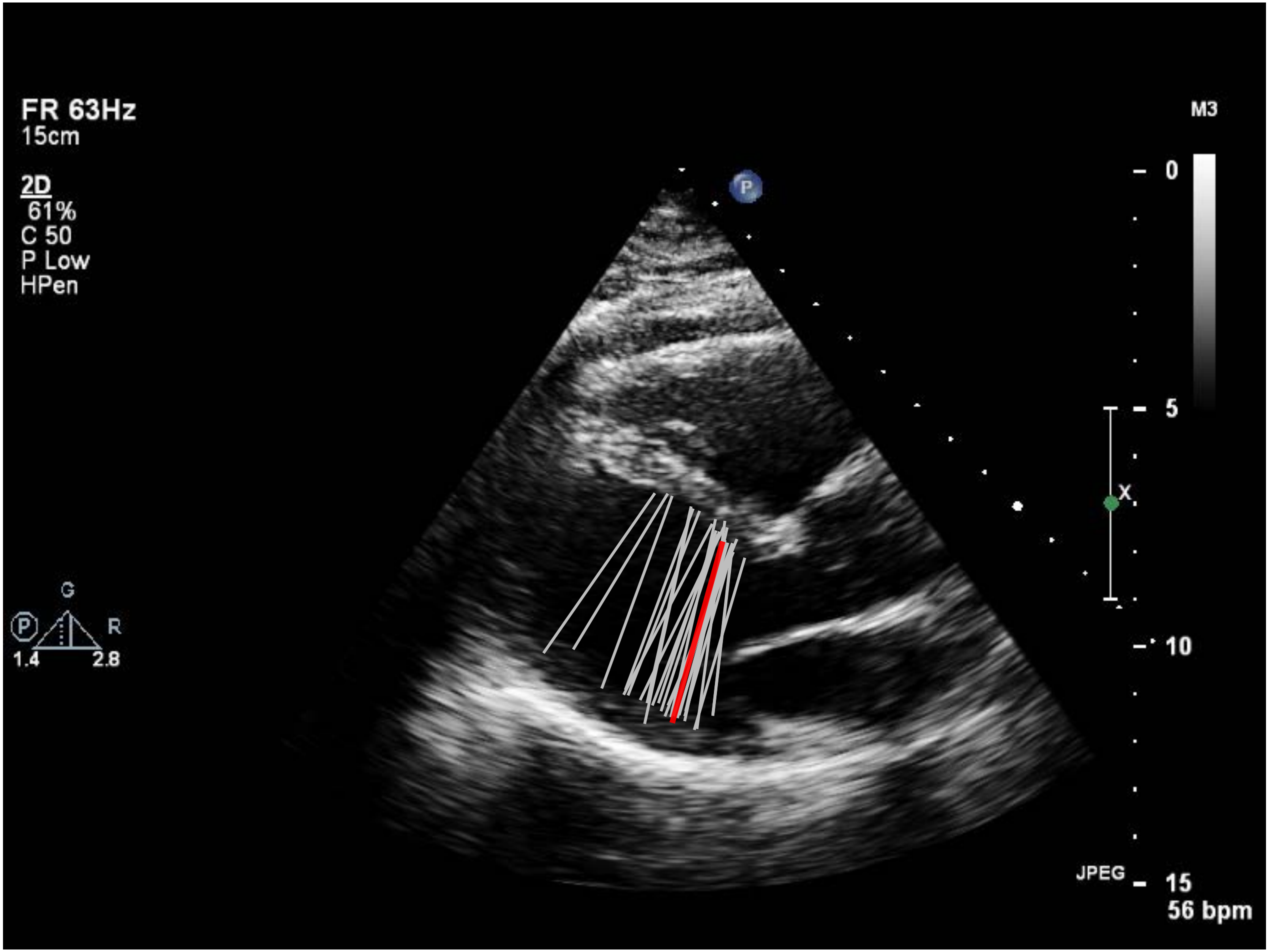

Pt 98 Diastole

Absolute difference between AI and expert consensus: 0.49 mm

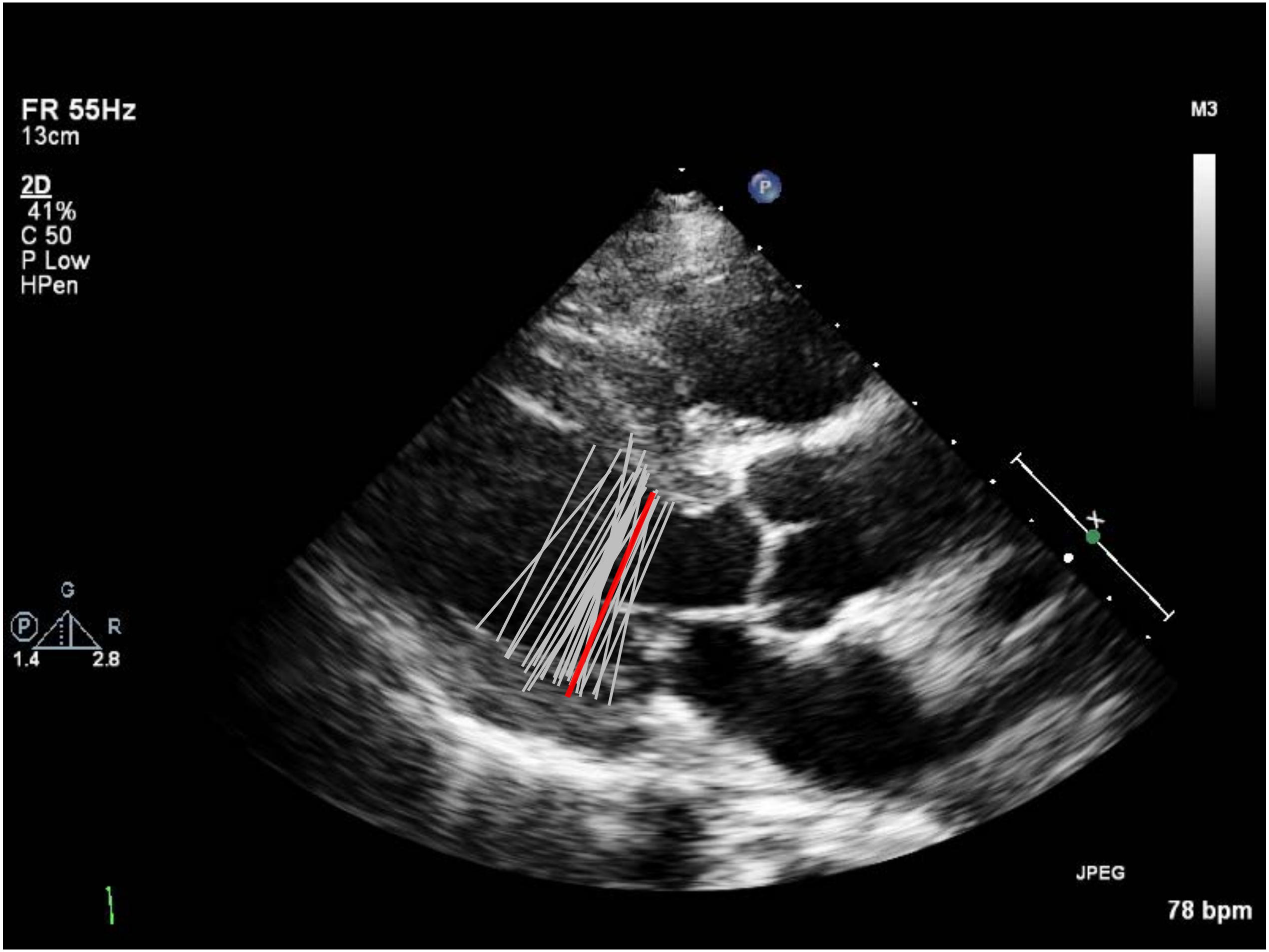

Pt 31 Diastole

Absolute difference between AI and expert consensus: 0.5 mm

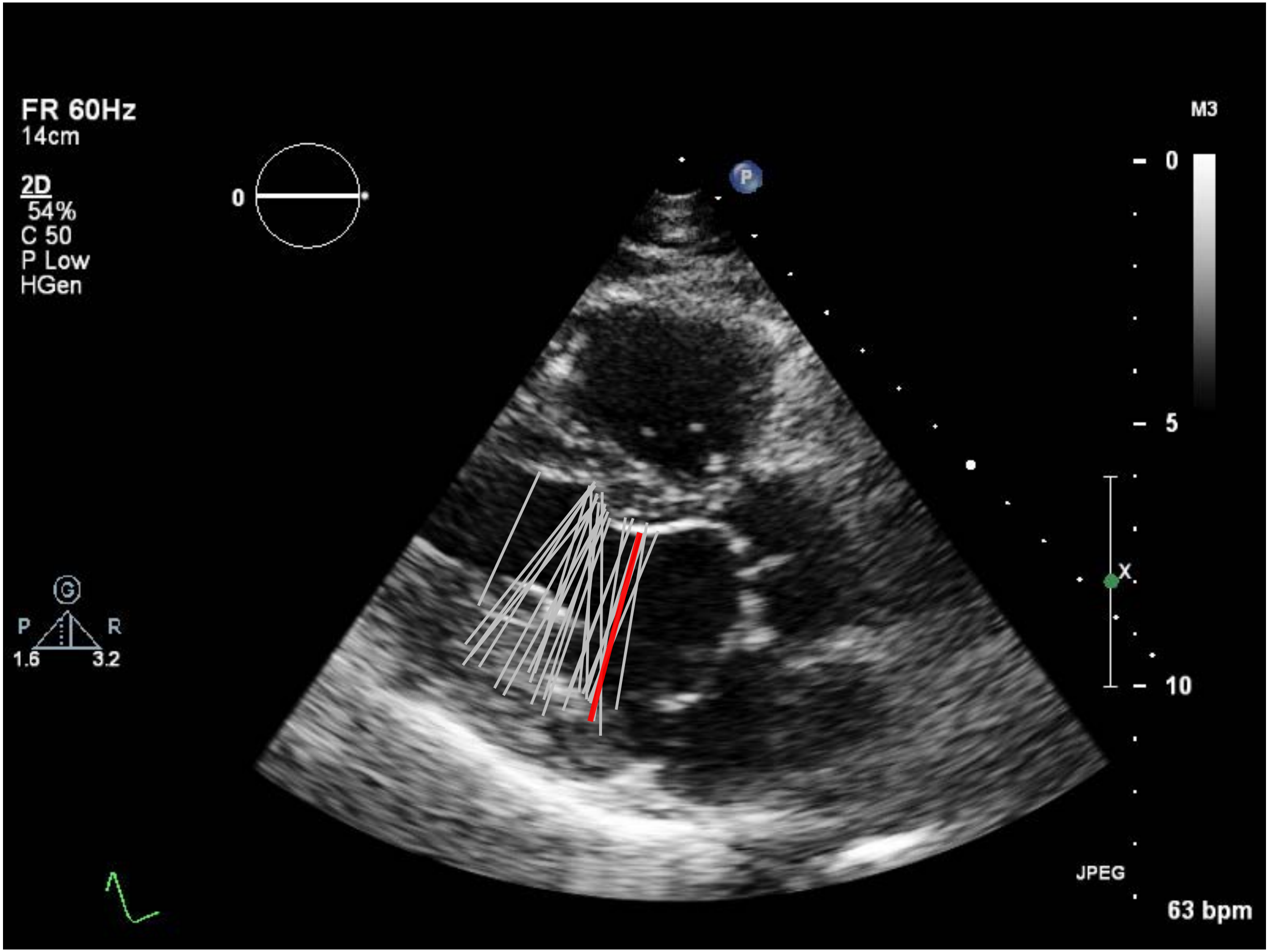

Pt 10 Diastole

Absolute difference between AI and expert consensus: 0.51 mm

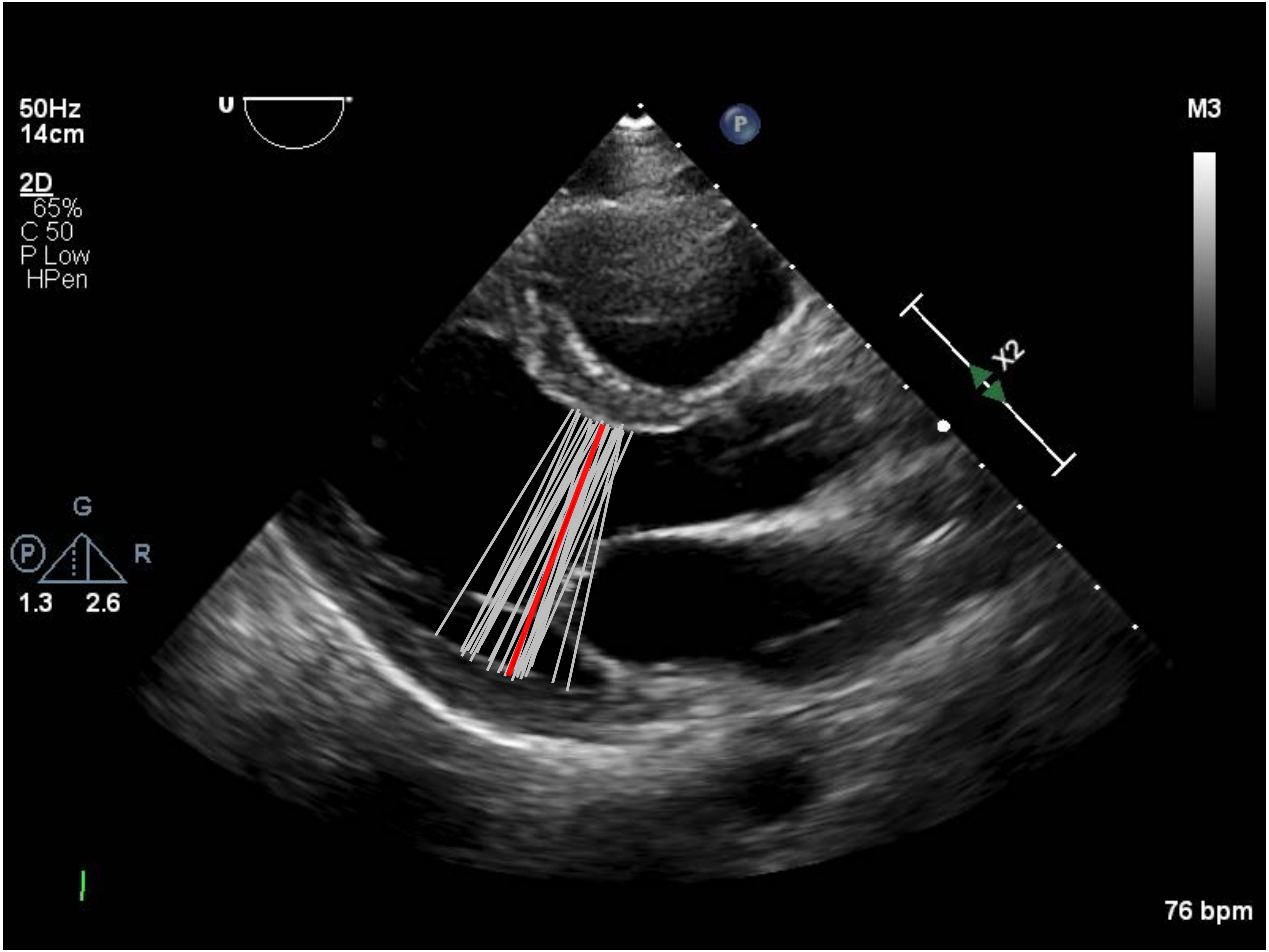

Pt 25 Diastole

Absolute difference between AI and expert consensus: 0.54 mm

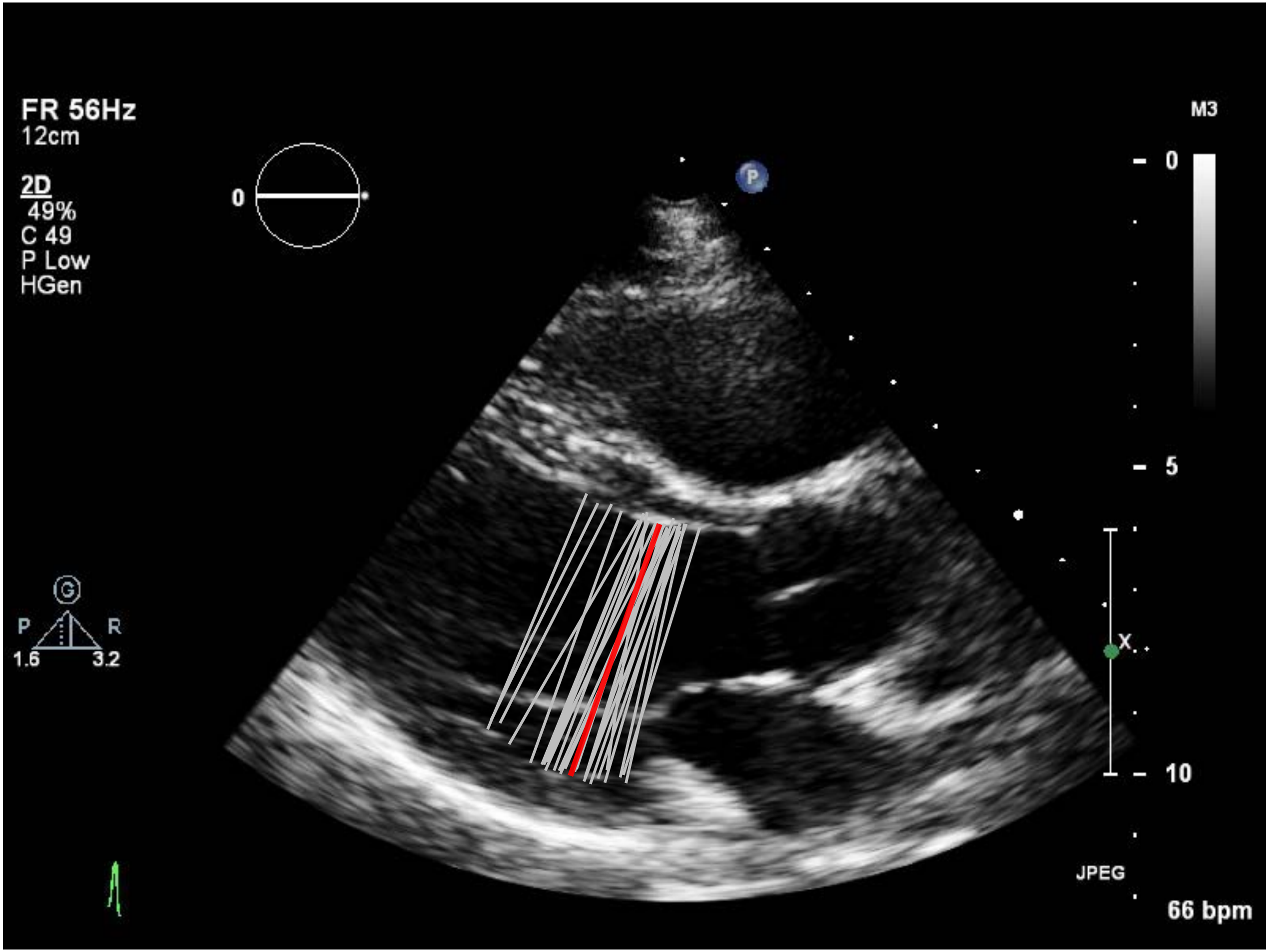

Pt 40 Diastole

Absolute difference between AI and expert consensus: 0.55 mm

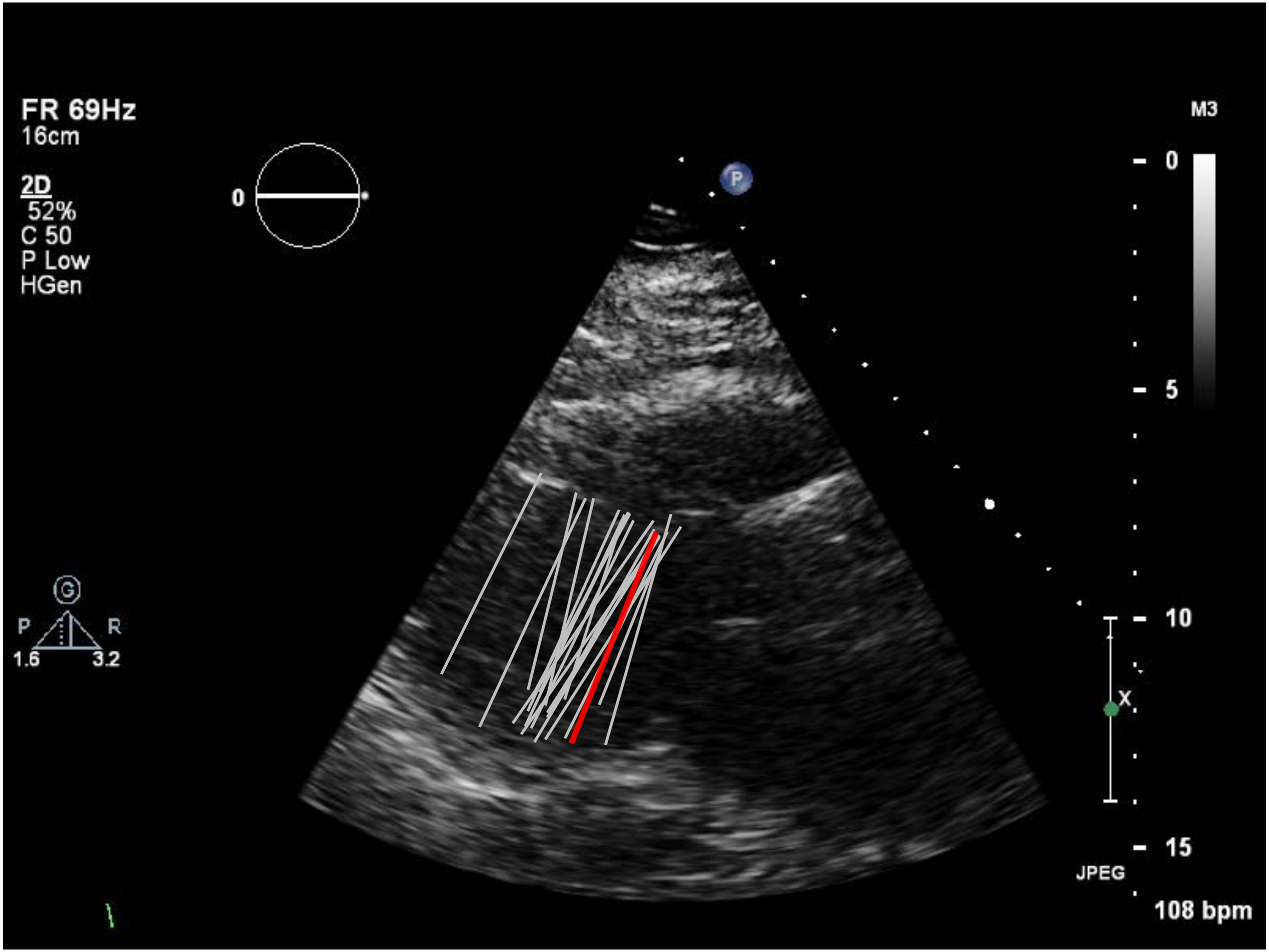

Pt 66 Diastole

Absolute difference between AI and expert consensus: 0.57 mm

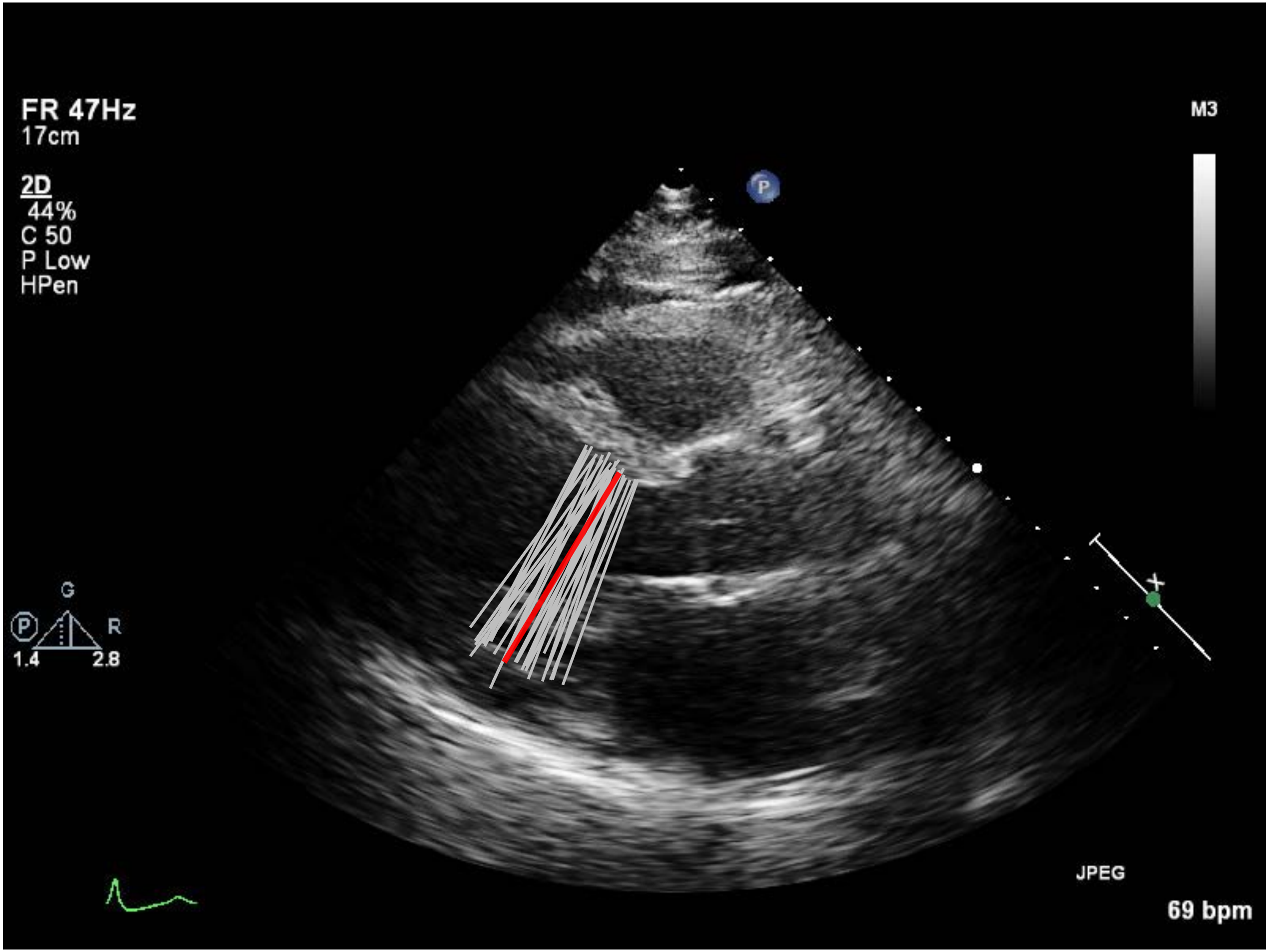

Pt 34 Systole

Absolute difference between AI and expert consensus: 0.57 mm

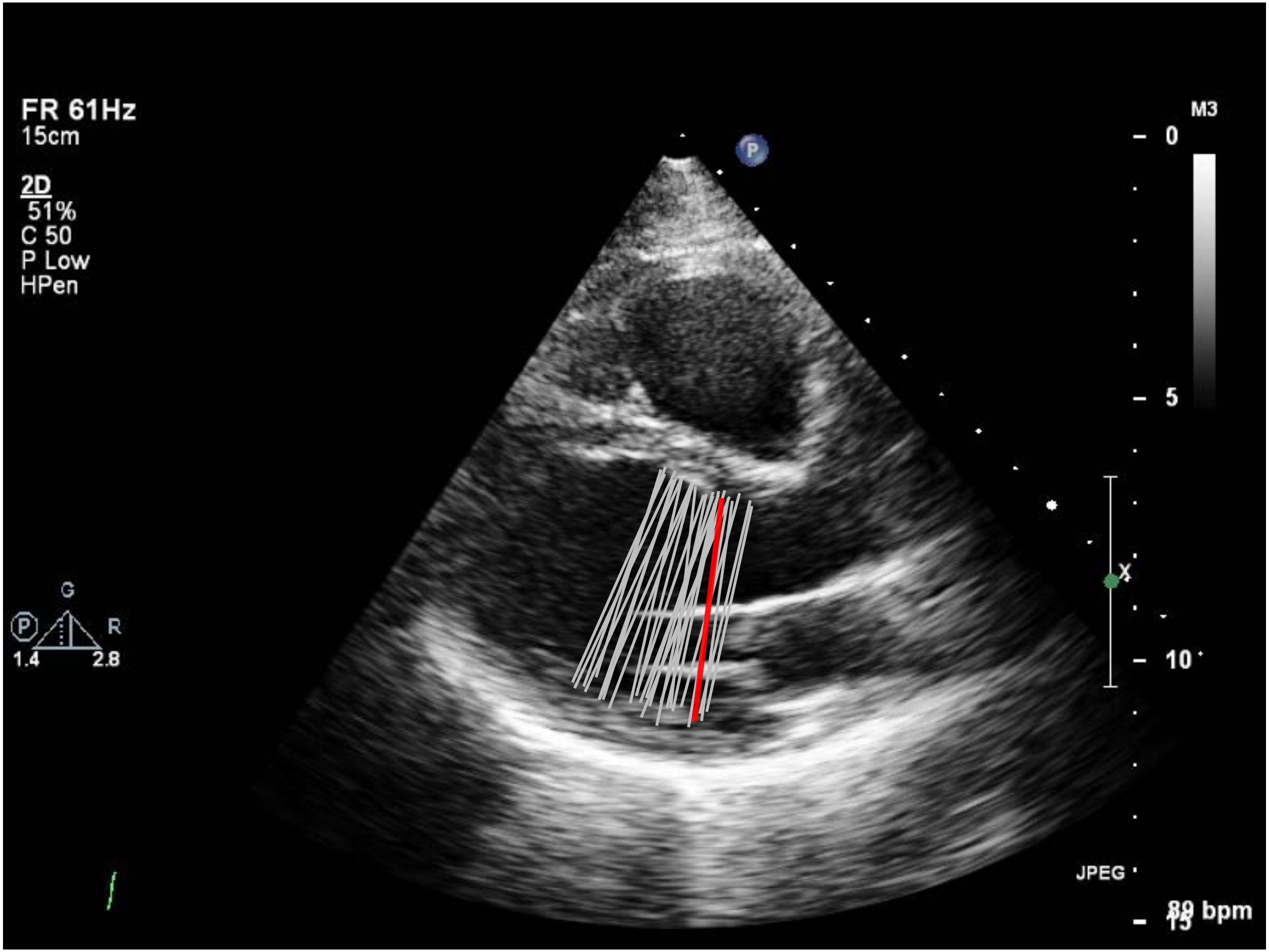

Pt 58 Diastole

Absolute difference between AI and expert consensus: 0.58 mm

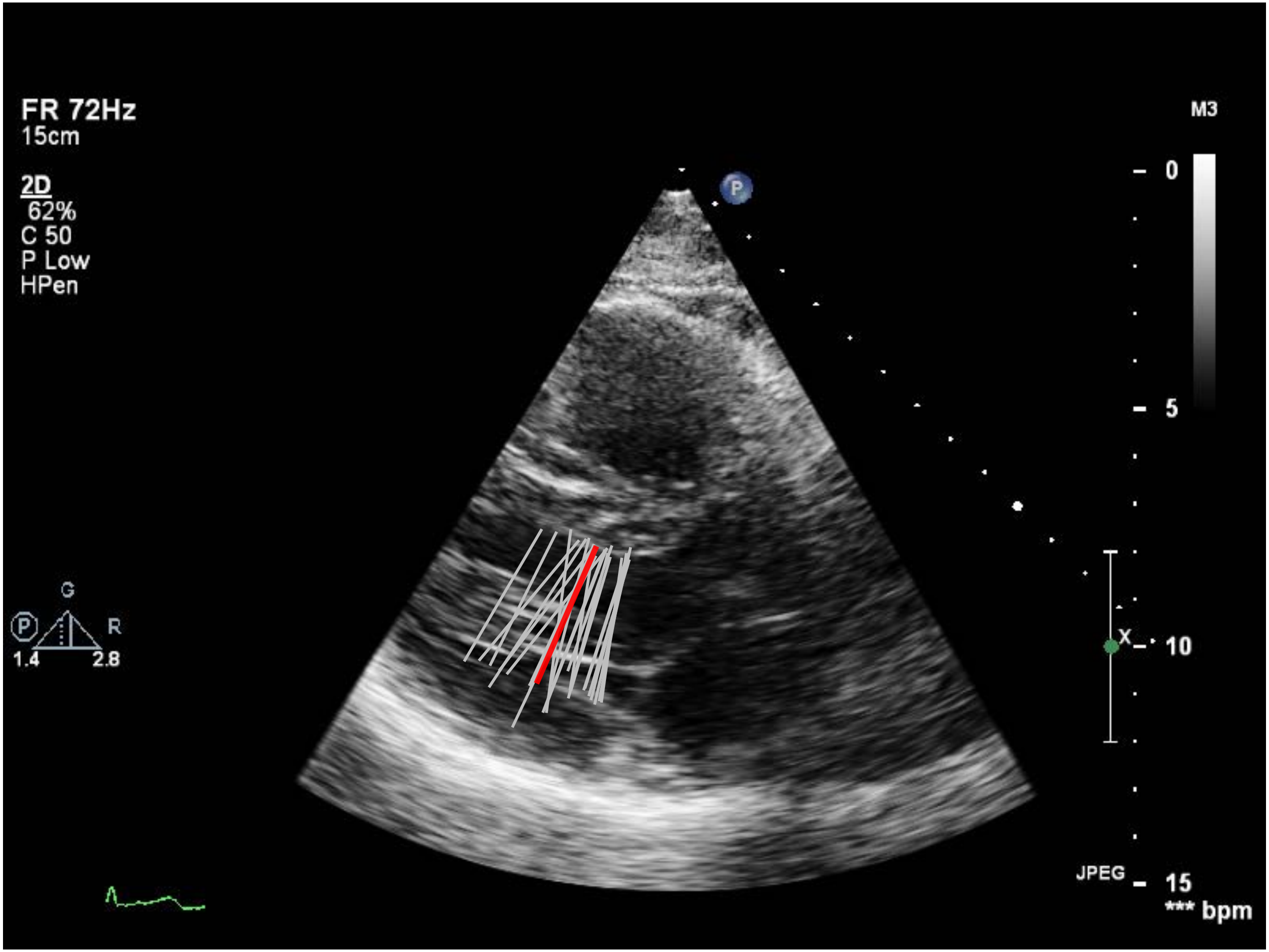

Pt 94 Systole

Absolute difference between AI and expert consensus: 0.58 mm

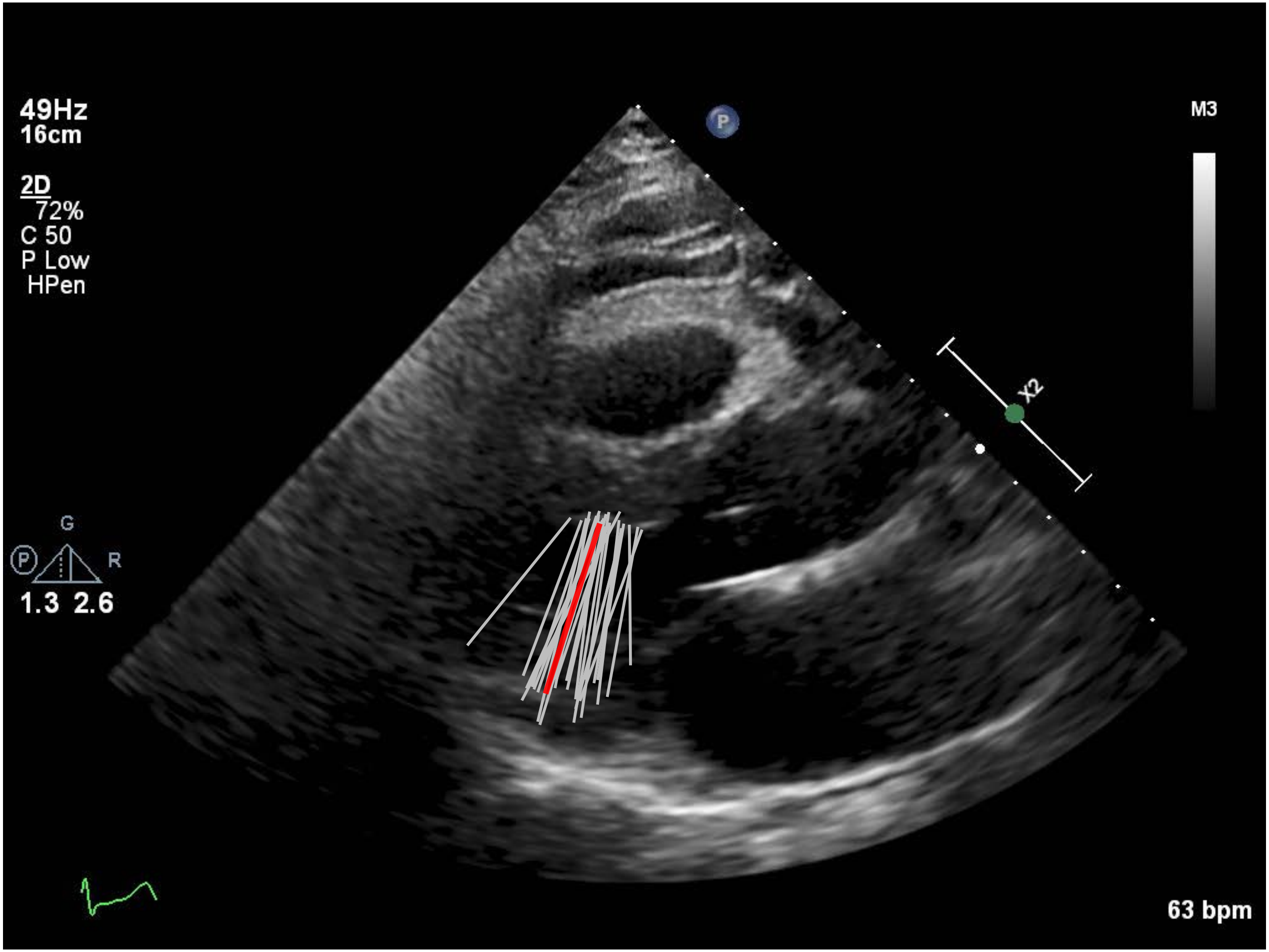

Pt 24 Systole

Absolute difference between AI and expert consensus: 0.61 mm

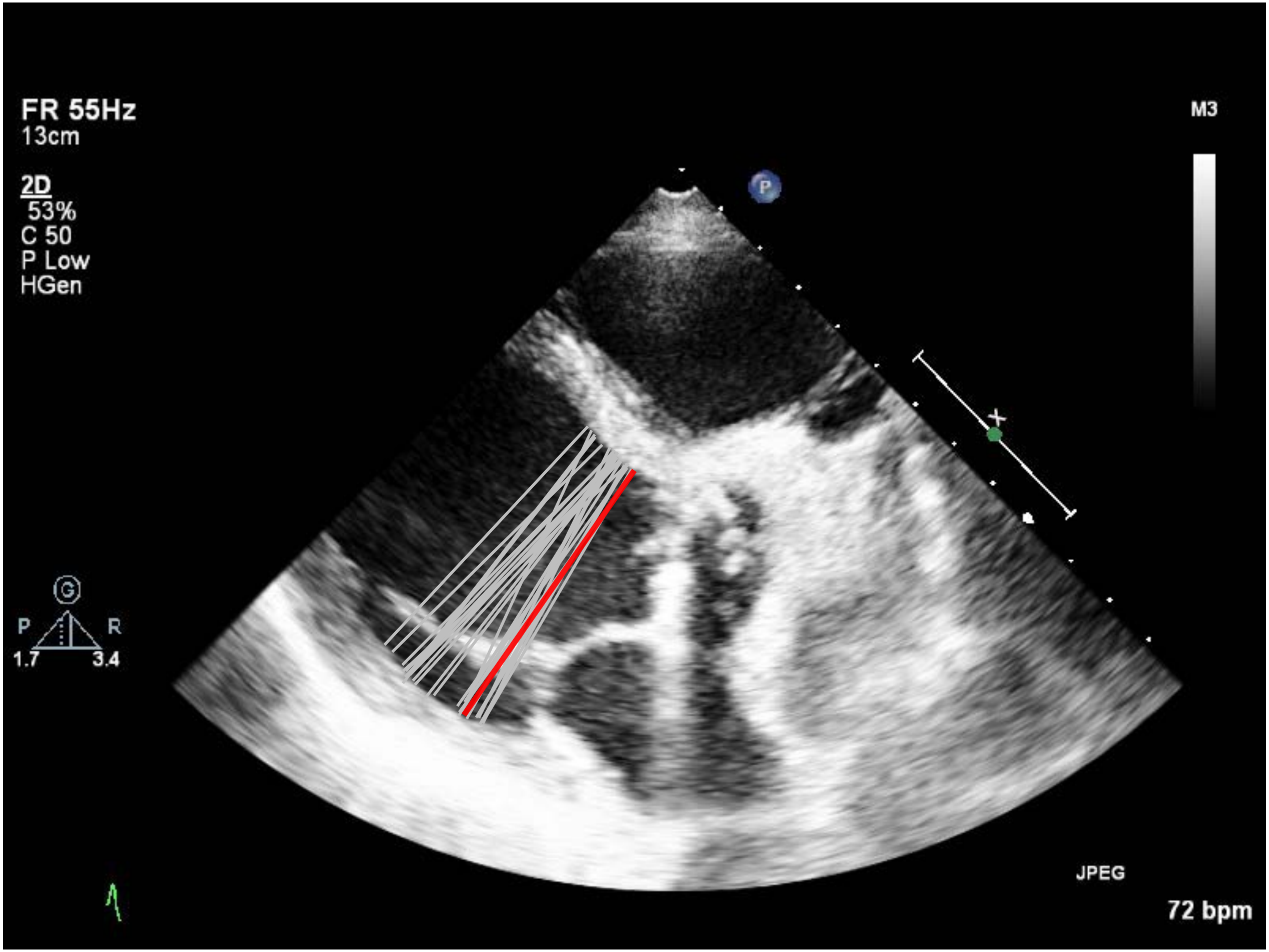

Pt 28 Diastole

Absolute difference between AI and expert consensus: 0.61 mm

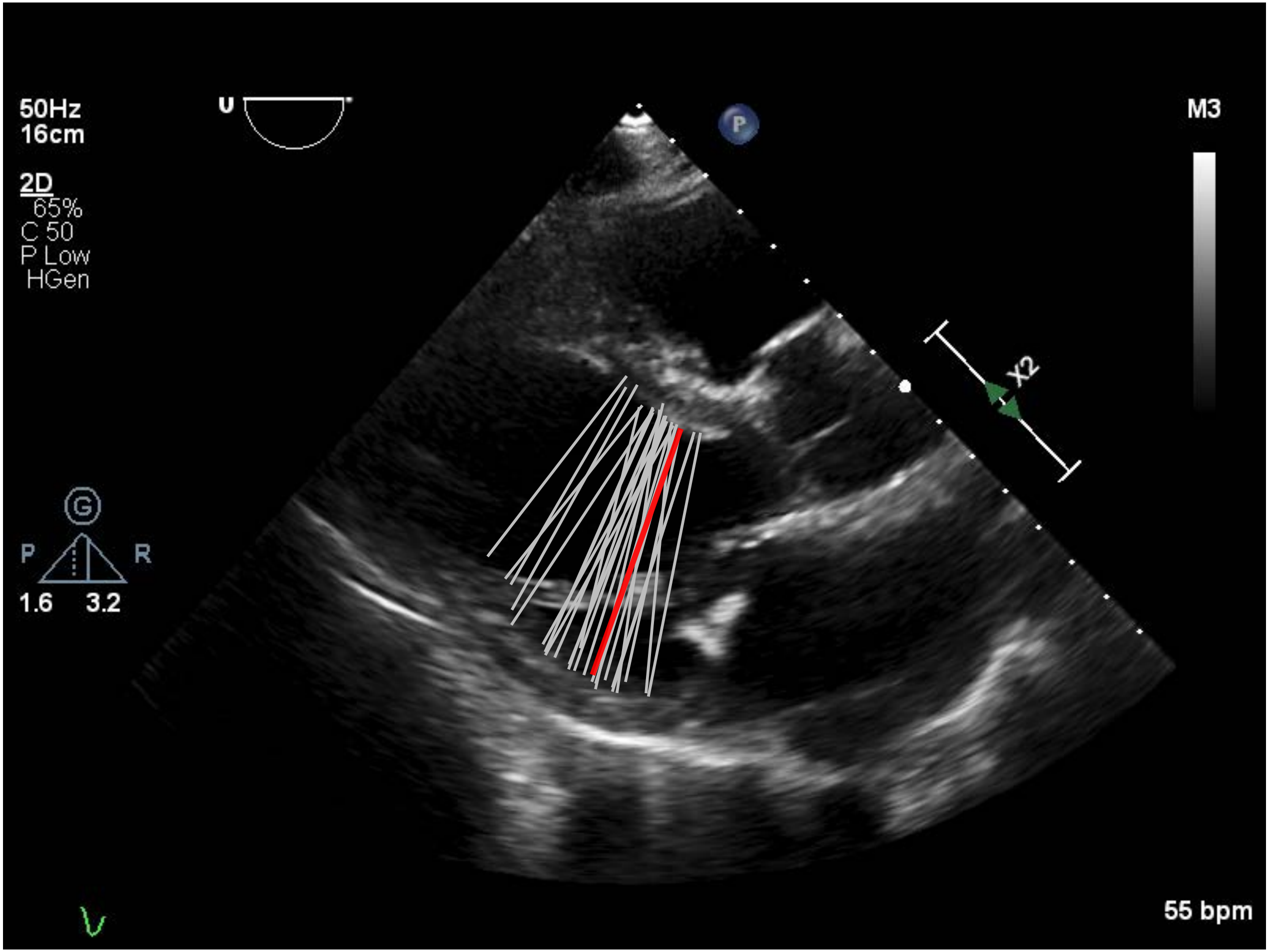

Pt 78 Diastole

Absolute difference between AI and expert consensus: 0.66 mm

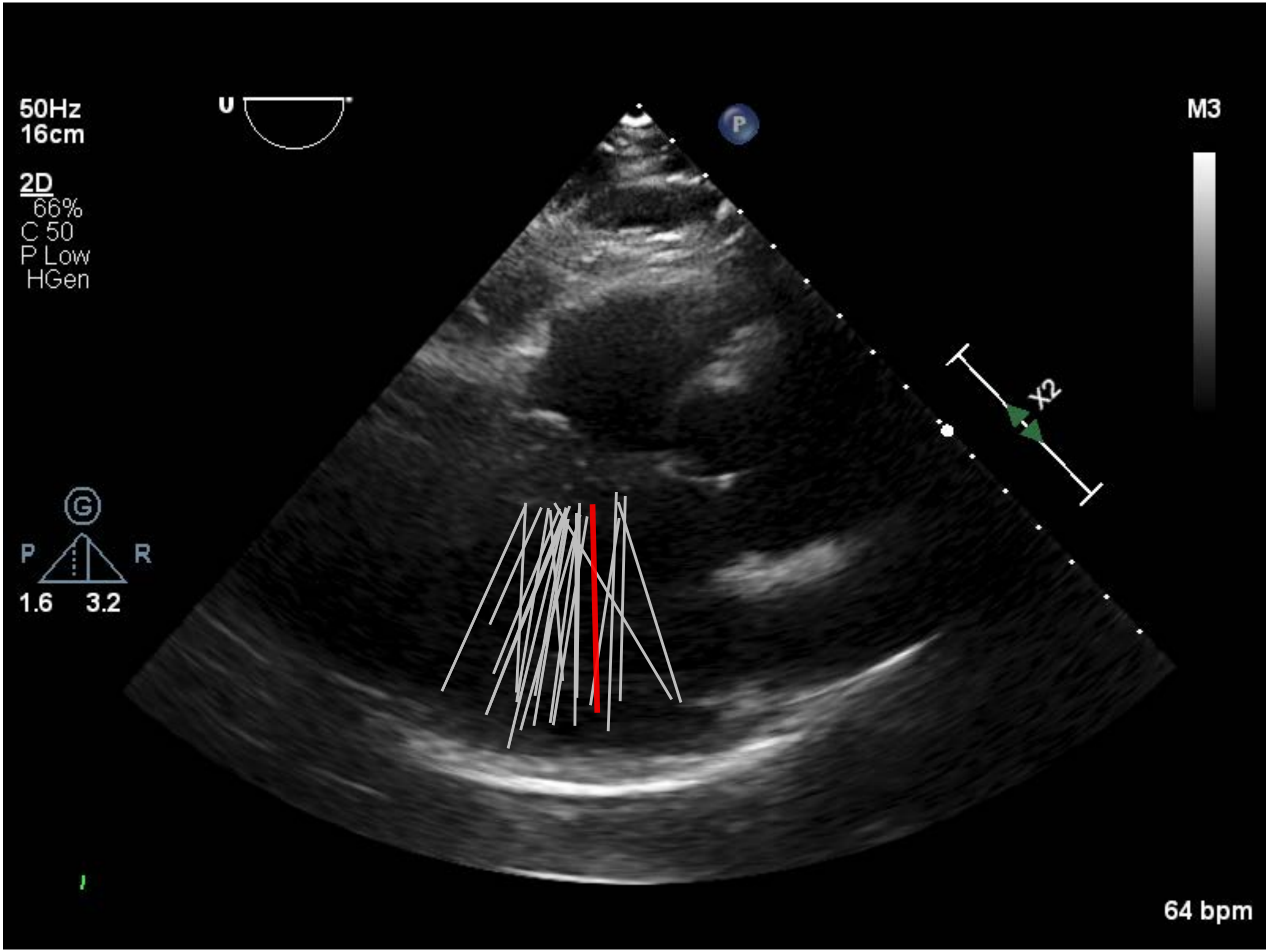

Pt 20 Systole

Absolute difference between AI and expert consensus: 0.67 mm

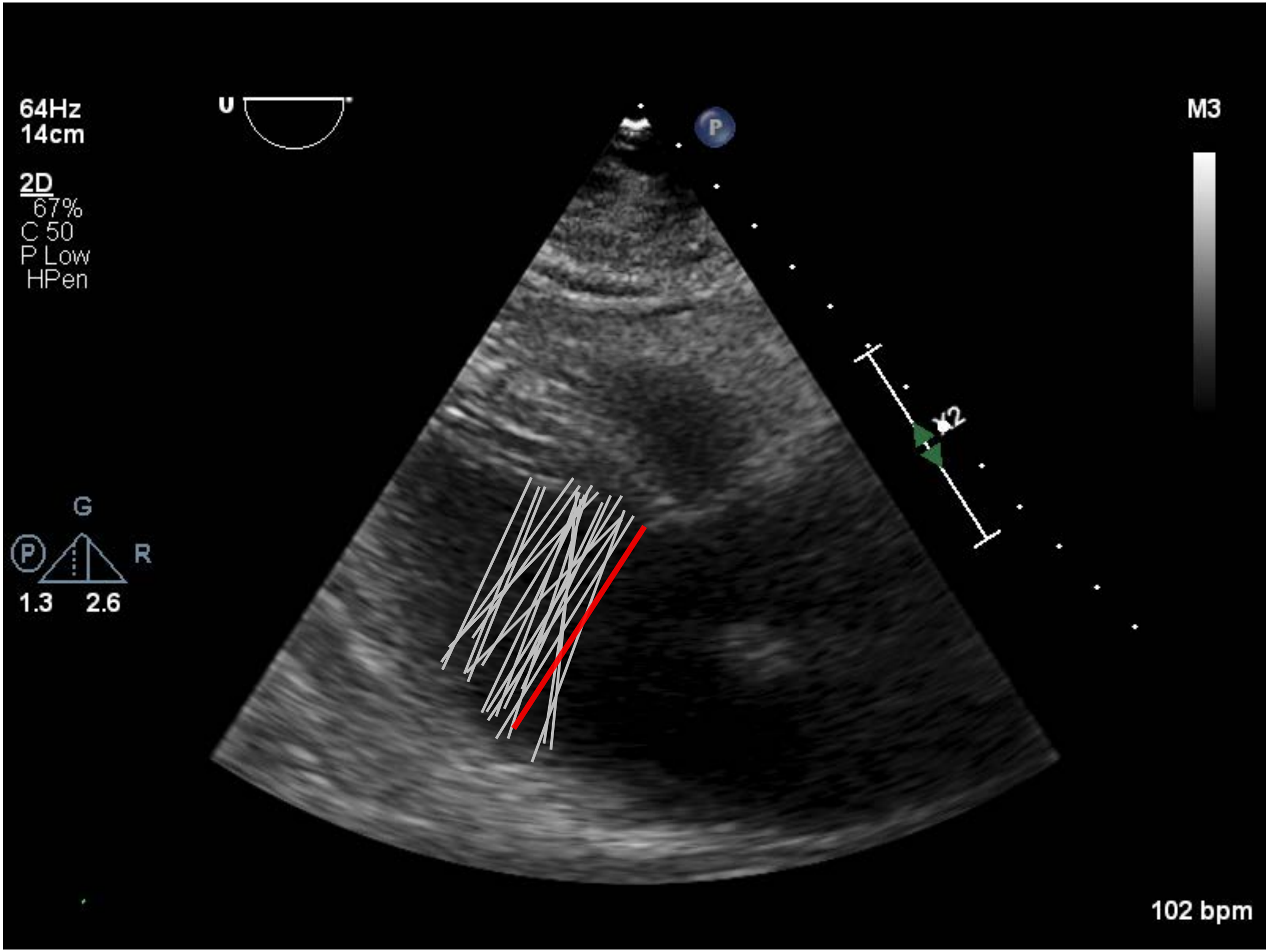

Pt 99 Diastole

Absolute difference between AI and expert consensus: 0.67 mm

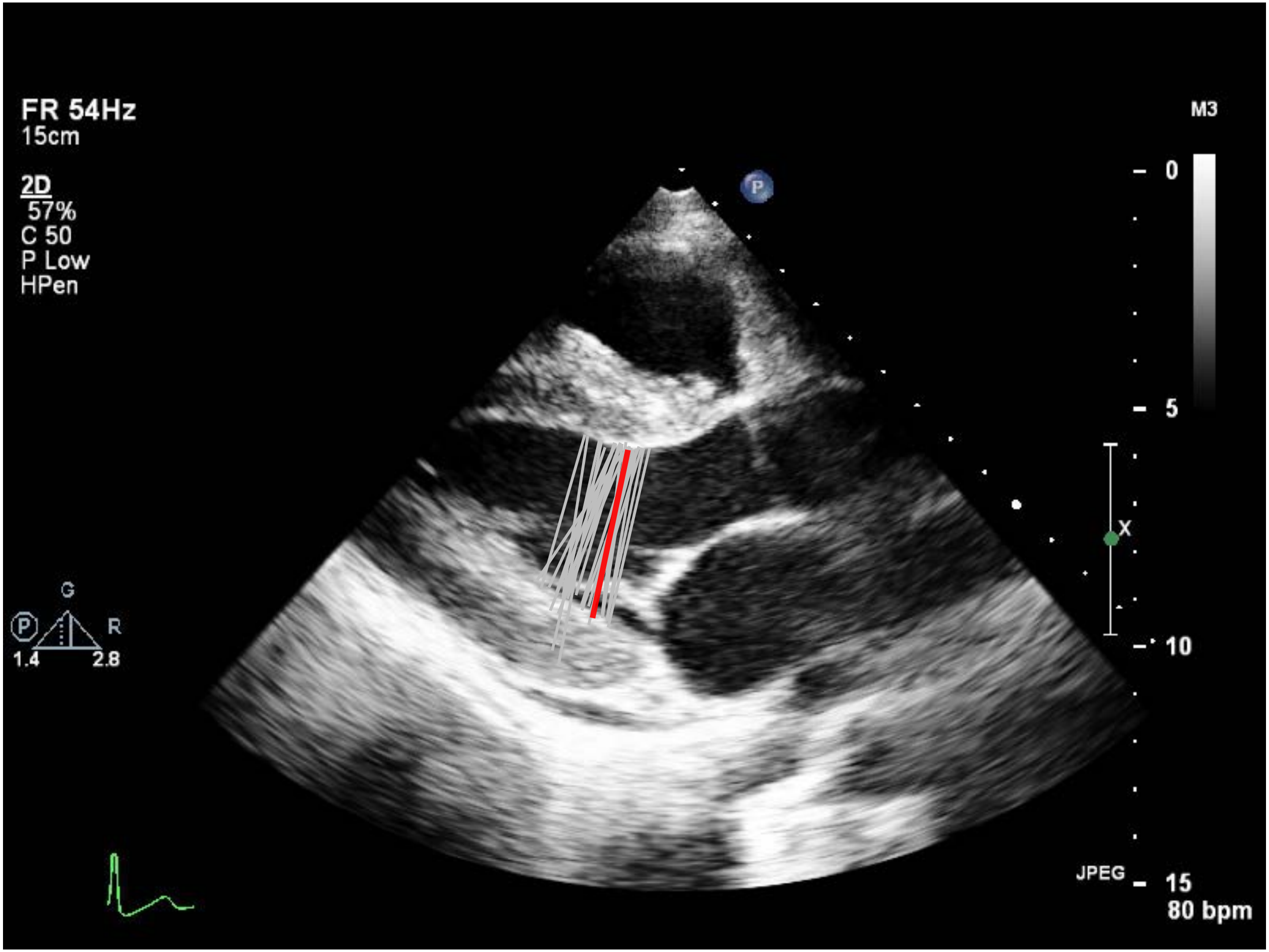

Pt 82 Systole

Absolute difference between AI and expert consensus: 0.7 mm

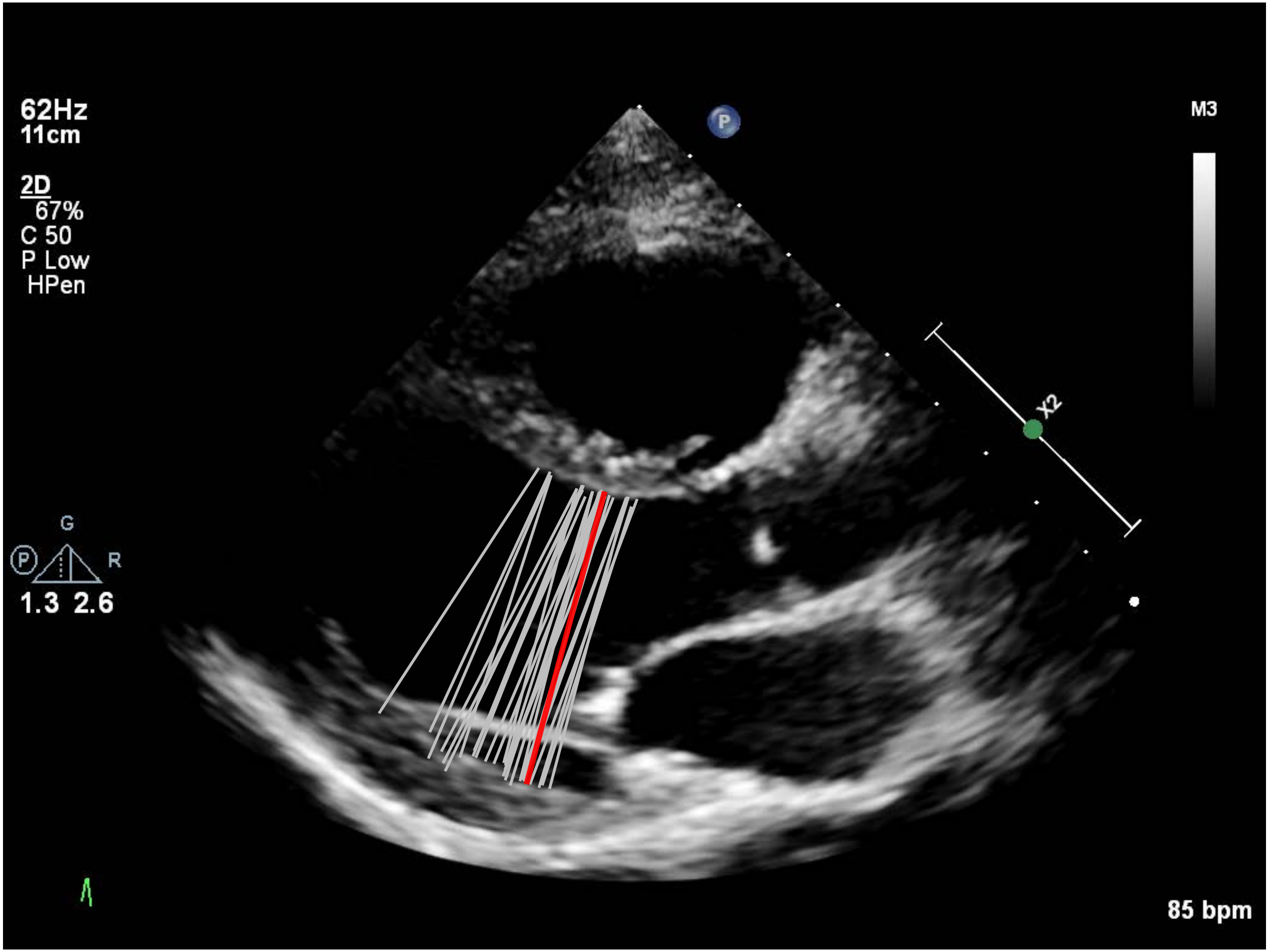

Pt 2 Diastole
Absolute difference between AI and expert consensus: 0.71 mm

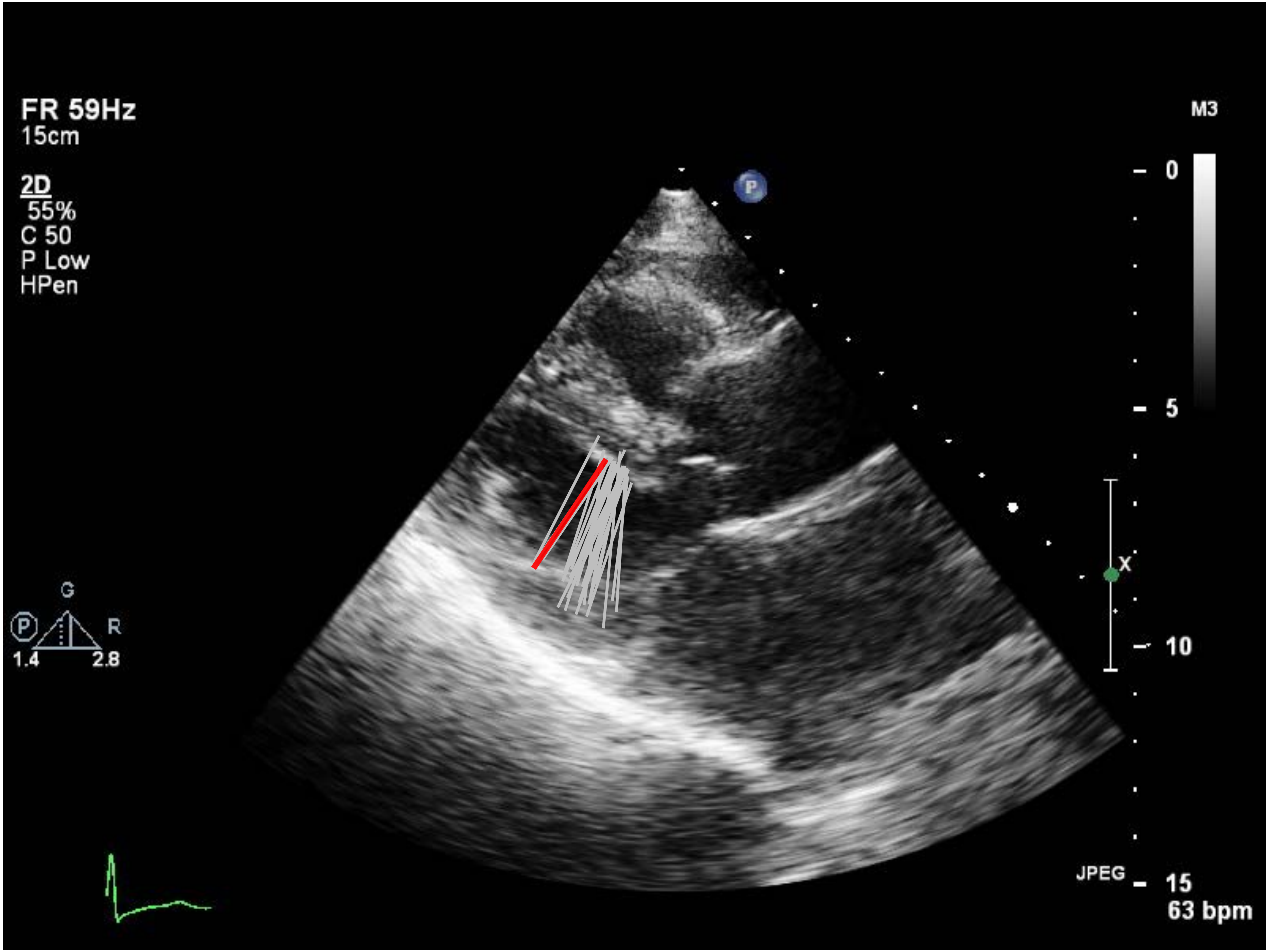

Pt 45 Systole

Absolute difference between AI and expert consensus: 0.72 mm

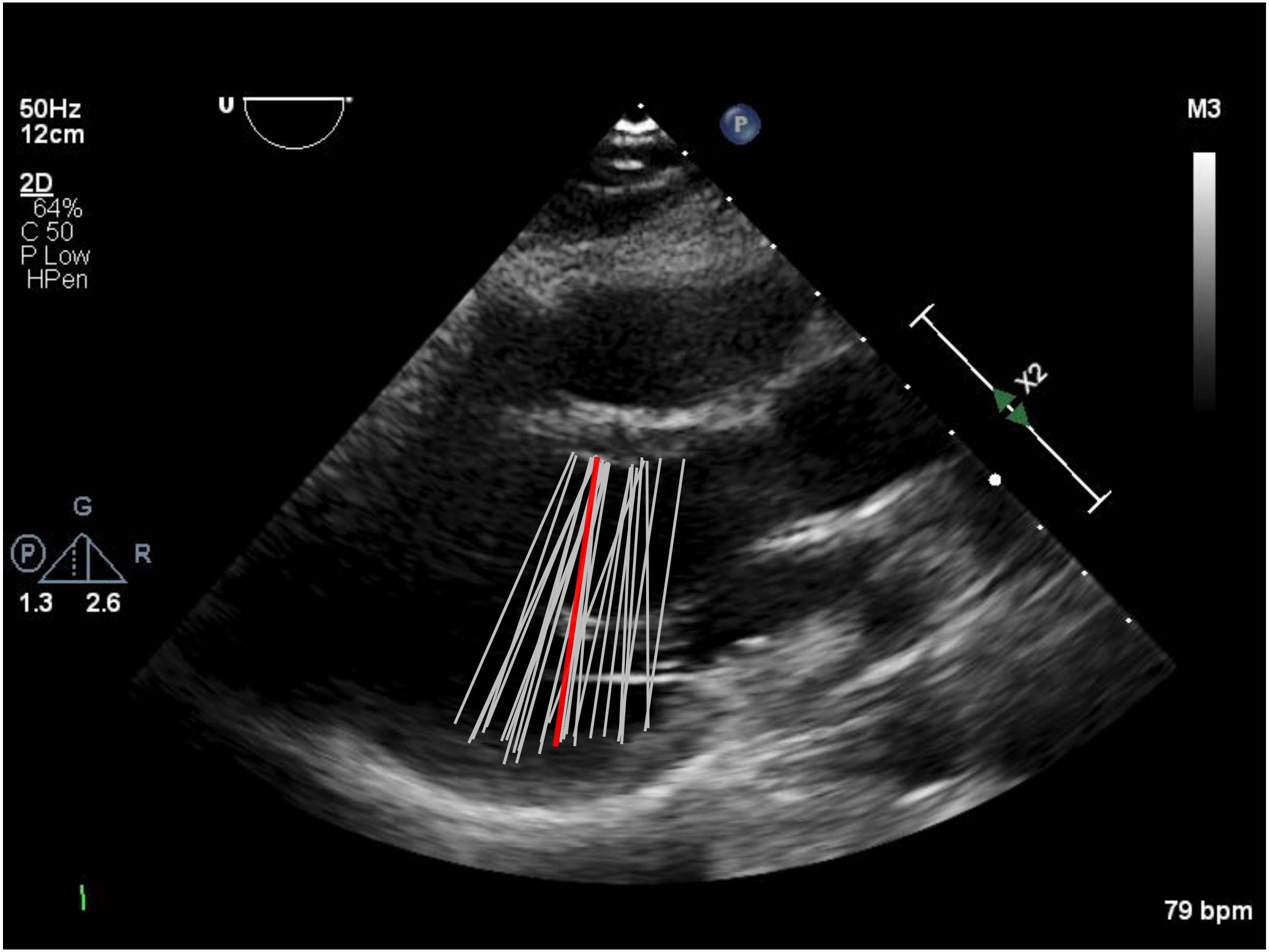

Pt 55 Diastole

Absolute difference between AI and expert consensus: 0.72 mm

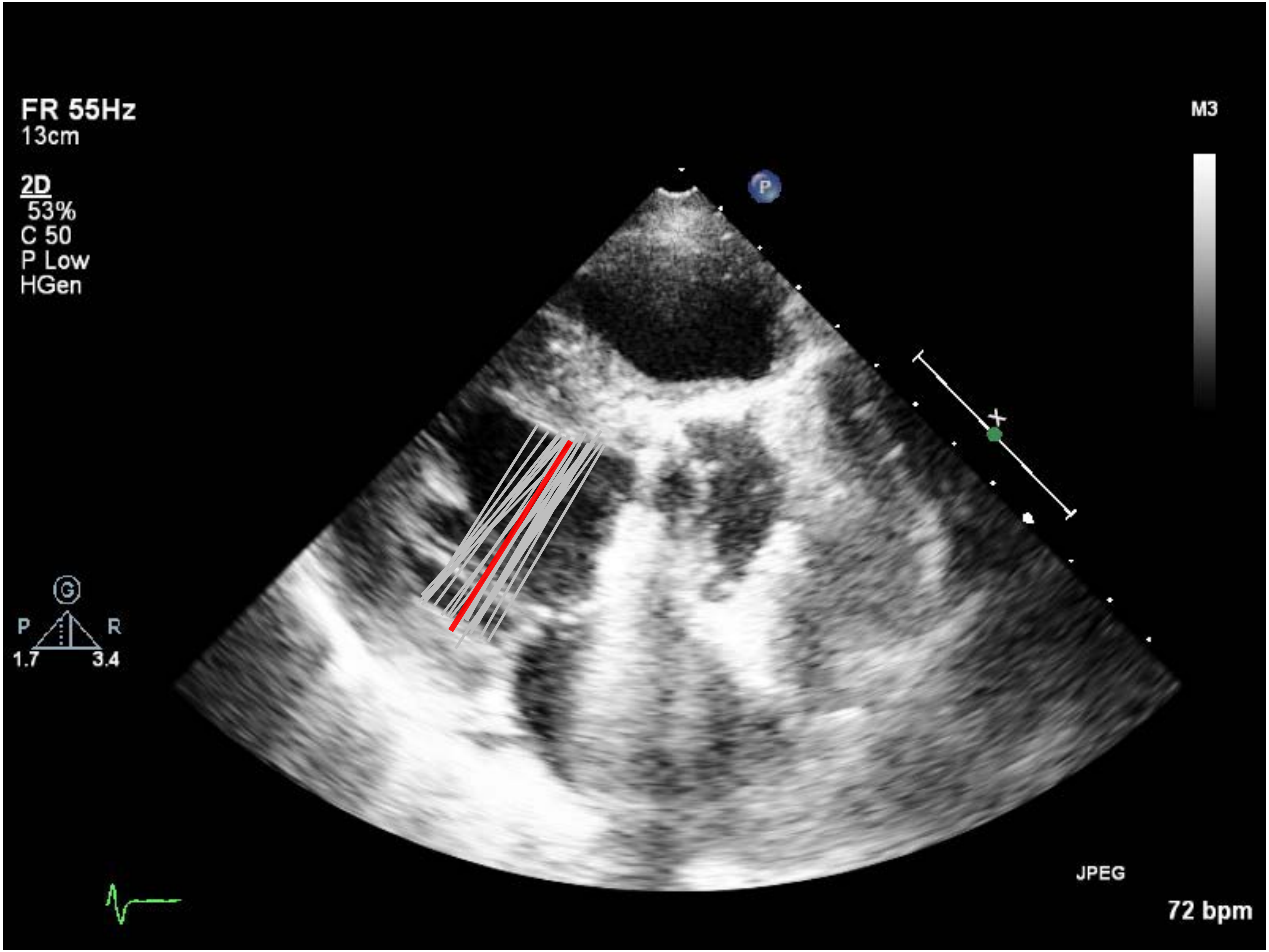

Pt 28 Systole

Absolute difference between AI and expert consensus: 0.73 mm

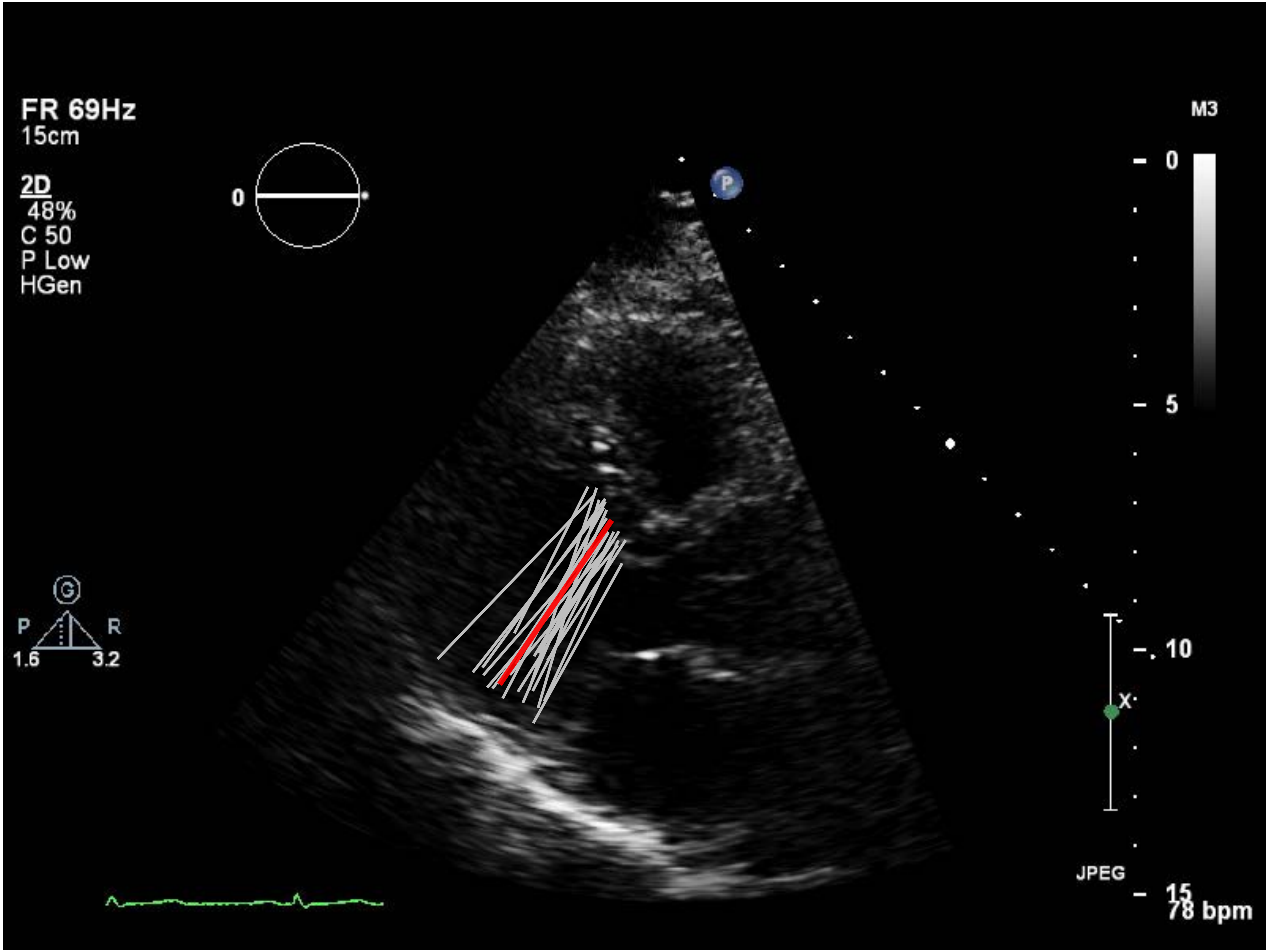

Pt 59 Systole

Absolute difference between AI and expert consensus: 0.73 mm

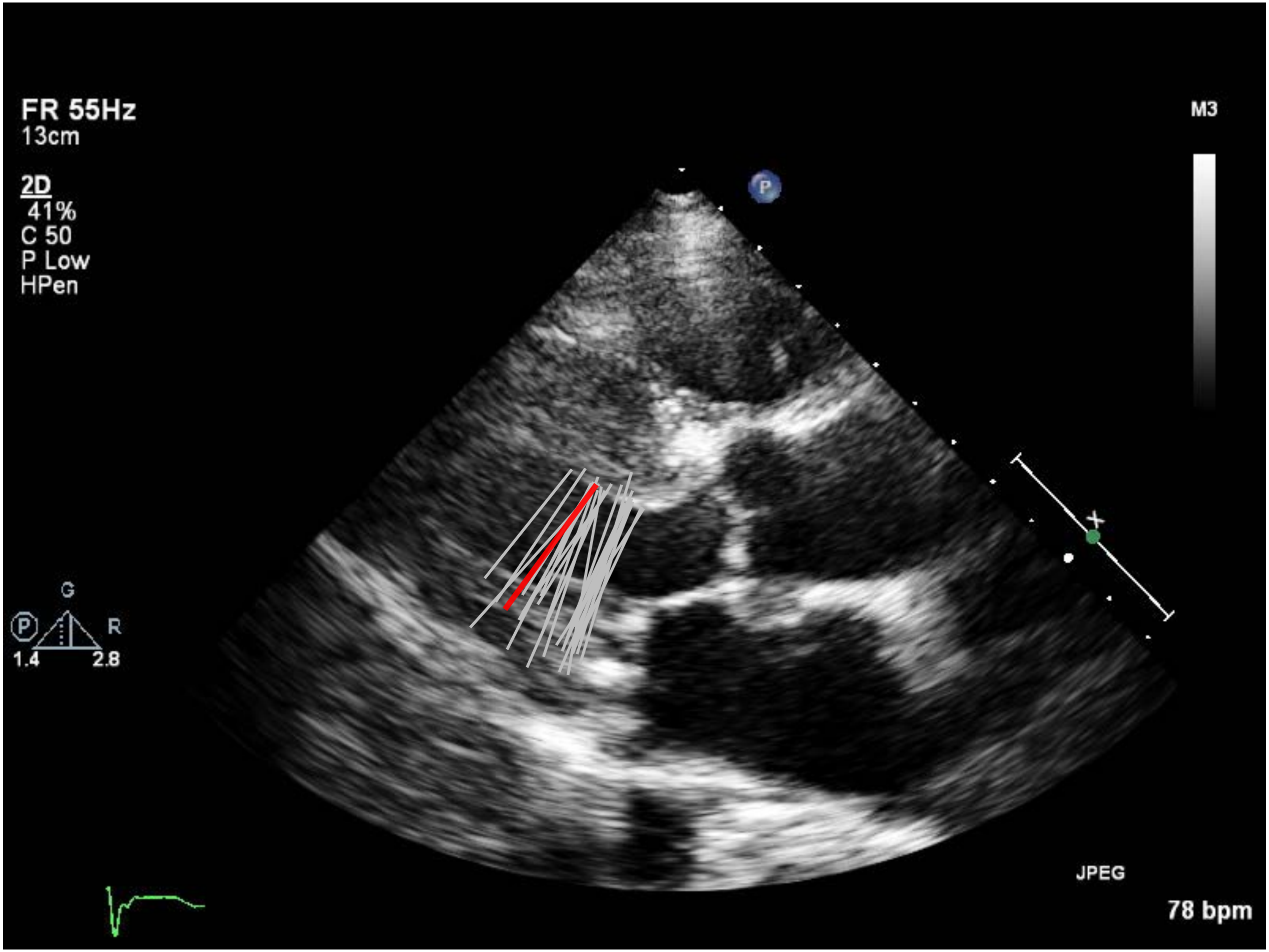

Pt 31 Systole

Absolute difference between AI and expert consensus: 0.73 mm

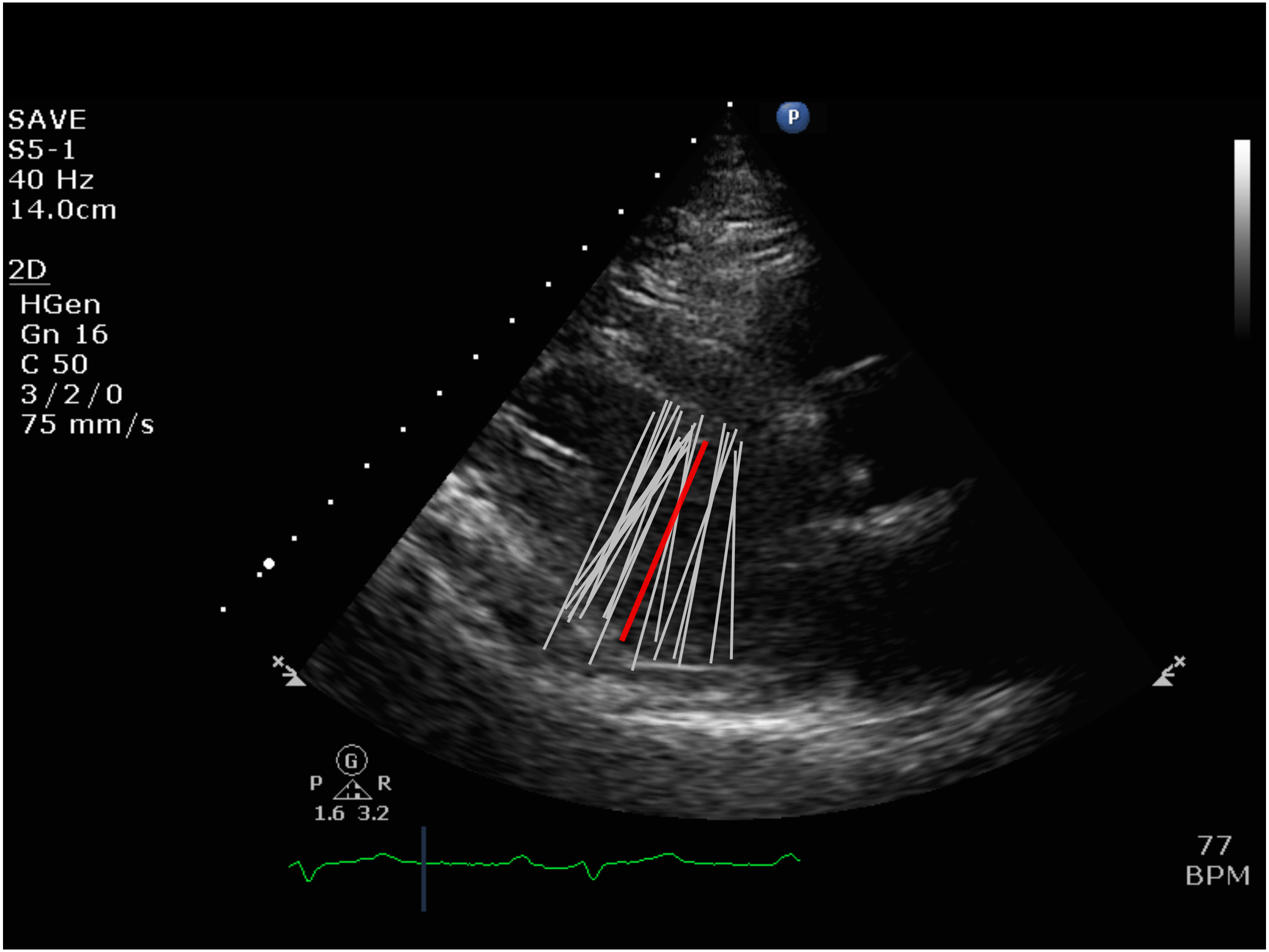

Pt 76 Systole

Absolute difference between AI and expert consensus: 0.75 mm

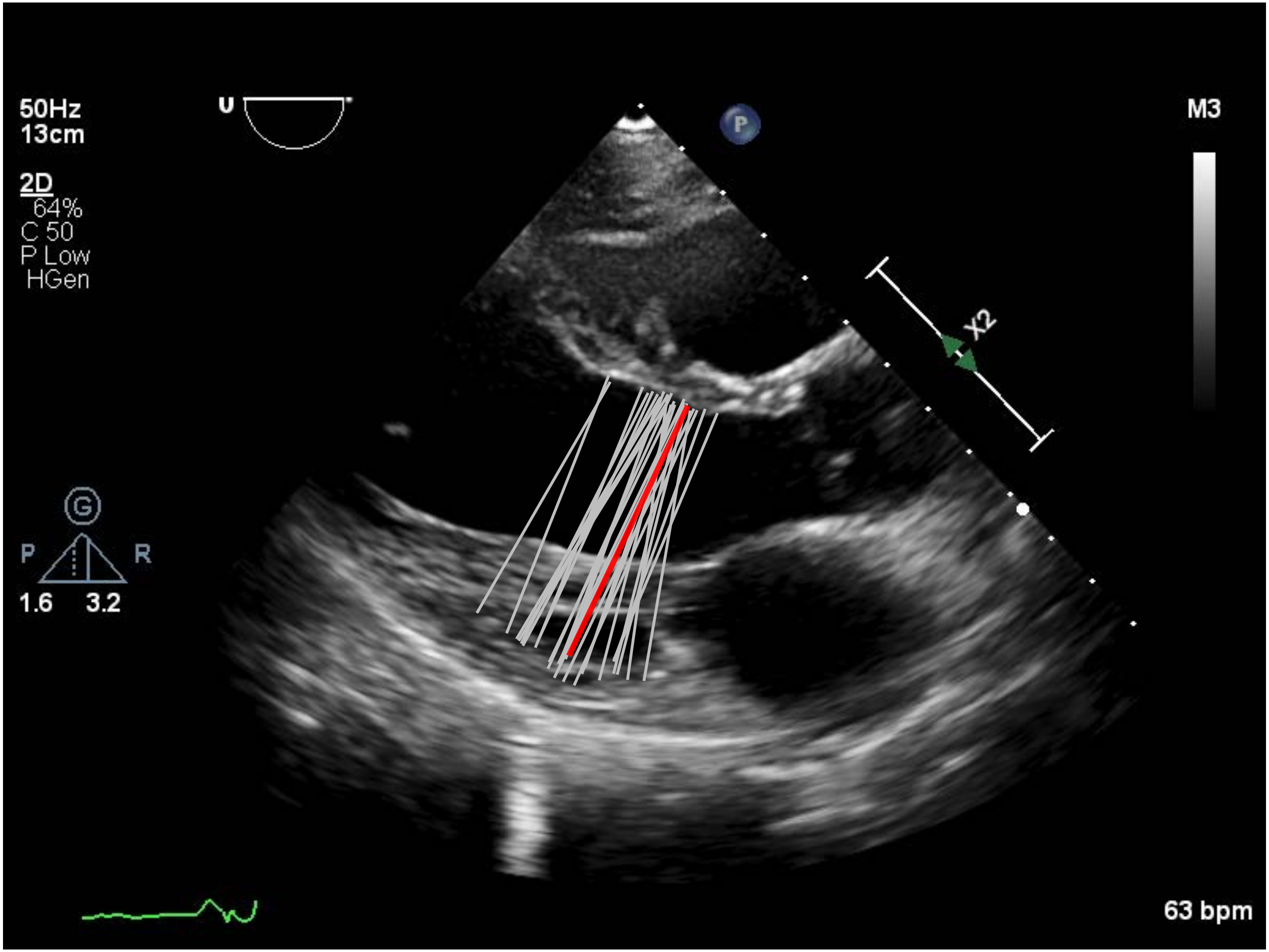

Pt 16 Diastole

Absolute difference between AI and expert consensus: 0.76 mm

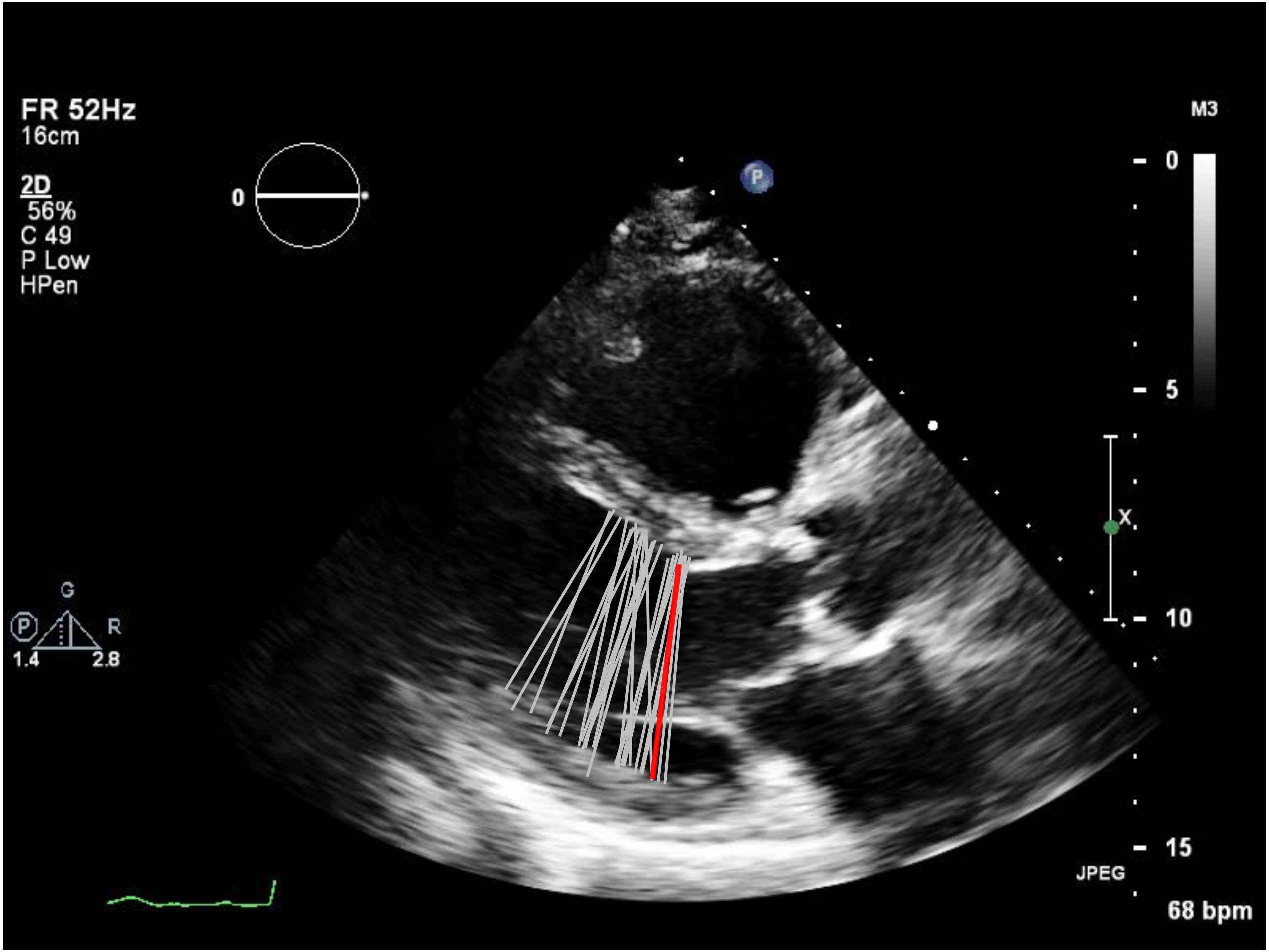

Pt 37 Diastole

Absolute difference between AI and expert consensus: 0.76 mm

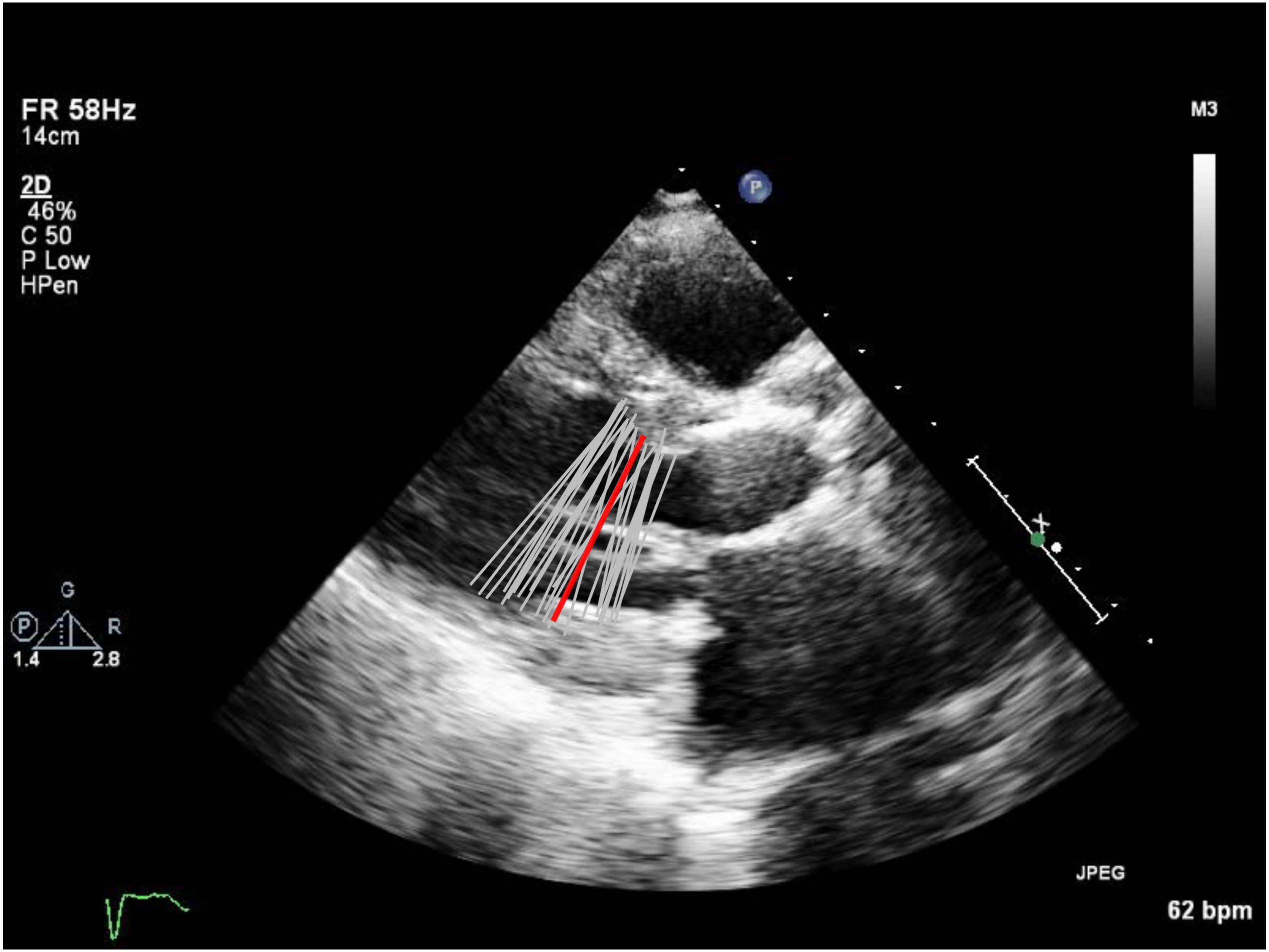

Pt 92 Systole

Absolute difference between AI and expert consensus: 0.78 mm

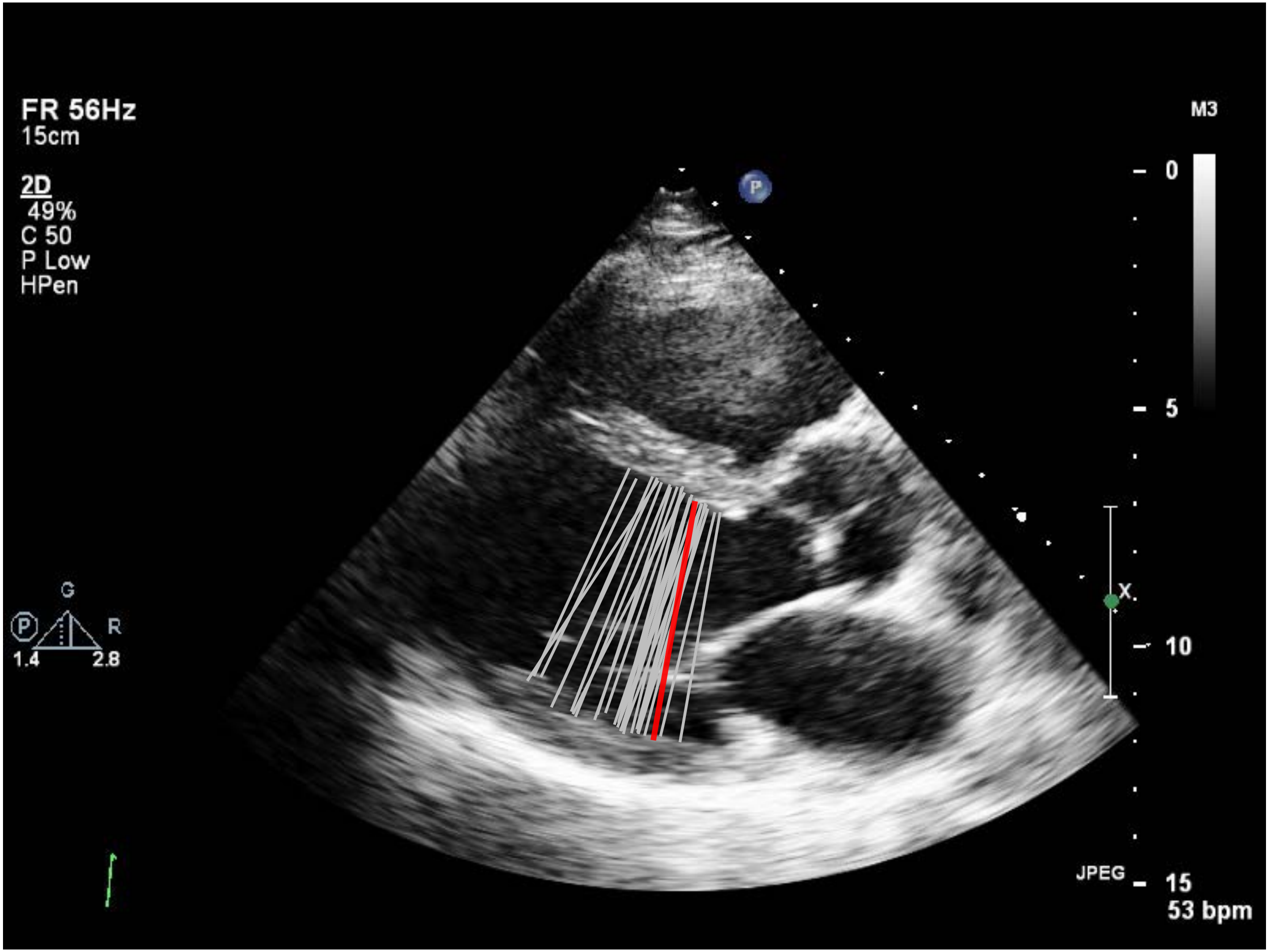

Pt 29 Diastole

Absolute difference between AI and expert consensus: 0.78 mm

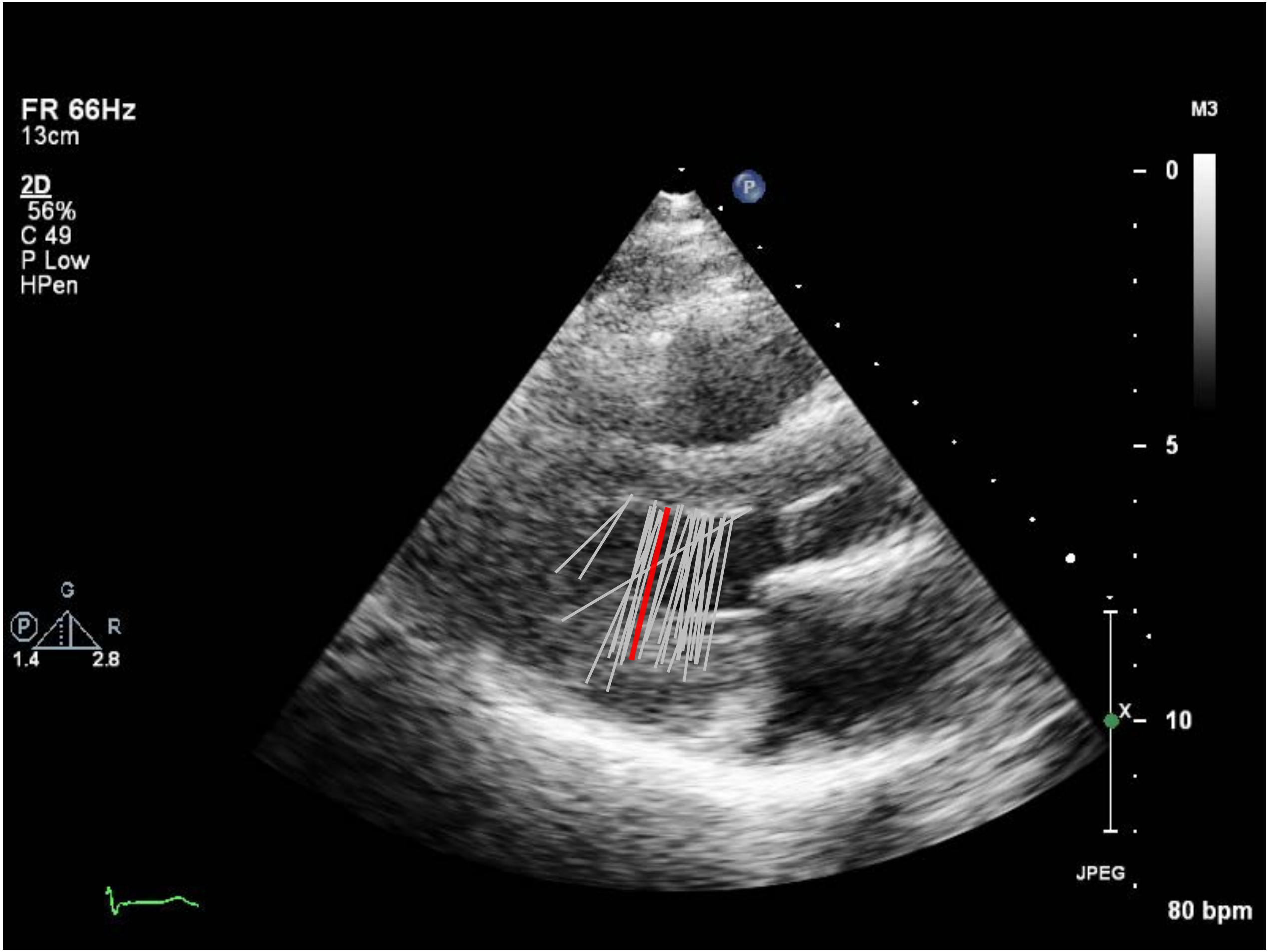

Pt 91 Systole

Absolute difference between AI and expert consensus: 0.79 mm

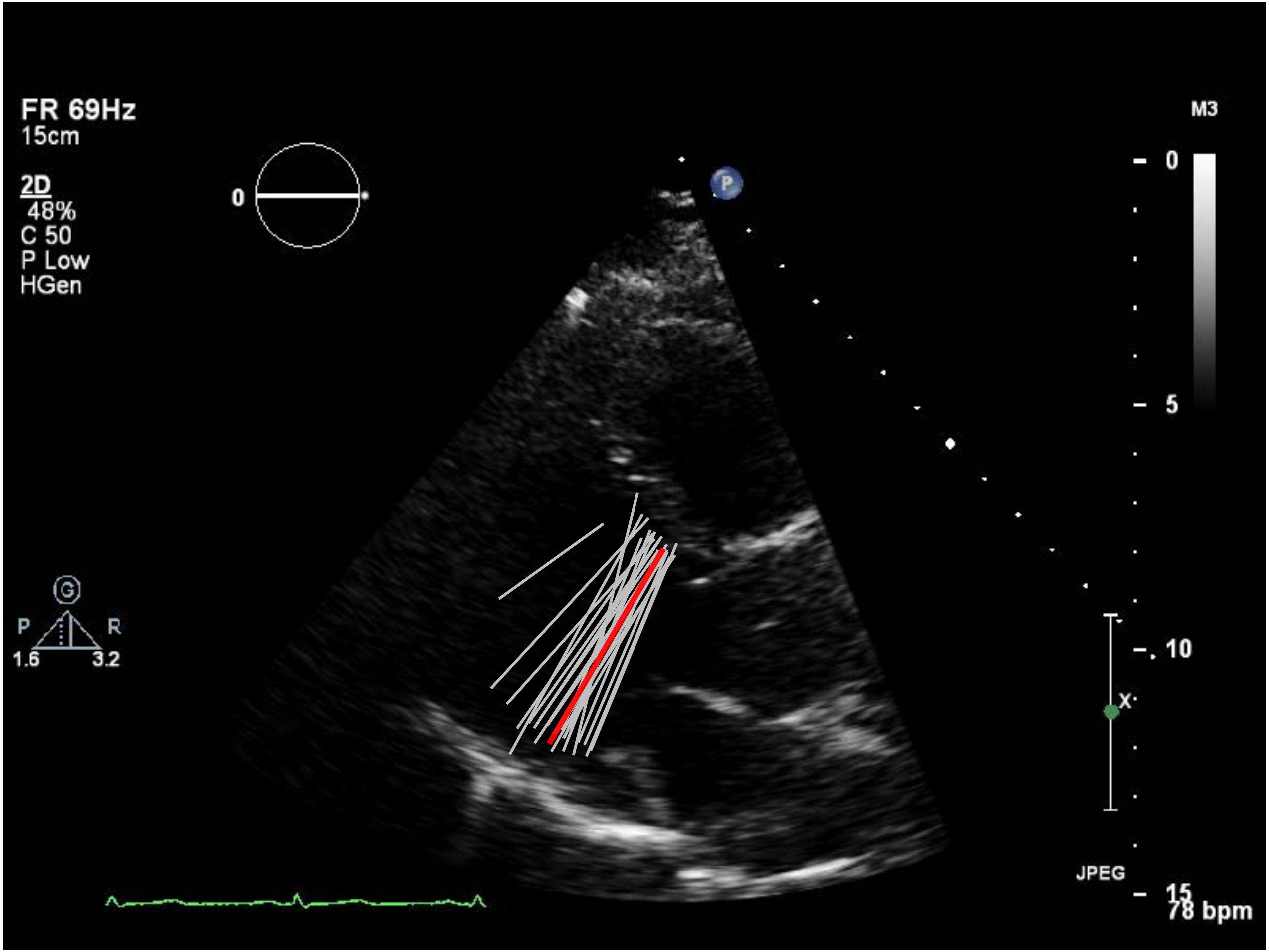

Pt 59 Diastole

Absolute difference between AI and expert consensus: 0.8 mm

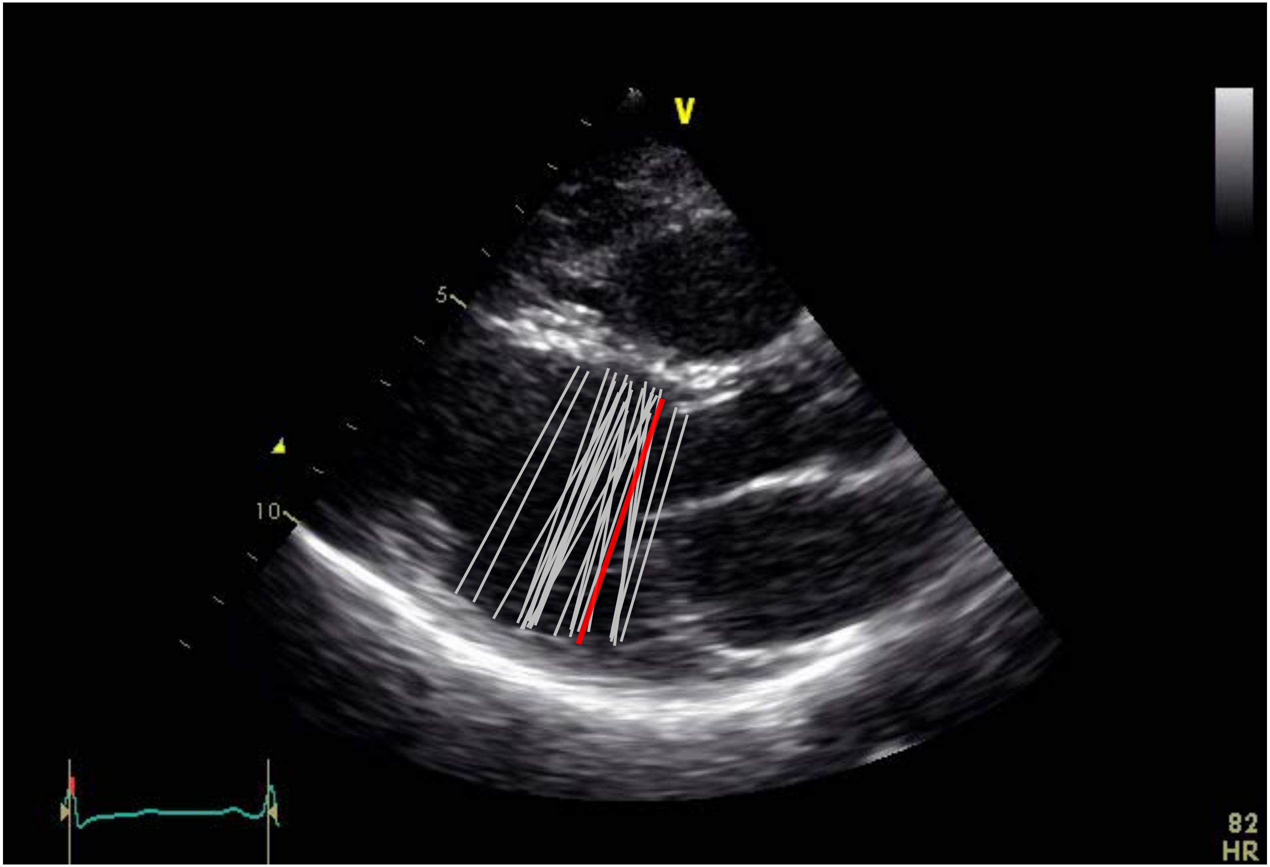

Pt 35 Diastole

Absolute difference between AI and expert consensus: 0.81 mm

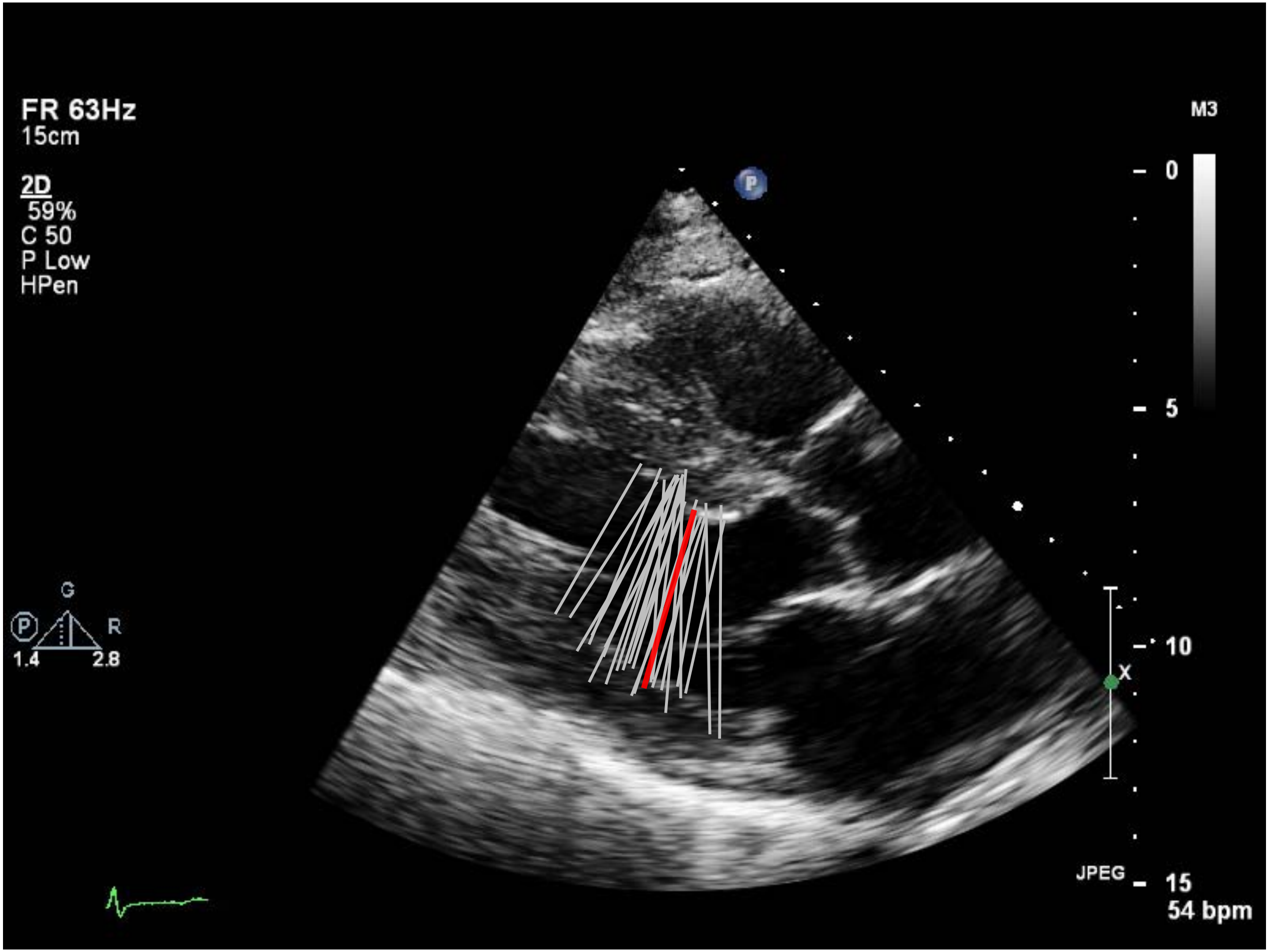

Pt 69 Systole

Absolute difference between AI and expert consensus: 0.81 mm

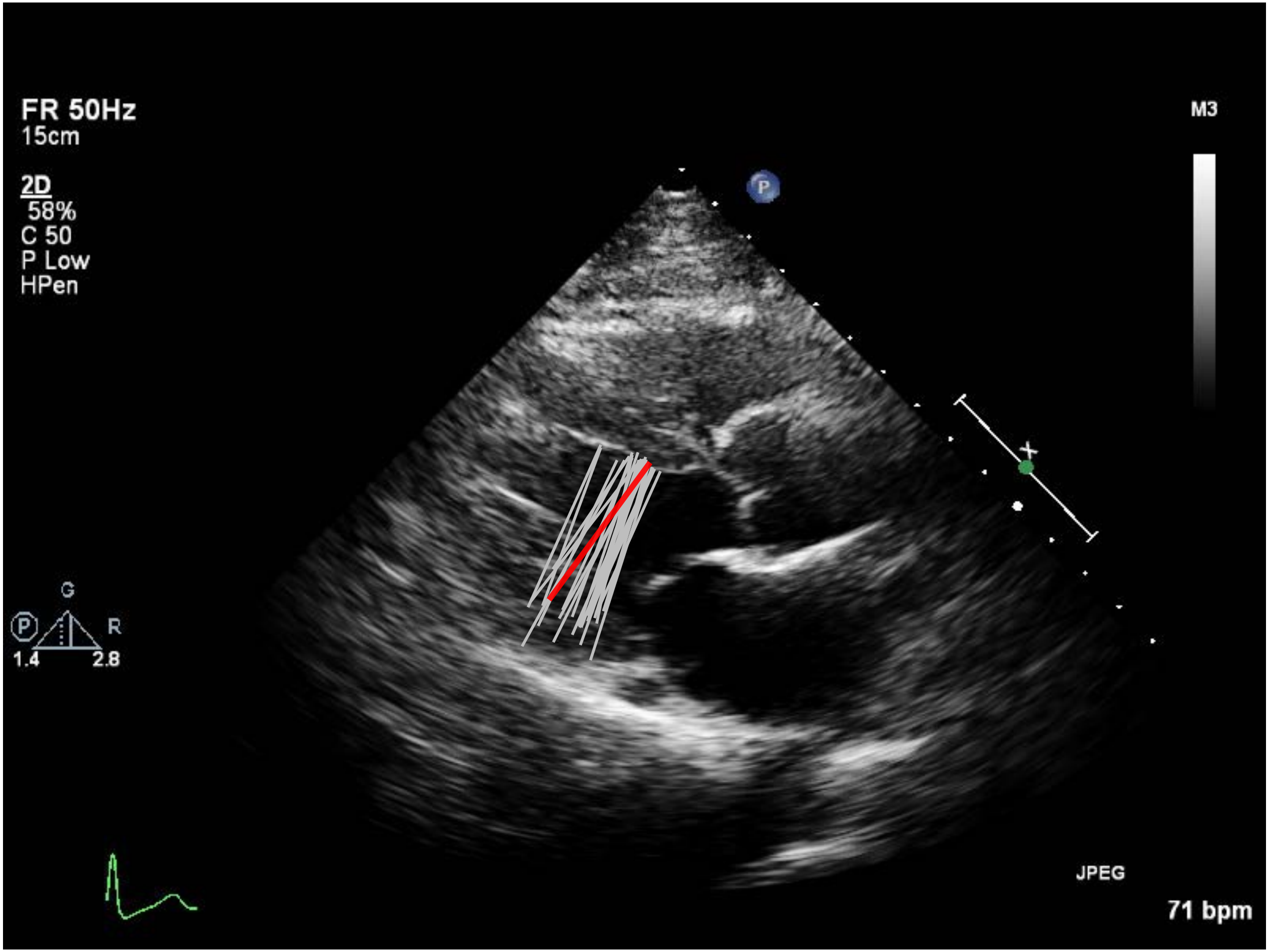

Pt 77 Systole

Absolute difference between AI and expert consensus: 0.82 mm

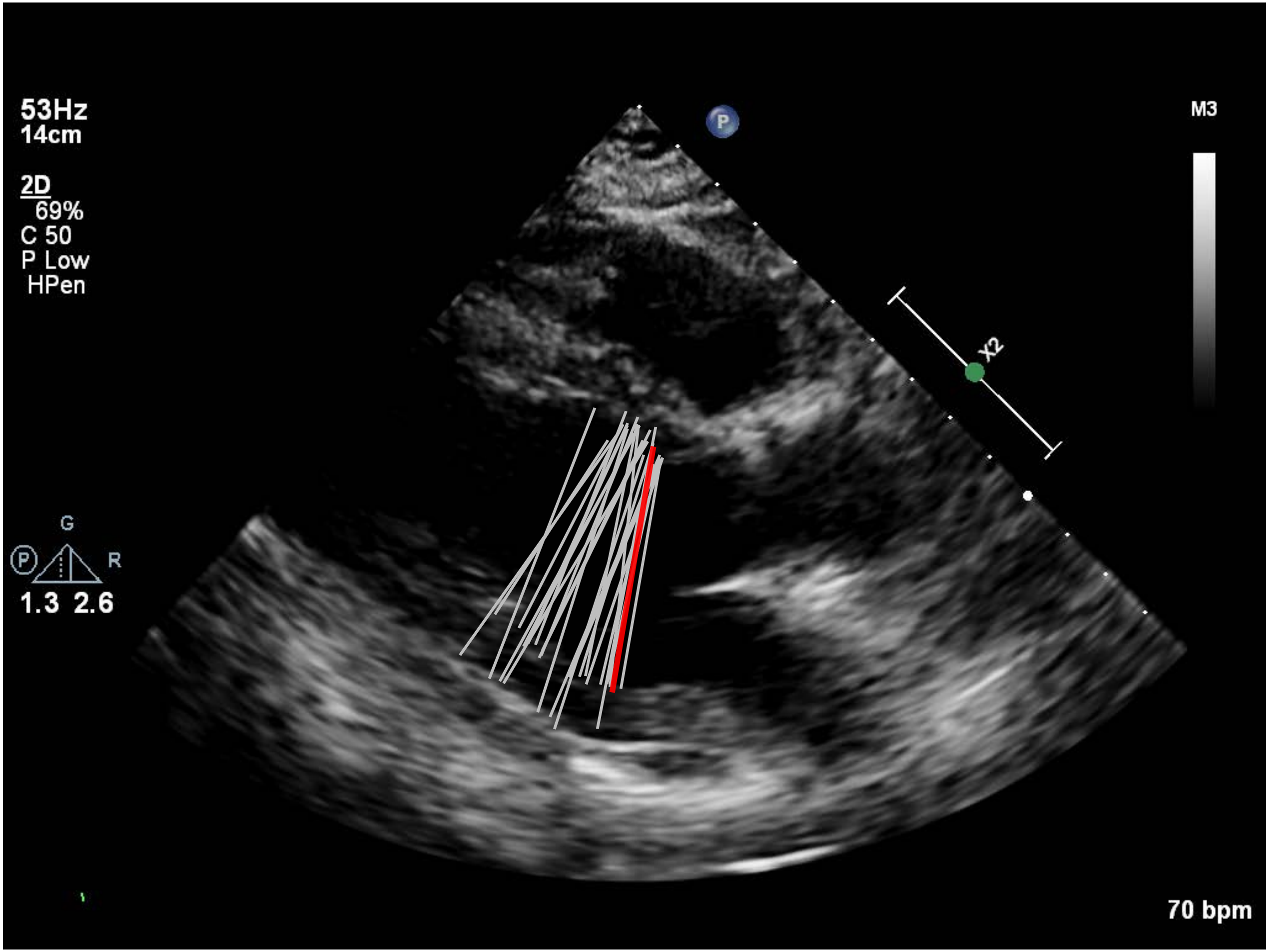

Pt 83 Diastole
Absolute difference between AI and expert consensus: 0.82 mm

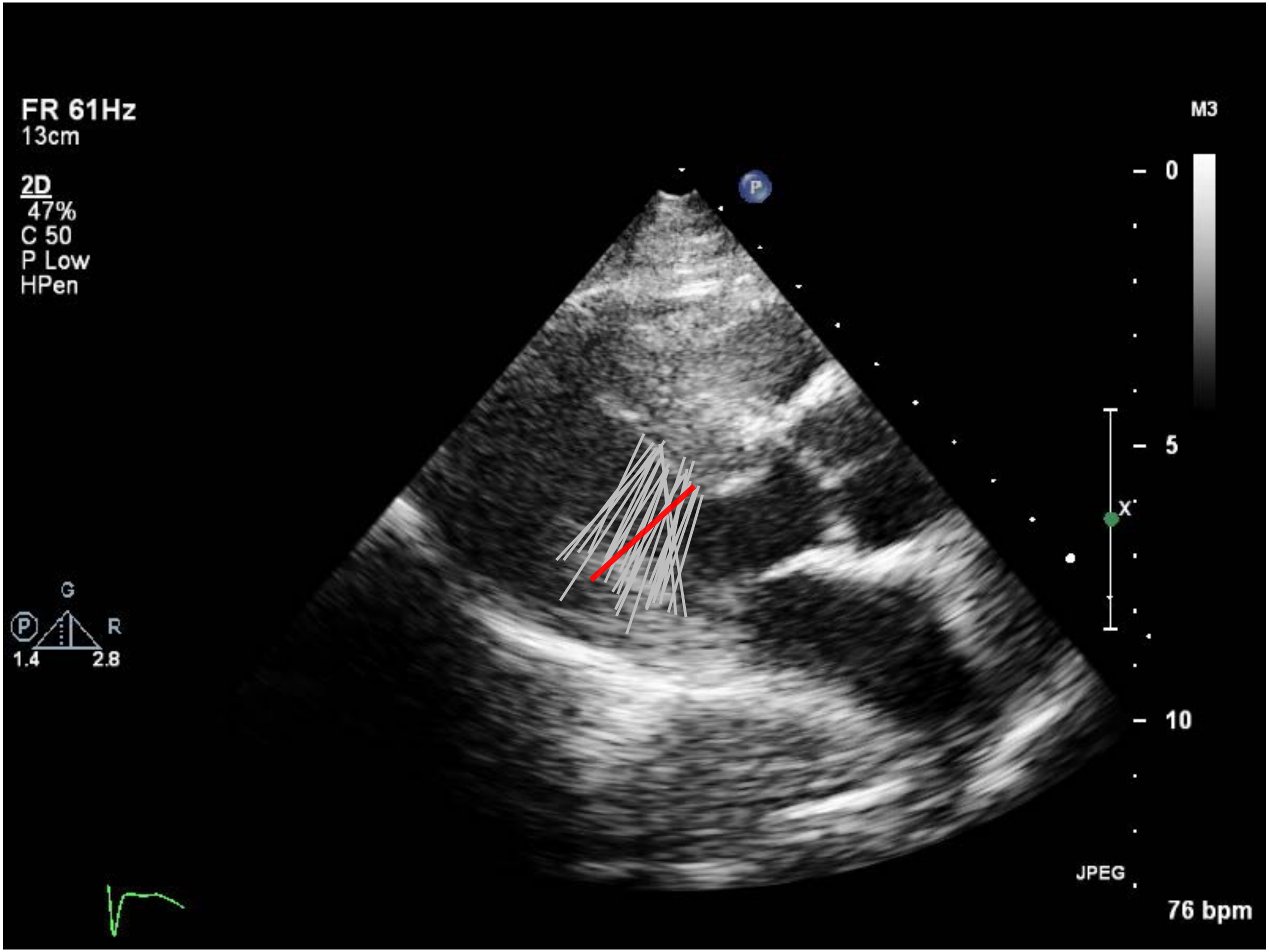

Pt 41 Systole

Absolute difference between AI and expert consensus: 0.83 mm

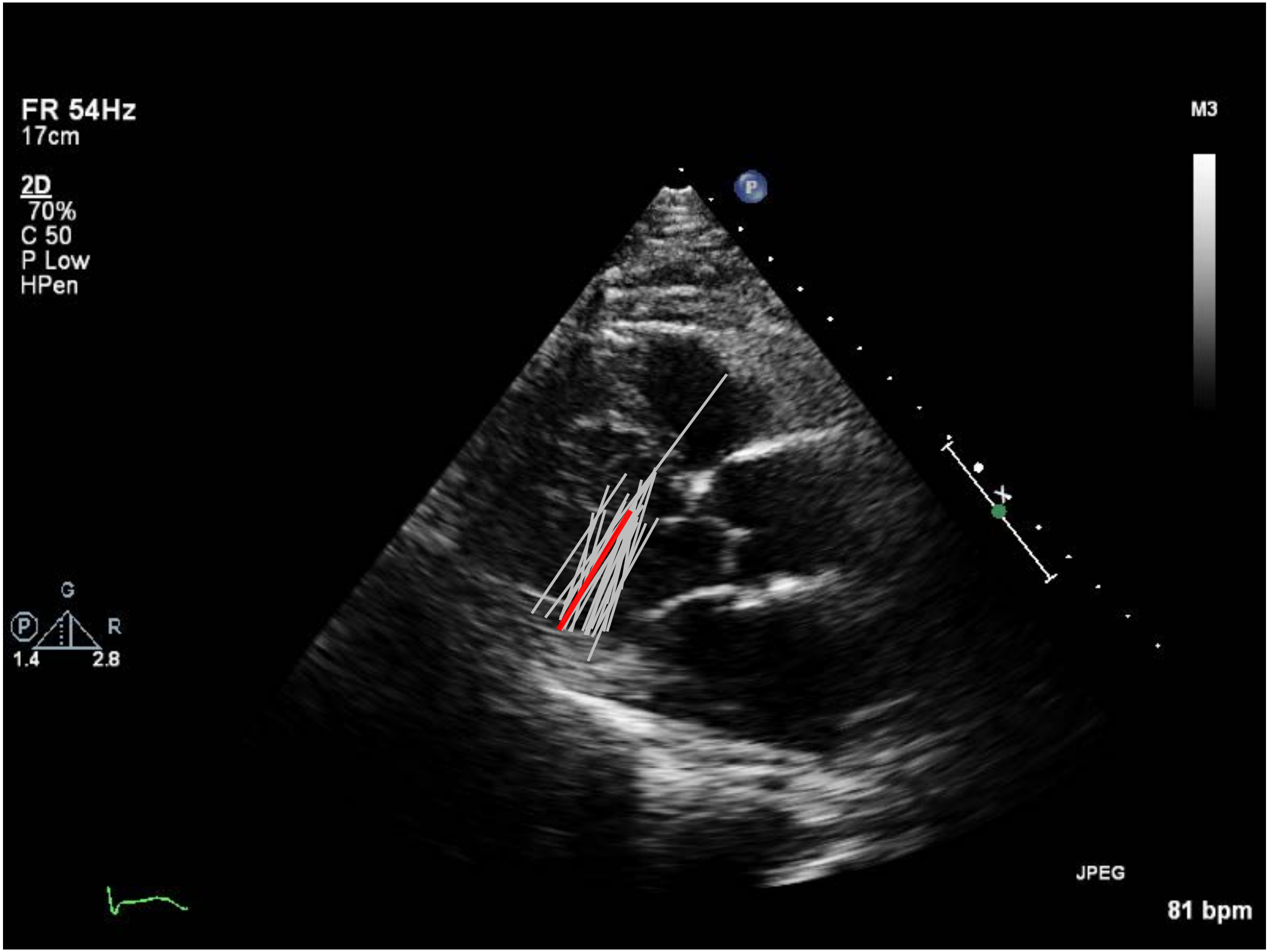

Pt 90 Systole

Absolute difference between AI and expert consensus: 0.83 mm

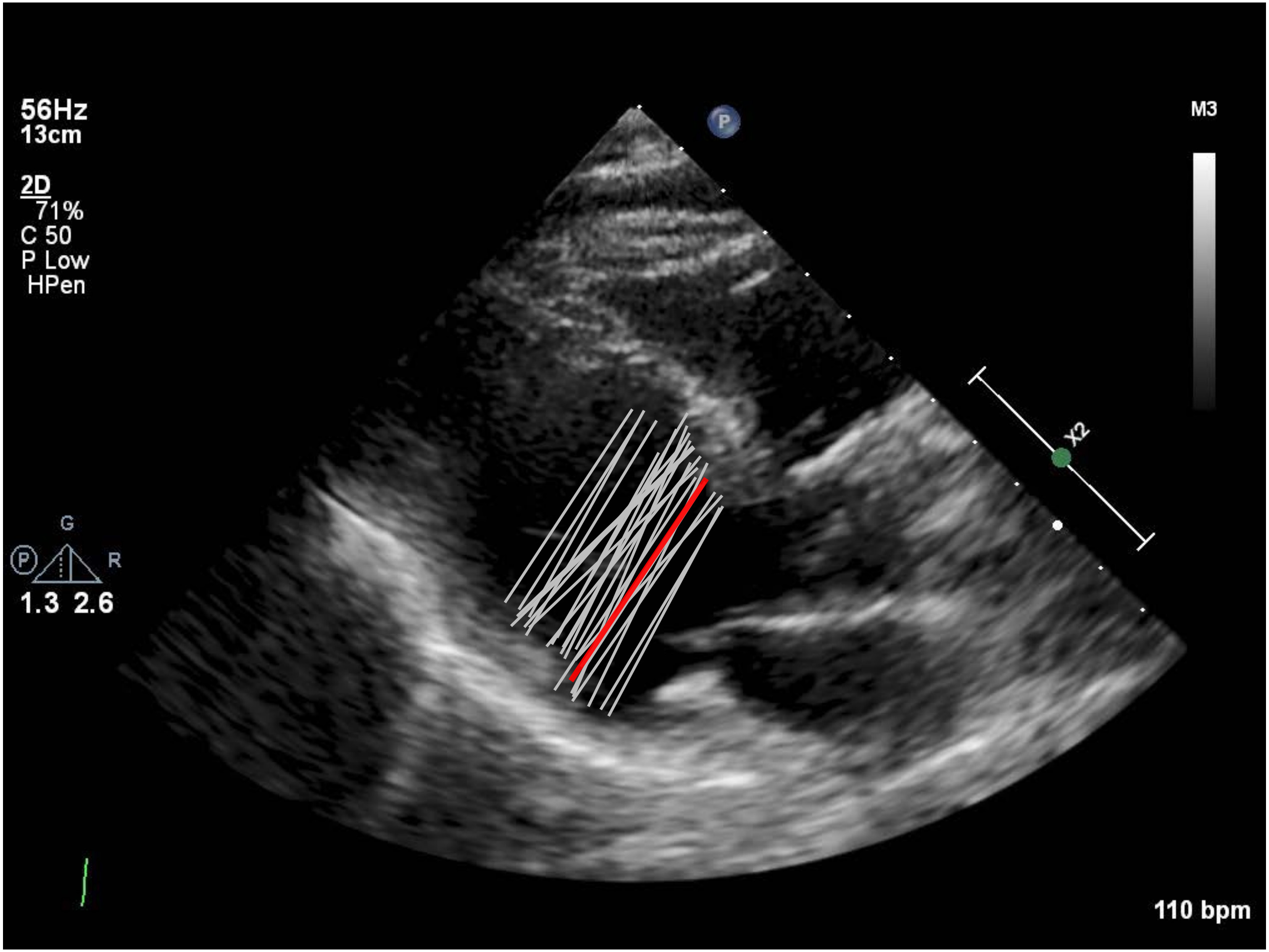

Pt 26 Diastole

Absolute difference between AI and expert consensus: 0.84 mm

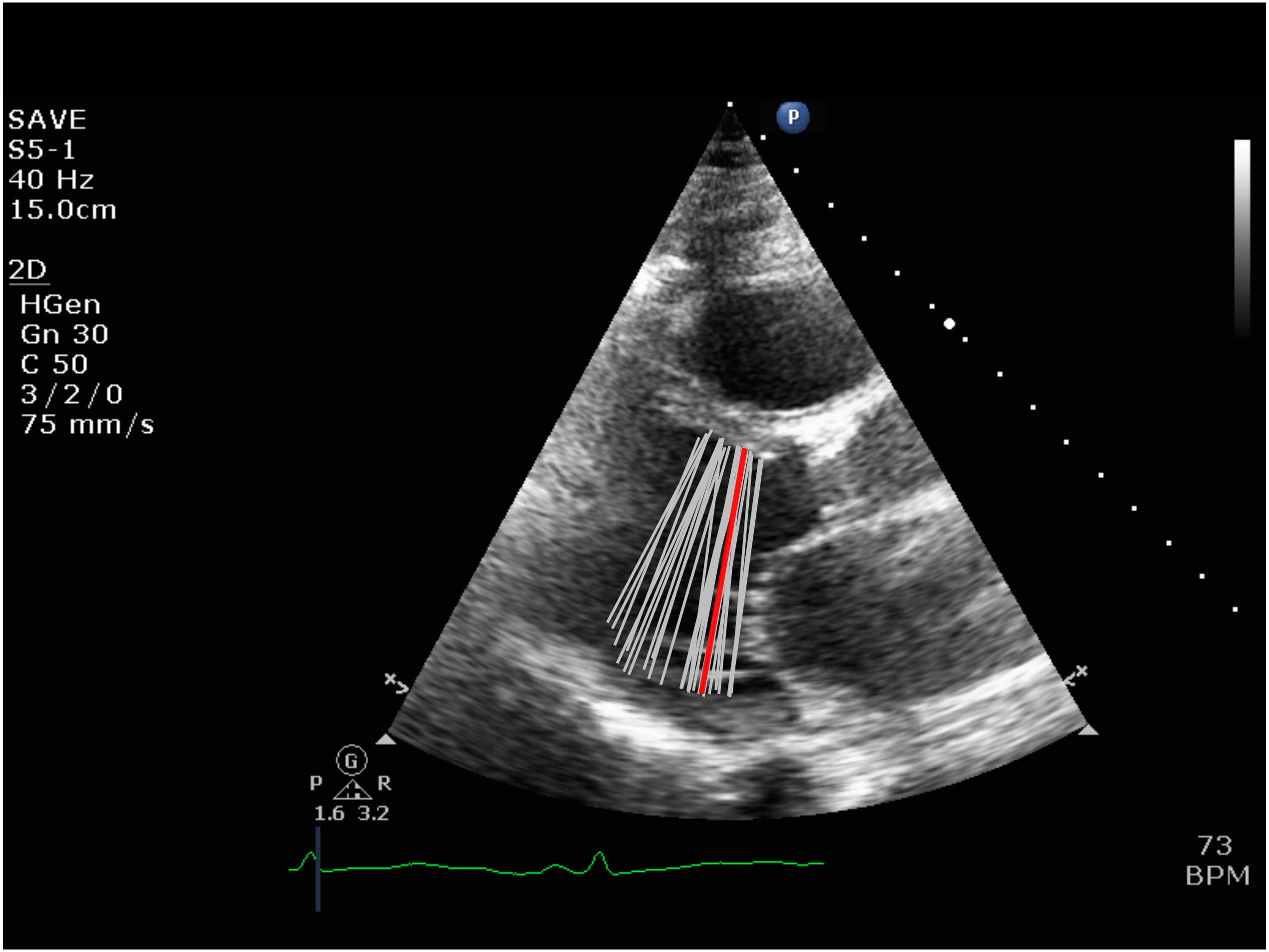

Pt 14 Diastole

Absolute difference between AI and expert consensus: 0.84 mm

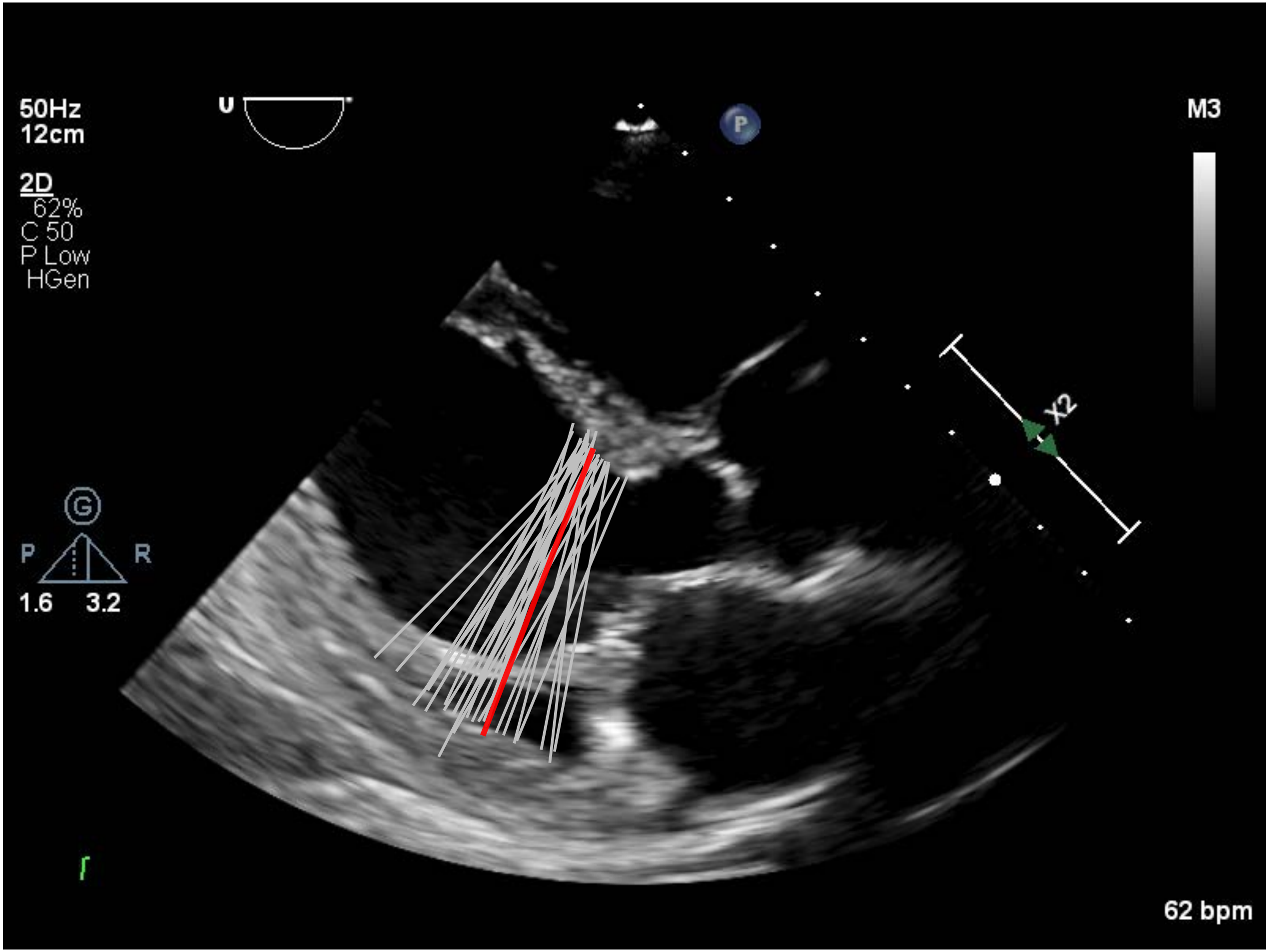

Pt 9 Diastole

Absolute difference between AI and expert consensus: 0.88 mm

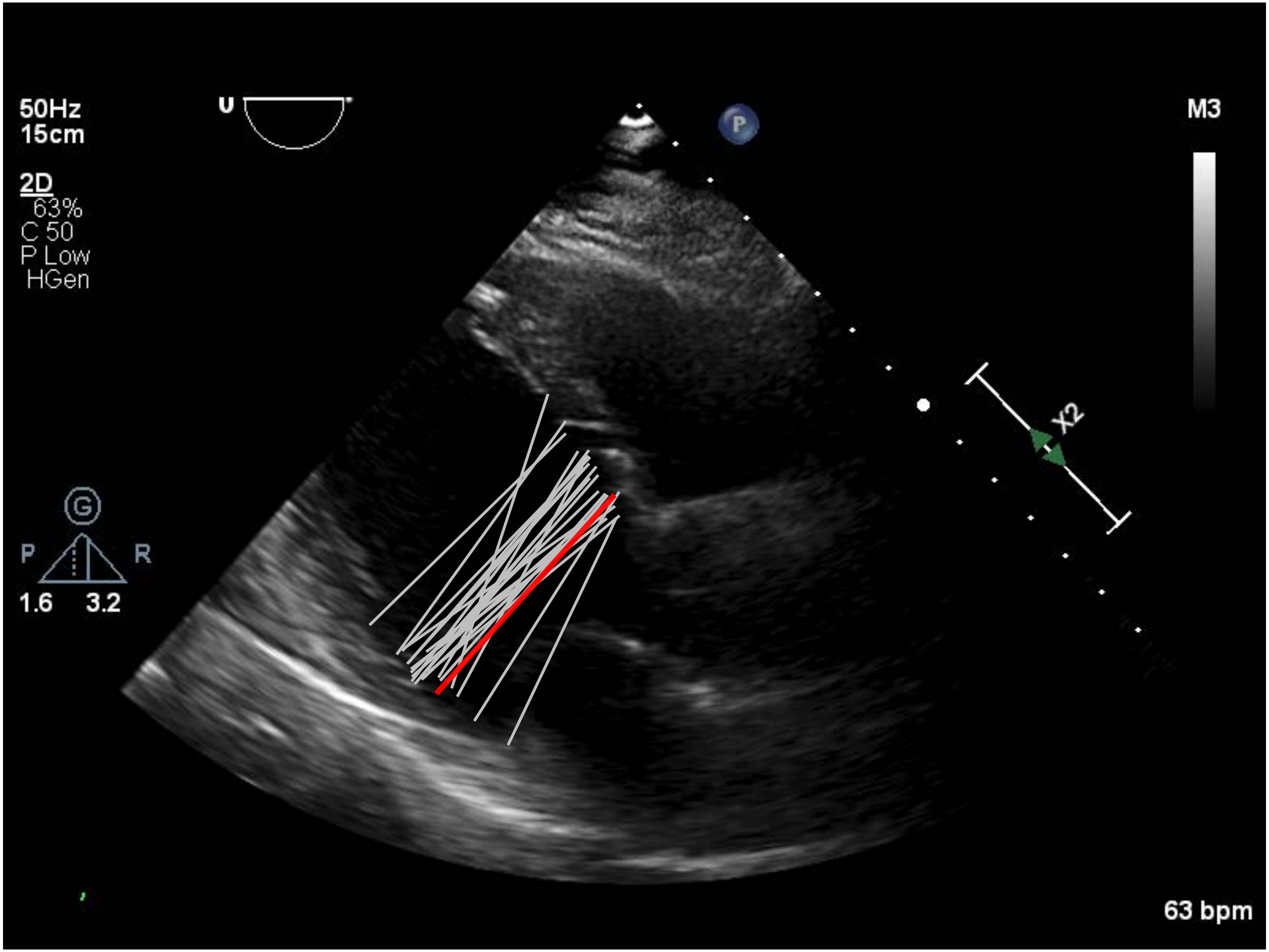

Pt 95 Diastole

Absolute difference between AI and expert consensus: 0.89 mm

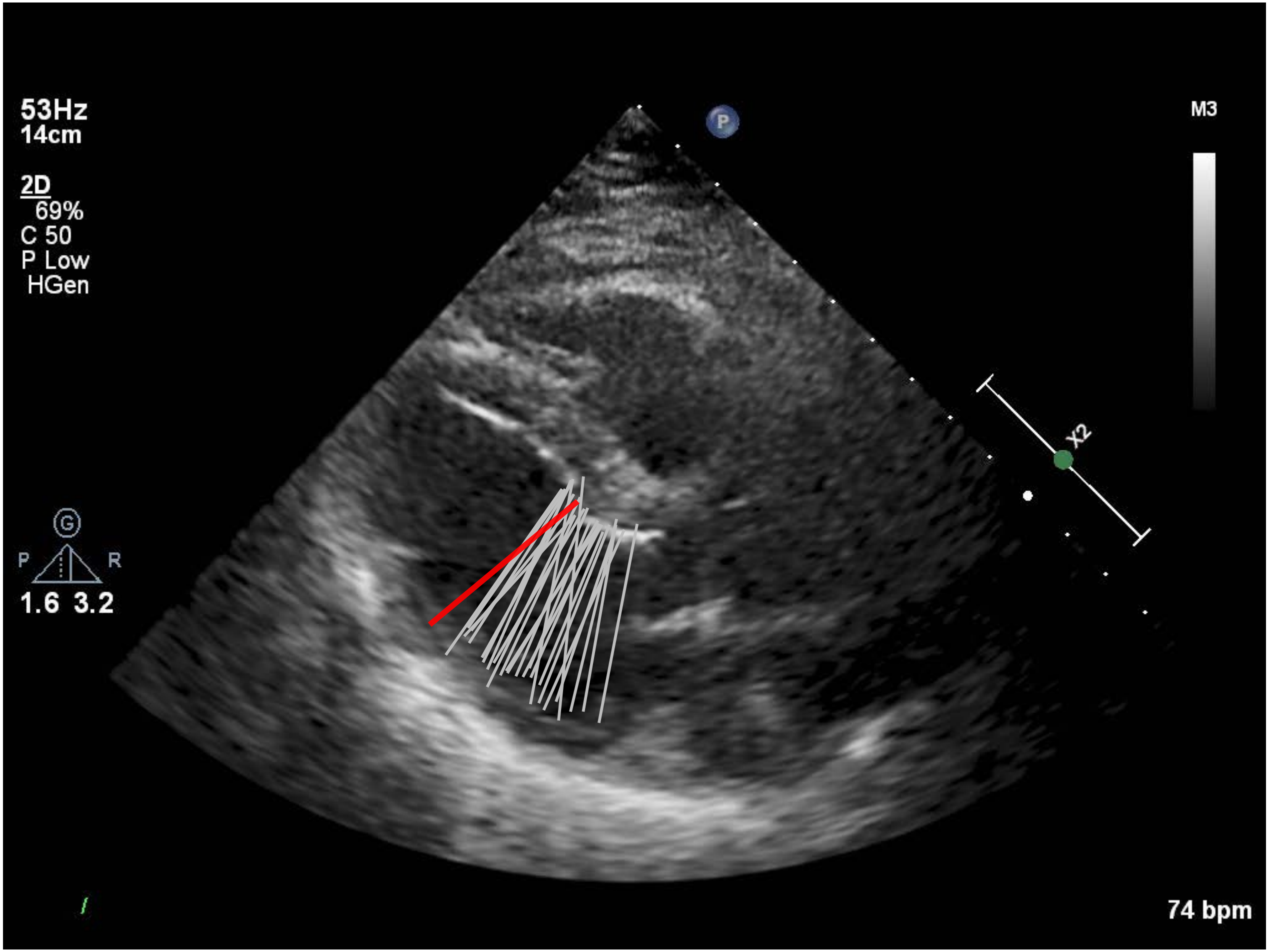

Pt 42 Diastole
Absolute difference between AI and expert consensus: 0.9 mm

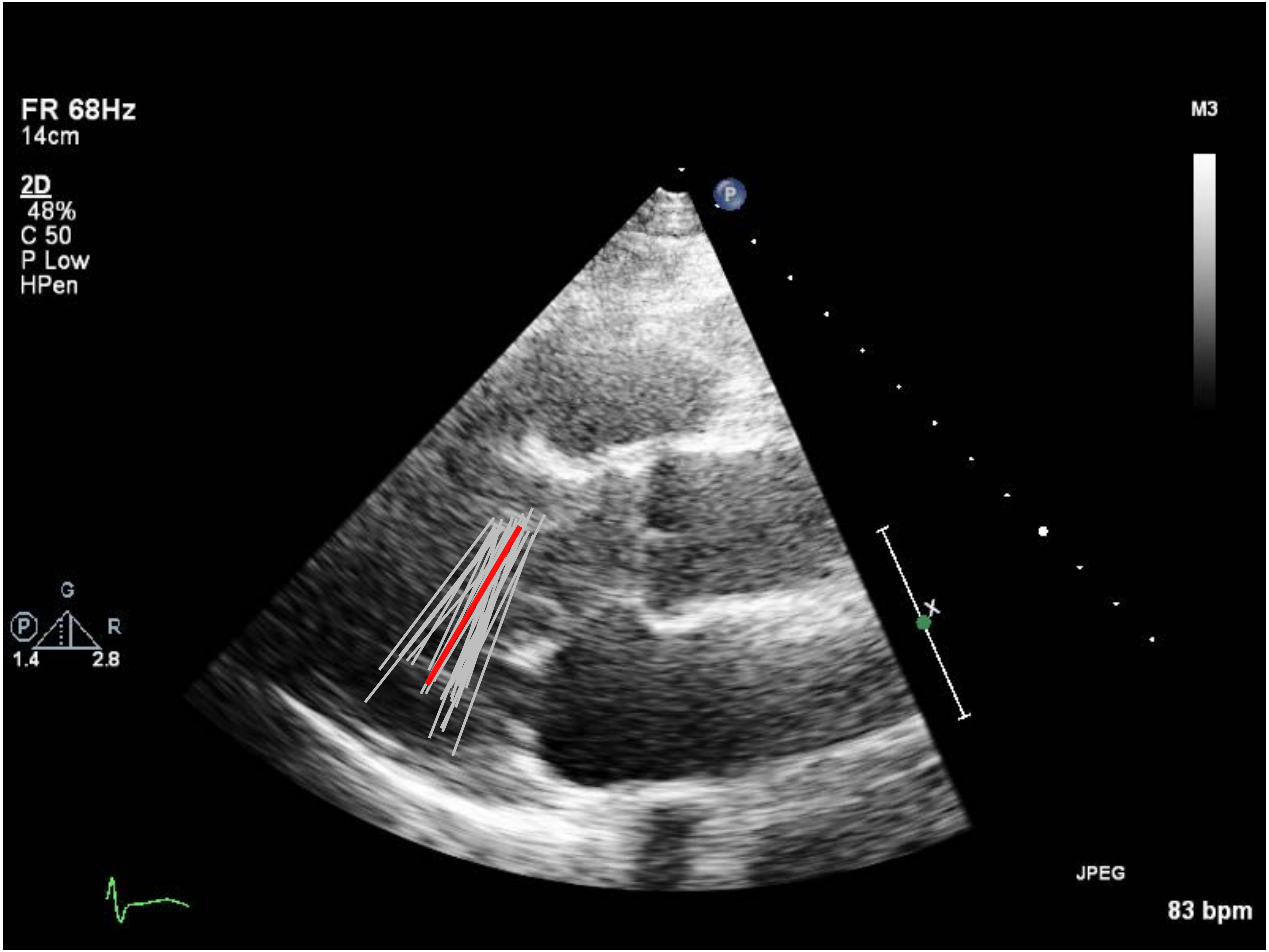

Pt 86 Systole

Absolute difference between AI and expert consensus: 0.92 mm

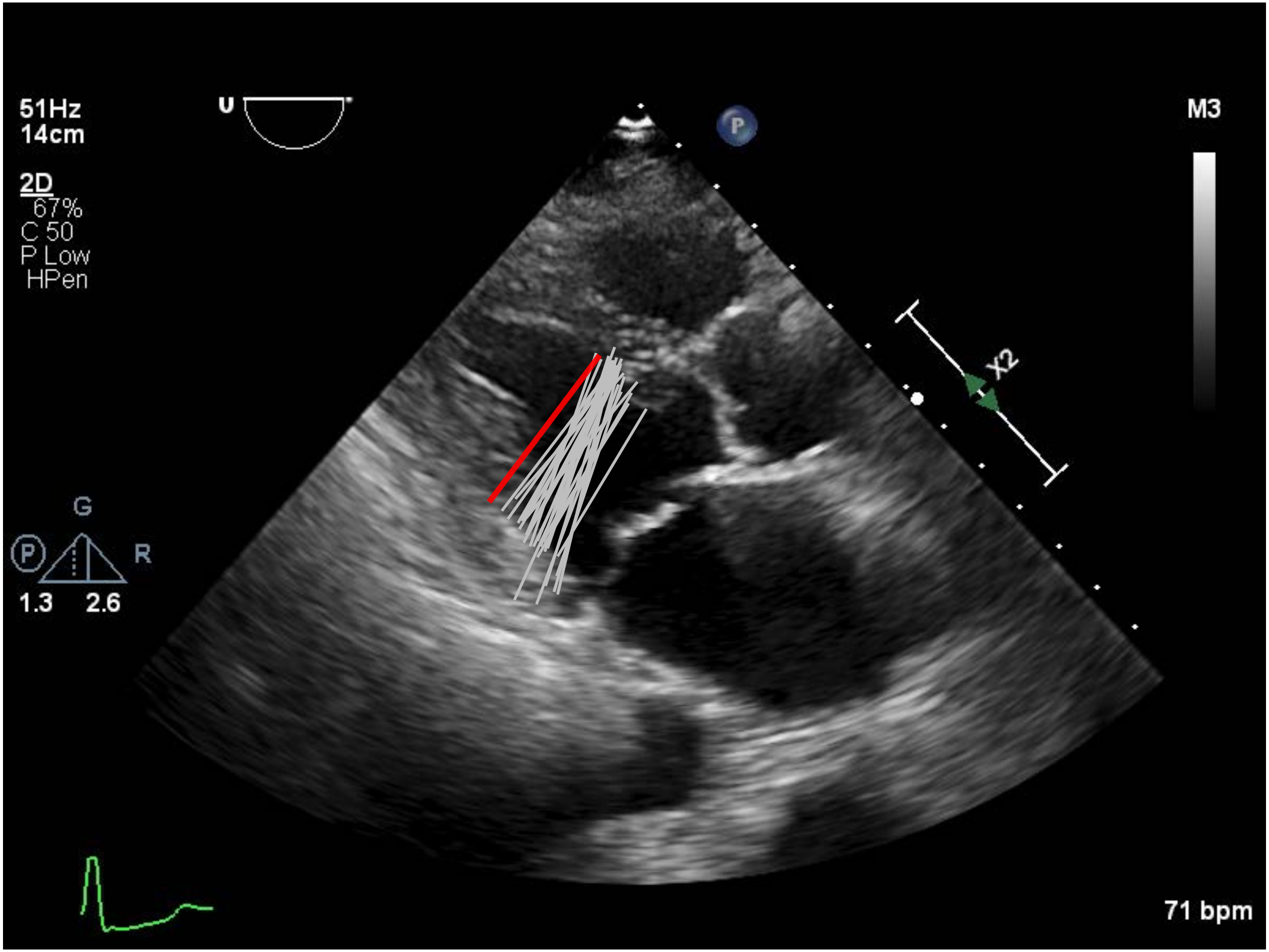

Pt 88 Systole

Absolute difference between AI and expert consensus: 0.94 mm

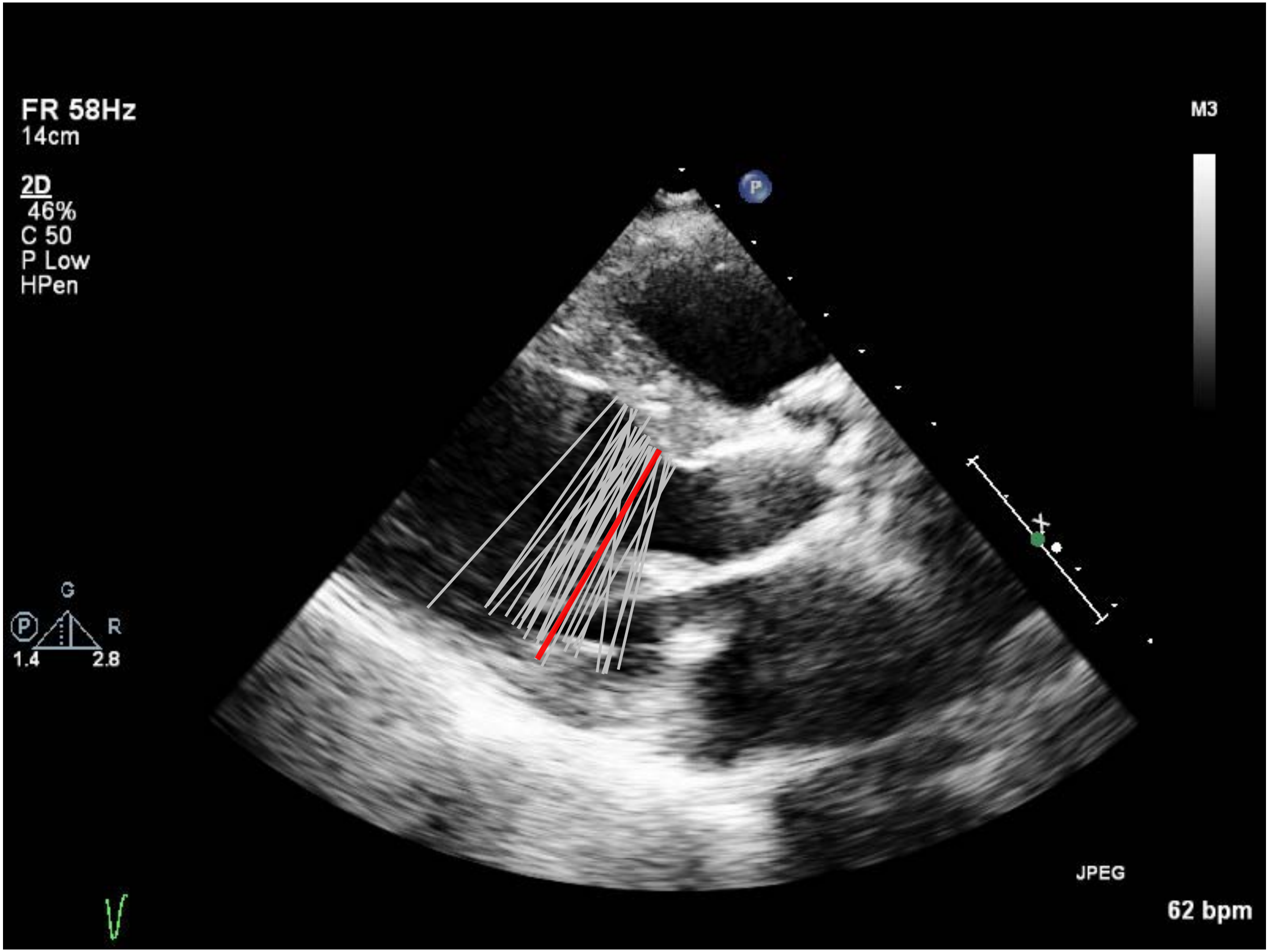

Pt 92 Diastole

Absolute difference between AI and expert consensus: 0.96 mm

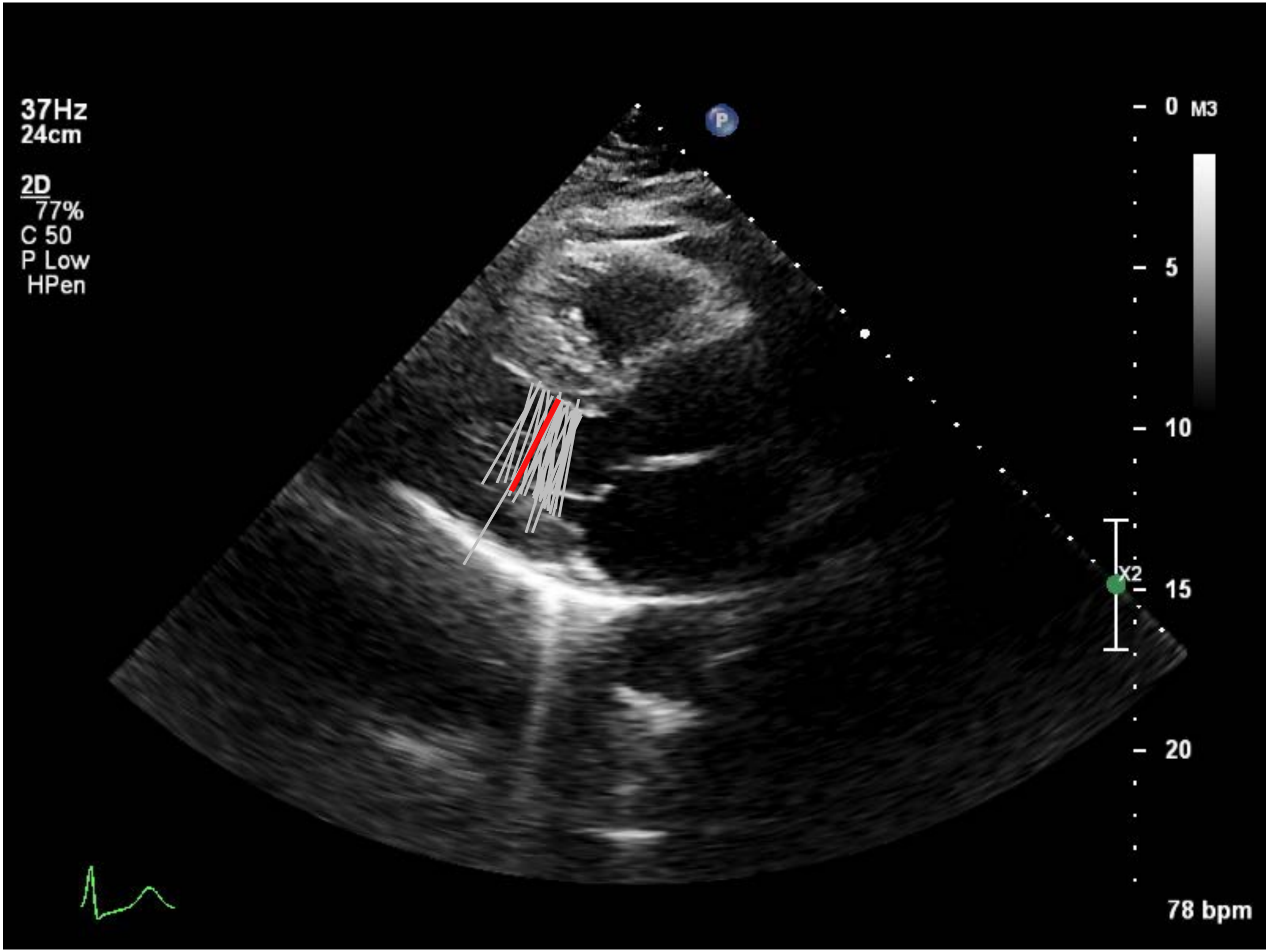

Pt 23 Systole

Absolute difference between AI and expert consensus: 0.96 mm

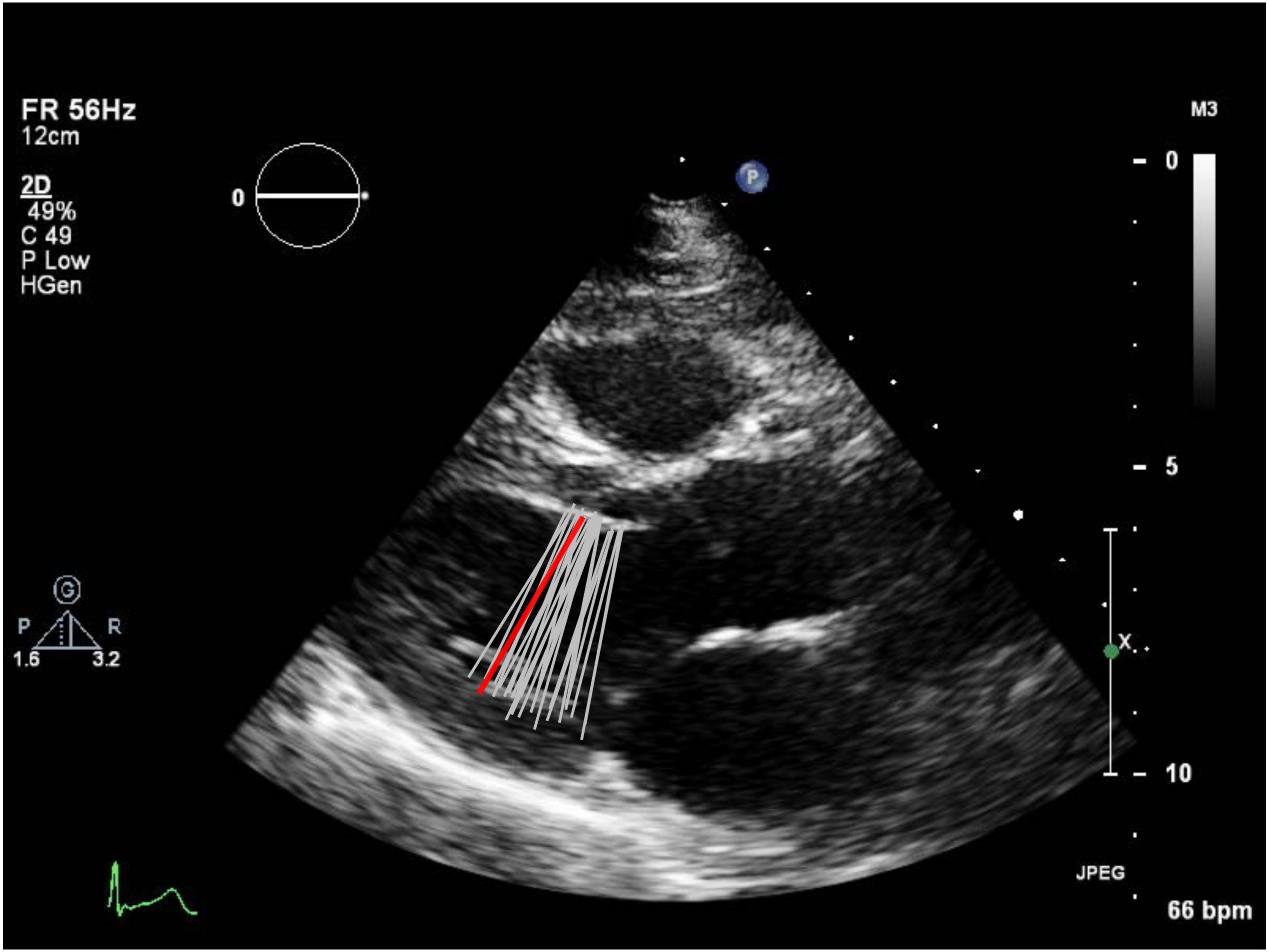

Pt 40 Systole

Absolute difference between AI and expert consensus: 0.98 mm

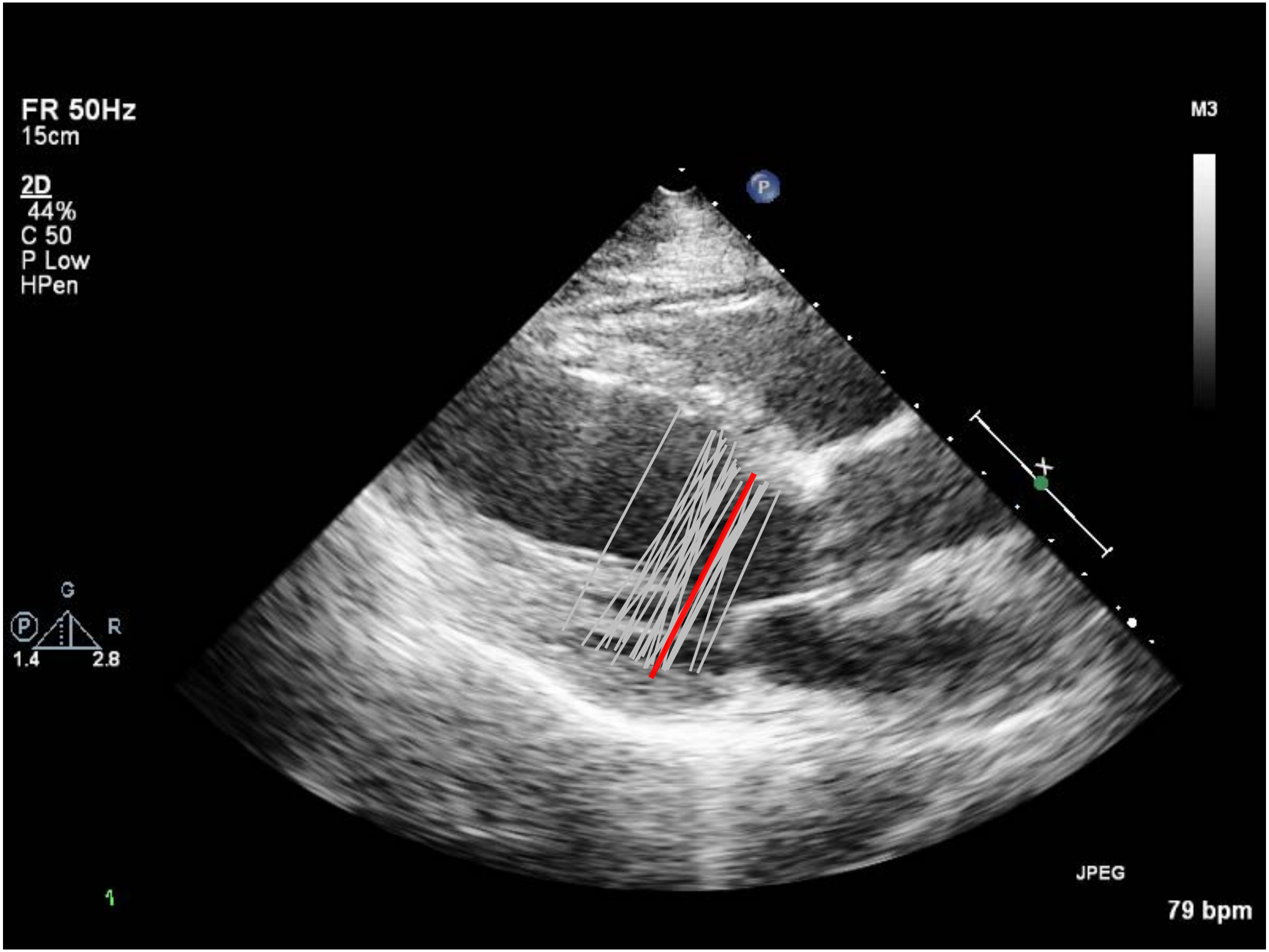

Pt 12 Diastole

Absolute difference between AI and expert consensus: 1 mm

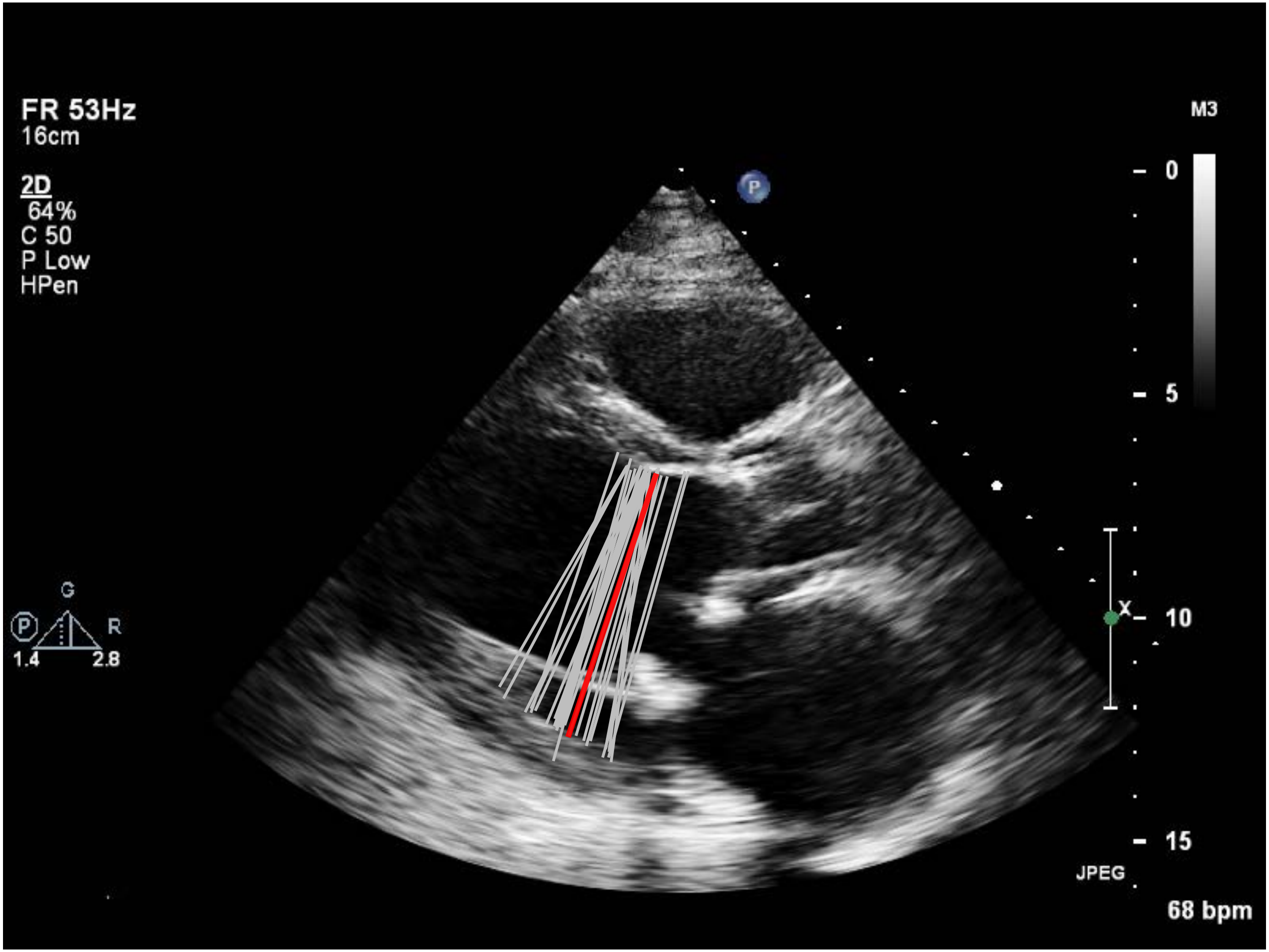

Pt 56 Diastole

Absolute difference between AI and expert consensus: 1 mm

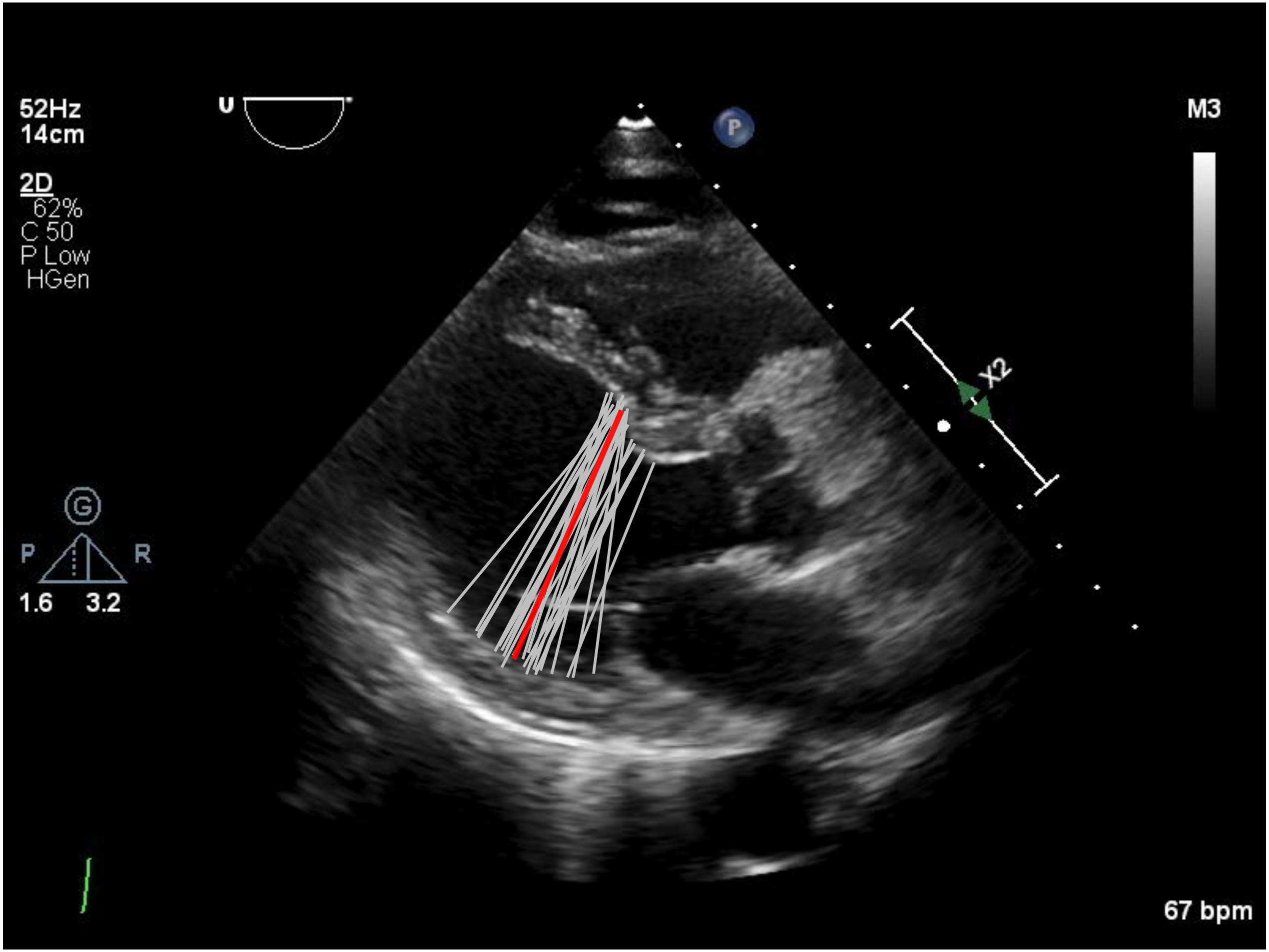

Pt 80 Diastole

Absolute difference between AI and expert consensus: 1 mm

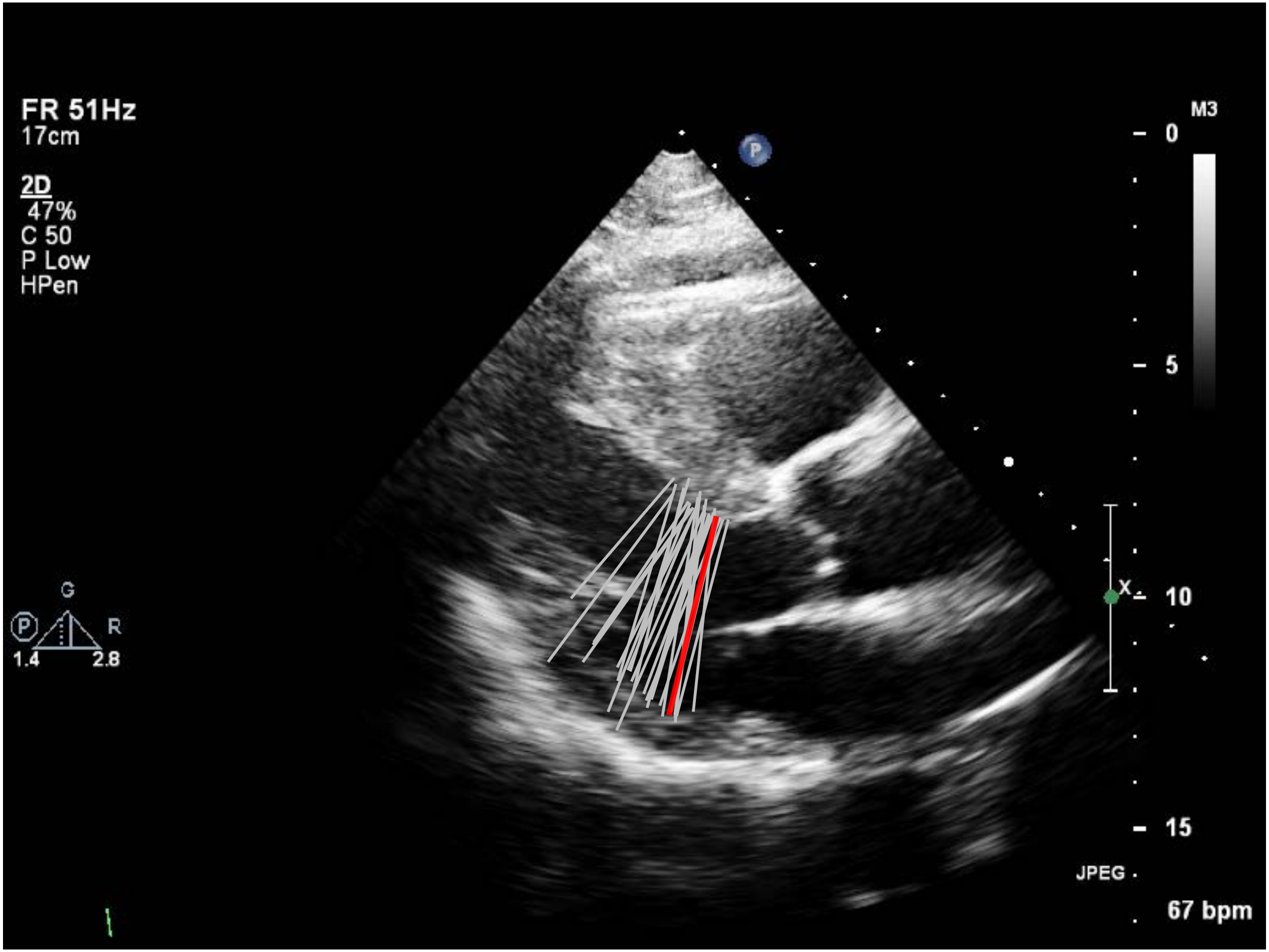

Pt 5 Diastole

Absolute difference between AI and expert consensus: 1.1 mm

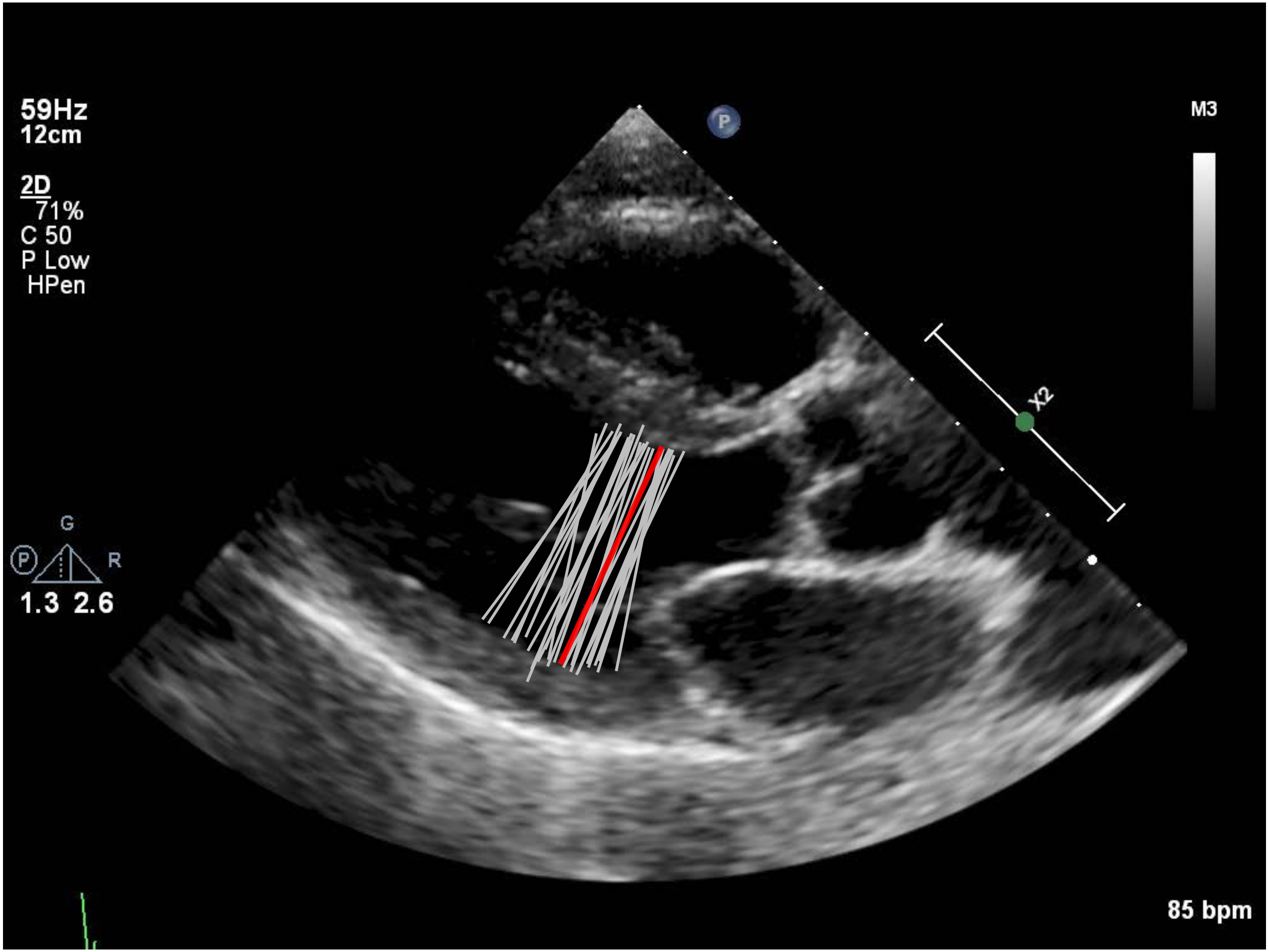

Pt 75 Diastole
Absolute difference between AI and expert consensus: 1.1 mm

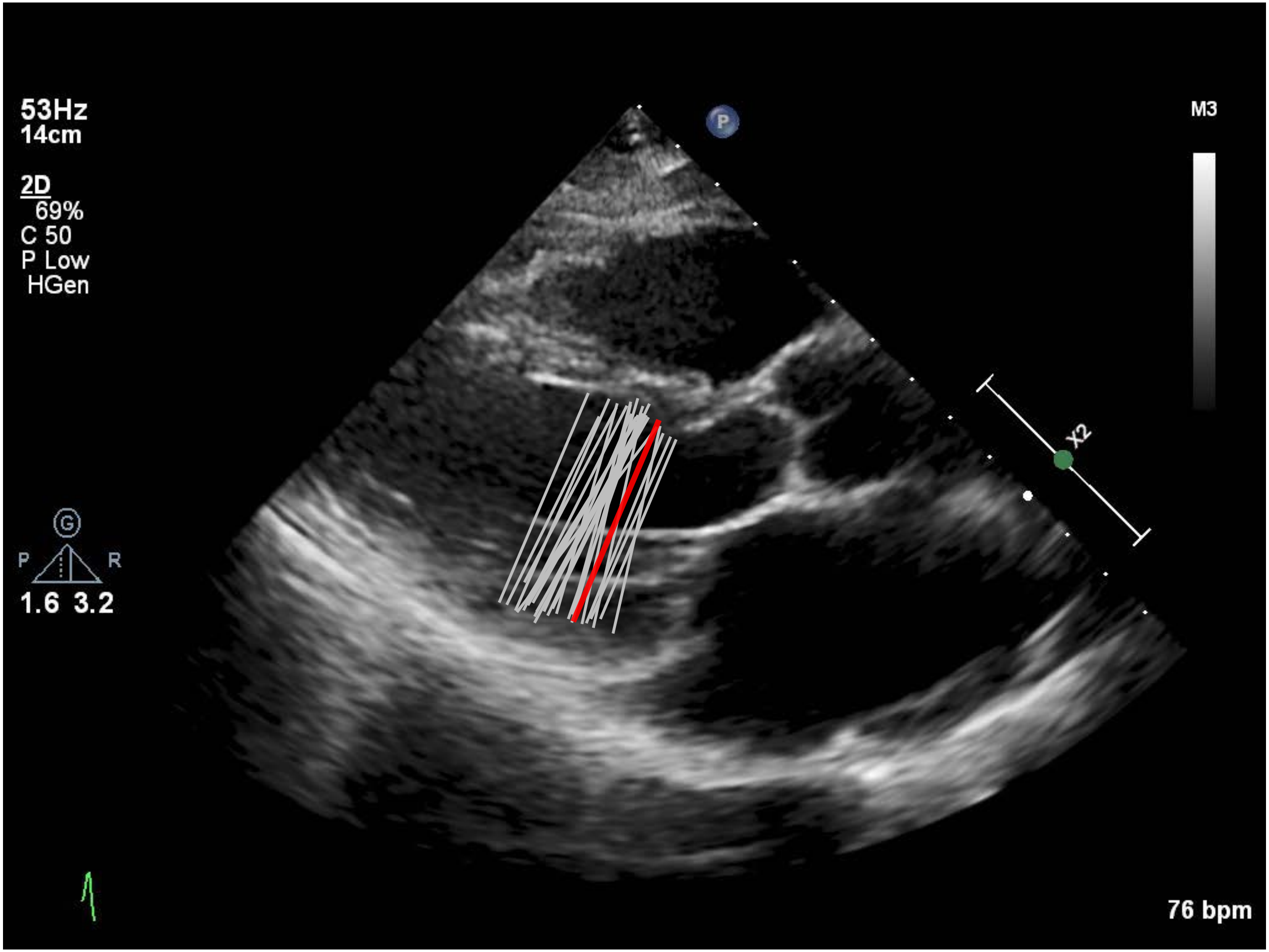

Pt 87 Diastole

Absolute difference between AI and expert consensus: 1.1 mm

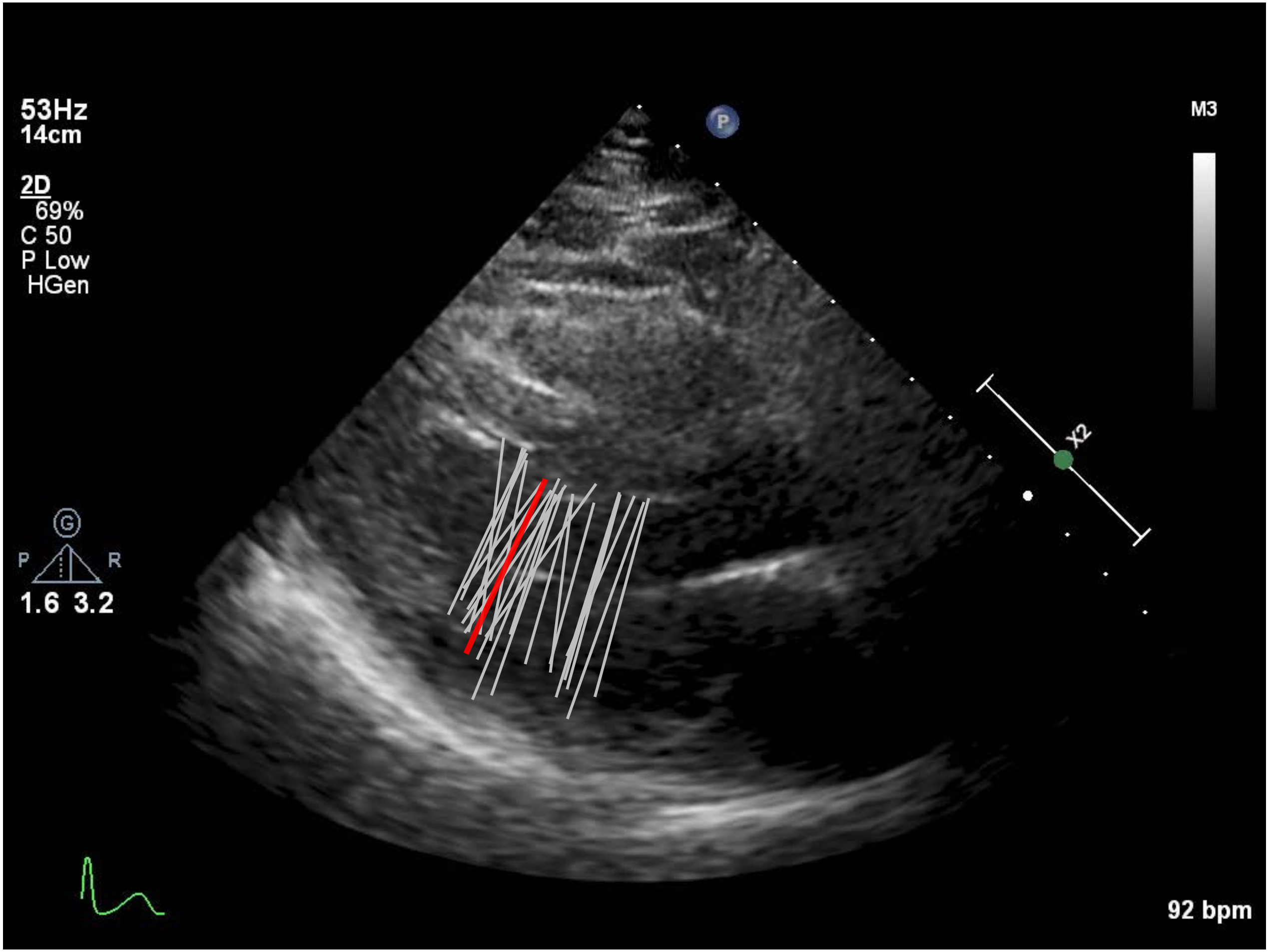

Pt 70 Systole
Absolute difference between AI and expert consensus: 1.1 mm

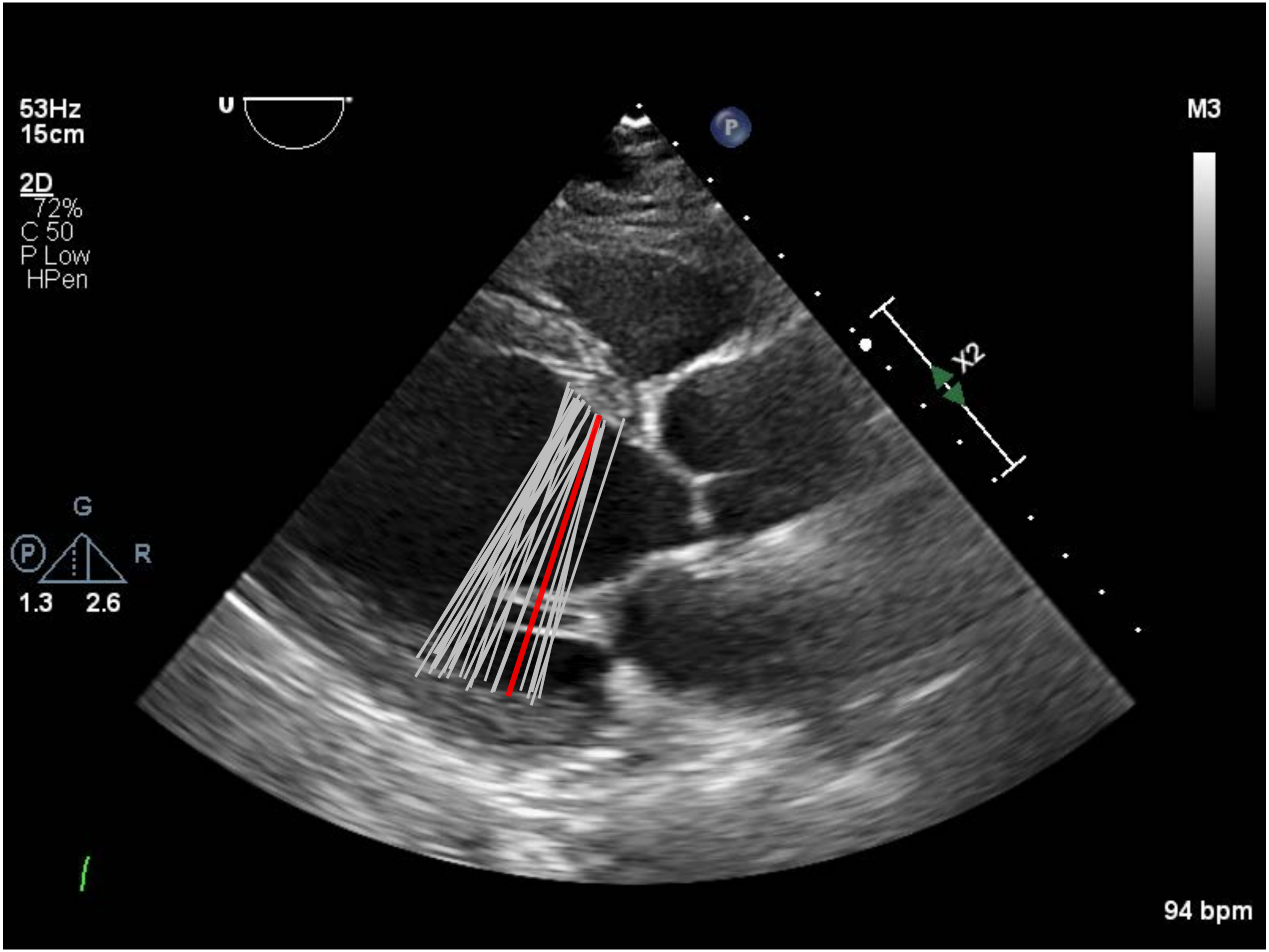

Pt 61 Diastole

Absolute difference between AI and expert consensus: 1.1 mm

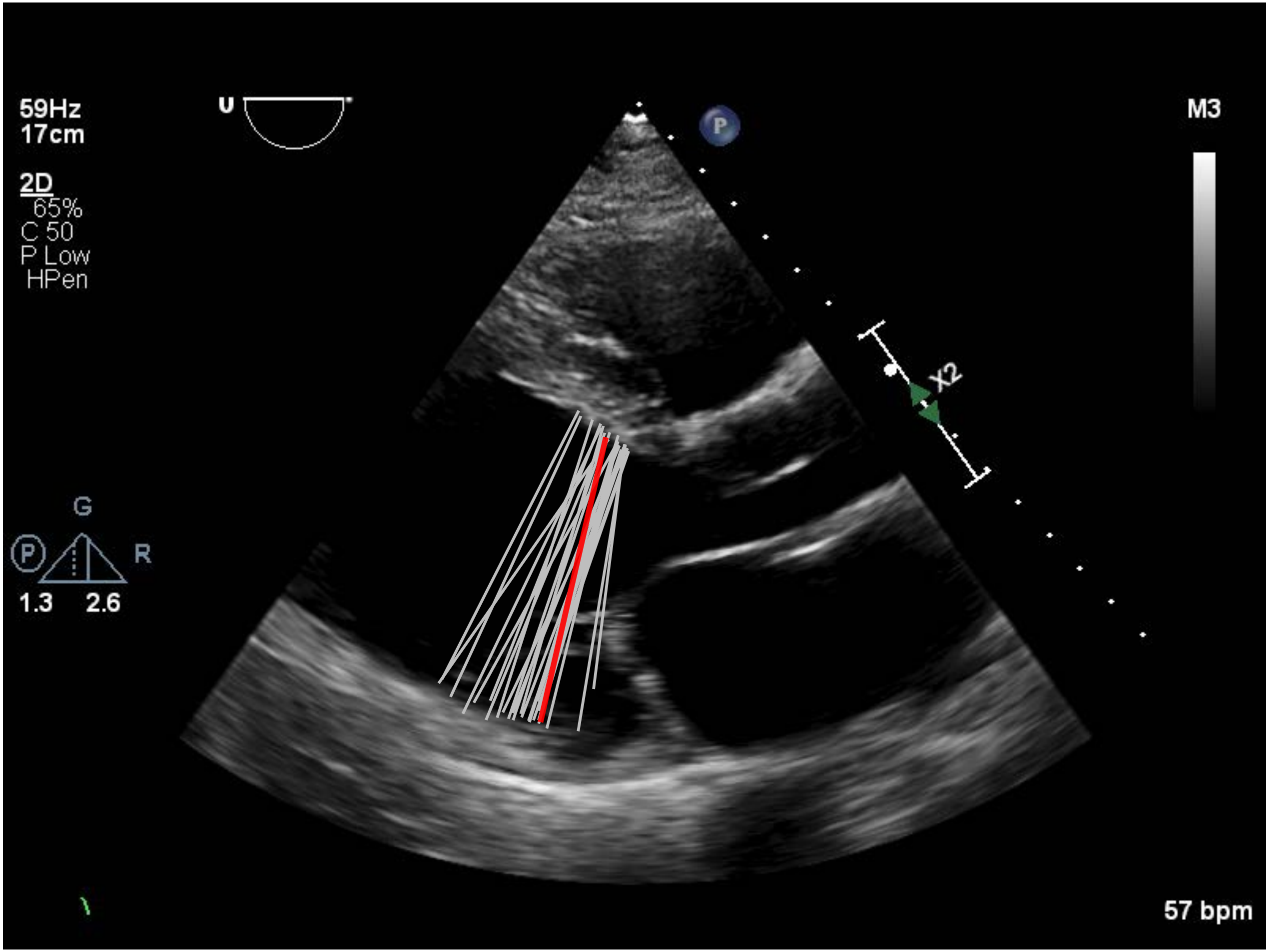

Pt 79 Diastole

Absolute difference between AI and expert consensus: 1.1 mm

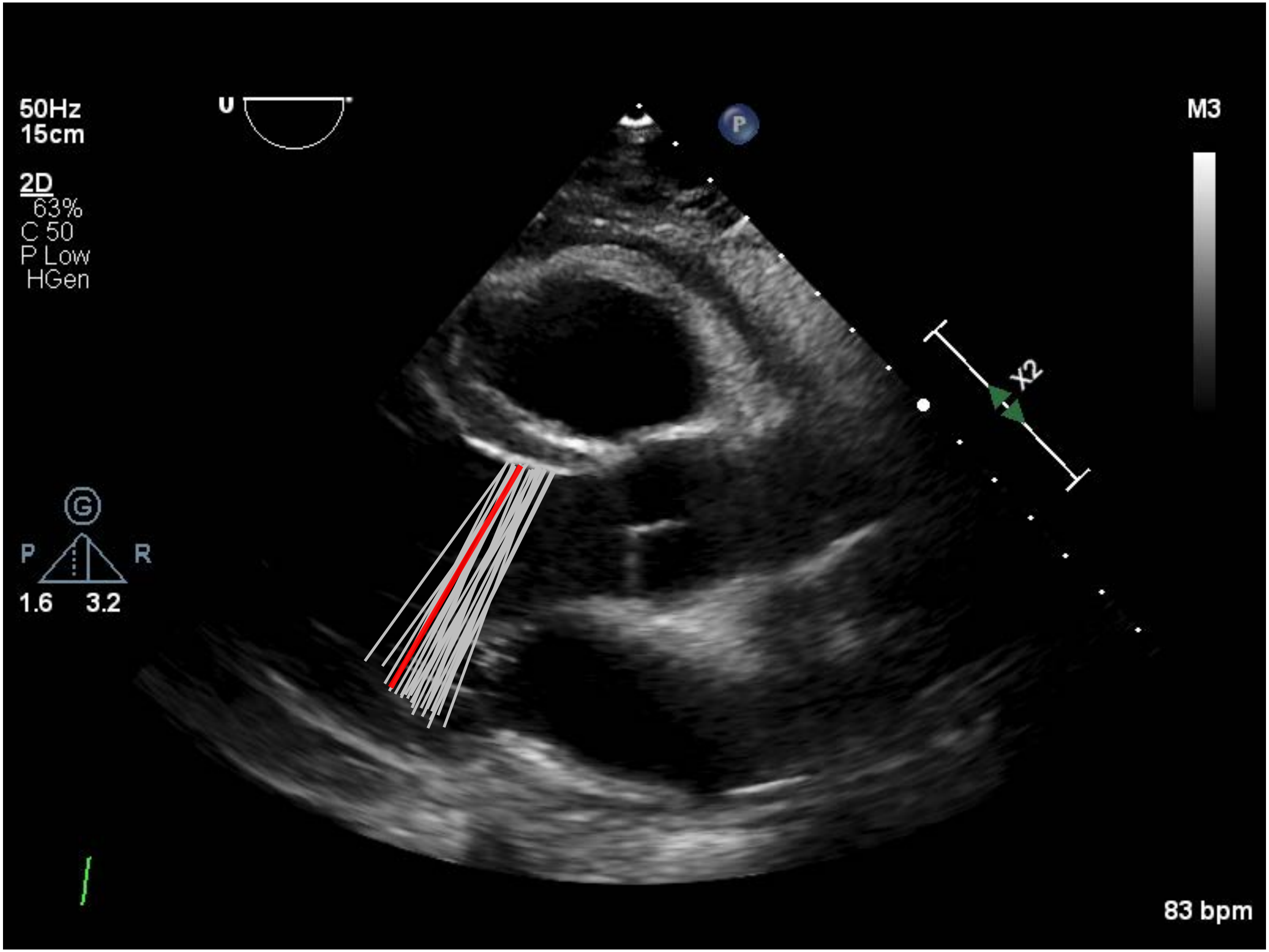

Pt 4 Diastole

Absolute difference between AI and expert consensus: 1.1 mm

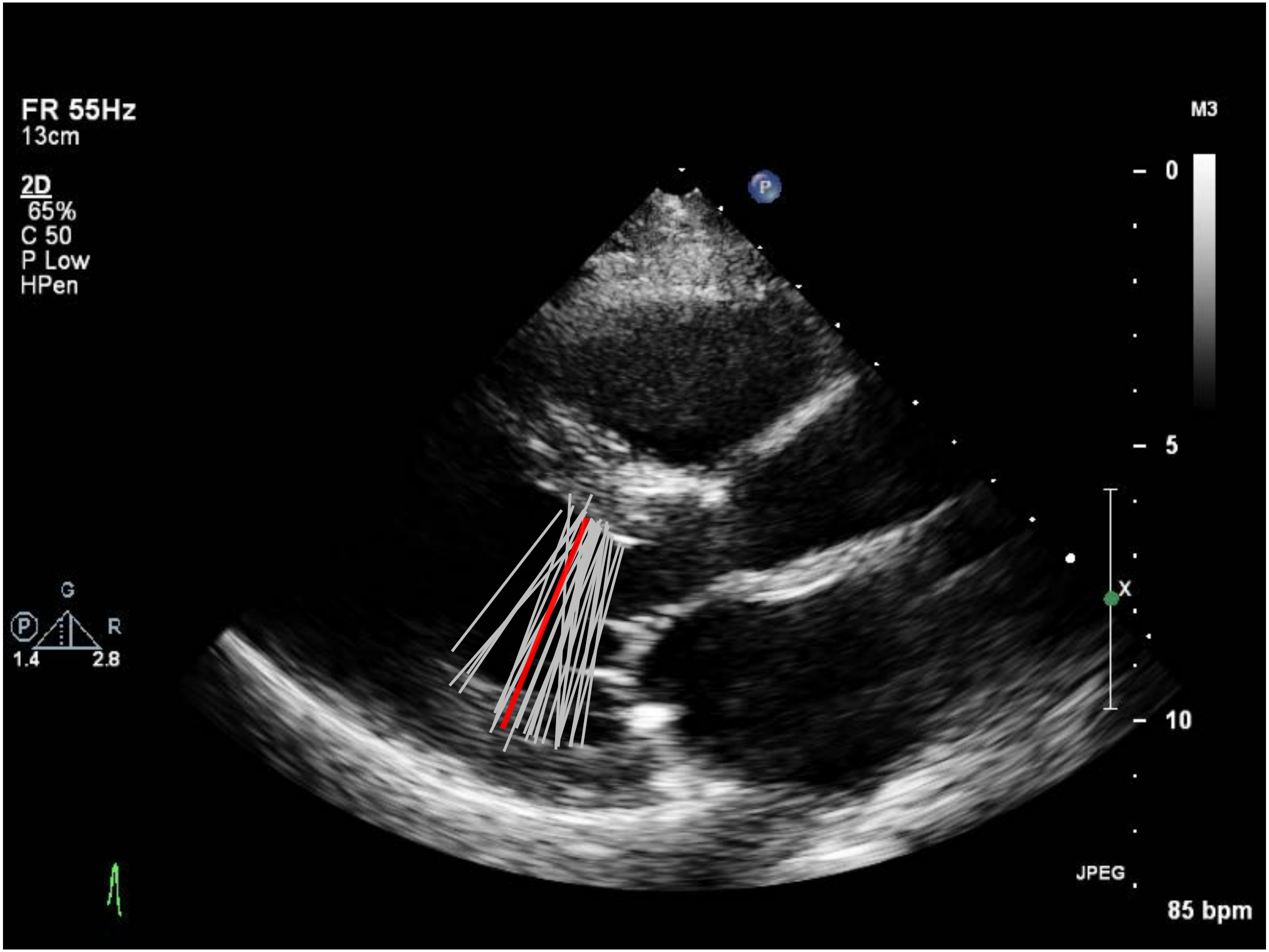

Pt 73 Diastole

Absolute difference between AI and expert consensus: 1.1 mm

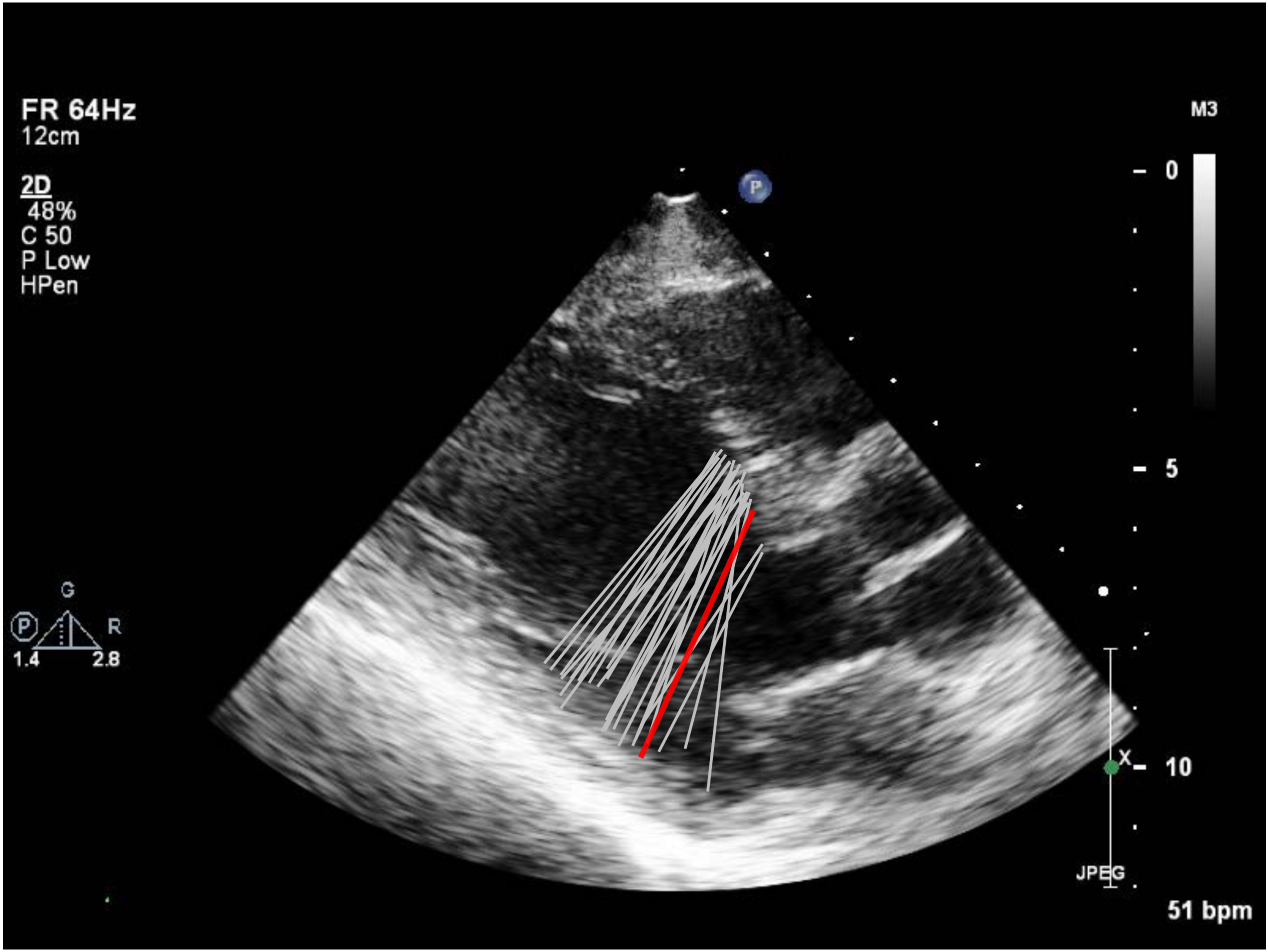

Pt 36 Diastole

Absolute difference between AI and expert consensus: 1.1 mm

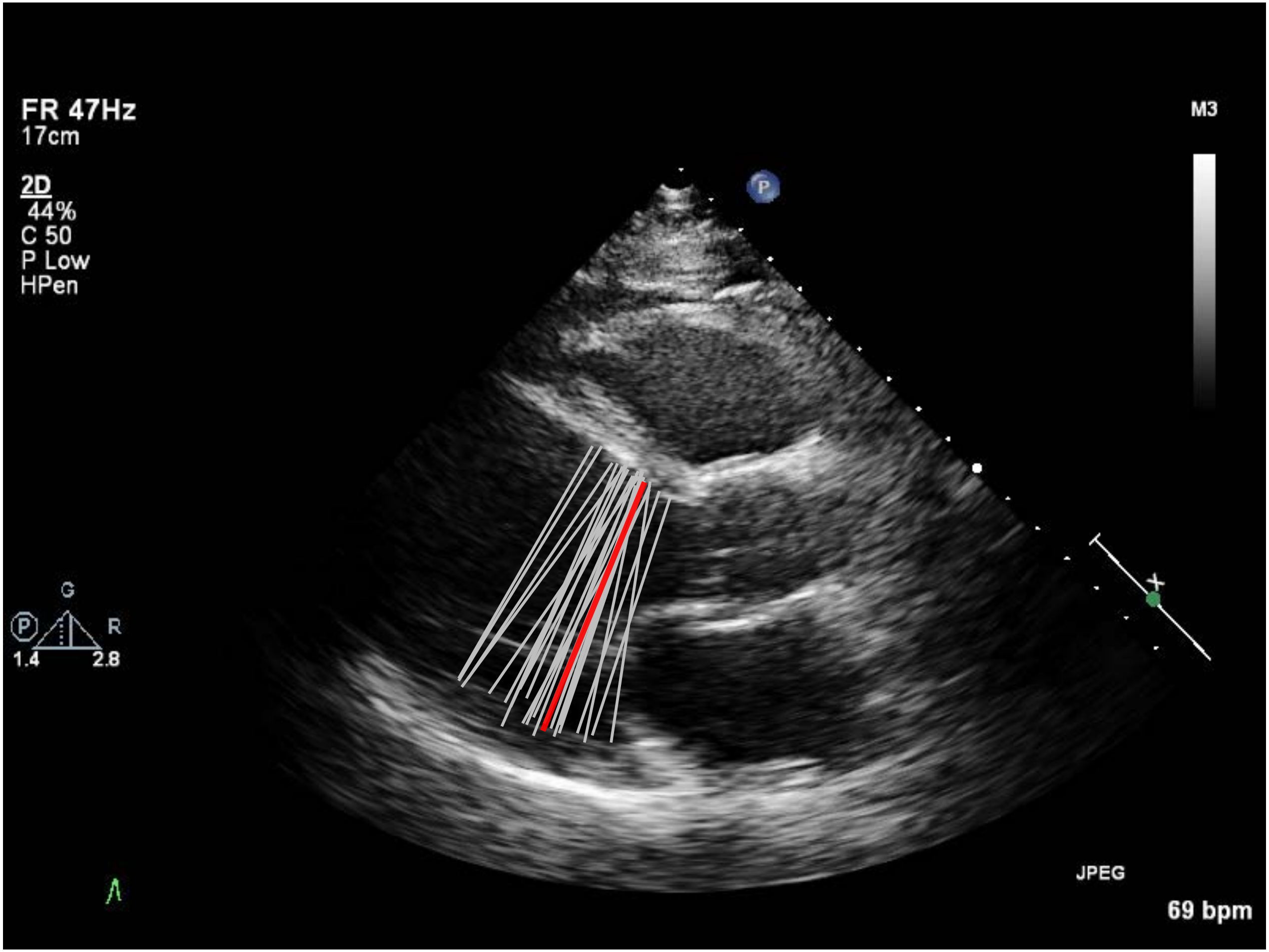

Pt 34 Diastole

Absolute difference between AI and expert consensus: 1.2 mm

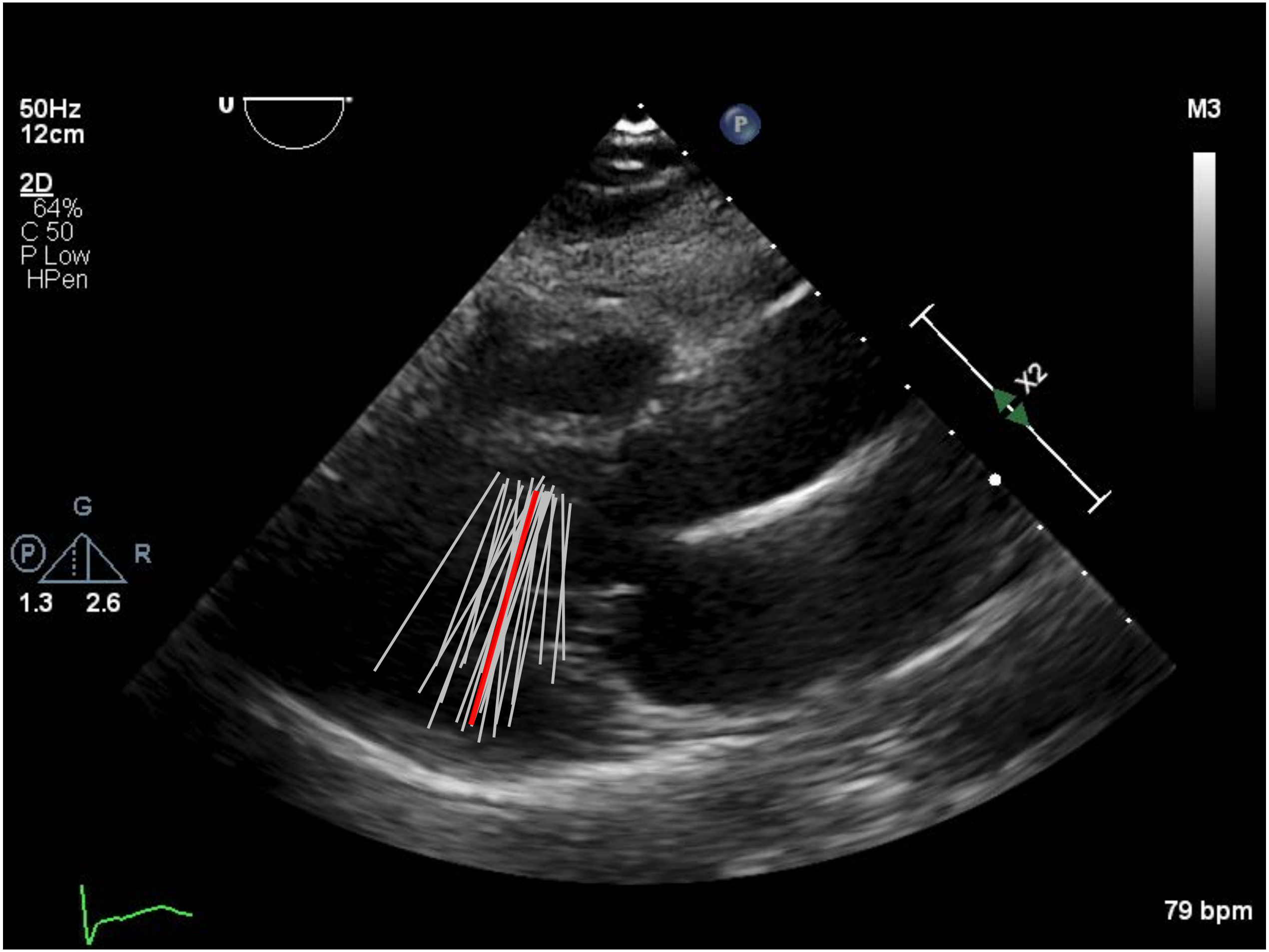

Pt 55 Systole

Absolute difference between AI and expert consensus: 1.2 mm

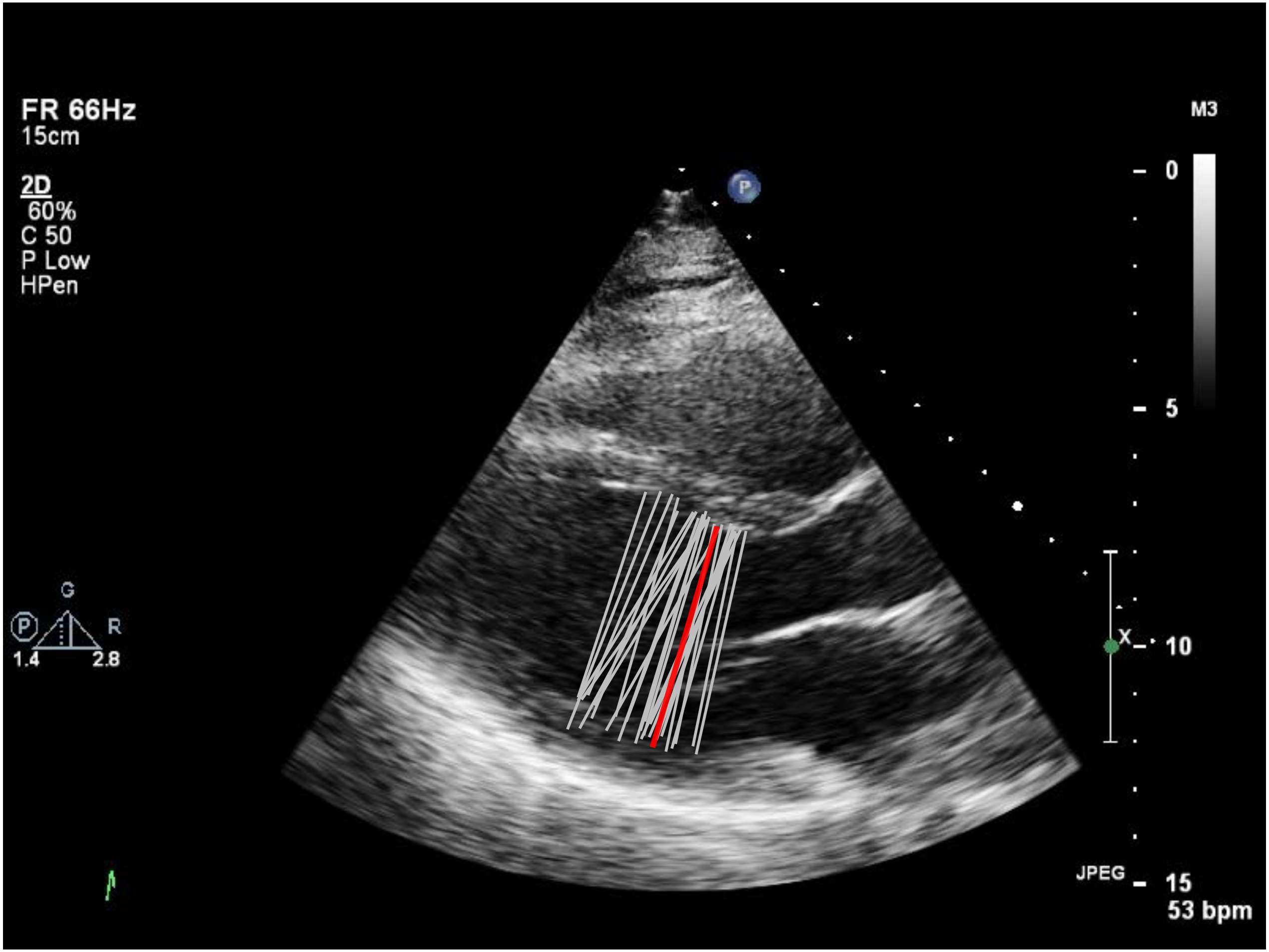

Pt 51 Diastole

Absolute difference between AI and expert consensus: 1.3 mm

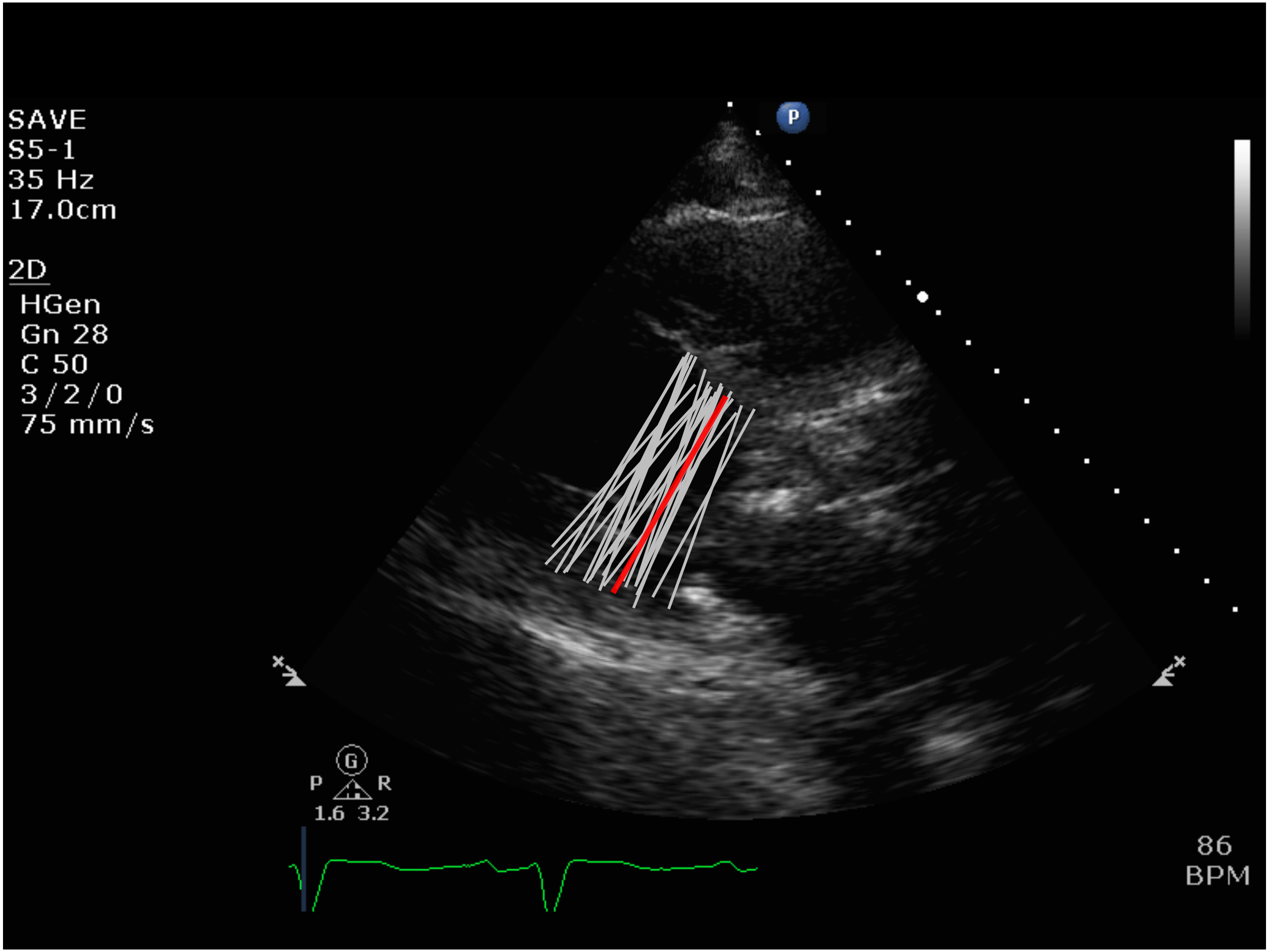

Pt 18 Diastole

Absolute difference between AI and expert consensus: 1.3 mm

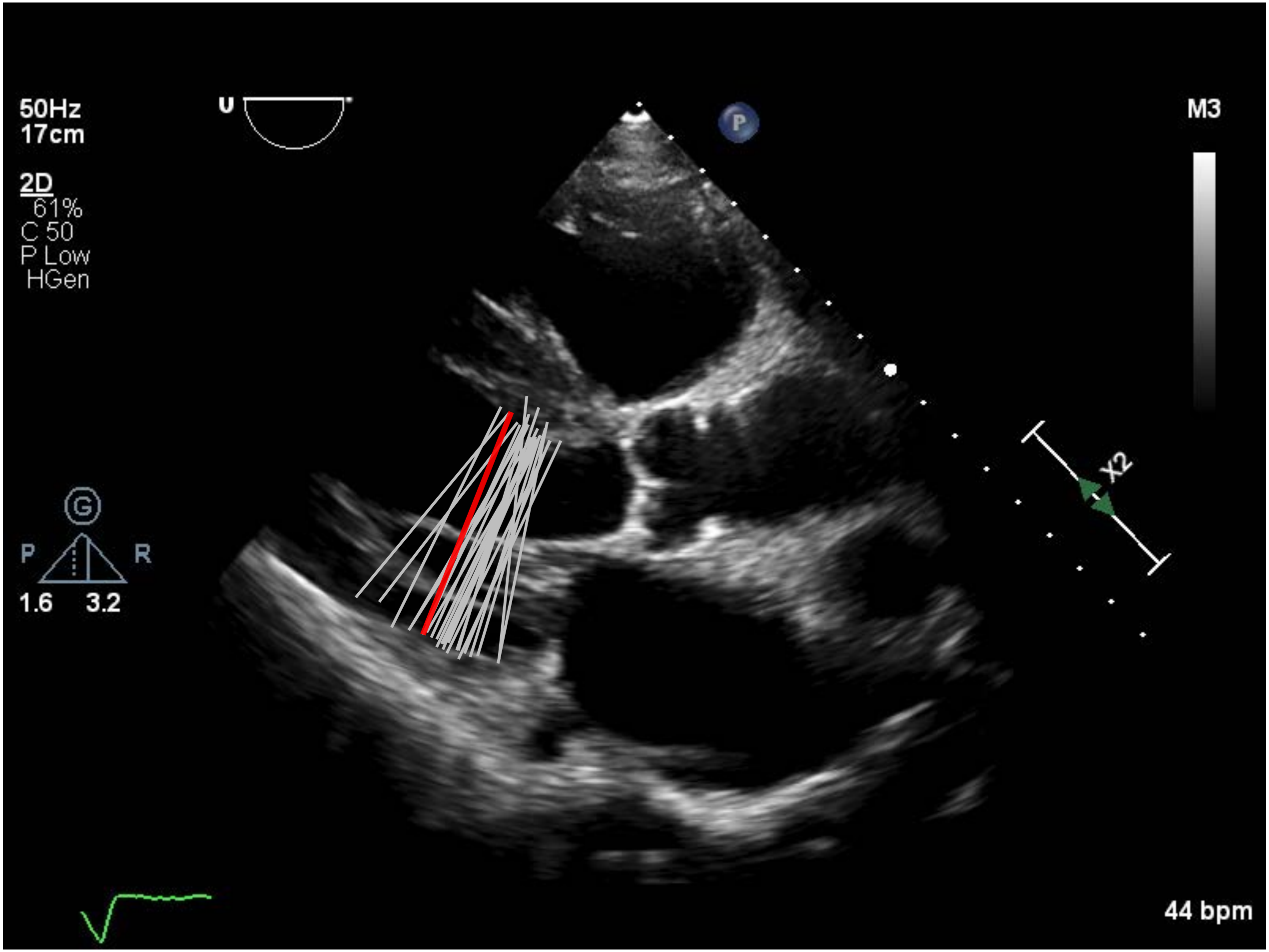

Pt 52 Systole

Absolute difference between AI and expert consensus: 1.3 mm

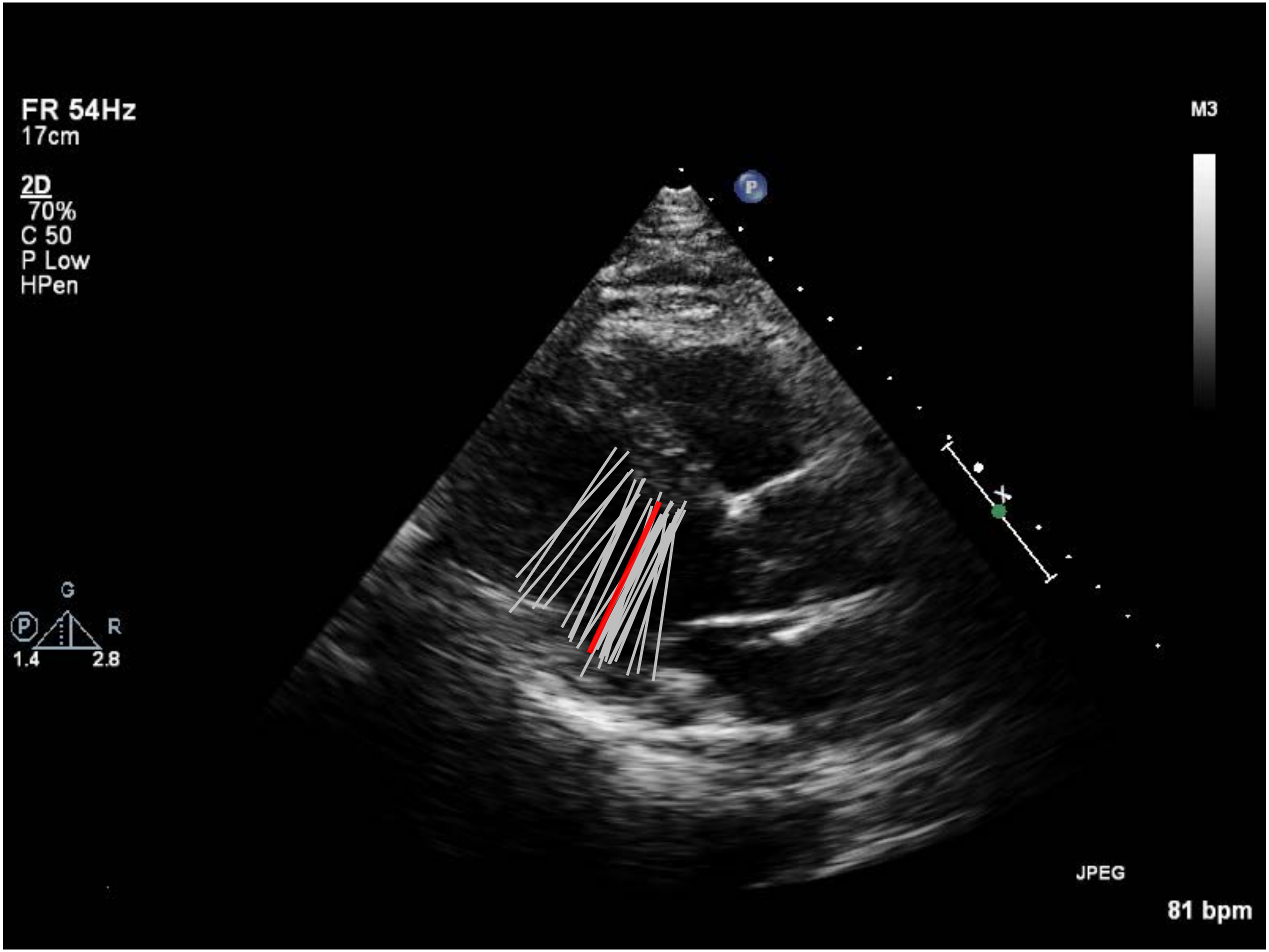

Pt 90 Diastole

Absolute difference between AI and expert consensus: 1.3 mm

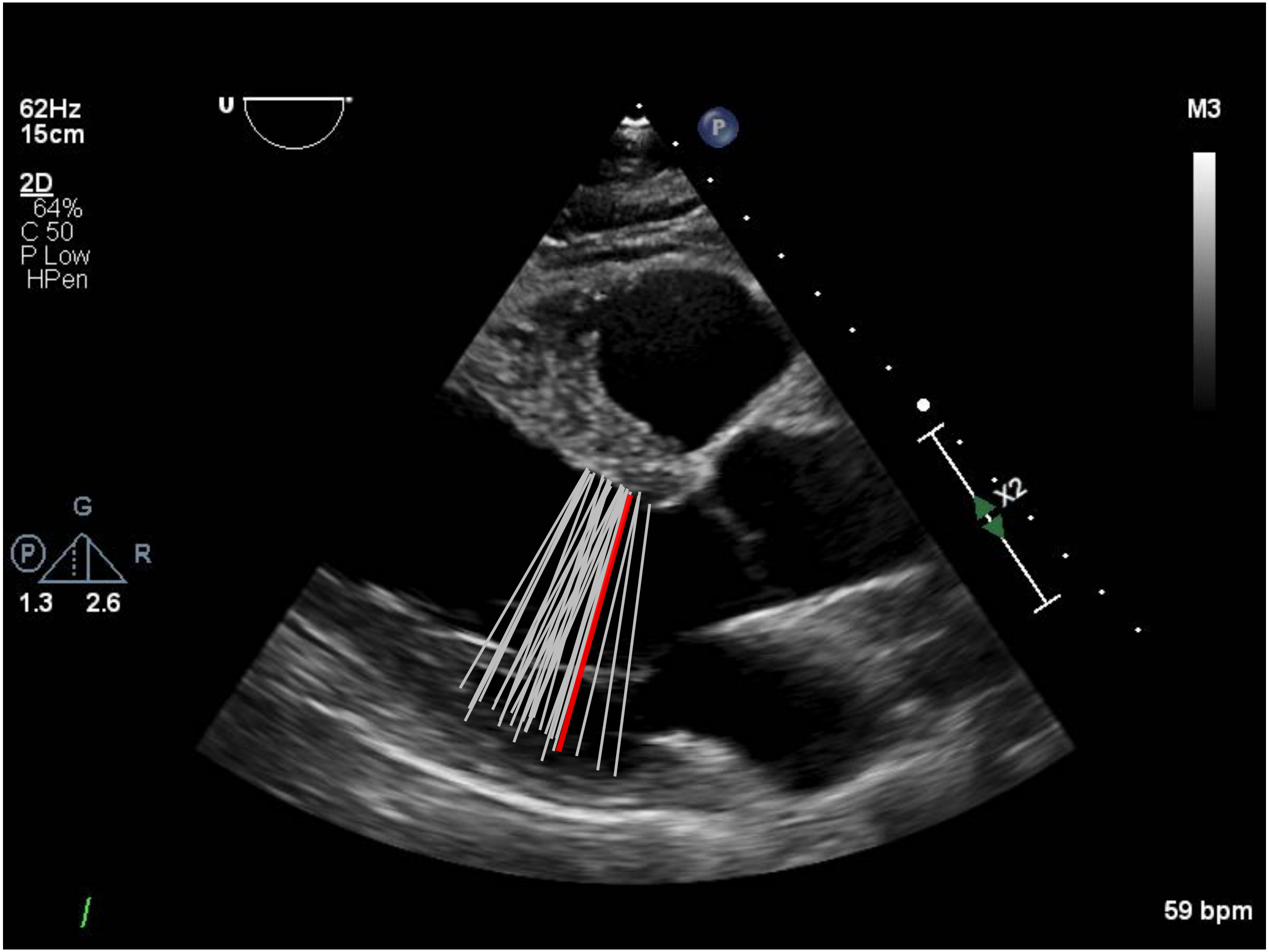

Pt 60 Diastole

Absolute difference between AI and expert consensus: 1.3 mm

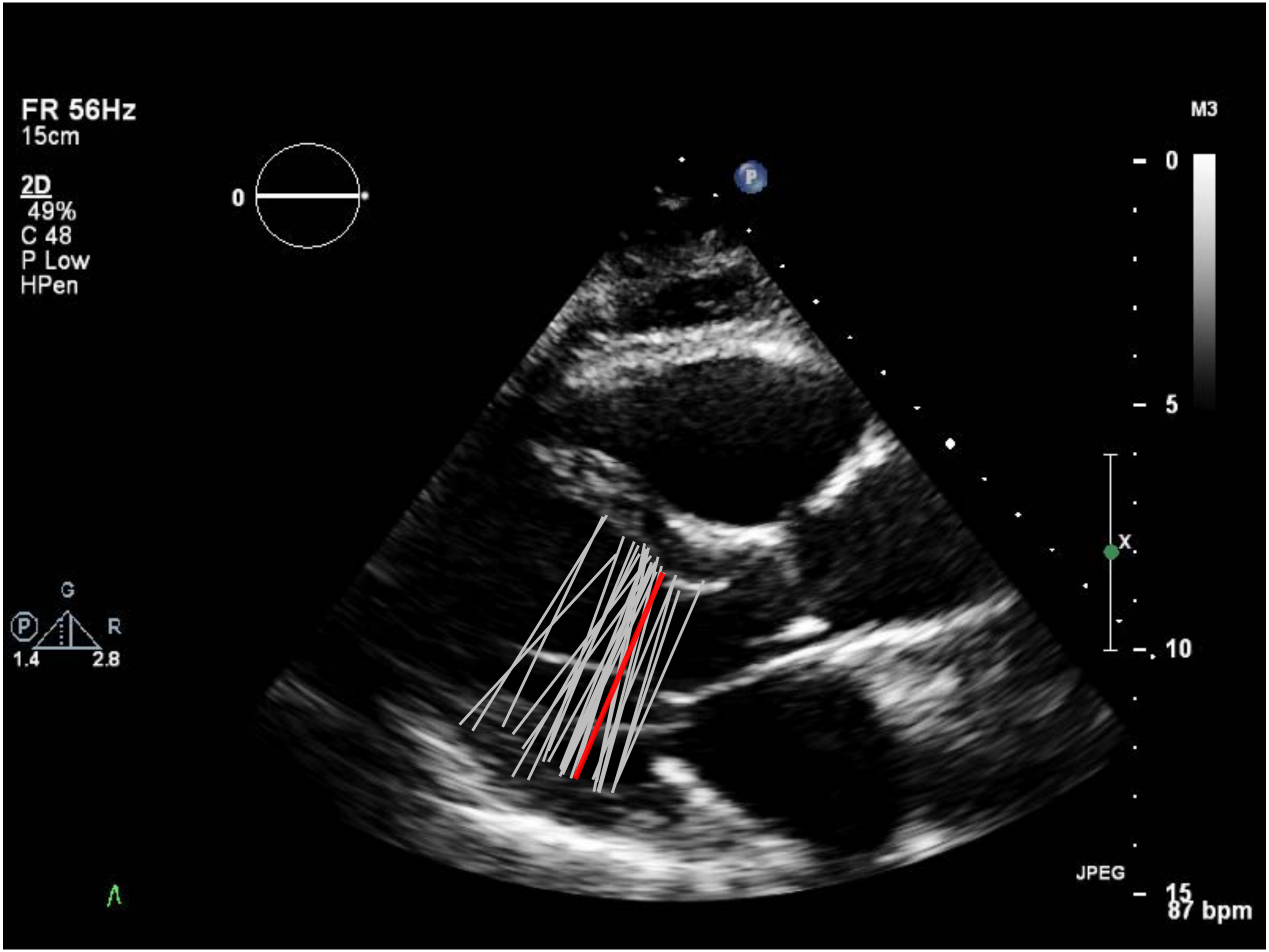

Pt 21 Diastole

Absolute difference between AI and expert consensus: 1.3 mm

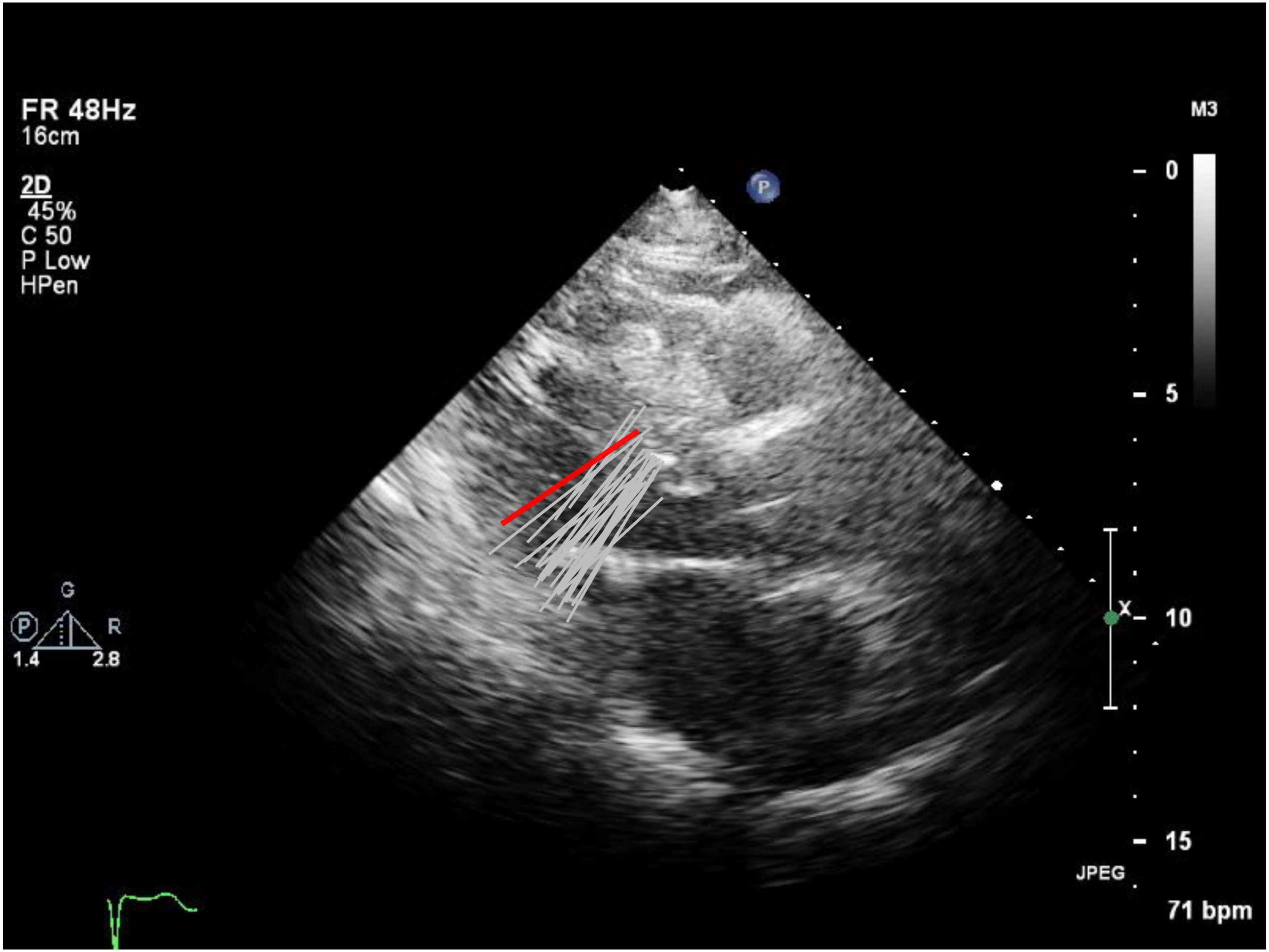

Pt 71 Systole

Absolute difference between AI and expert consensus: 1.4 mm

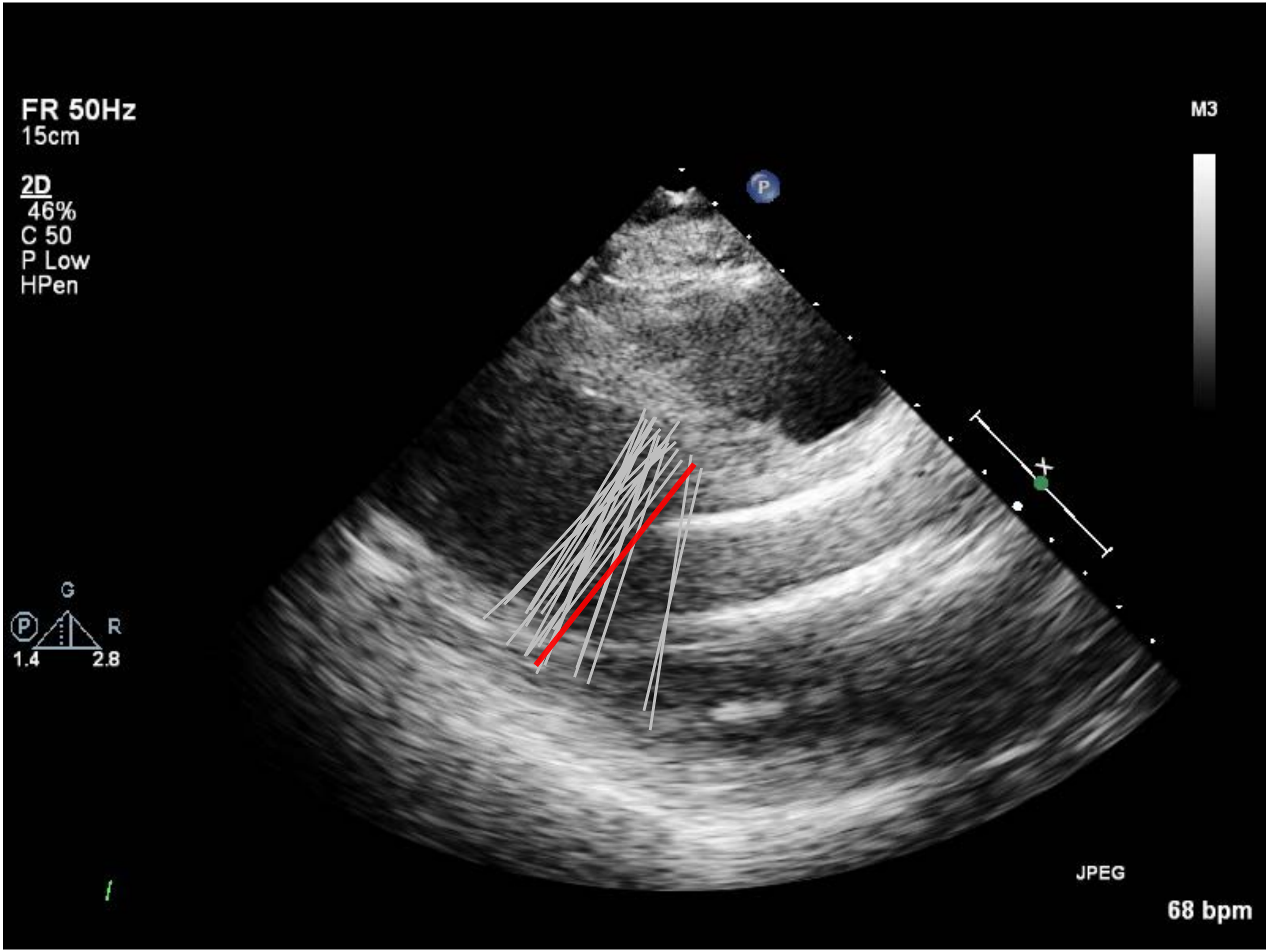

Pt 81 Diastole

Absolute difference between AI and expert consensus: 1.4 mm

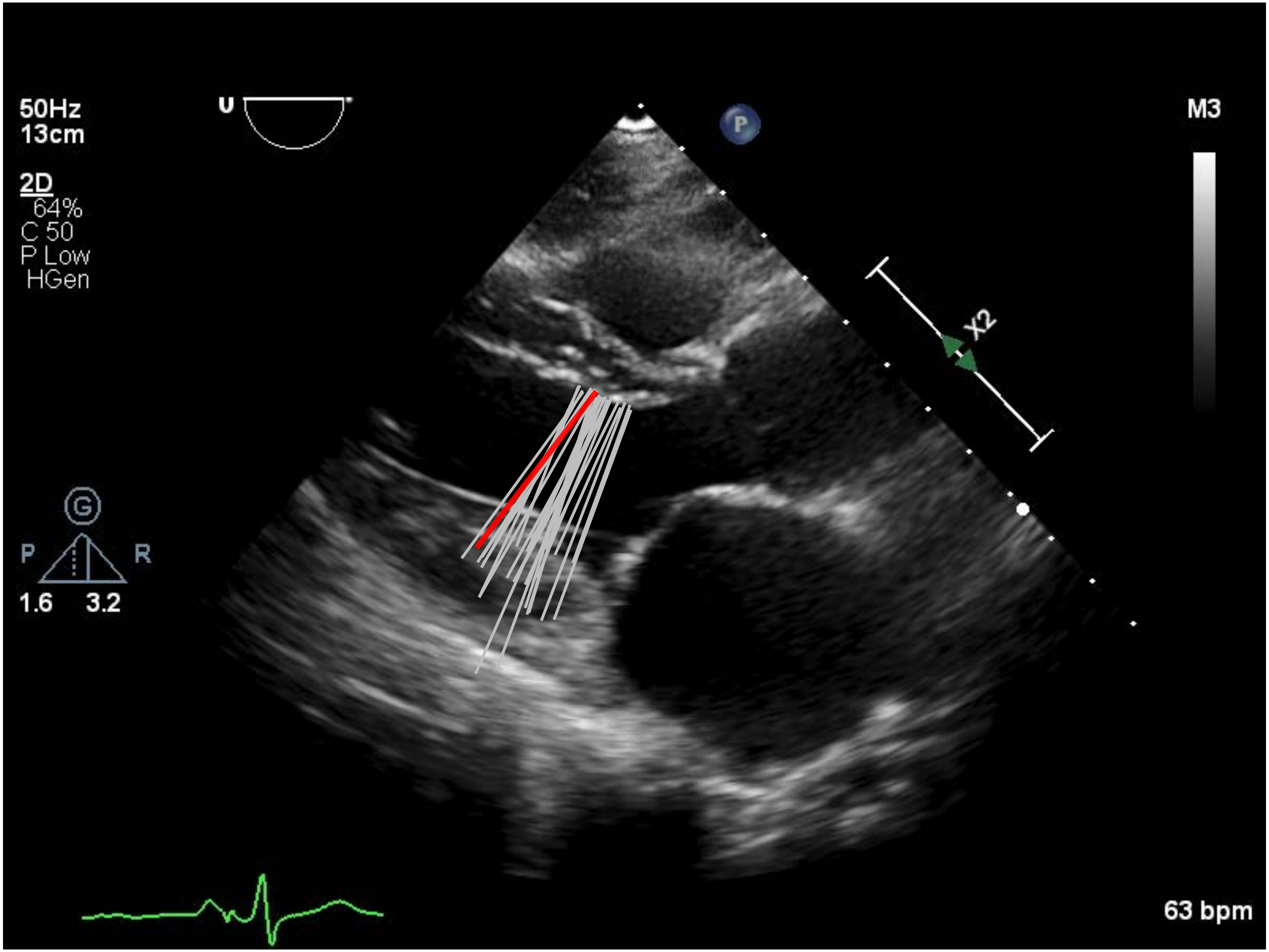

Pt 16 Systole

Absolute difference between AI and expert consensus: 1.4 mm

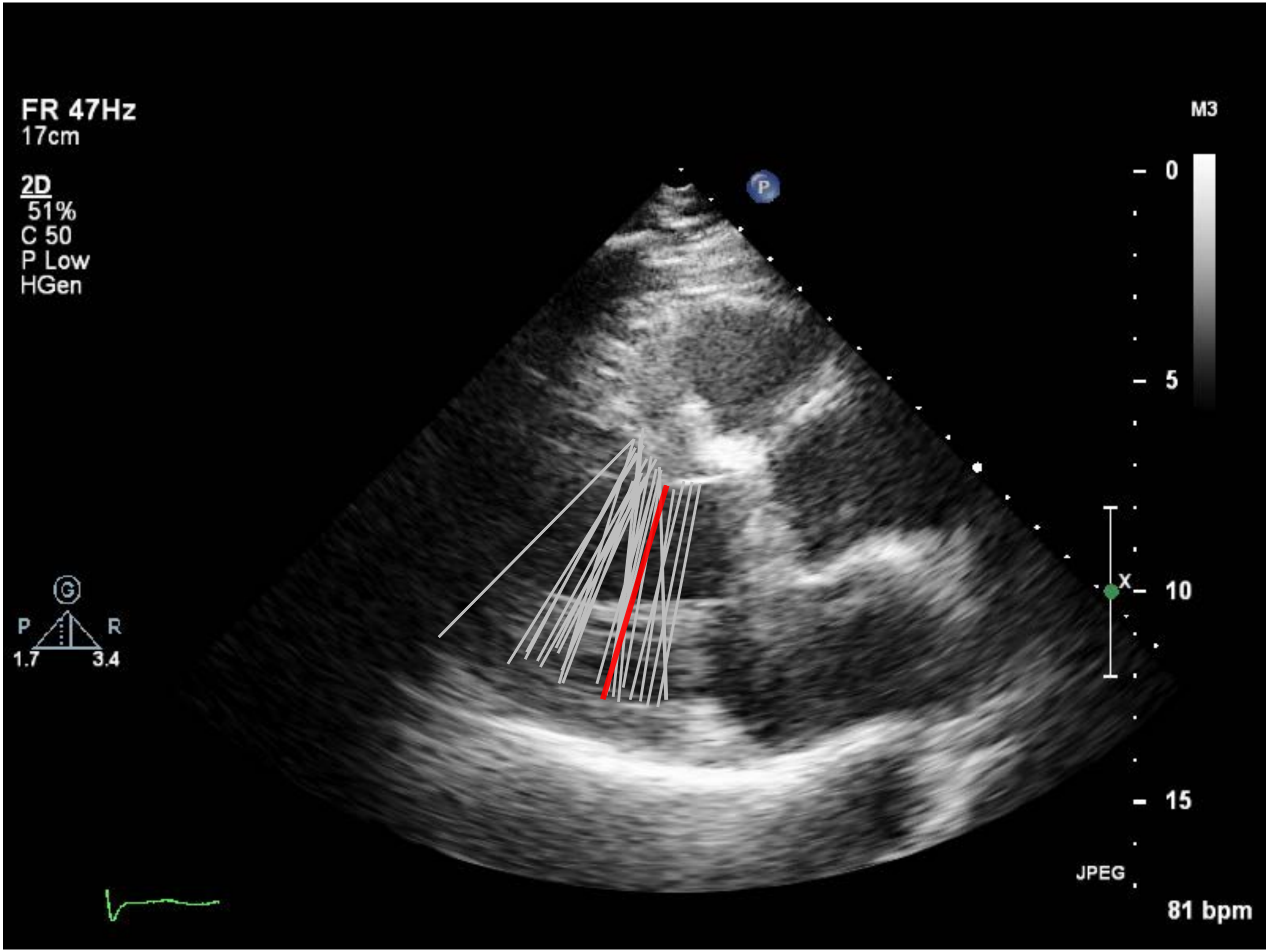

Pt 32 Systole

Absolute difference between AI and expert consensus: 1.4 mm

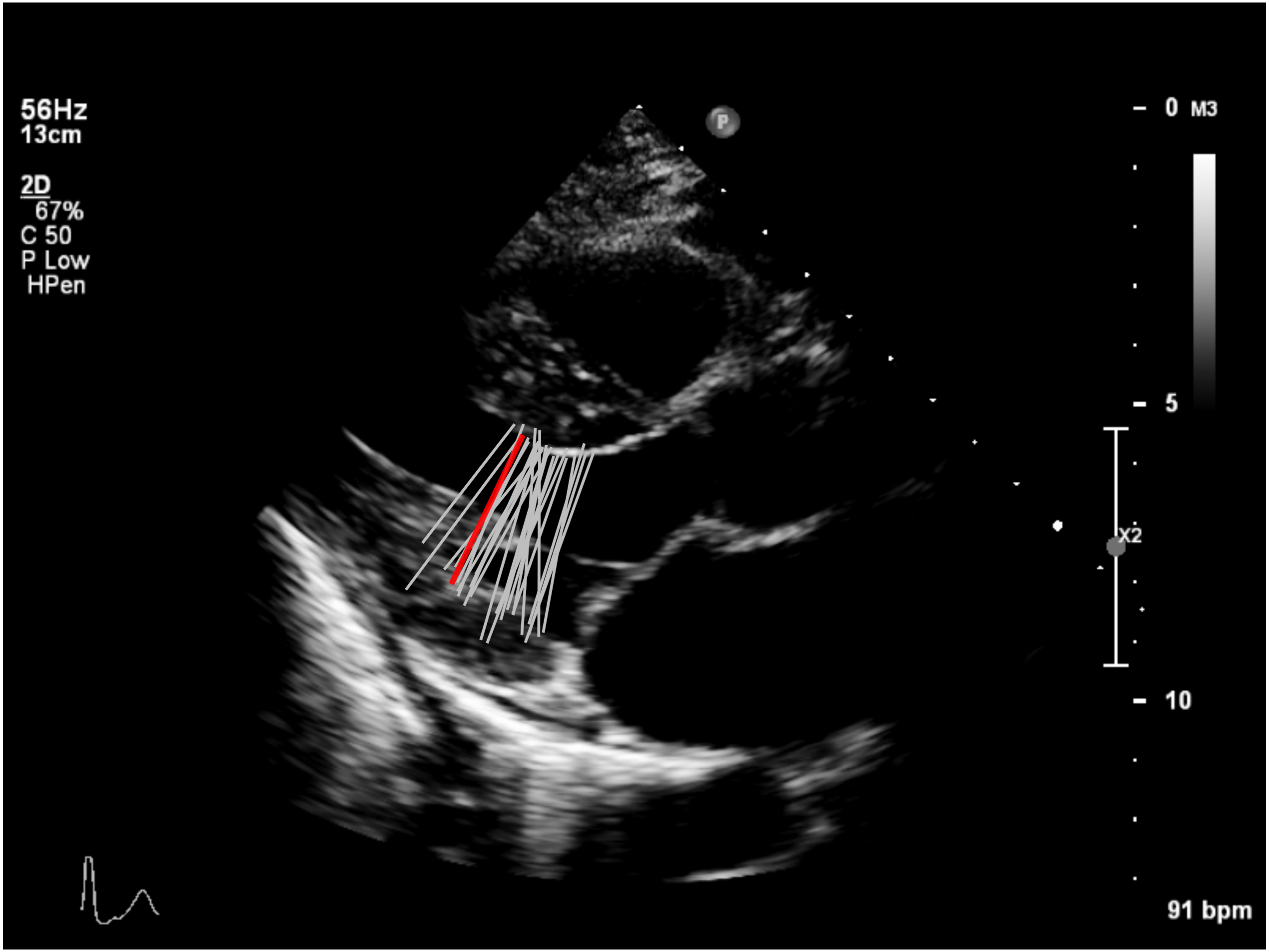

Pt 54 Systole

Absolute difference between AI and expert consensus: 1.4 mm

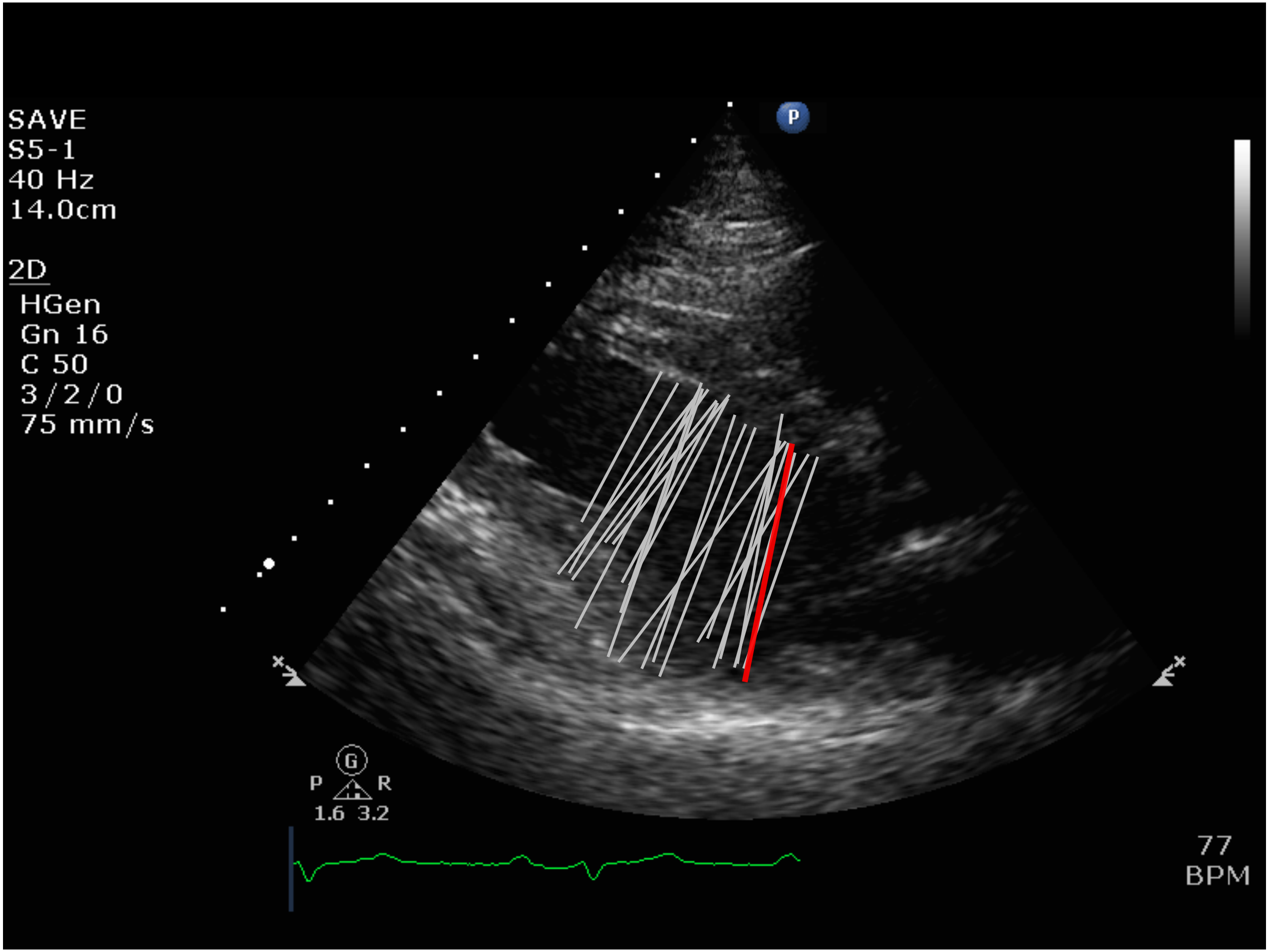

Pt 76 Diastole

Absolute difference between AI and expert consensus: 1.4 mm

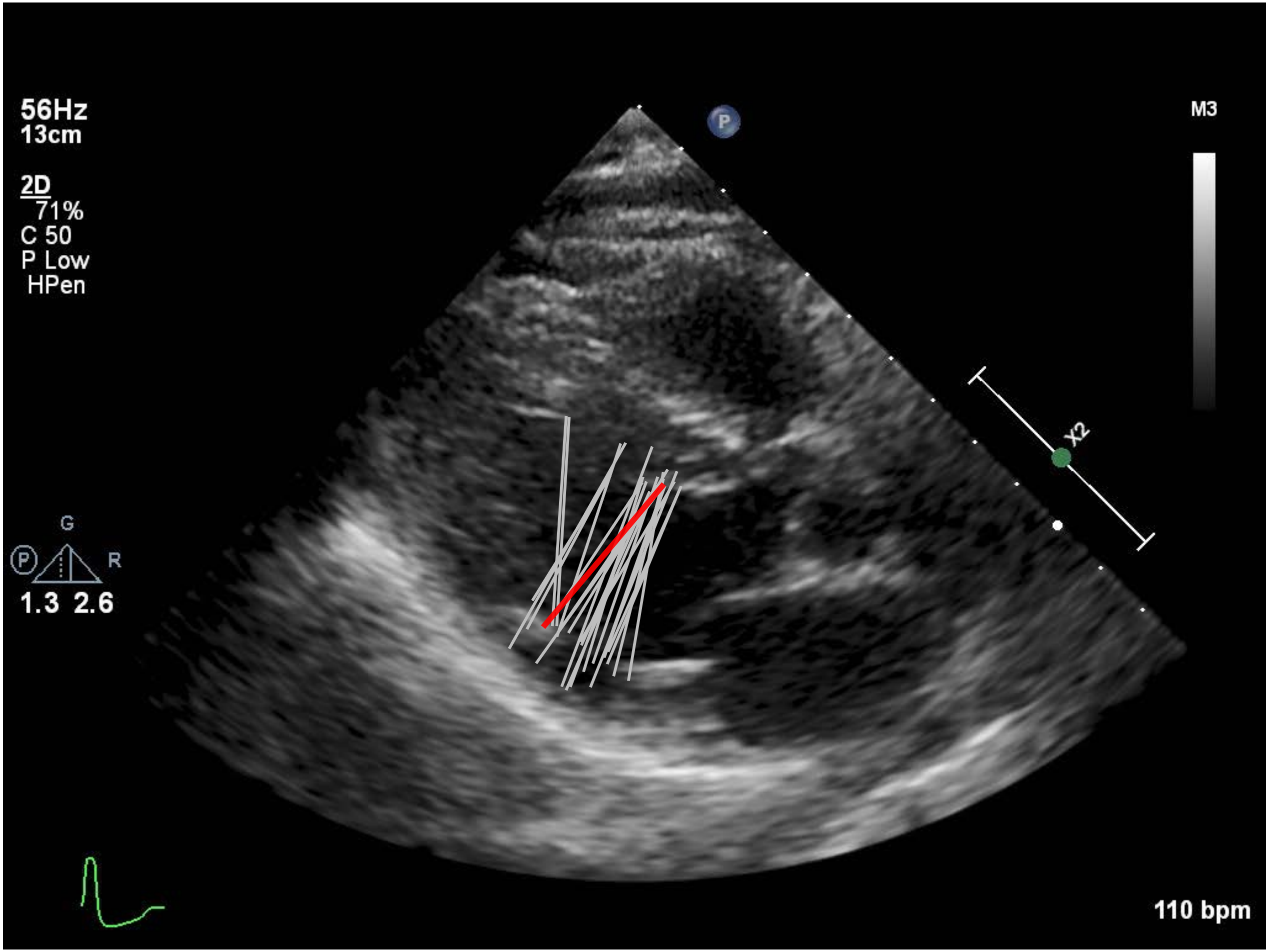

Pt 26 Systole
Absolute difference between AI and expert consensus: 1.5 mm

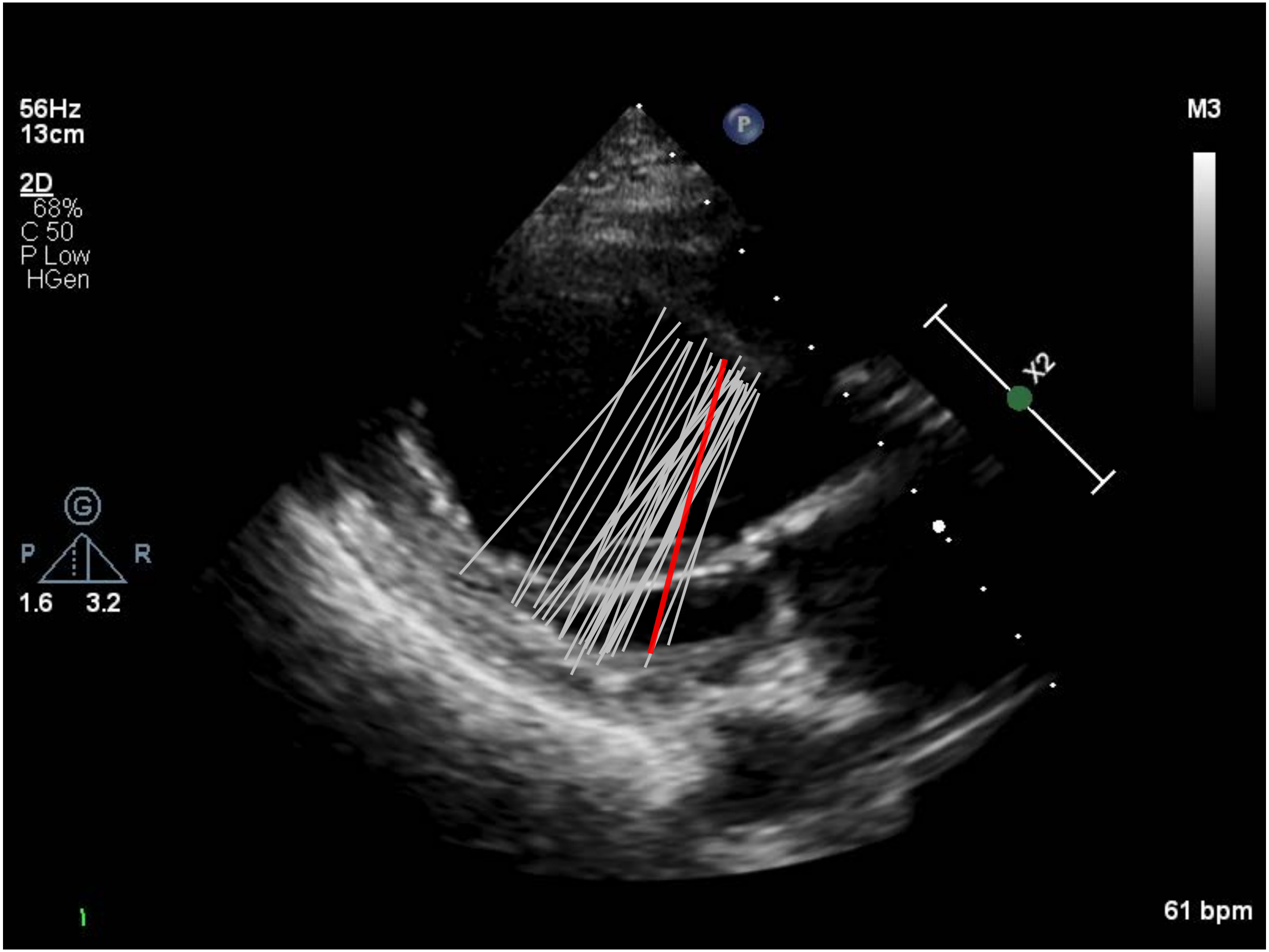

Pt 44 Diastole

Absolute difference between AI and expert consensus: 1.5 mm

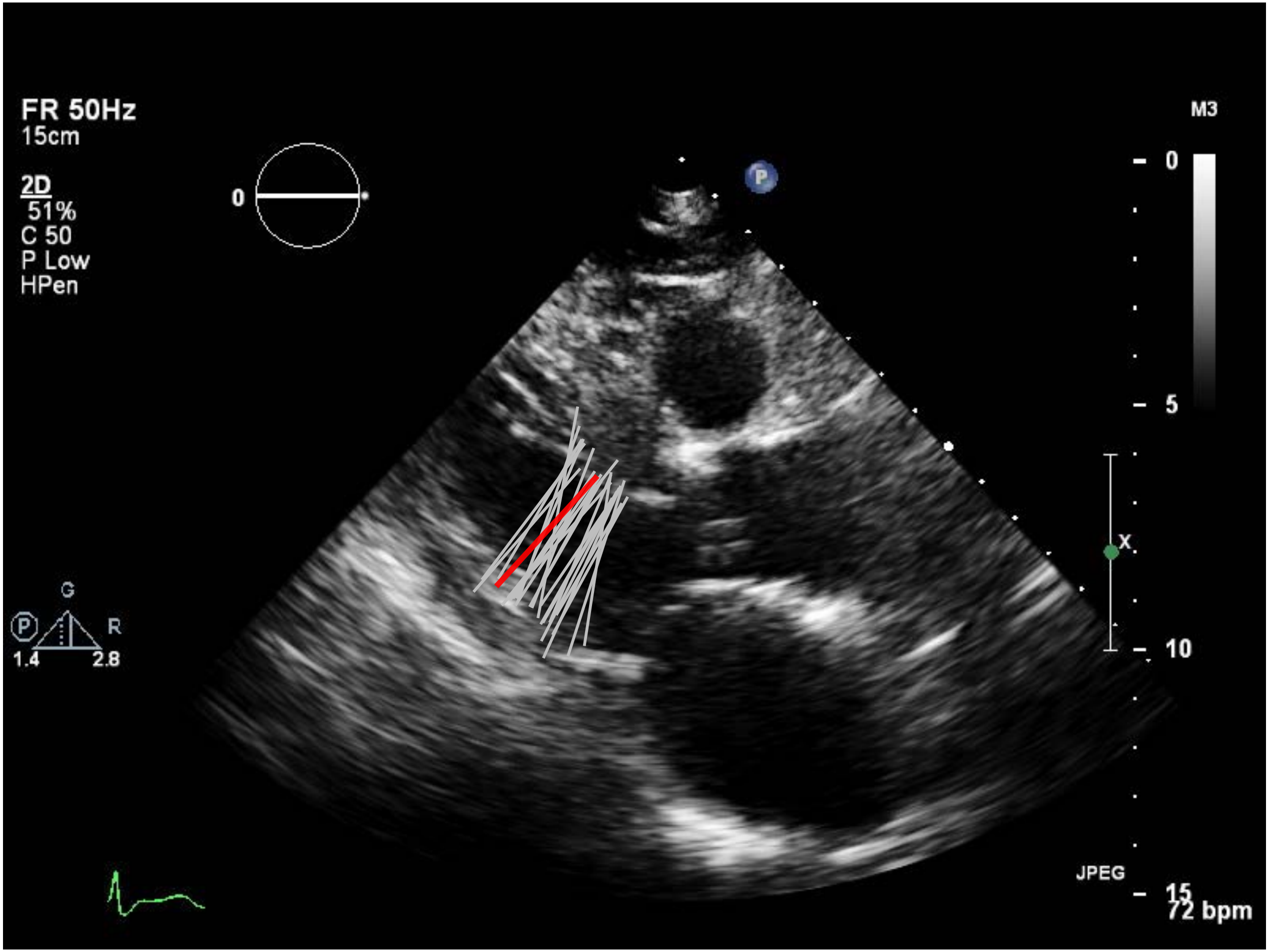

Pt 30 Systole

Absolute difference between AI and expert consensus: 1.5 mm

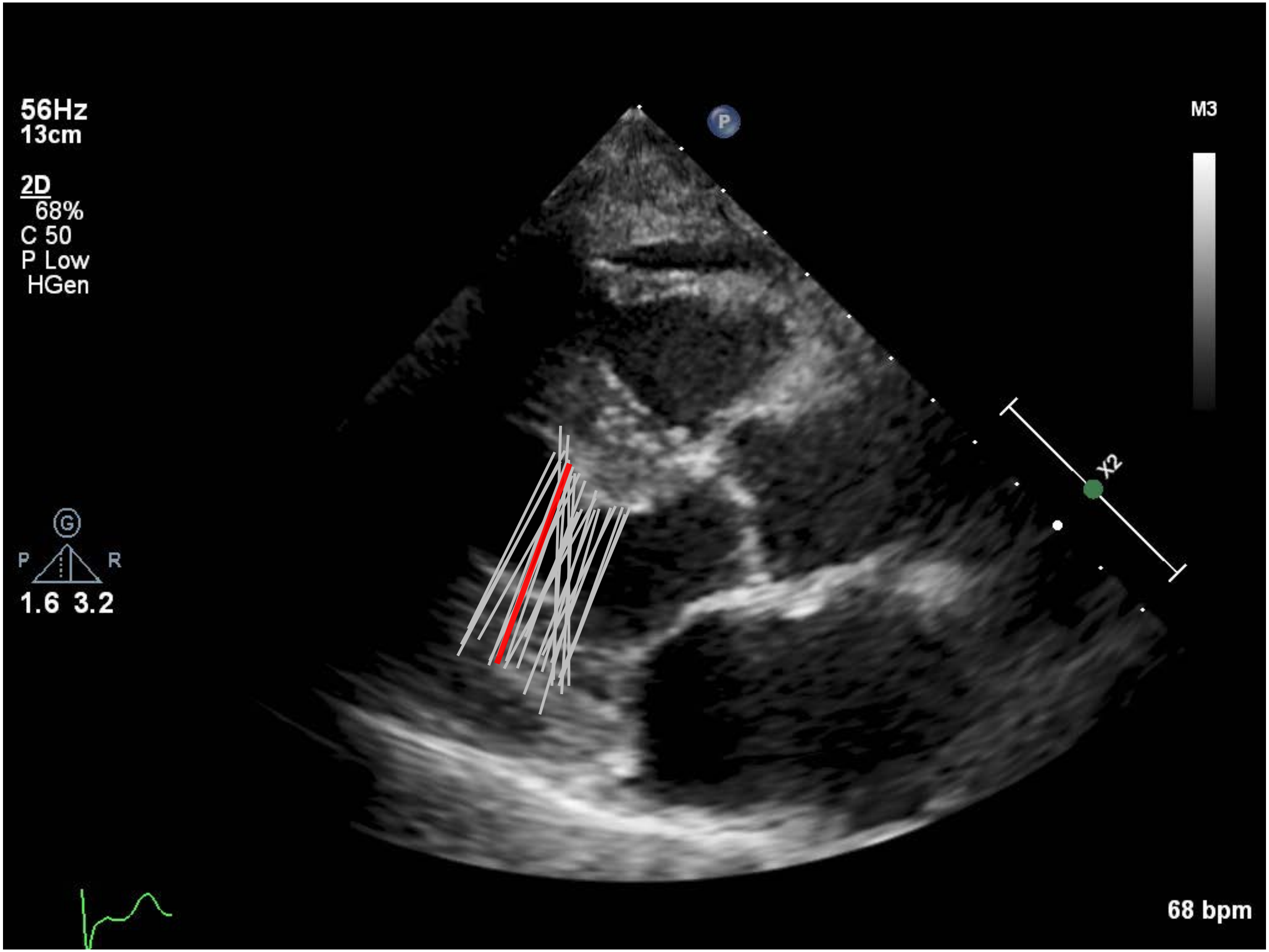

Pt 57 Systole

Absolute difference between AI and expert consensus: 1.5 mm

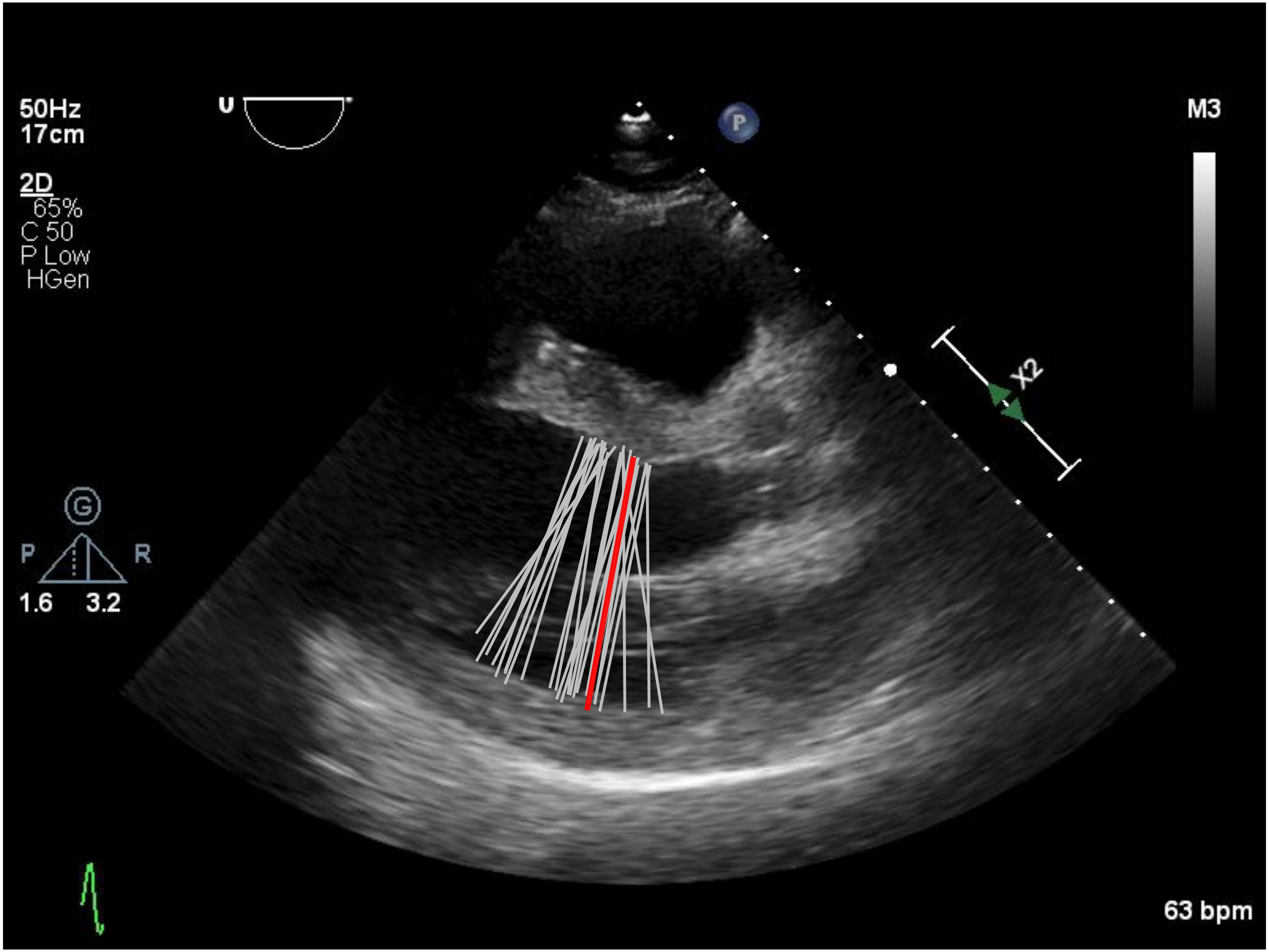

Pt 68 Diastole

Absolute difference between AI and expert consensus: 1.6 mm

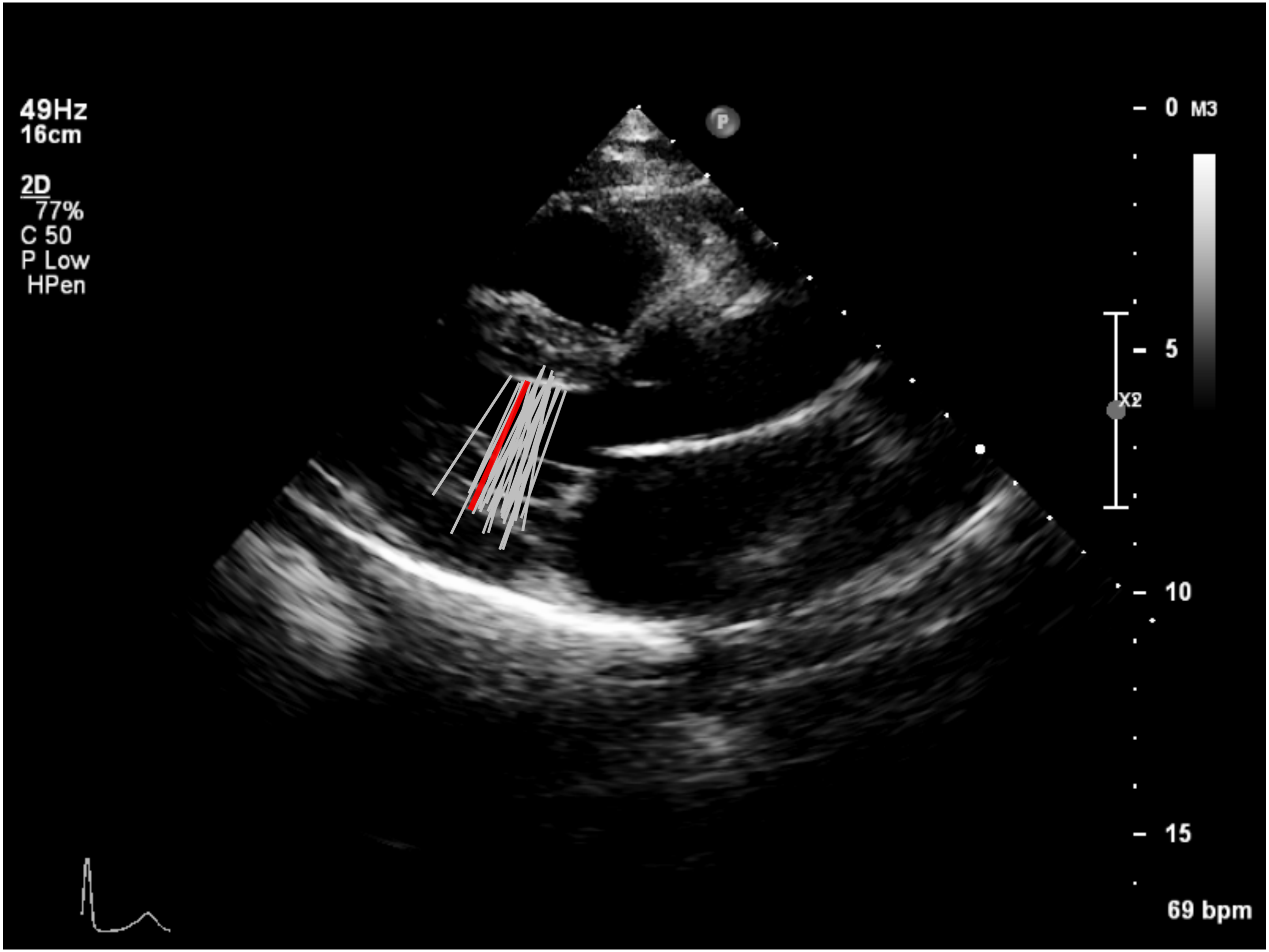

Pt 38 Systole

Absolute difference between AI and expert consensus: 1.6 mm

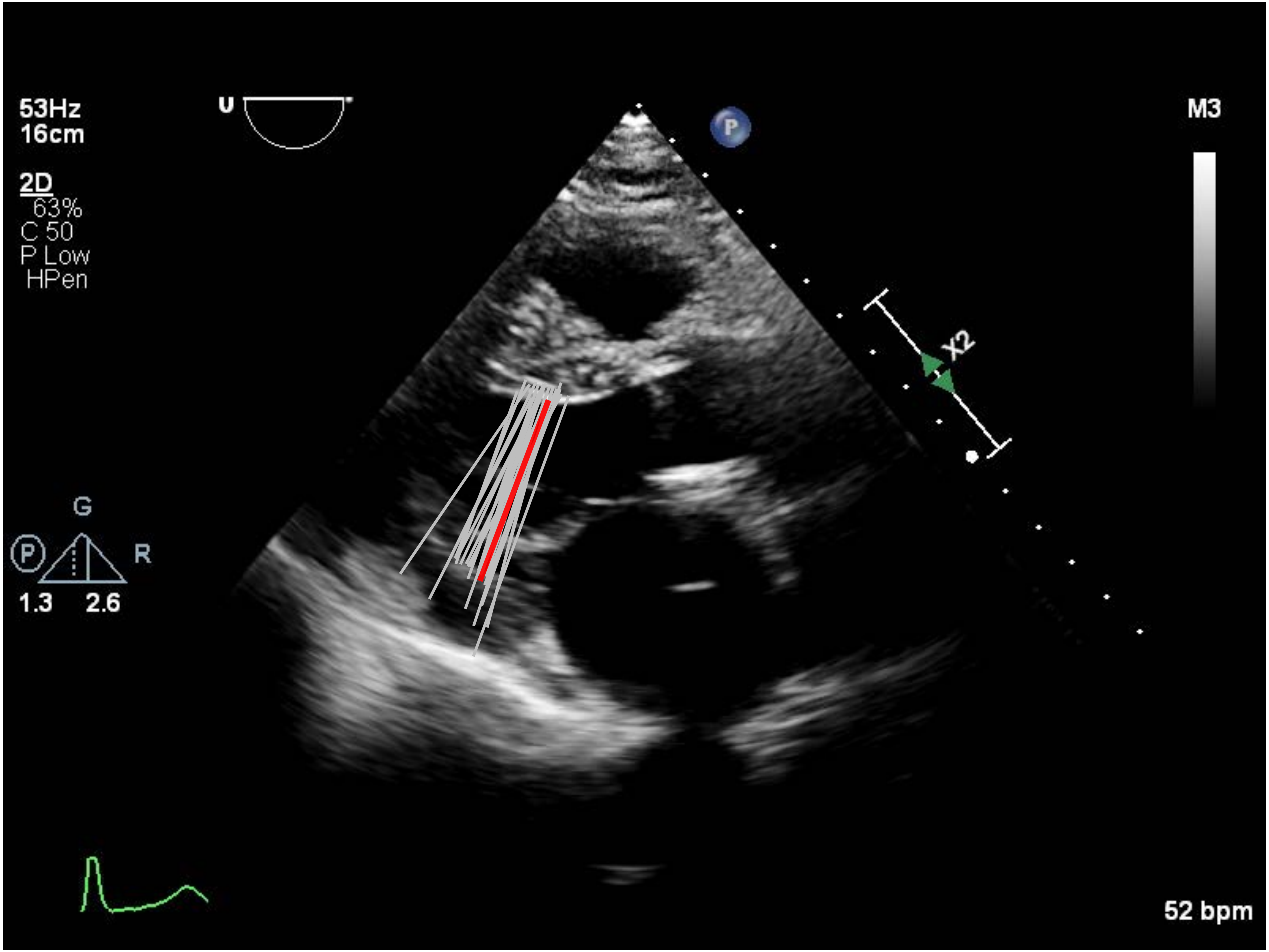

Pt 8 Systole

Absolute difference between AI and expert consensus: 1.6 mm

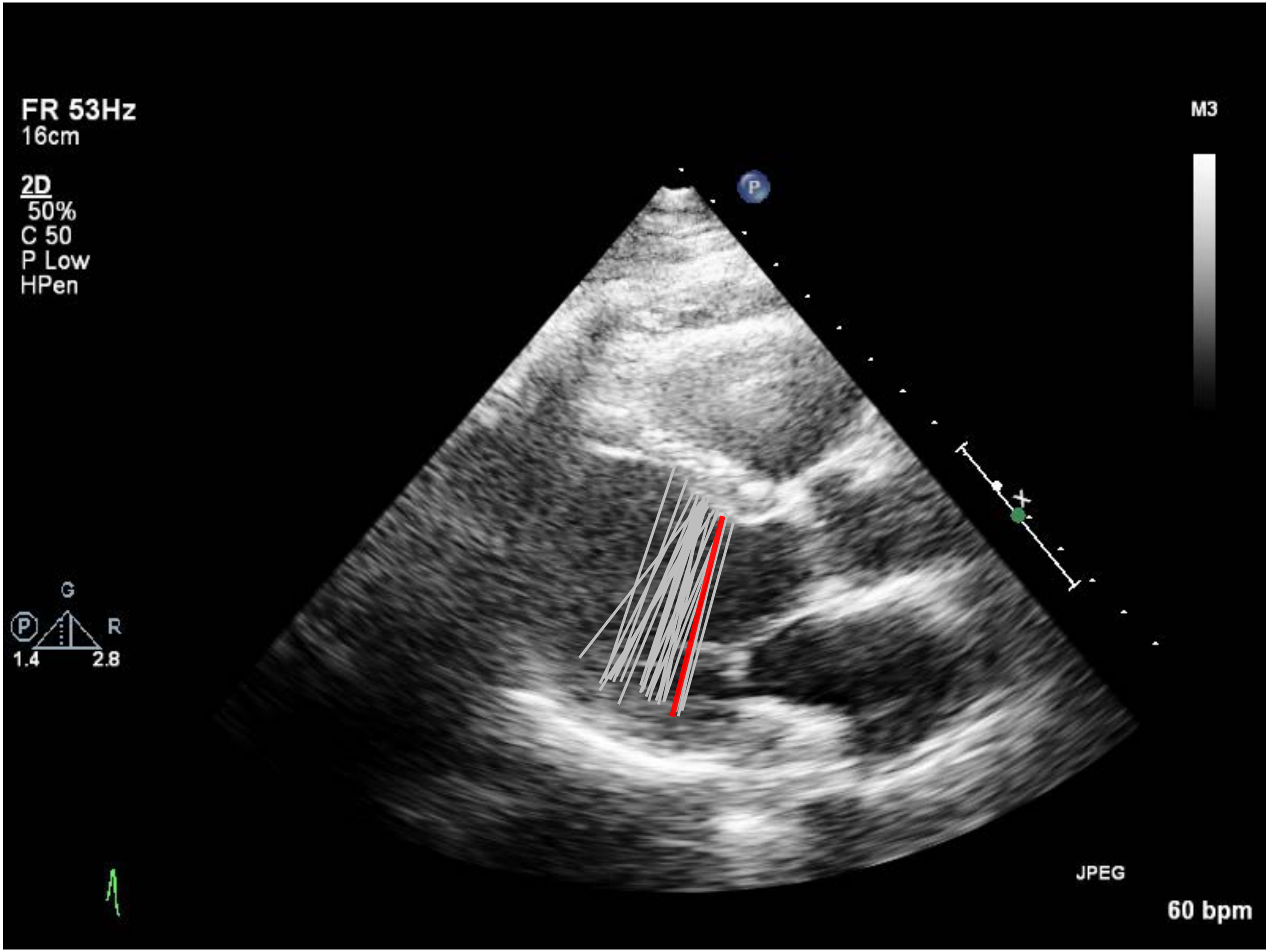

Pt 47 Diastole

Absolute difference between AI and expert consensus: 1.6 mm

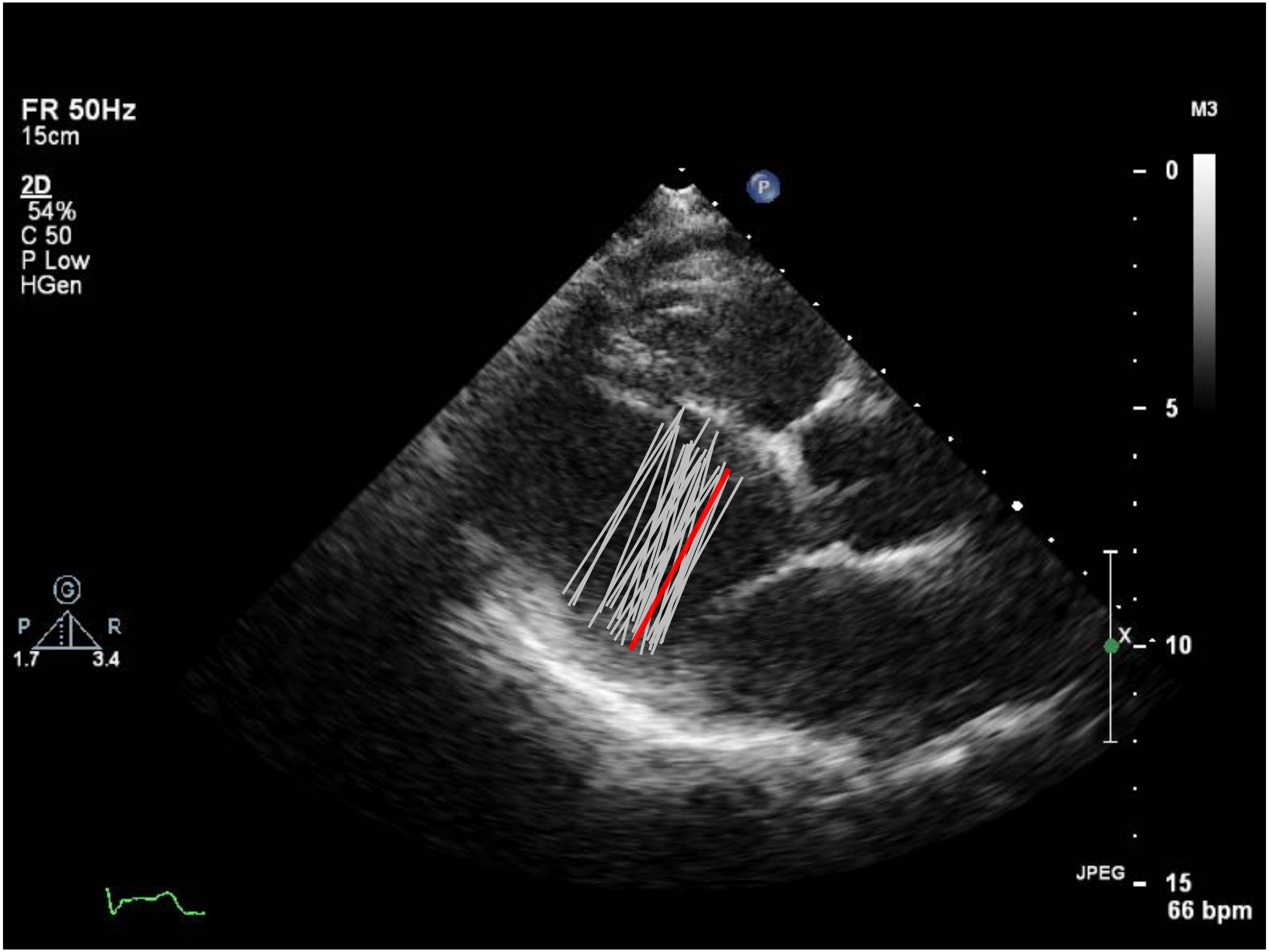

Pt 33 Systole

Absolute difference between AI and expert consensus: 1.6 mm

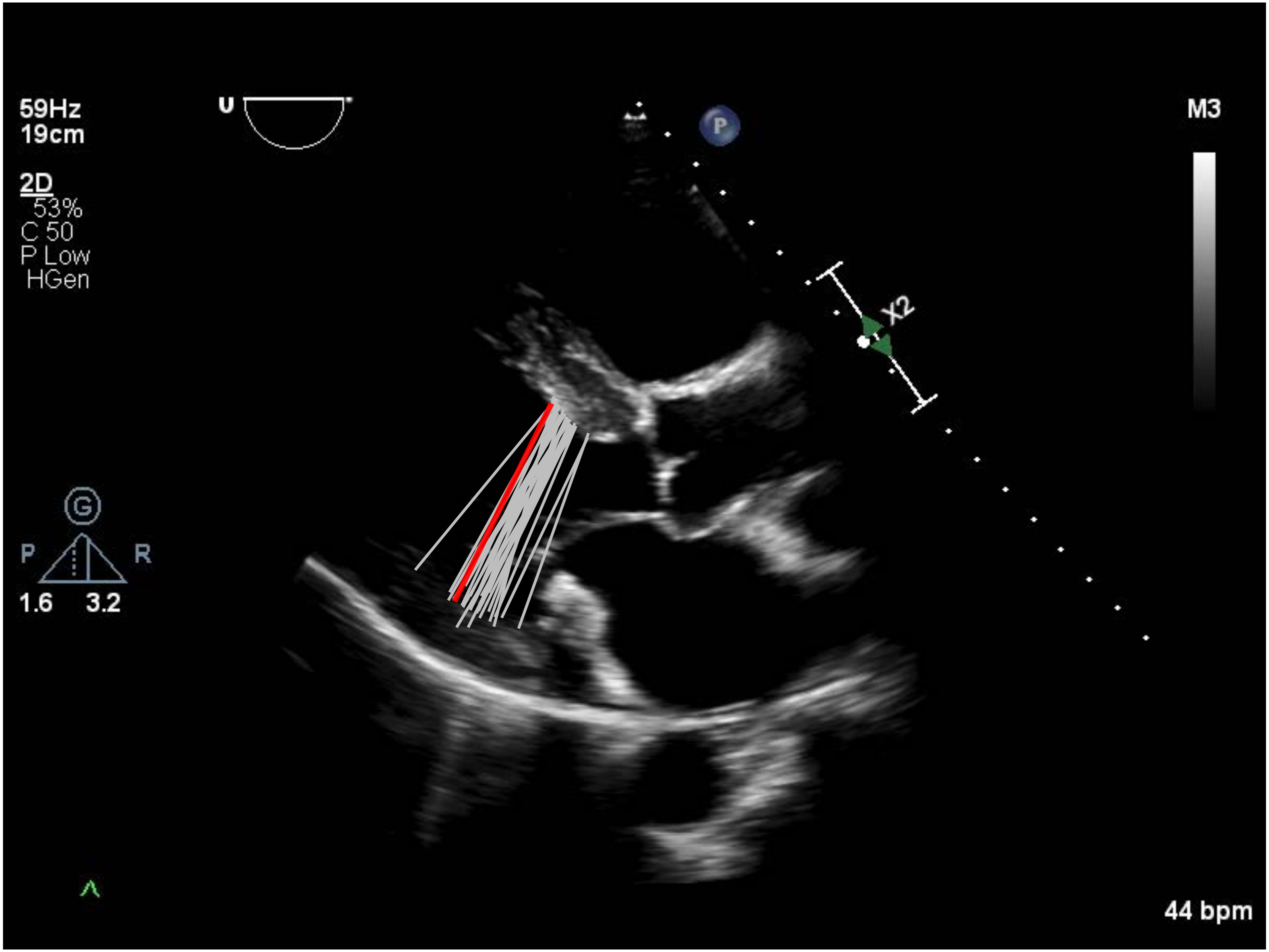

Pt 13 Diastole

Absolute difference between AI and expert consensus: 1.6 mm

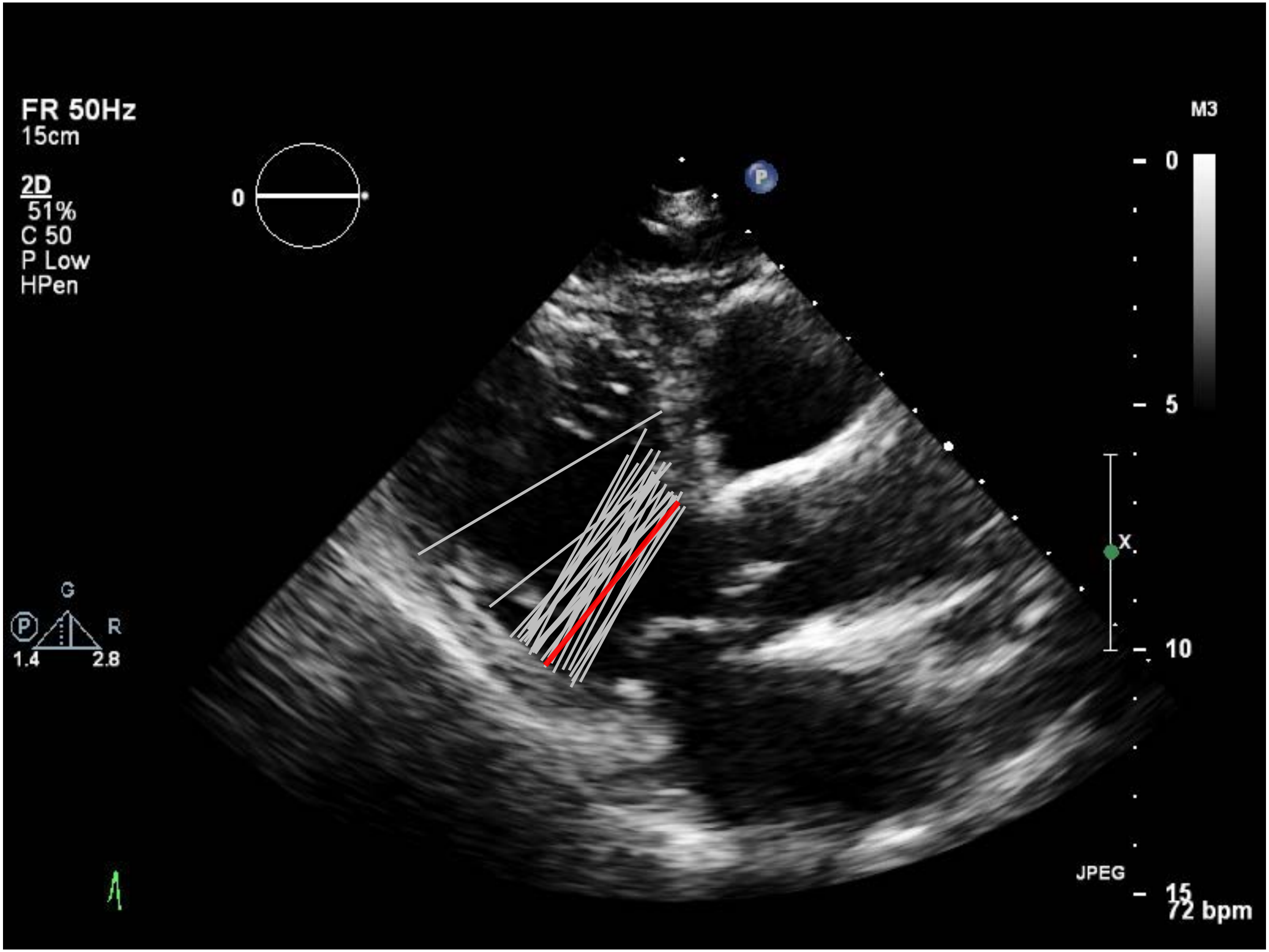

Pt 30 Diastole

Absolute difference between AI and expert consensus: 1.6 mm

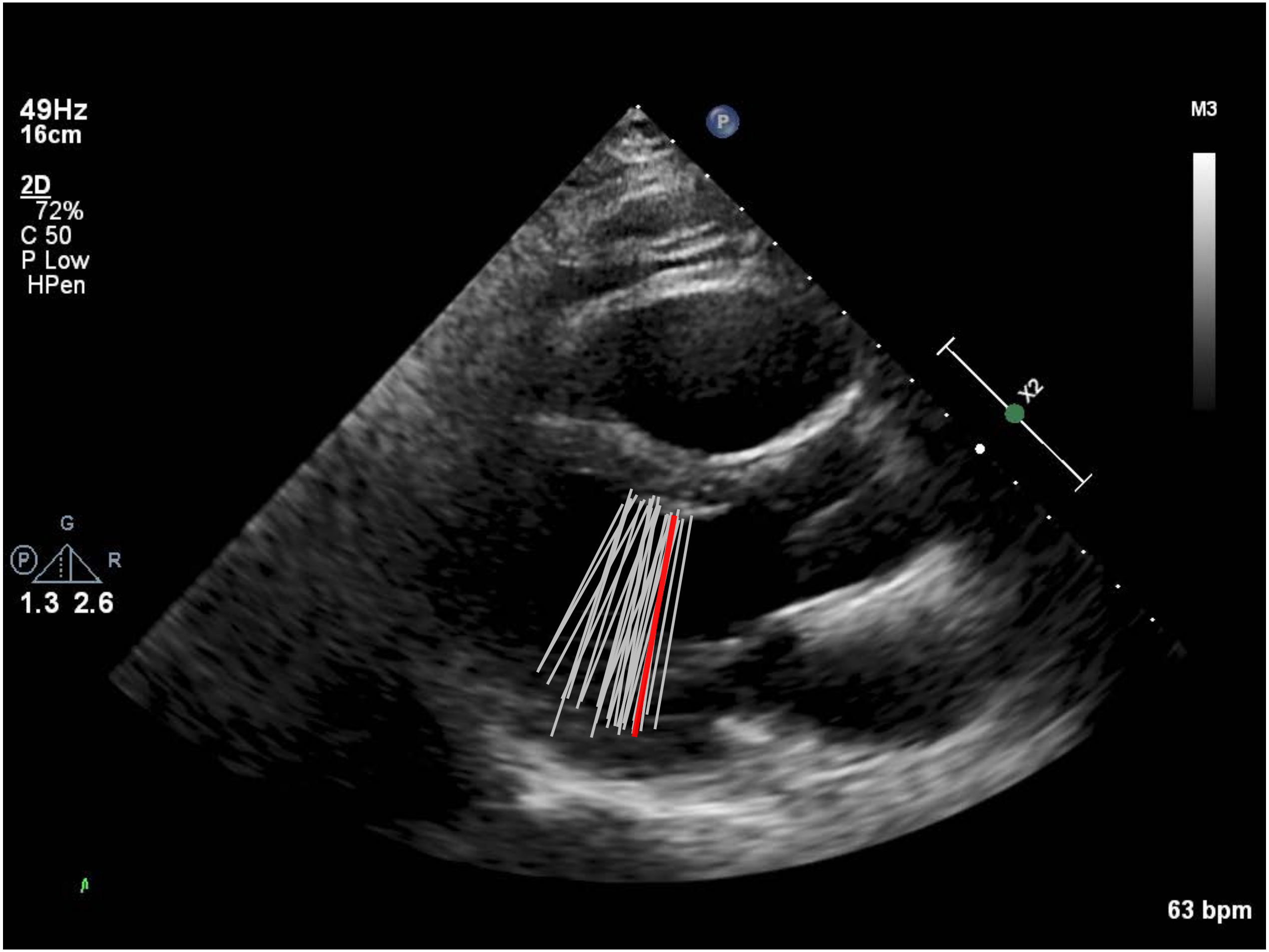

Pt 24 Diastole
Absolute difference between AI and expert consensus: 1.6 mm

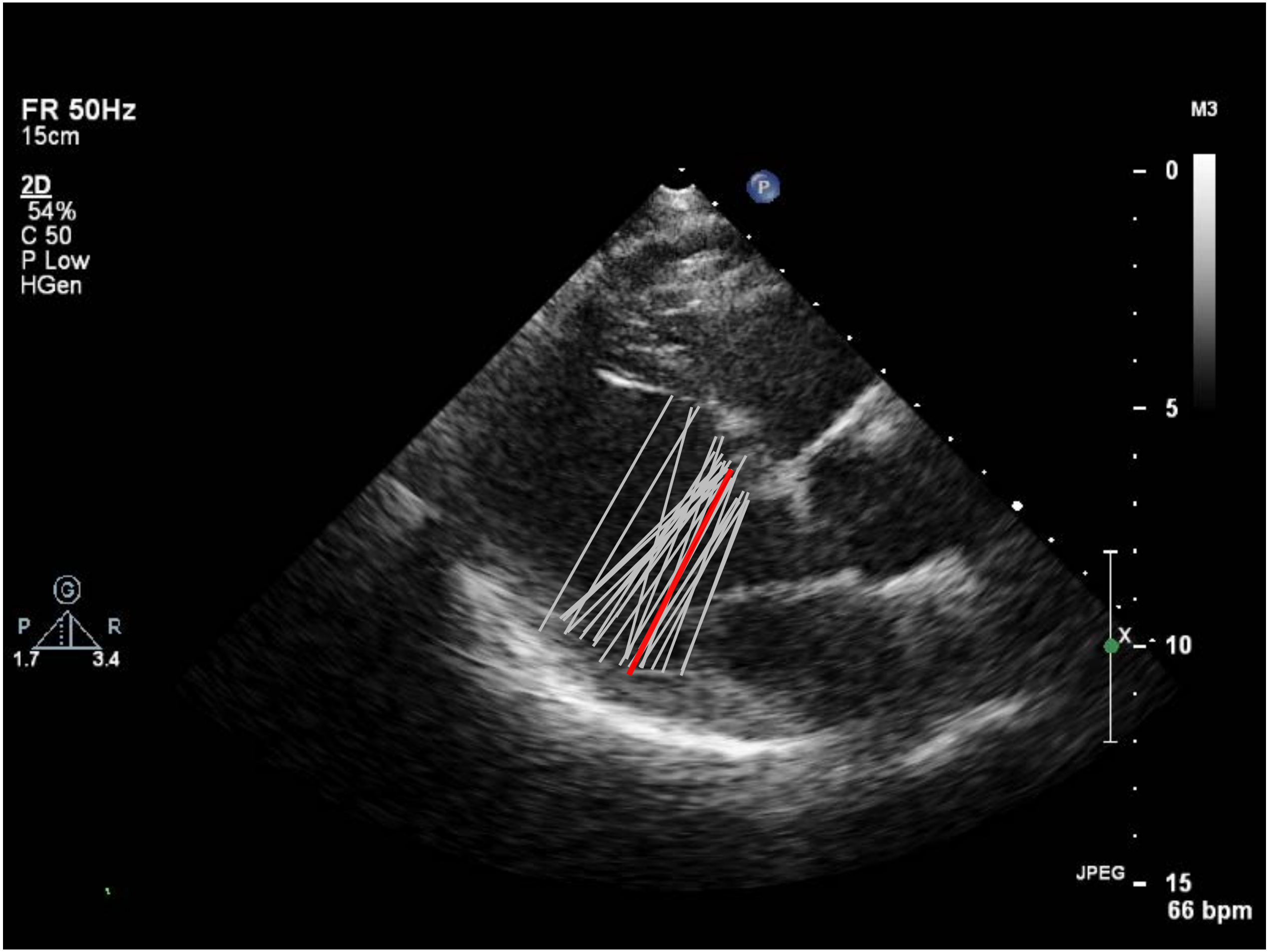

Pt 33 Diastole

Absolute difference between AI and expert consensus: 1.7 mm

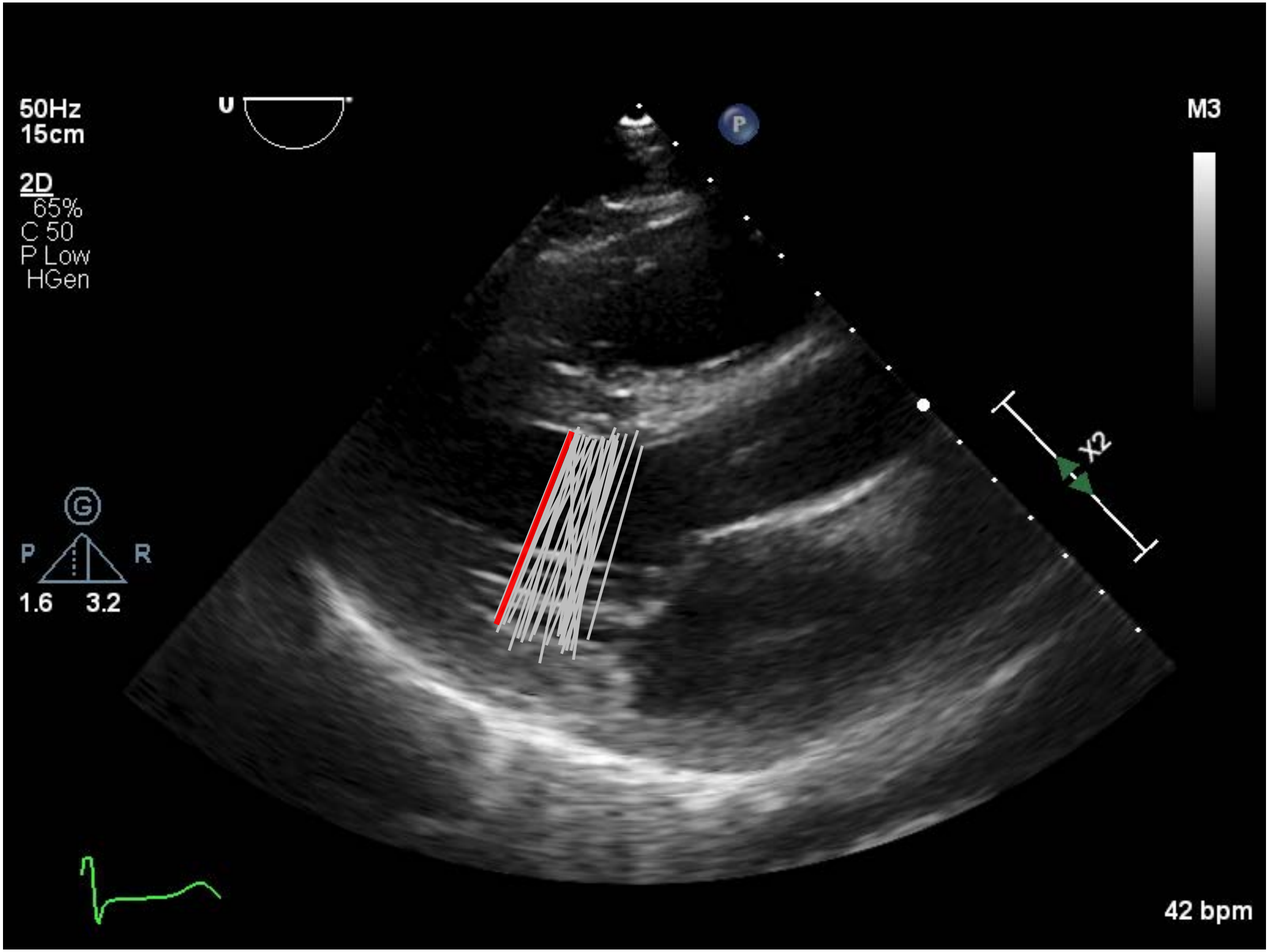

Pt 22 Systole

Absolute difference between AI and expert consensus: 1.7 mm

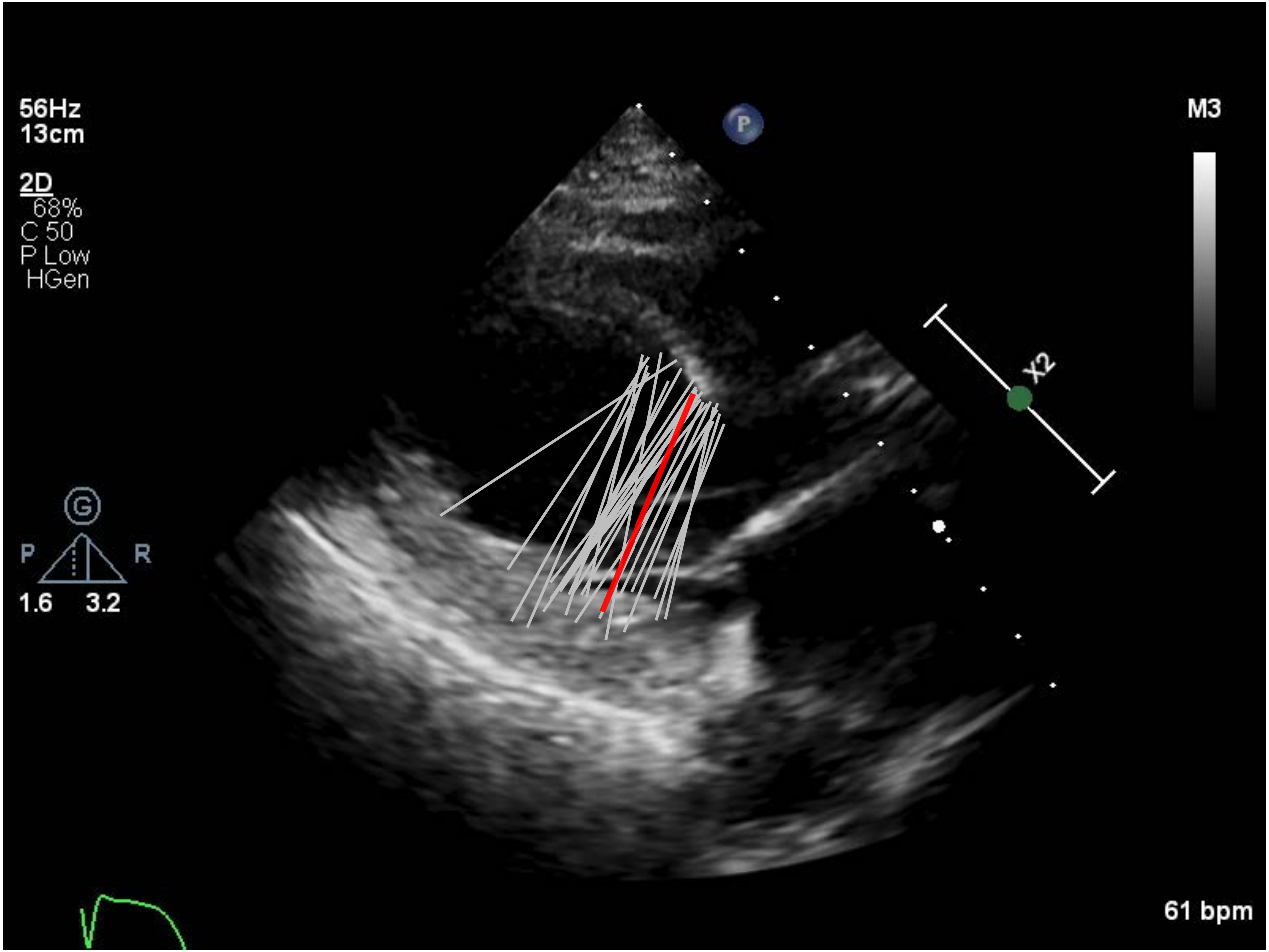

Pt 44 Systole

Absolute difference between AI and expert consensus: 1.8 mm

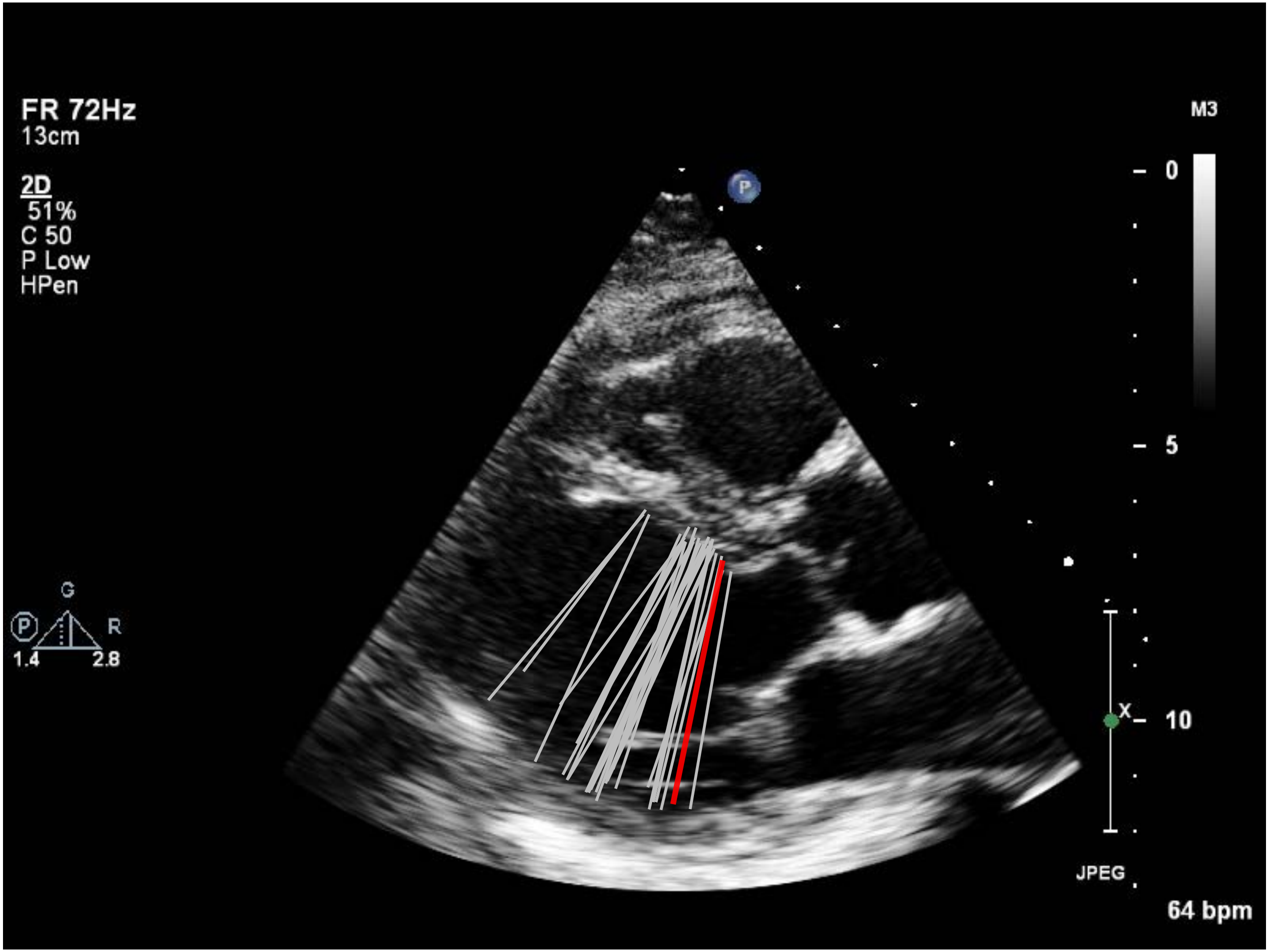

Pt 48 Diastole

Absolute difference between AI and expert consensus: 1.8 mm

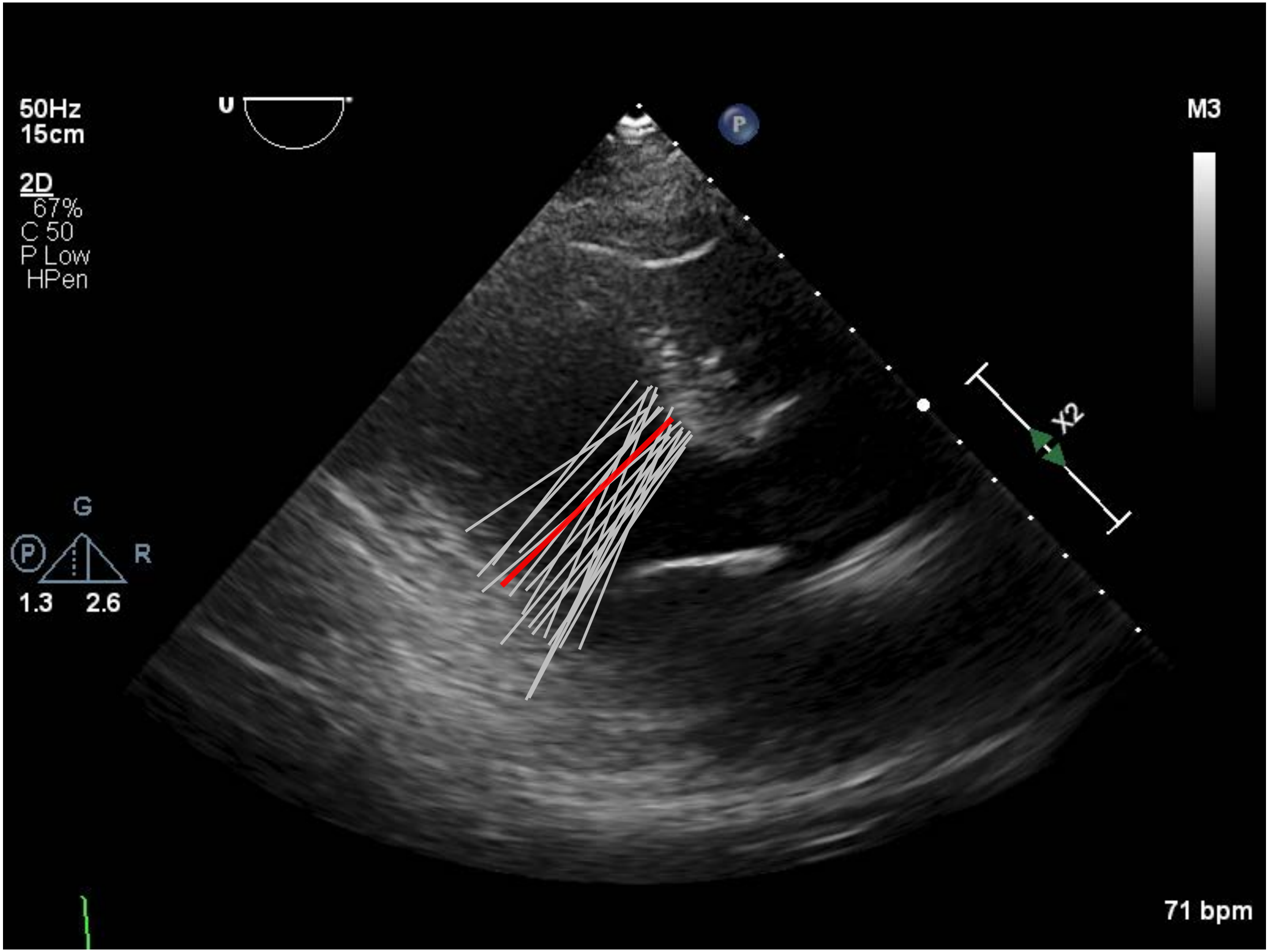

Pt 97 Diastole

Absolute difference between AI and expert consensus: 1.8 mm

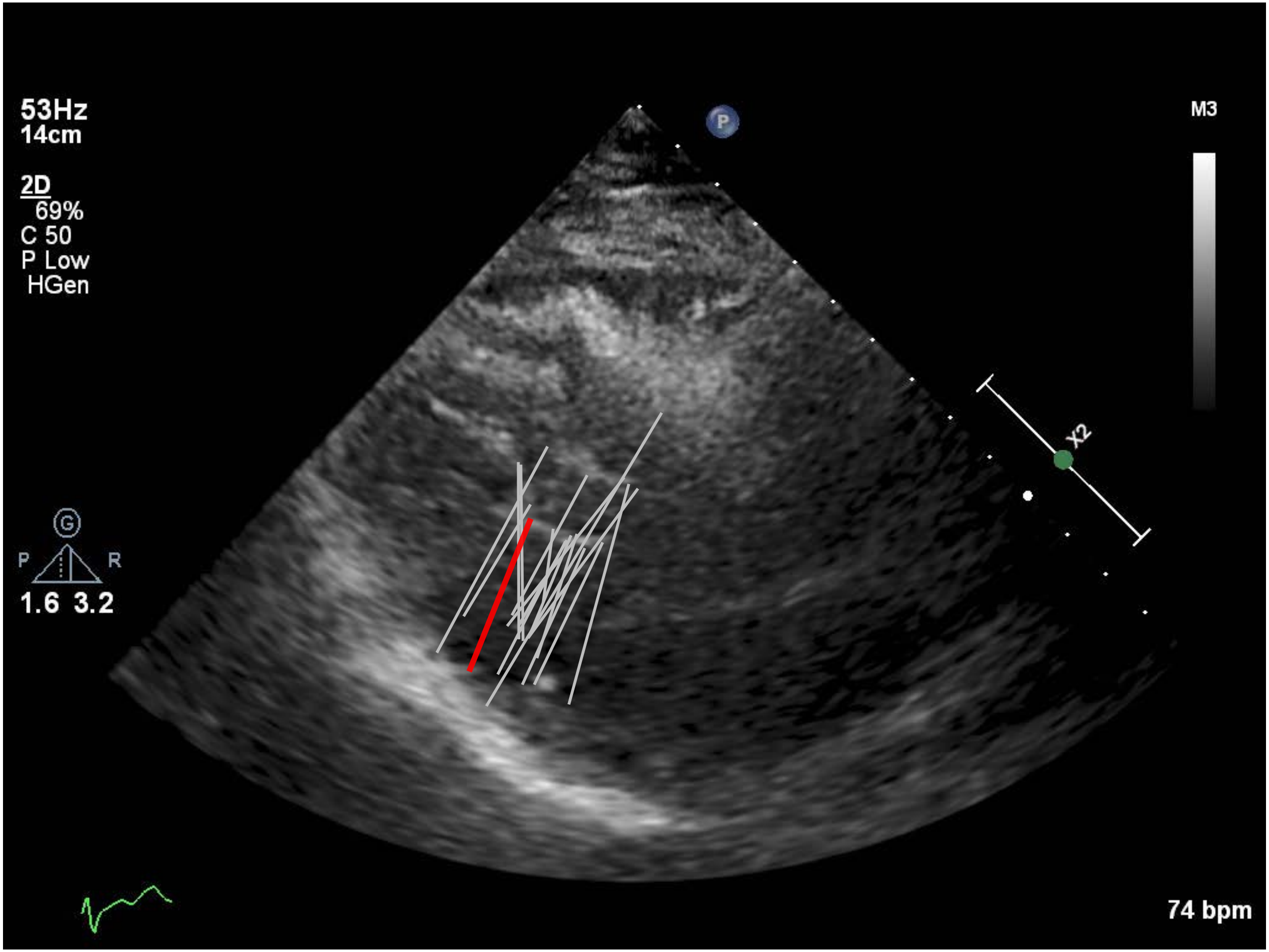

Pt 42 Systole
Absolute difference between AI and expert consensus: 1.9 mm

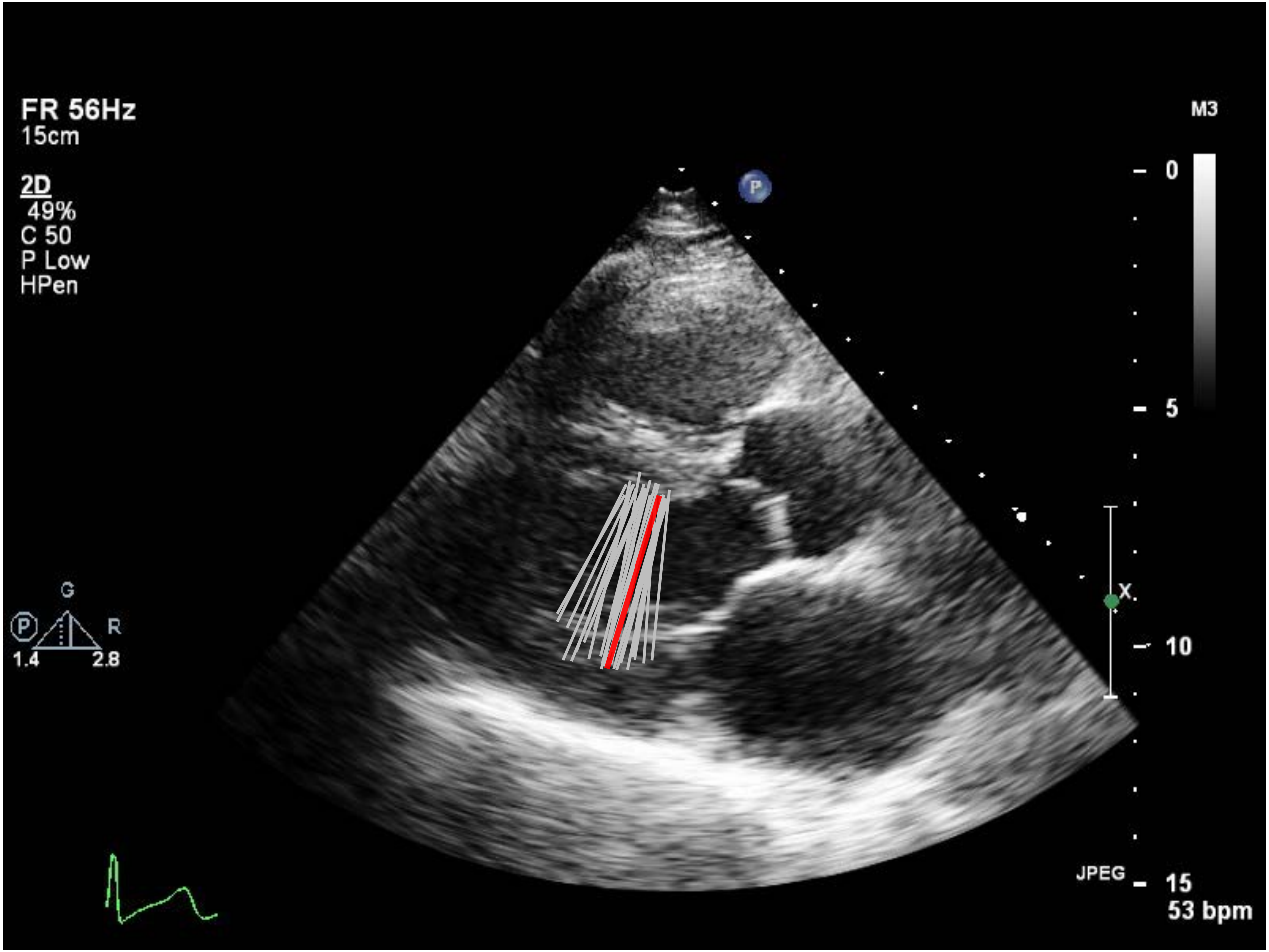

Pt 29 Systole

Absolute difference between AI and expert consensus: 1.9 mm

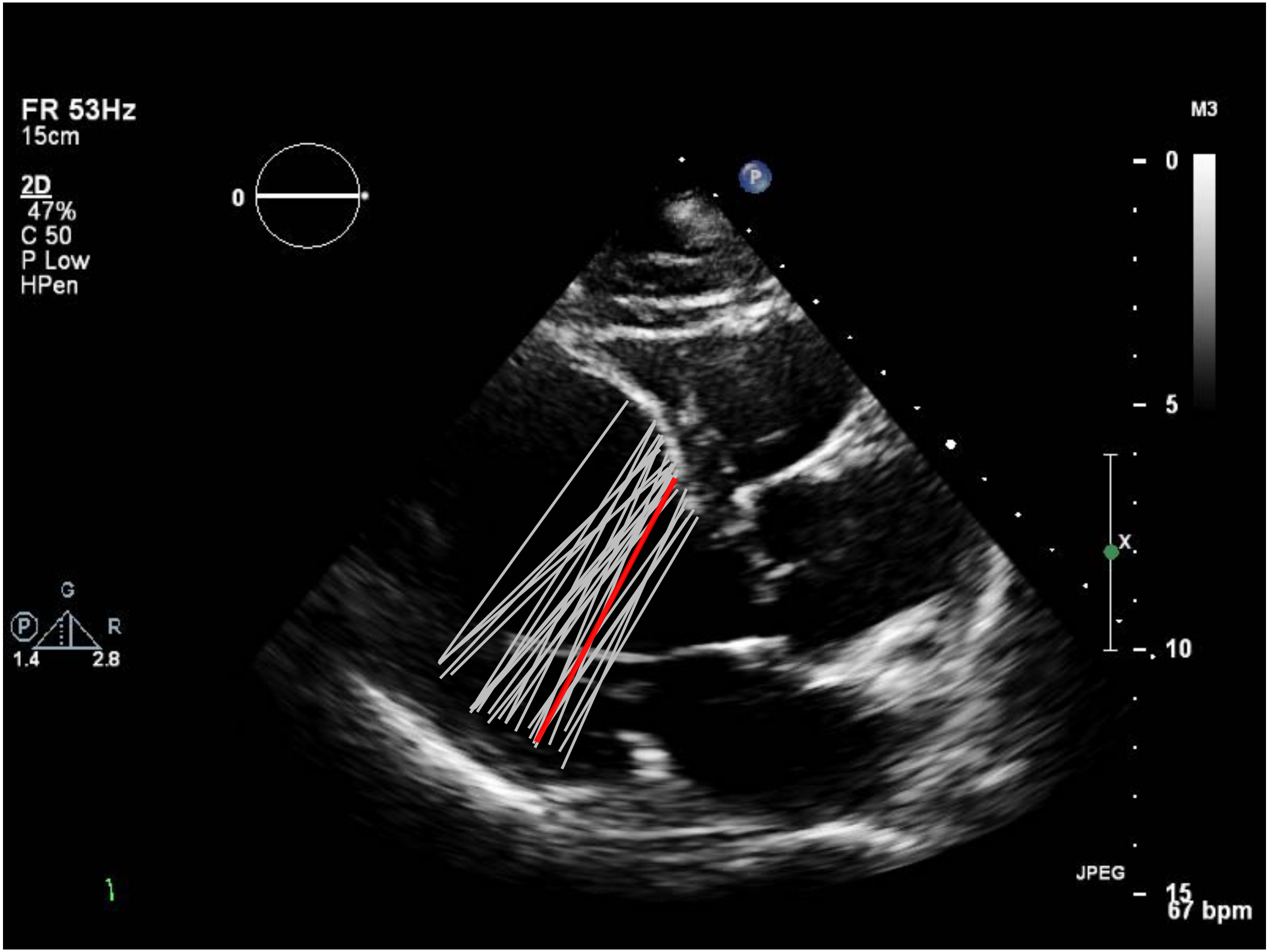

Pt 43 Diastole

Absolute difference between AI and expert consensus: 1.9 mm

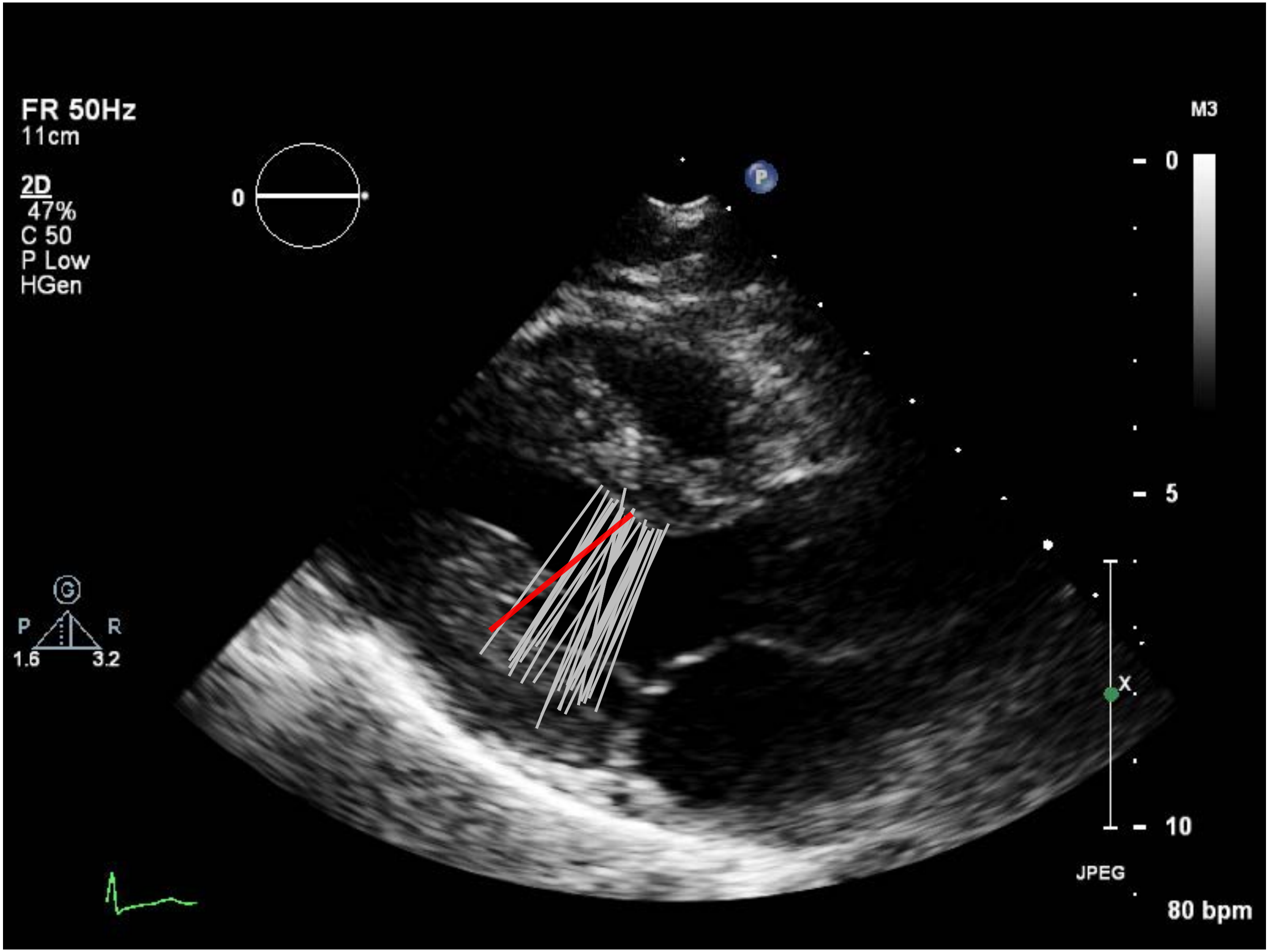

Pt 17 Systole

Absolute difference between AI and expert consensus: 1.9 mm

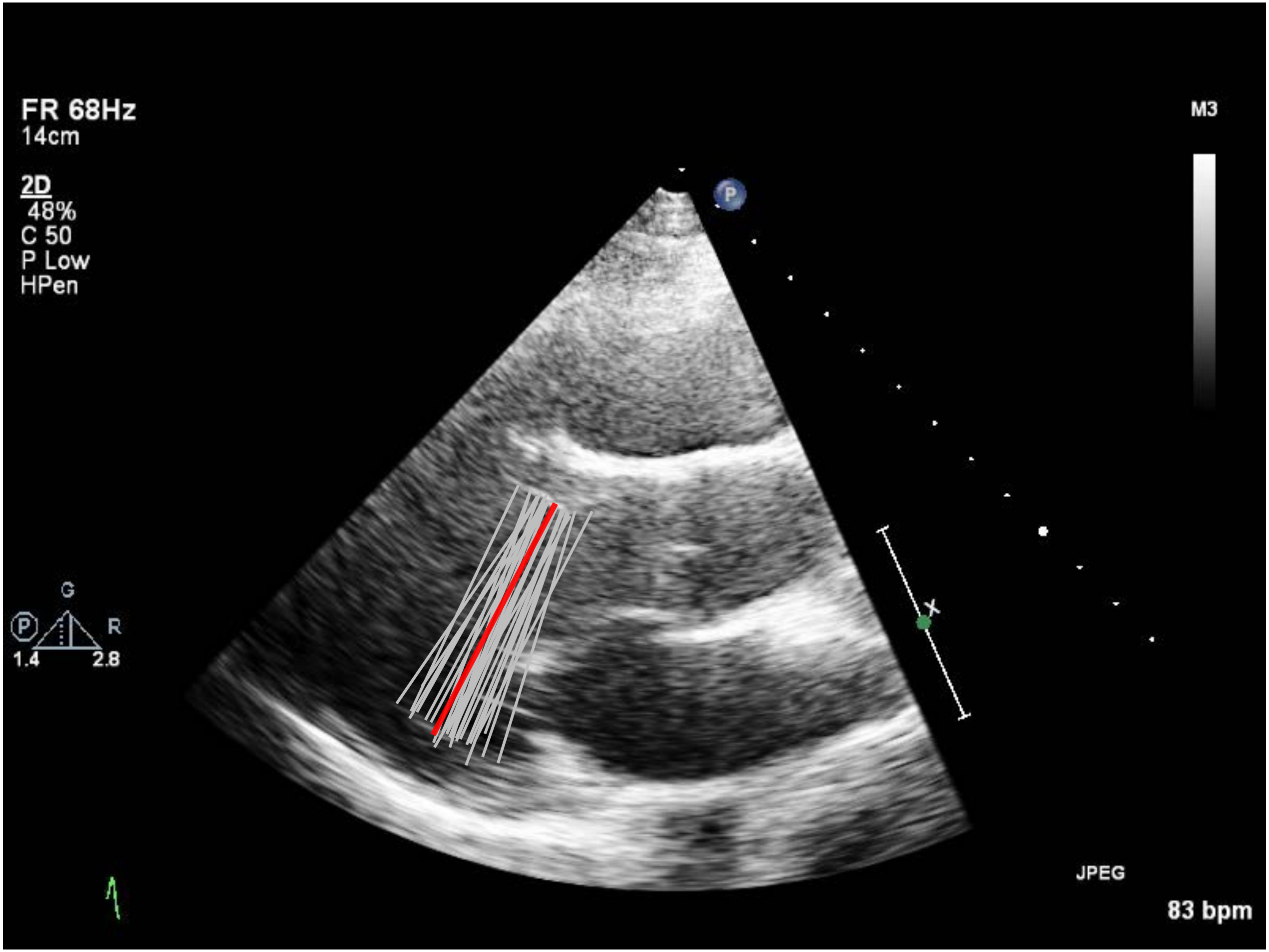

Pt 86 Diastole

Absolute difference between AI and expert consensus: 1.9 mm

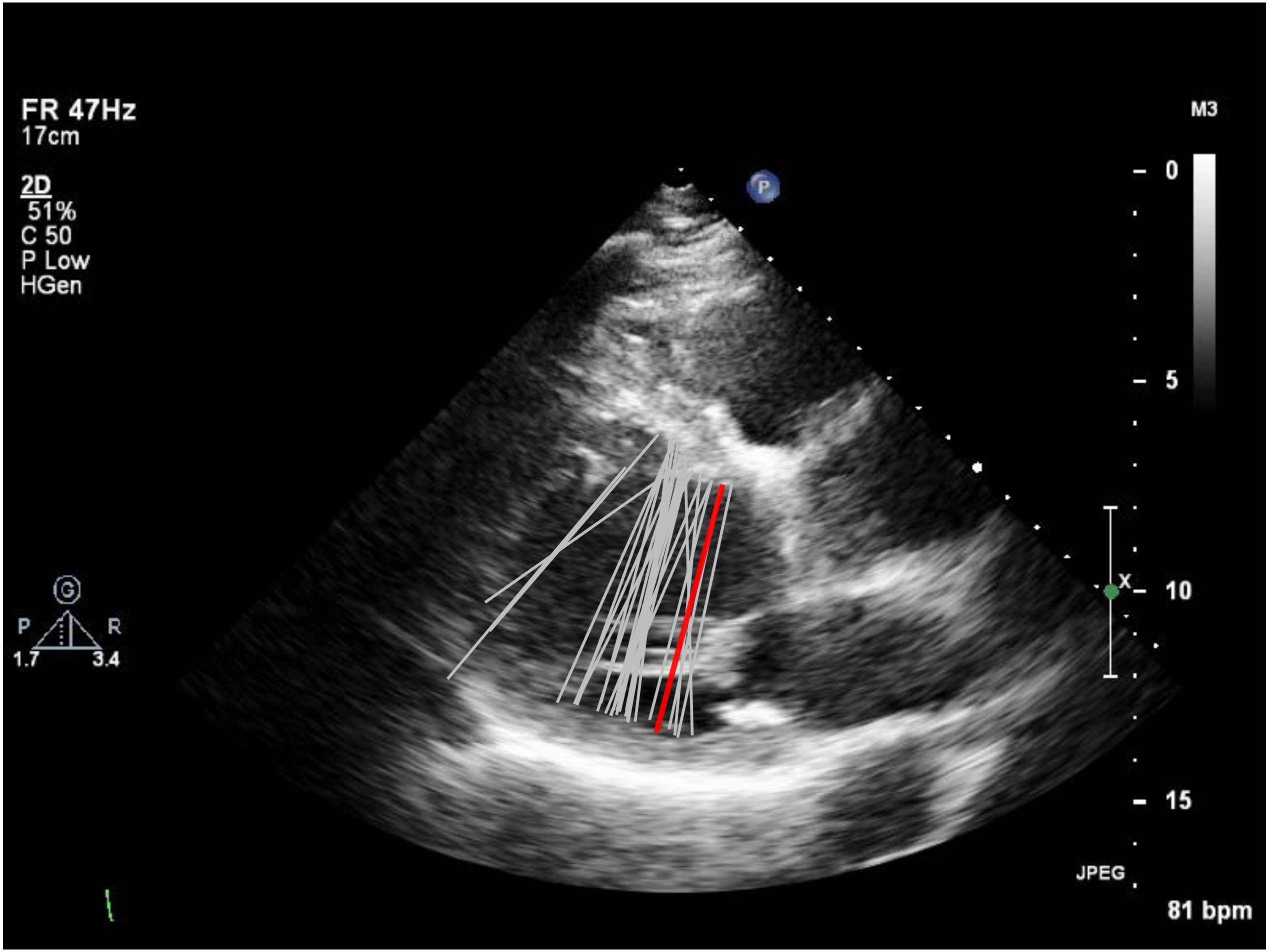

Pt 32 Diastole

Absolute difference between AI and expert consensus: 1.9 mm

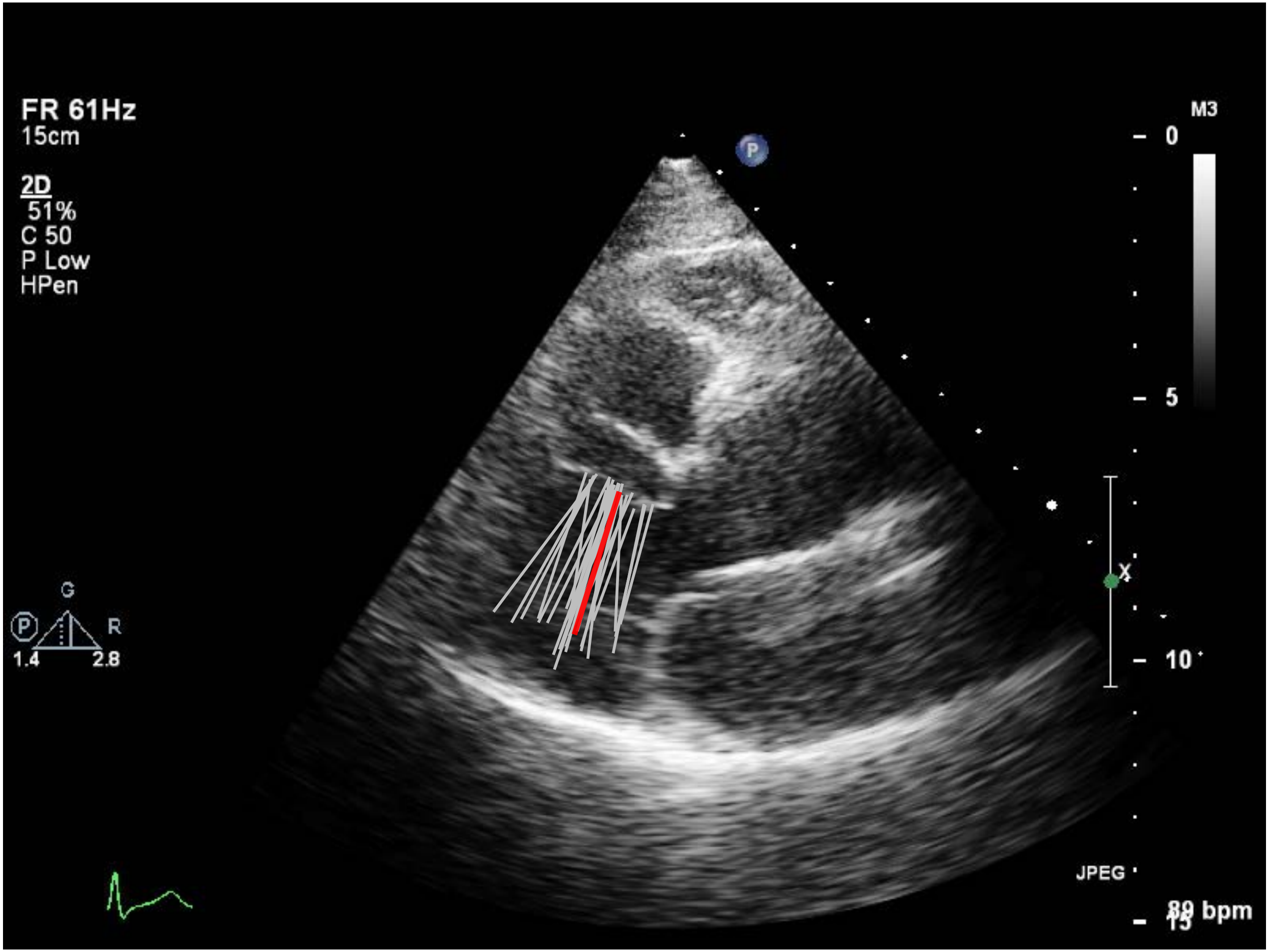

Pt 58 Systole

Absolute difference between AI and expert consensus: 1.9 mm

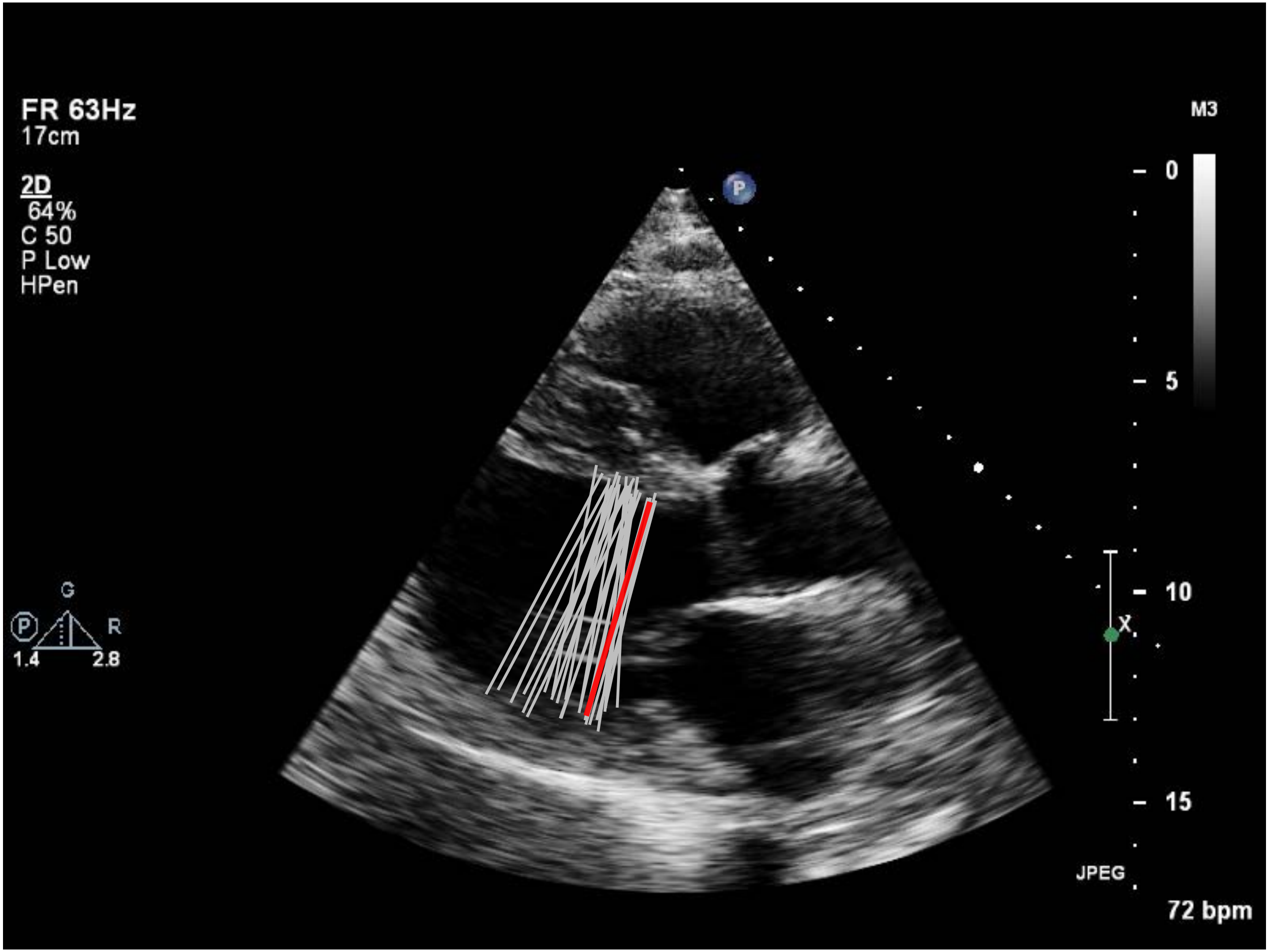

Pt 64 Diastole

Absolute difference between AI and expert consensus: 1.9 mm

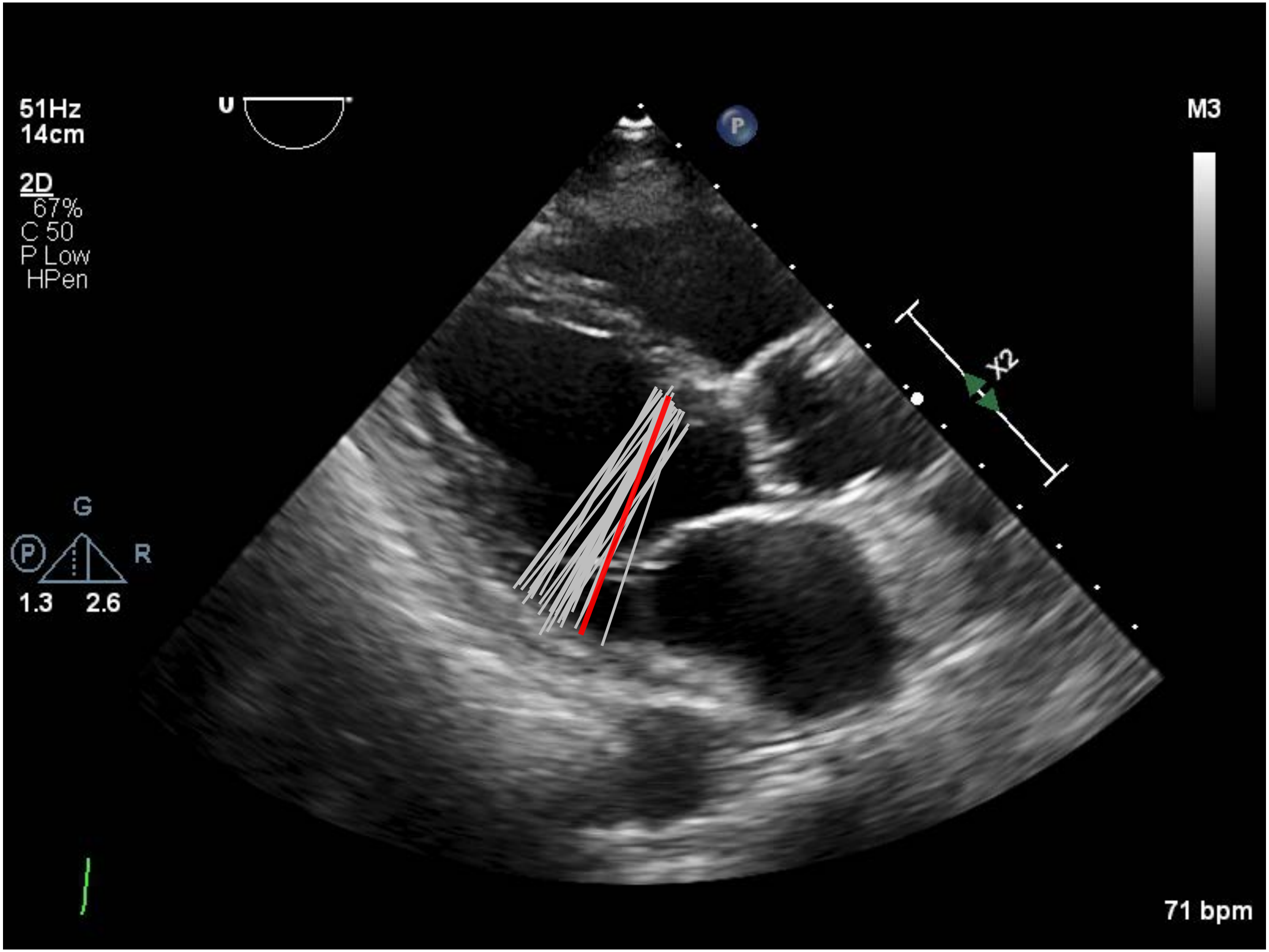

Pt 88 Diastole

Absolute difference between AI and expert consensus: 2 mm

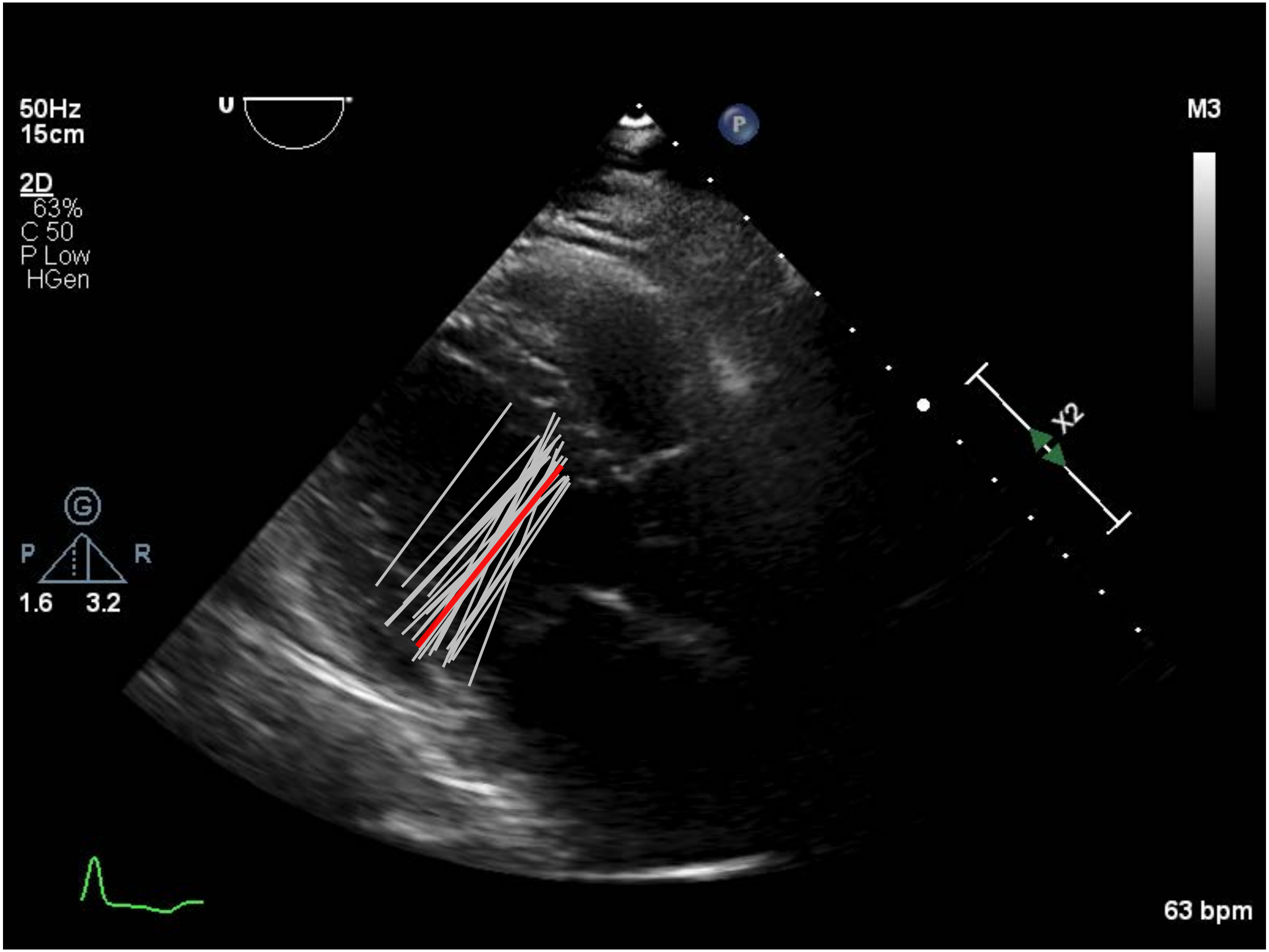

Pt 95 Systole

Absolute difference between AI and expert consensus: 2 mm

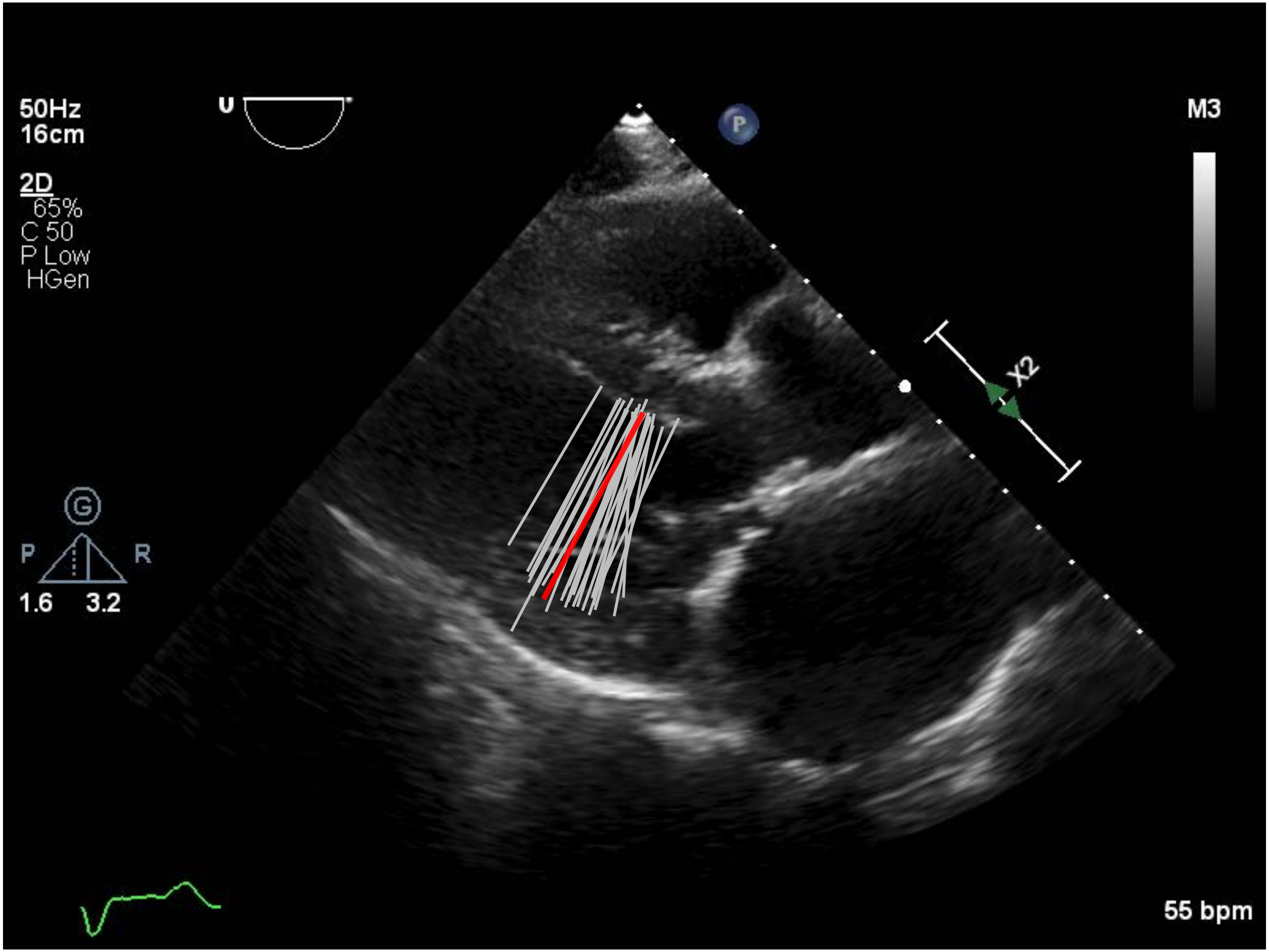

Pt 78 Systole

Absolute difference between AI and expert consensus: 2 mm

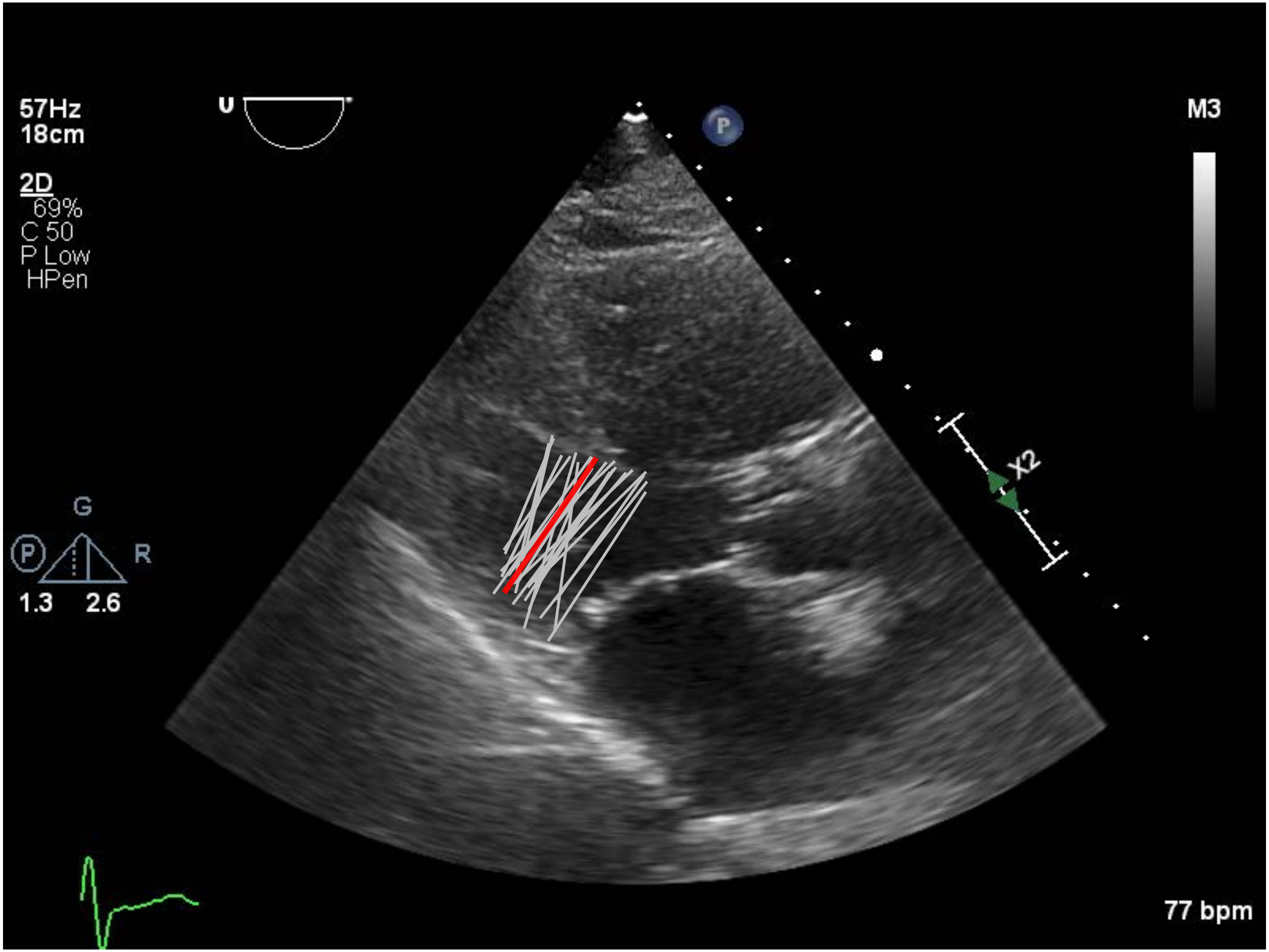

Pt 15 Systole

Absolute difference between AI and expert consensus: 2 mm

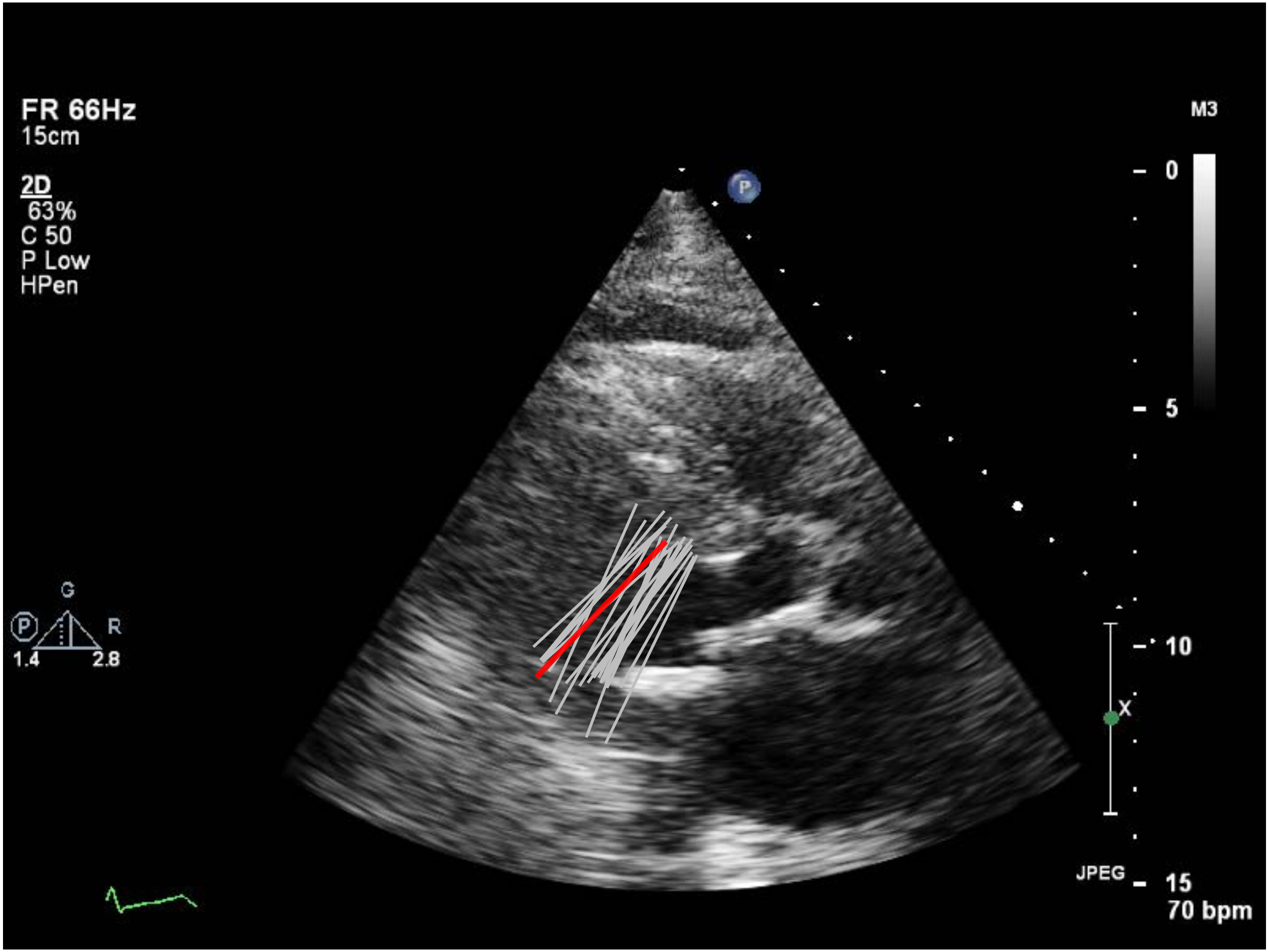

Pt 62 Systole

Absolute difference between AI and expert consensus: 2.1 mm

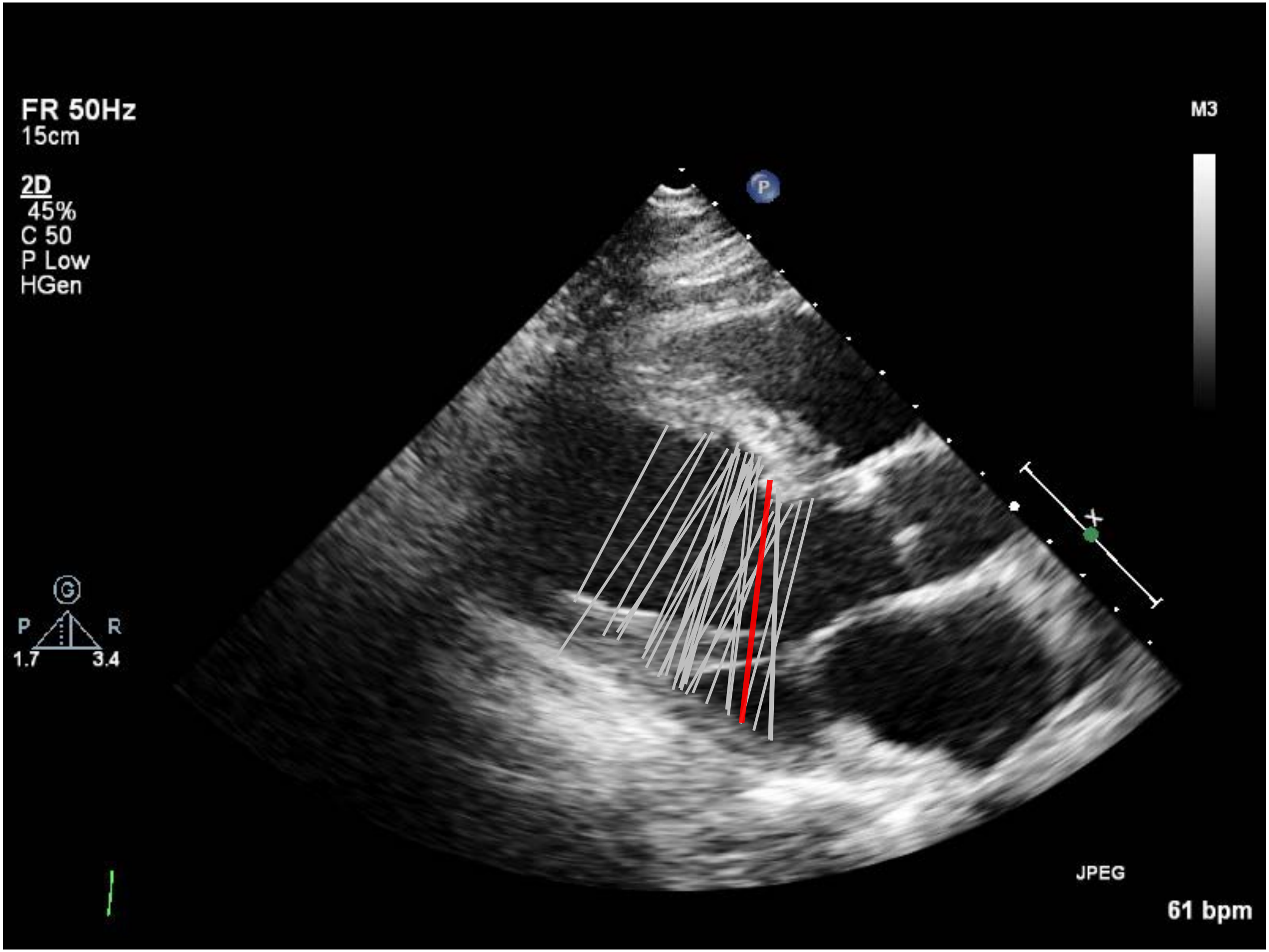

Pt 85 Diastole

Absolute difference between AI and expert consensus: 2.1 mm

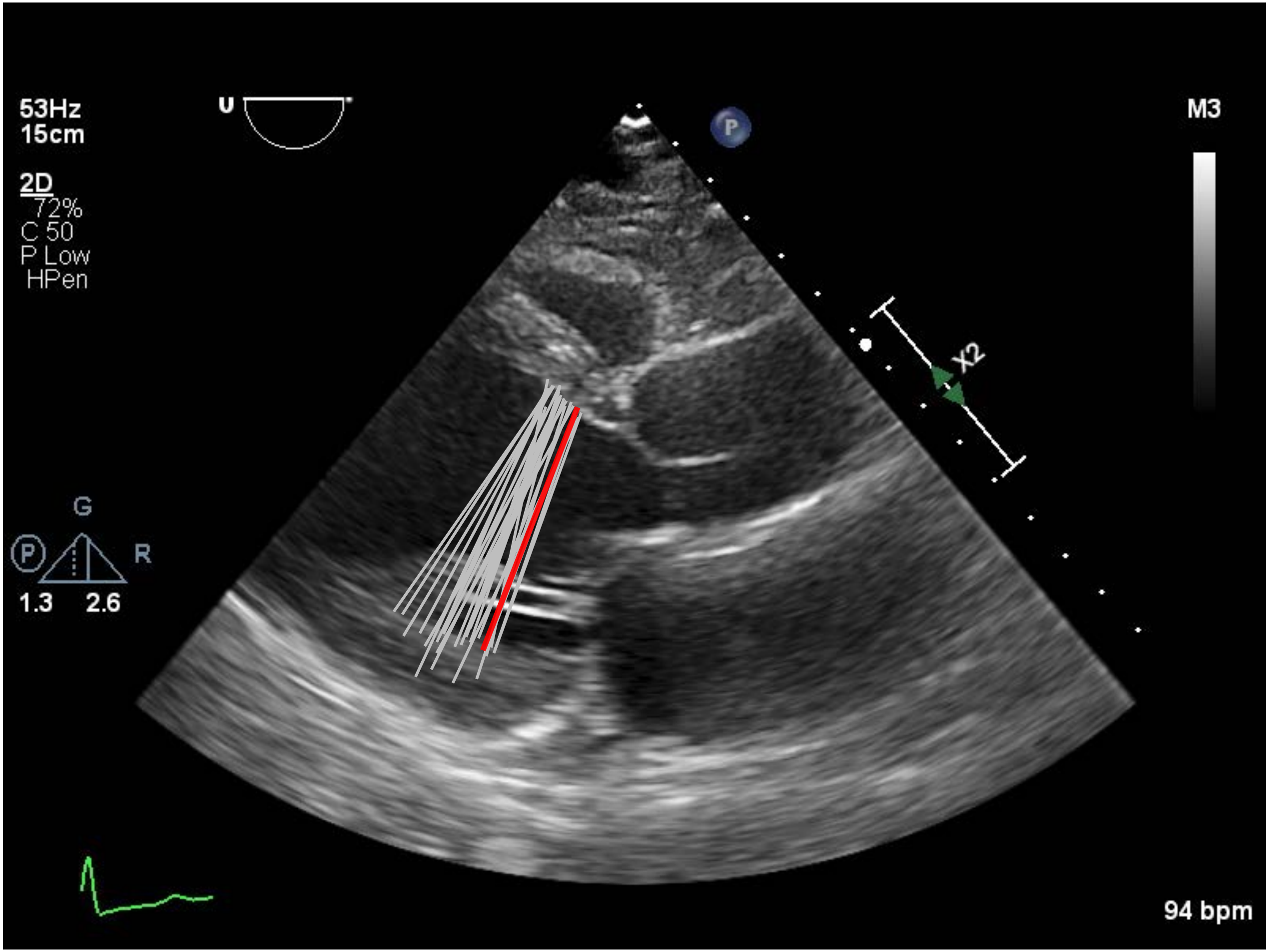

Pt 61 Systole

Absolute difference between AI and expert consensus: 2.2 mm

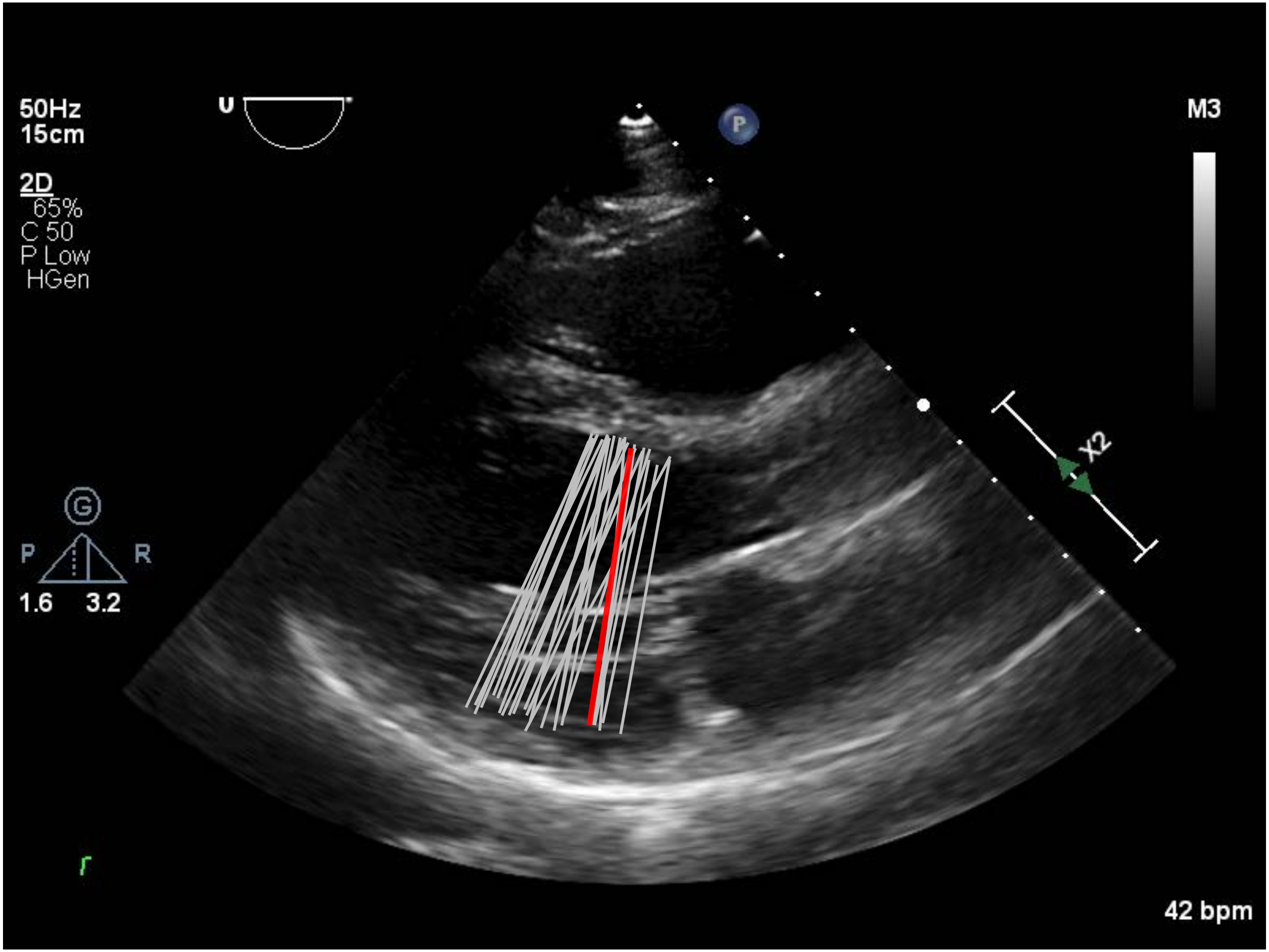

Pt 22 Diastole

Absolute difference between AI and expert consensus: 2.2 mm

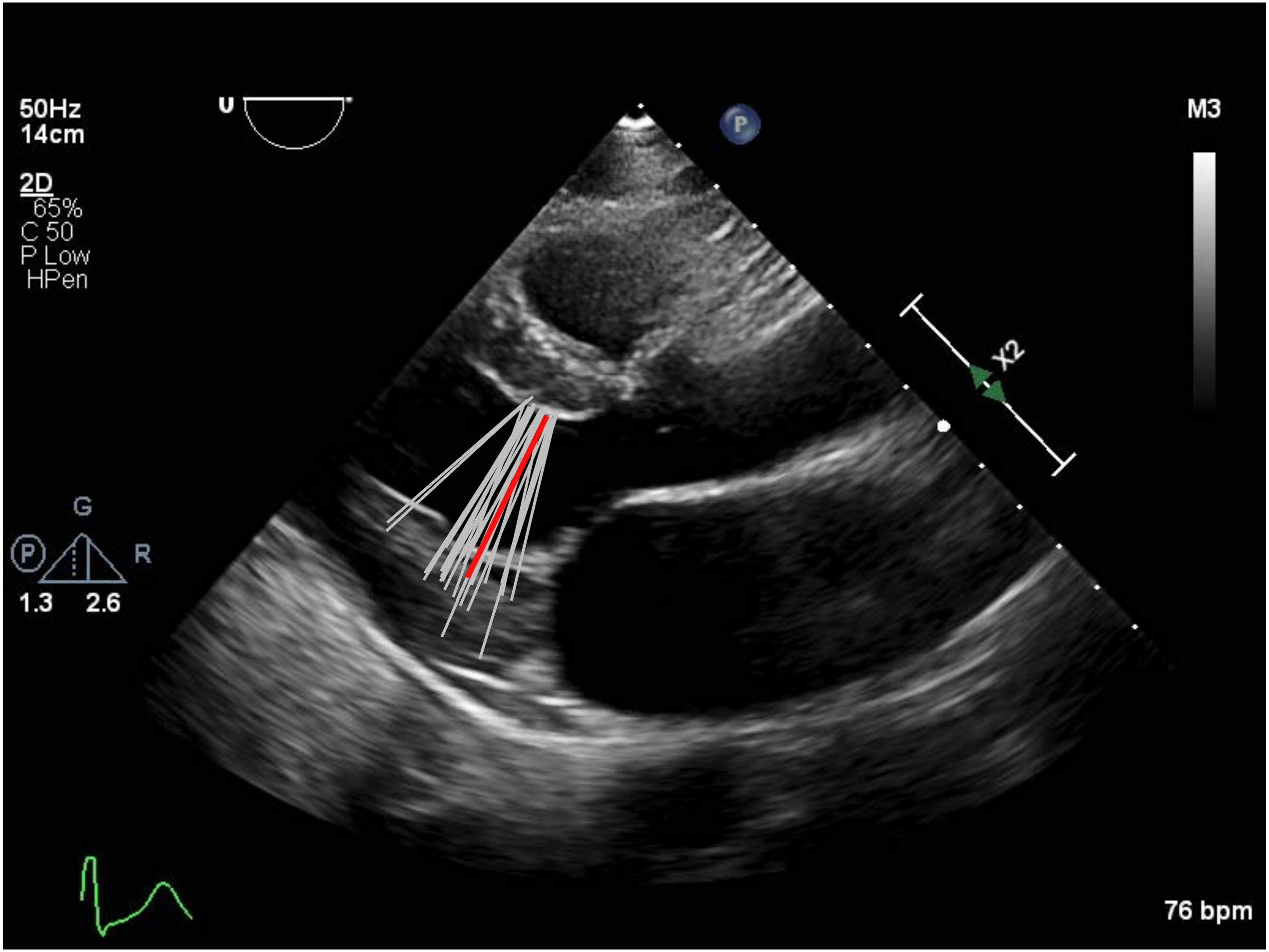

Pt 25 Systole

Absolute difference between AI and expert consensus: 2.2 mm

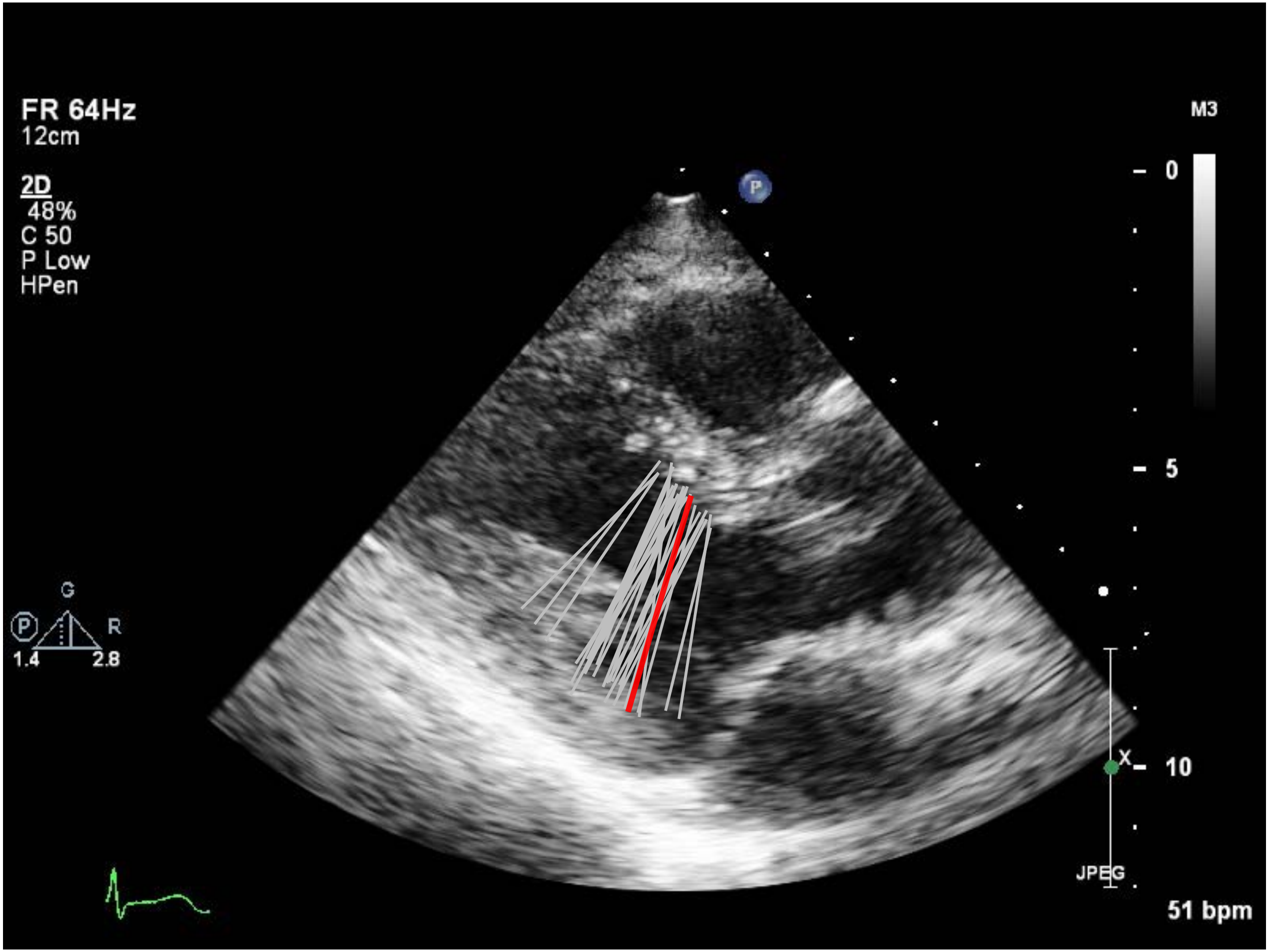

Pt 36 Systole

Absolute difference between AI and expert consensus: 2.3 mm

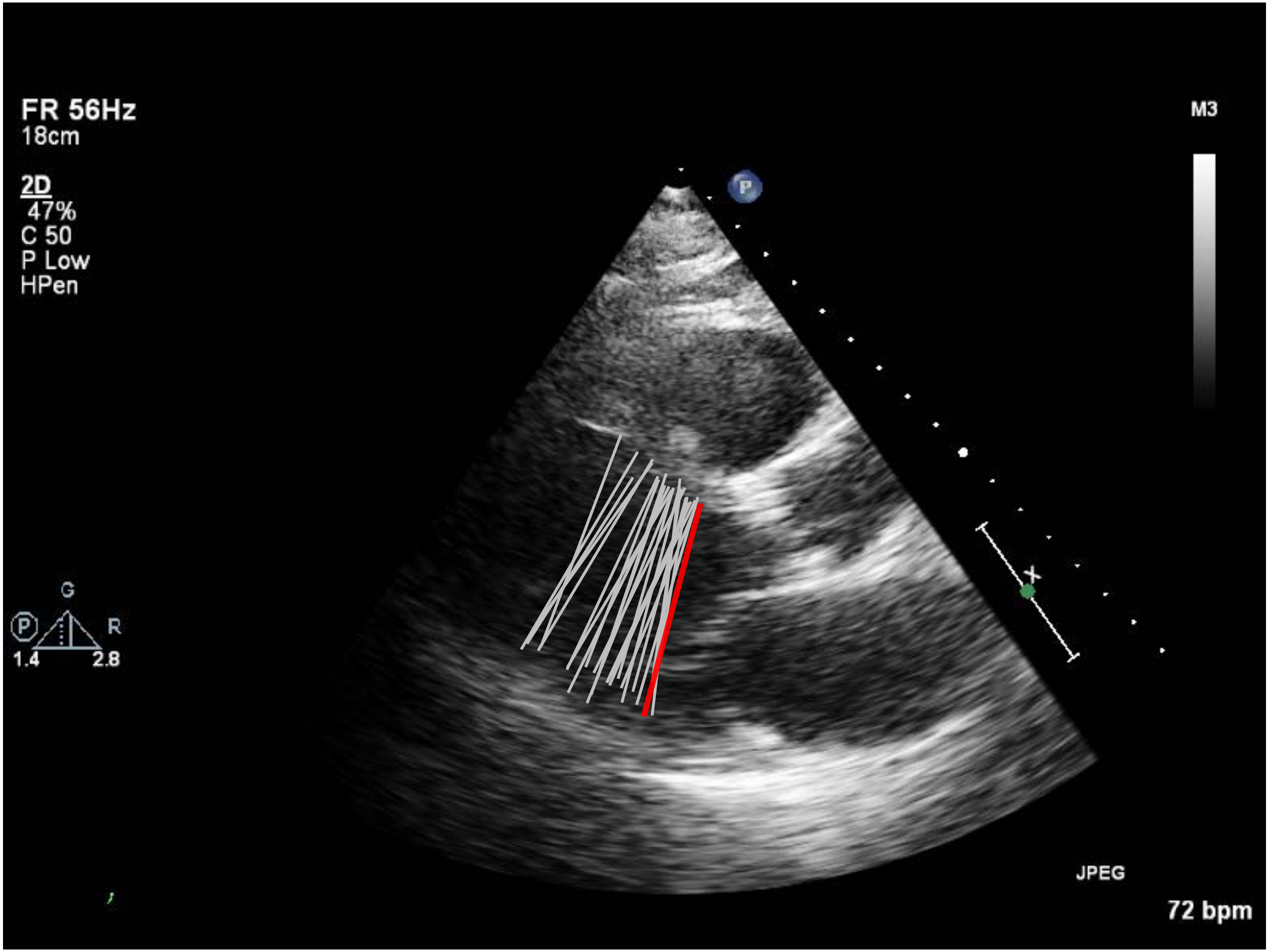

Pt 19 Diastole

Absolute difference between AI and expert consensus: 2.4 mm

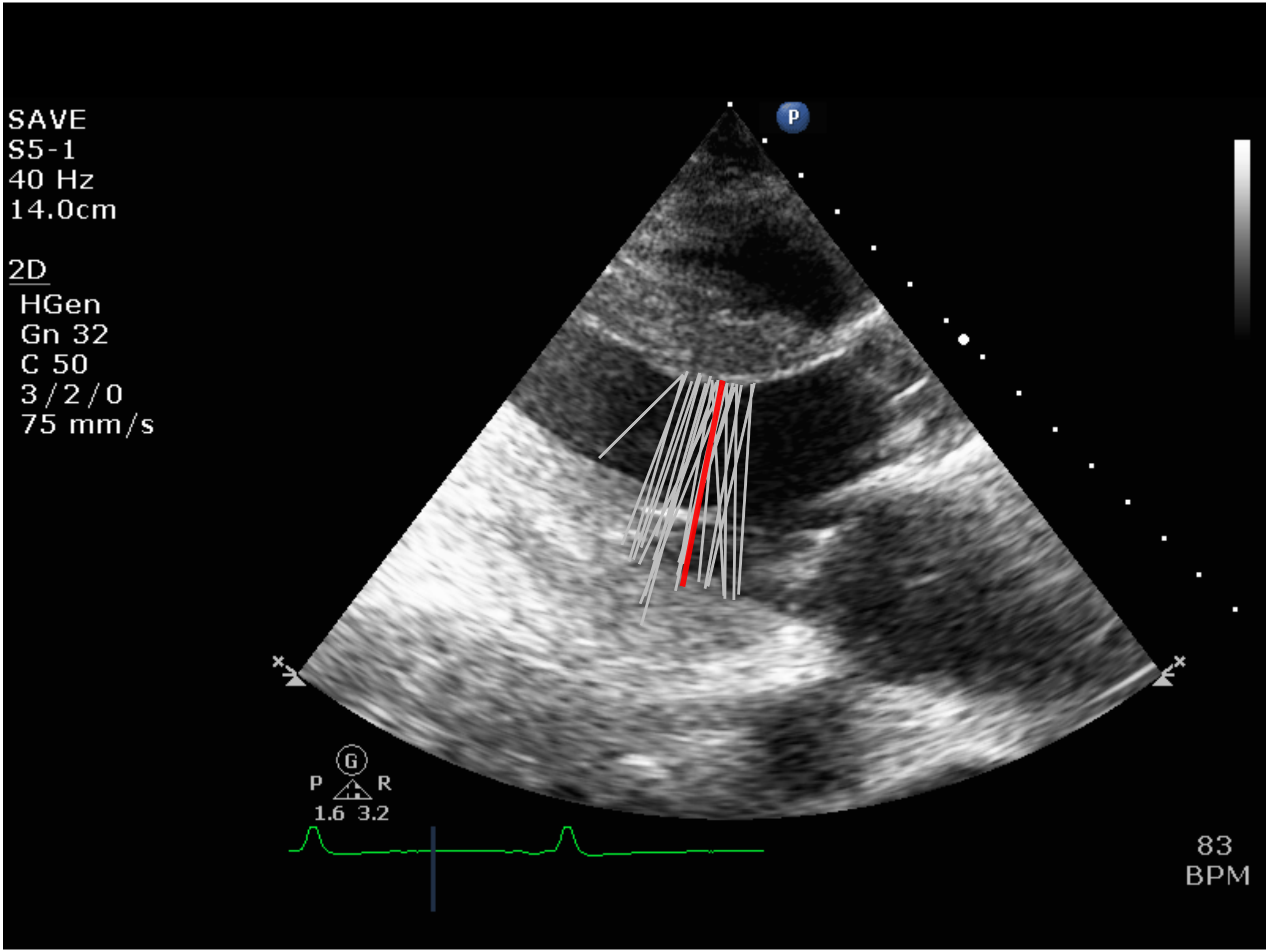

Pt 84 Systole

Absolute difference between AI and expert consensus: 2.4 mm

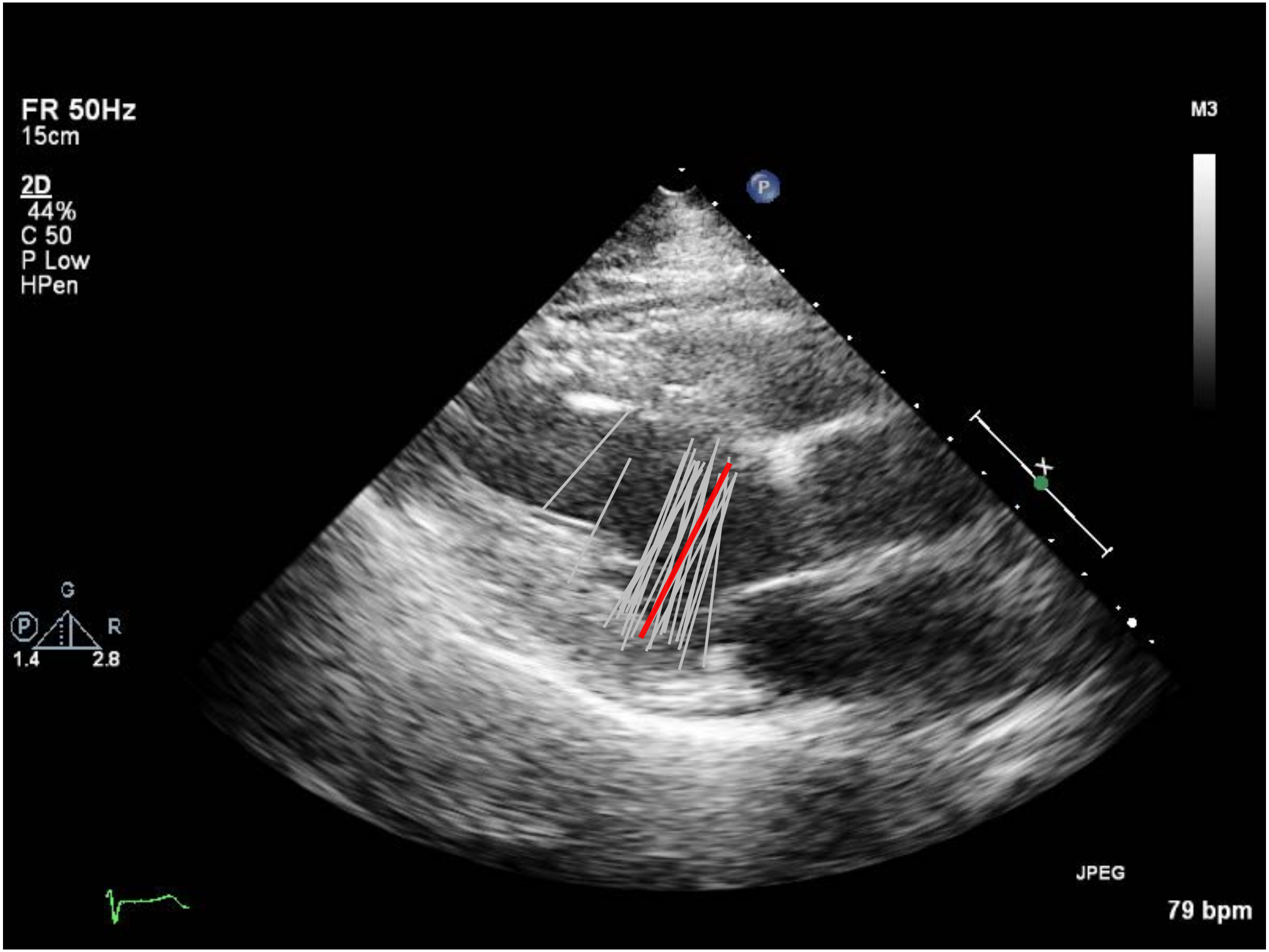

Pt 12 Systole

Absolute difference between AI and expert consensus: 2.5 mm

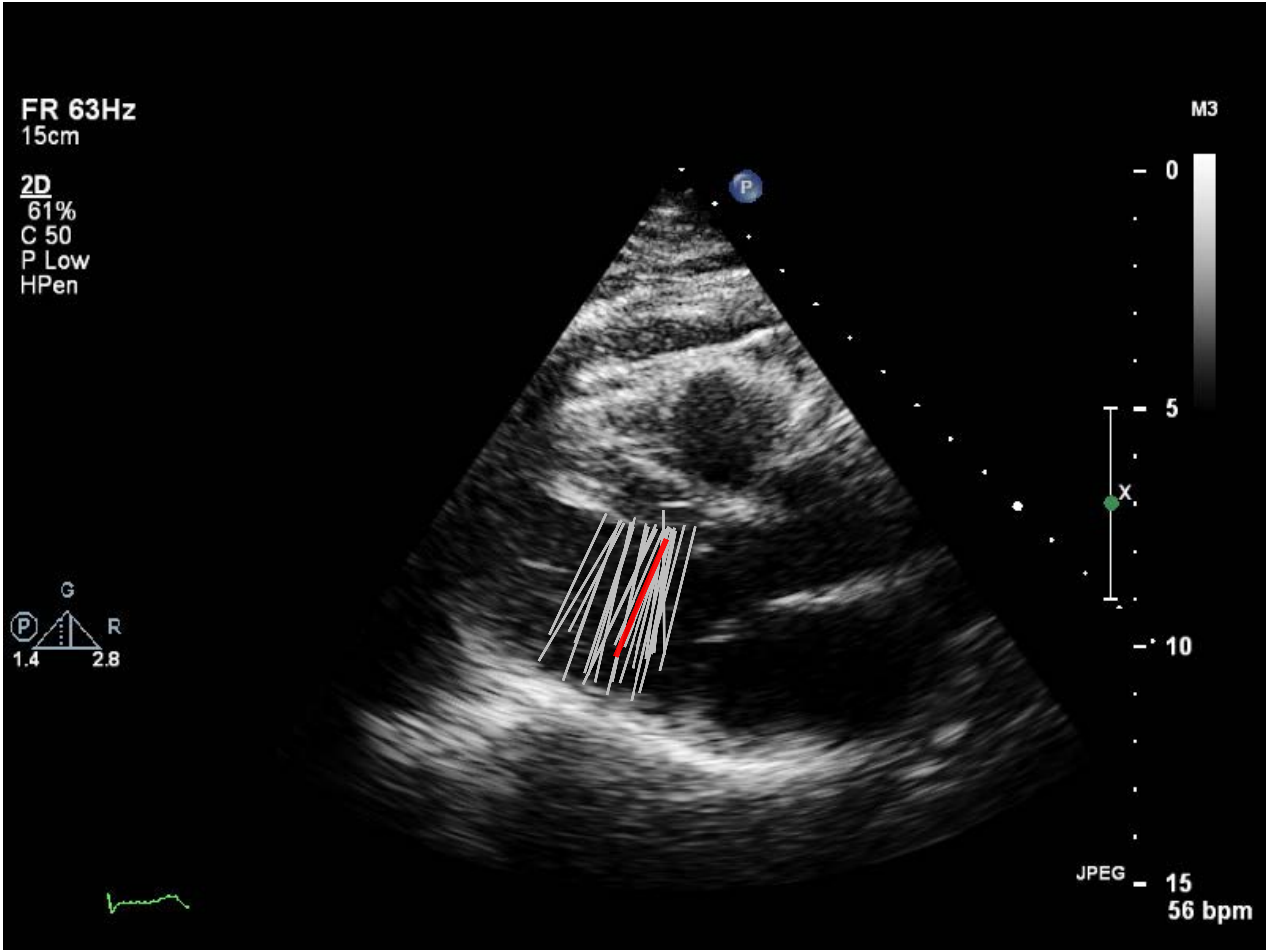

Pt 98 Systole

Absolute difference between AI and expert consensus: 2.5 mm

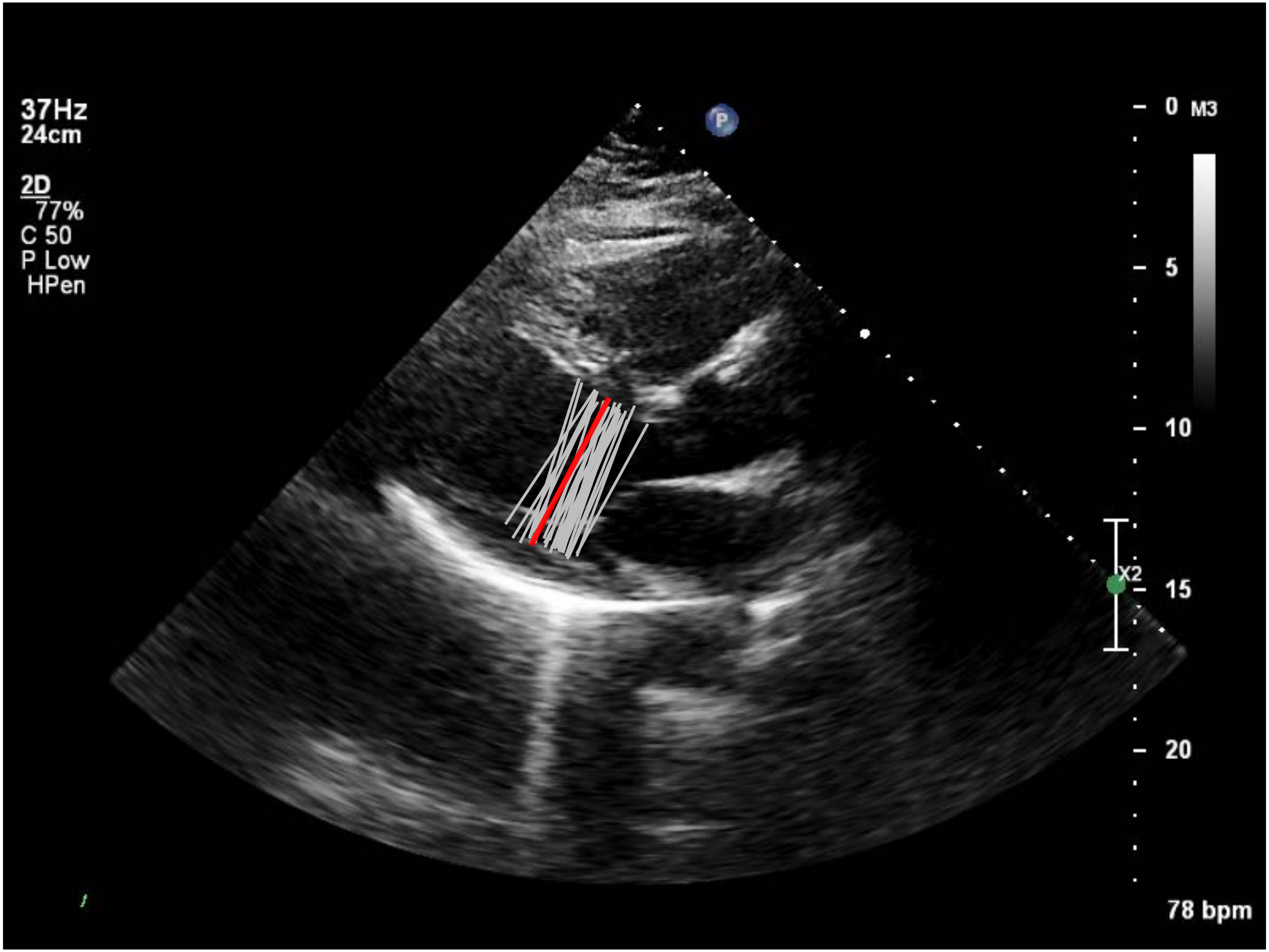

Pt 23 Diastole

Absolute difference between AI and expert consensus: 2.6 mm

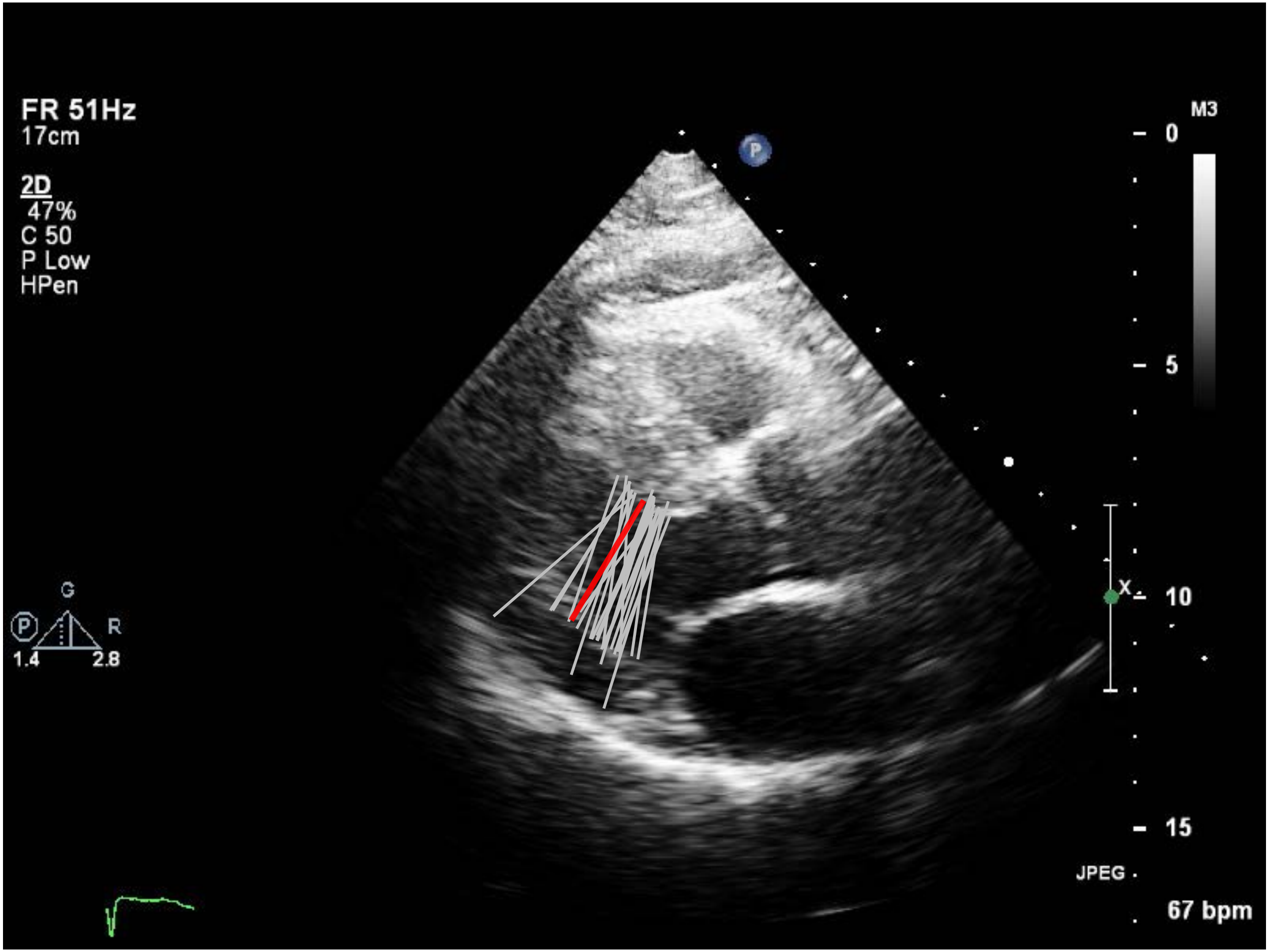

Pt 5 Systole

Absolute difference between AI and expert consensus: 2.6 mm

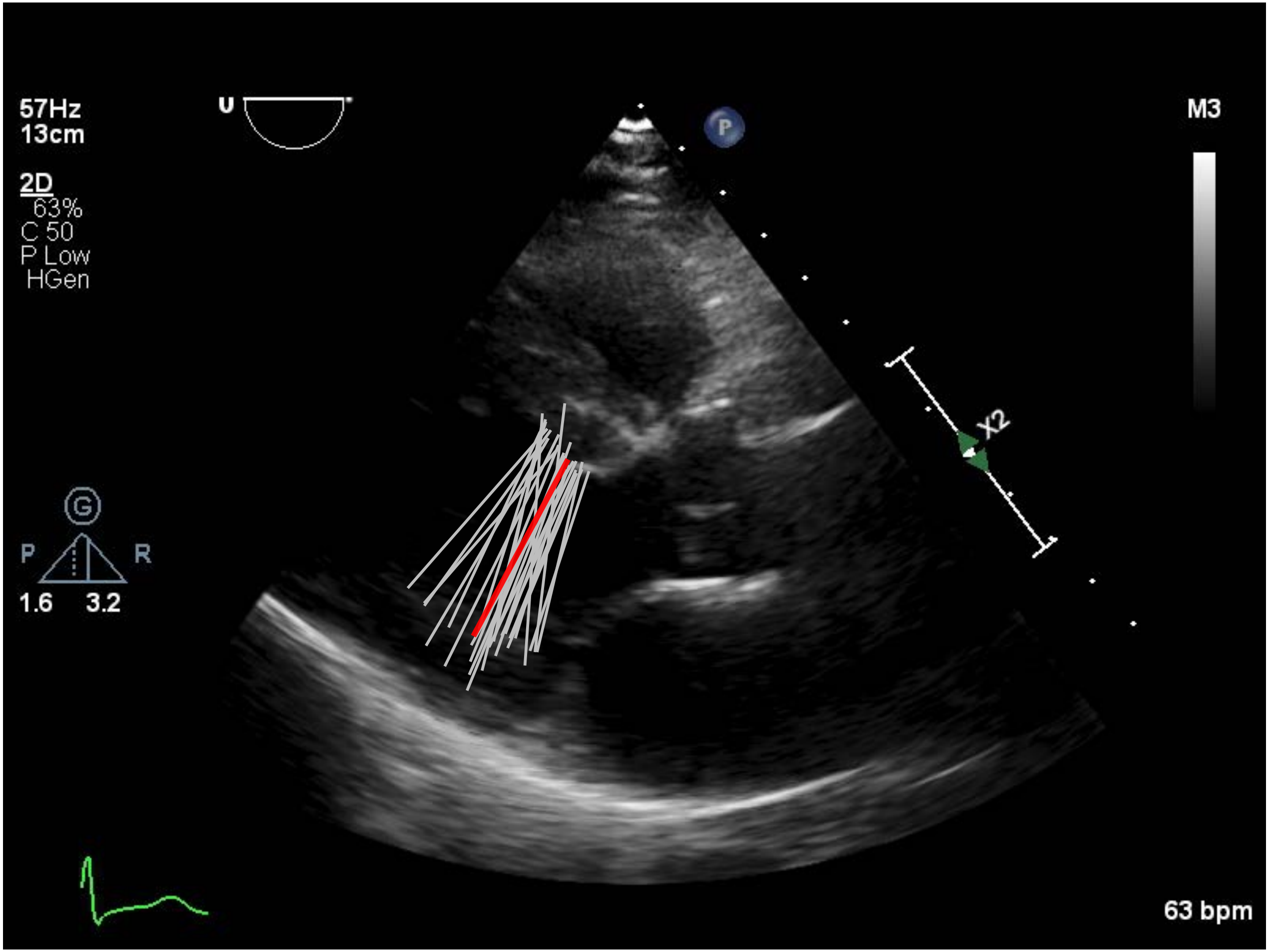

Pt 53 Systole

Absolute difference between AI and expert consensus: 2.7 mm

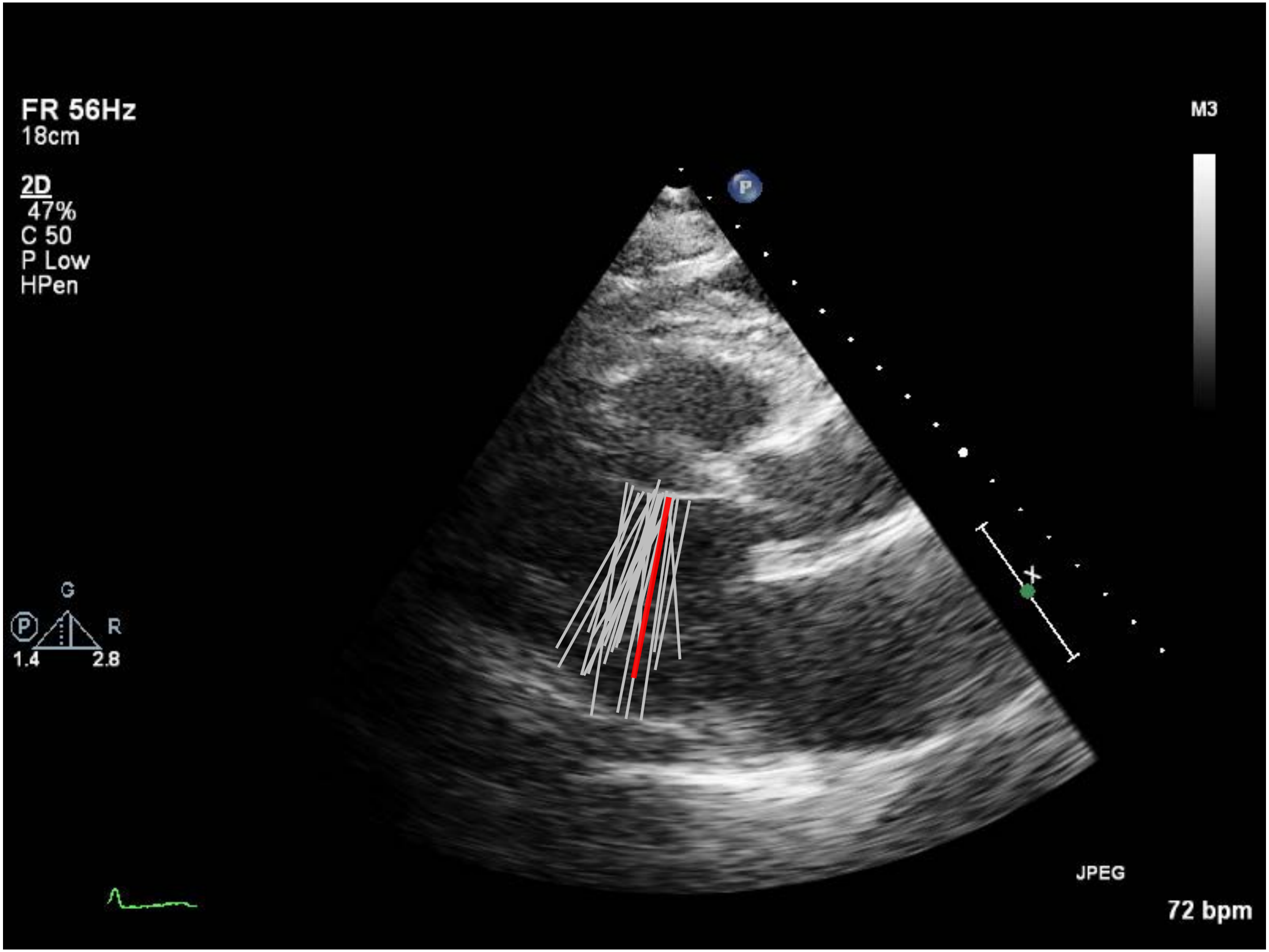

Pt 19 Systole

Absolute difference between AI and expert consensus: 2.8 mm

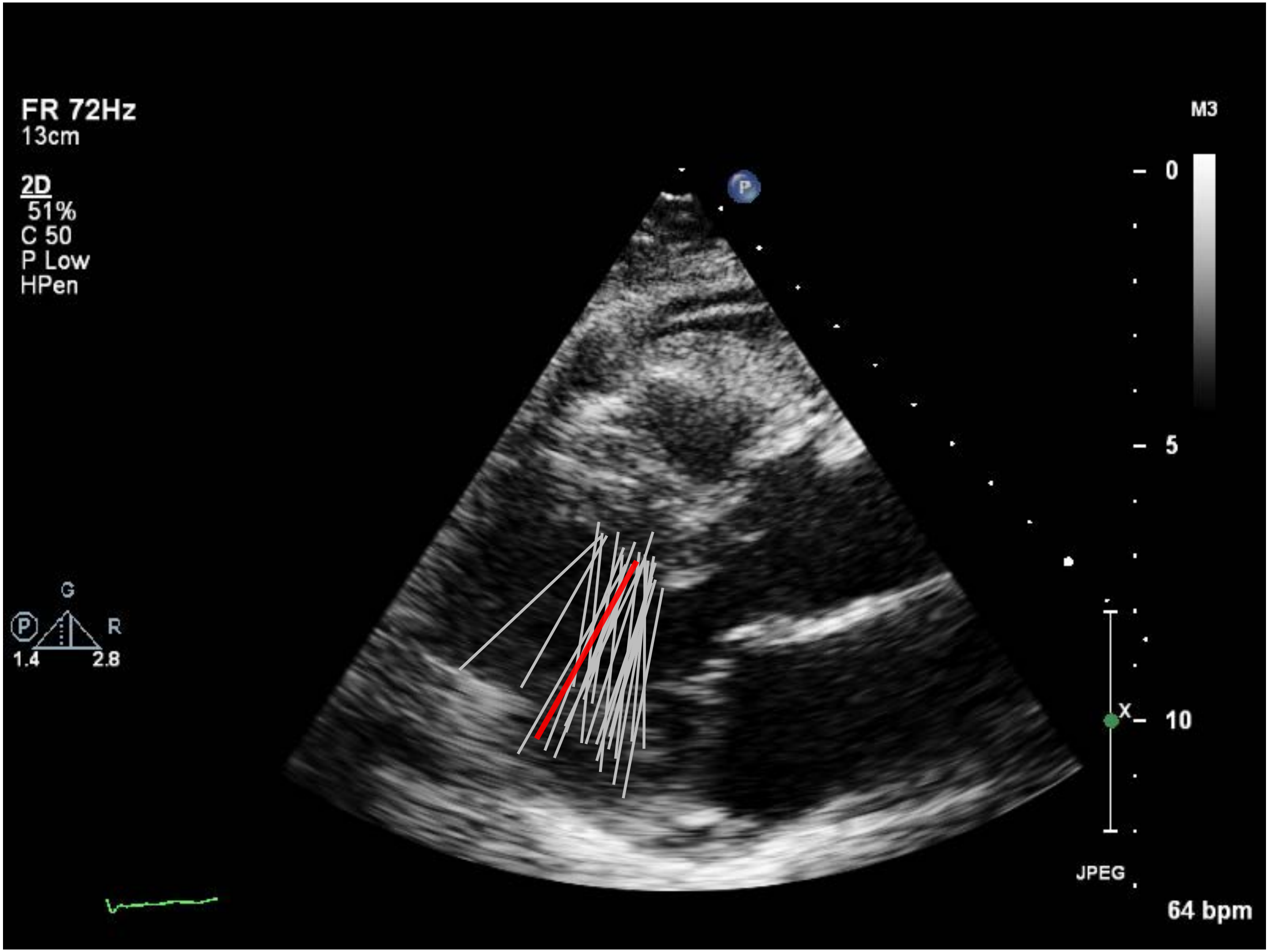

Pt 48 Systole

Absolute difference between AI and expert consensus: 2.8 mm

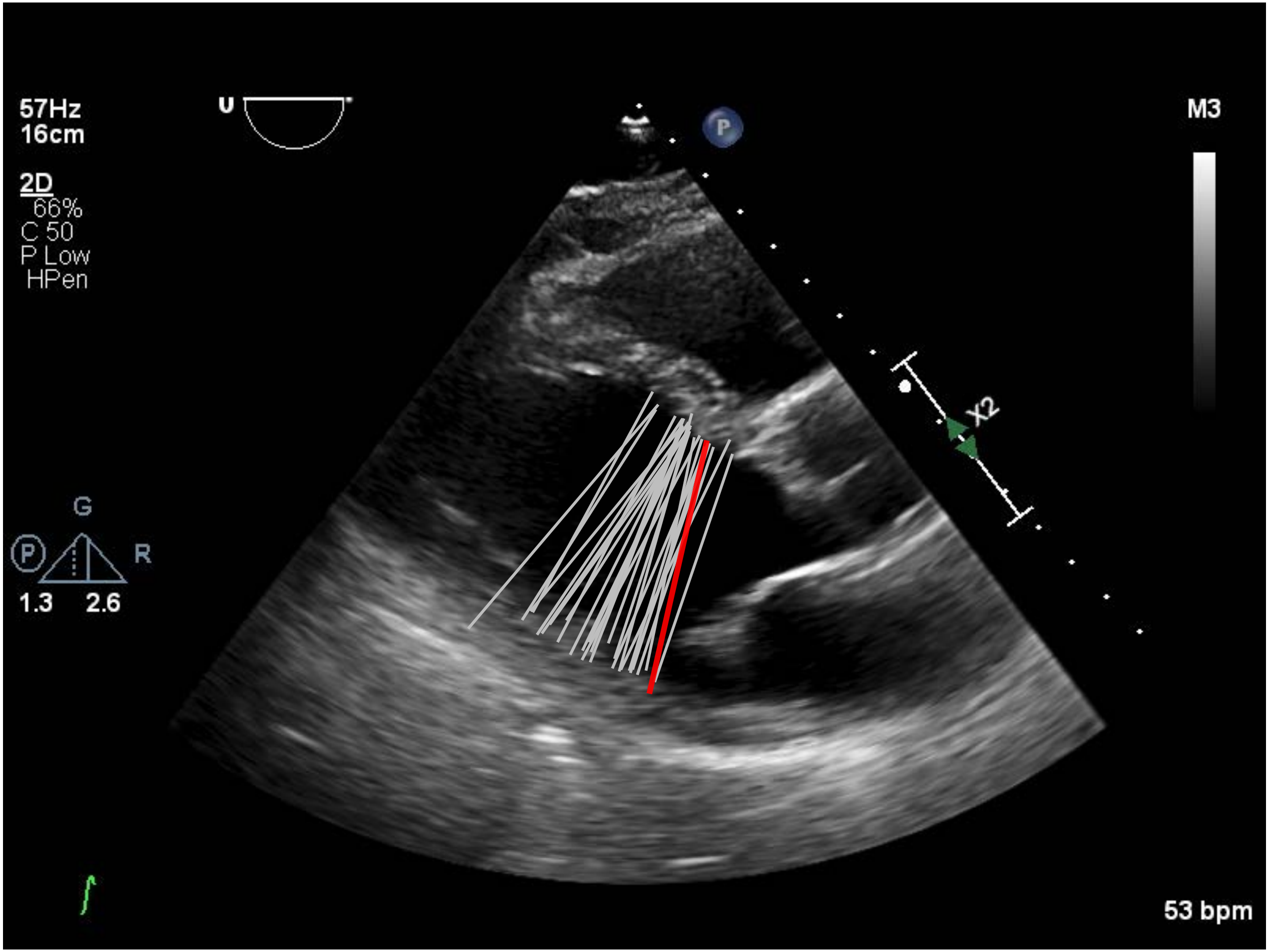

Pt 7 Diastole

Absolute difference between AI and expert consensus: 2.9 mm

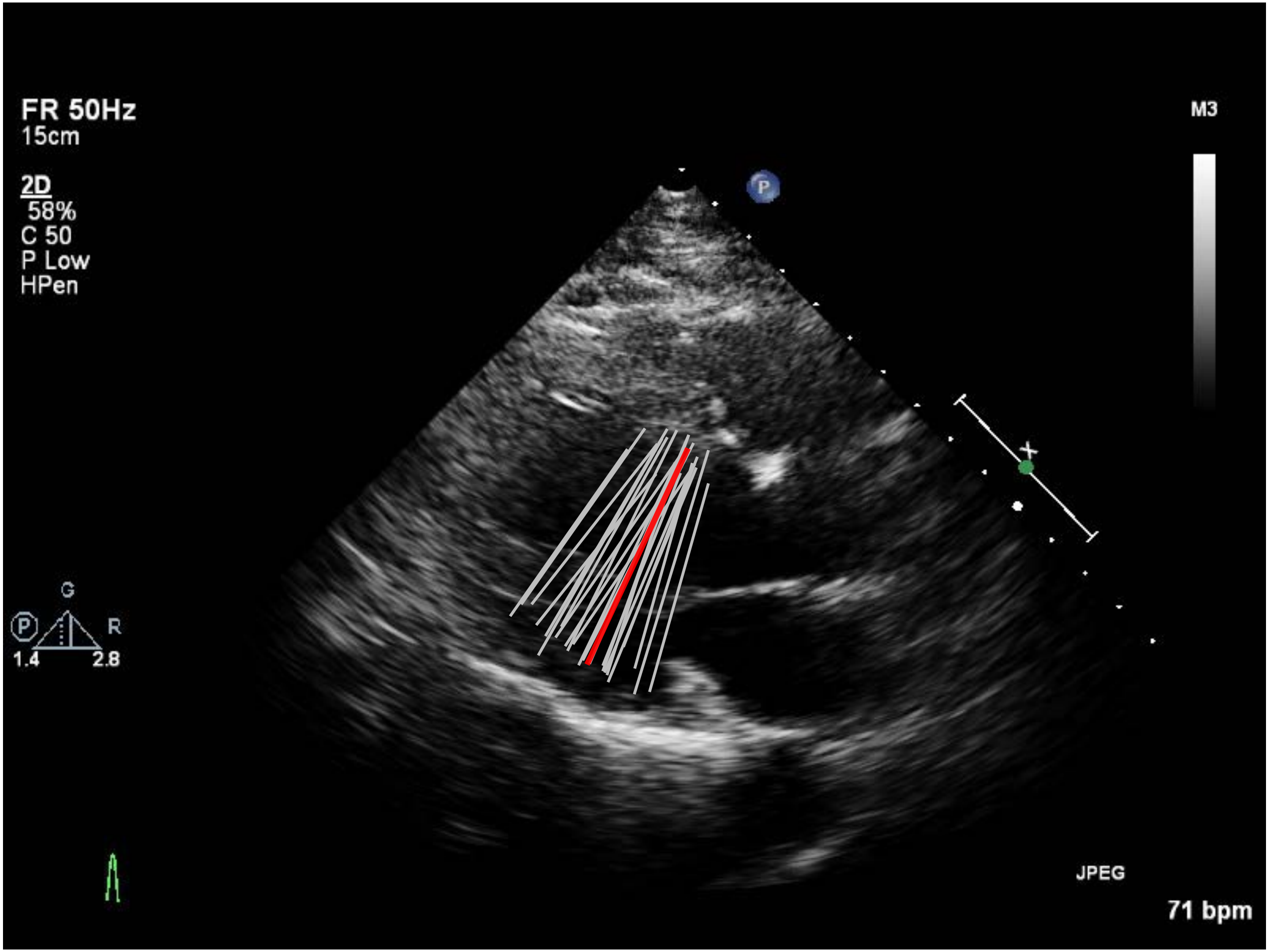

Pt 77 Diastole

Absolute difference between AI and expert consensus: 2.9 mm

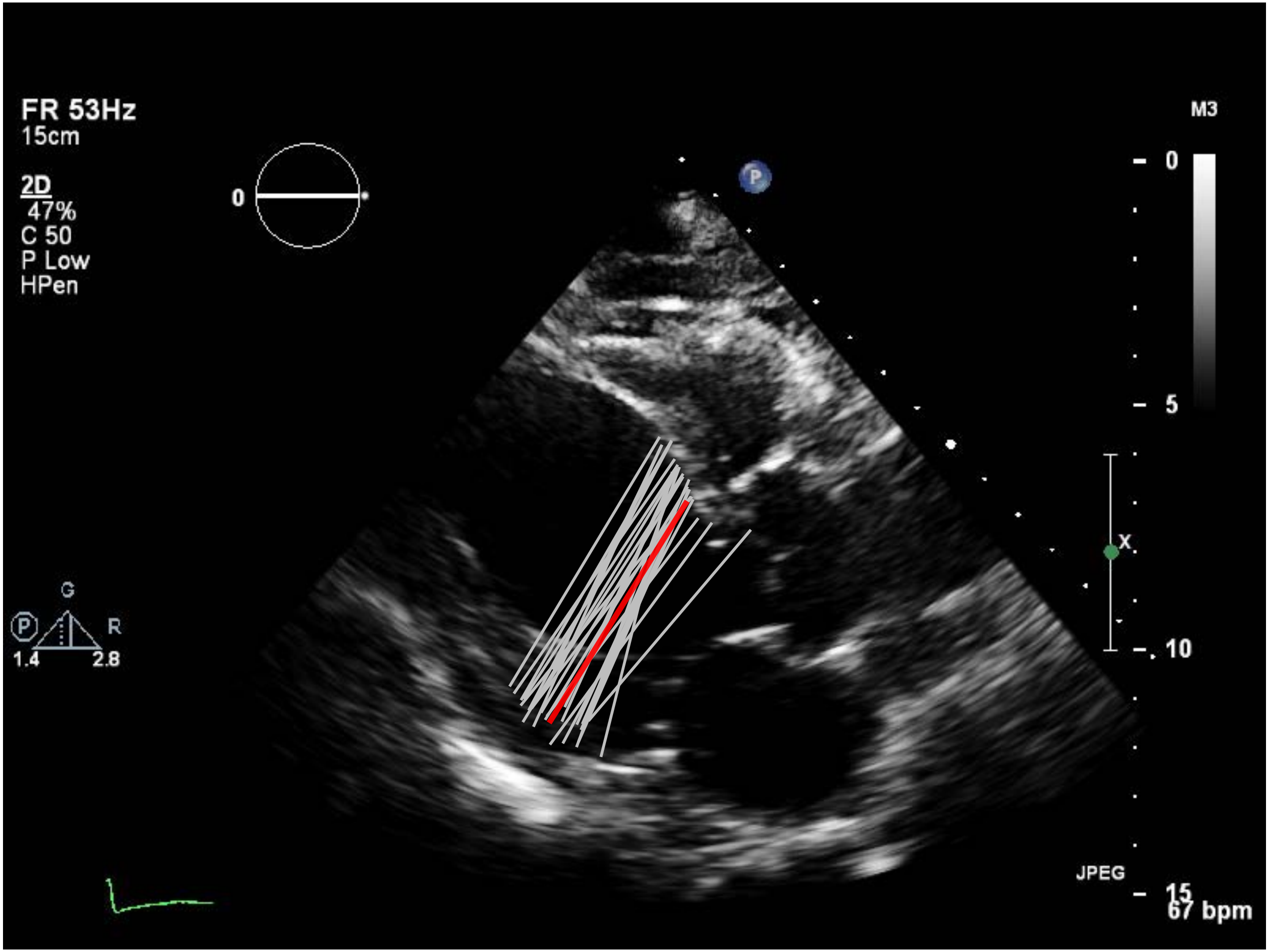

Pt 43 Systole

Absolute difference between AI and expert consensus: 3 mm

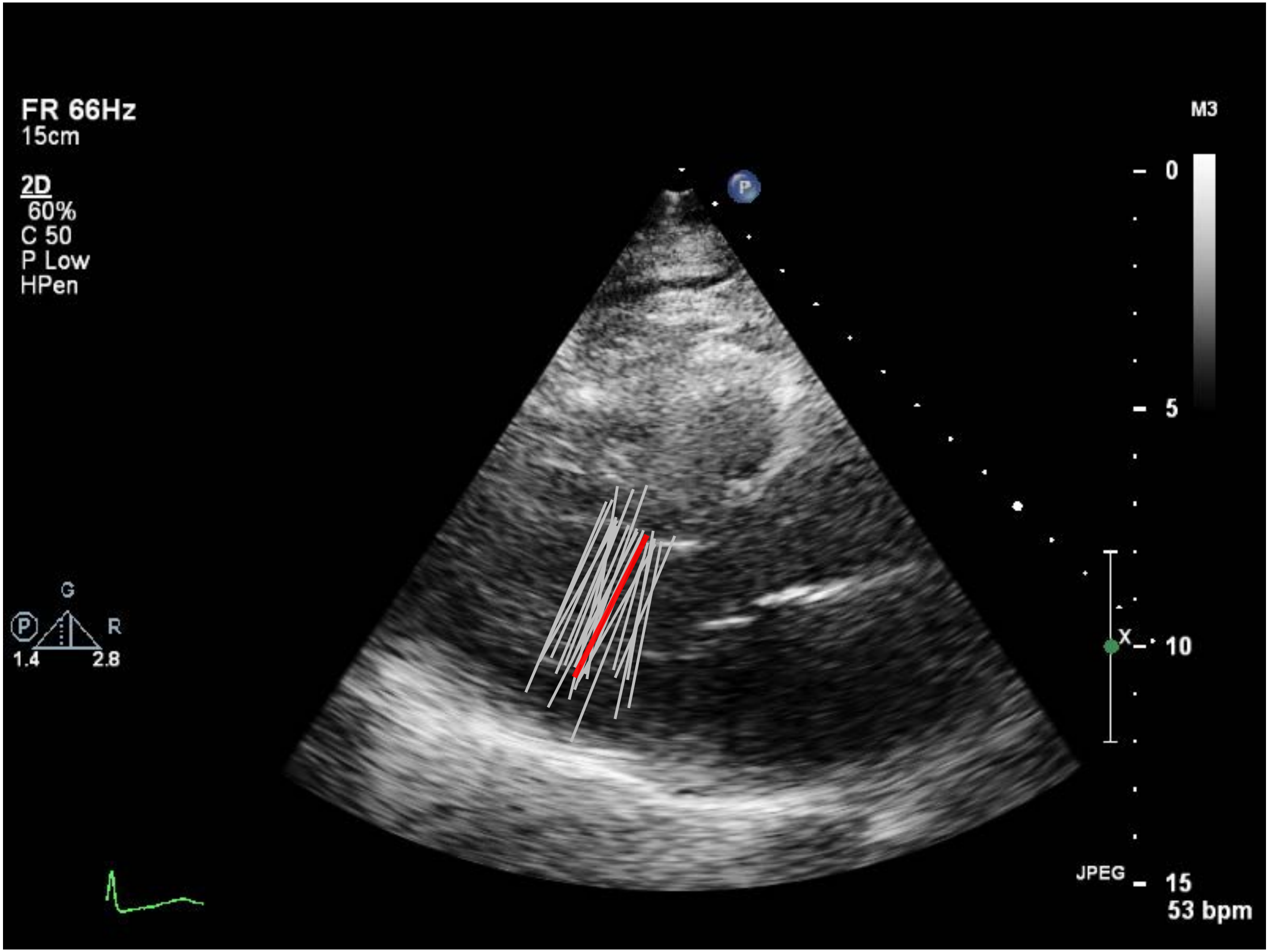

Pt 51 Systole

Absolute difference between AI and expert consensus: 3 mm

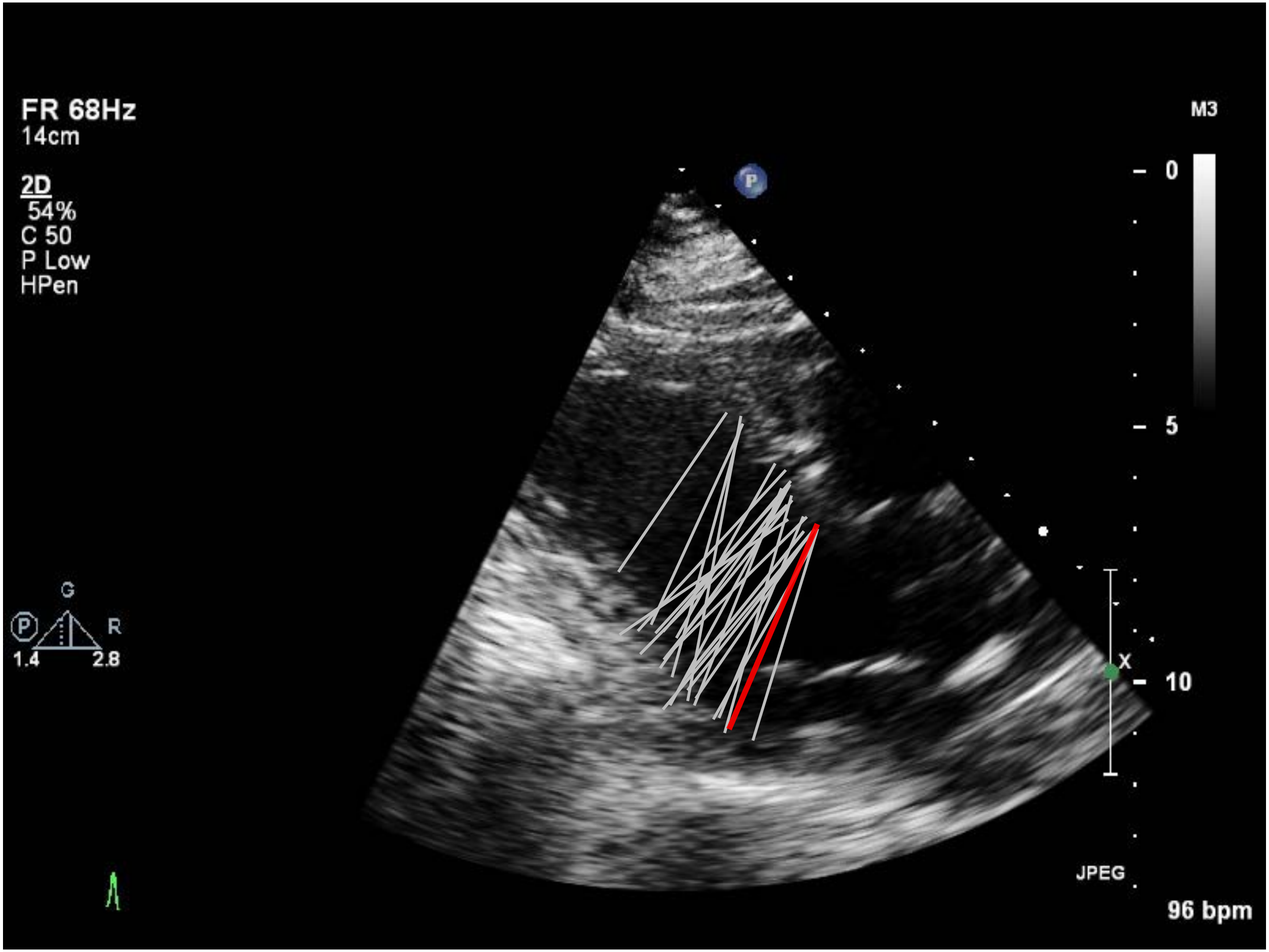

Pt 49 Diastole

Absolute difference between AI and expert consensus: 3 mm

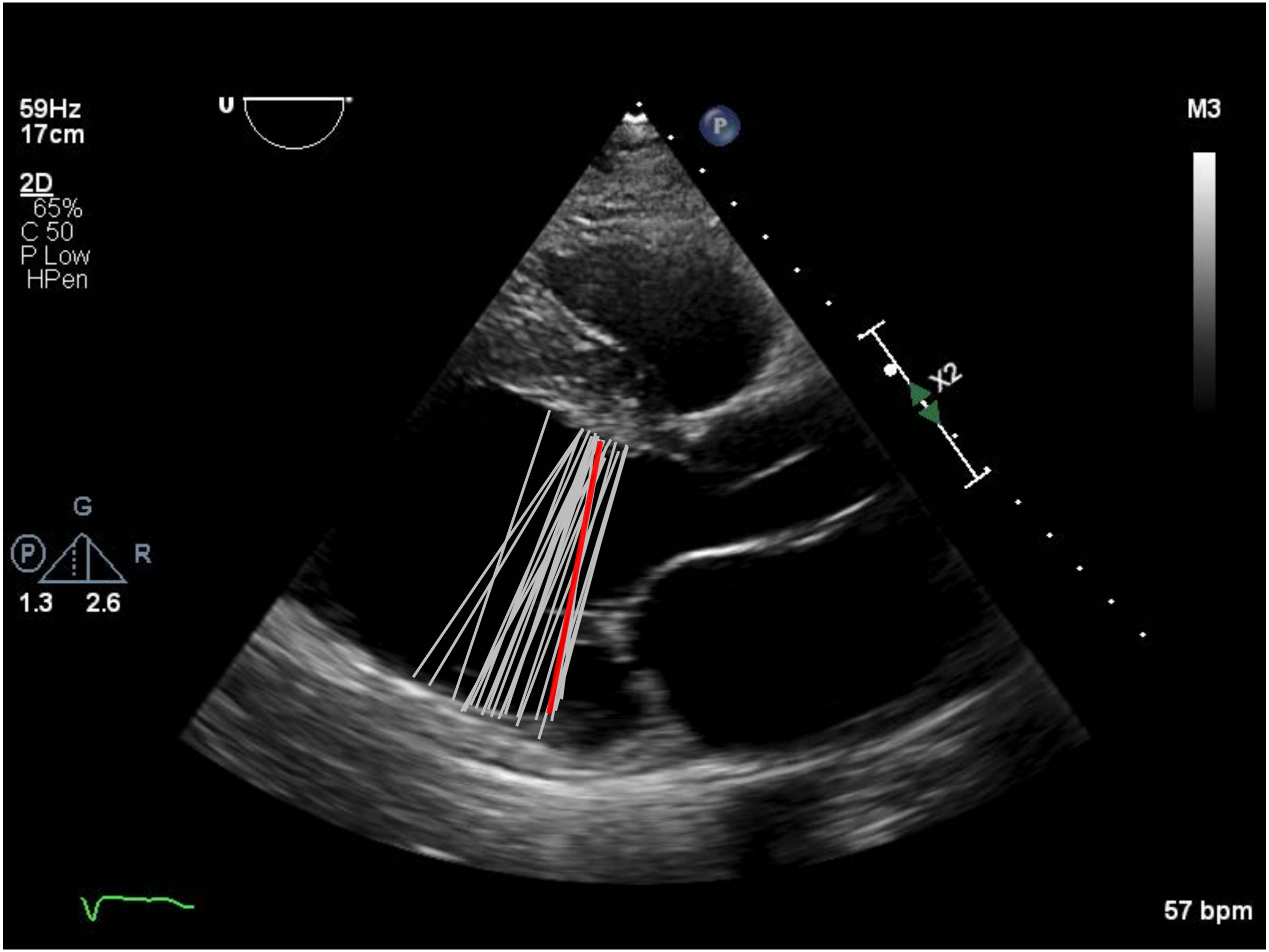

Pt 79 Systole

Absolute difference between AI and expert consensus: 3.1 mm

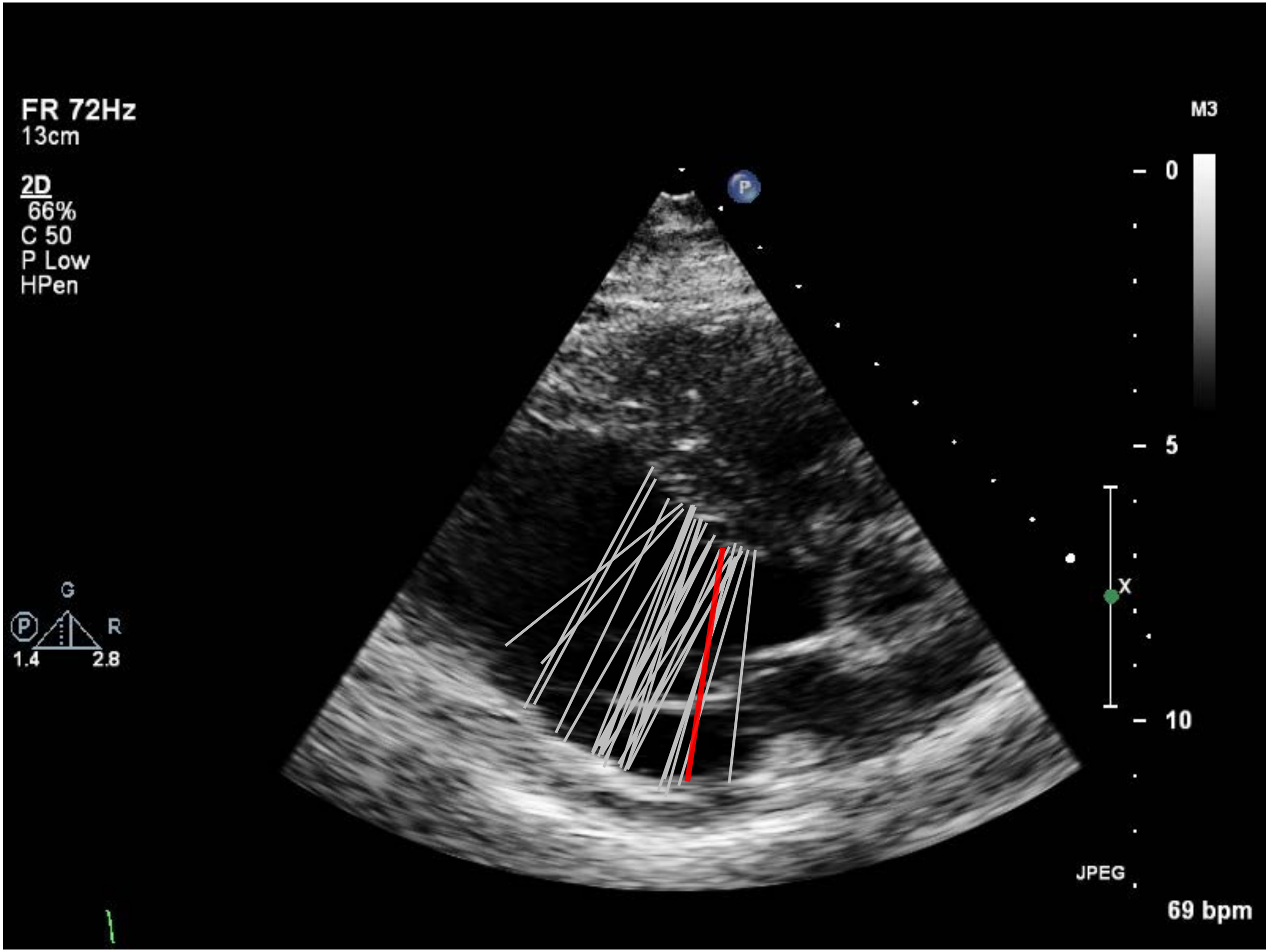

Pt 67 Diastole

Absolute difference between AI and expert consensus: 3.1 mm

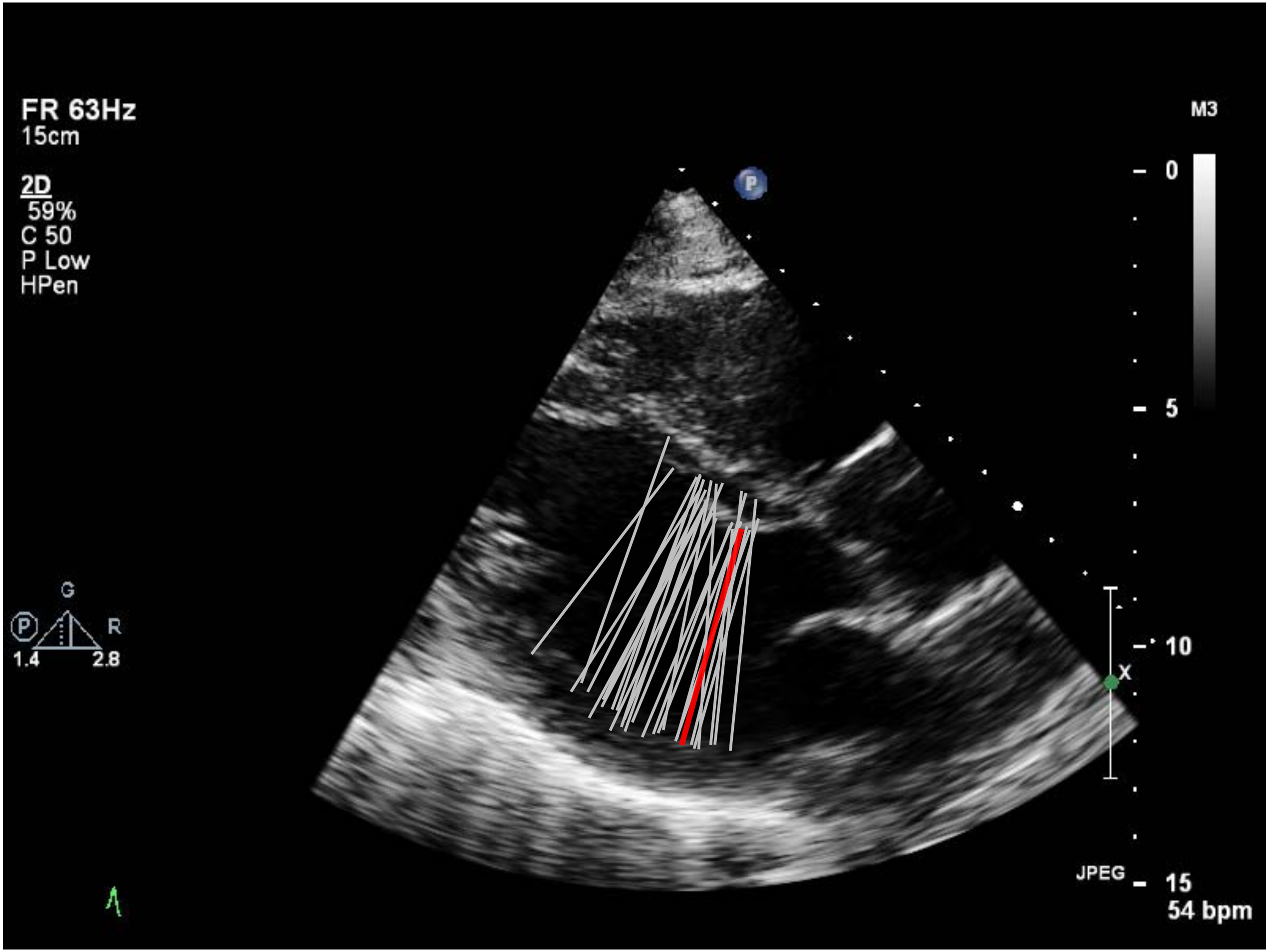

Pt 69 Diastole

Absolute difference between AI and expert consensus: 3.2 mm

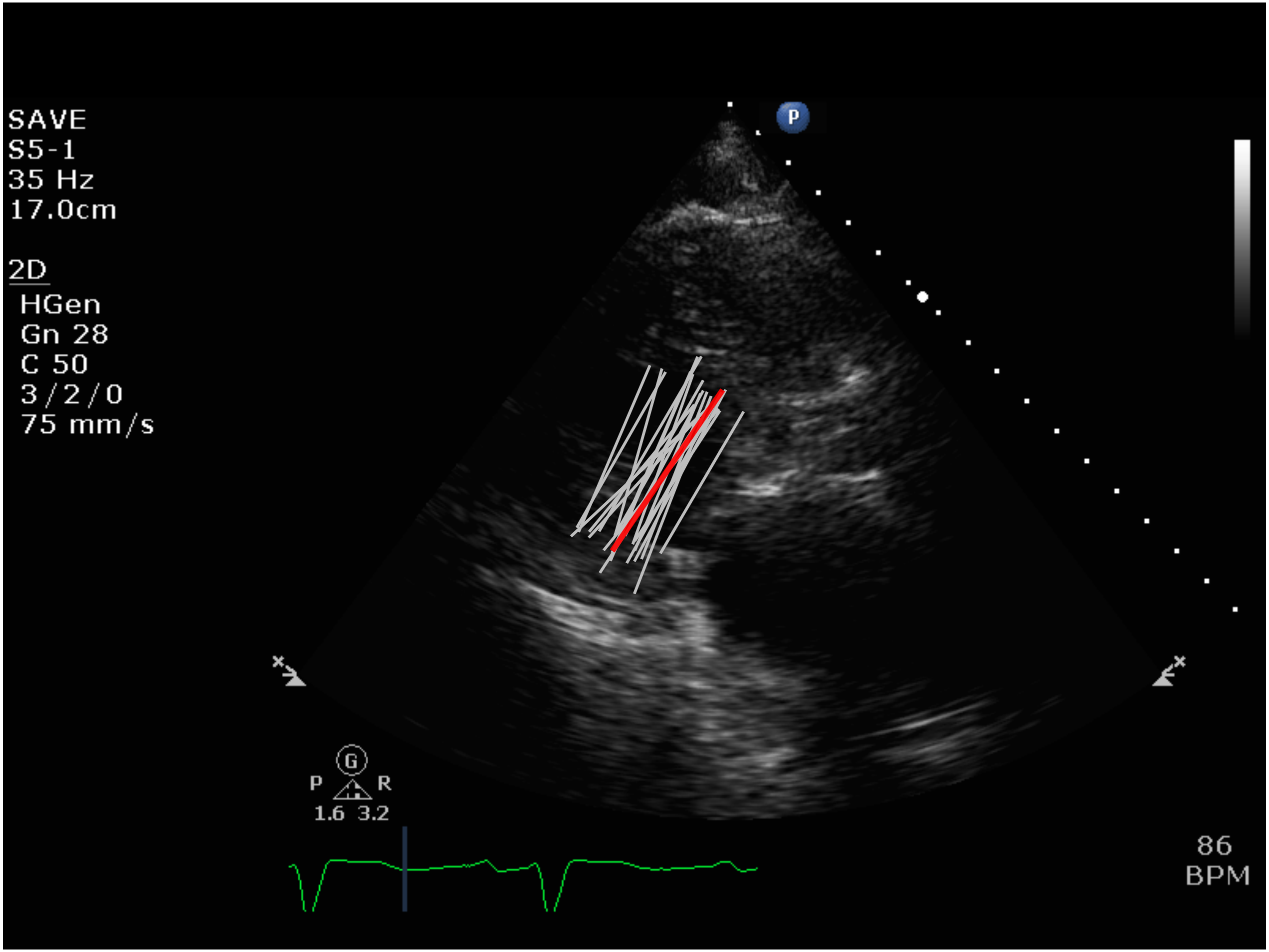

Pt 18 Systole

Absolute difference between AI and expert consensus: 3.2 mm

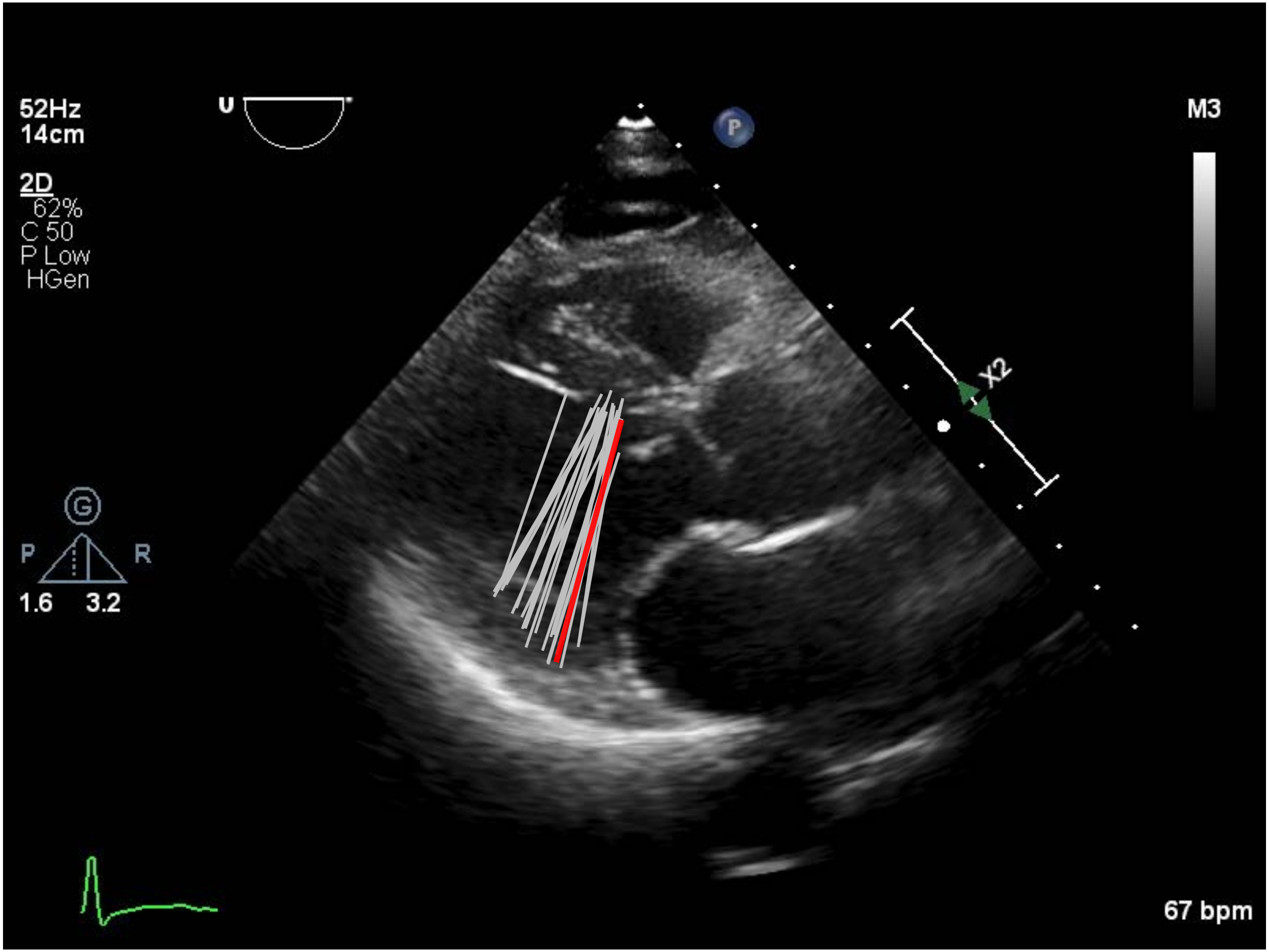

Pt 80 Systole

Absolute difference between AI and expert consensus: 3.3 mm

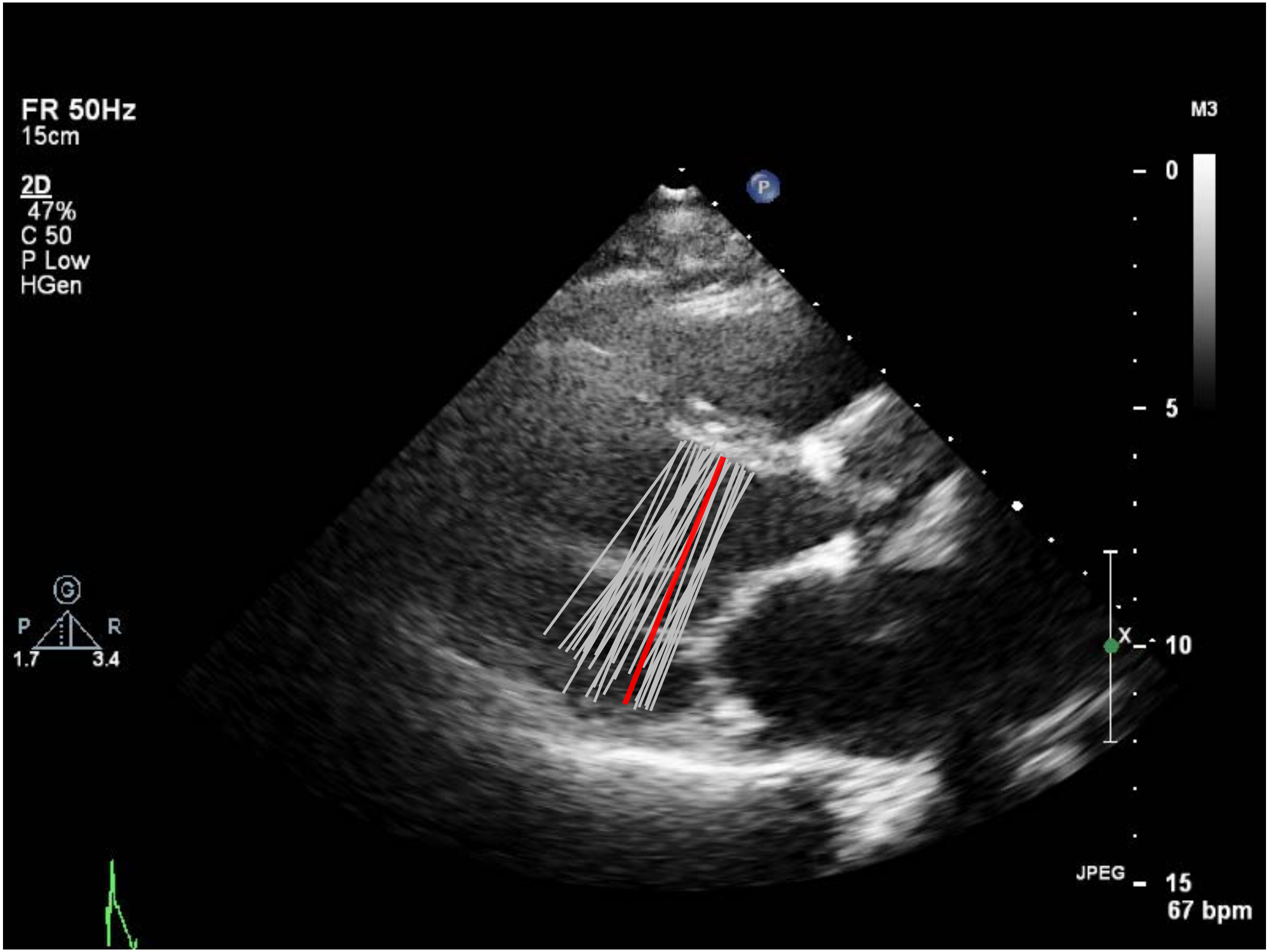

Pt 72 Diastole

Absolute difference between AI and expert consensus: 3.3 mm

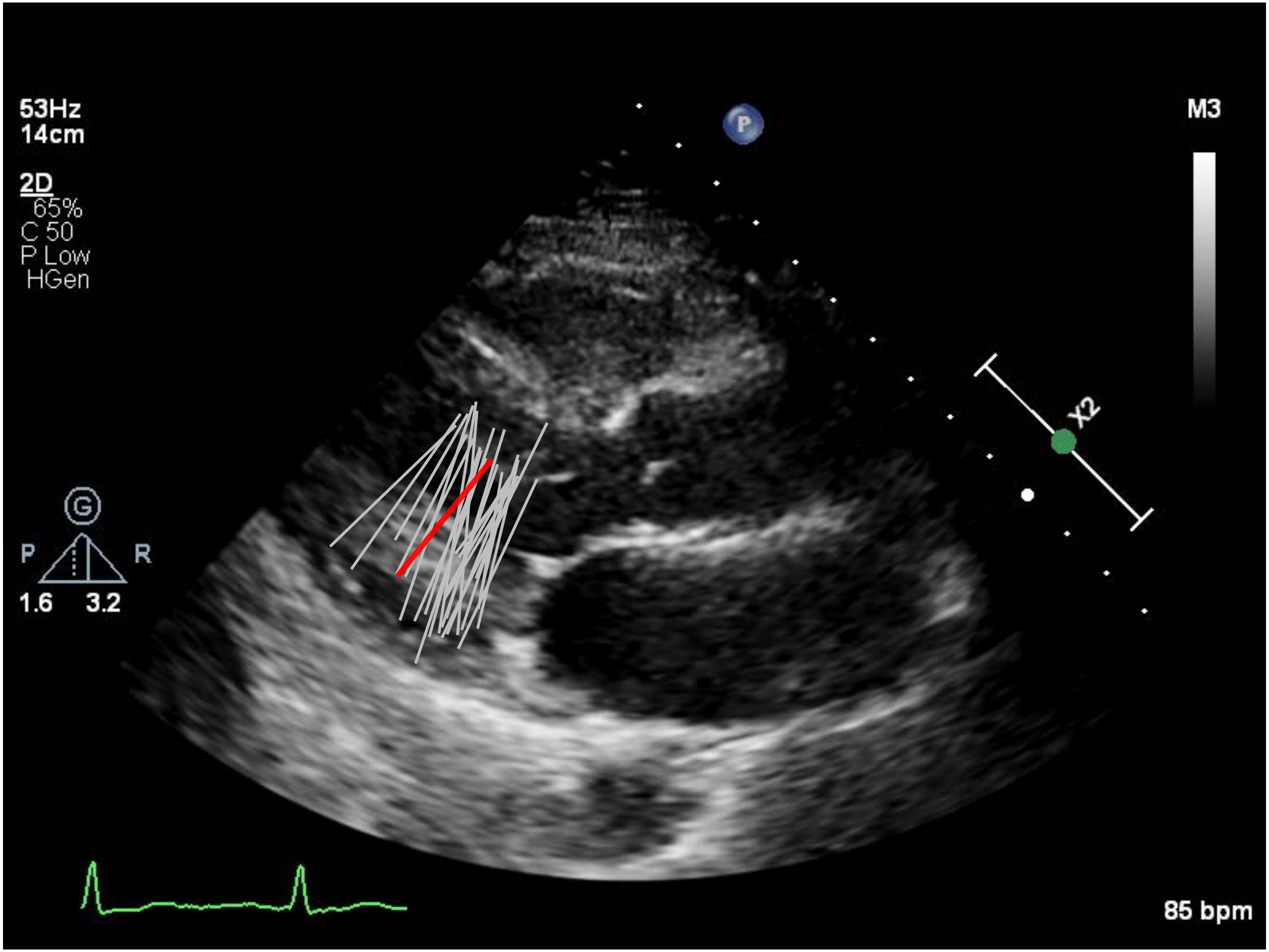

Pt 74 Systole

Absolute difference between AI and expert consensus: 3.4 mm

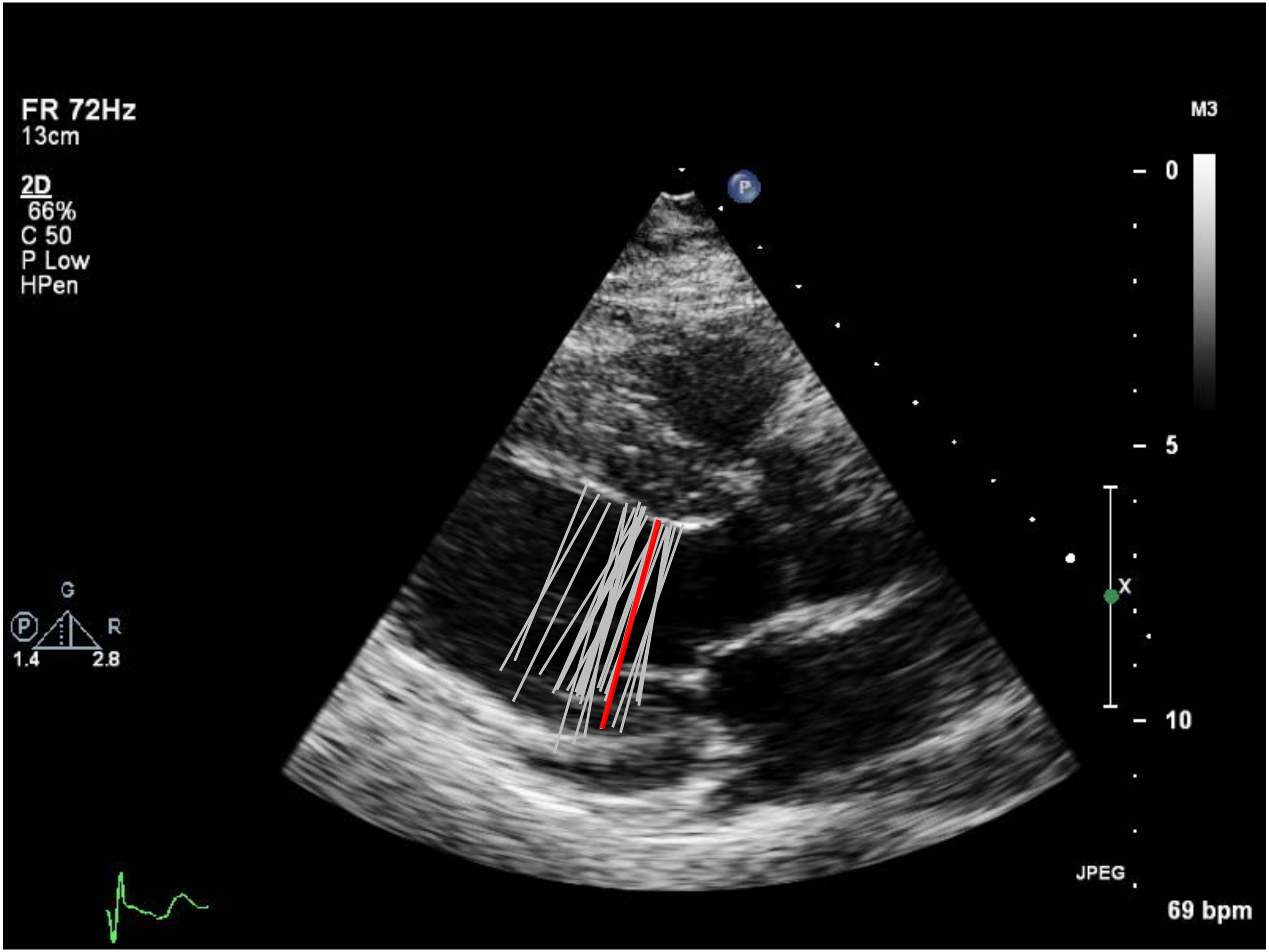

Pt 67 Systole

Absolute difference between AI and expert consensus: 3.5 mm

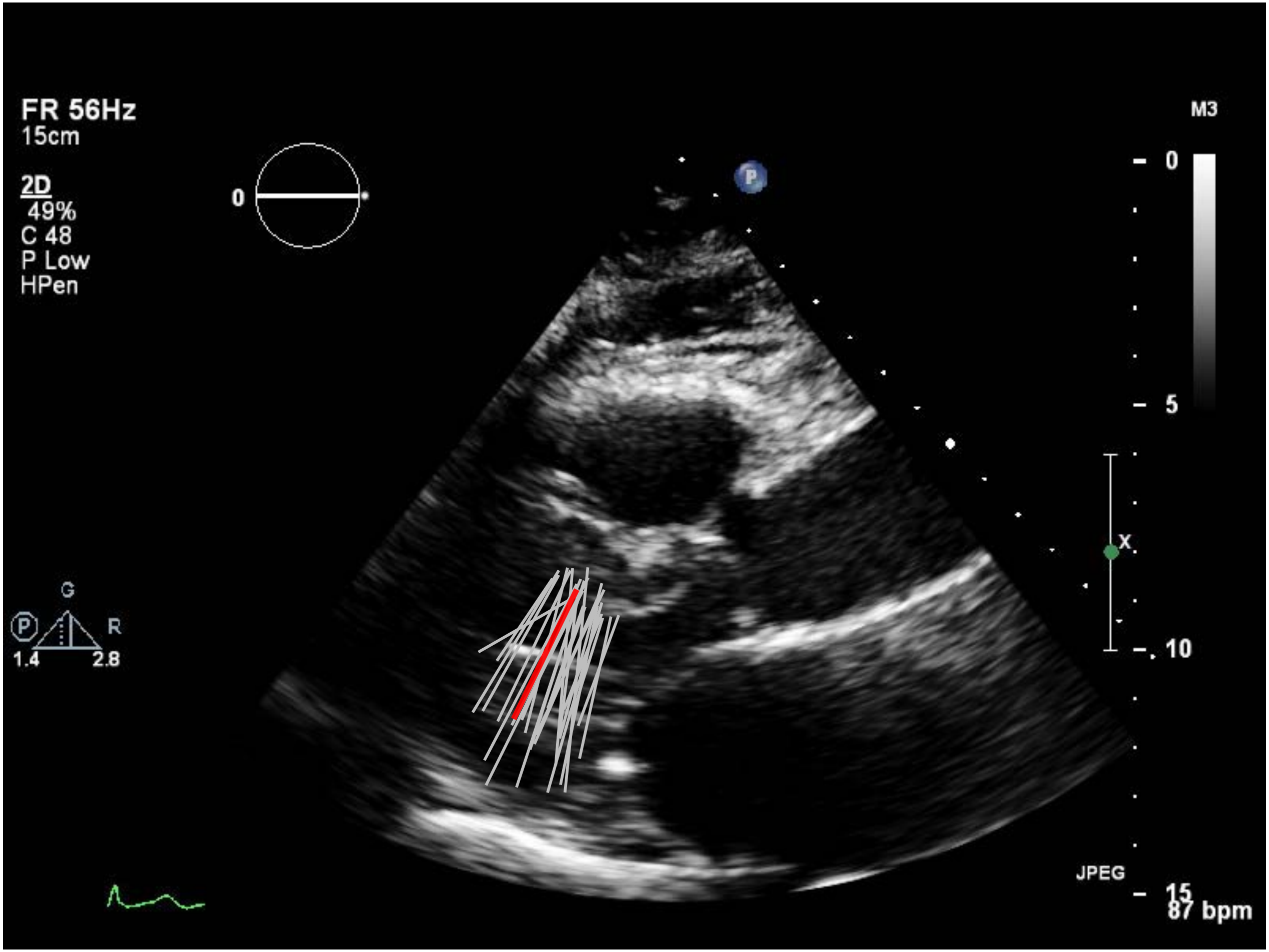

Pt 21 Systole

Absolute difference between AI and expert consensus: 3.7 mm

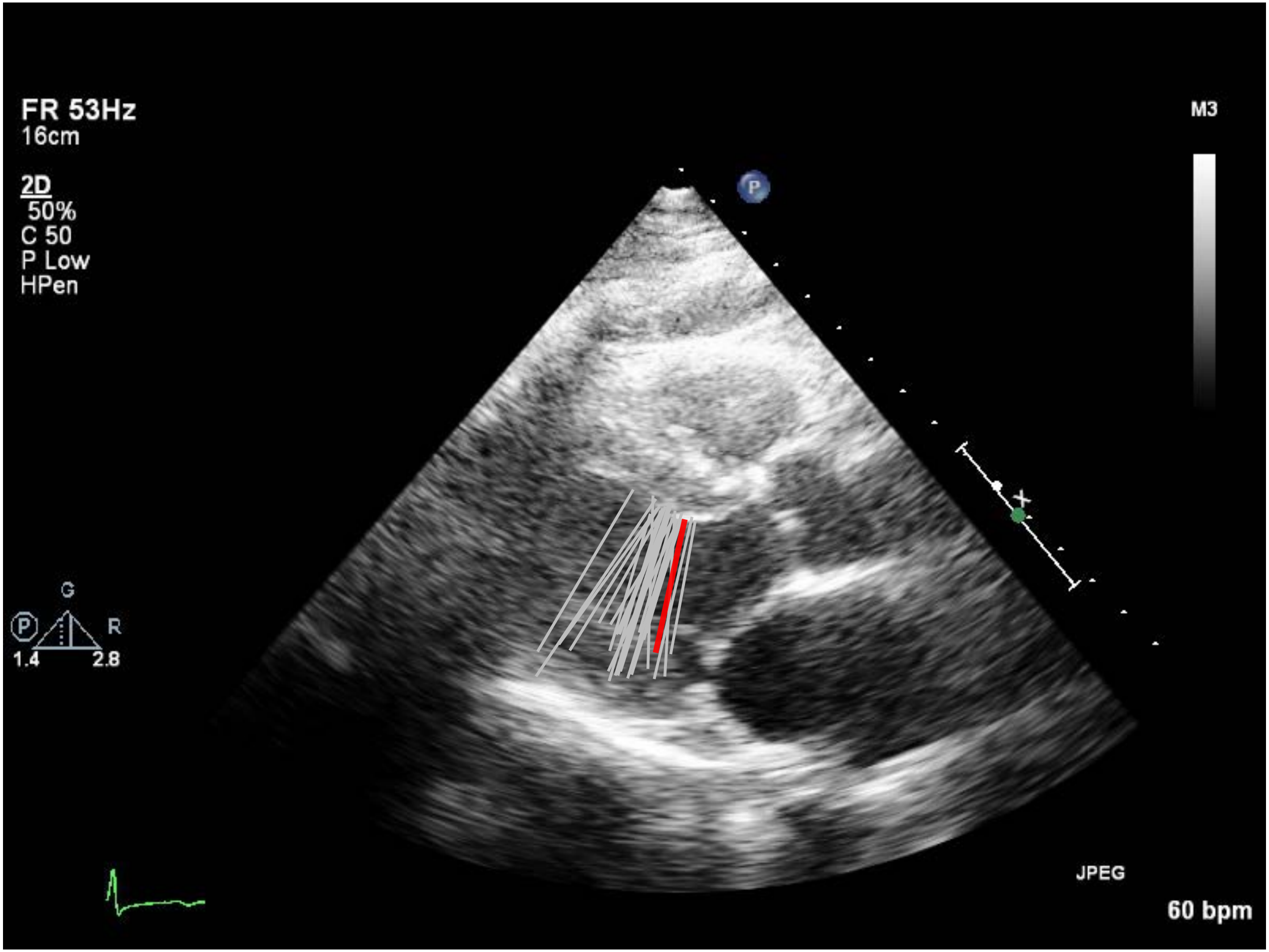

Pt 47 Systole

Absolute difference between AI and expert consensus: 3.7 mm

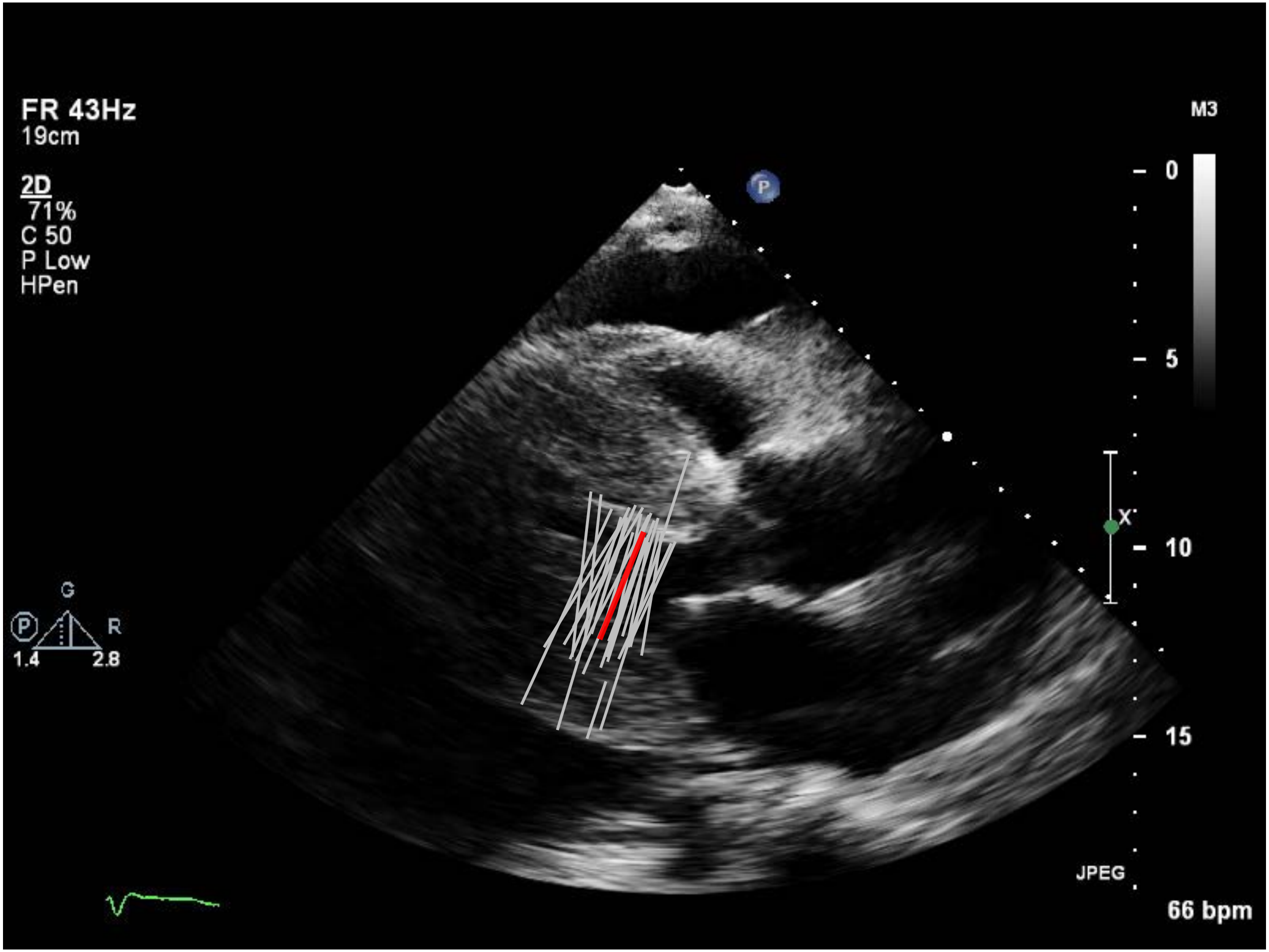

Pt 100 Systole

Absolute difference between AI and expert consensus: 3.7 mm

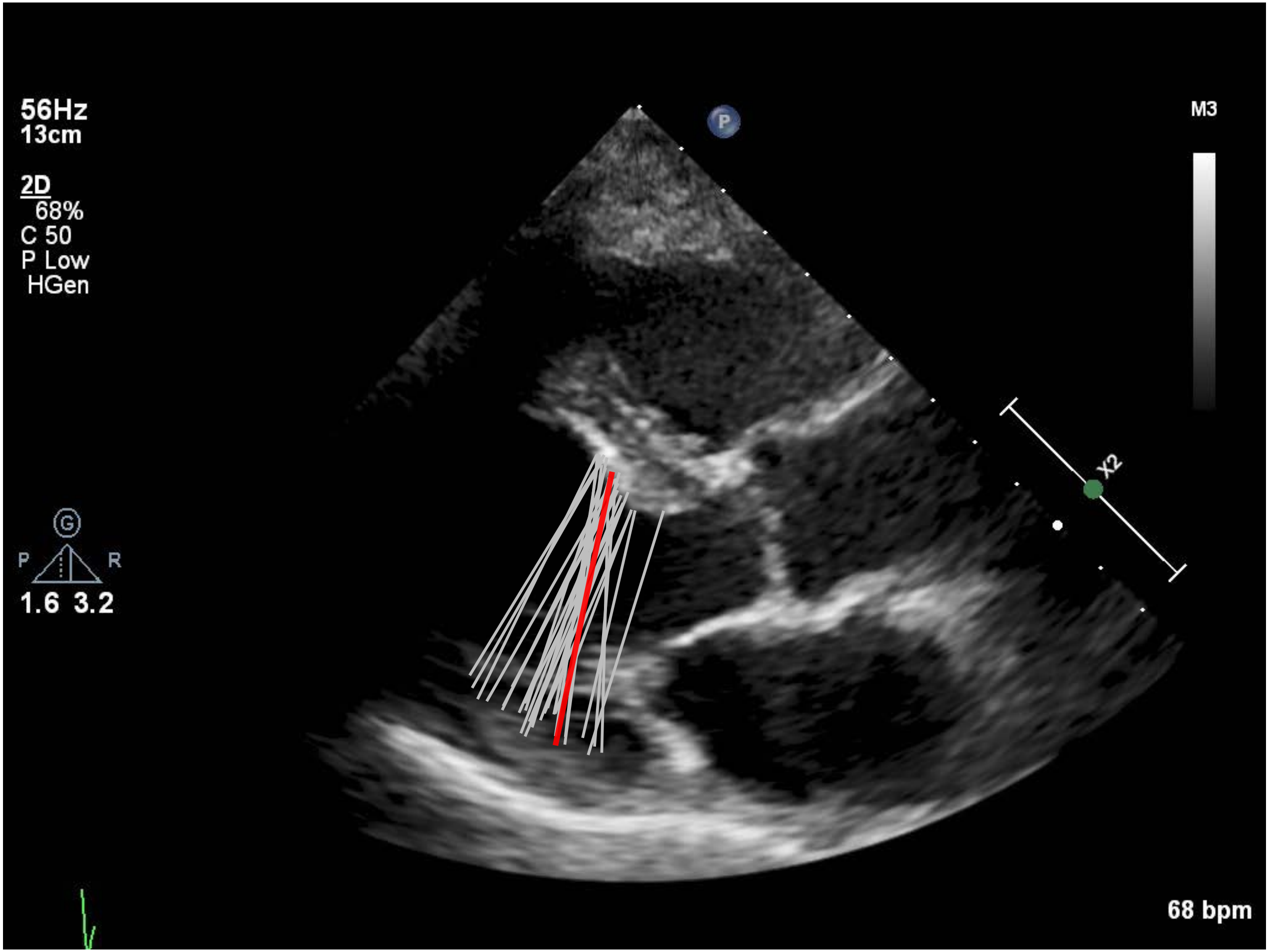

Pt 57 Diastole

Absolute difference between AI and expert consensus: 3.7 mm

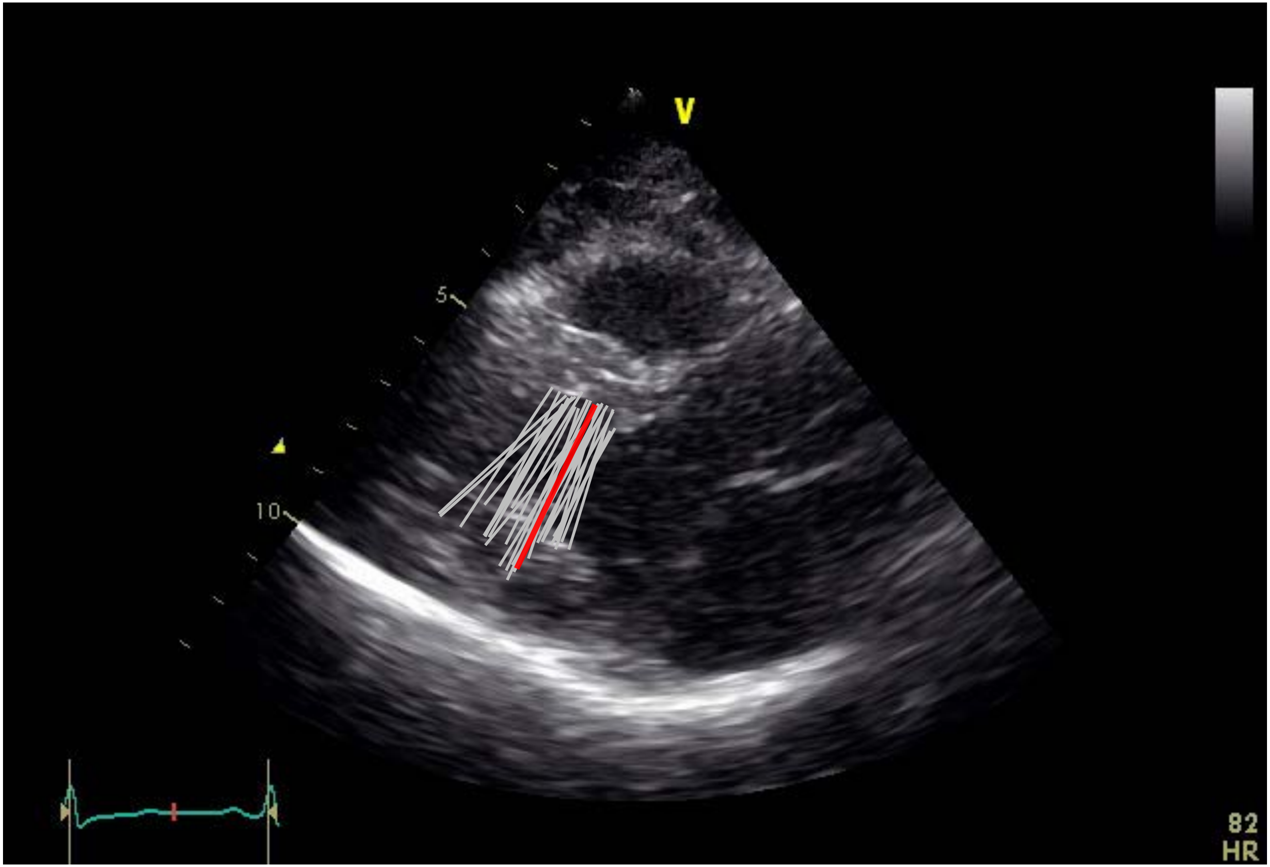

Pt 35 Systole

Absolute difference between AI and expert consensus: 3.9 mm

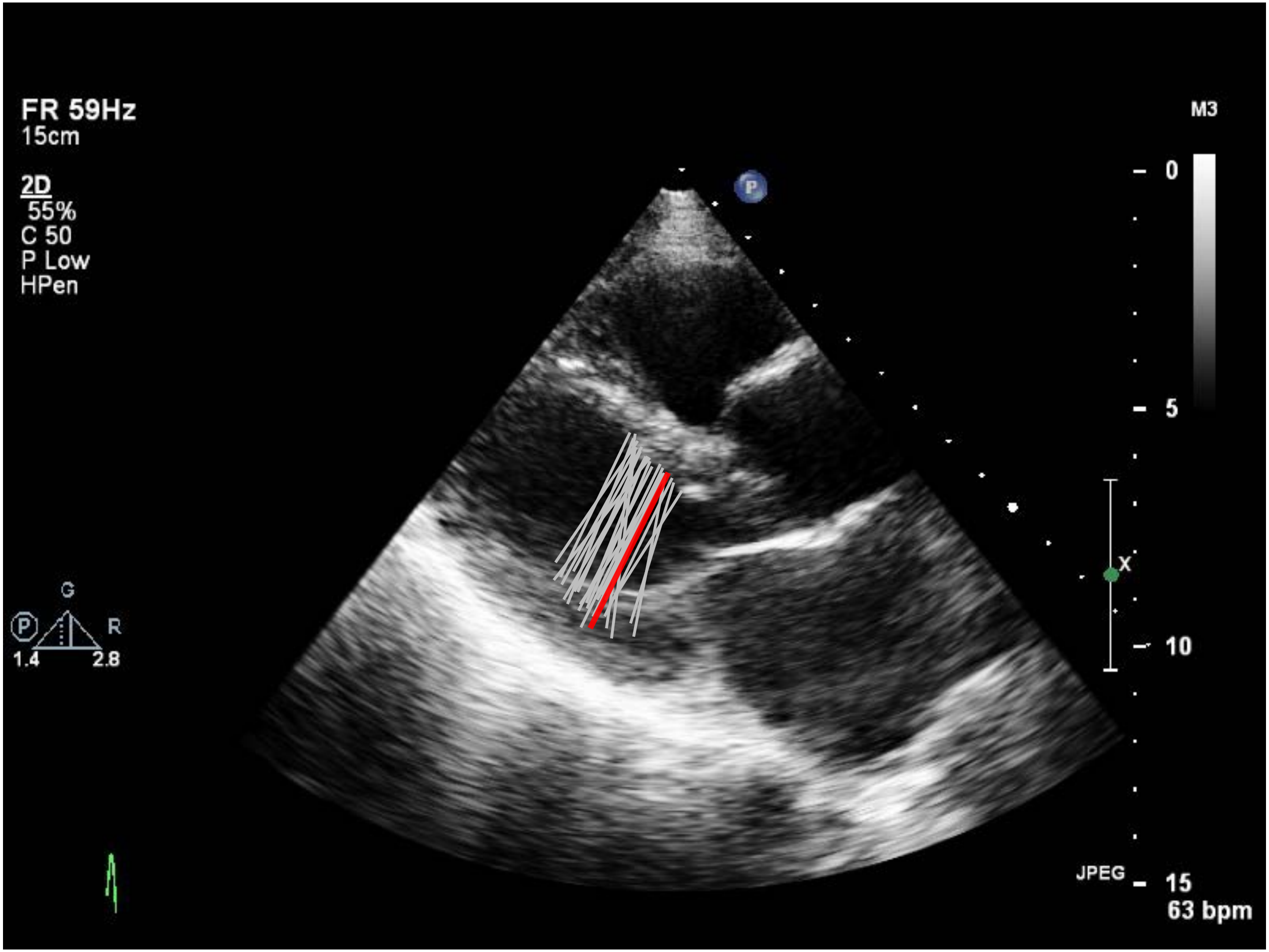

Pt 45 Diastole

Absolute difference between AI and expert consensus: 3.9 mm

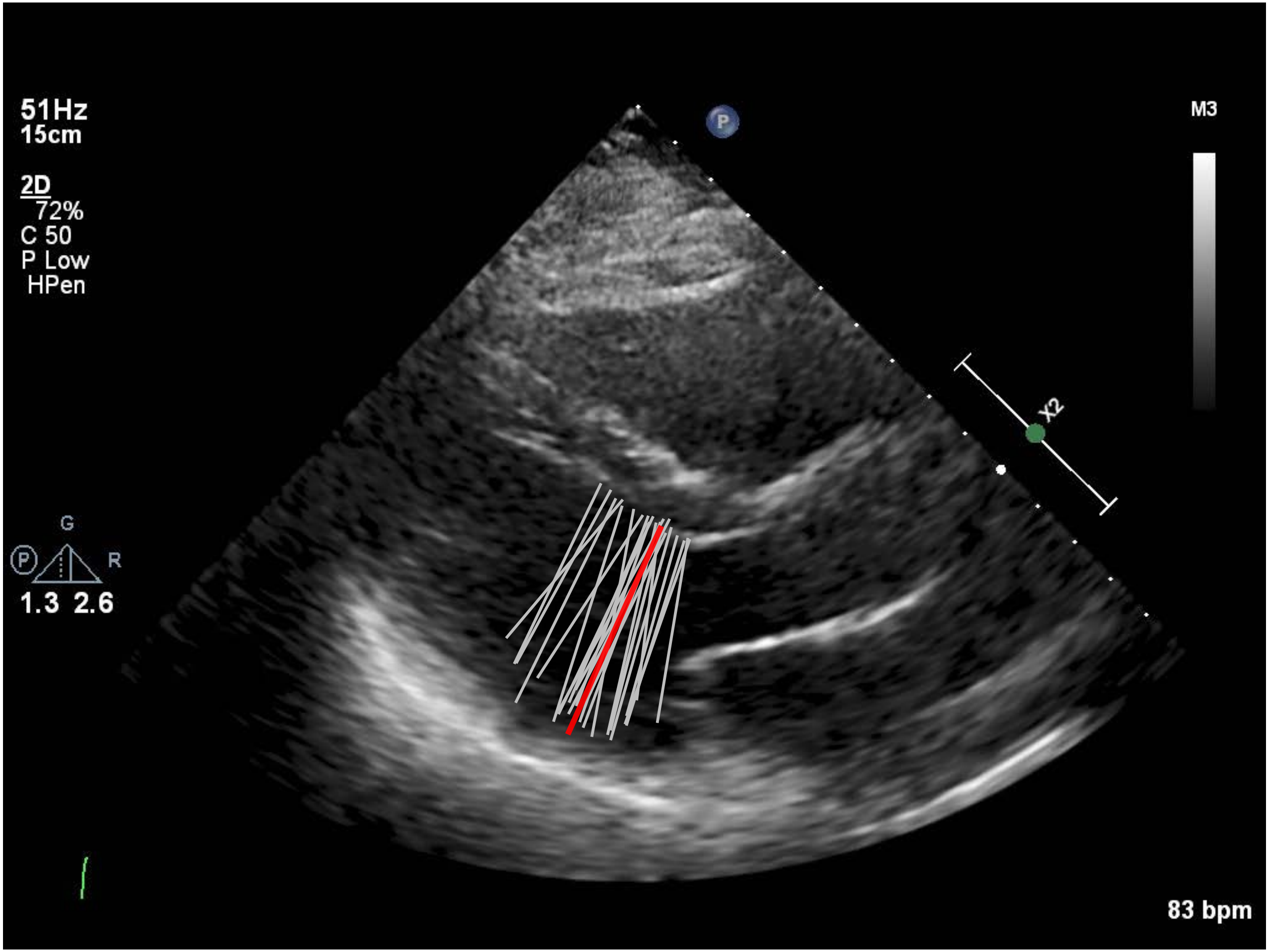

Pt 63 Diastole
Absolute difference between AI and expert consensus: 4 mm

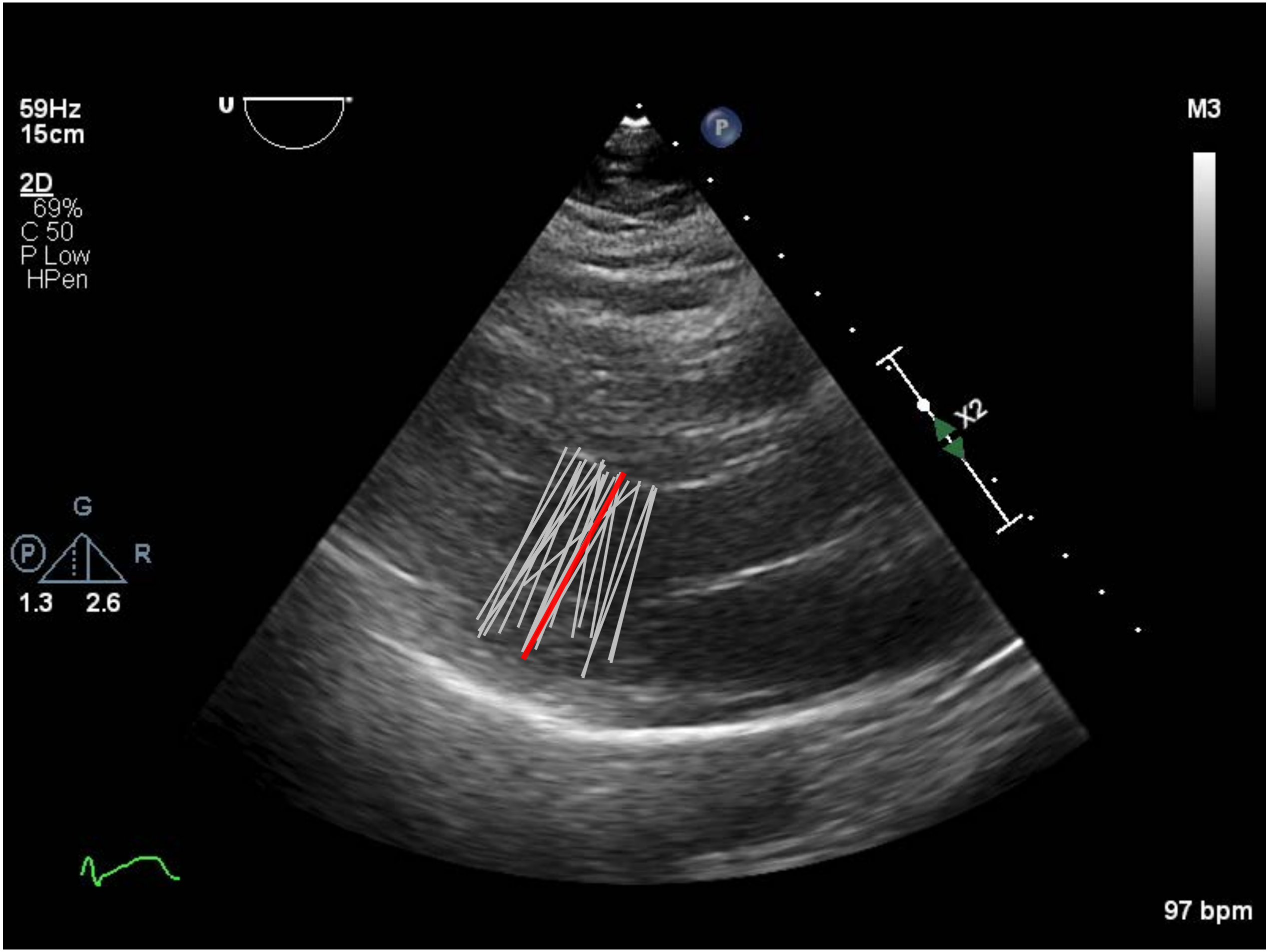

Pt 39 Systole

Absolute difference between AI and expert consensus: 4.1 mm

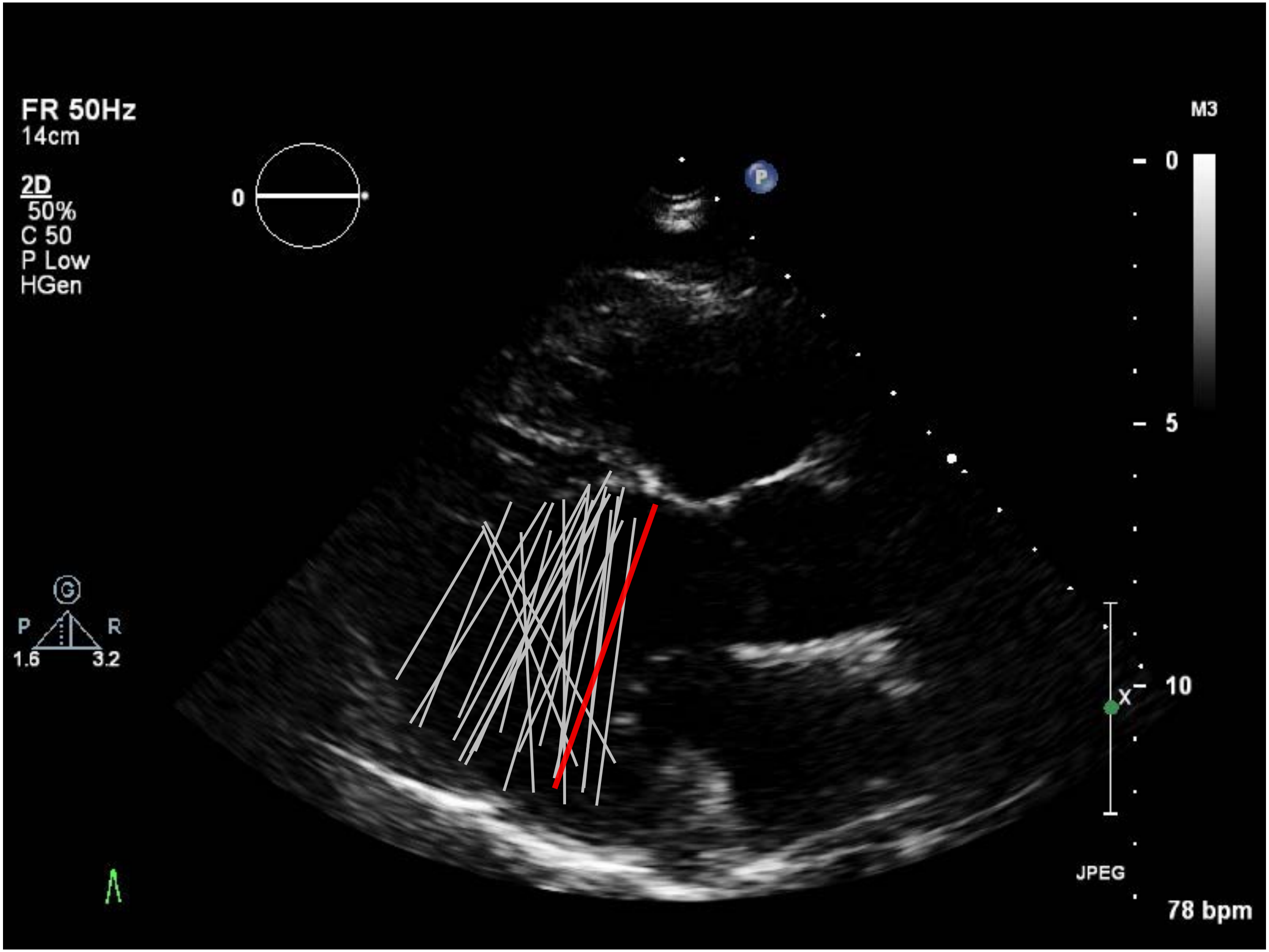

Pt 50 Diastole

Absolute difference between AI and expert consensus: 4.3 mm

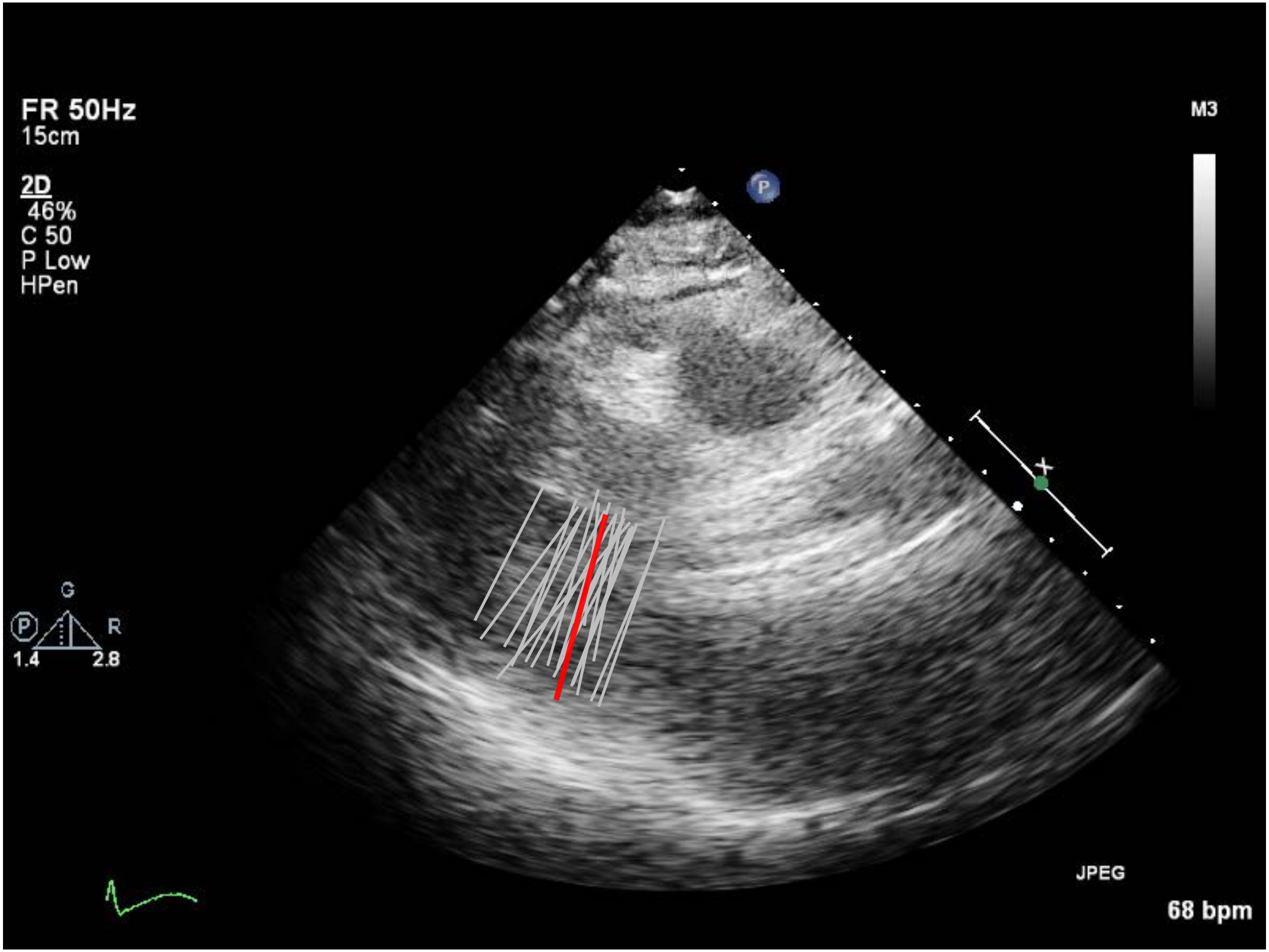

Pt 81 Systole

Absolute difference between AI and expert consensus: 4.4 mm

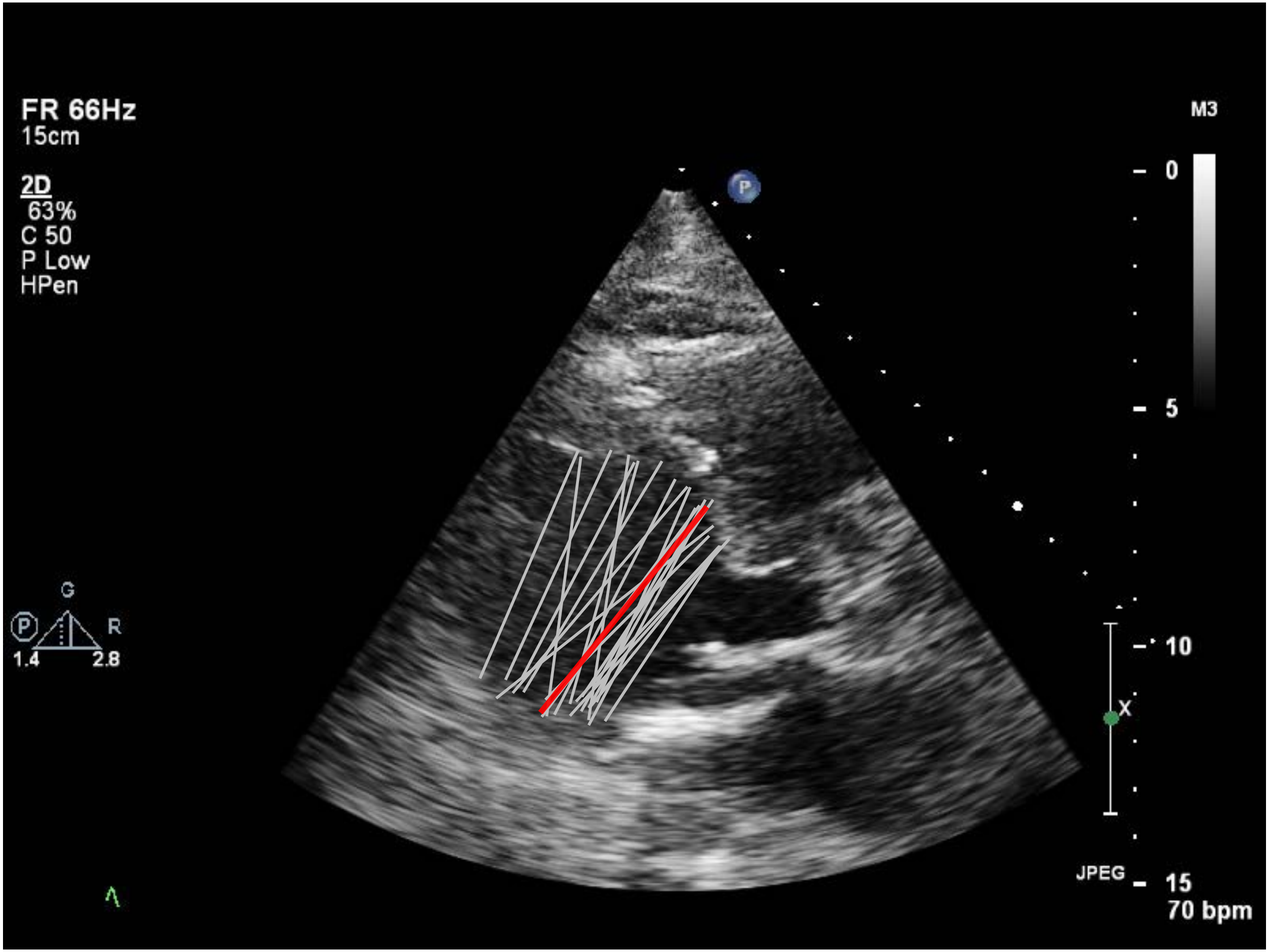

Pt 62 Diastole

Absolute difference between AI and expert consensus: 4.4 mm

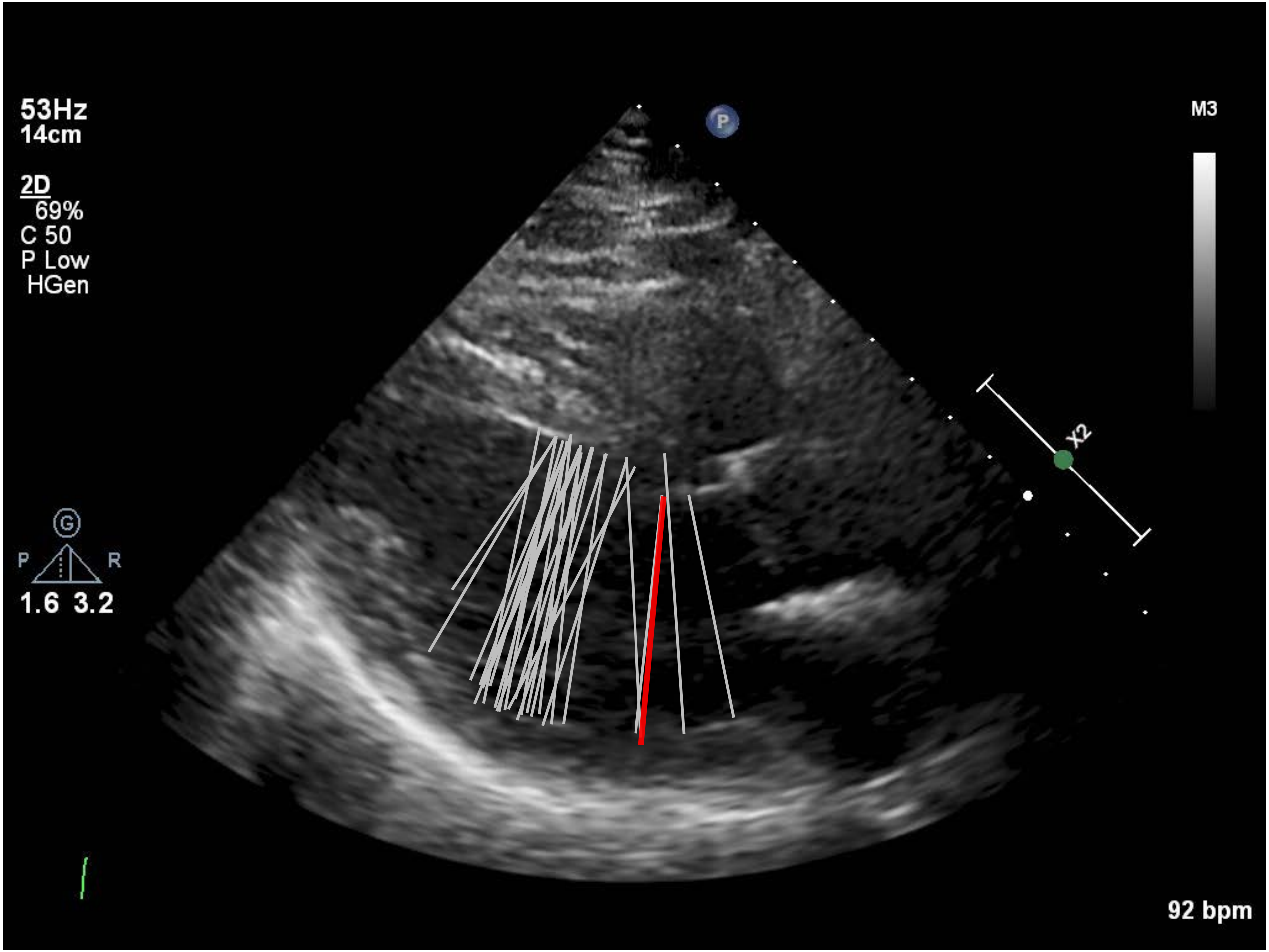

Pt 70 Diastole

Absolute difference between AI and expert consensus: 4.6 mm

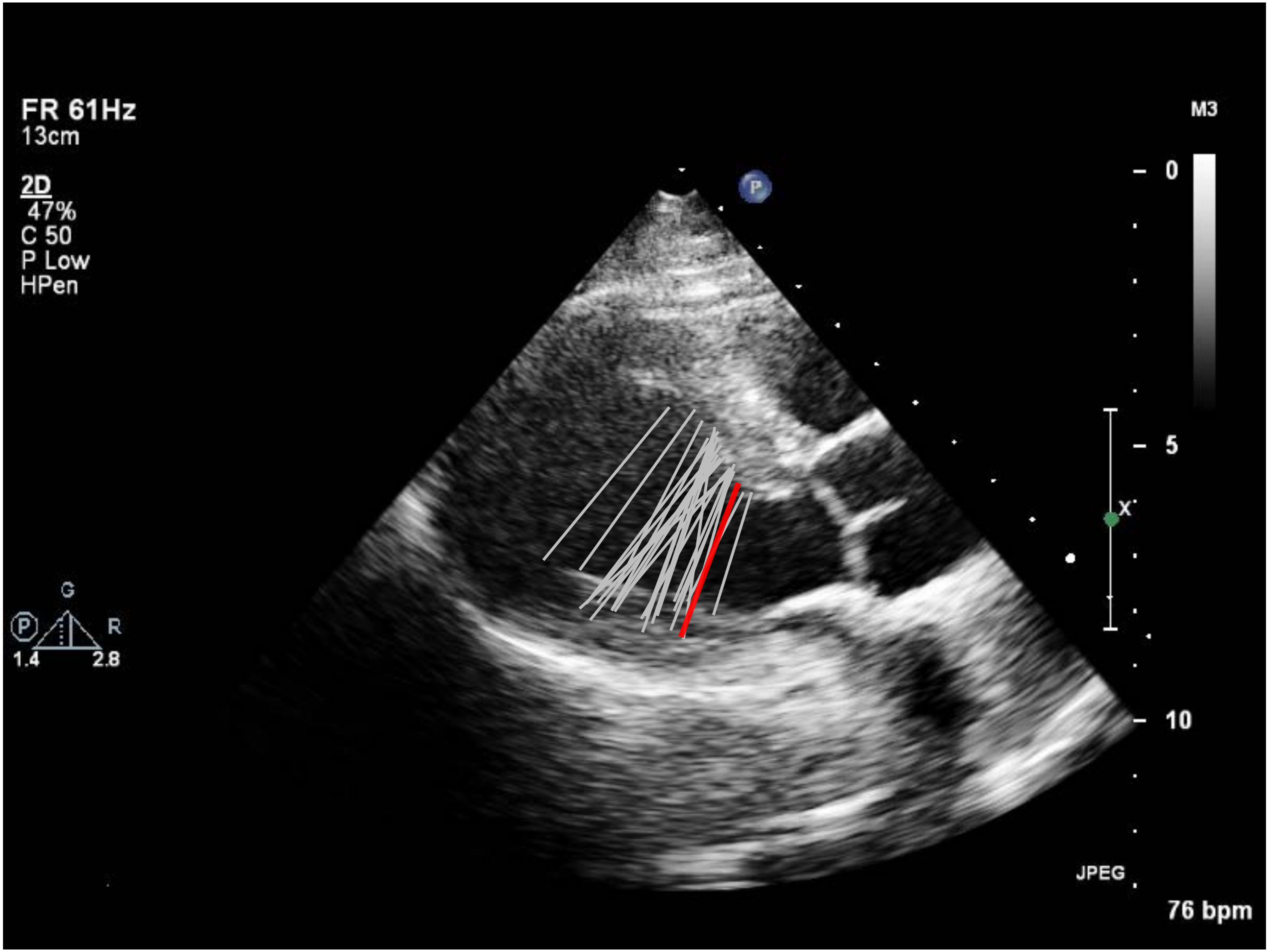

Pt 41 Diastole

Absolute difference between AI and expert consensus: 4.8 mm

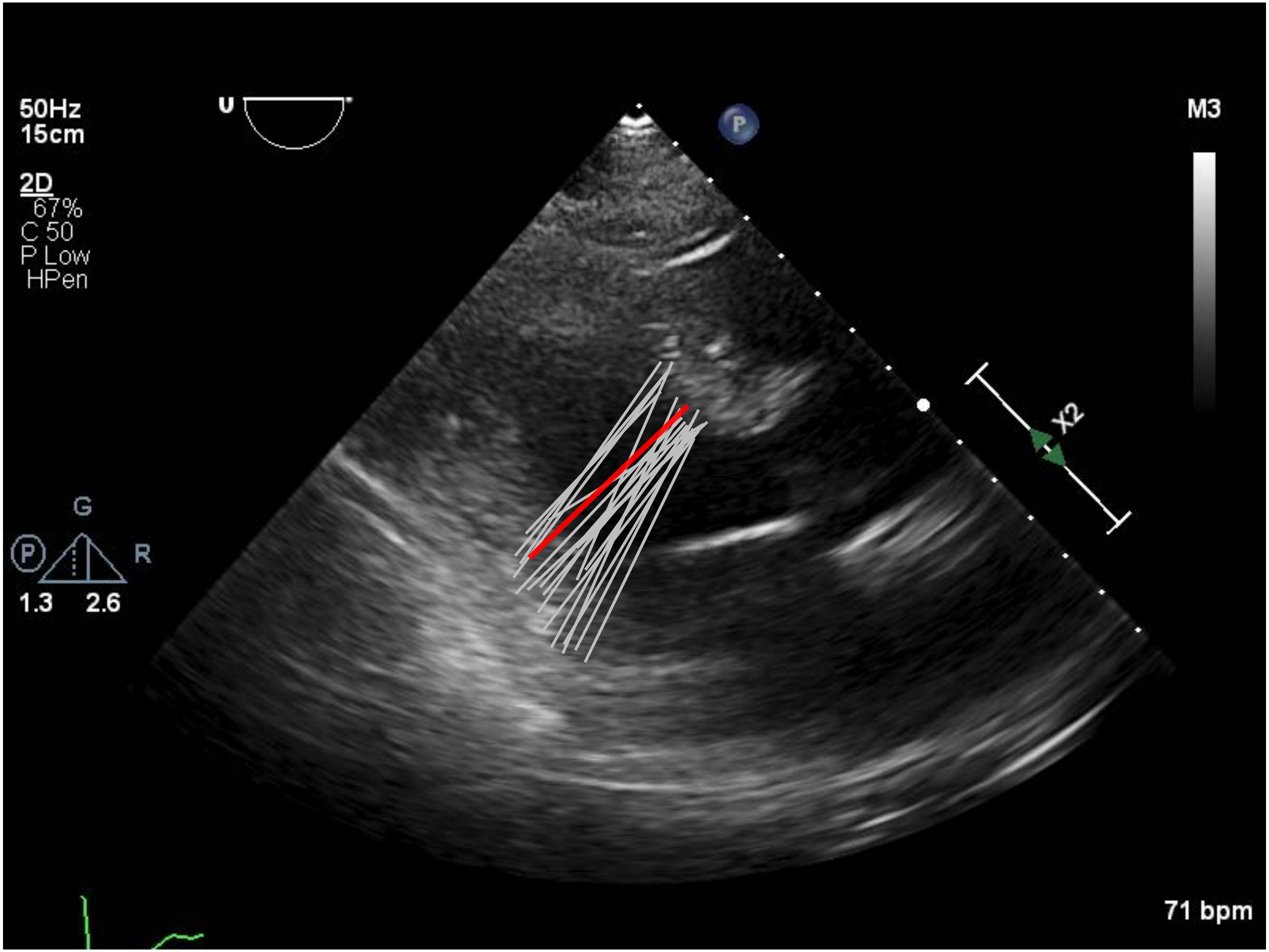

Pt 97 Systole

Absolute difference between AI and expert consensus: 4.8 mm

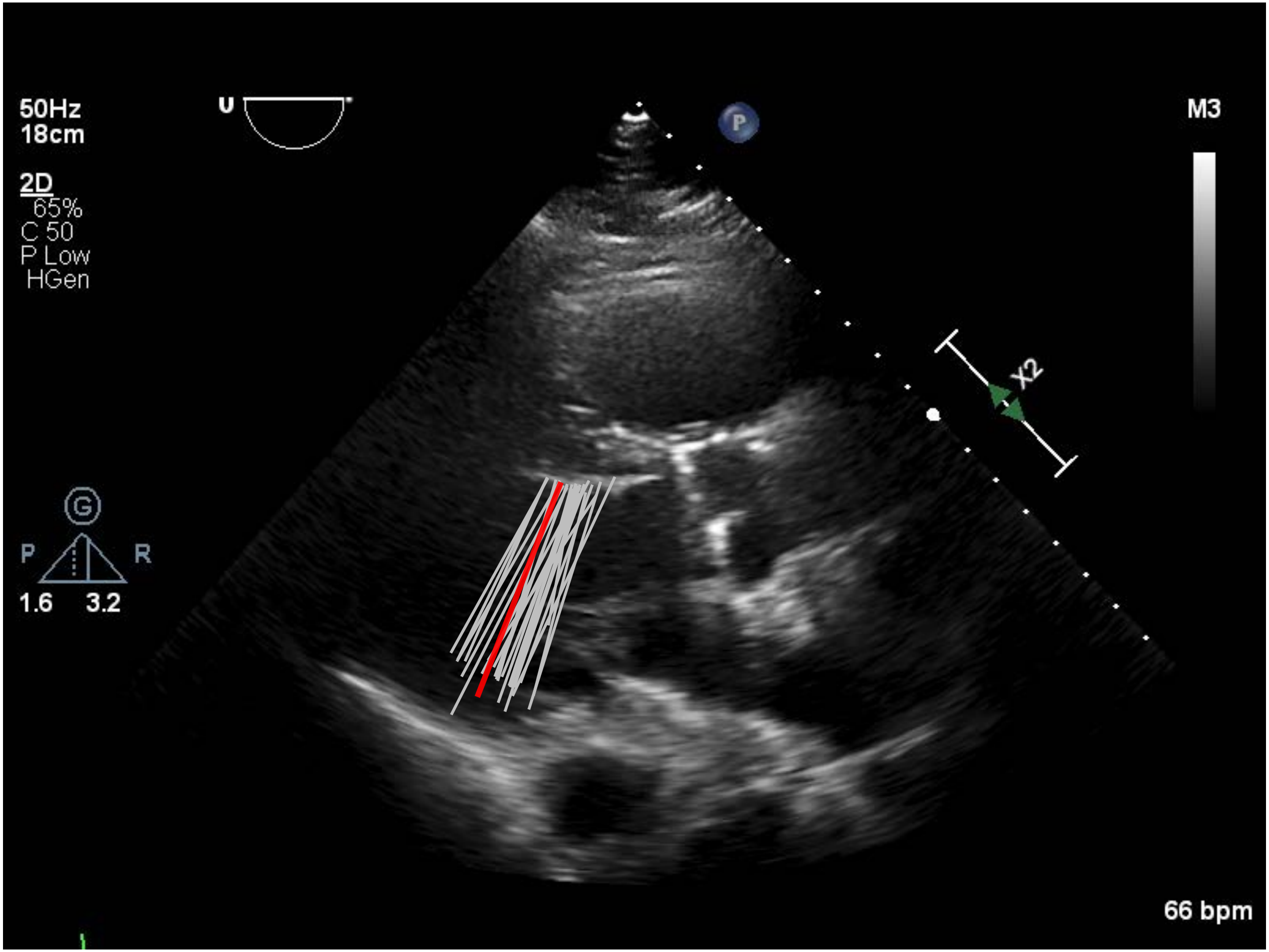

Pt 11 Diastole

Absolute difference between AI and expert consensus: 5 mm

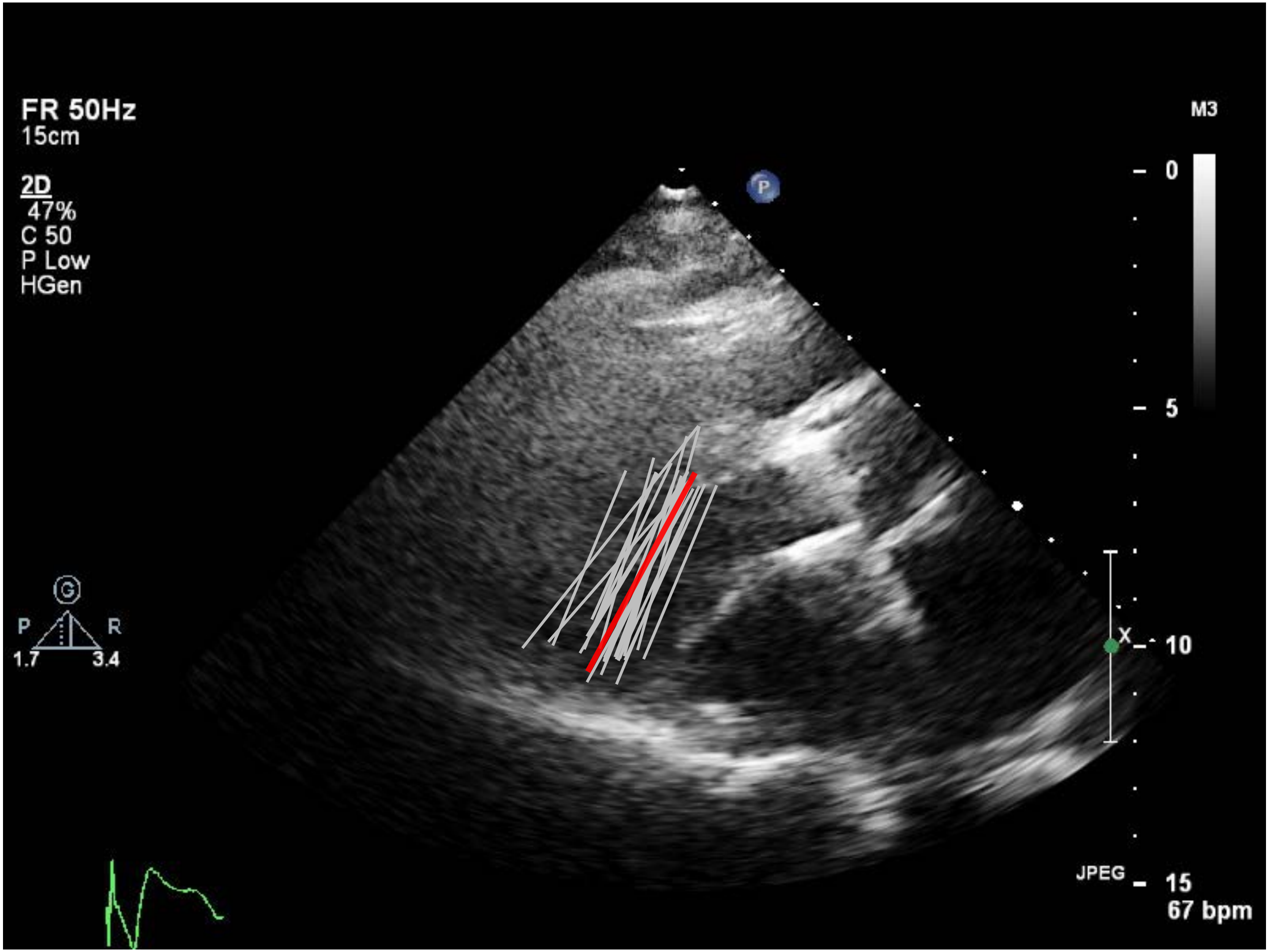

Pt 72 Systole

Absolute difference between AI and expert consensus: 5 mm

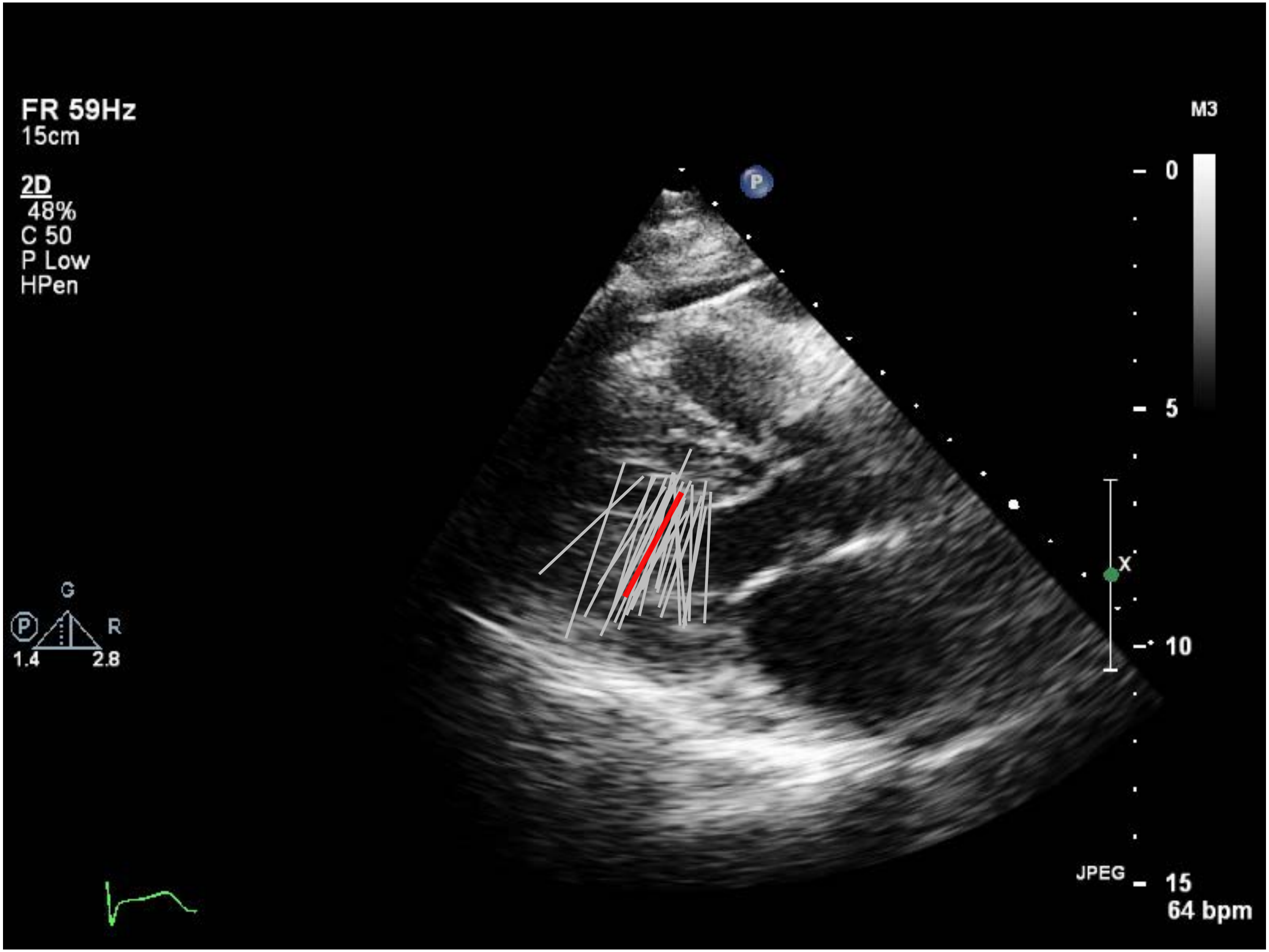

Pt 27 Systole

Absolute difference between AI and expert consensus: 5.2 mm

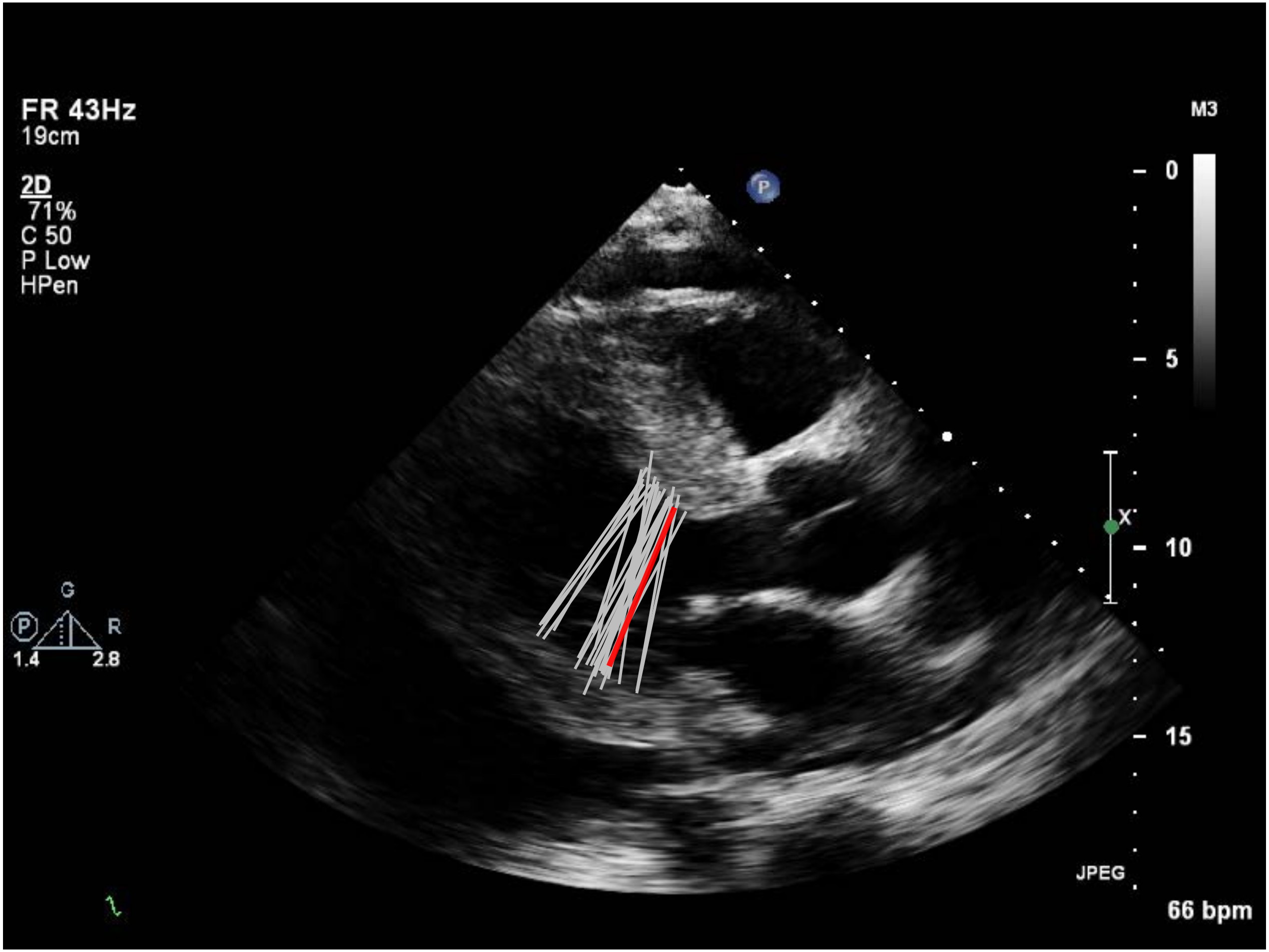

Pt 100 Diastole

Absolute difference between AI and expert consensus: 5.3 mm

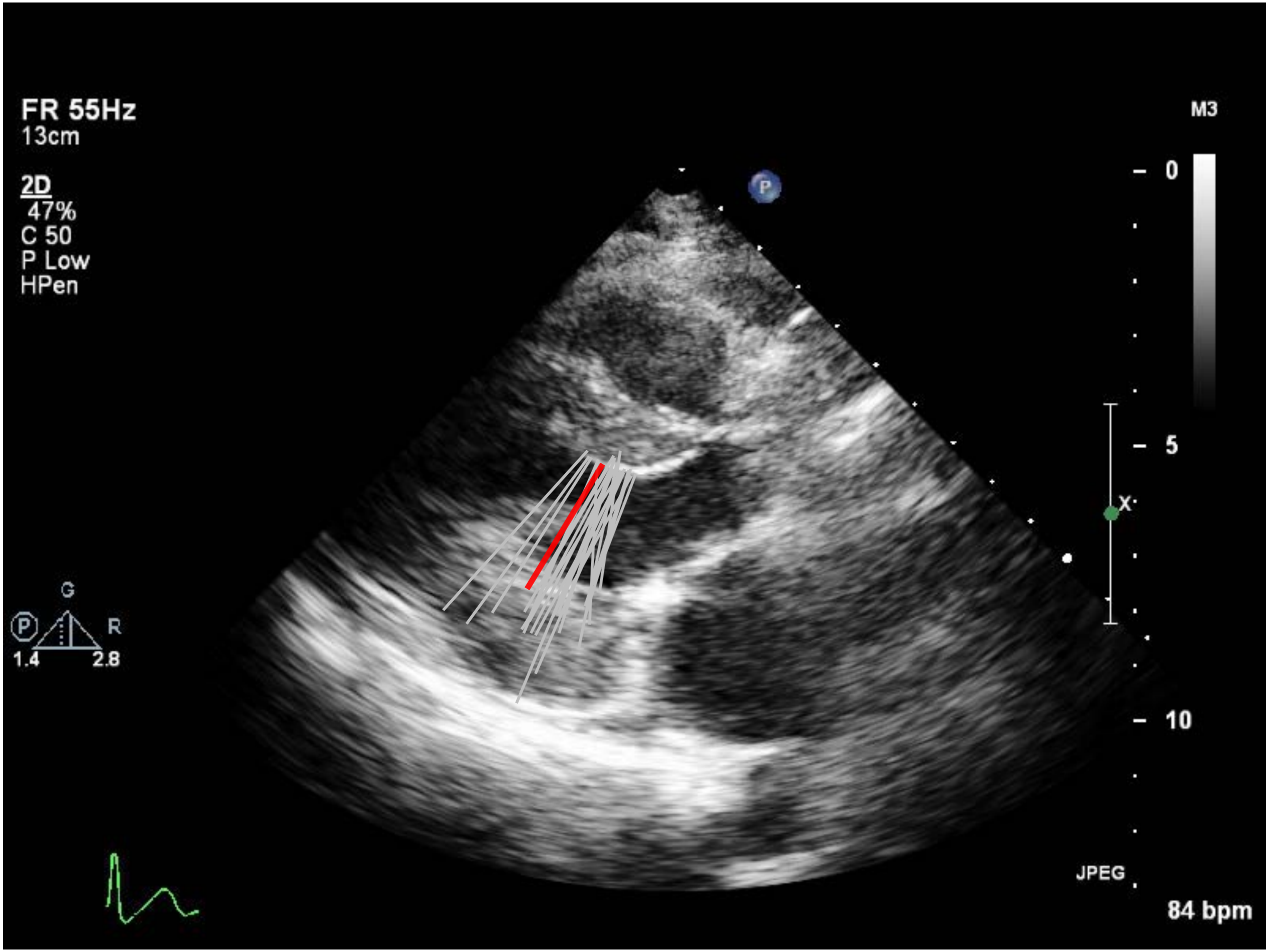

Pt 46 Systole

Absolute difference between AI and expert consensus: 5.3 mm

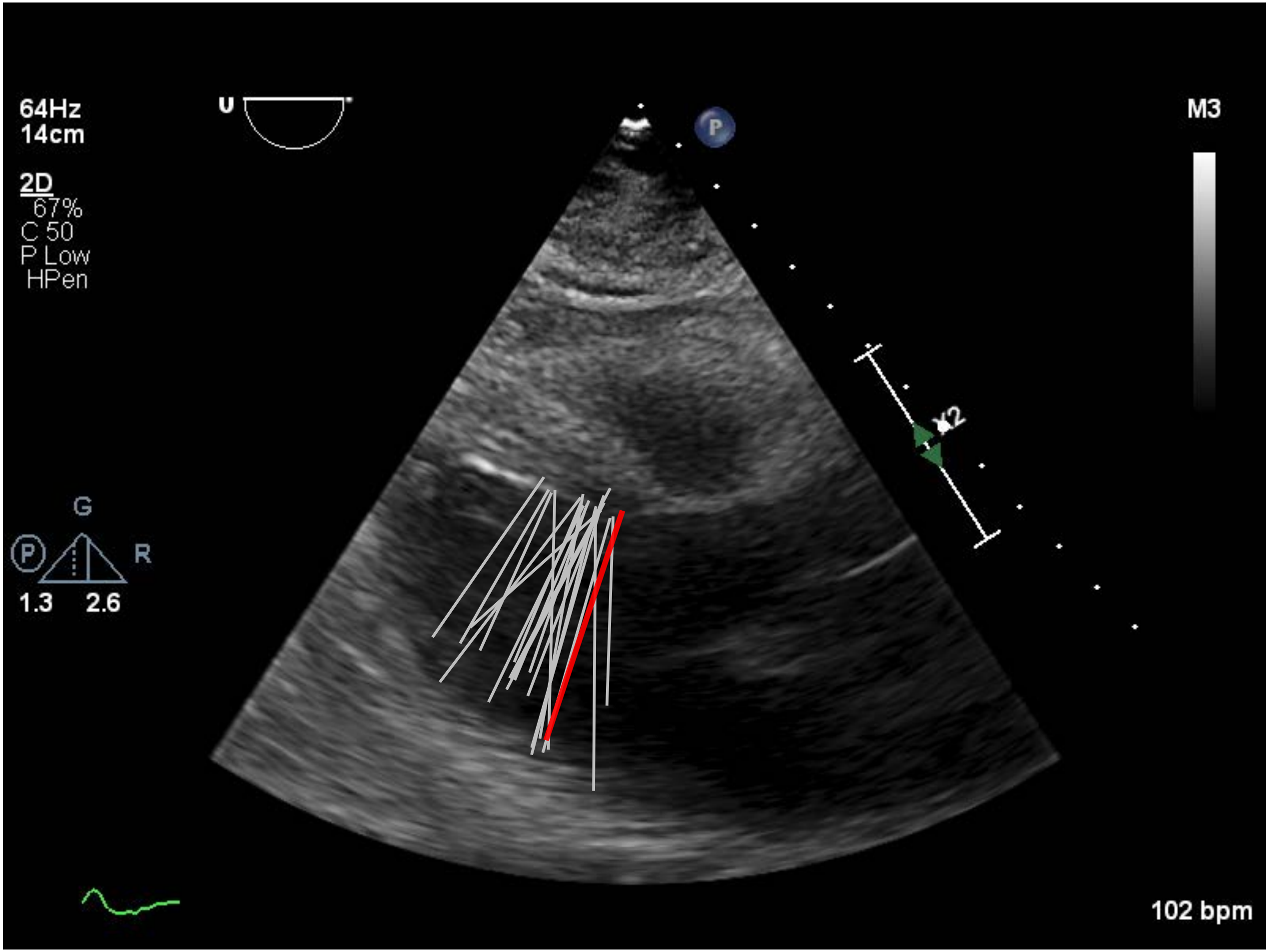

Pt 99 Systole

Absolute difference between AI and expert consensus: 5.7 mm

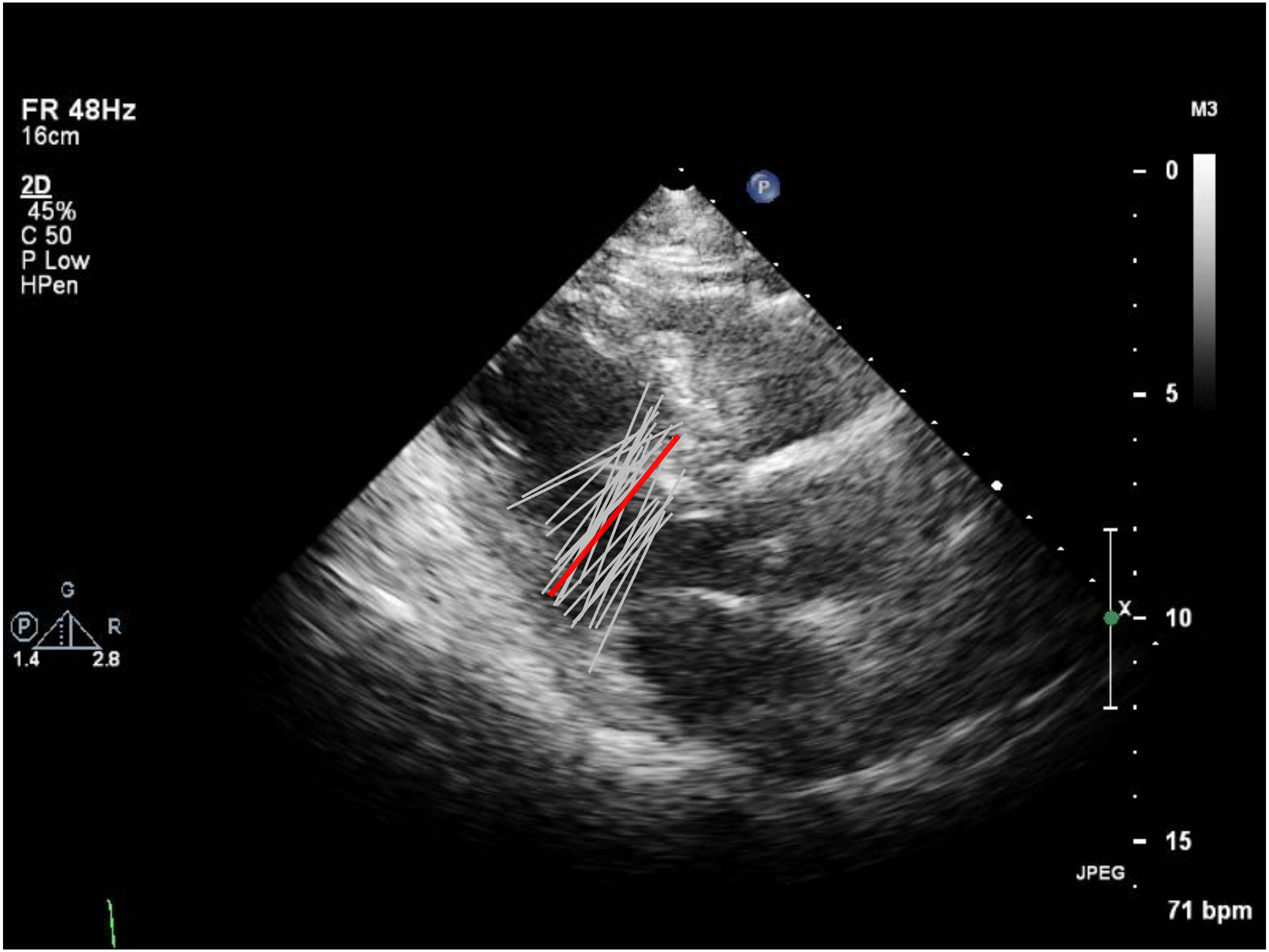

Pt 71 Diastole

Absolute difference between AI and expert consensus: 5.8 mm

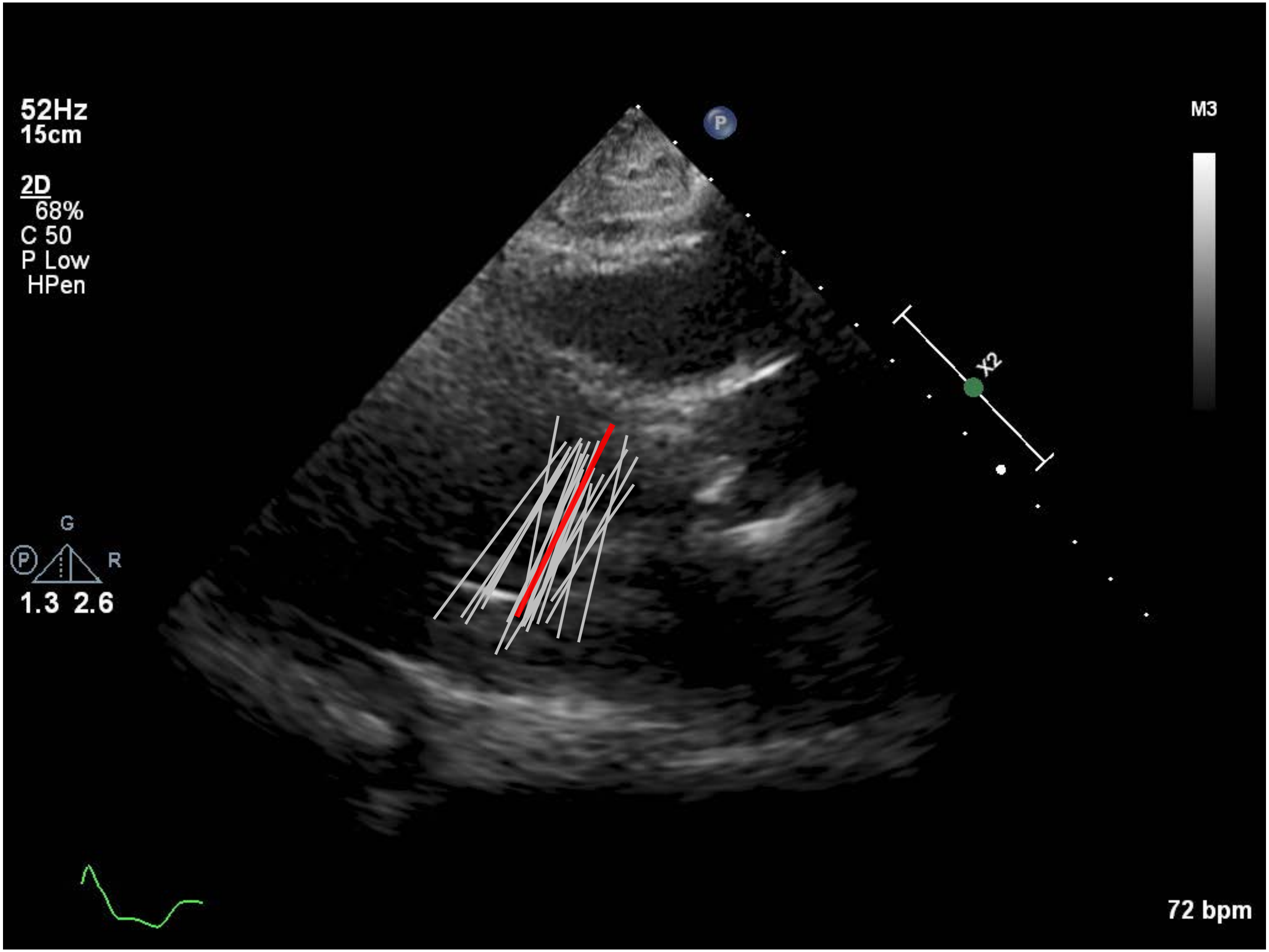

Pt 6 Systole
Absolute difference between AI and expert consensus: 5.9 mm

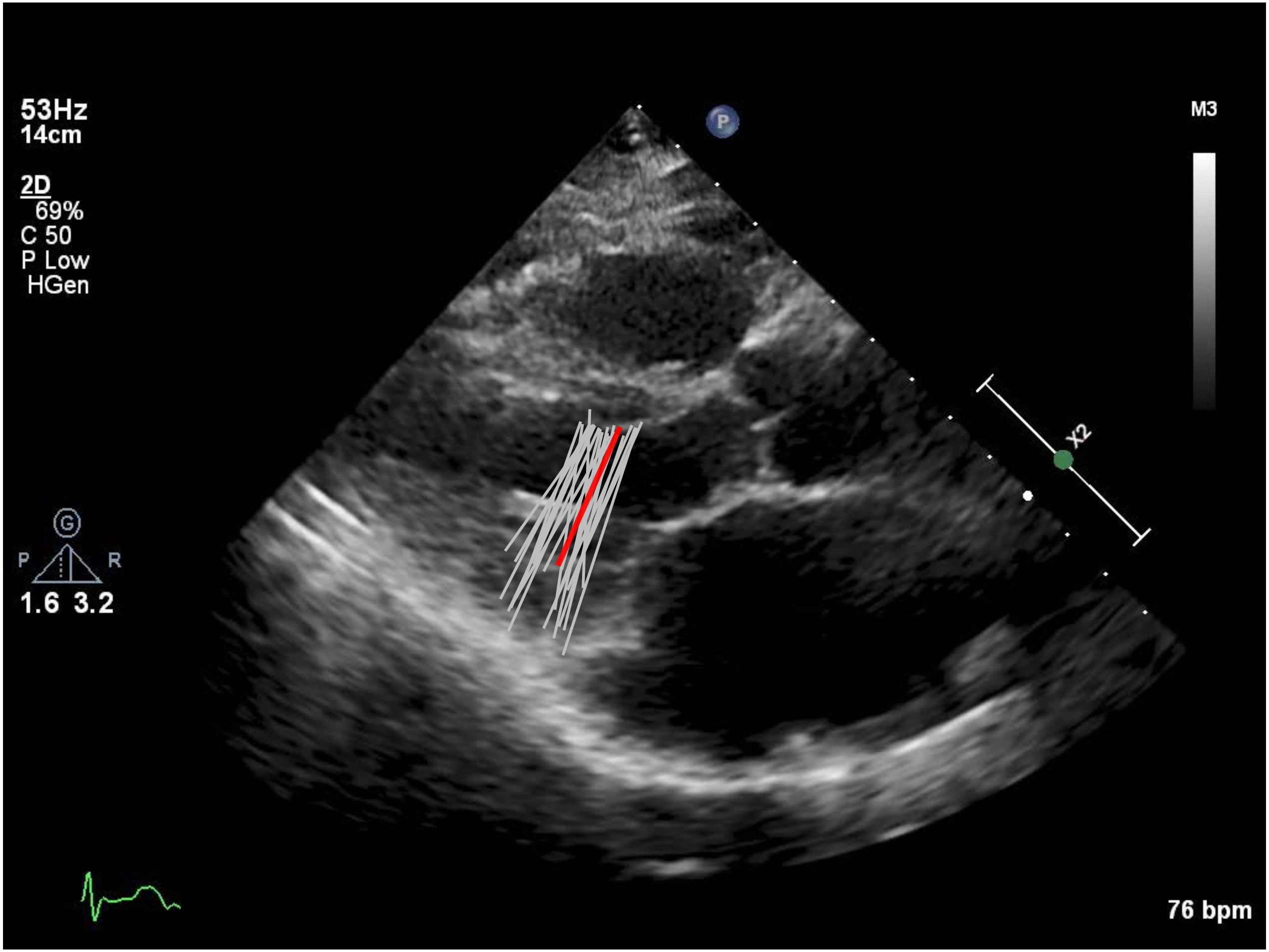

Pt 87 Systole
Absolute difference between AI and expert consensus: 6 mm

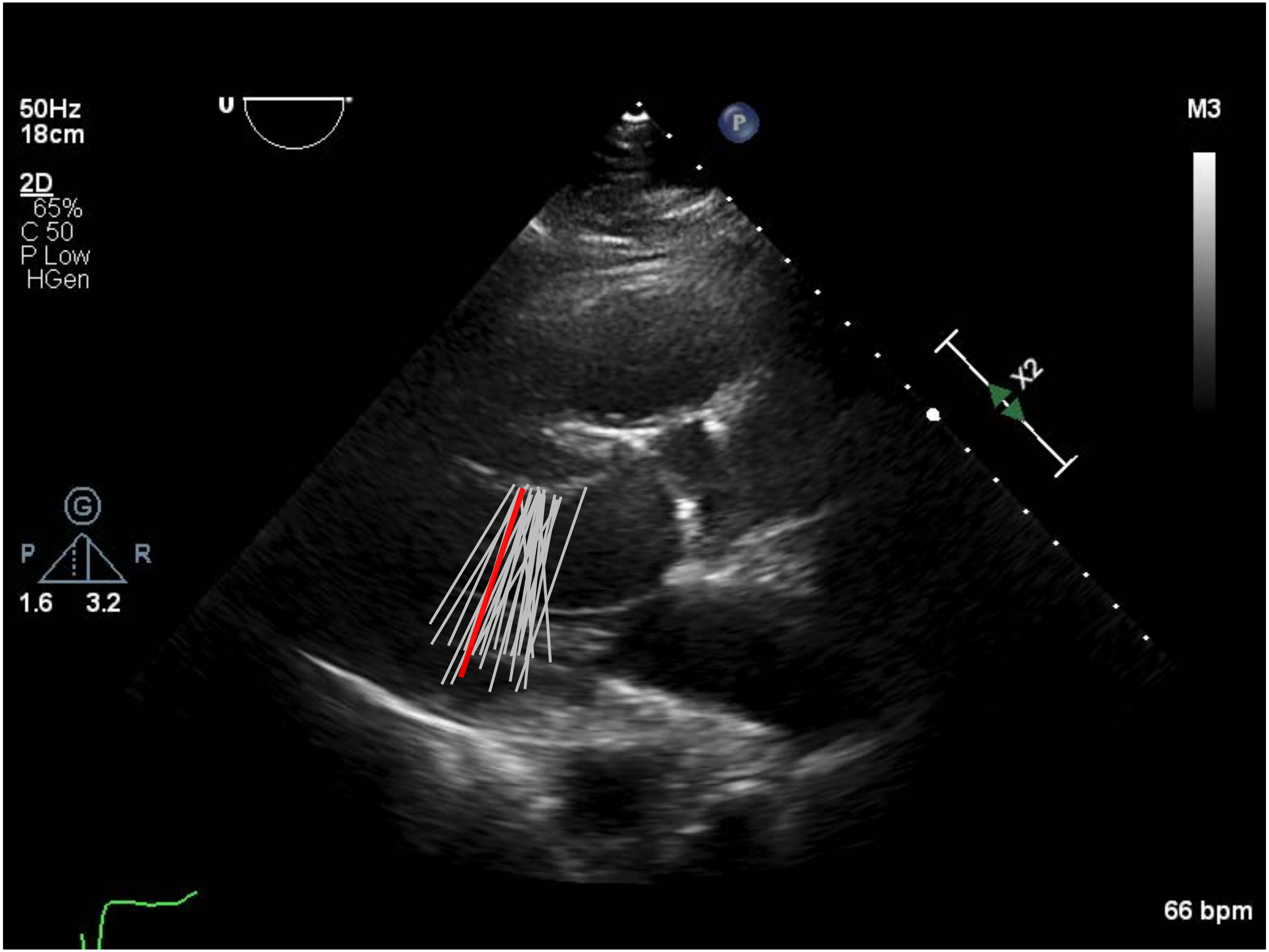

Pt 11 Systole

Absolute difference between AI and expert consensus: 6.1 mm

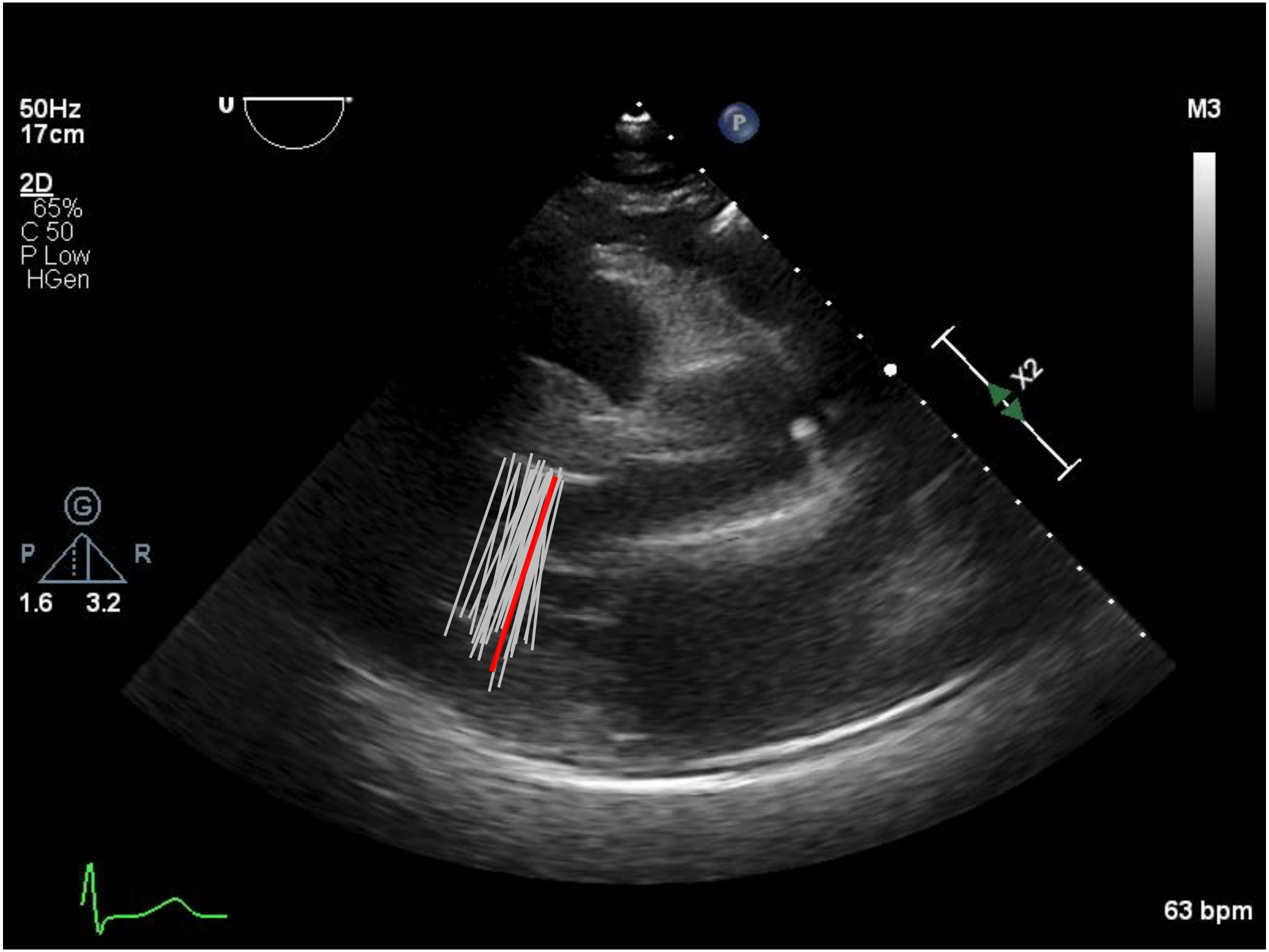

Pt 68 Systole

Absolute difference between AI and expert consensus: 6.3 mm

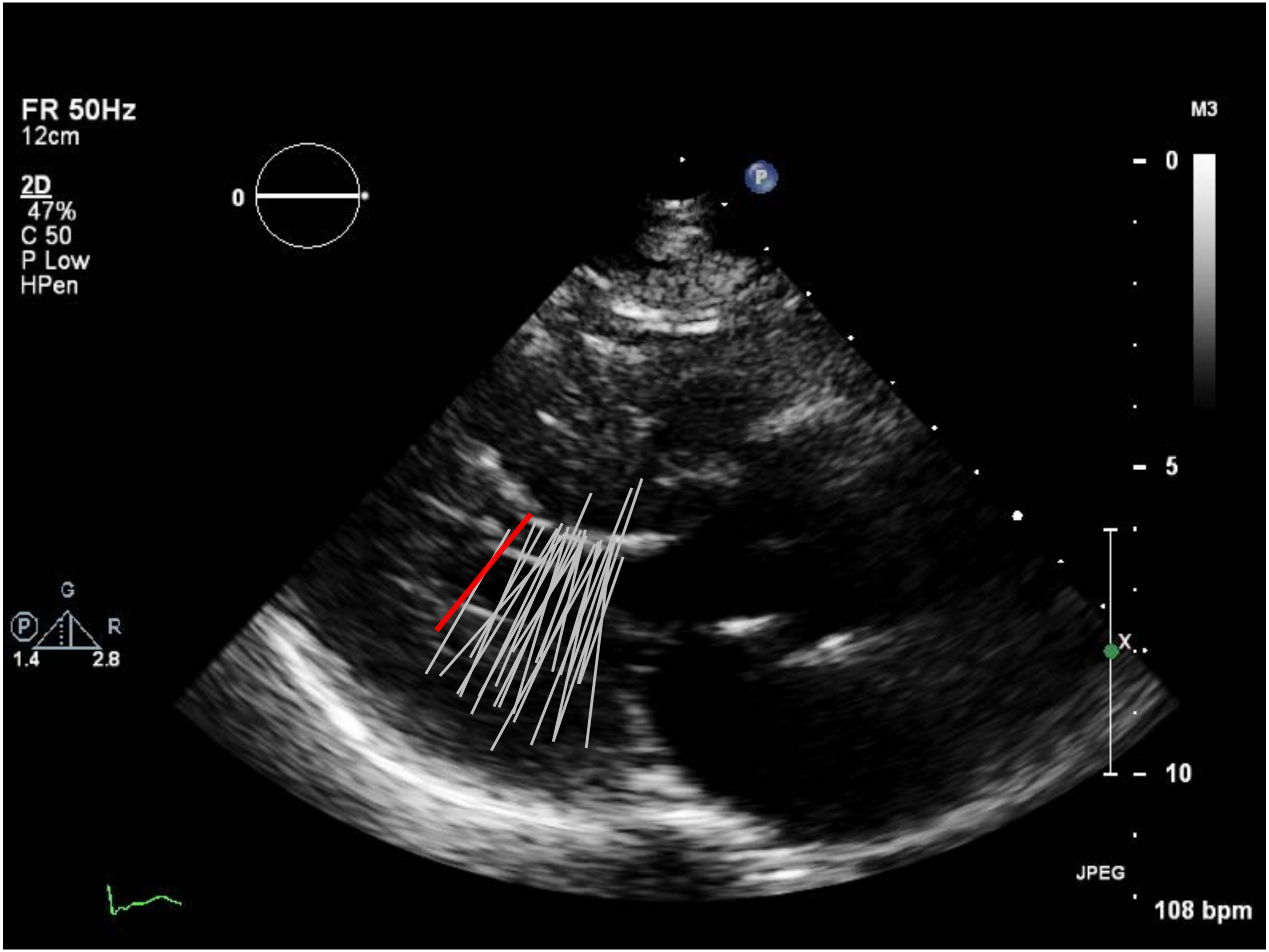

Pt 89 Systole

Absolute difference between AI and expert consensus: 6.8 mm

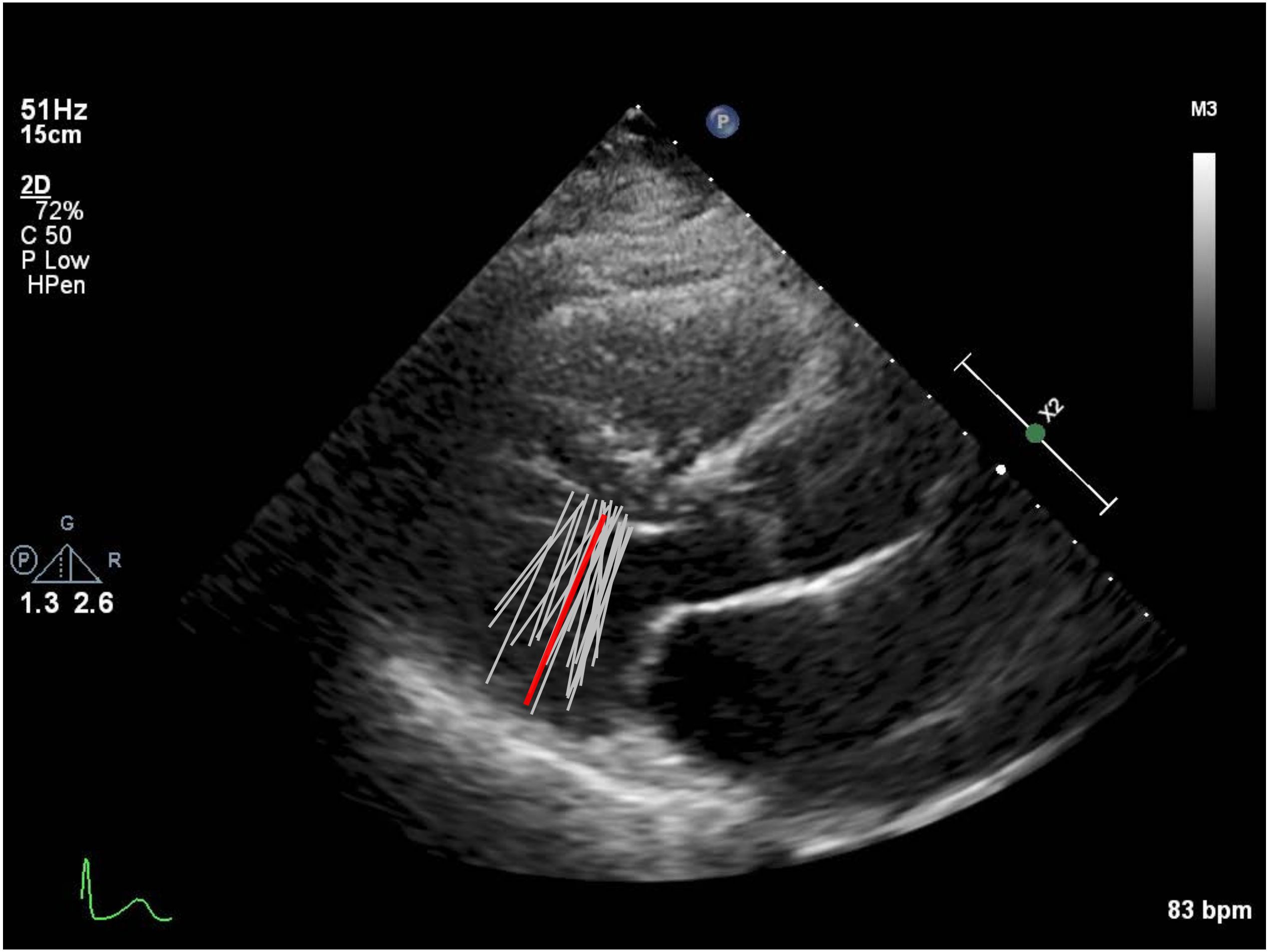

Pt 63 Systole
Absolute difference between AI and expert consensus: 6.8 mm

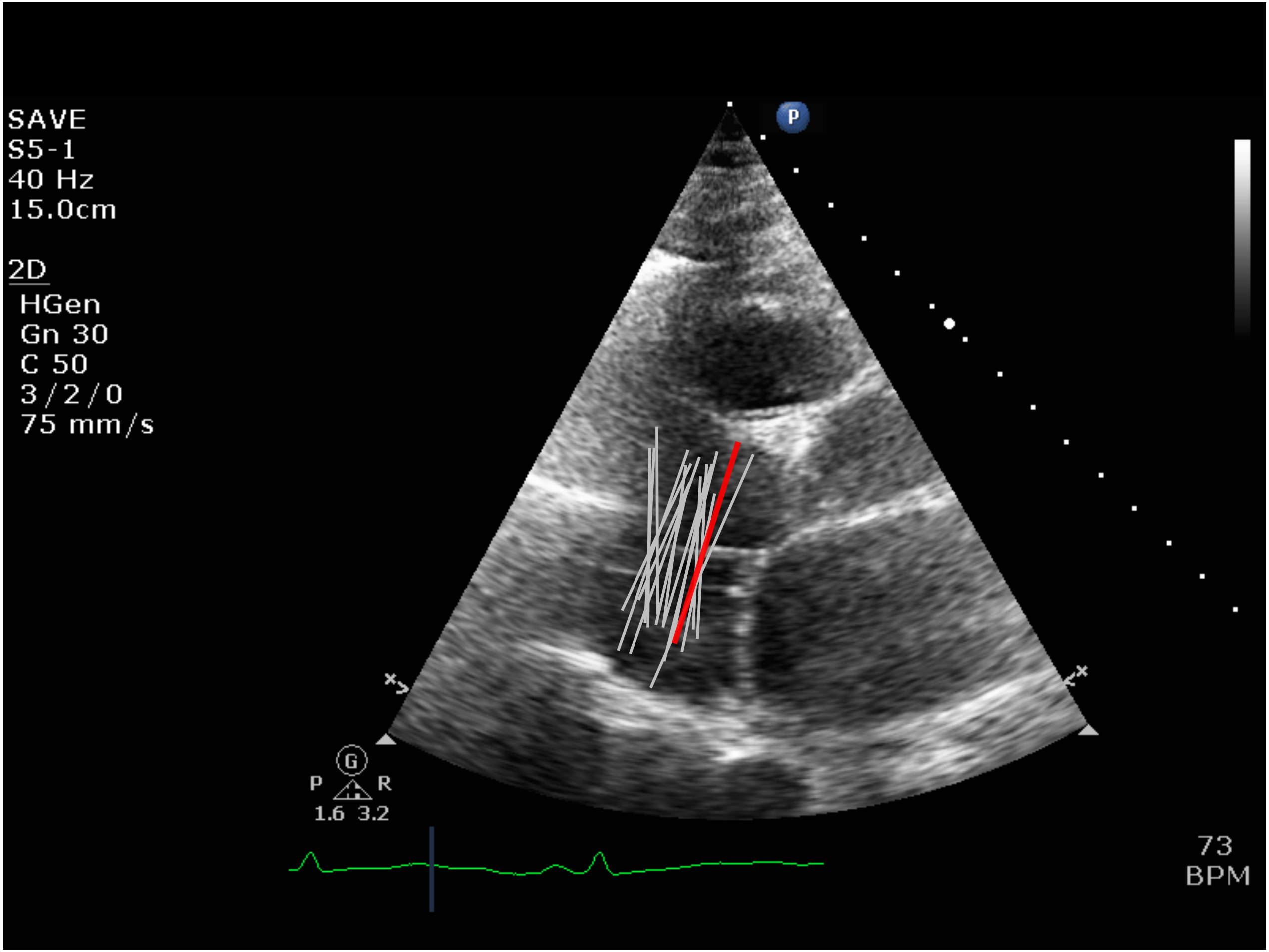

Pt 14 Systole

Absolute difference between AI and expert consensus: 7 mm

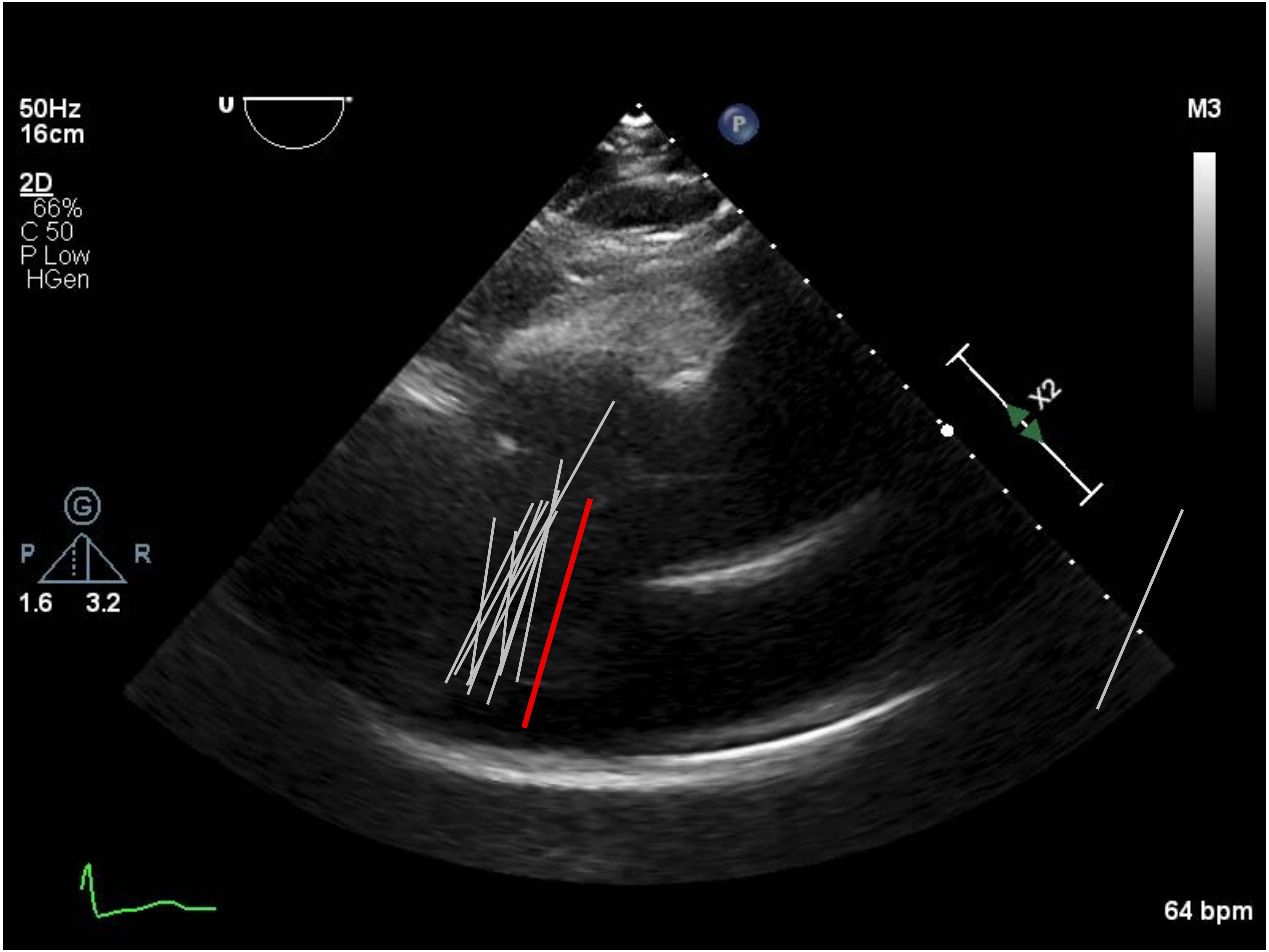

Pt 20 Diastole

Absolute difference between AI and expert consensus: 7.4 mm

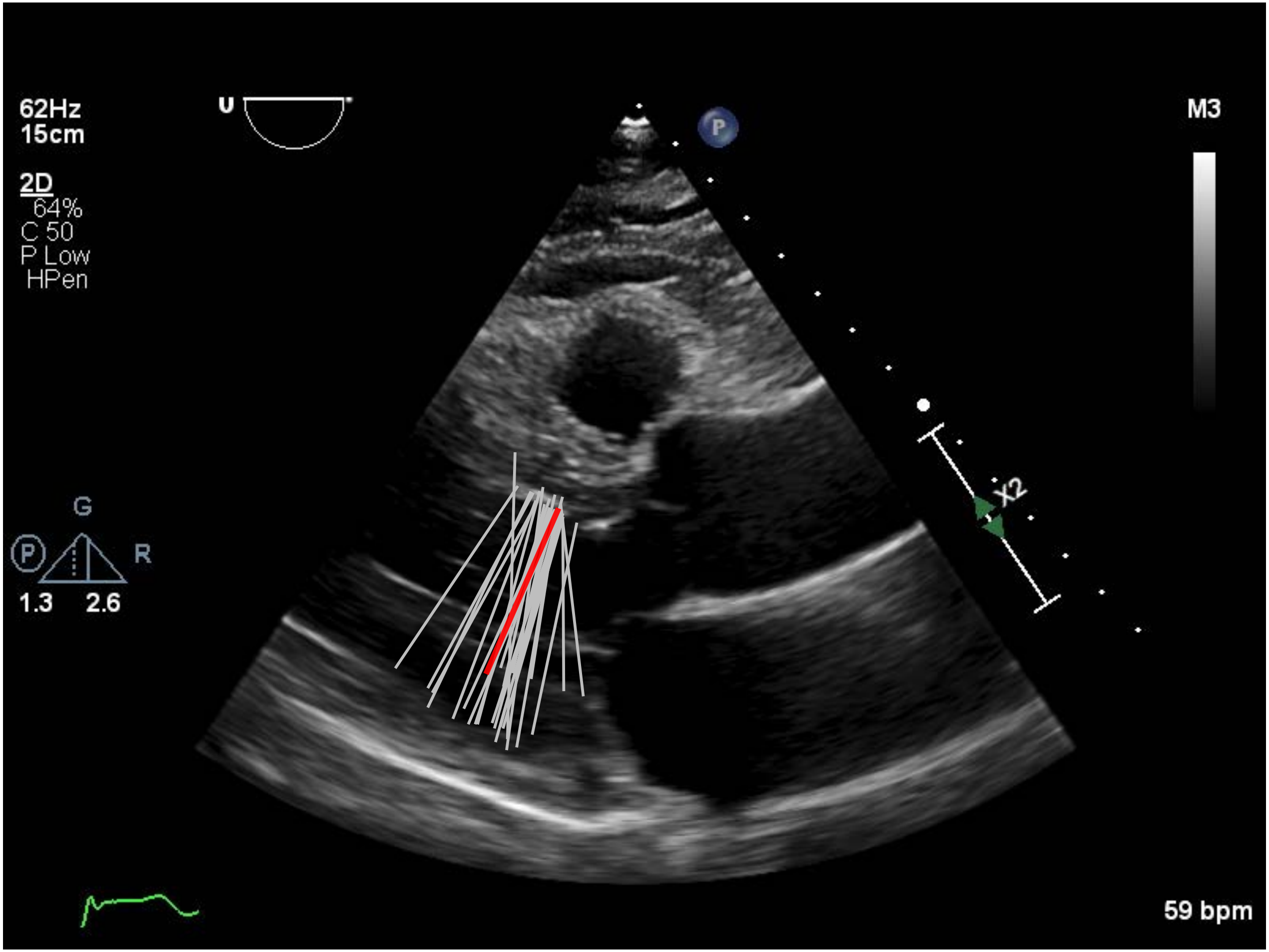

Pt 60 Systole

Absolute difference between AI and expert consensus: 8 mm

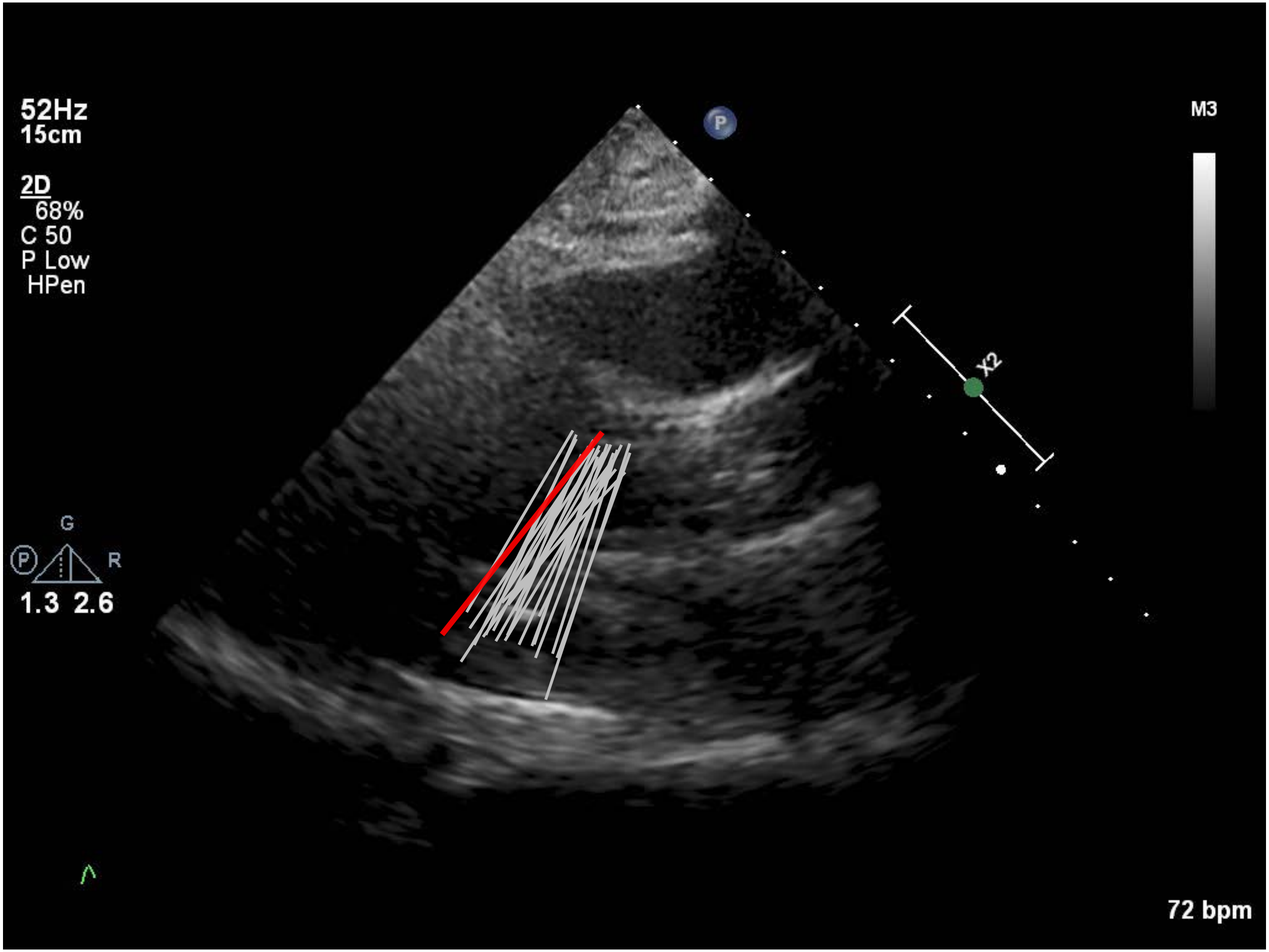

Pt 6 Diastole
Absolute difference between AI and expert consensus: 8.5 mm

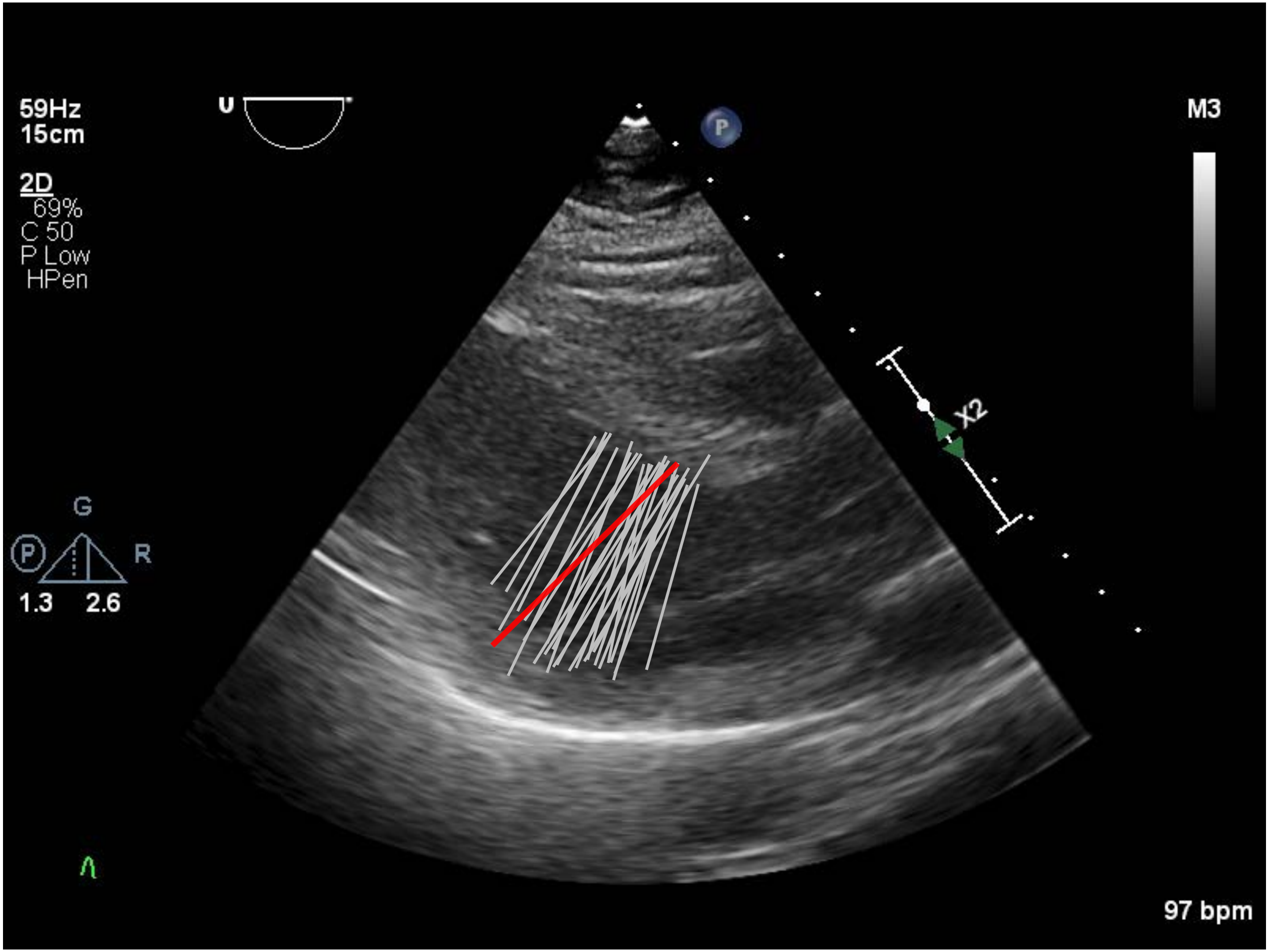

Pt 39 Diastole

Absolute difference between AI and expert consensus: 8.7 mm

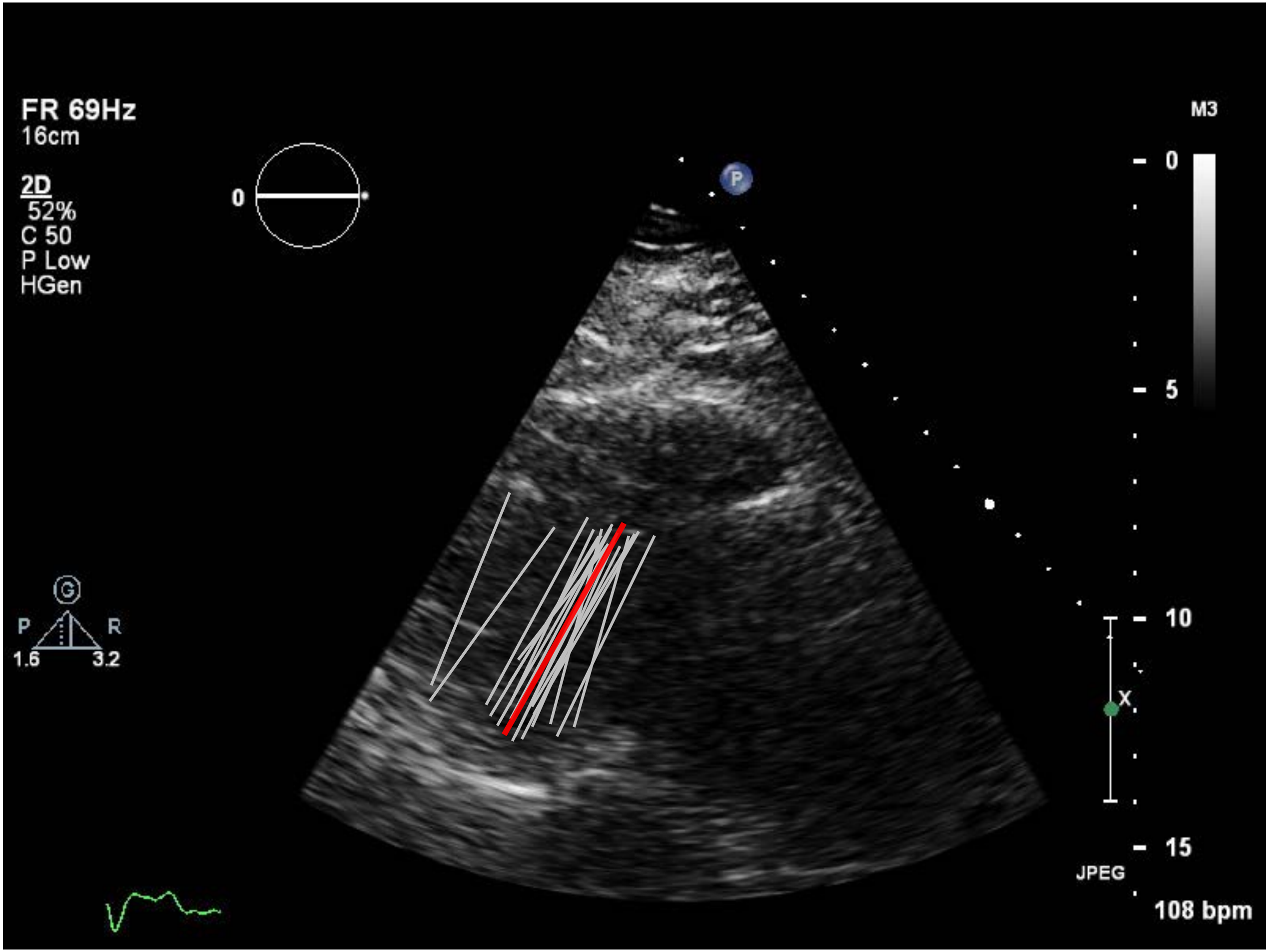

Pt 66 Systole

Absolute difference between AI and expert consensus: 8.8 mm

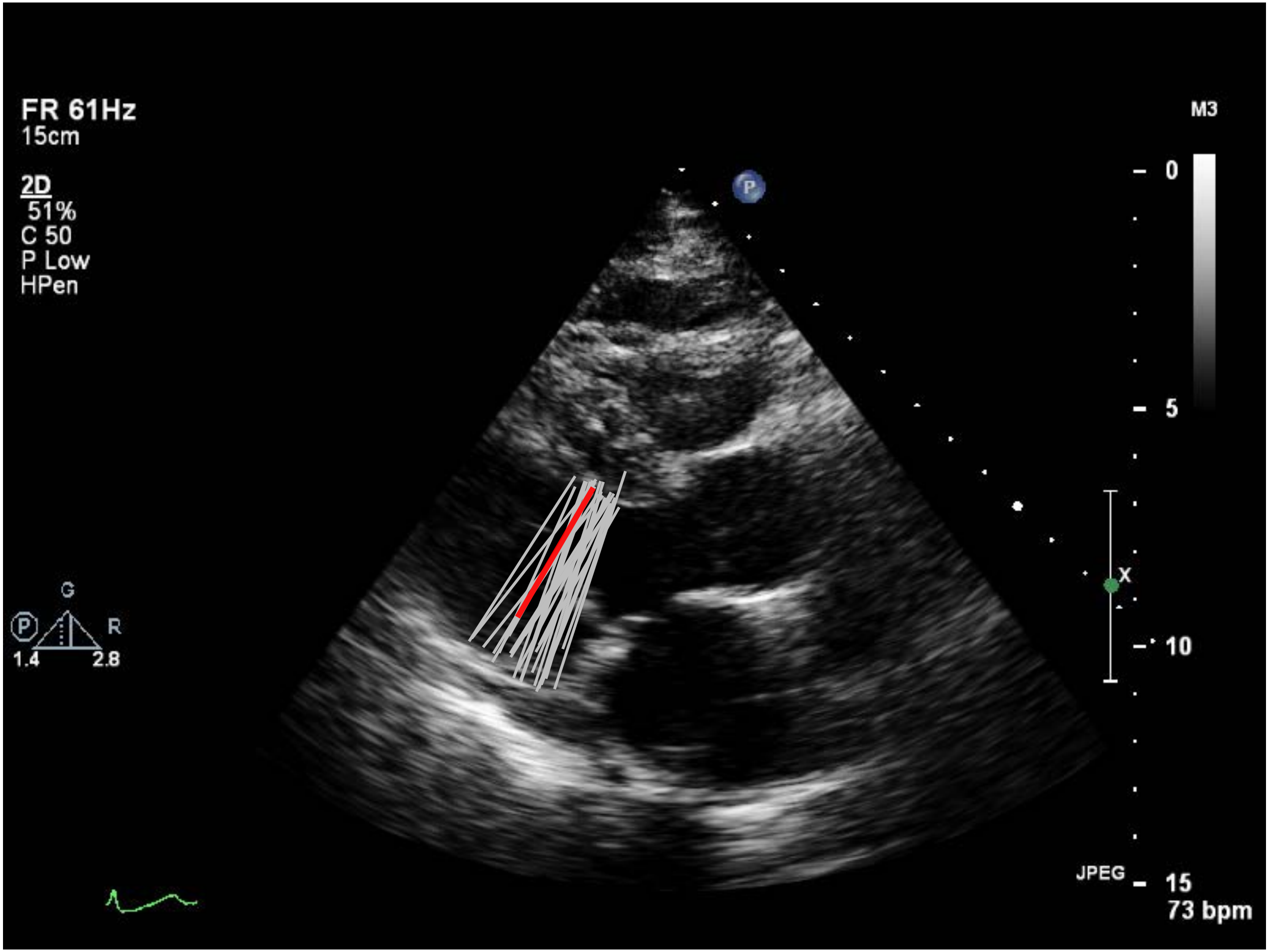

Pt 93 Systole

Absolute difference between AI and expert consensus: 9 mm

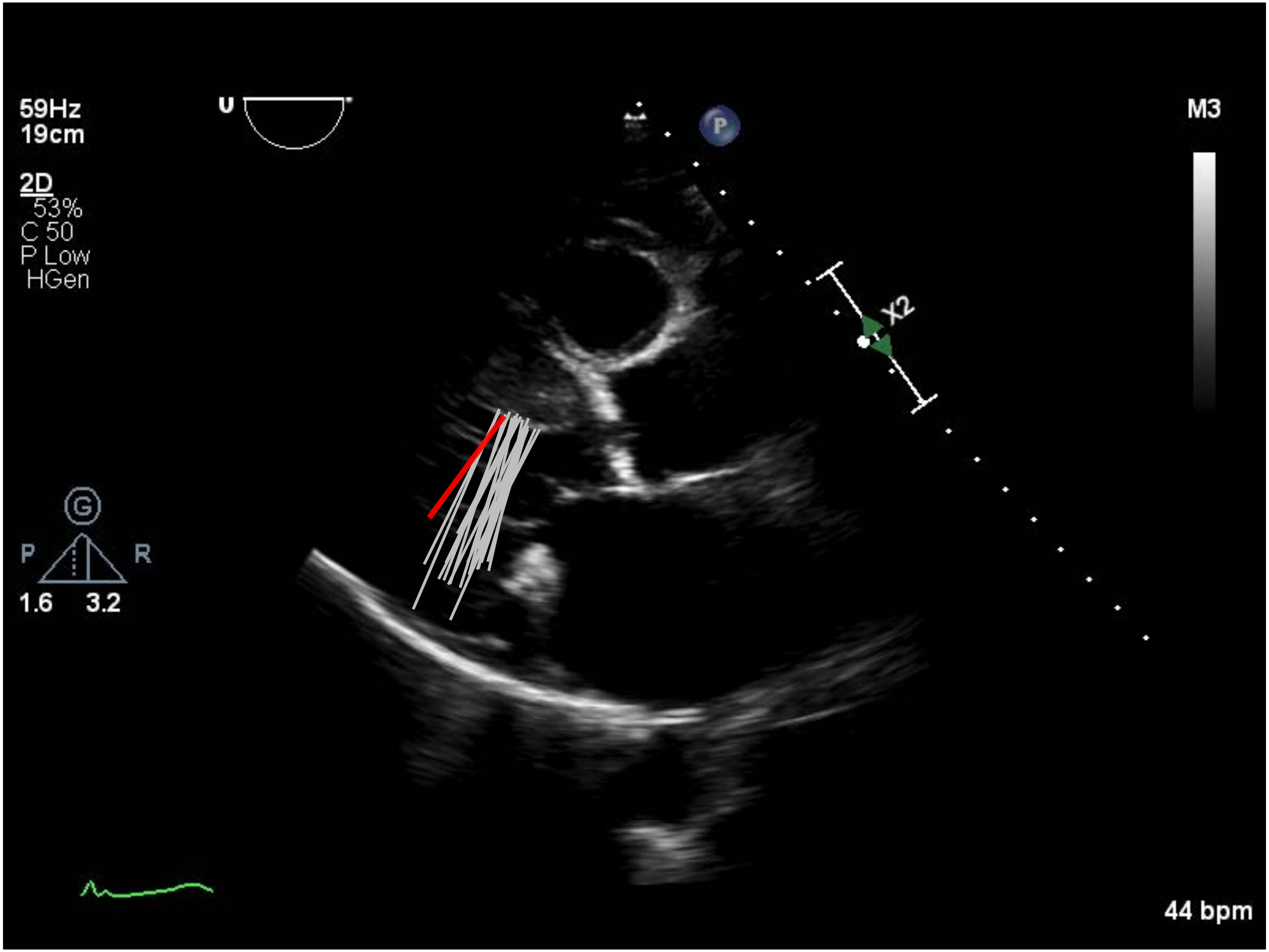

Pt 13 Systole

Absolute difference between AI and expert consensus: 9 mm

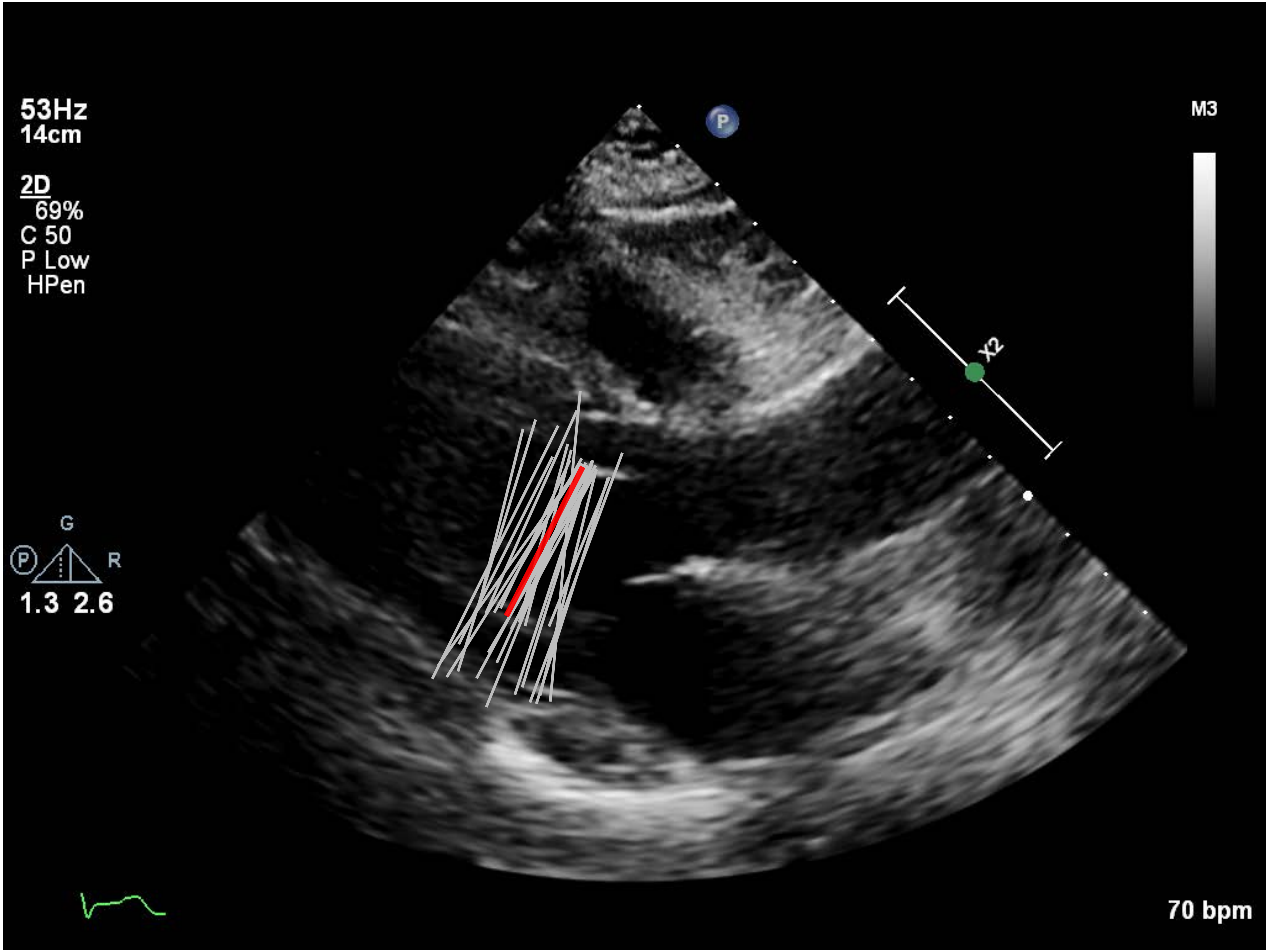

Pt 83 Systole
Absolute difference between AI and expert consensus: 9.2 mm

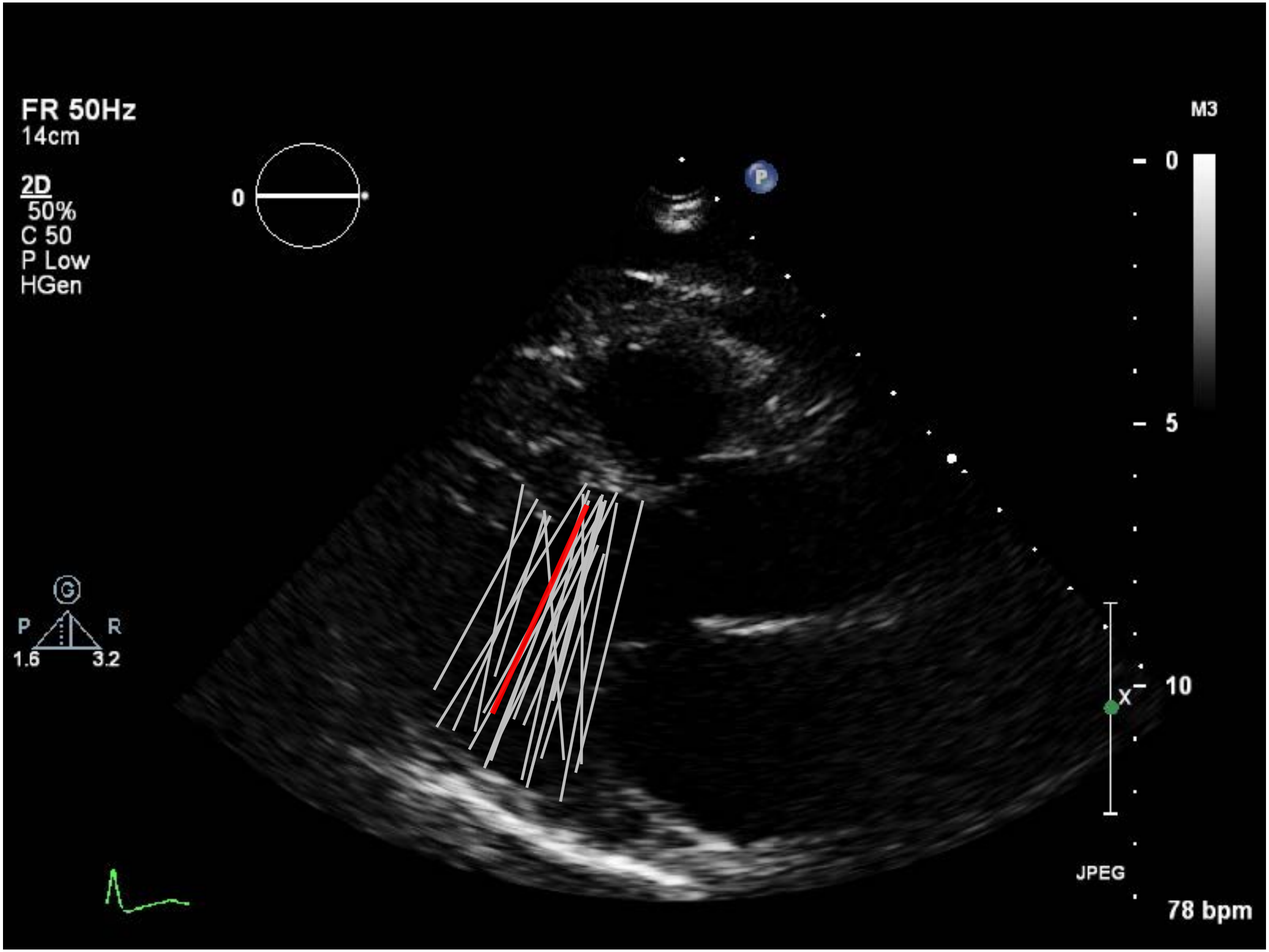

Pt 50 Systole

Absolute difference between AI and expert consensus: 9.2 mm

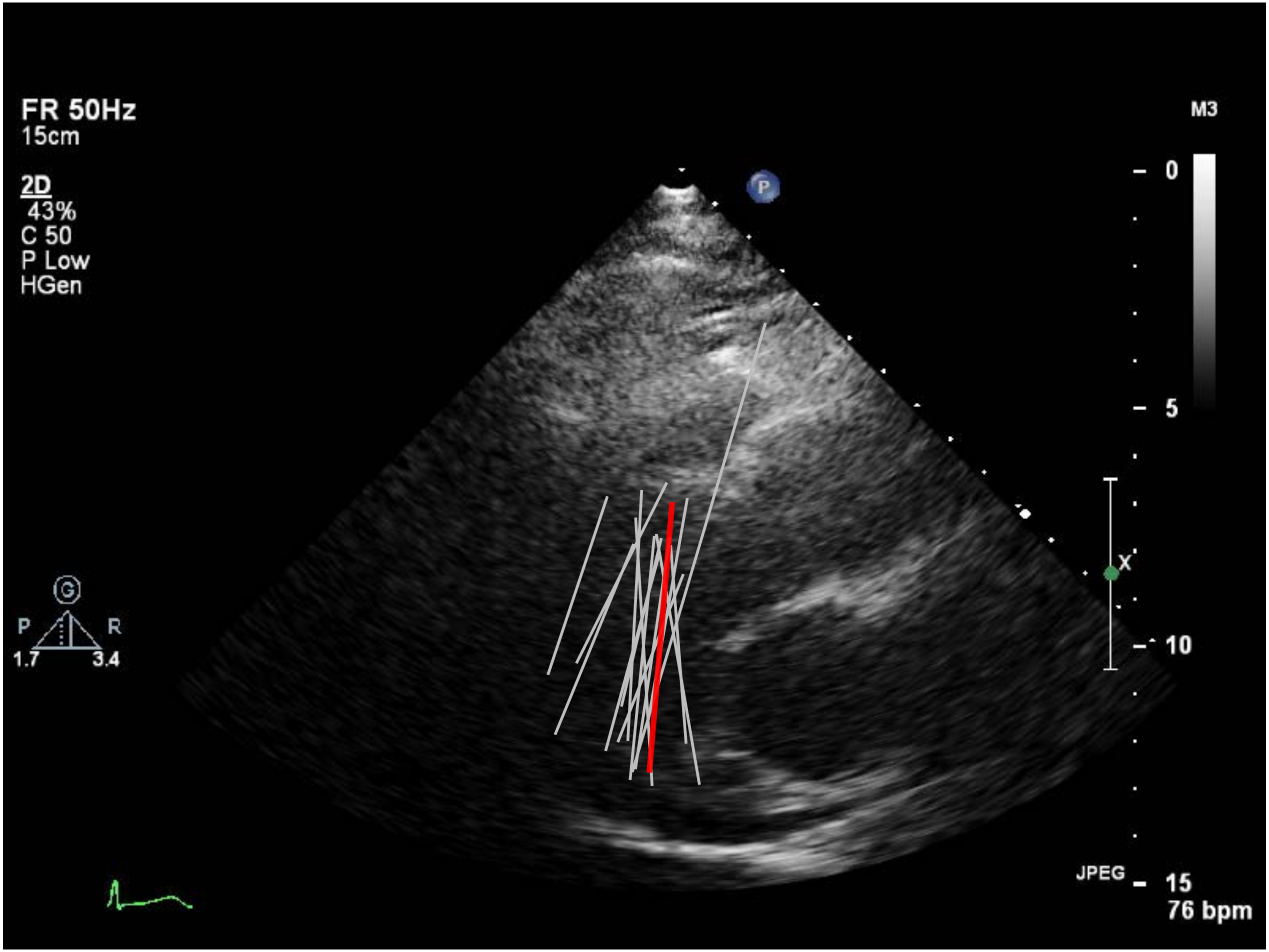

Pt 1 Systole

Absolute difference between AI and expert consensus: 9.4 mm

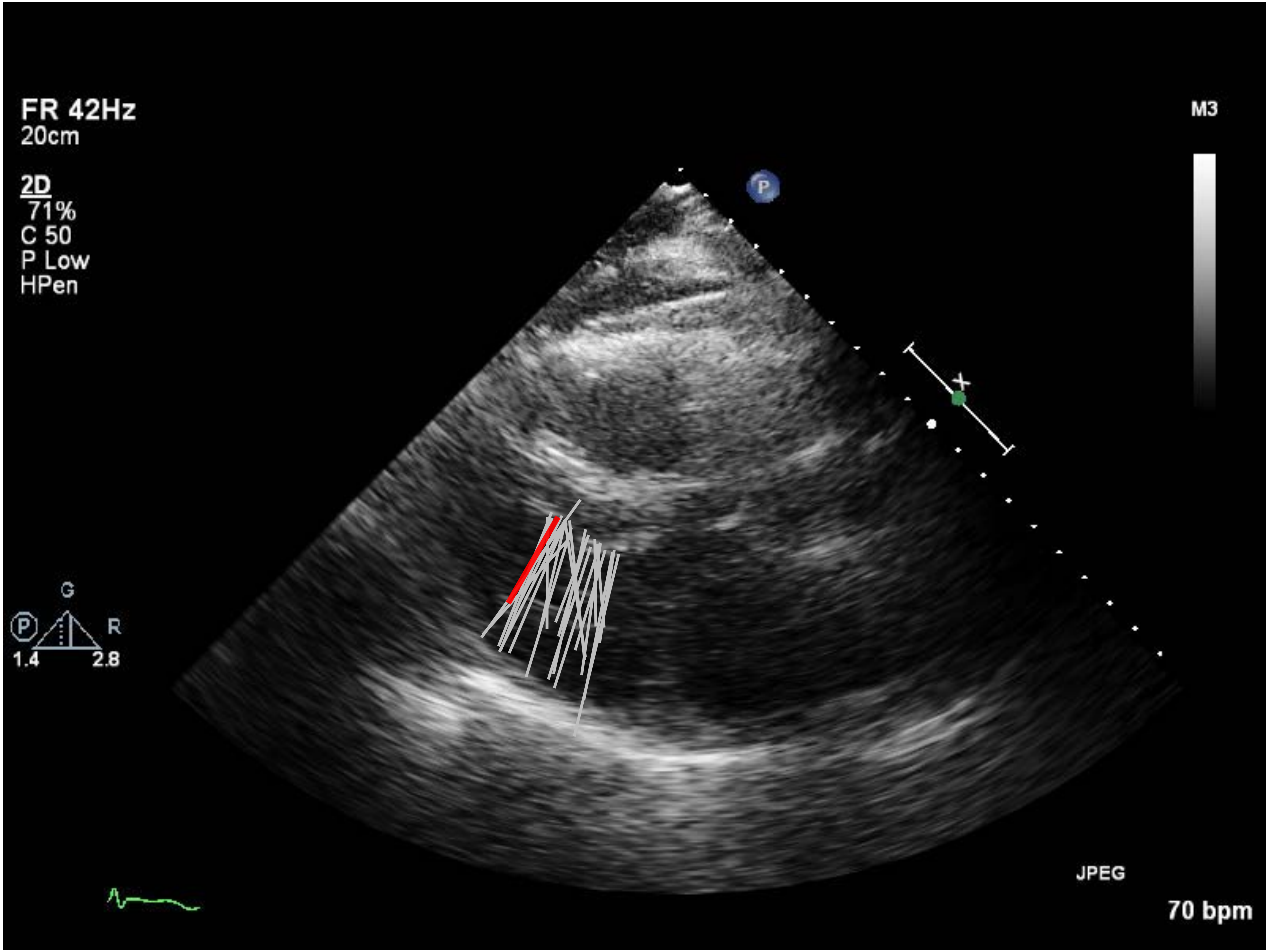

Pt 96 Systole

Absolute difference between AI and expert consensus: 9.5 mm

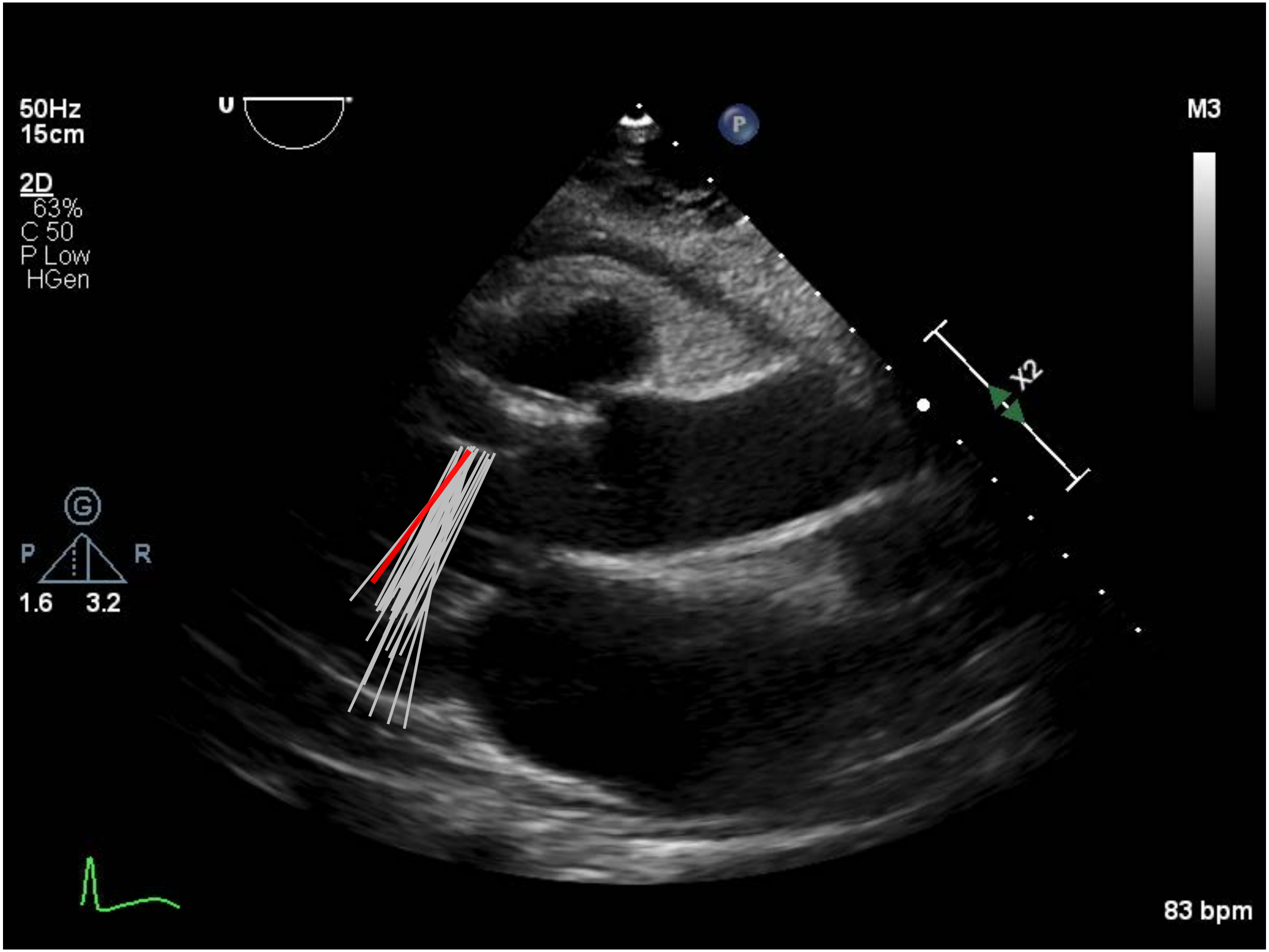

Pt 4 Systole

Absolute difference between AI and expert consensus: 9.7 mm

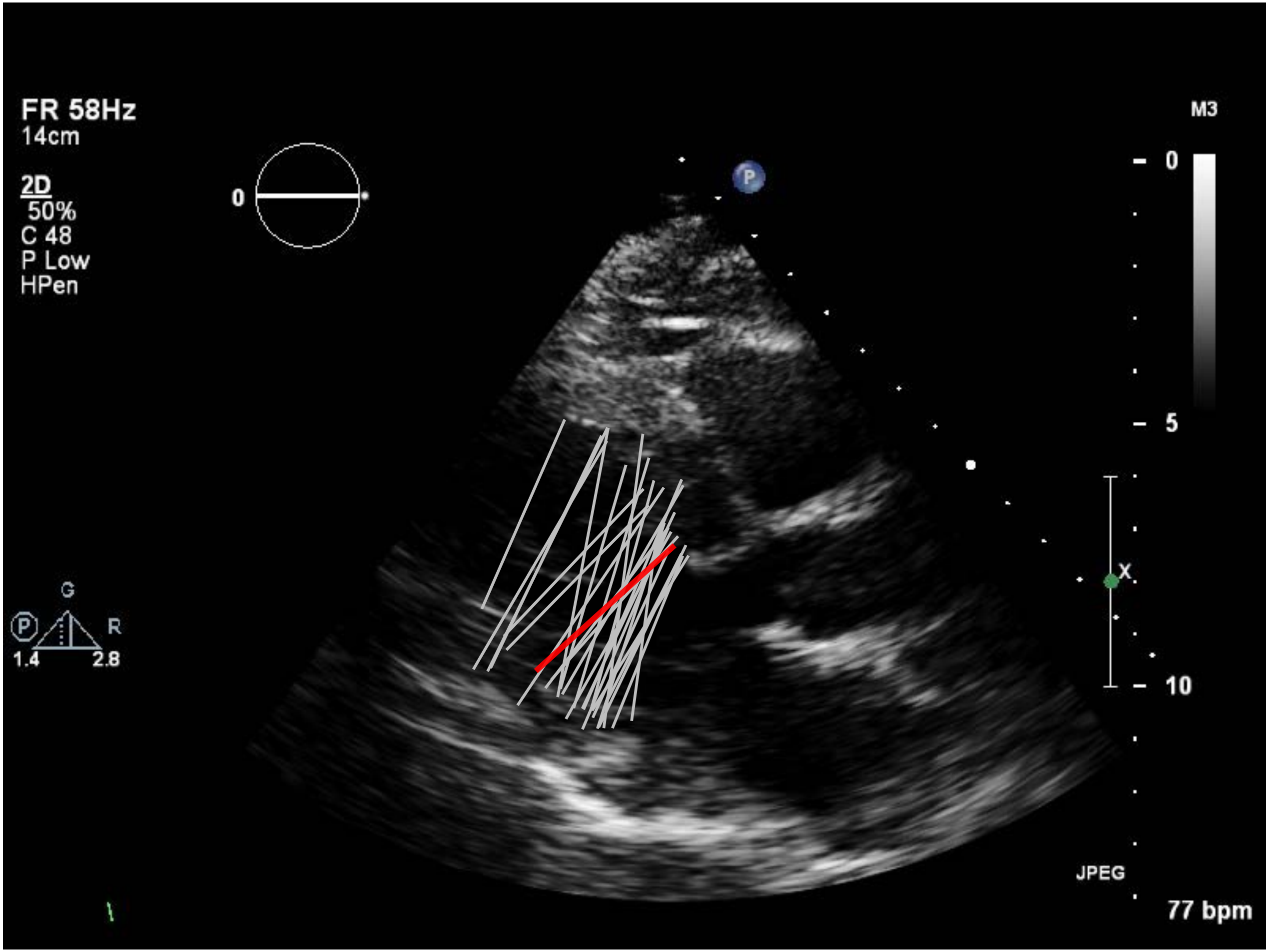

Pt 3 Diastole

Absolute difference between AI and expert consensus: 9.8 mm

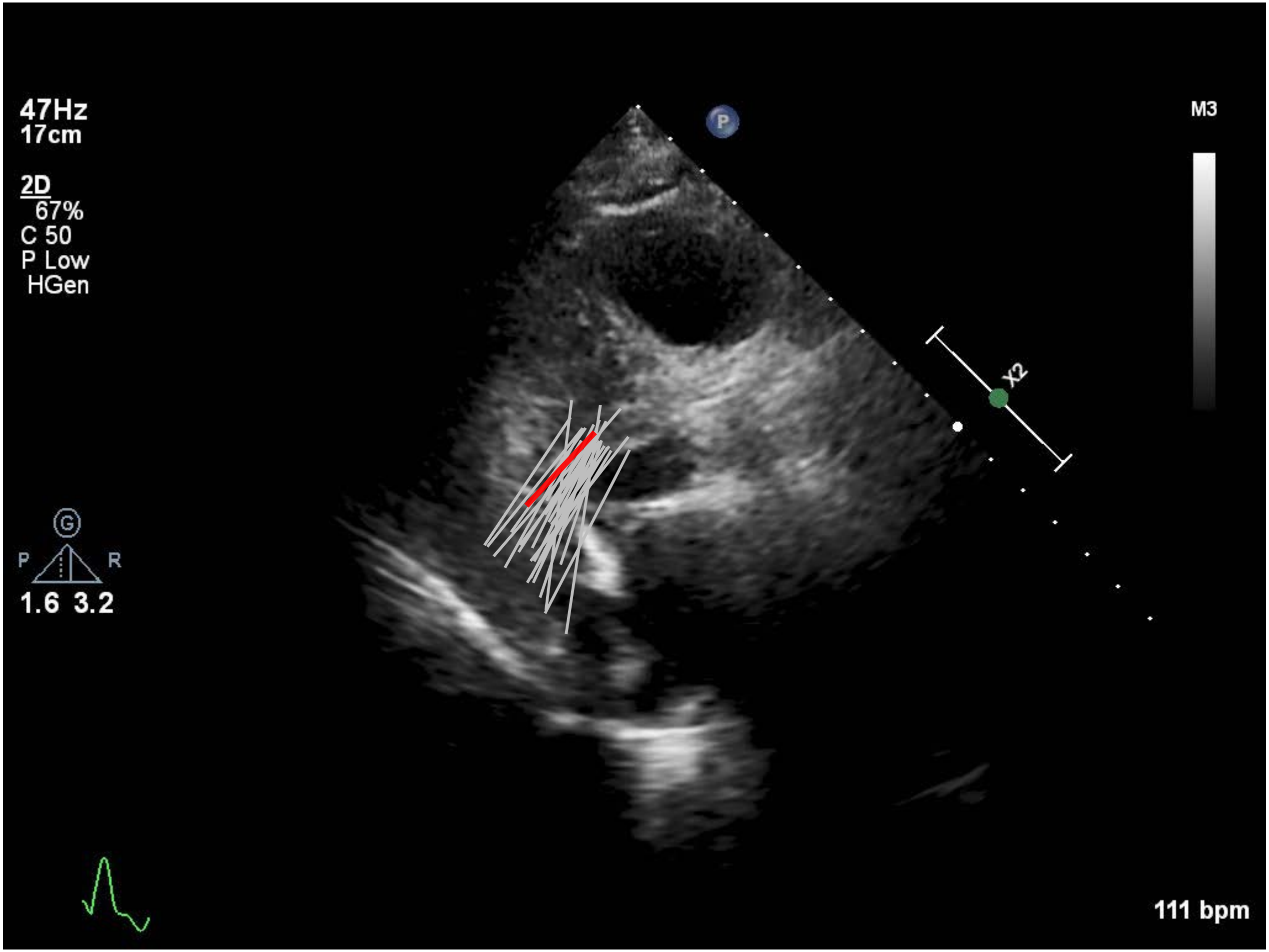

Pt 65 Systole
Absolute difference between AI and expert consensus: 10 mm

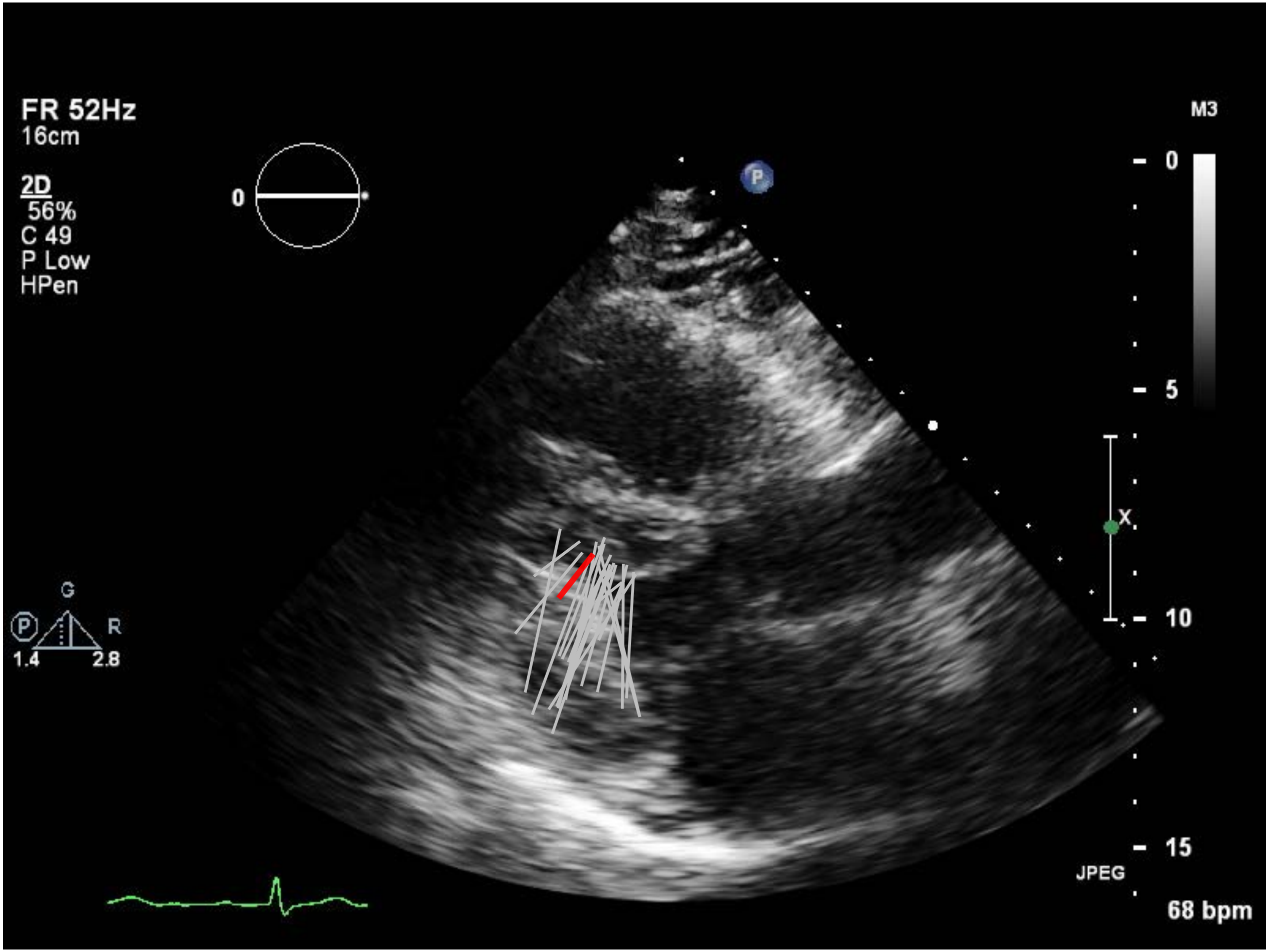

Pt 37 Systole

Absolute difference between AI and expert consensus: 16 mm

Supplemental Figure VII: Full disclosure IVSd measurement dataset

The following 200 images show the AI (red) and expert (grey) interventricular septum distance measurements (IVSd) on the end-diastolic and end-systolic frames of the 100 validation images. The images are ordered by the absolute difference between AI and expert consensus LVID measurement.

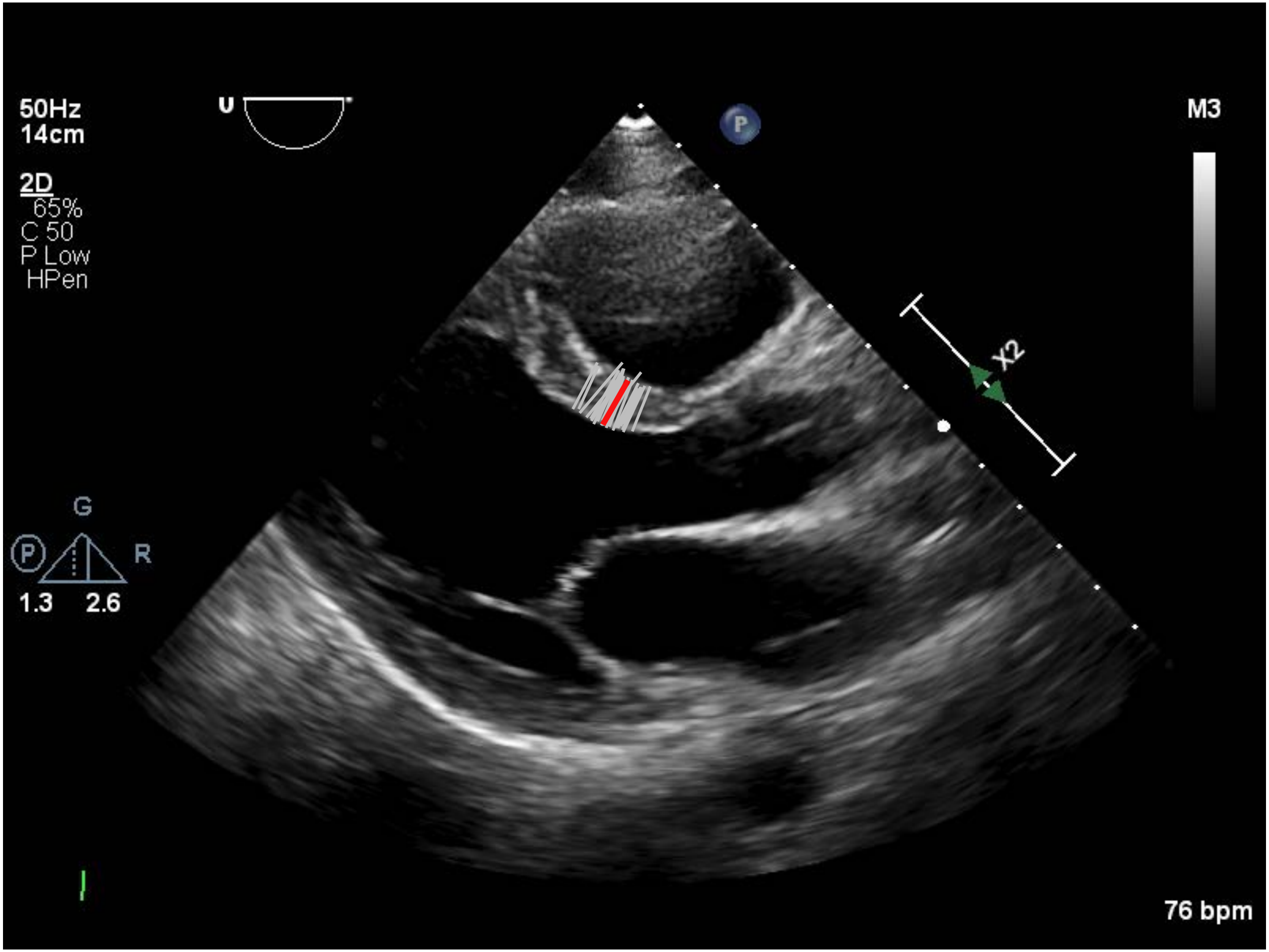

Pt 25 Diastole

Absolute difference between AI and expert consensus: 0.0023 mm

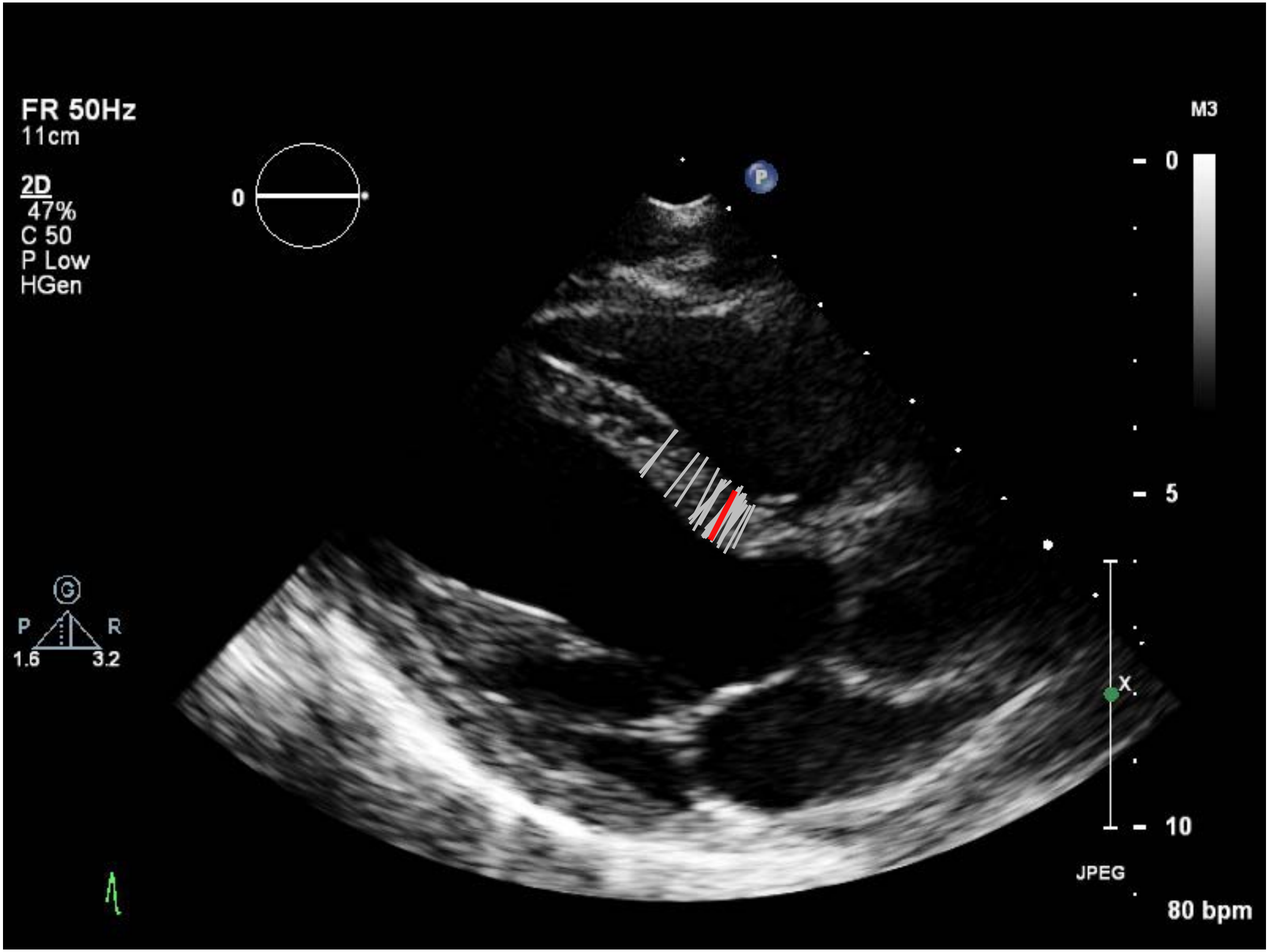

Pt 17 Diastole

Absolute difference between AI and expert consensus: 0.03 mm

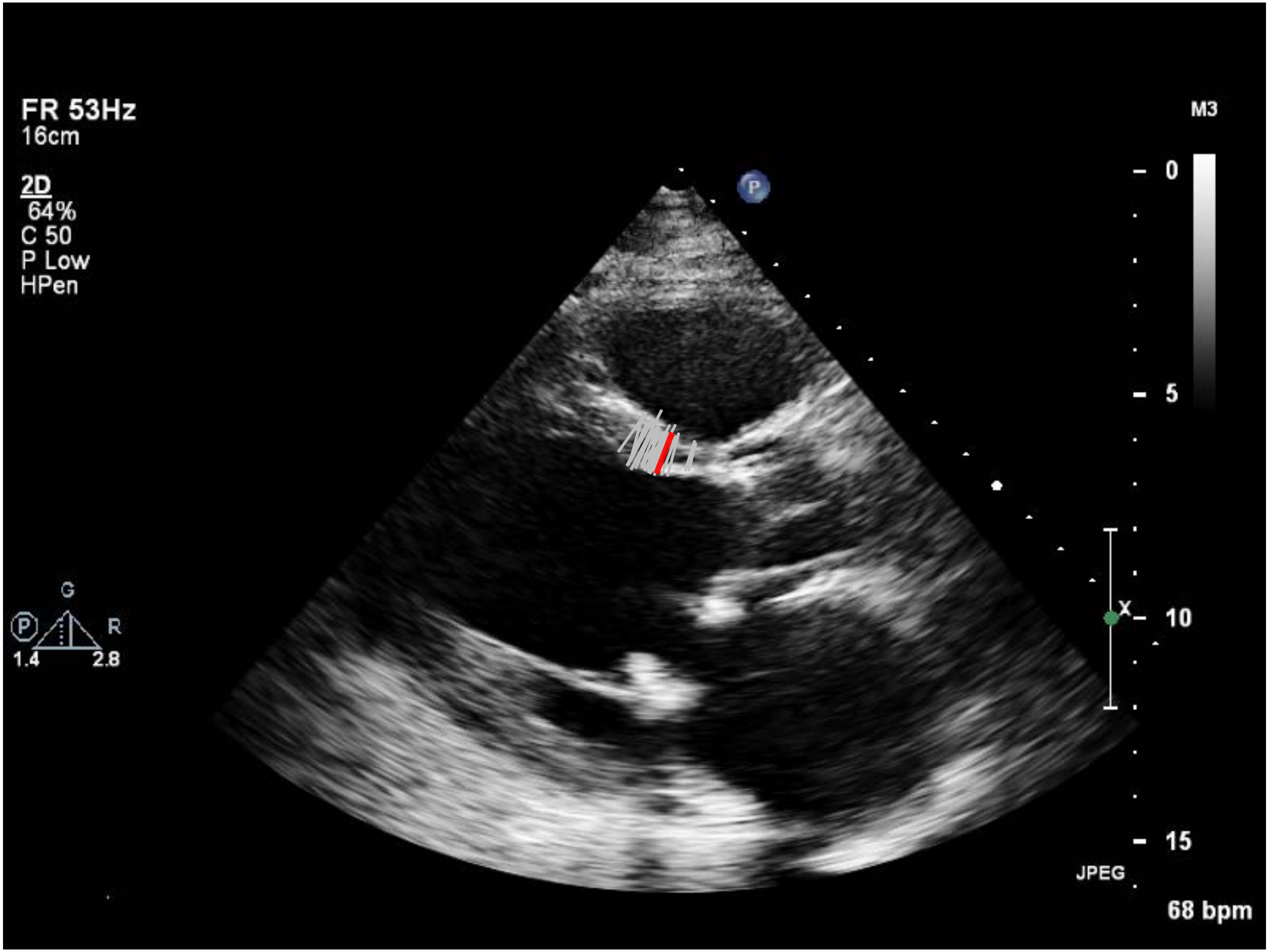

Pt 56 Diastole

Absolute difference between AI and expert consensus: 0.03 mm

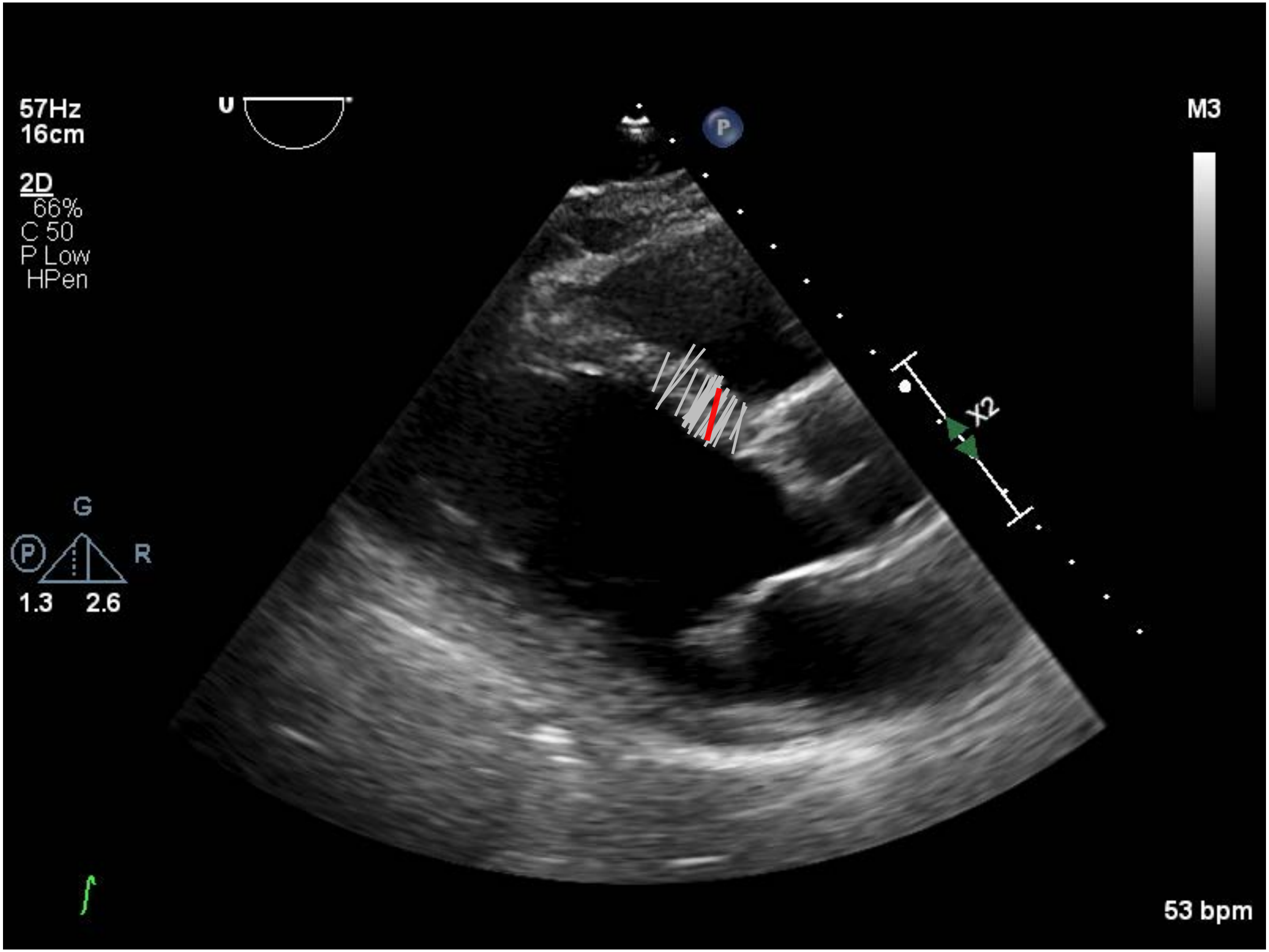

Pt 7 Diastole

Absolute difference between AI and expert consensus: 0.04 mm

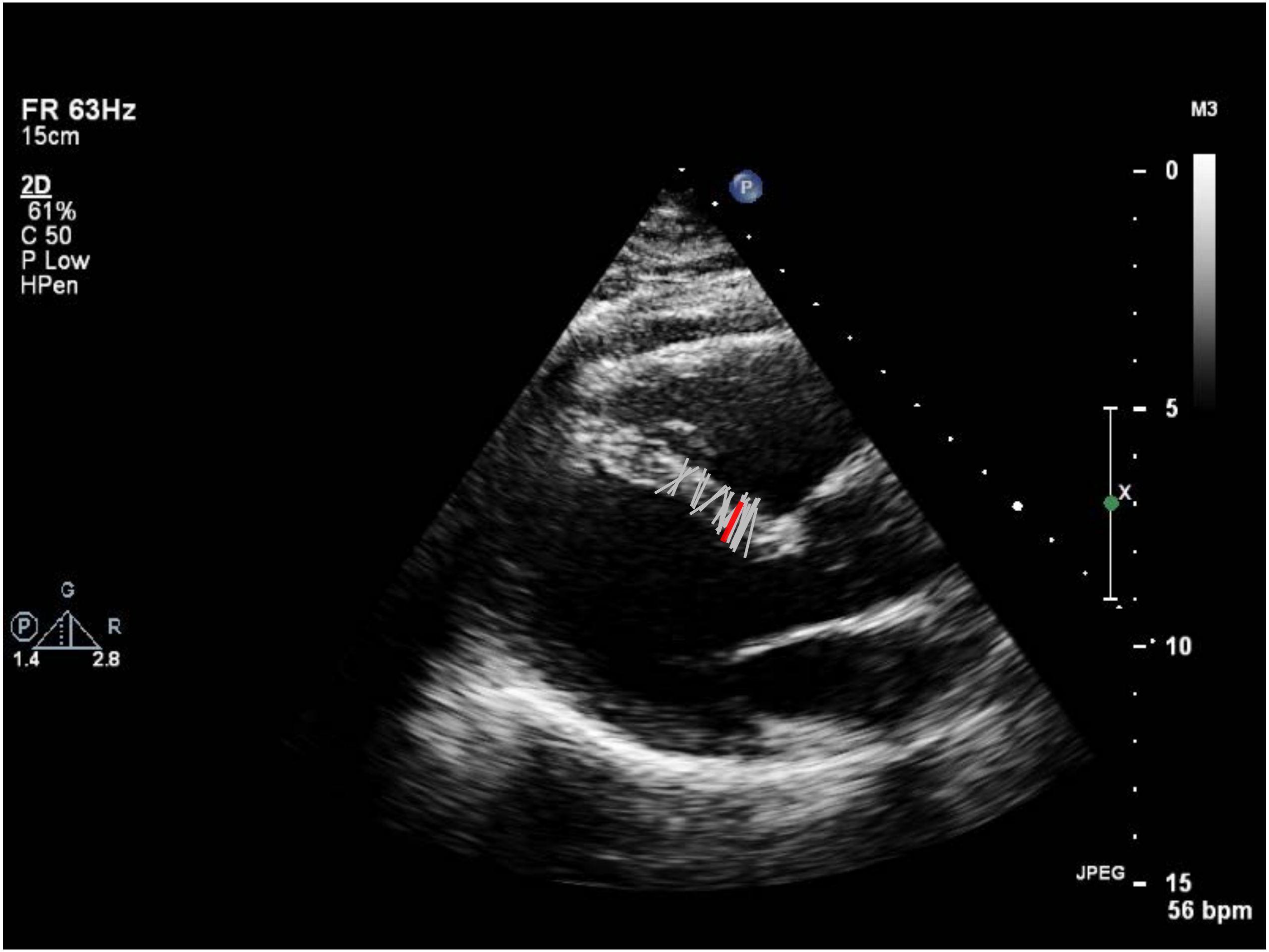

Pt 98 Diastole

Absolute difference between AI and expert consensus: 0.048 mm

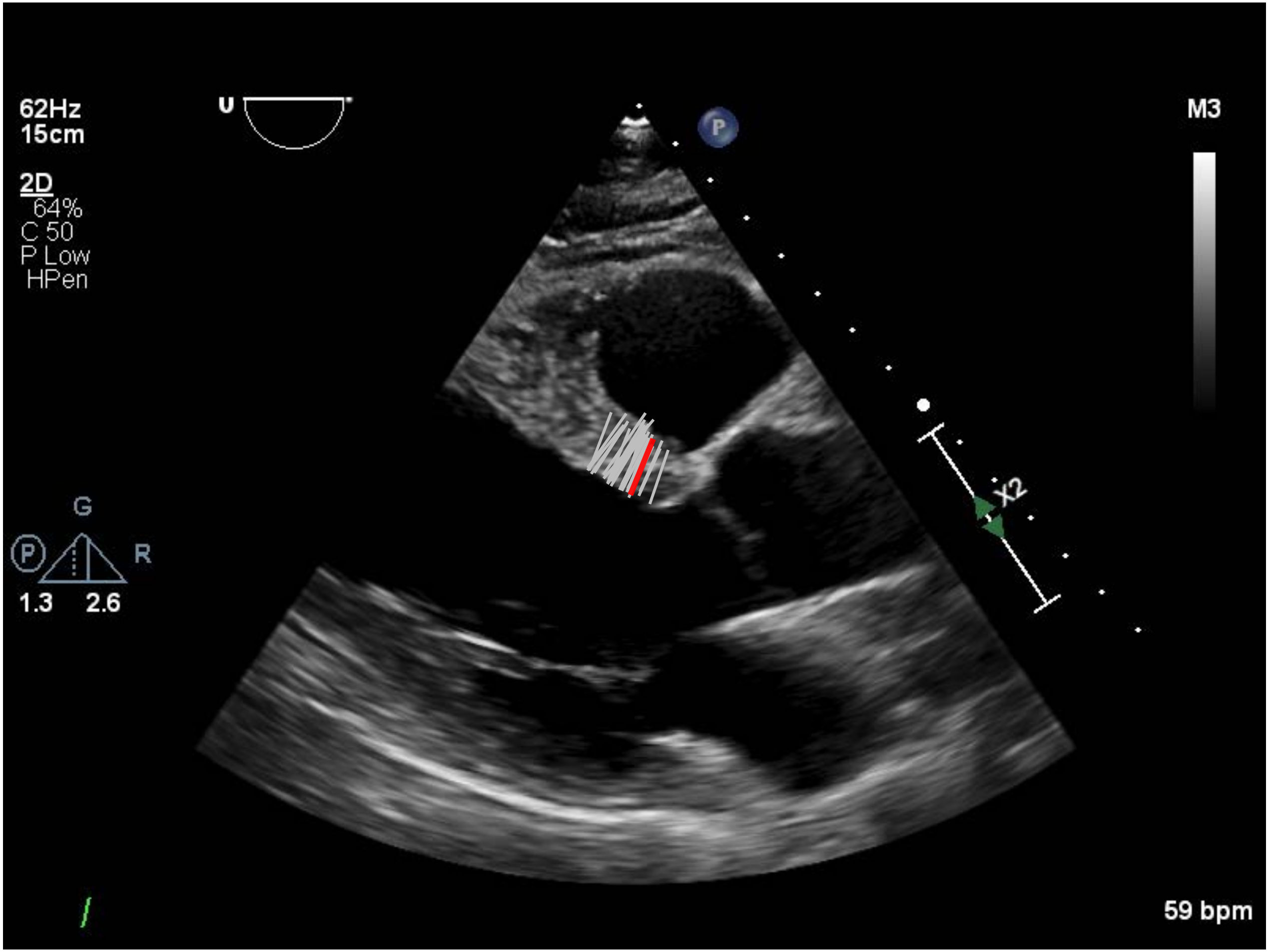

Pt 60 Diastole

Absolute difference between AI and expert consensus: 0.052 mm

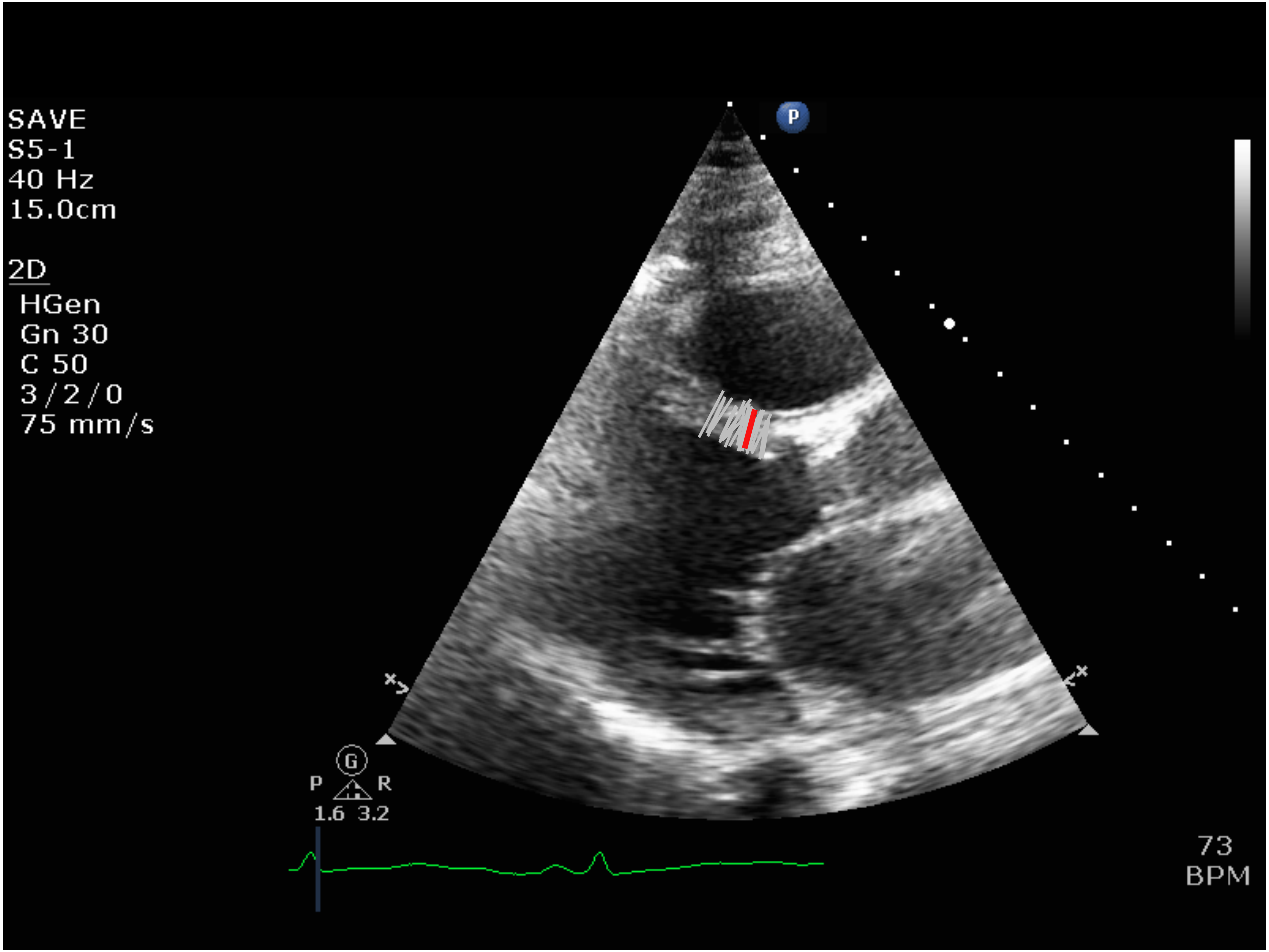

Pt 14 Diastole

Absolute difference between AI and expert consensus: 0.057 mm

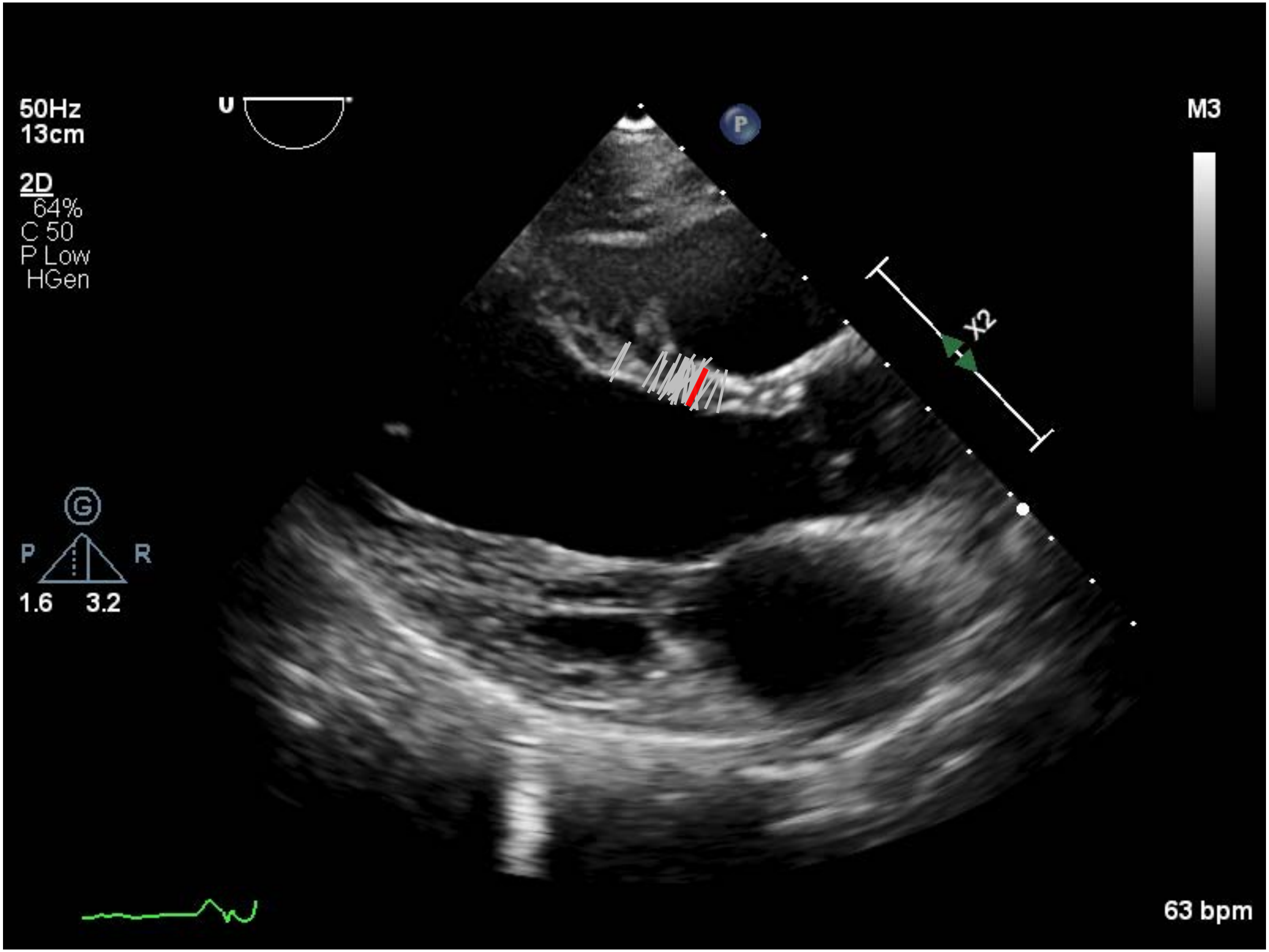

Pt 16 Diastole

Absolute difference between AI and expert consensus: 0.08 mm

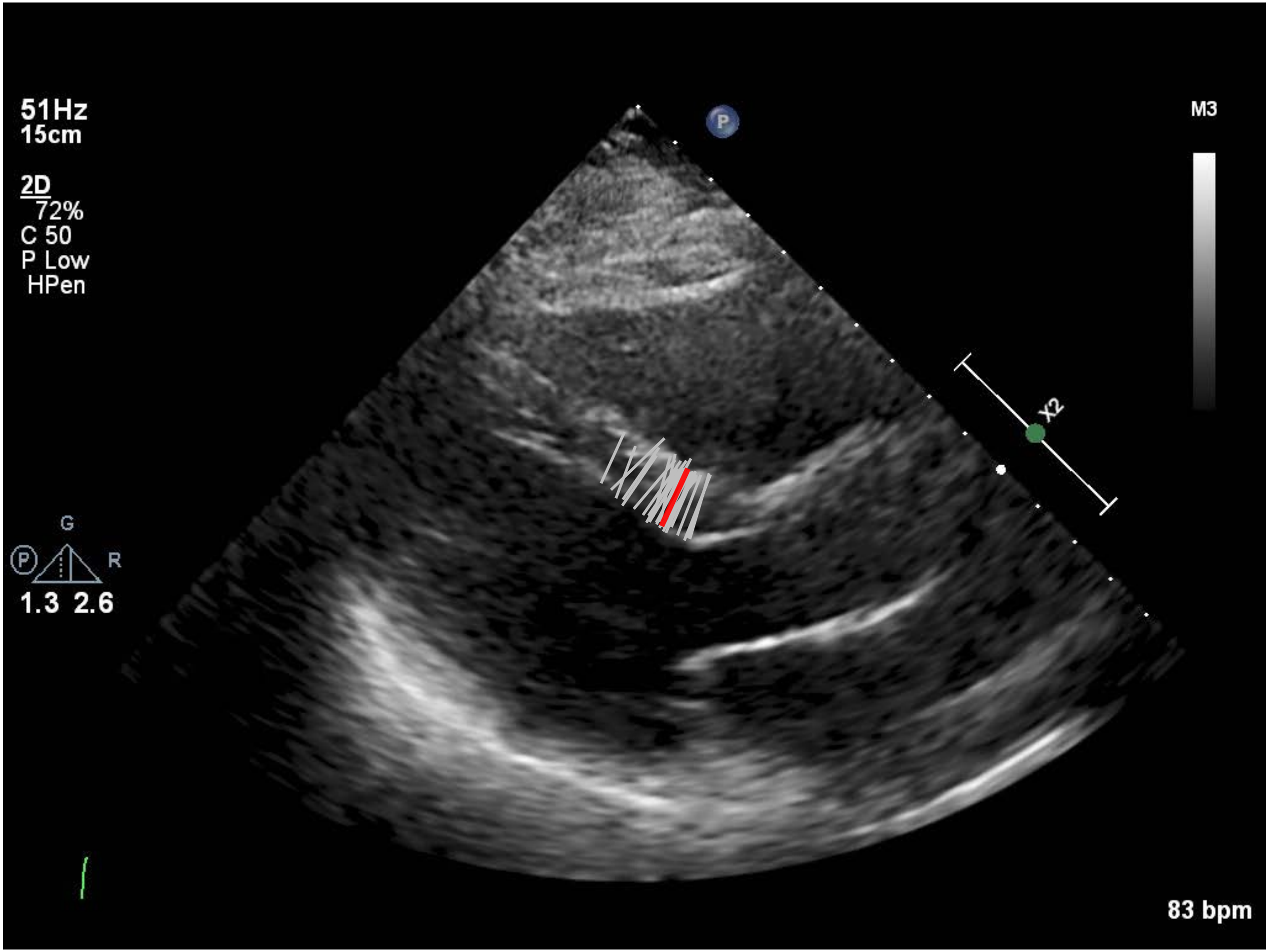

Pt 63 Diastole

Absolute difference between AI and expert consensus: 0.087 mm

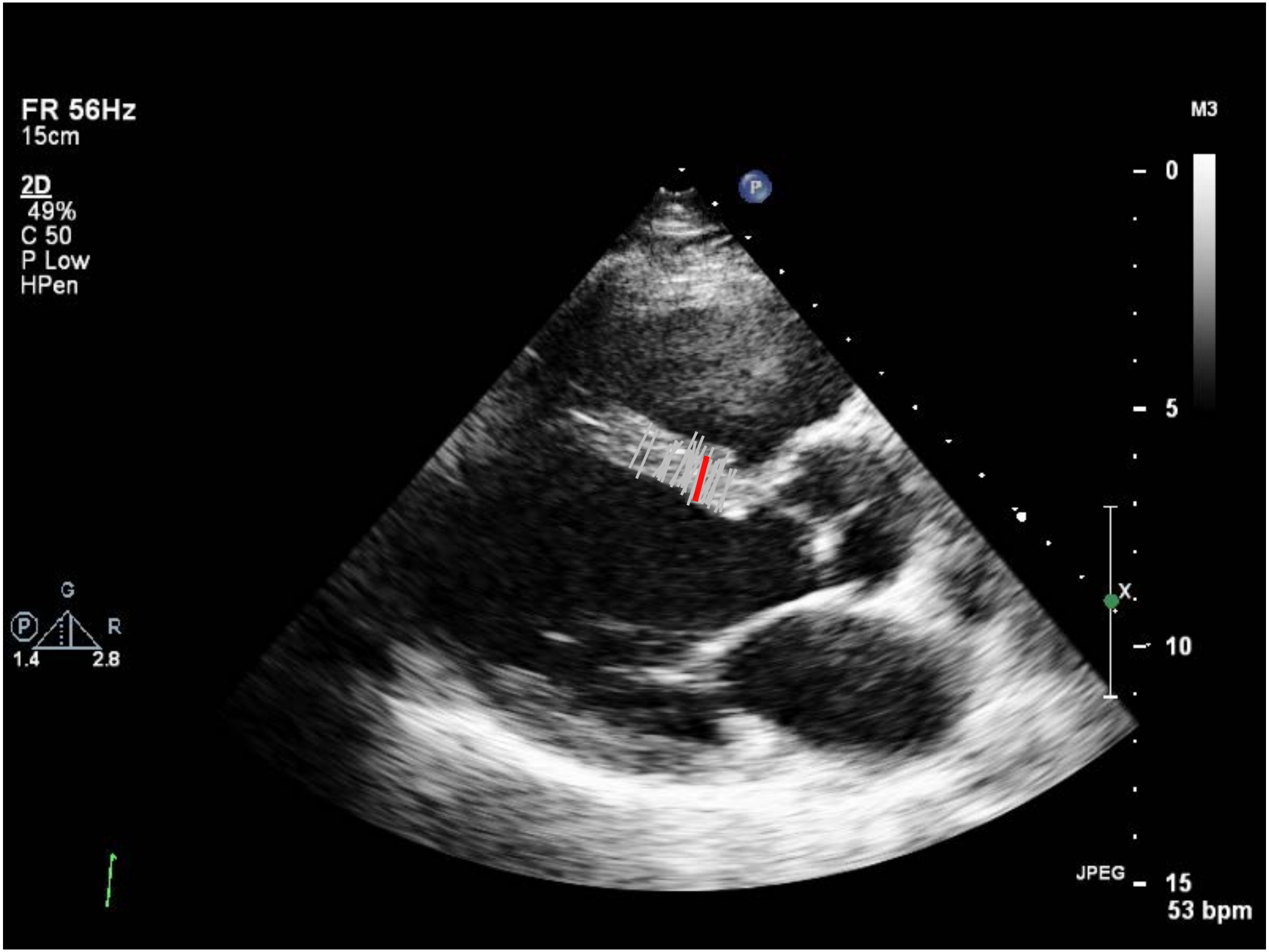

Pt 29 Diastole

Absolute difference between AI and expert consensus: 0.094 mm

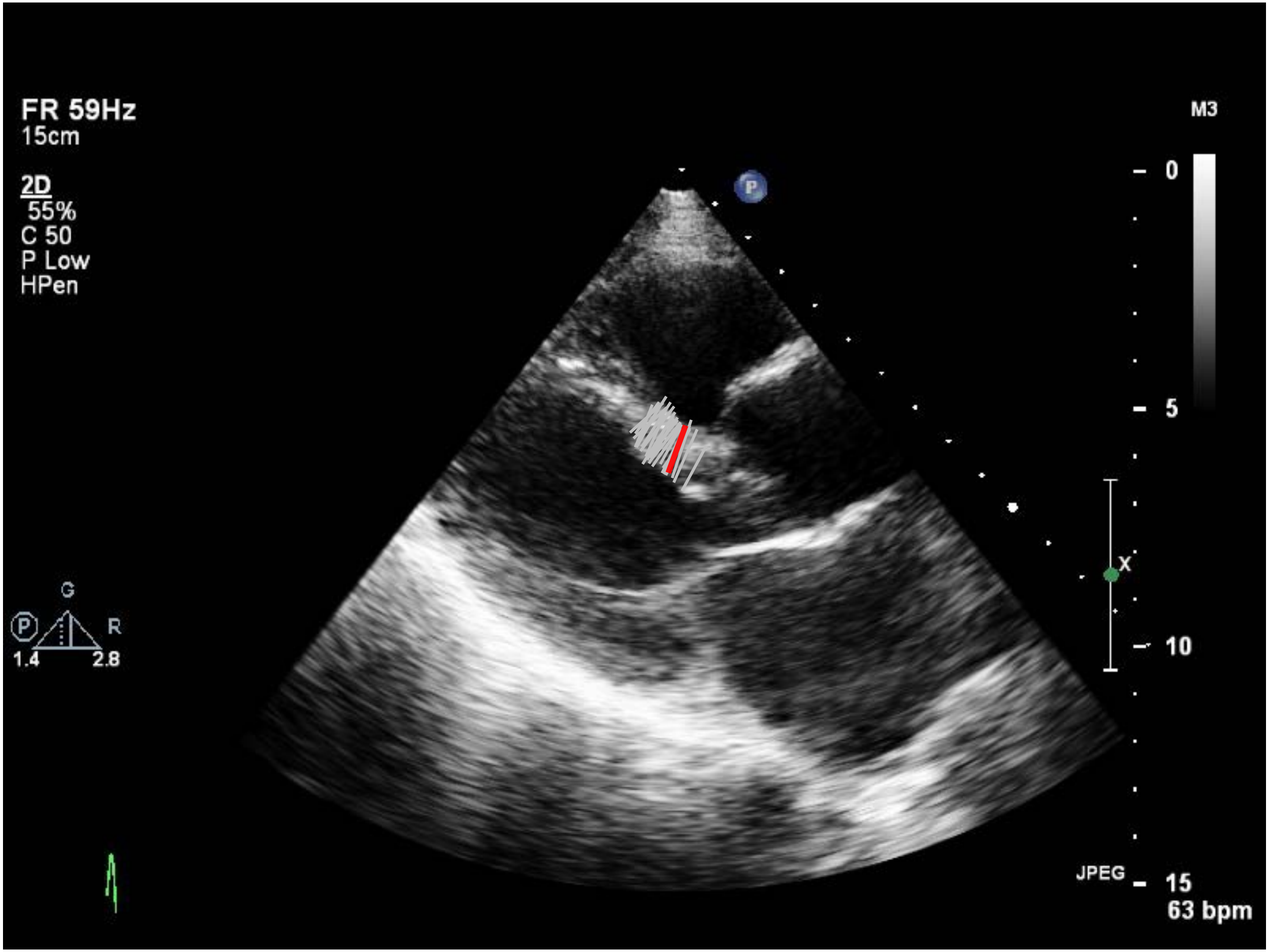

Pt 45 Diastole

Absolute difference between AI and expert consensus: 0.11 mm

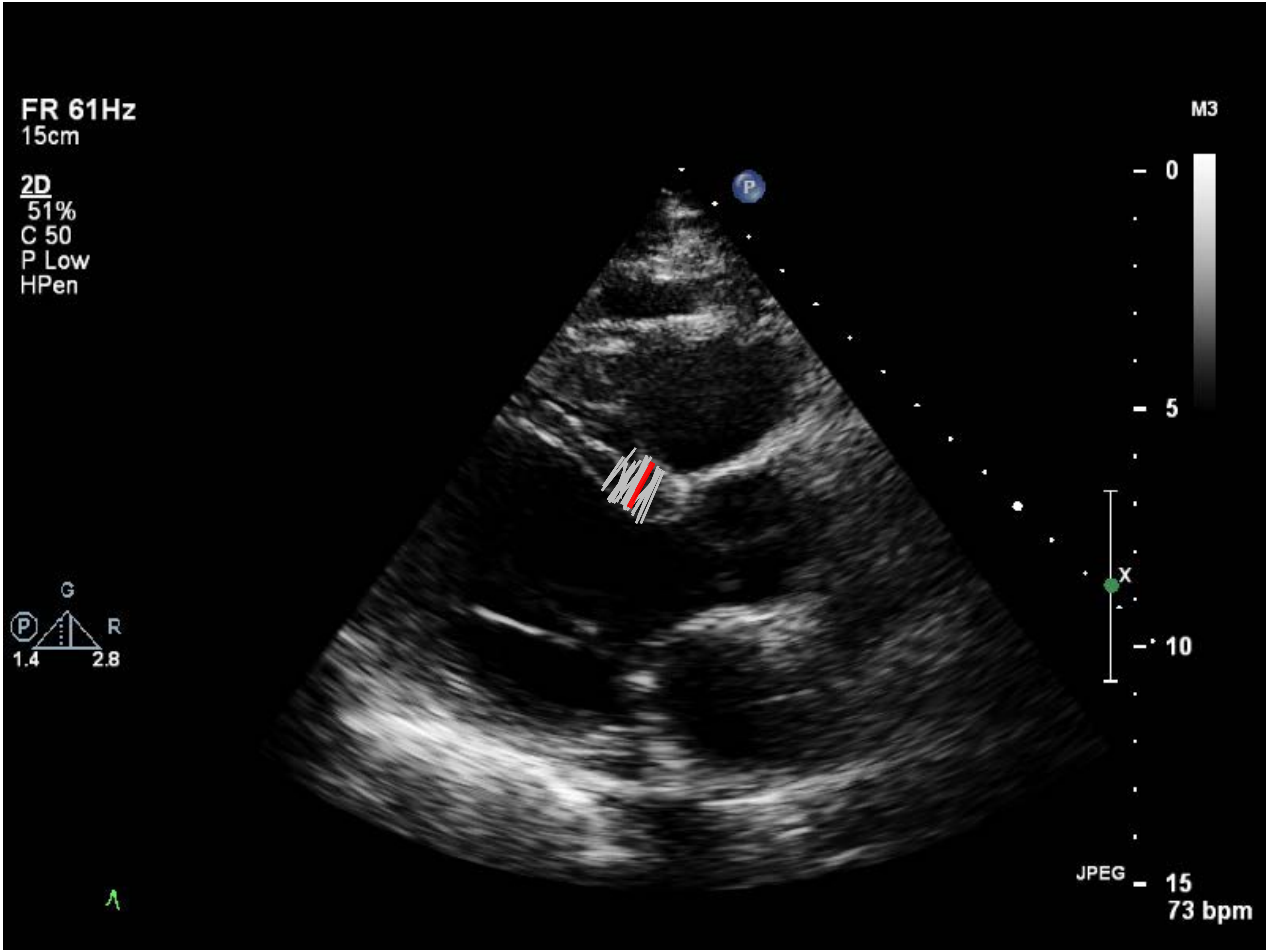

Pt 93 Diastole

Absolute difference between AI and expert consensus: 0.11 mm

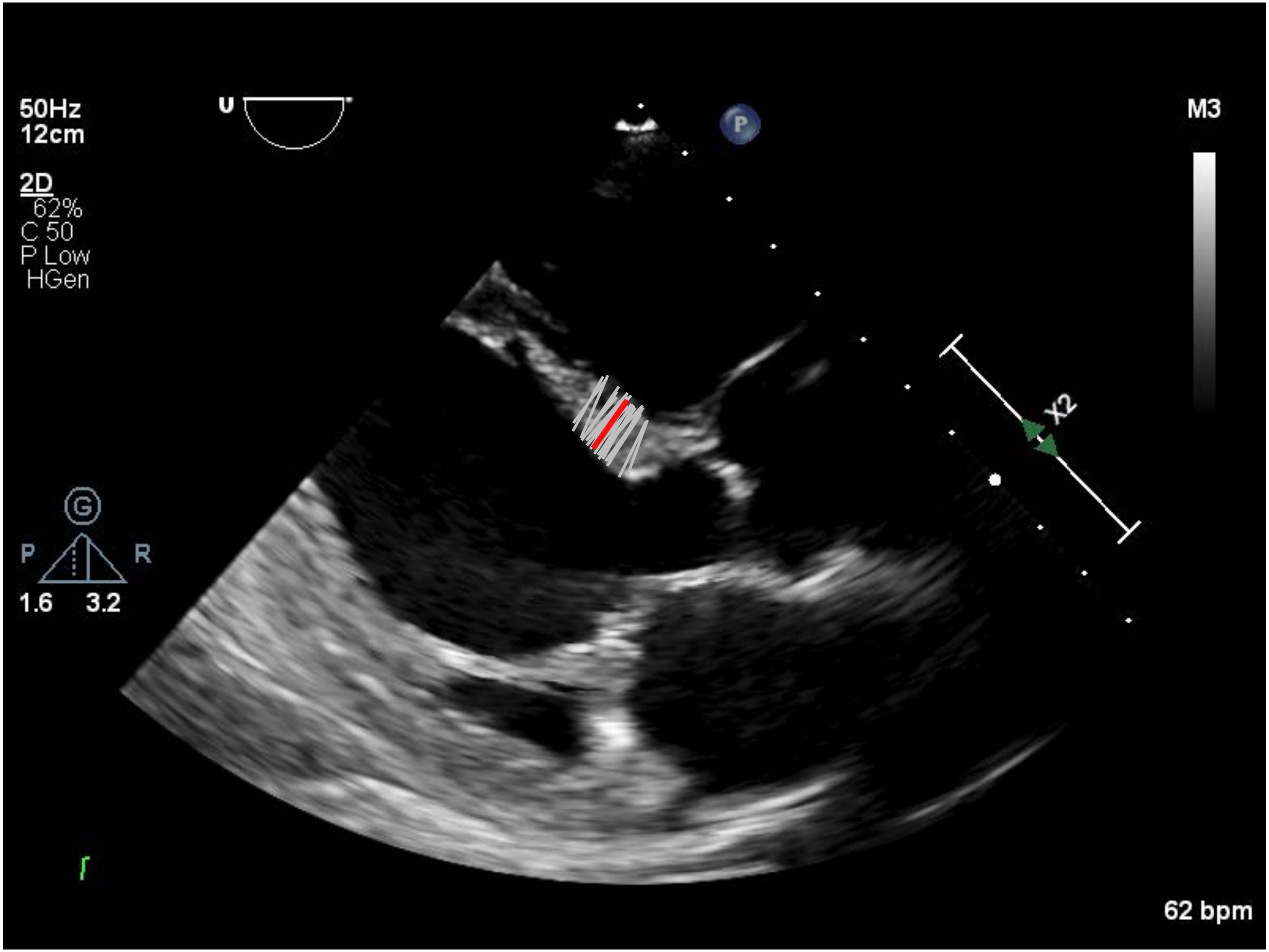

Pt 9 Diastole

Absolute difference between AI and expert consensus: 0.13 mm

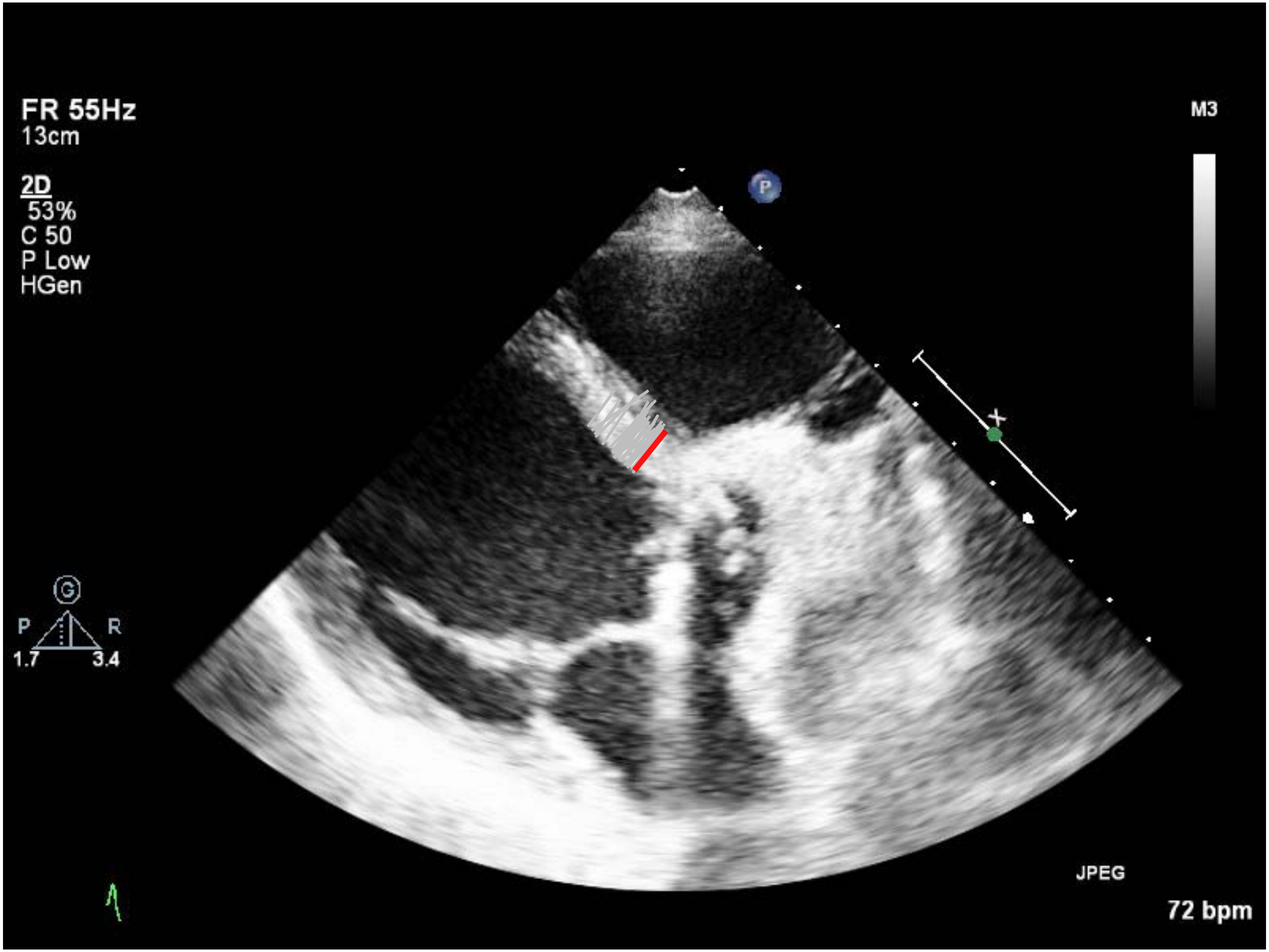

Pt 28 Diastole

Absolute difference between AI and expert consensus: 0.2 mm

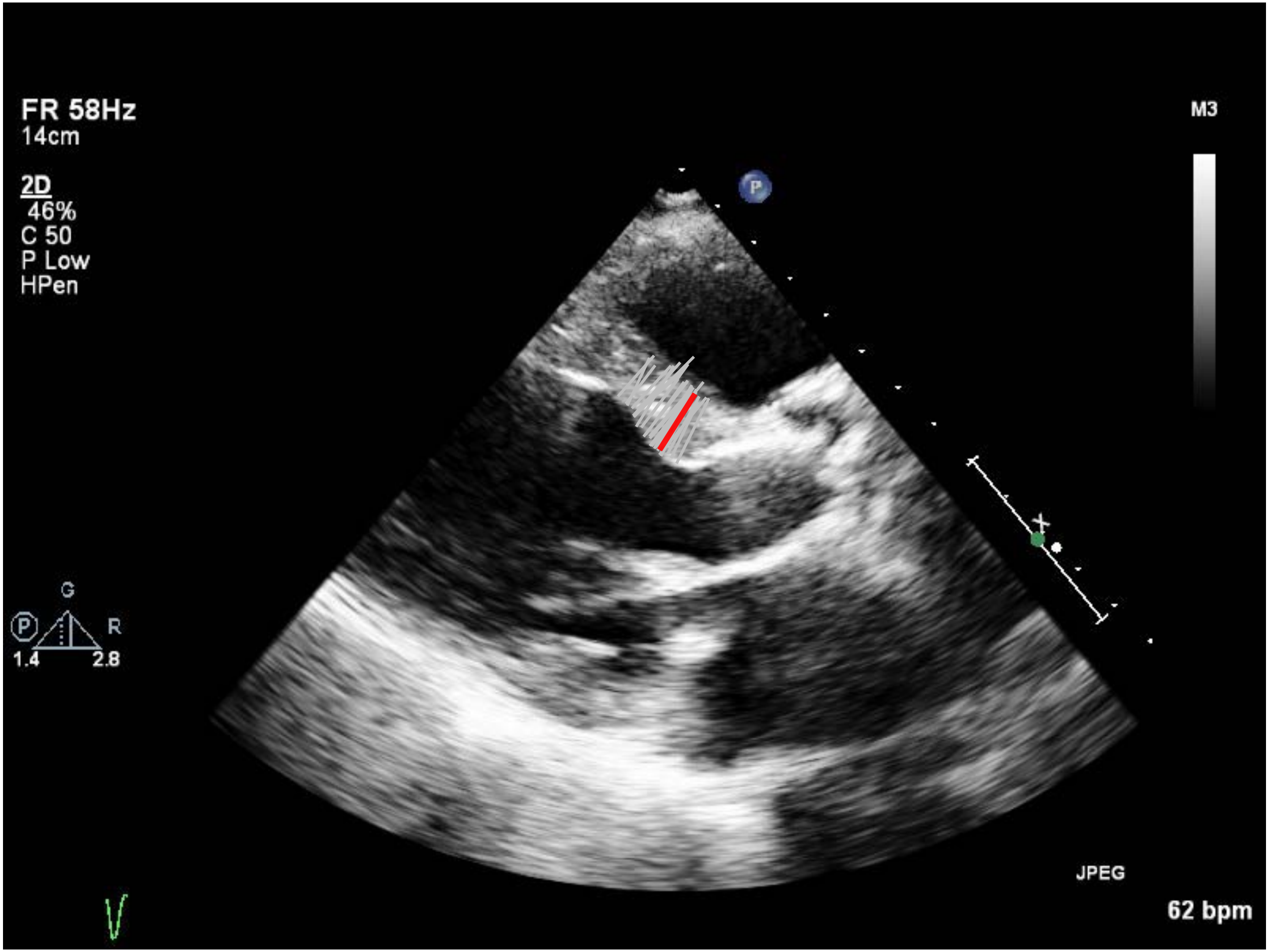

Pt 92 Diastole

Absolute difference between AI and expert consensus: 0.2 mm

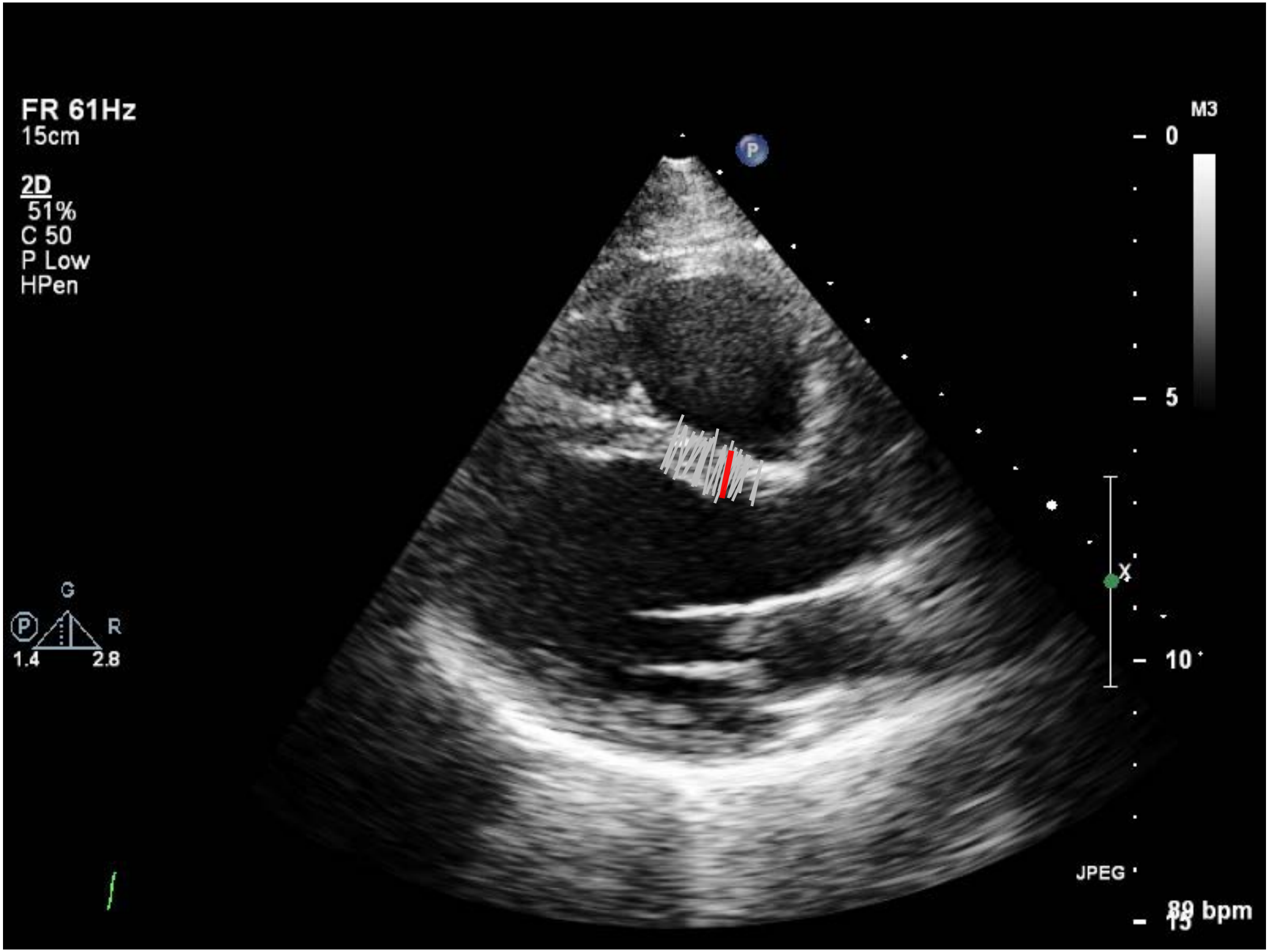

Pt 58 Diastole

Absolute difference between AI and expert consensus: 0.22 mm

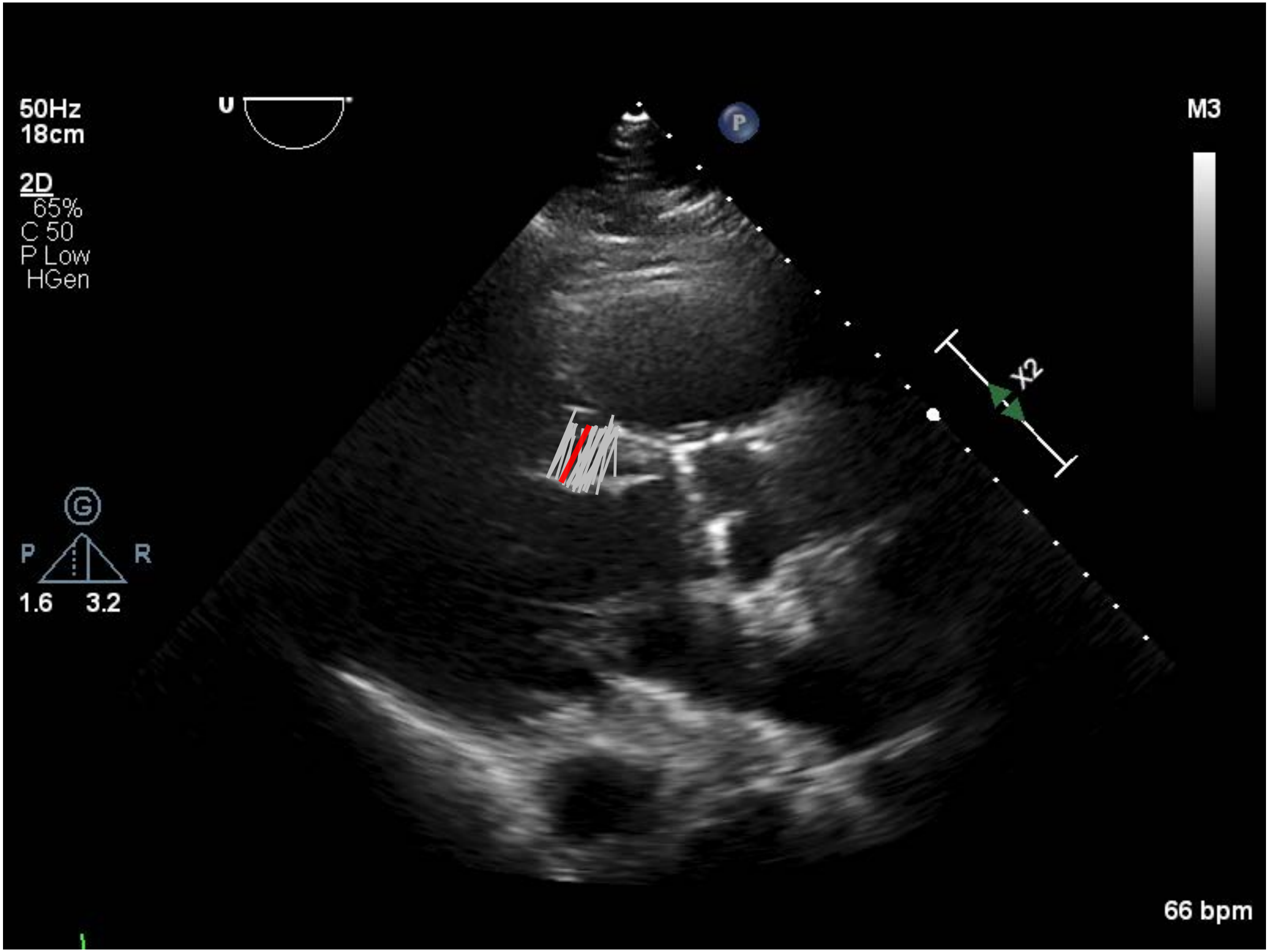

Pt 11 Diastole

Absolute difference between AI and expert consensus: 0.23 mm

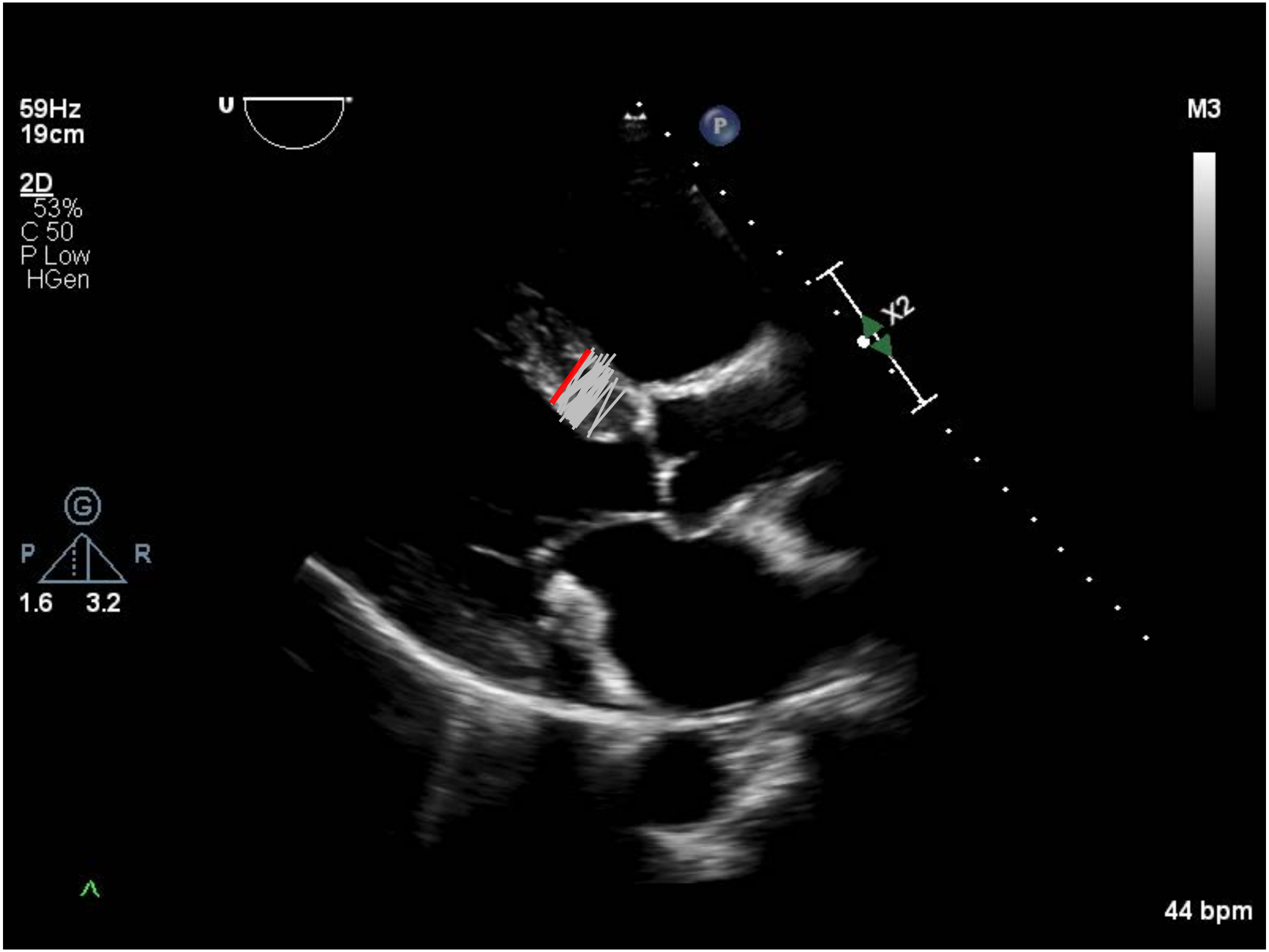

Pt 13 Diastole

Absolute difference between AI and expert consensus: 0.24 mm

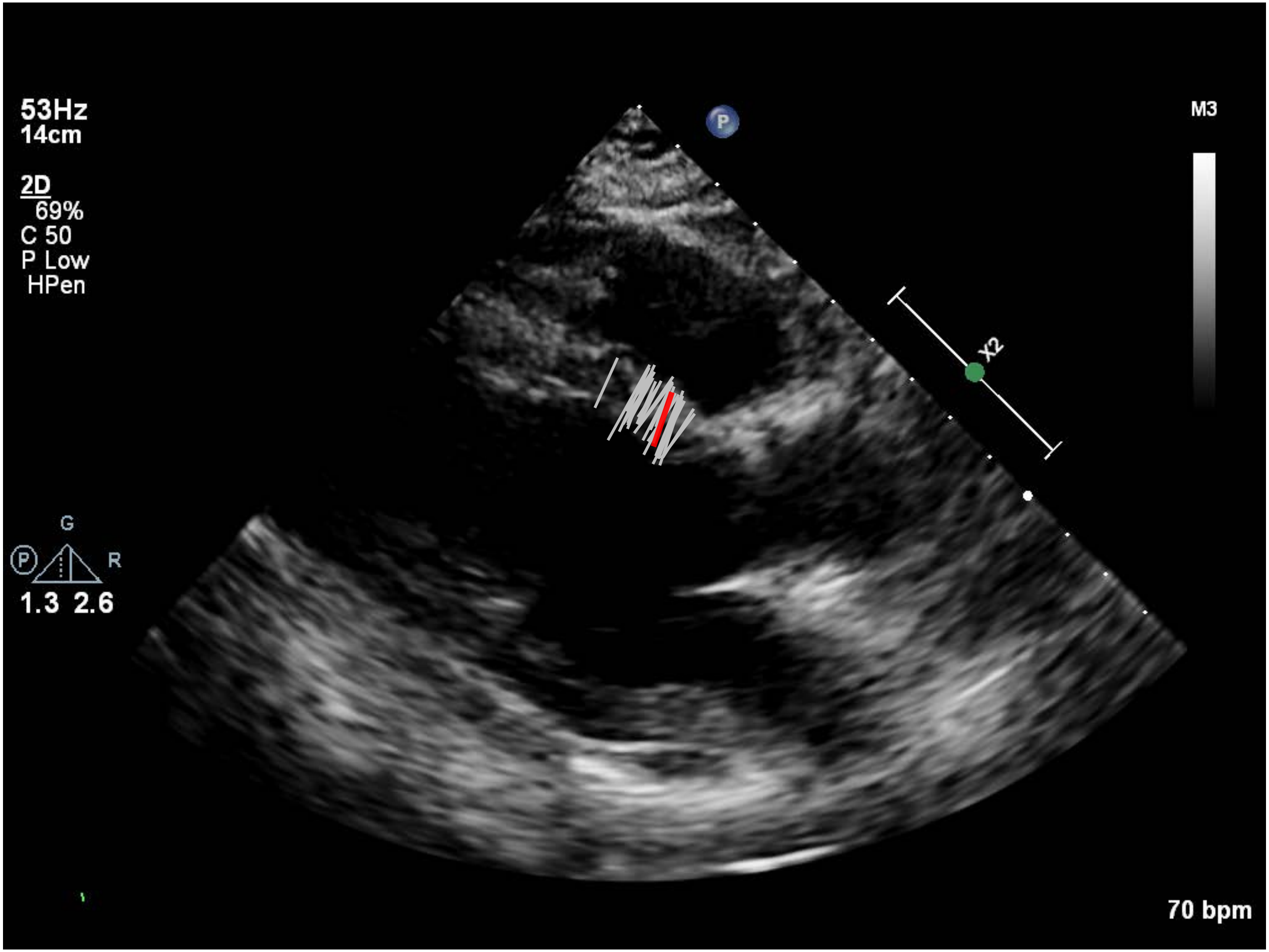

Pt 83 Diastole
Absolute difference between AI and expert consensus: 0.24 mm

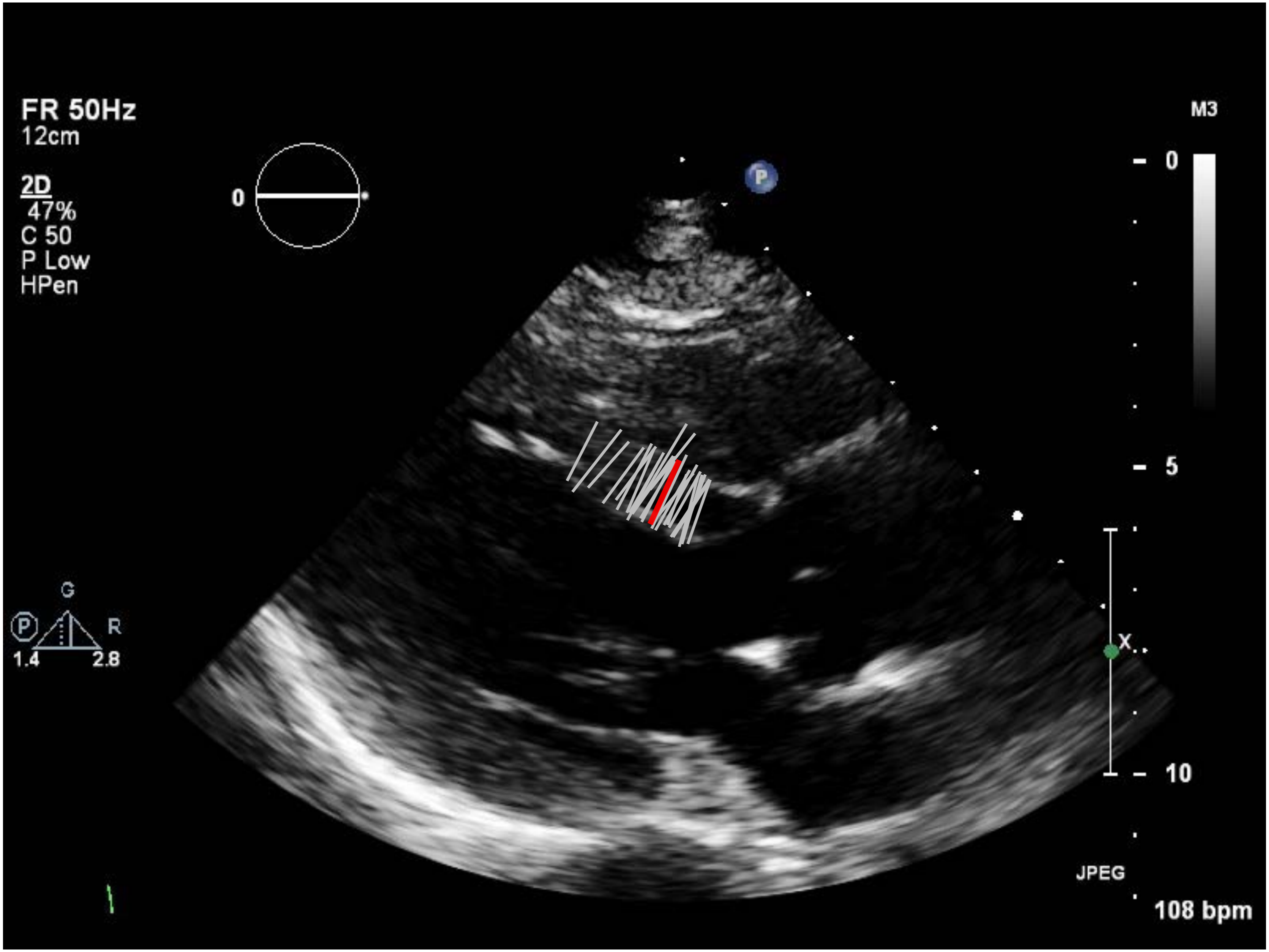

Pt 89 Diastole

Absolute difference between AI and expert consensus: 0.24 mm

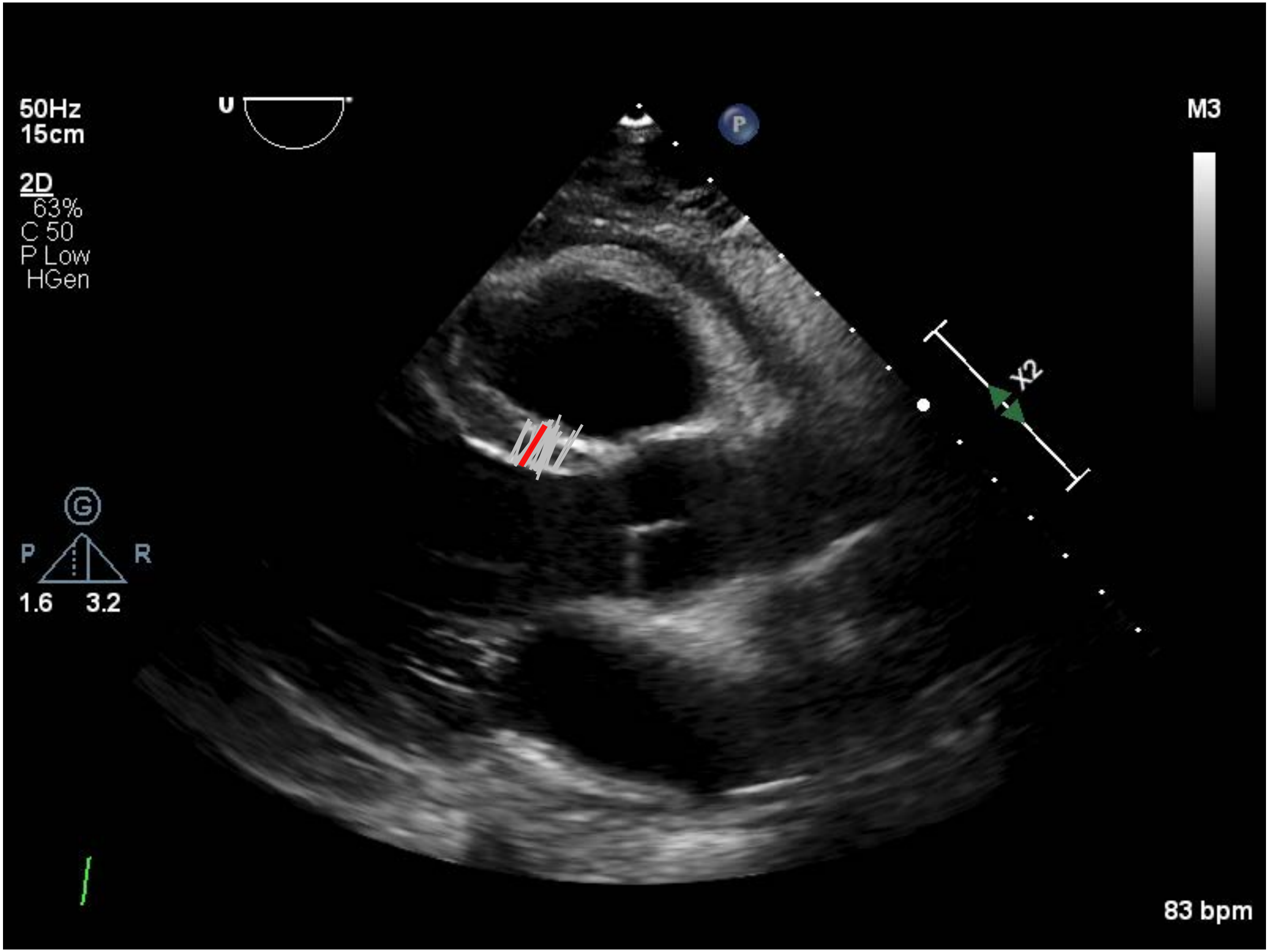

Pt 4 Diastole

Absolute difference between AI and expert consensus: 0.27 mm

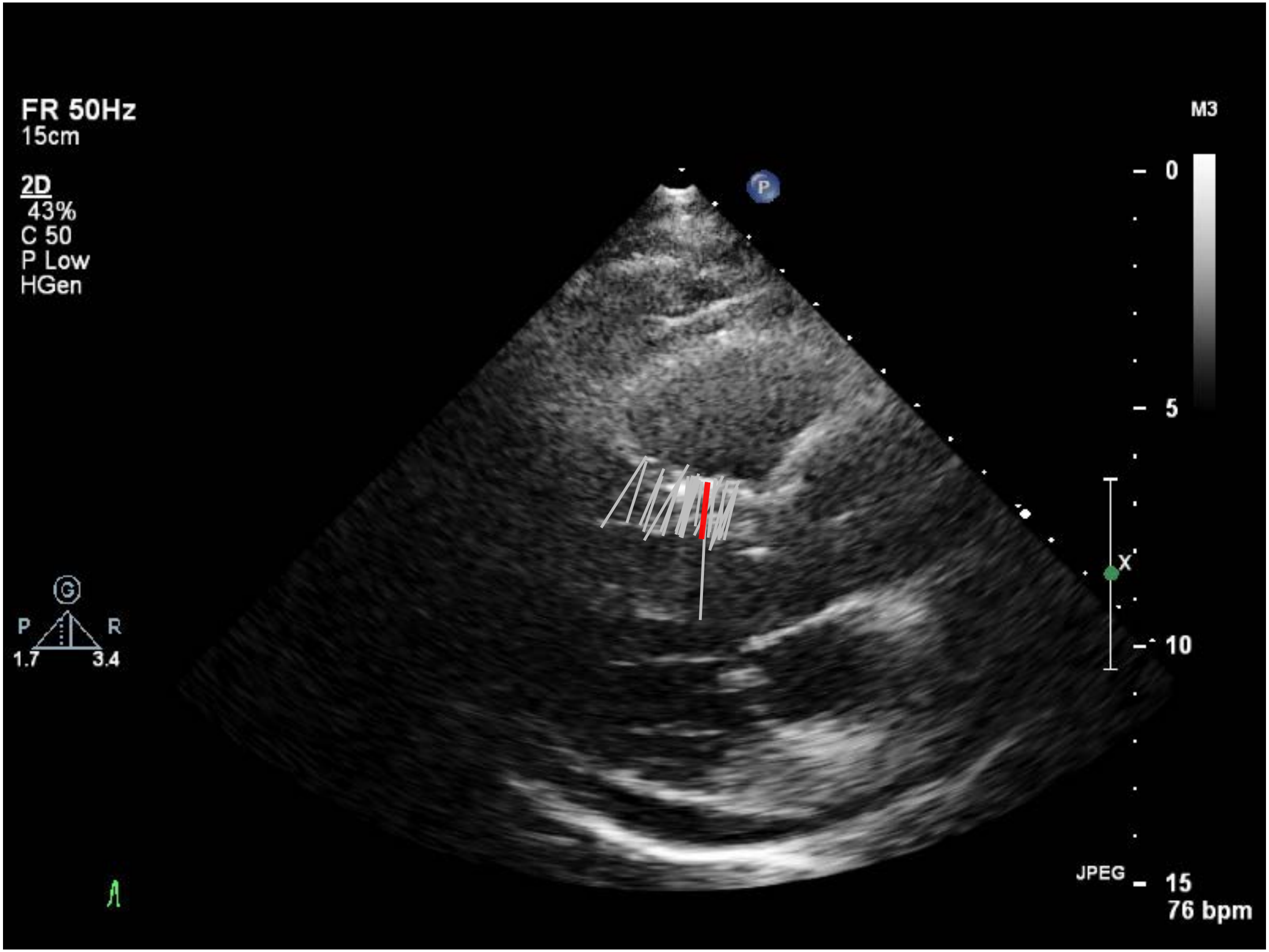

Pt 1 Diastole

Absolute difference between AI and expert consensus: 0.27 mm

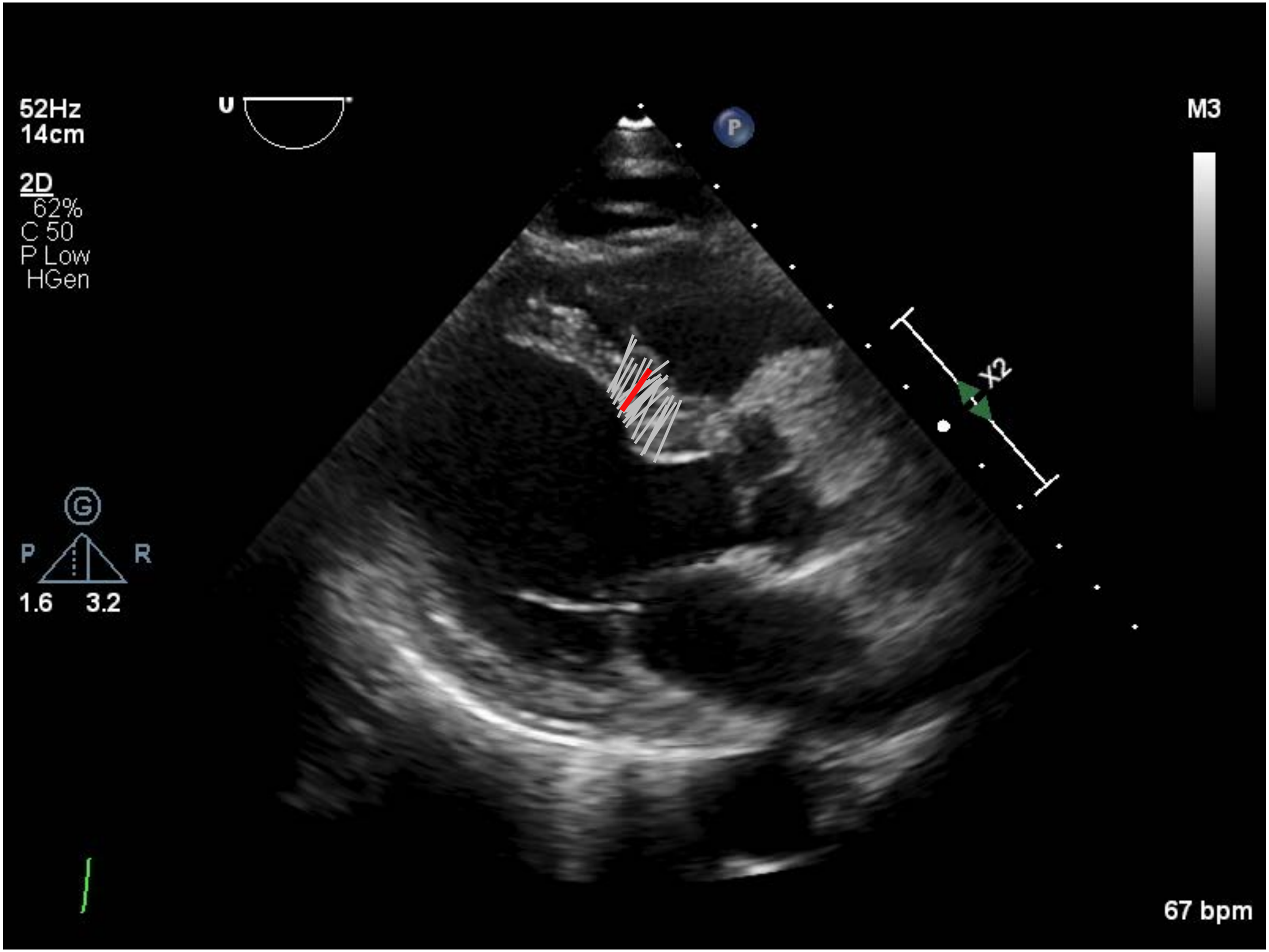

Pt 80 Diastole

Absolute difference between AI and expert consensus: 0.29 mm

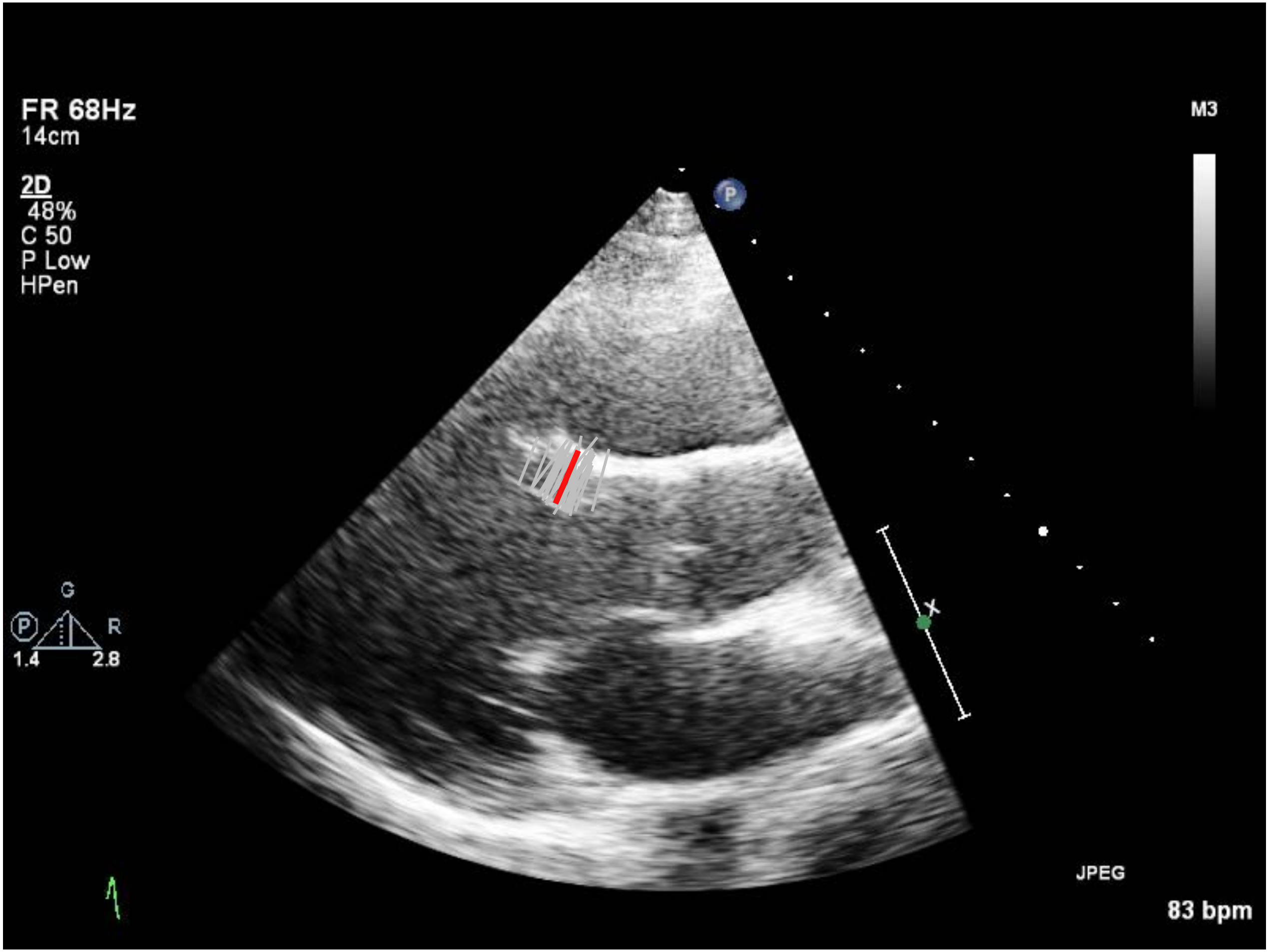

Pt 86 Diastole

Absolute difference between AI and expert consensus: 0.32 mm

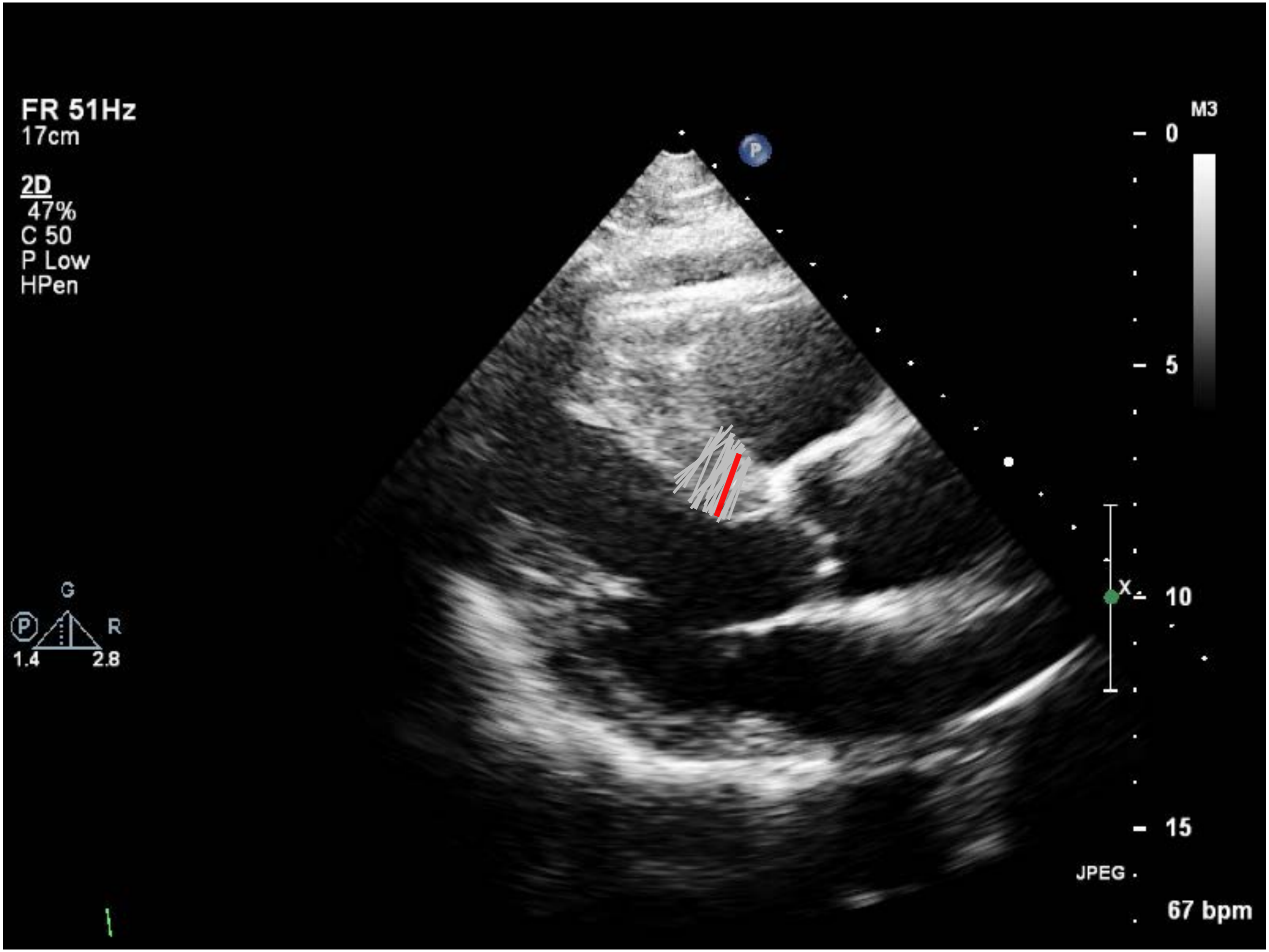

Pt 5 Diastole

Absolute difference between AI and expert consensus: 0.34 mm

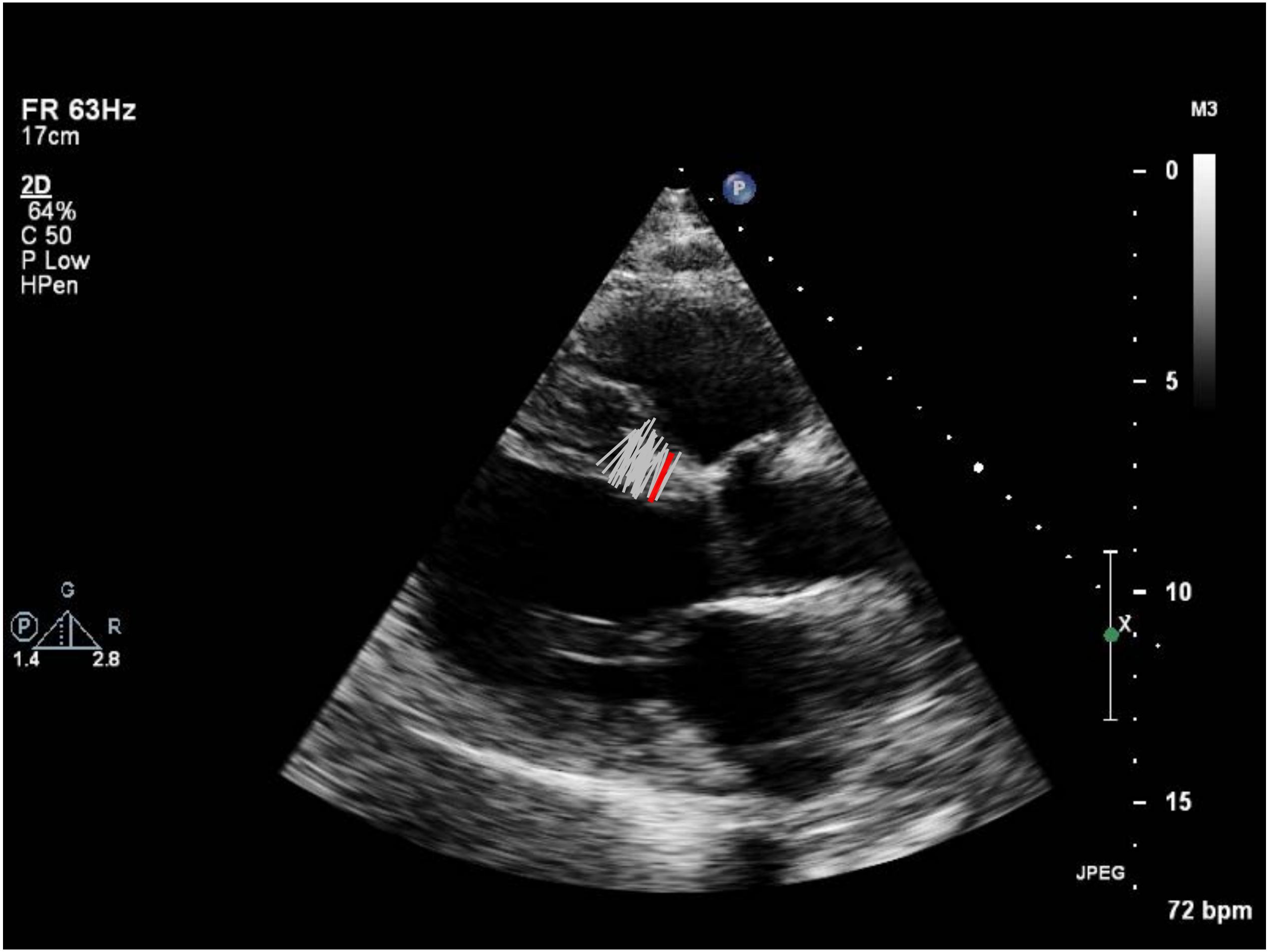

Pt 64 Diastole

Absolute difference between AI and expert consensus: 0.36 mm

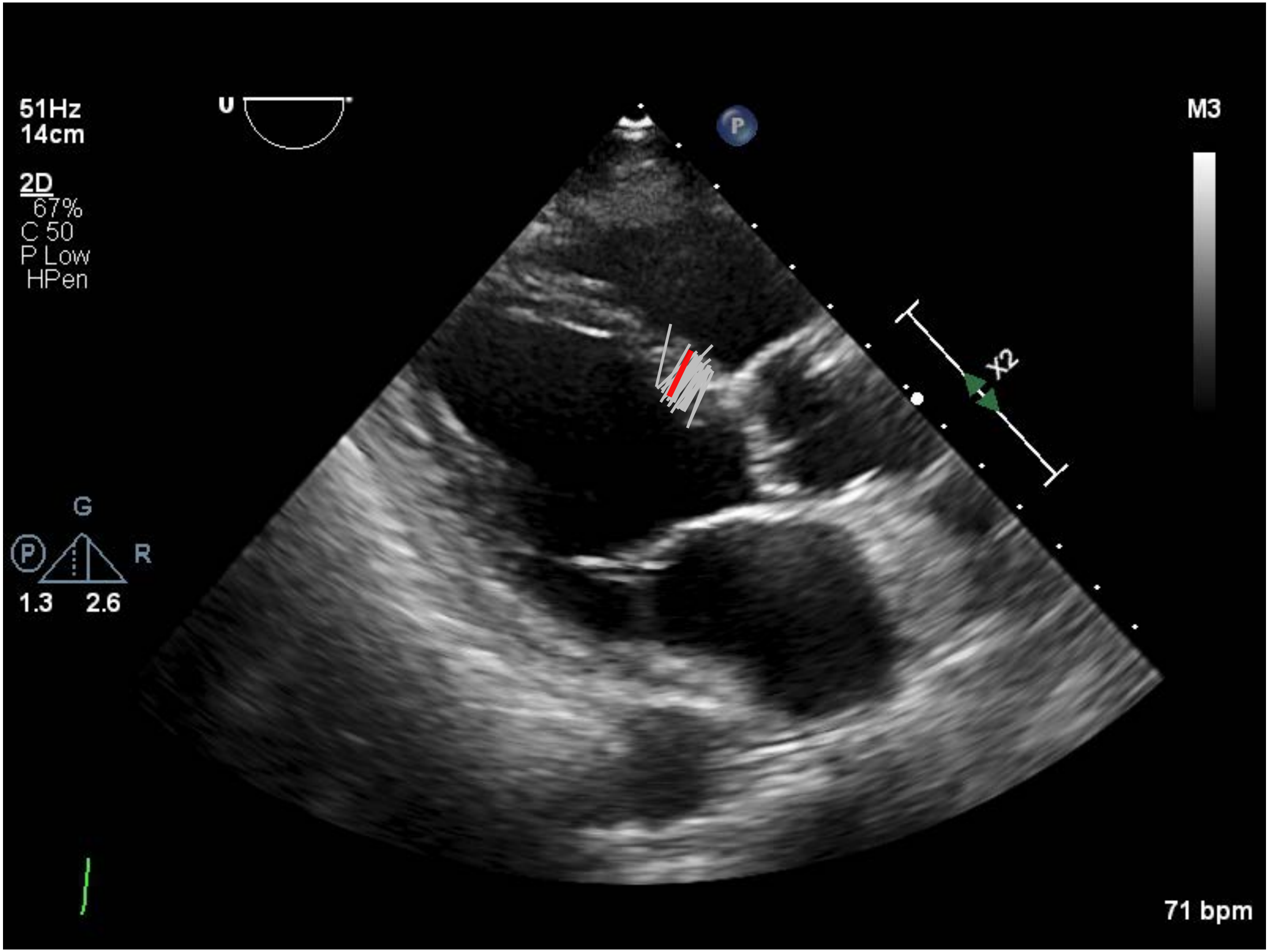

Pt 88 Diastole

Absolute difference between AI and expert consensus: 0.38 mm

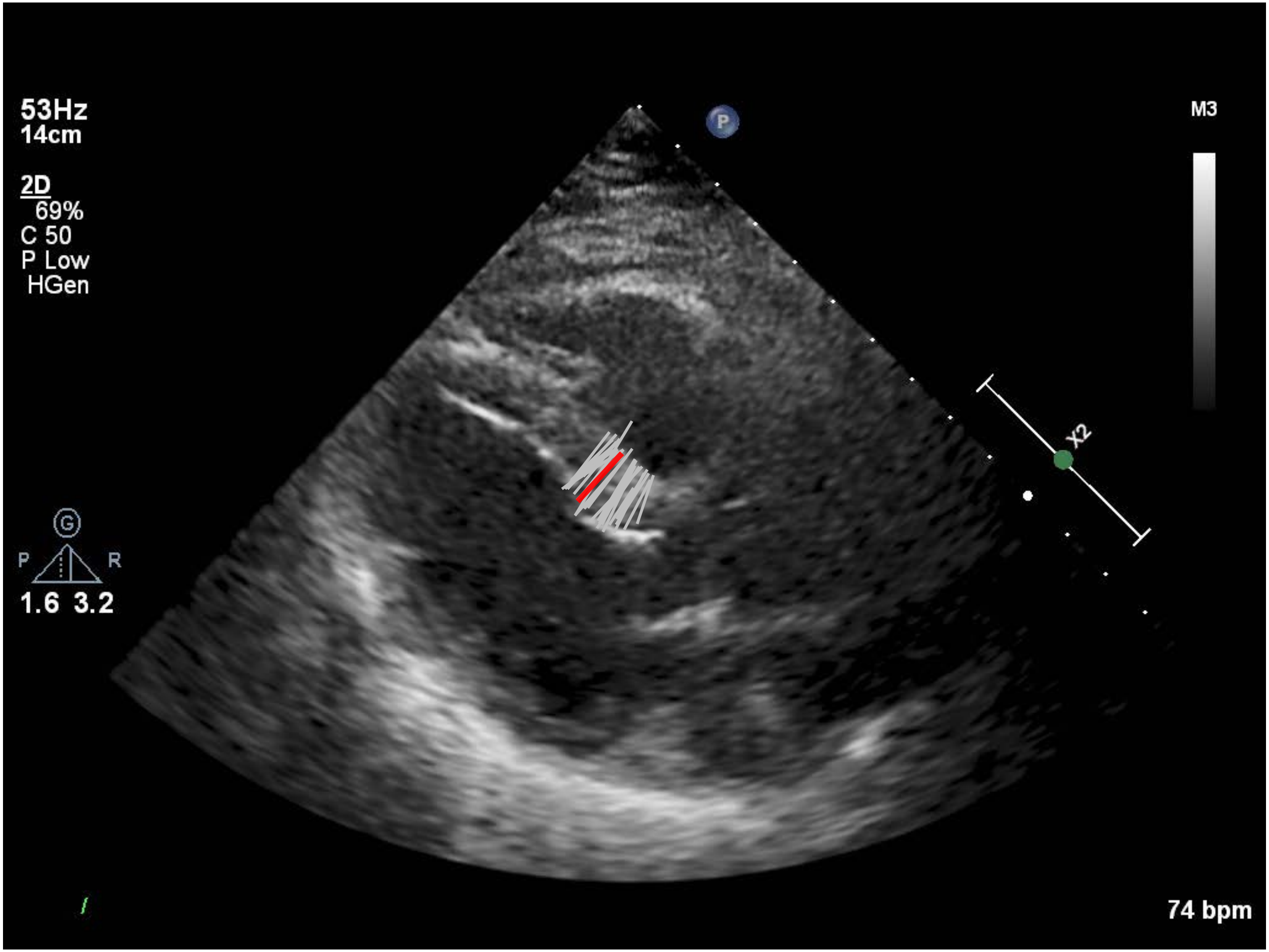

Pt 42 Diastole
Absolute difference between AI and expert consensus: 0.38 mm

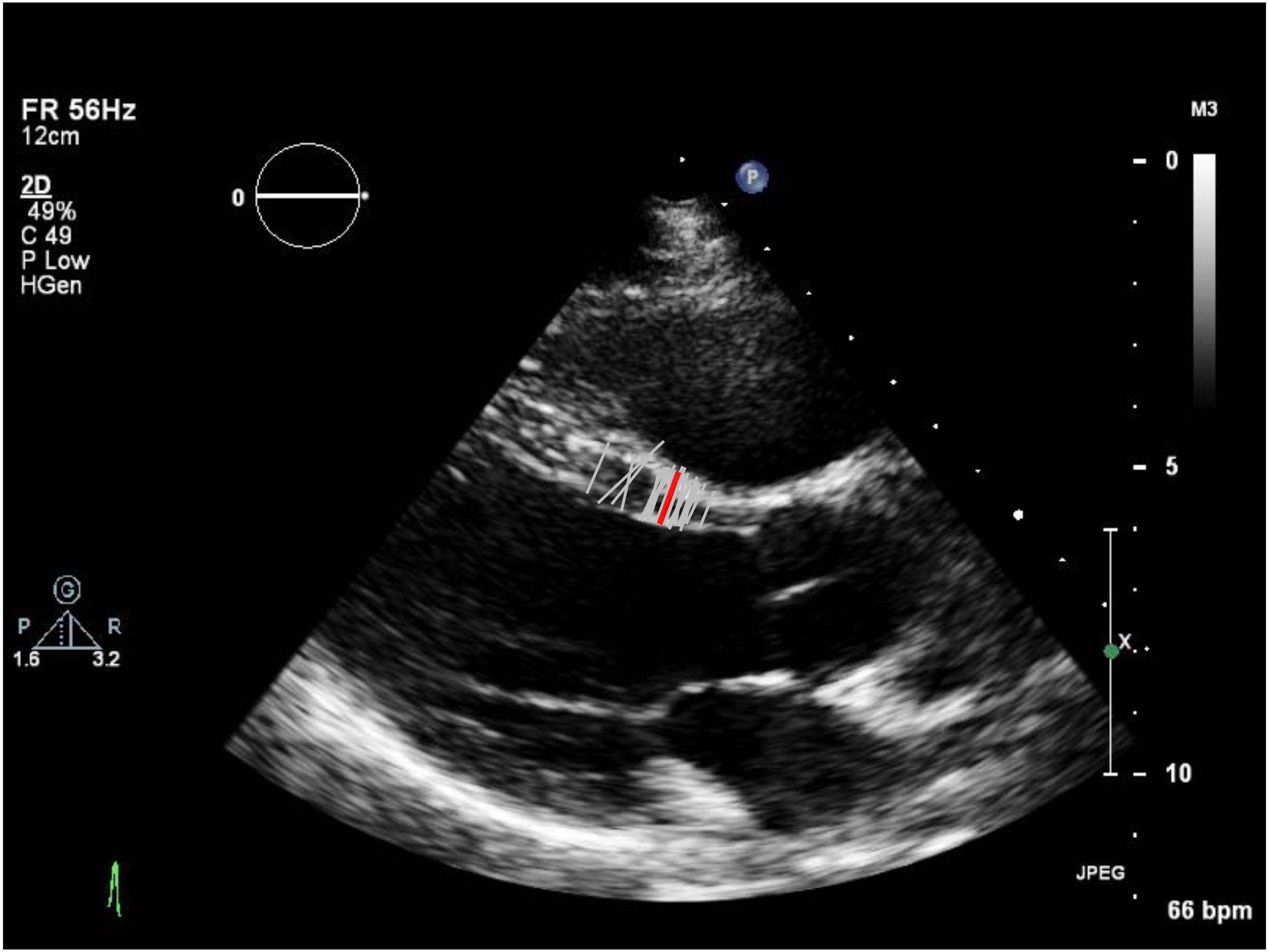

Pt 40 Diastole

Absolute difference between AI and expert consensus: 0.38 mm

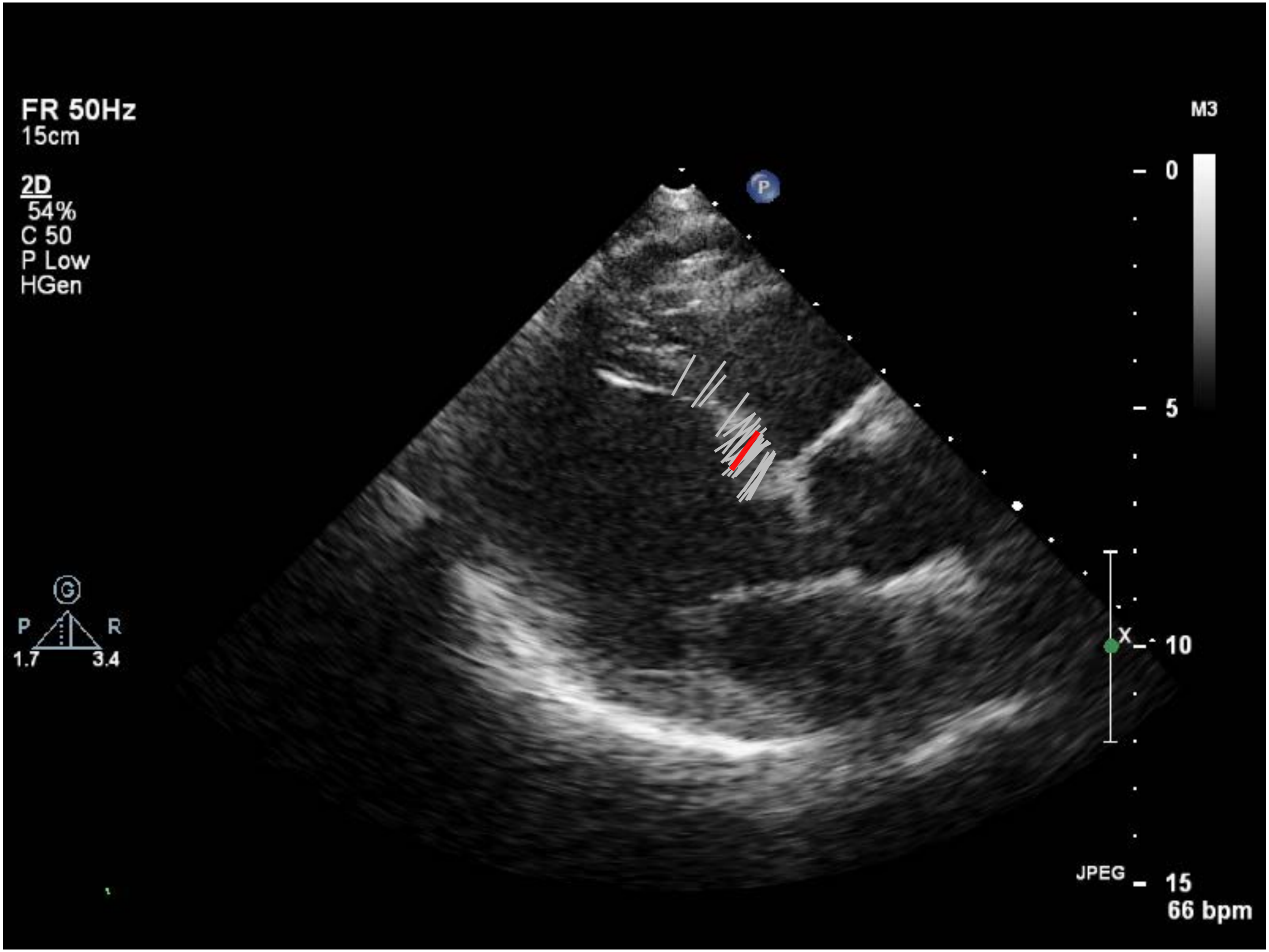

Pt 33 Diastole

Absolute difference between AI and expert consensus: 0.42 mm

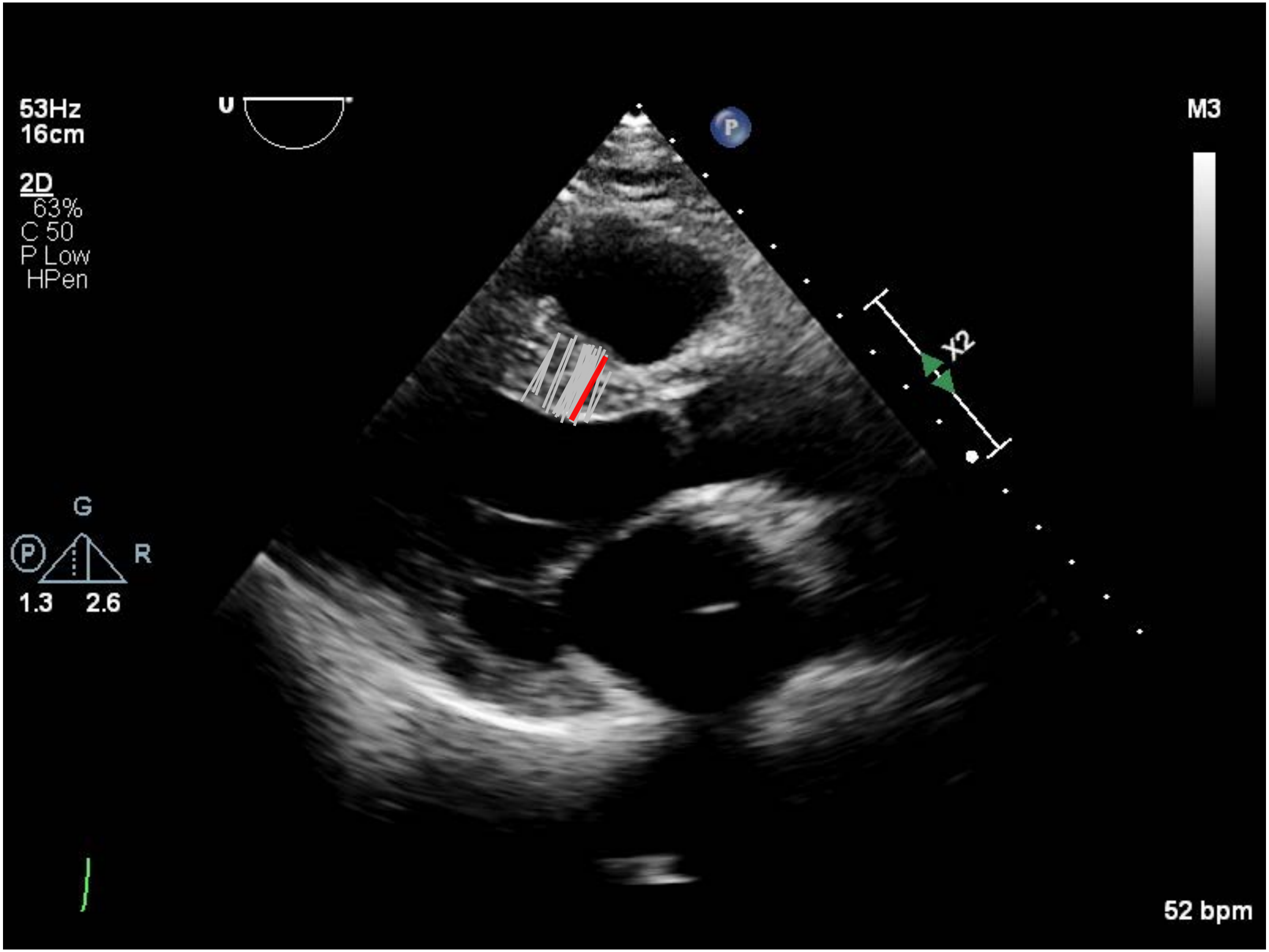

Pt 8 Diastole

Absolute difference between AI and expert consensus: 0.49 mm

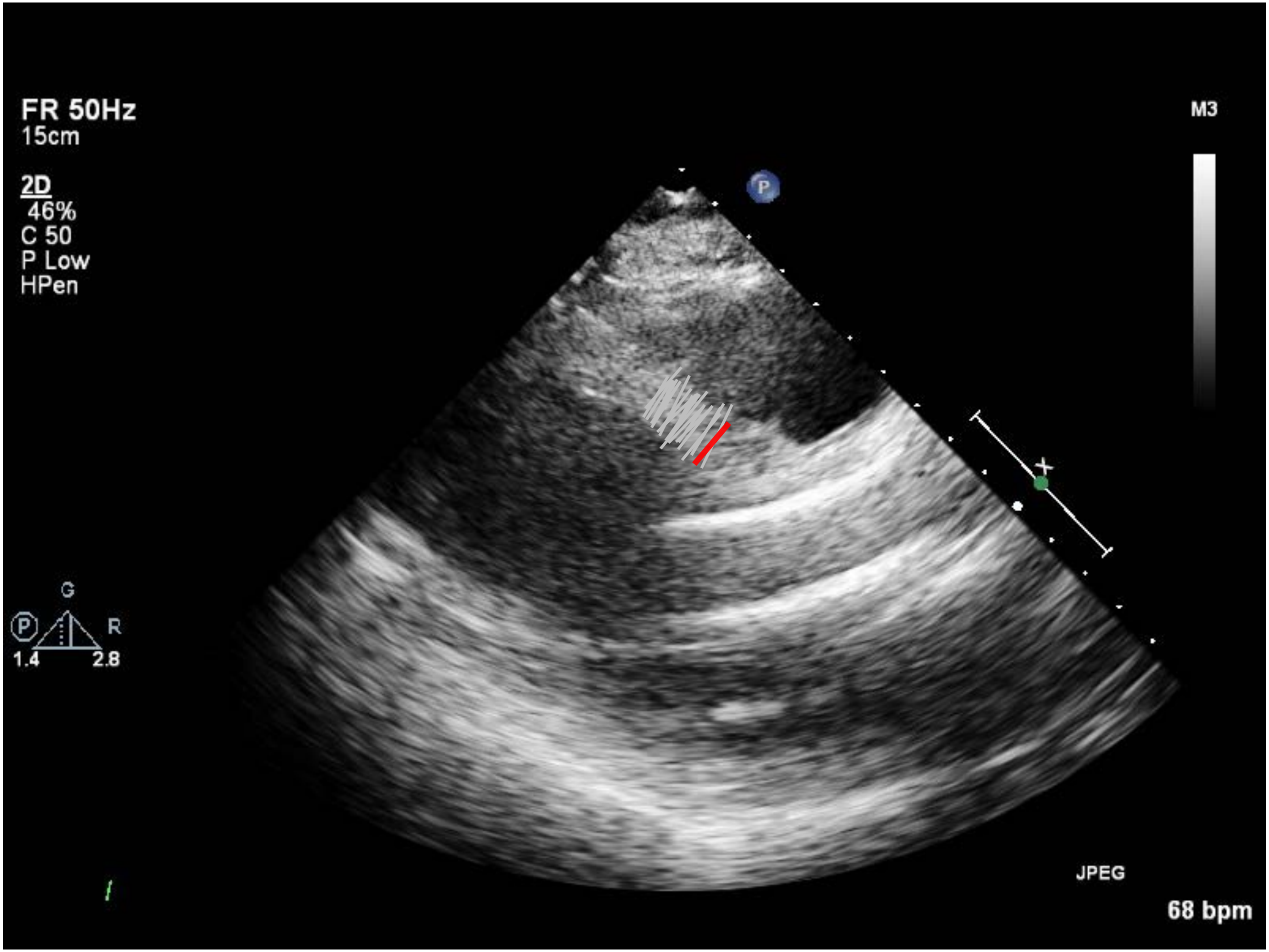

Pt 81 Diastole

Absolute difference between AI and expert consensus: 0.5 mm

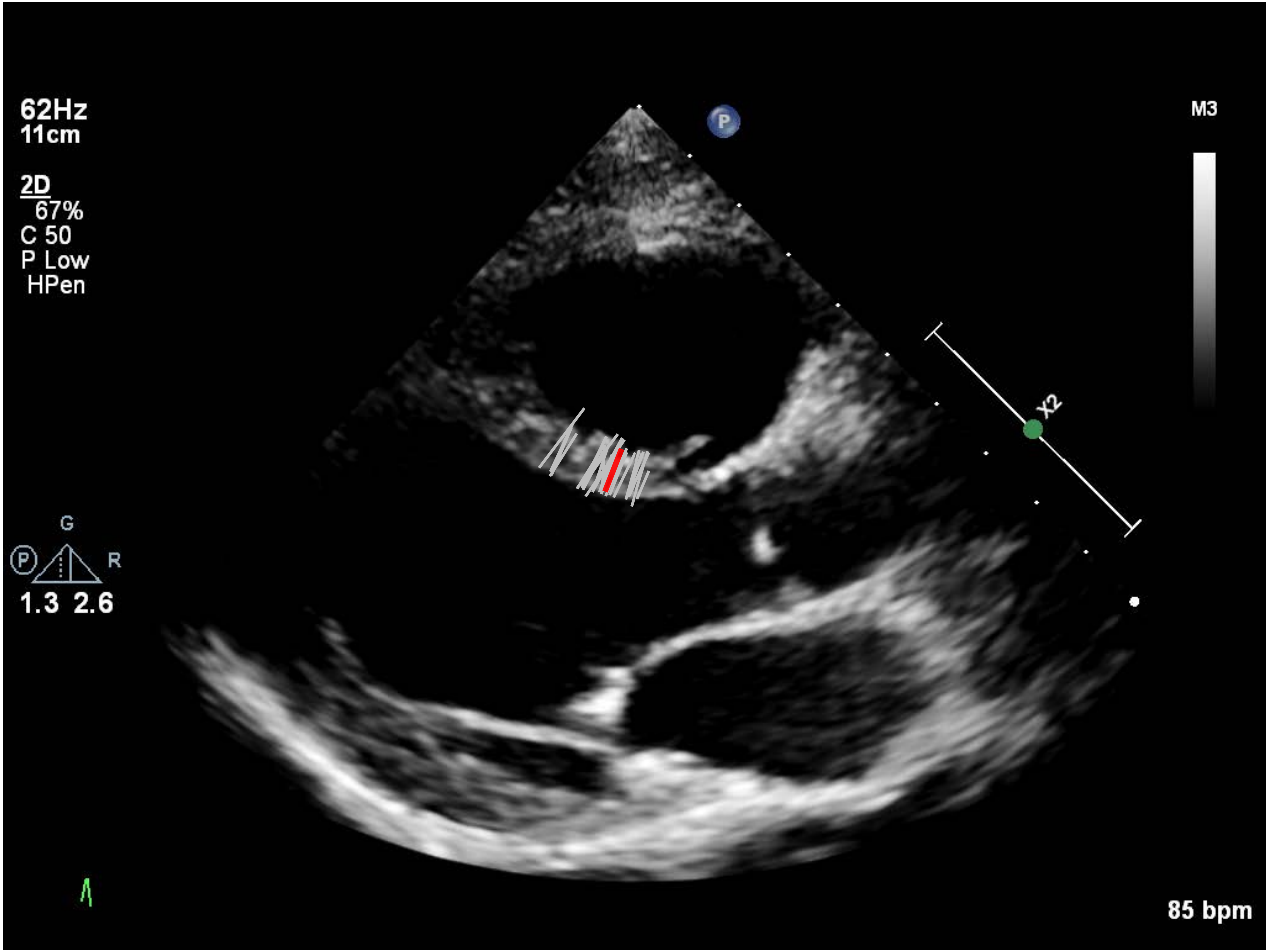

Pt 2 Diastole
Absolute difference between AI and expert consensus: 0.51 mm

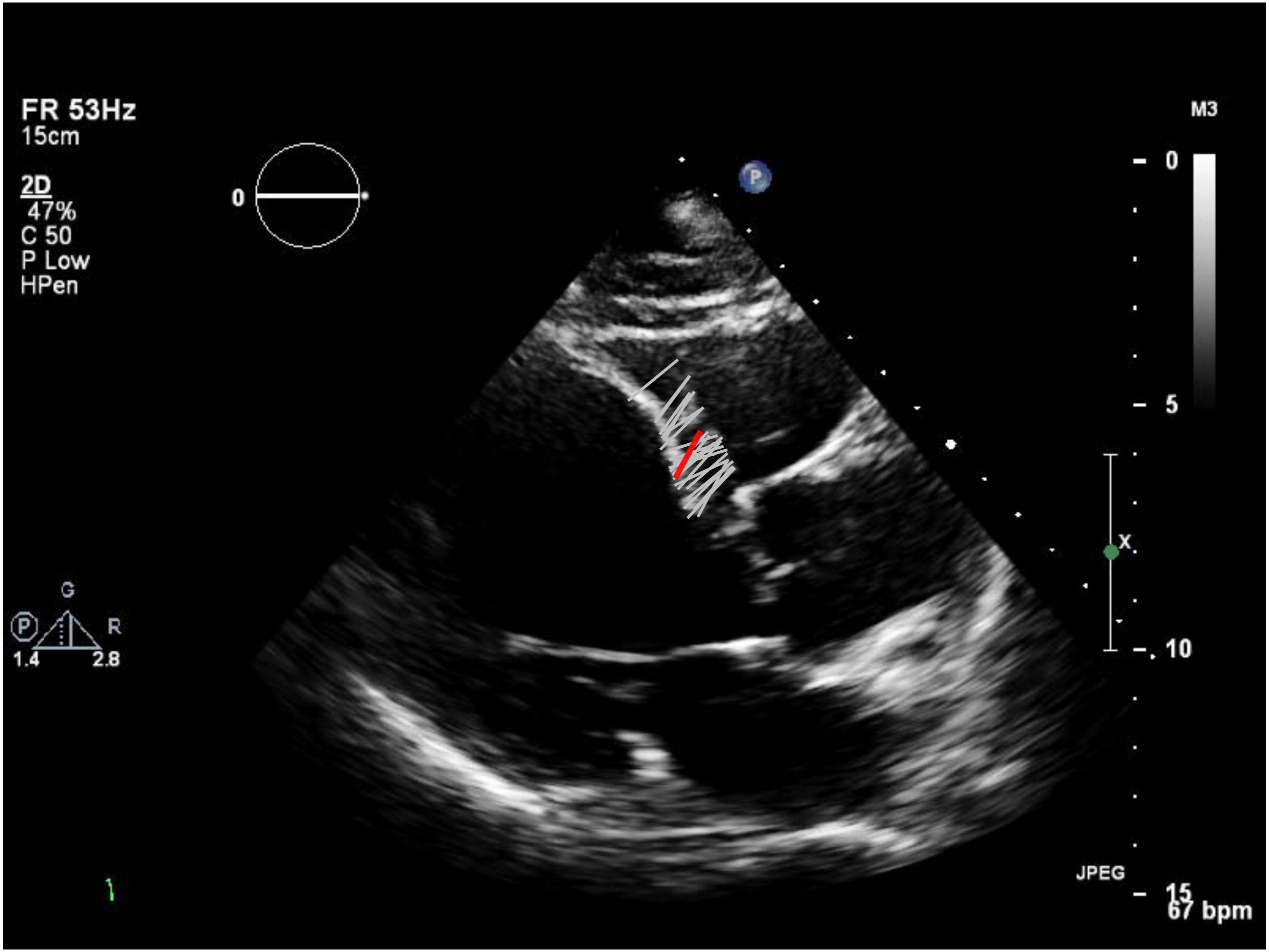

Pt 43 Diastole

Absolute difference between AI and expert consensus: 0.52 mm

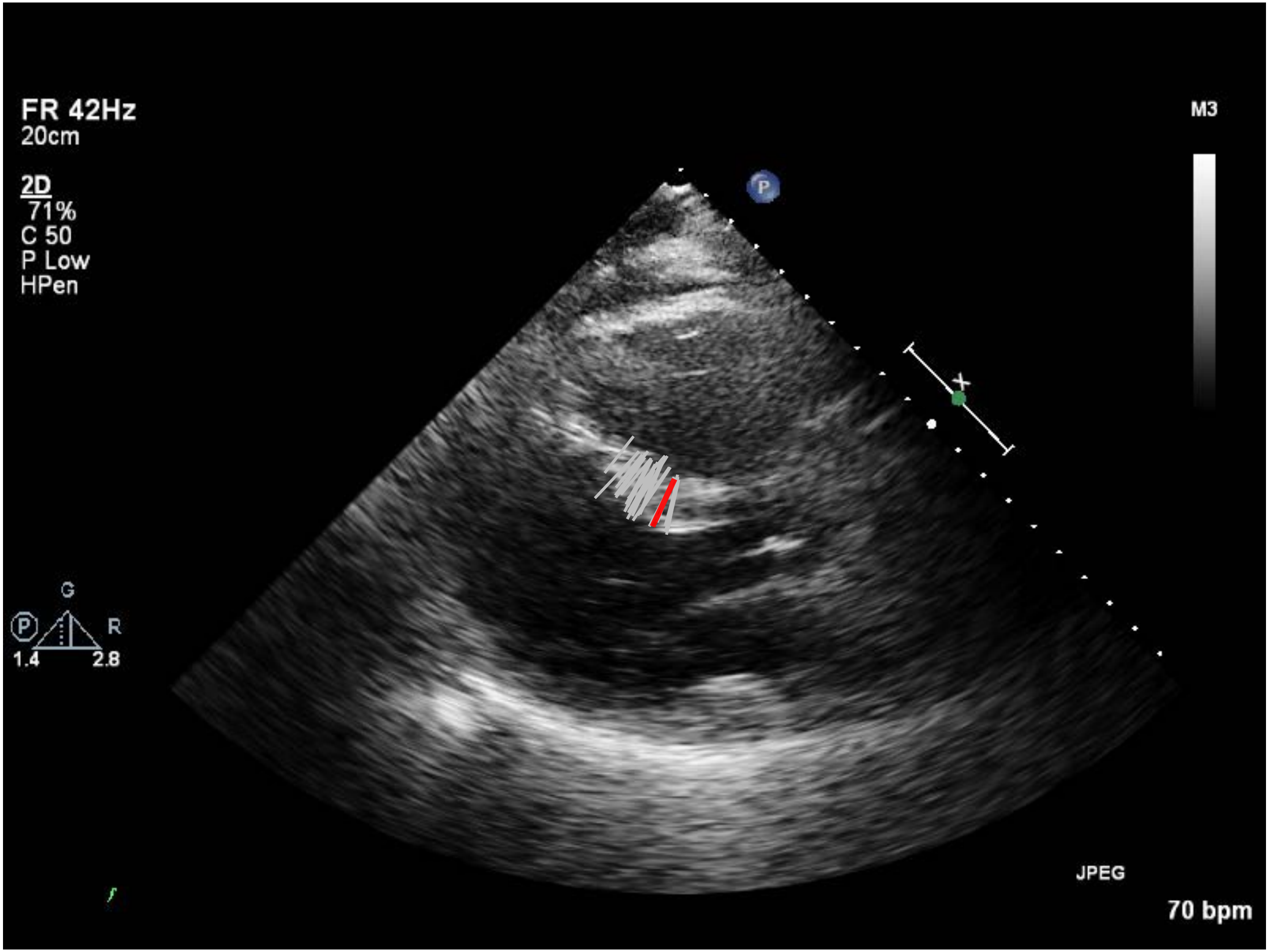

Pt 96 Diastole

Absolute difference between AI and expert consensus: 0.53 mm

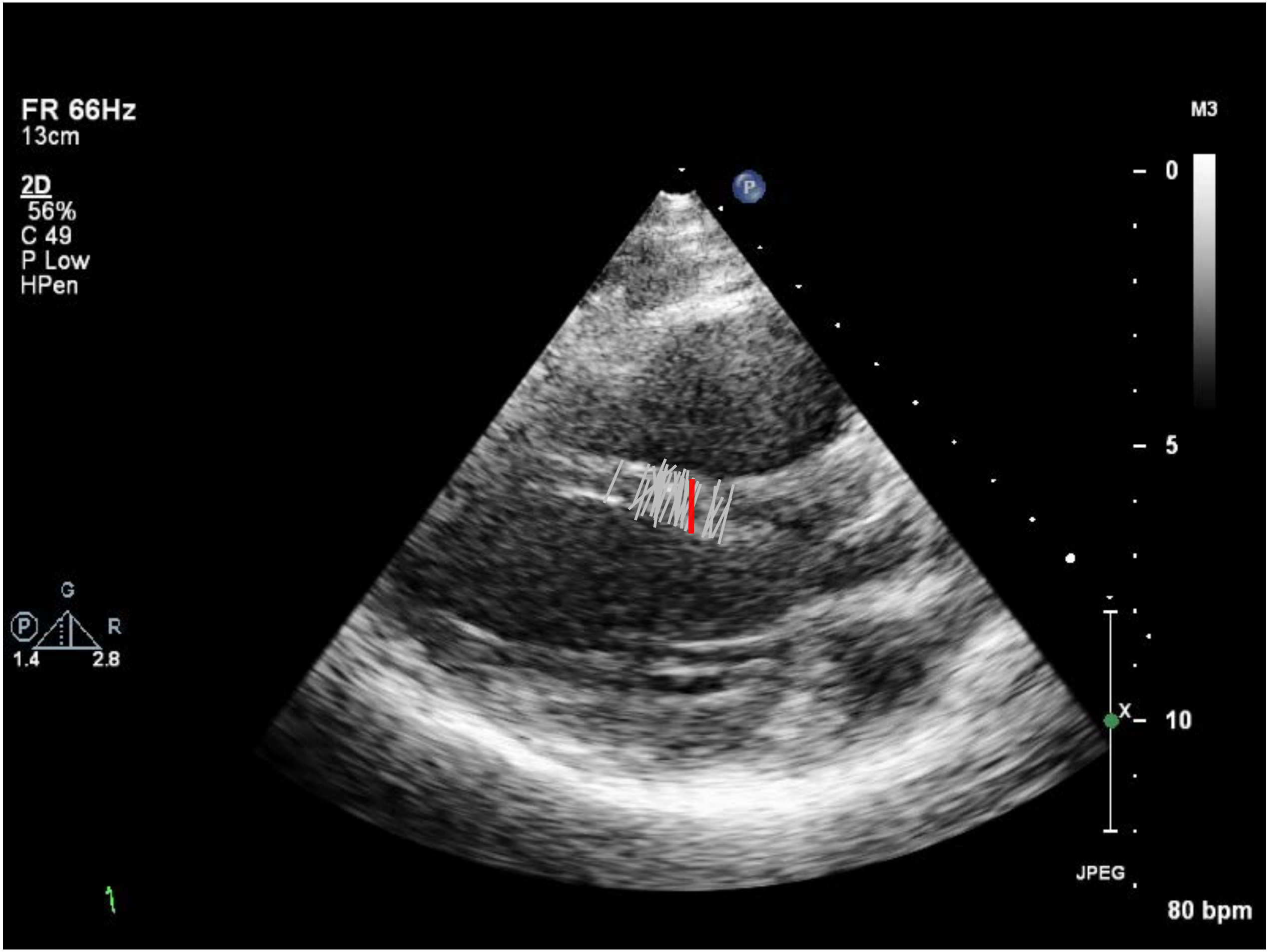

Pt 91 Diastole

Absolute difference between AI and expert consensus: 0.54 mm

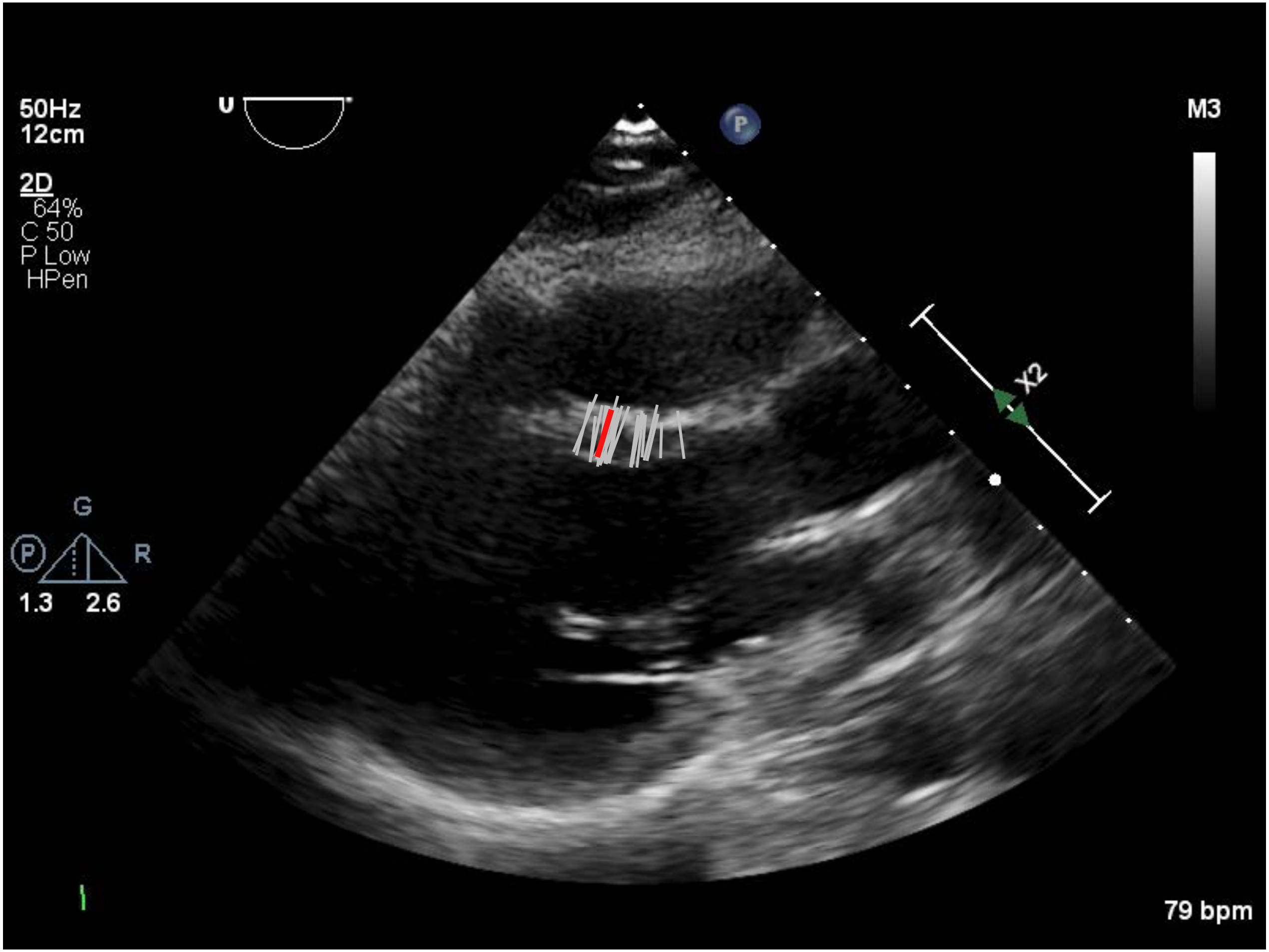

Pt 55 Diastole

Absolute difference between AI and expert consensus: 0.55 mm

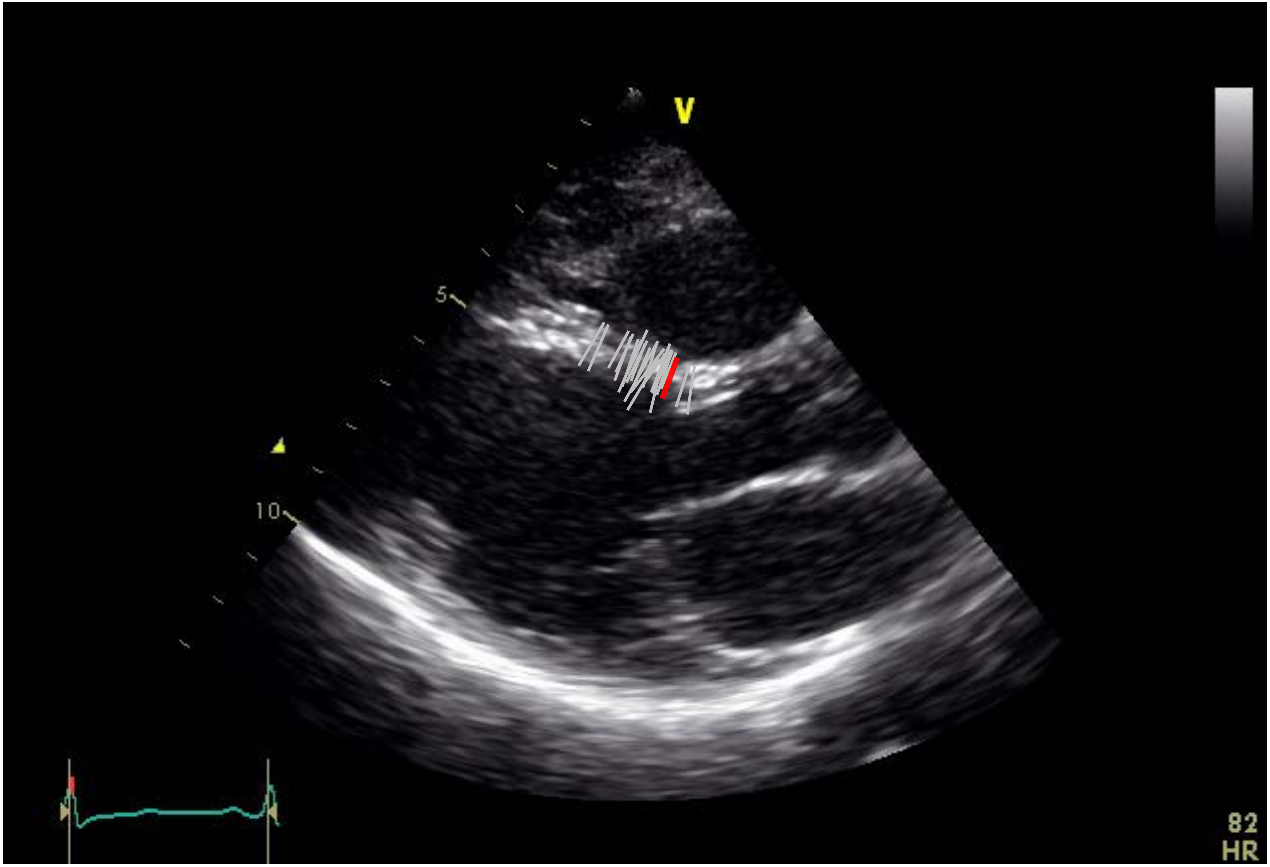

Pt 35 Diastole

Absolute difference between AI and expert consensus: 0.58 mm

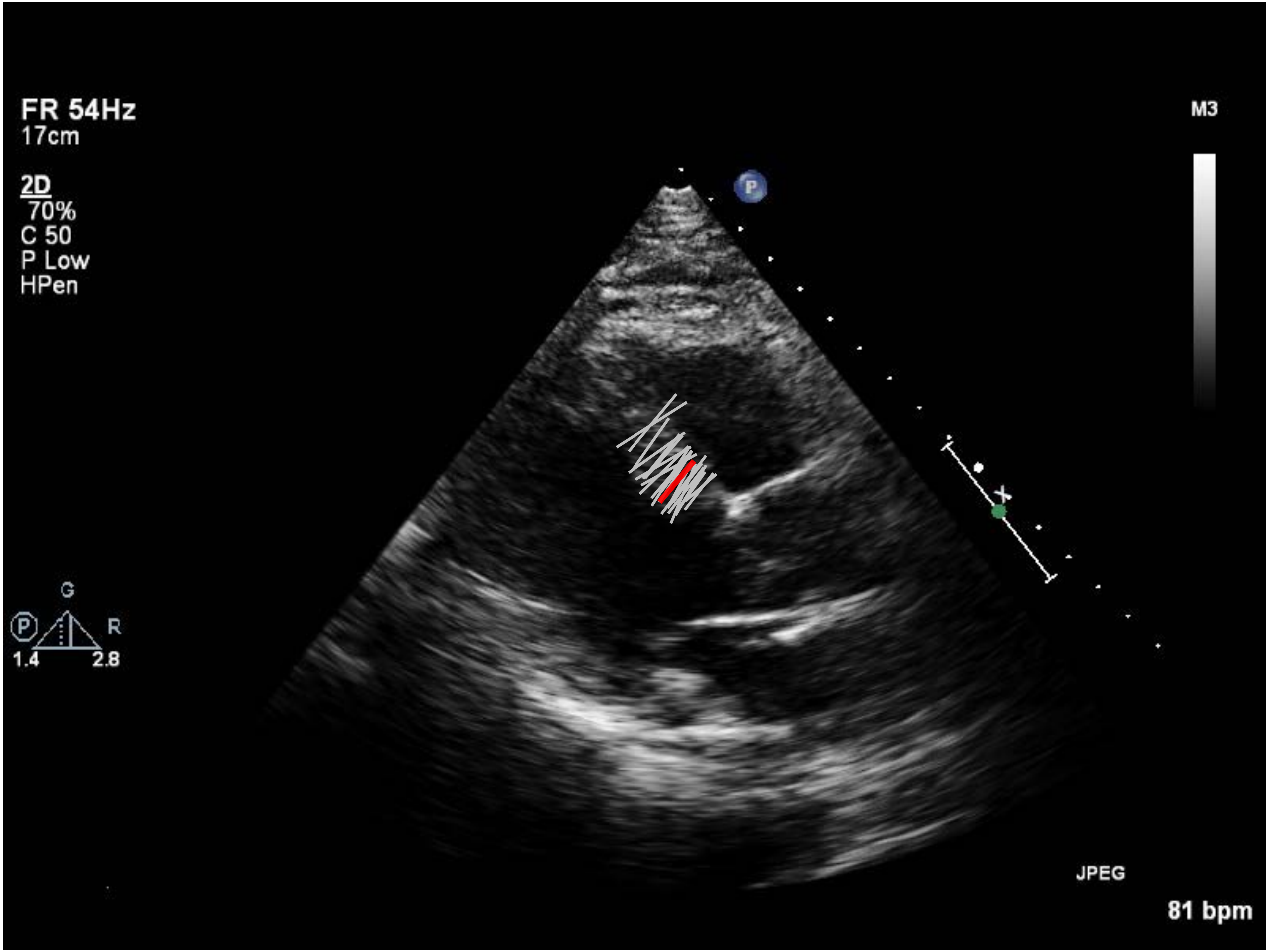

Pt 90 Diastole

Absolute difference between AI and expert consensus: 0.58 mm

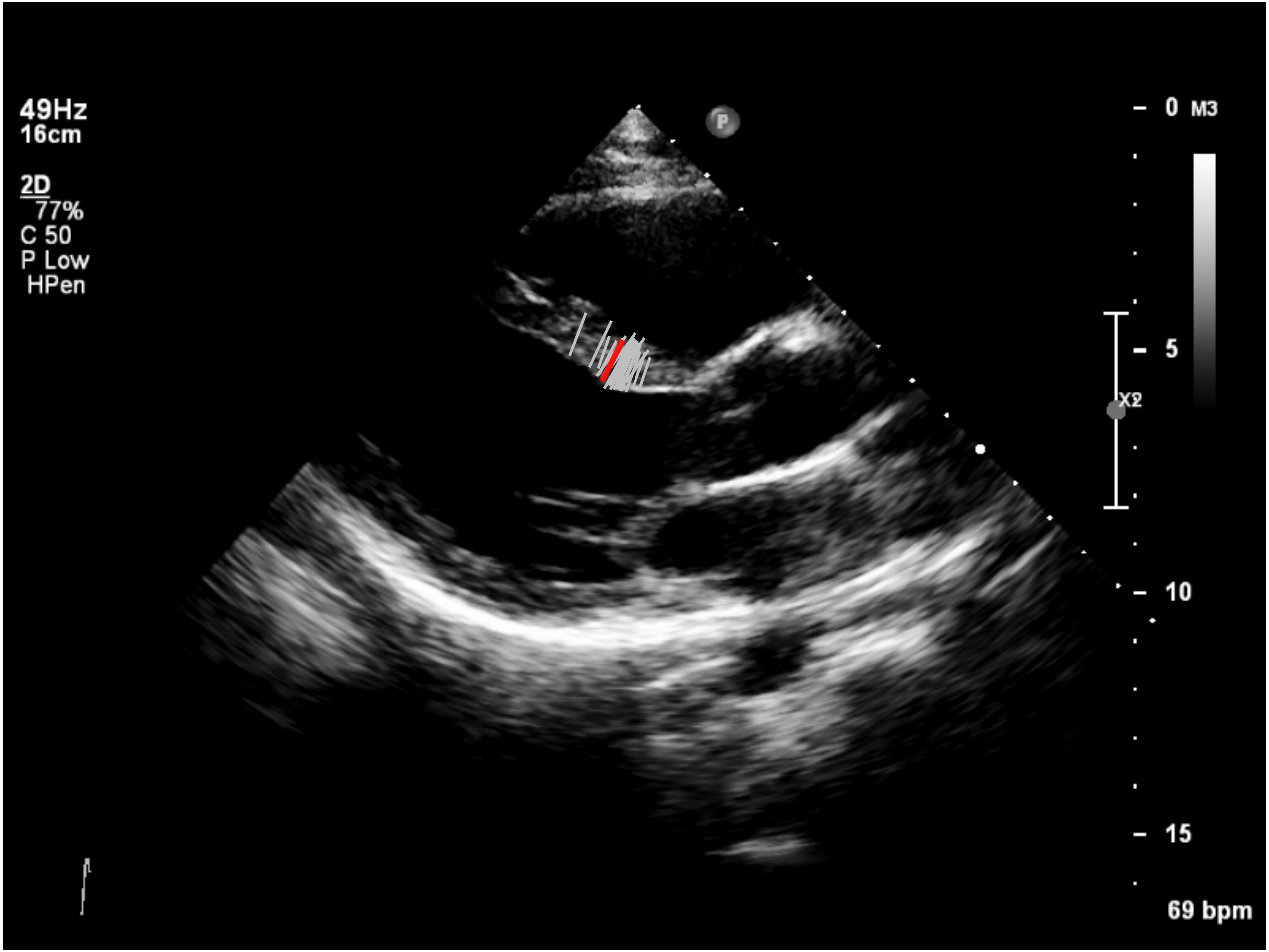

Pt 38 Diastole

Absolute difference between AI and expert consensus: 0.6 mm

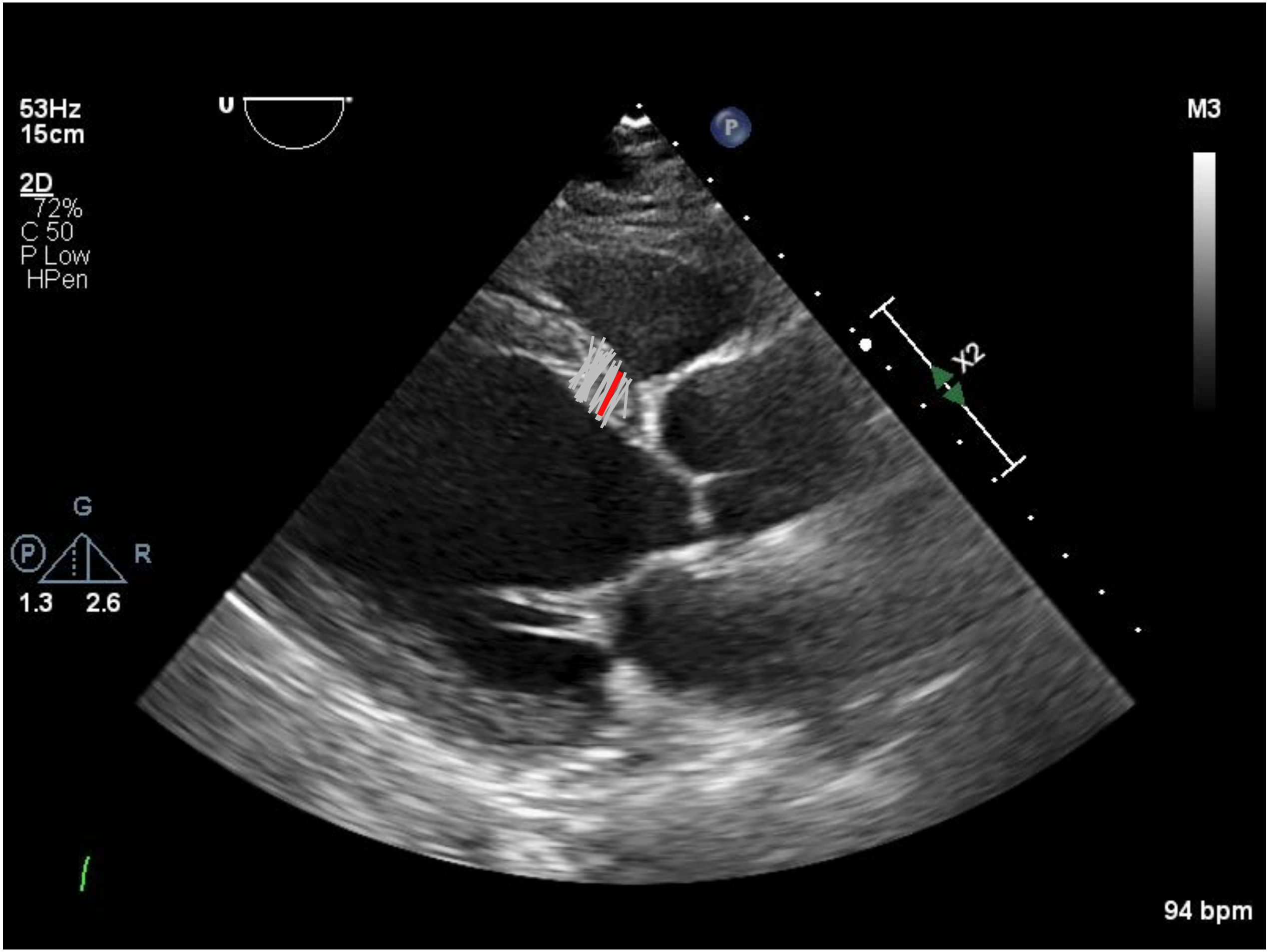

Pt 61 Diastole

Absolute difference between AI and expert consensus: 0.64 mm

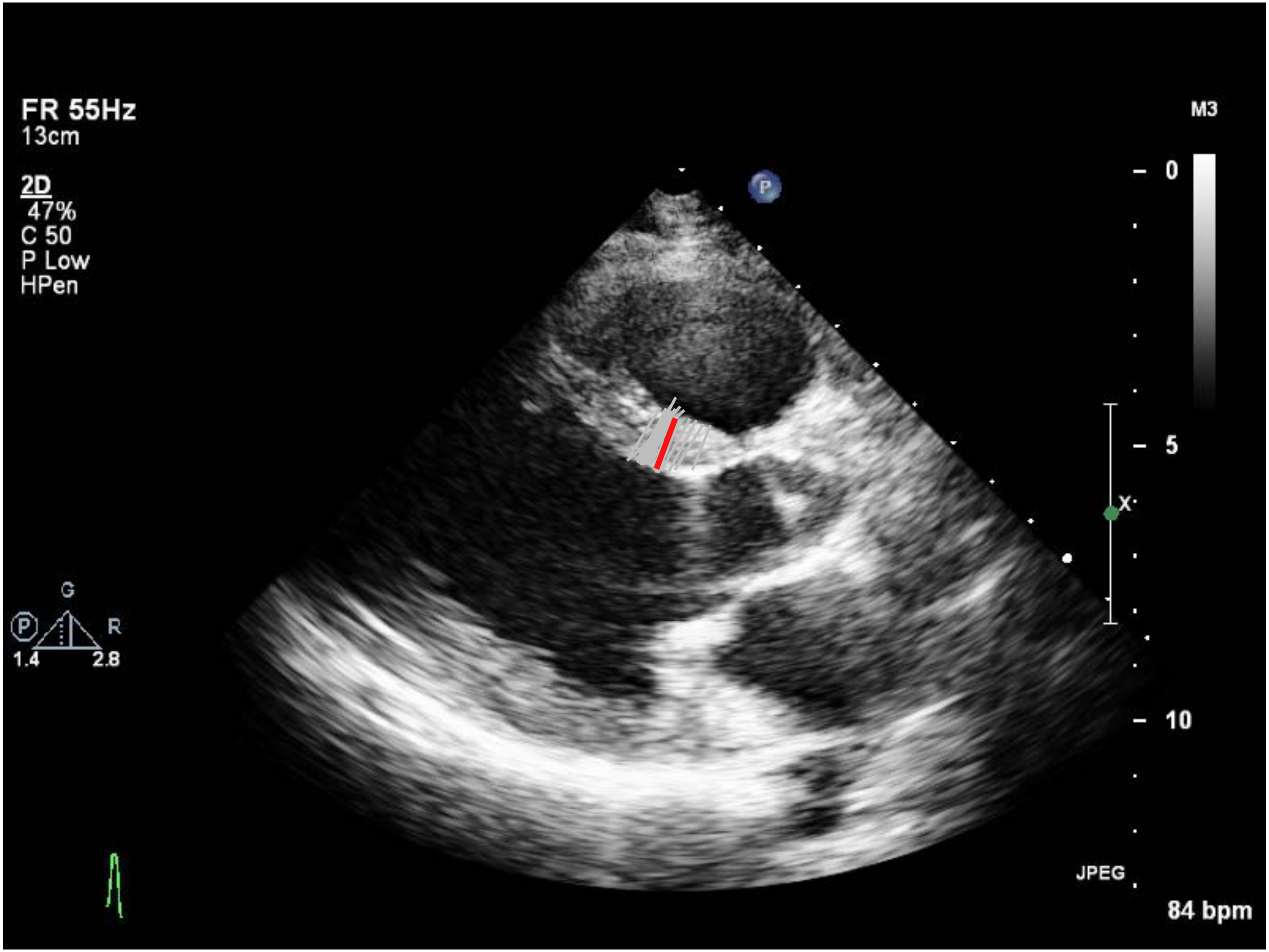

Pt 46 Diastole

Absolute difference between AI and expert consensus: 0.68 mm

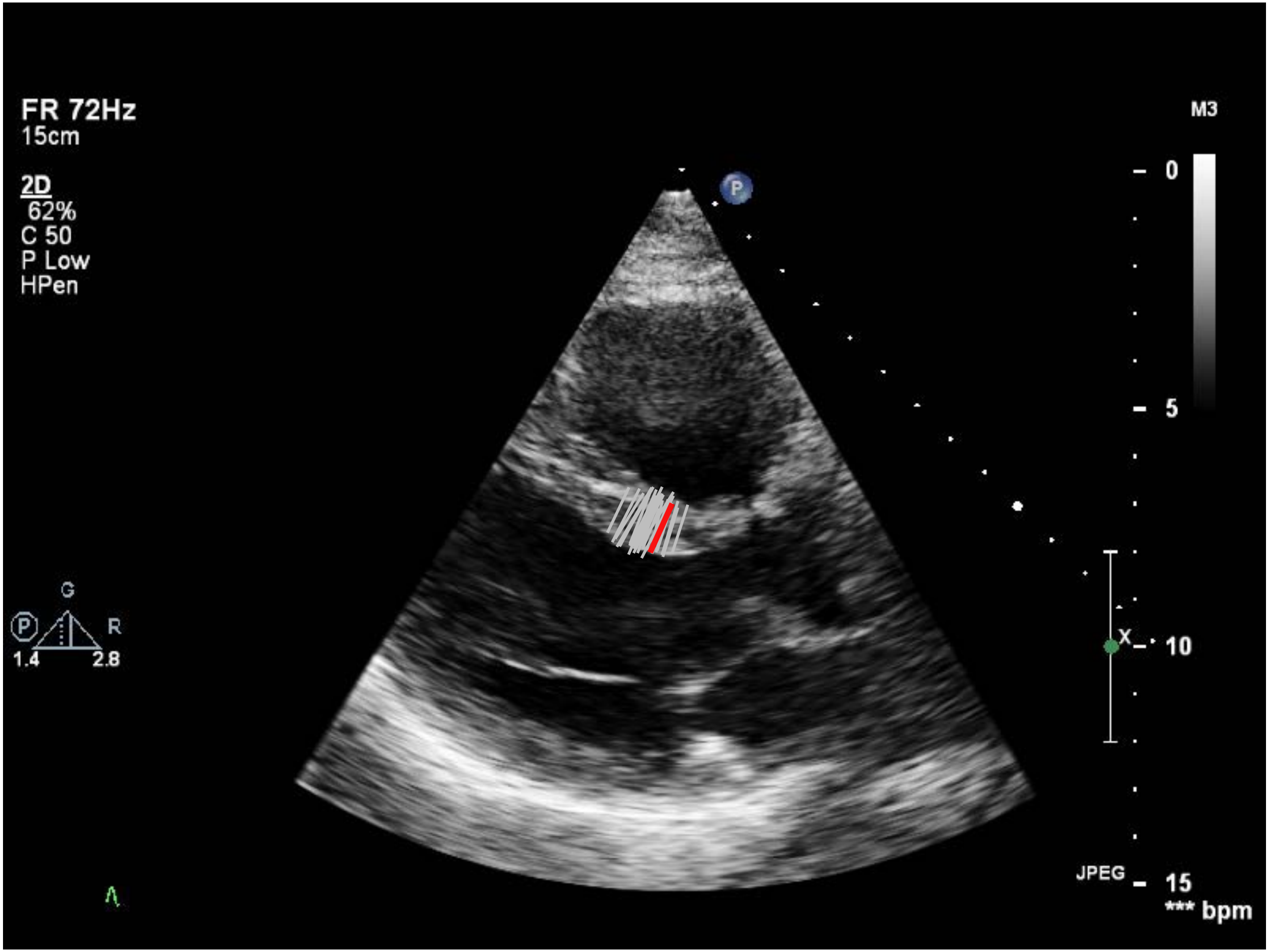

Pt 94 Diastole

Absolute difference between AI and expert consensus: 0.73 mm

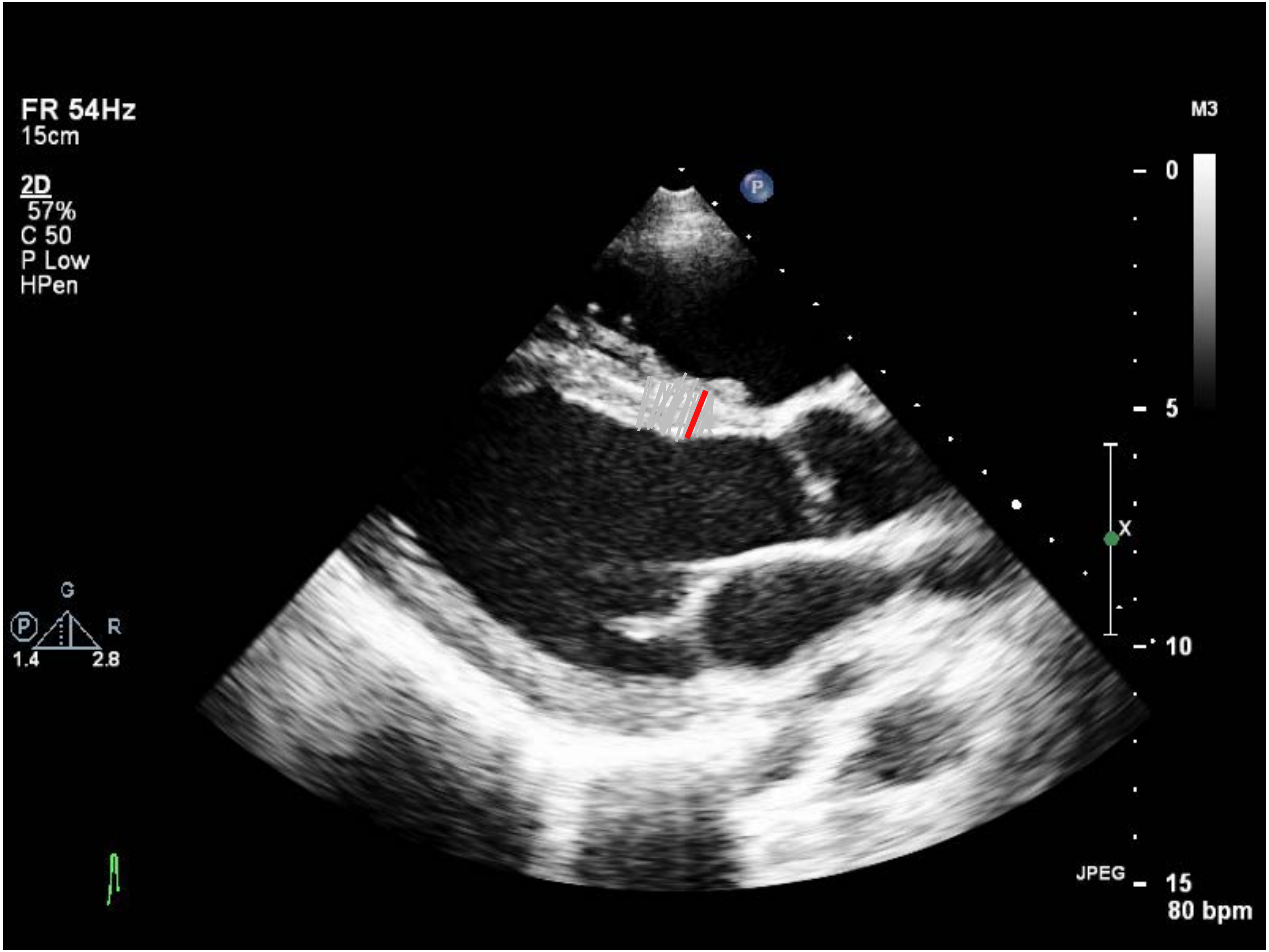

Pt 82 Diastole

Absolute difference between AI and expert consensus: 0.74 mm

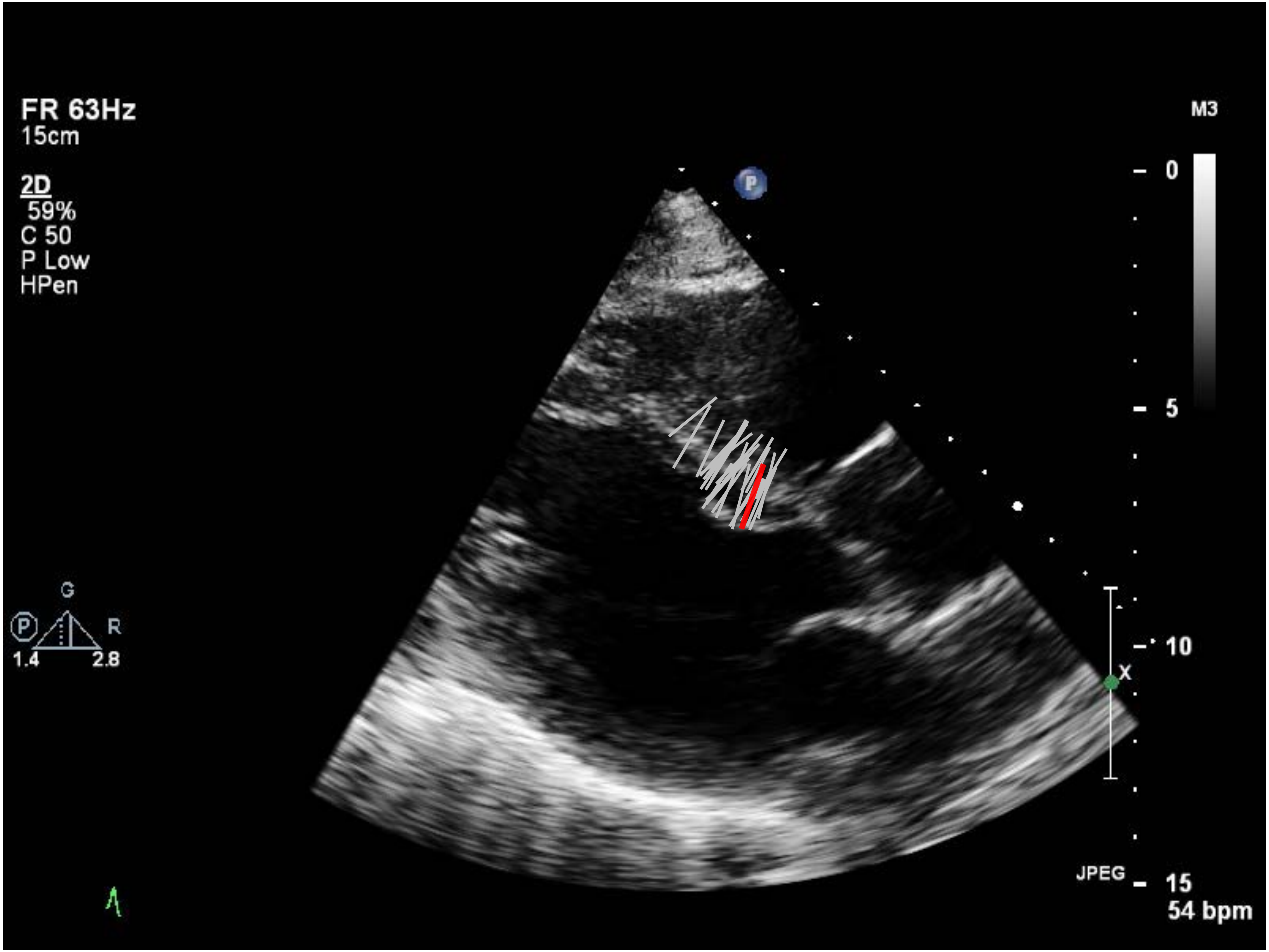

Pt 69 Diastole

Absolute difference between AI and expert consensus: 0.74 mm

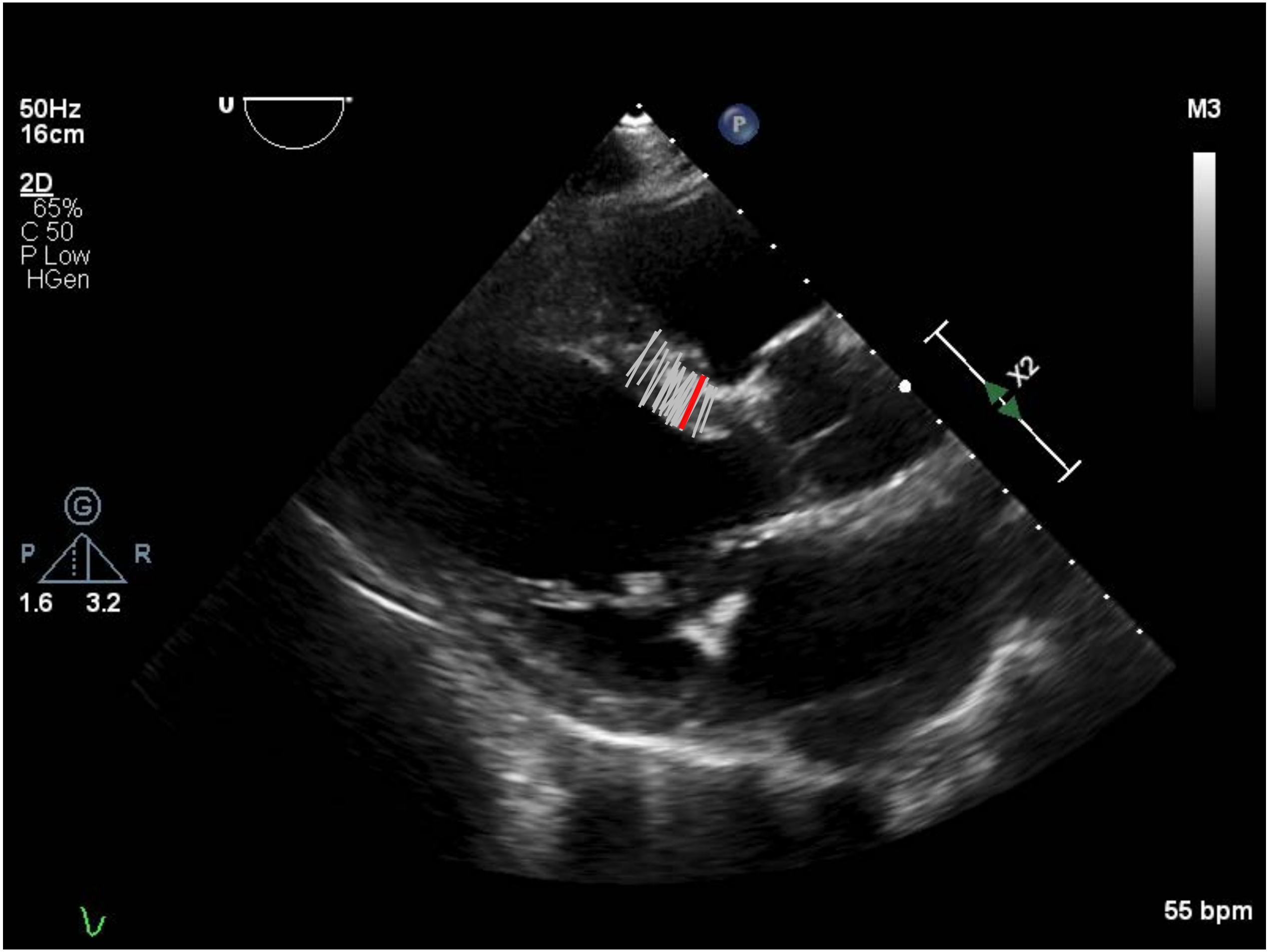

Pt 78 Diastole

Absolute difference between AI and expert consensus: 0.75 mm

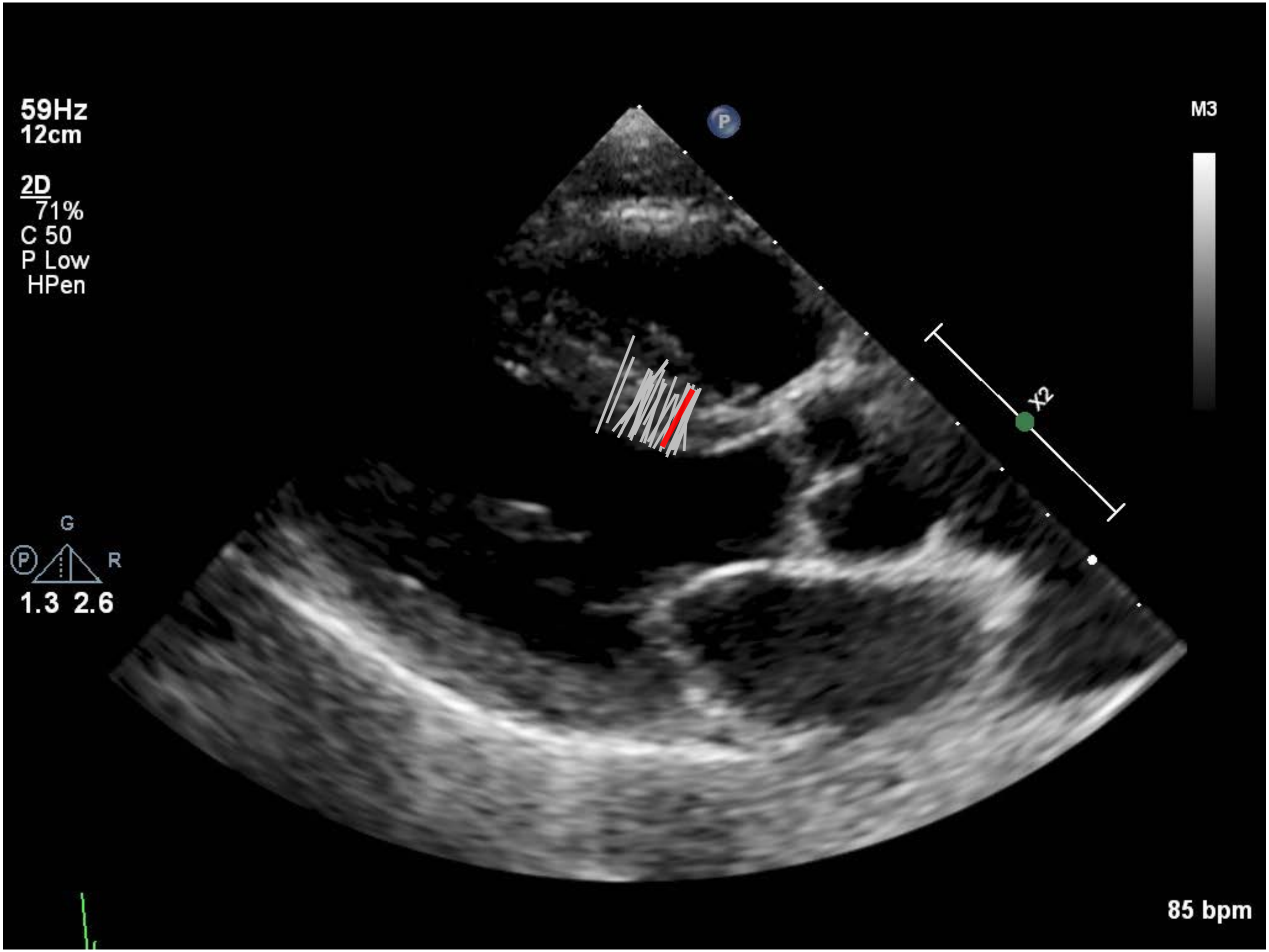

Pt 75 Diastole
Absolute difference between AI and expert consensus: 0.8 mm

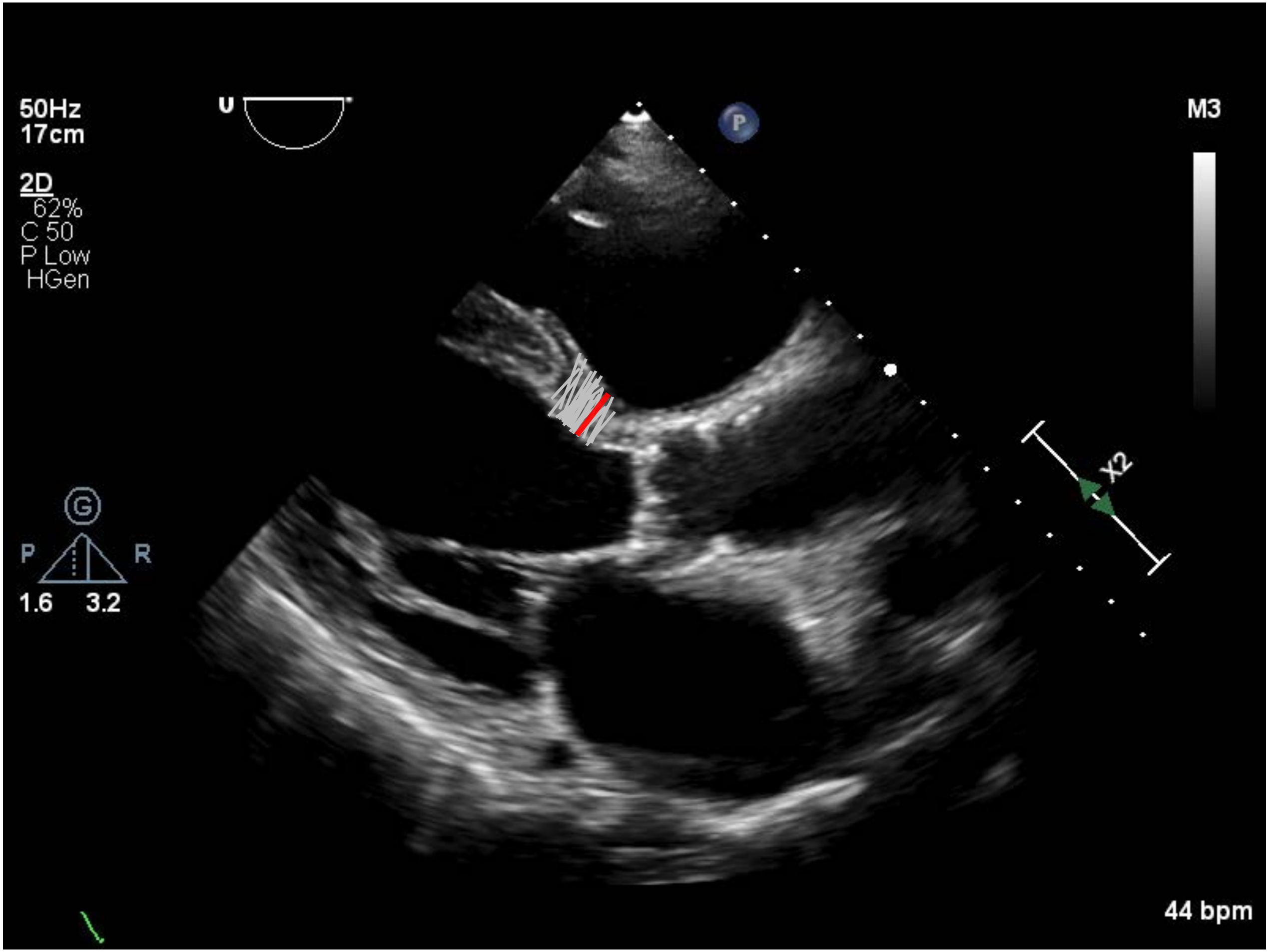

Pt 52 Diastole

Absolute difference between AI and expert consensus: 0.86 mm

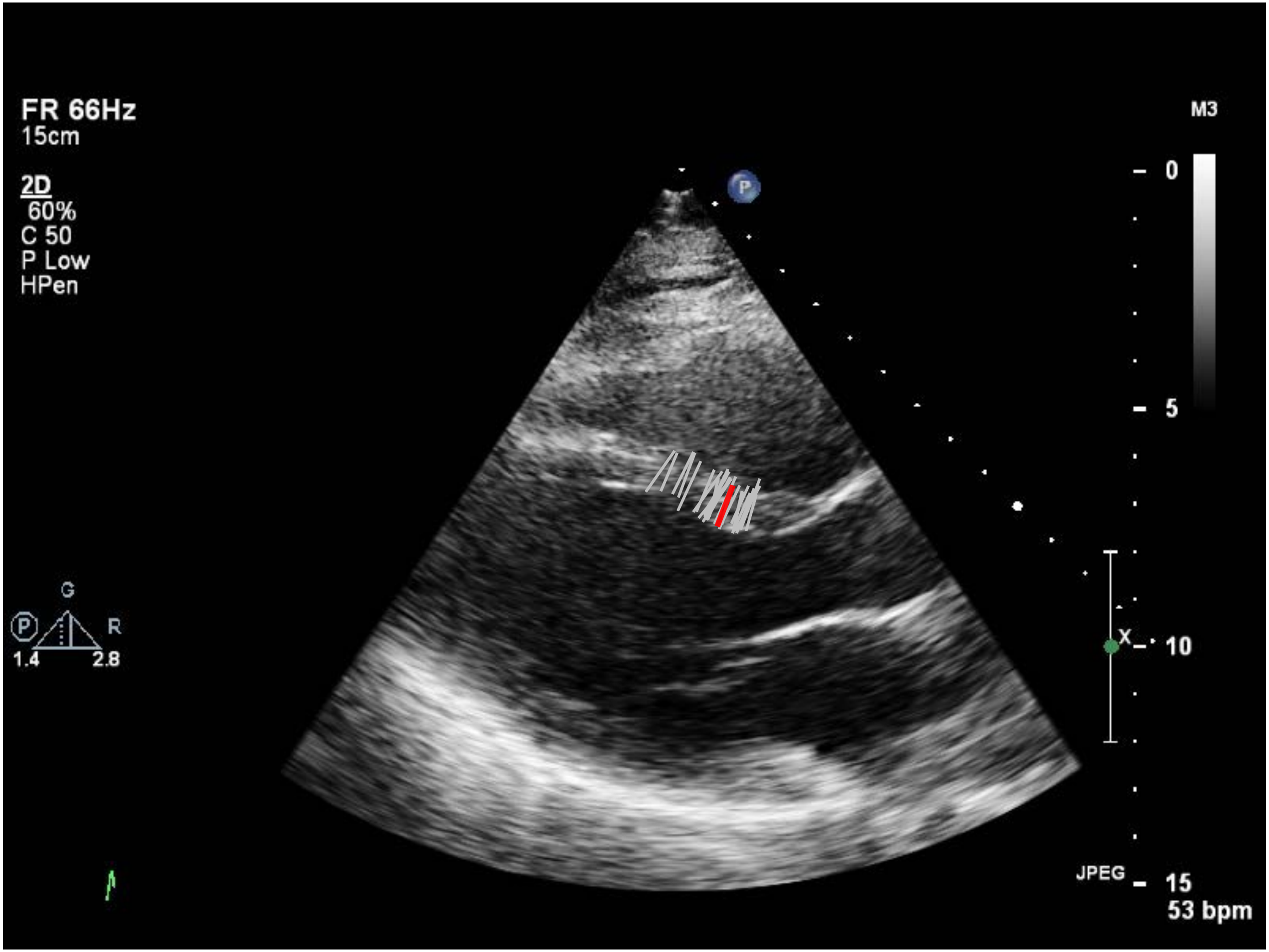

Pt 51 Diastole

Absolute difference between AI and expert consensus: 0.87 mm

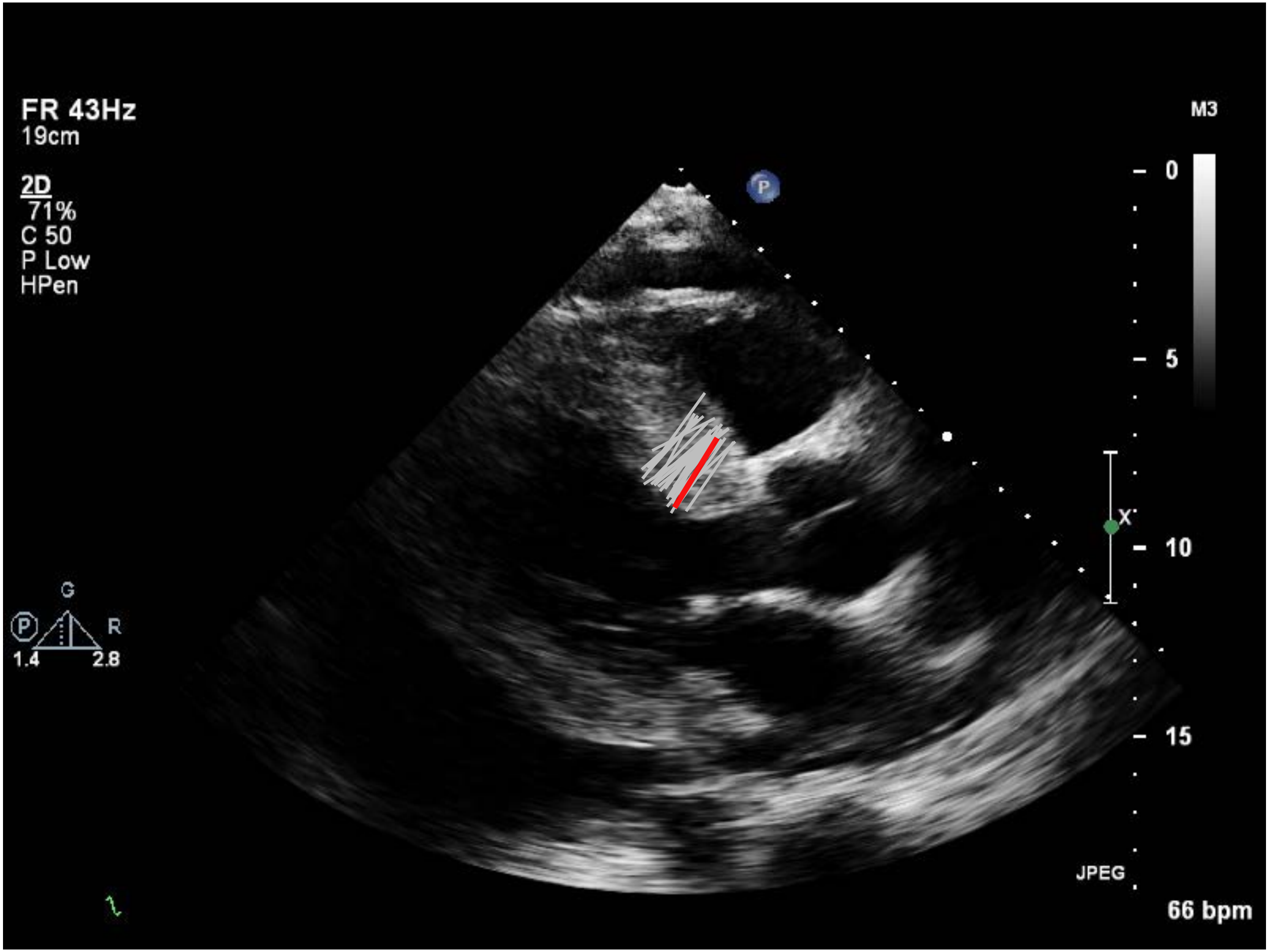

Pt 100 Diastole

Absolute difference between AI and expert consensus: 0.88 mm

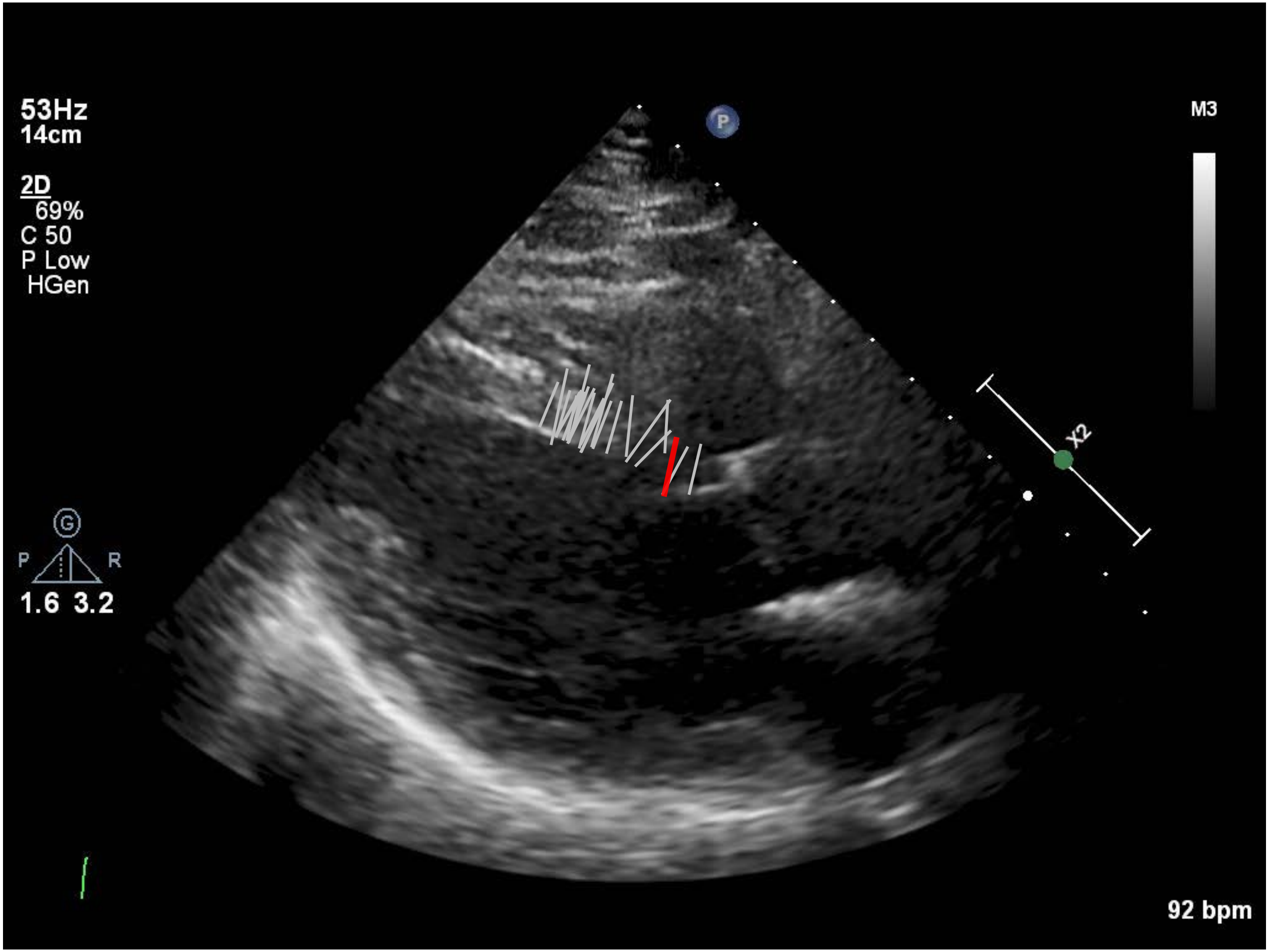

Pt 70 Diastole
Absolute difference between AI and expert consensus: 0.89 mm

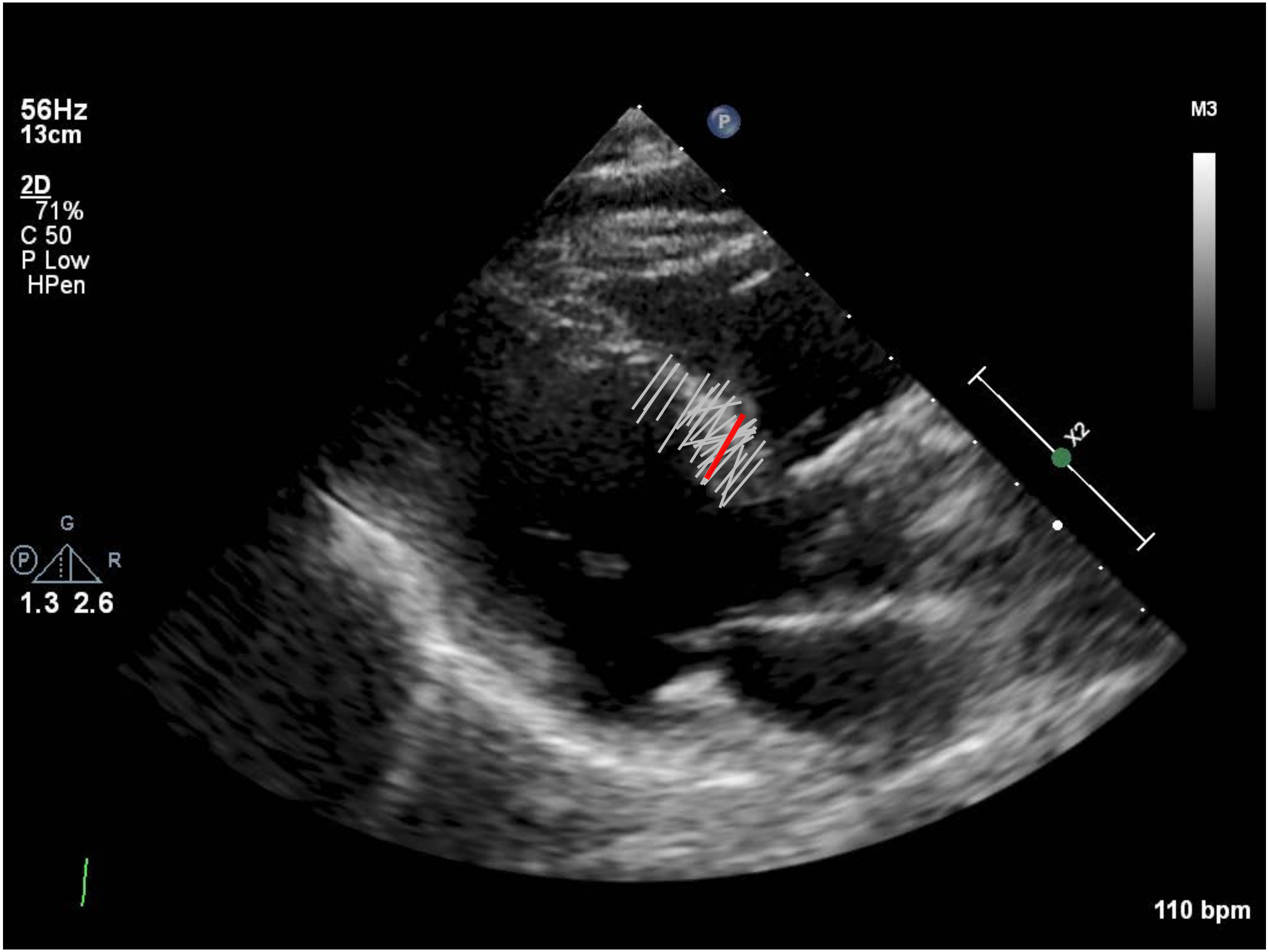

Pt 26 Diastole
Absolute difference between AI and expert consensus: 0.92 mm

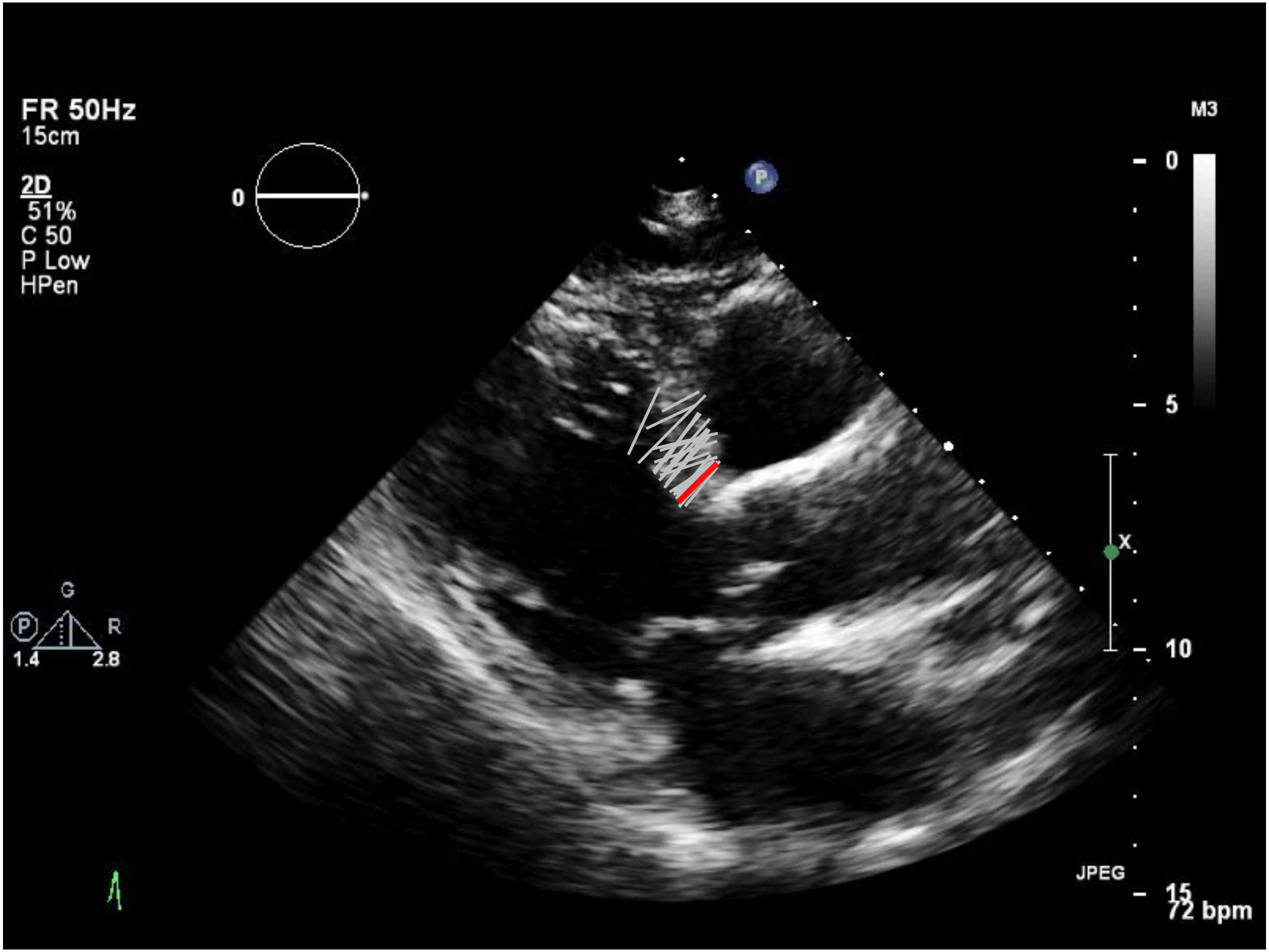

Pt 30 Diastole

Absolute difference between AI and expert consensus: 0.93 mm

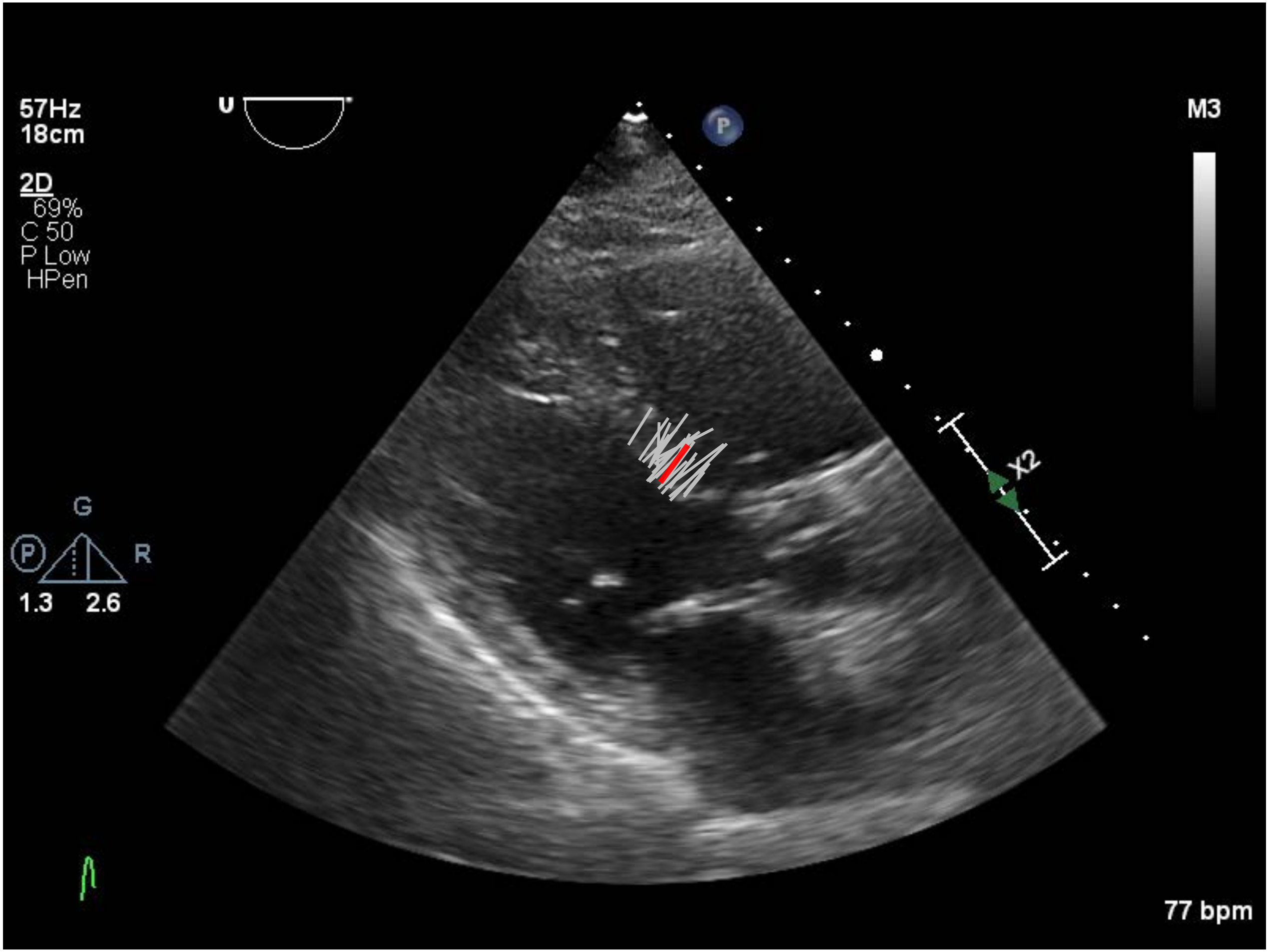

Pt 15 Diastole

Absolute difference between AI and expert consensus: 0.93 mm

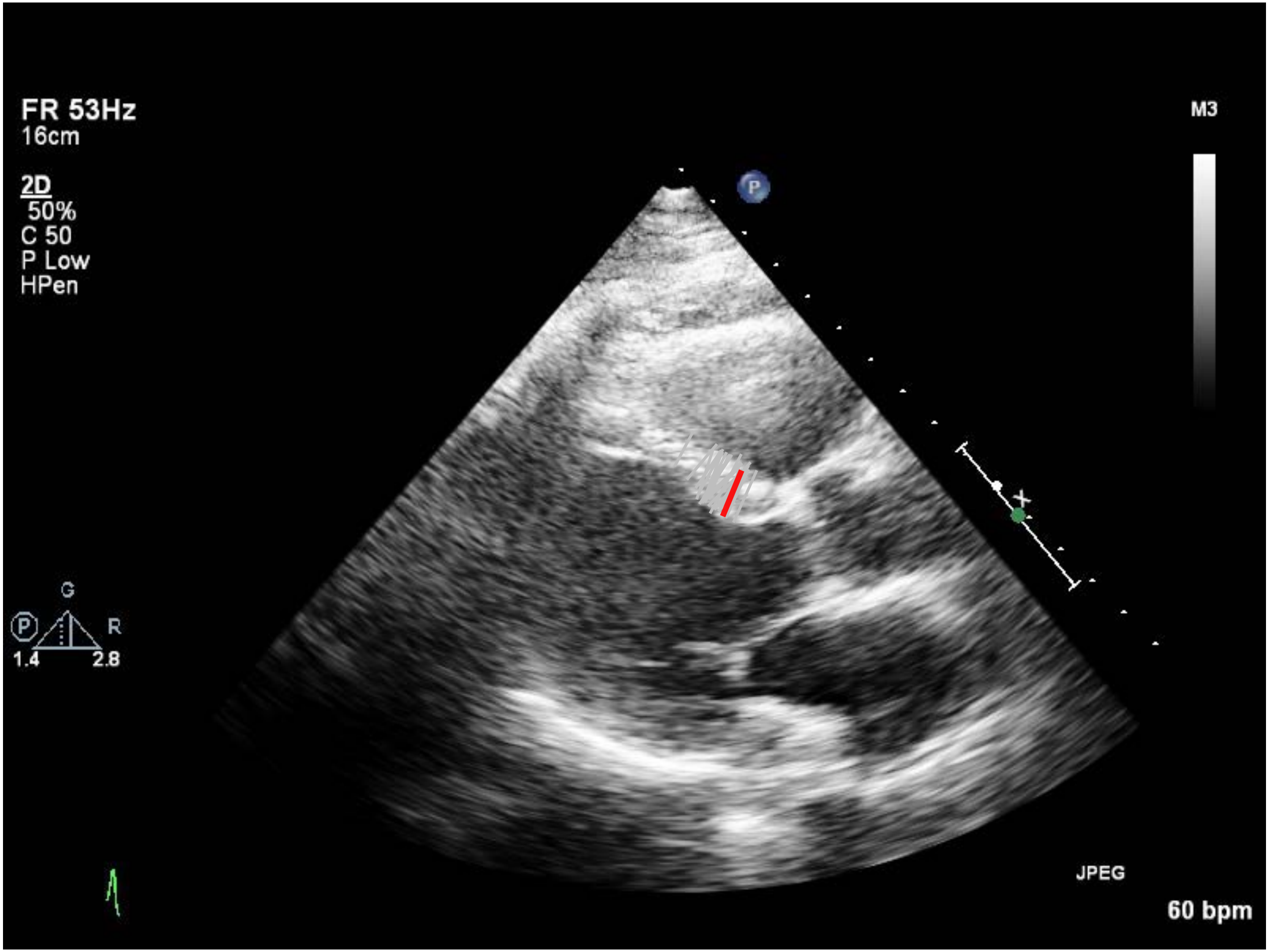

Pt 47 Diastole

Absolute difference between AI and expert consensus: 0.94 mm

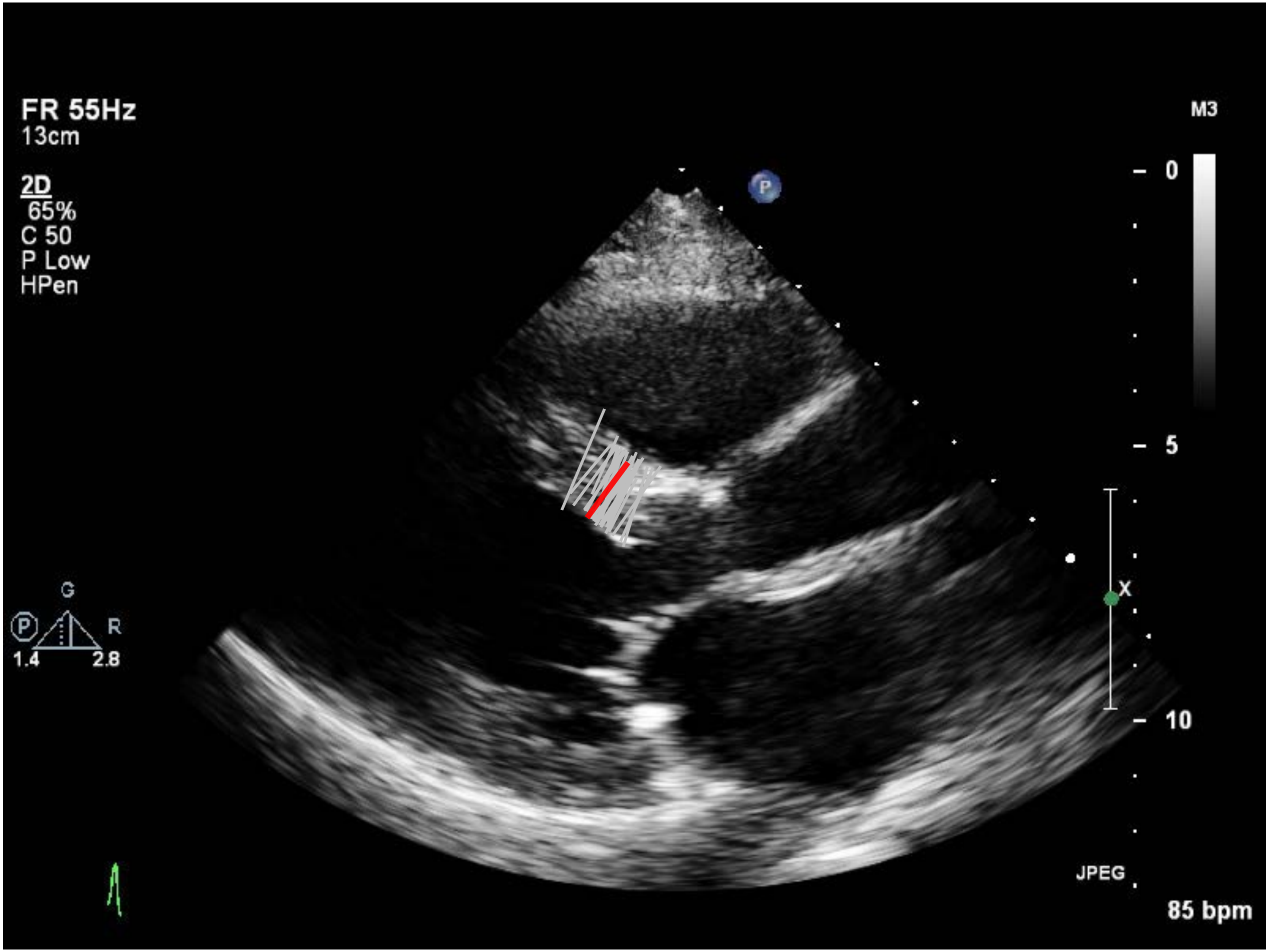

Pt 73 Diastole

Absolute difference between AI and expert consensus: 0.96 mm

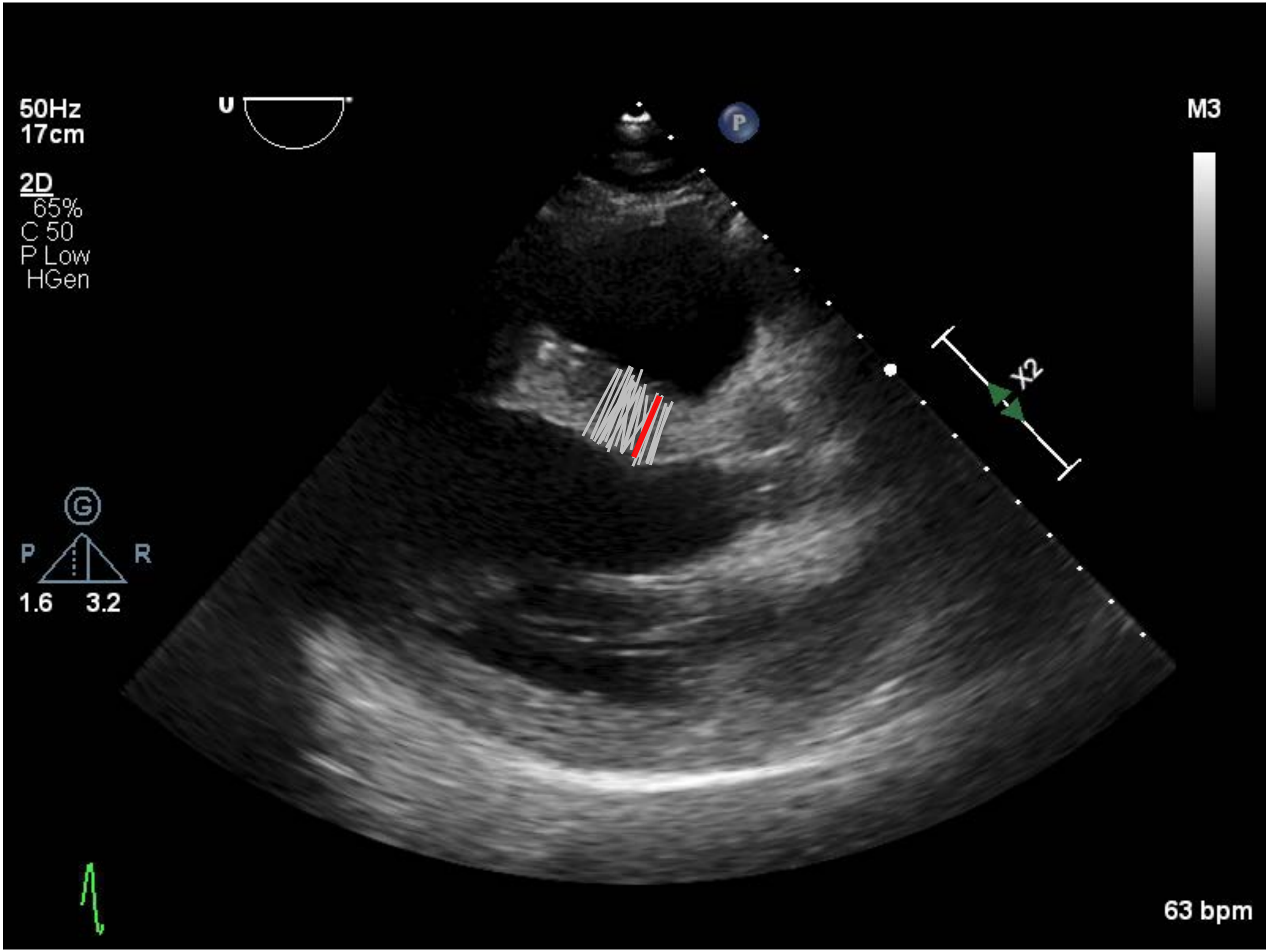

Pt 68 Diastole

Absolute difference between AI and expert consensus: 0.98 mm

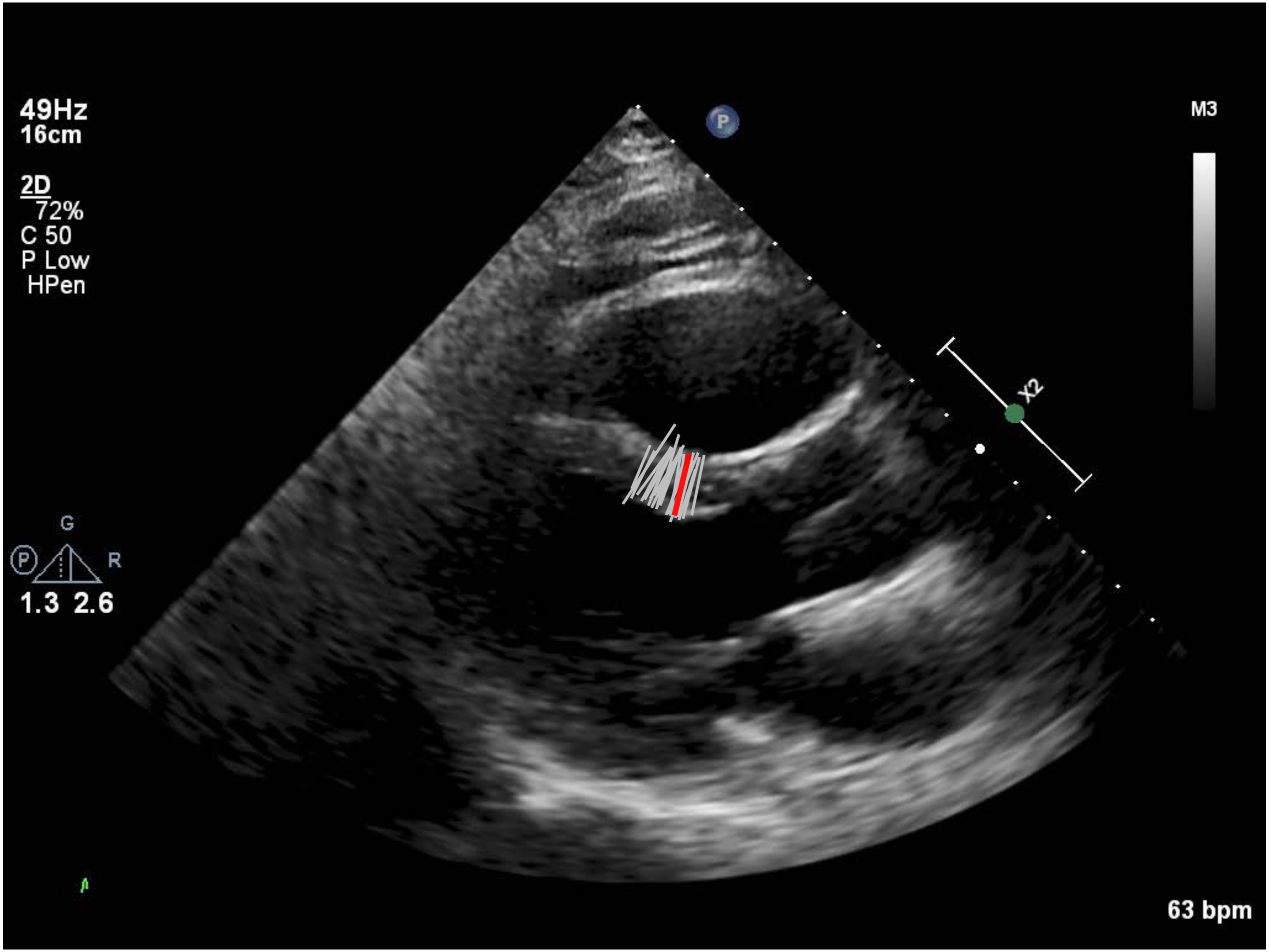

Pt 24 Diastole
Absolute difference between AI and expert consensus: 0.99 mm

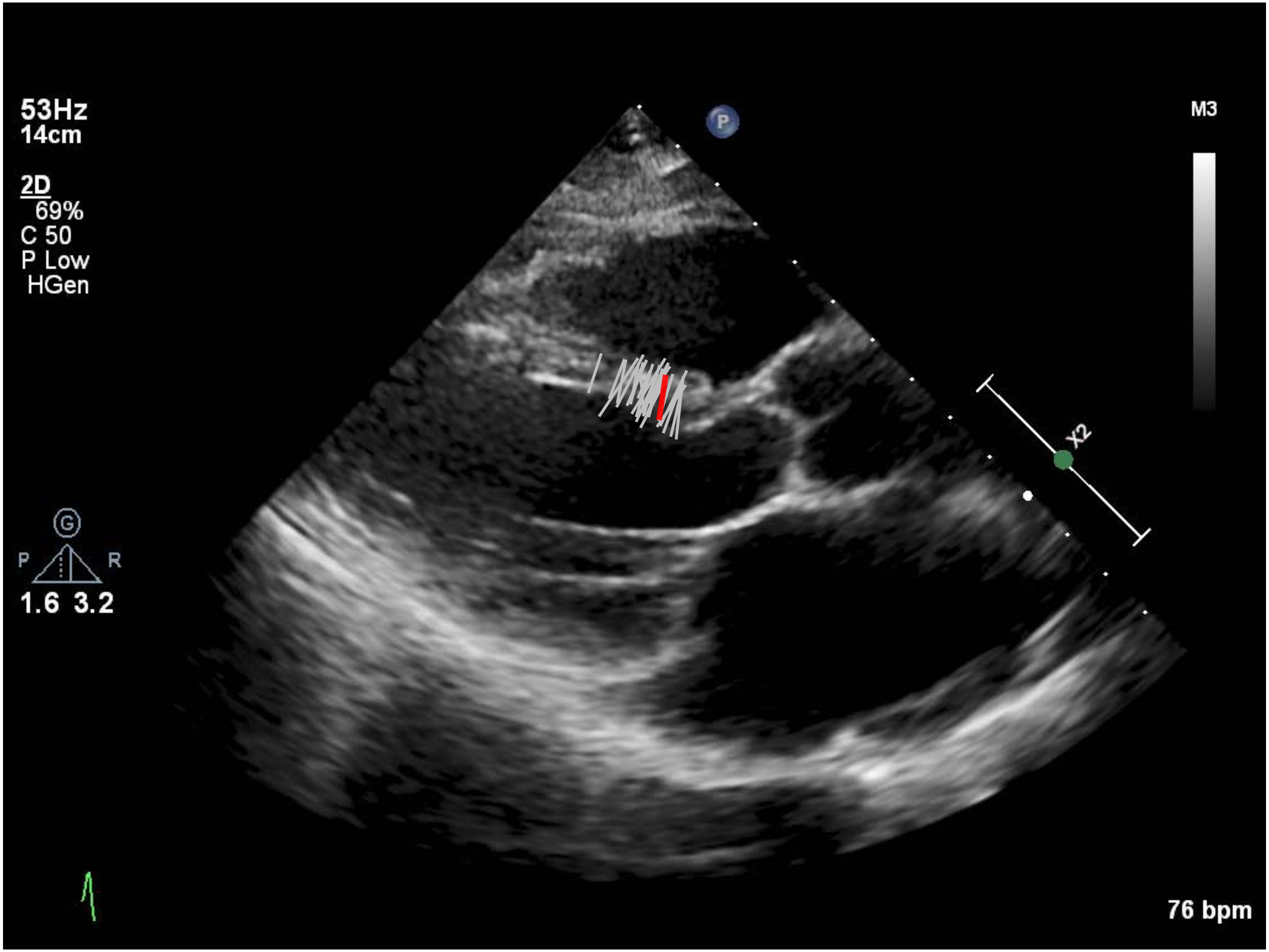

Pt 87 Diastole

Absolute difference between AI and expert consensus: 1.1 mm

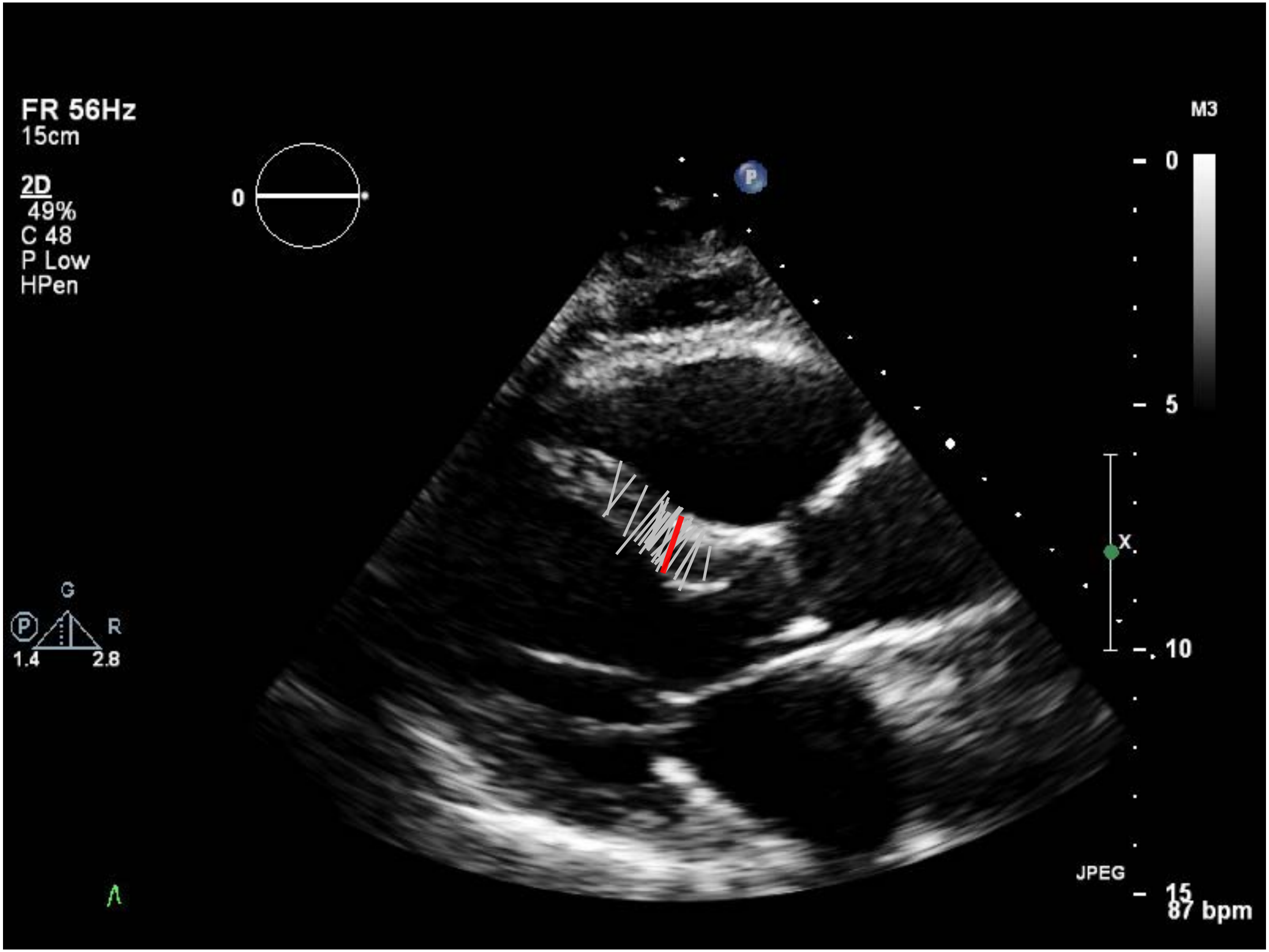

Pt 21 Diastole

Absolute difference between AI and expert consensus: 1.1 mm

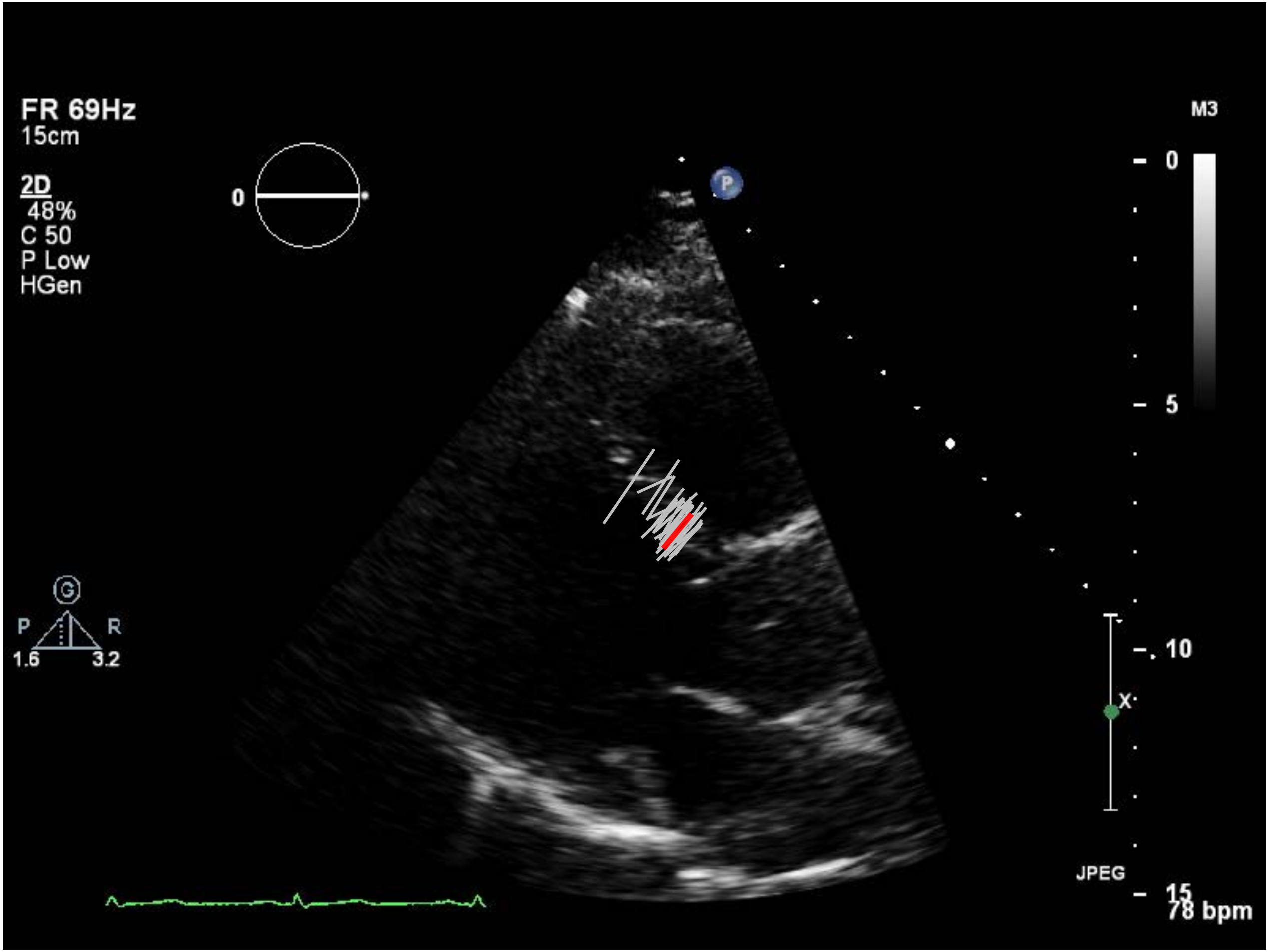

Pt 59 Diastole

Absolute difference between AI and expert consensus: 1.1 mm

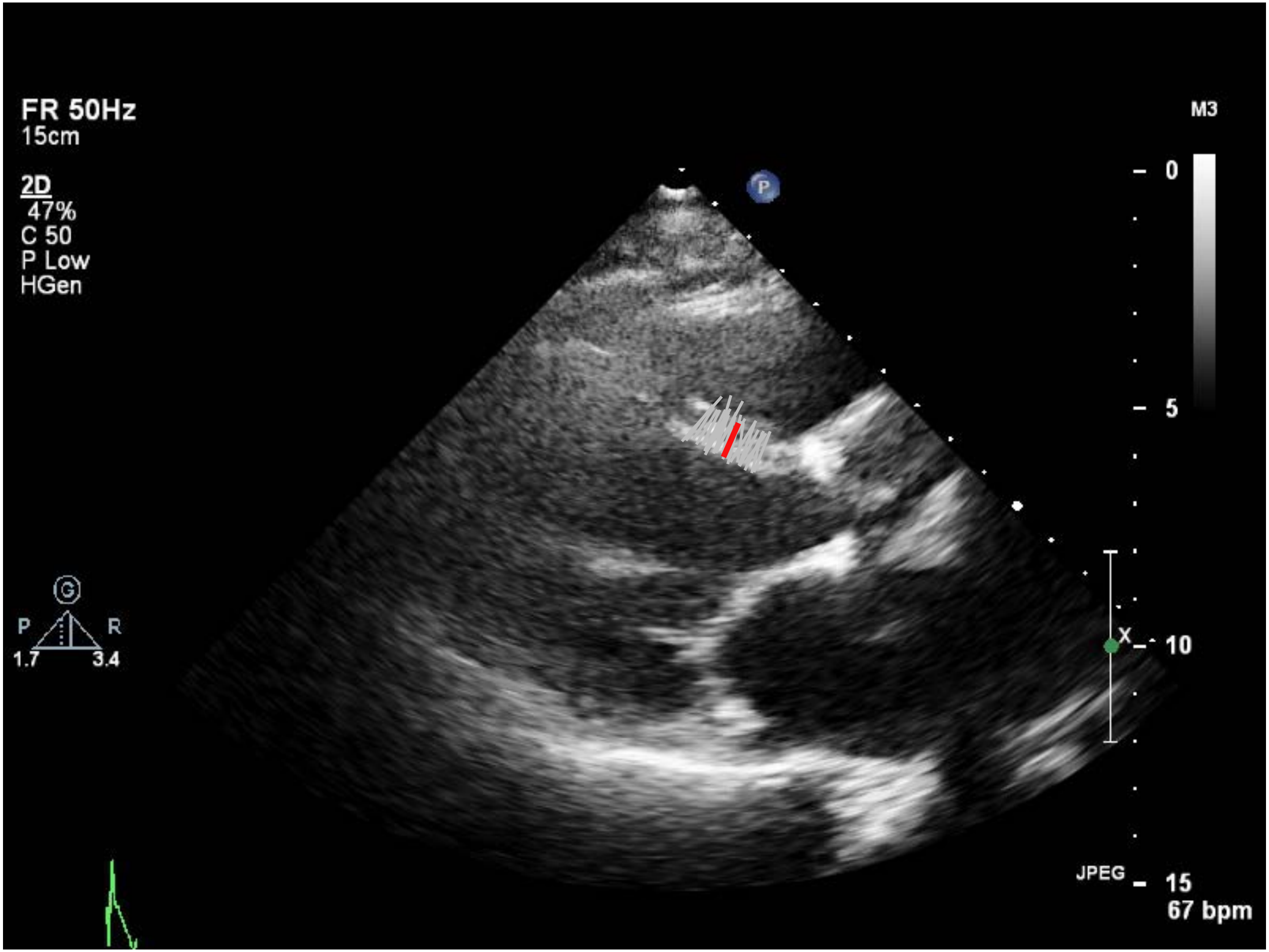

Pt 72 Diastole

Absolute difference between AI and expert consensus: 1.2 mm

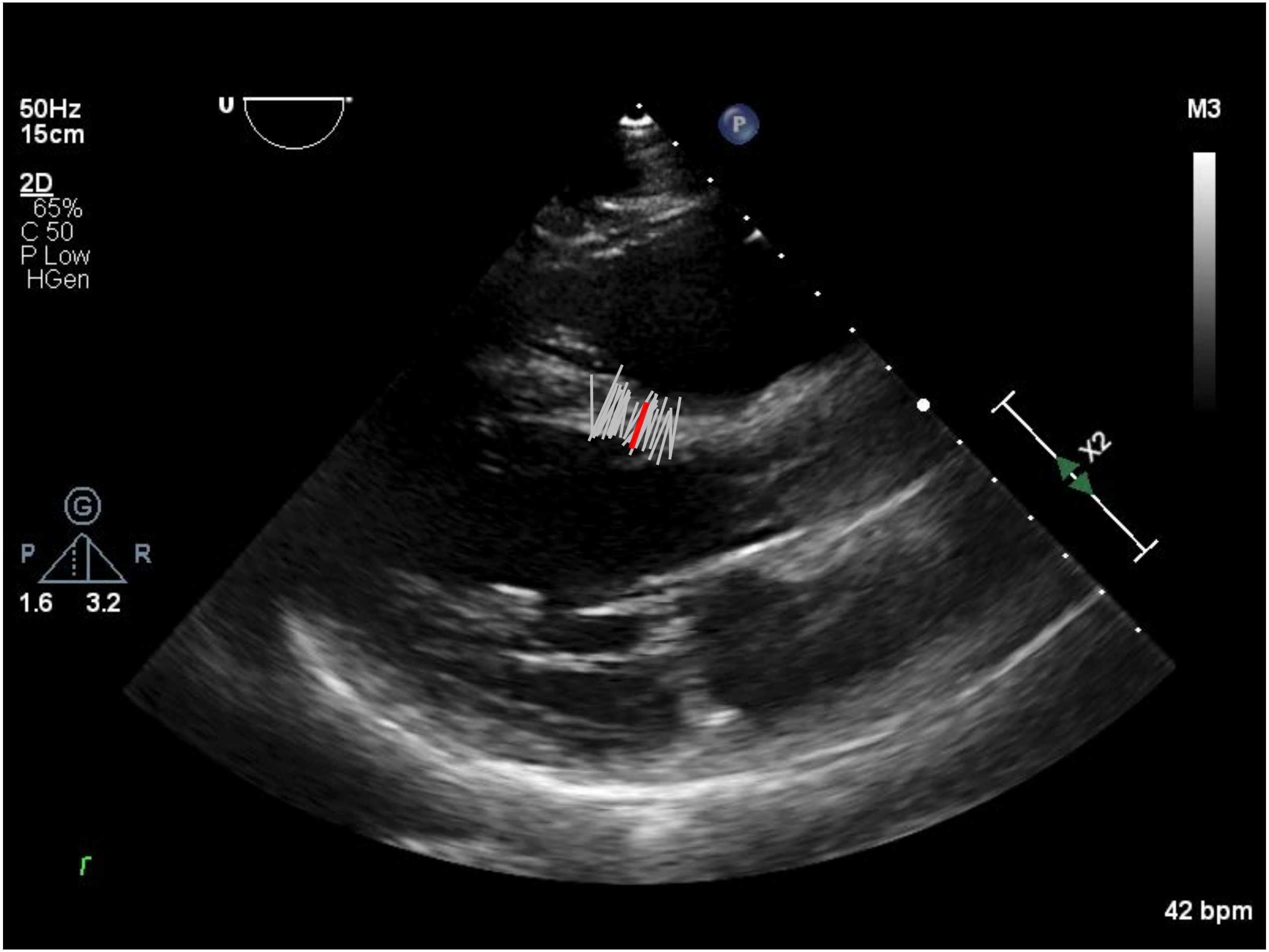

Pt 22 Diastole

Absolute difference between AI and expert consensus: 1.2 mm

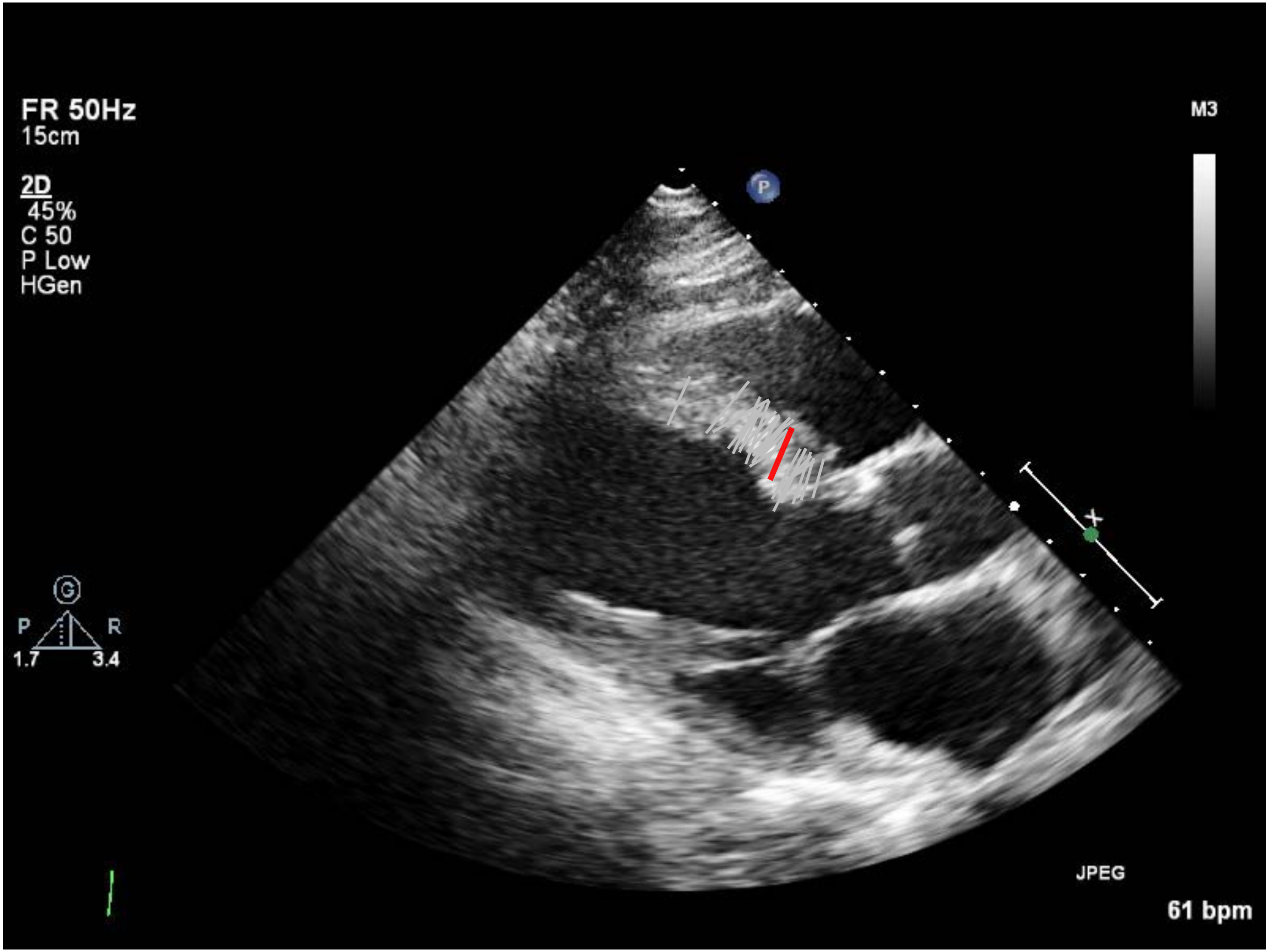

Pt 85 Diastole

Absolute difference between AI and expert consensus: 1.3 mm

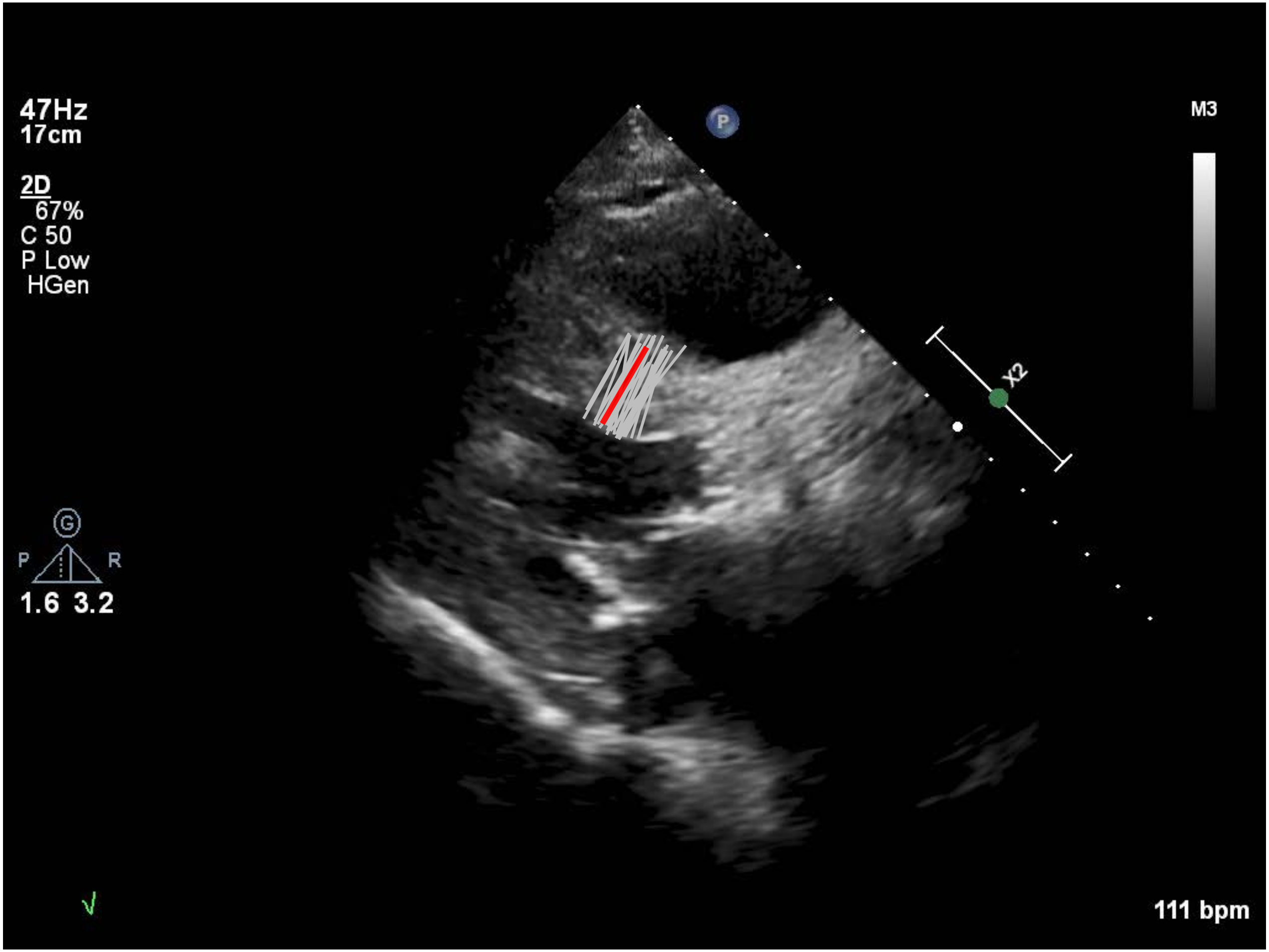

Pt 65 Diastole

Absolute difference between AI and expert consensus: 1.4 mm

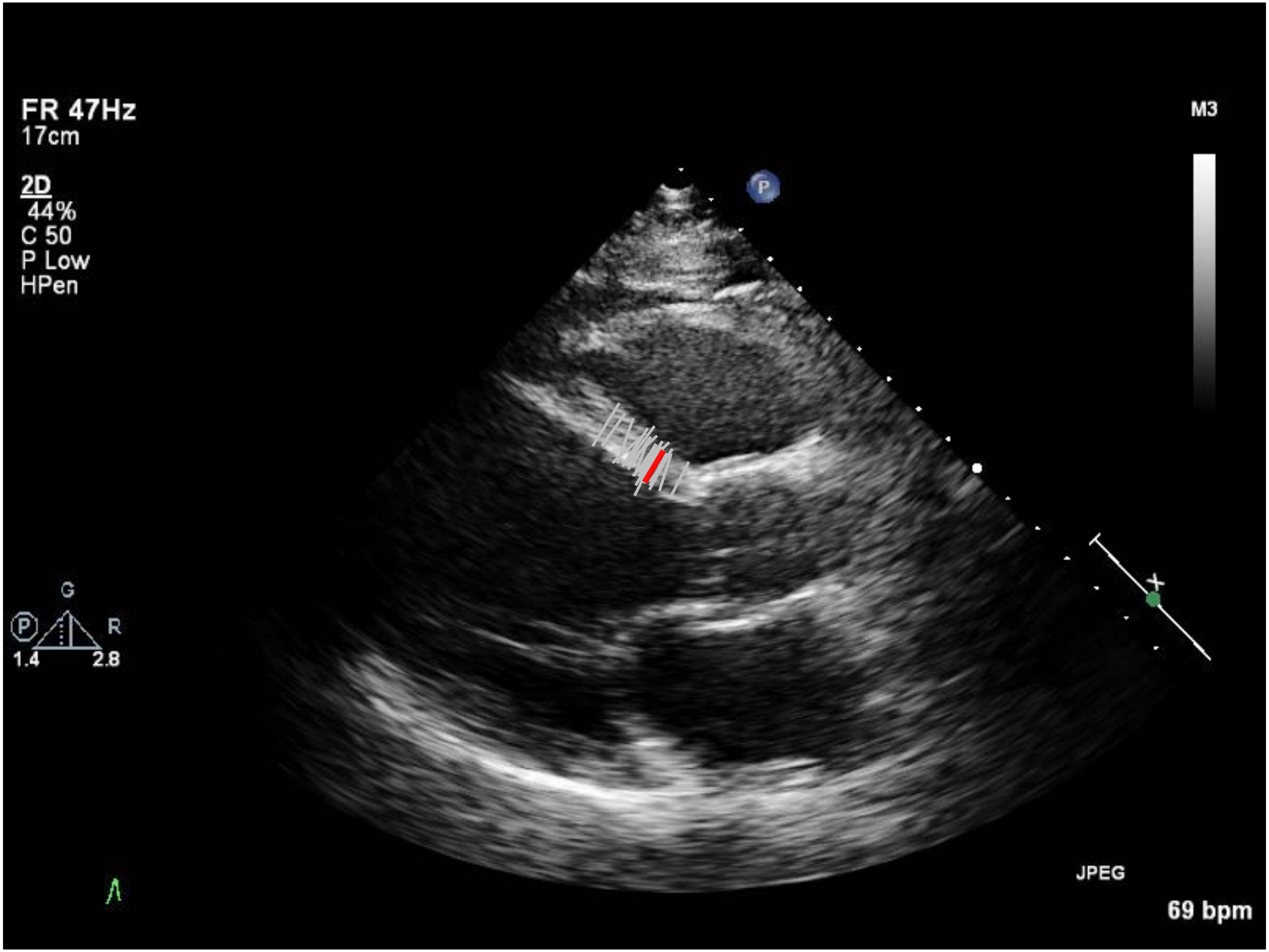

Pt 34 Diastole

Absolute difference between AI and expert consensus: 1.4 mm

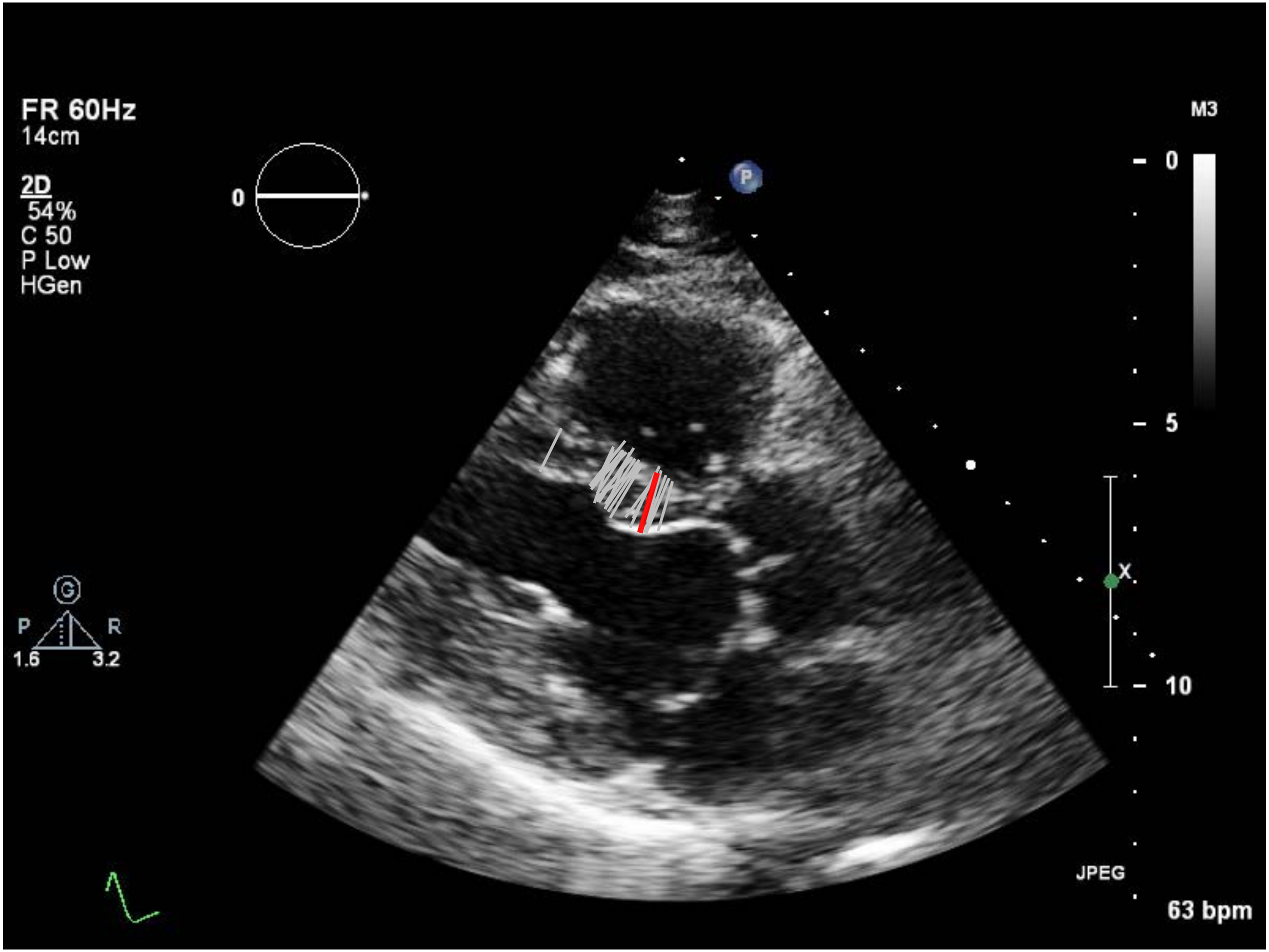

Pt 10 Diastole

Absolute difference between AI and expert consensus: 1.4 mm

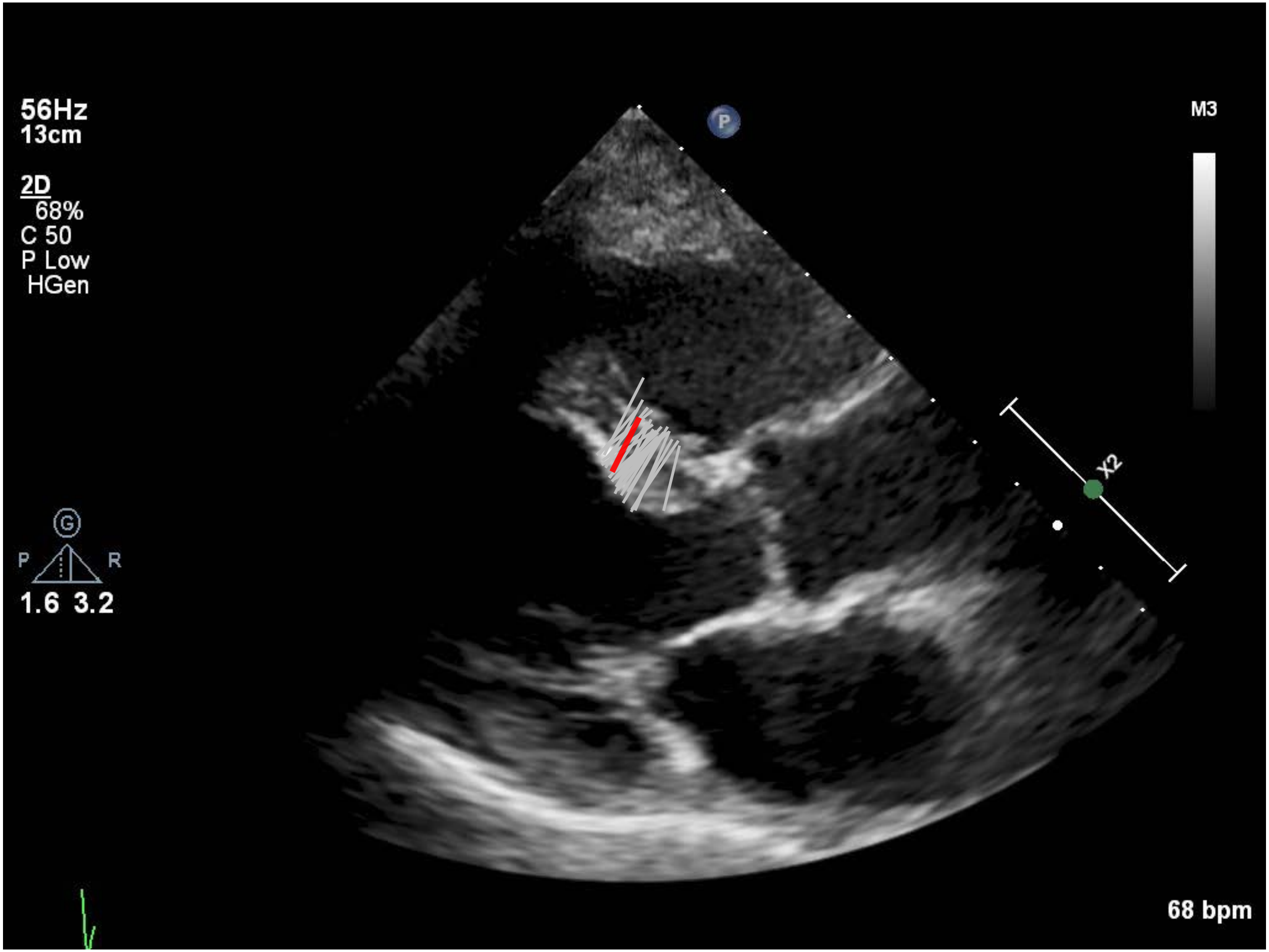

Pt 57 Diastole
Absolute difference between AI and expert consensus: 1.5 mm

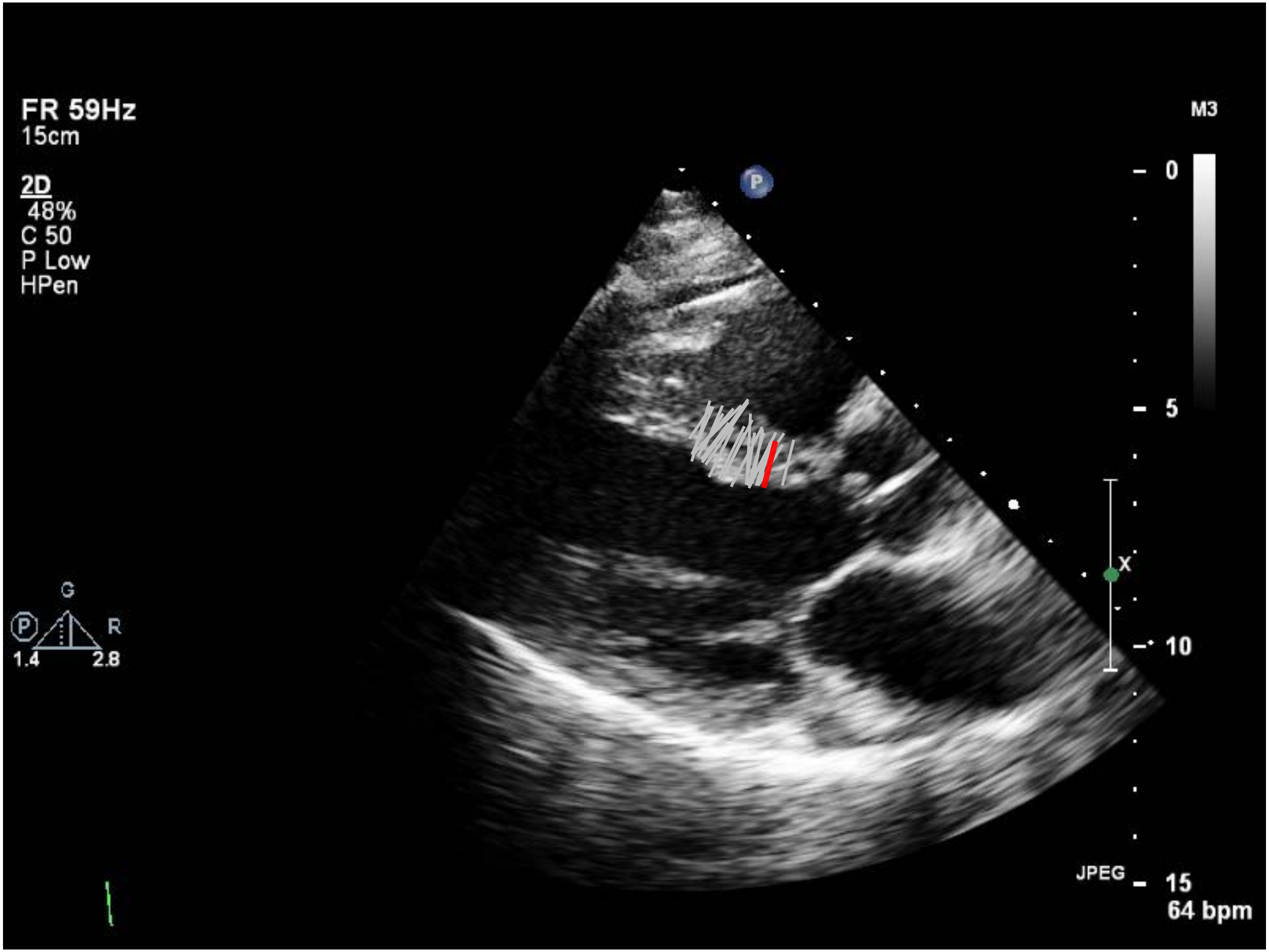

Pt 27 Diastole

Absolute difference between AI and expert consensus: 1.5 mm

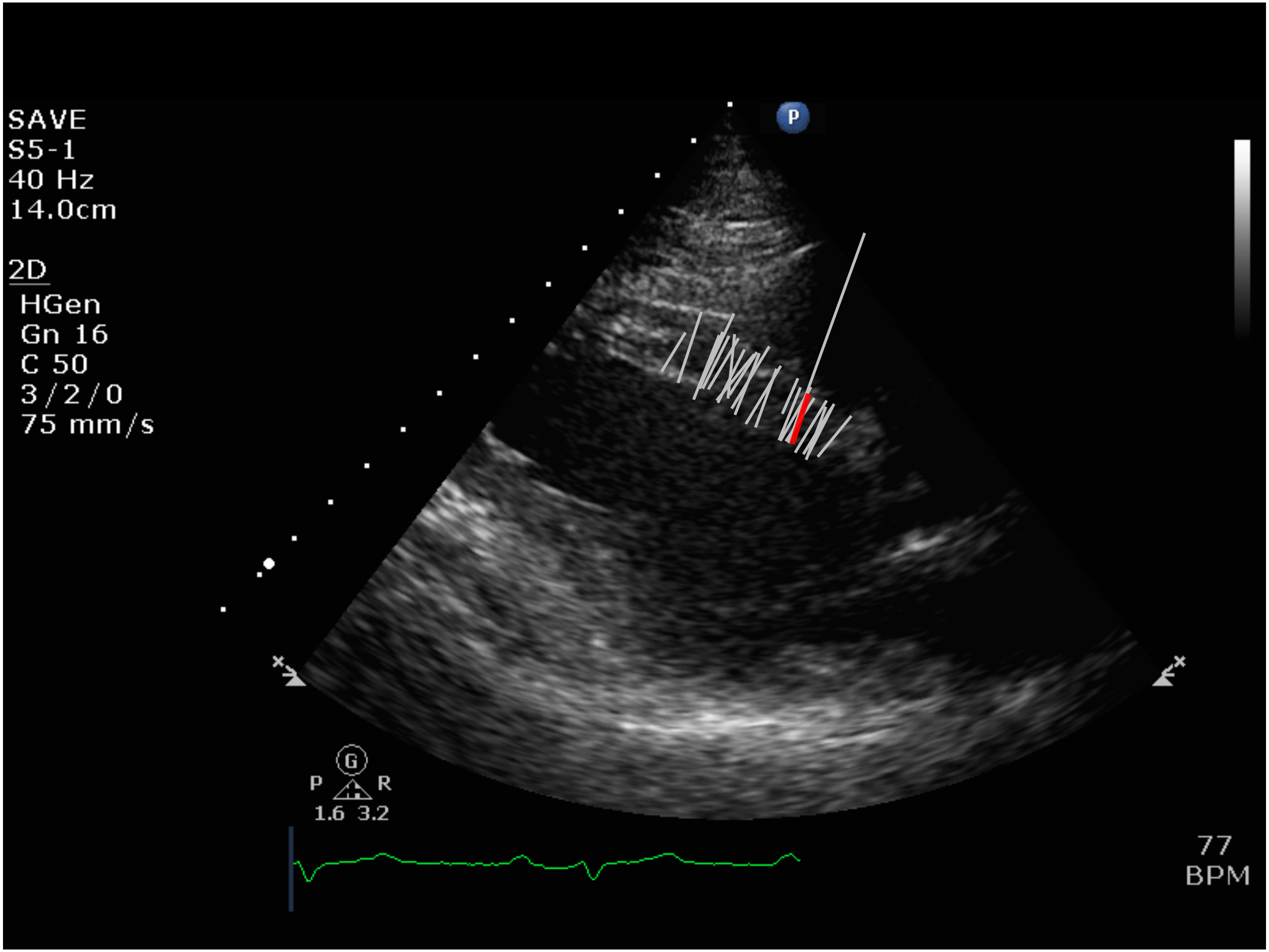

Pt 76 Diastole

Absolute difference between AI and expert consensus: 1.5 mm

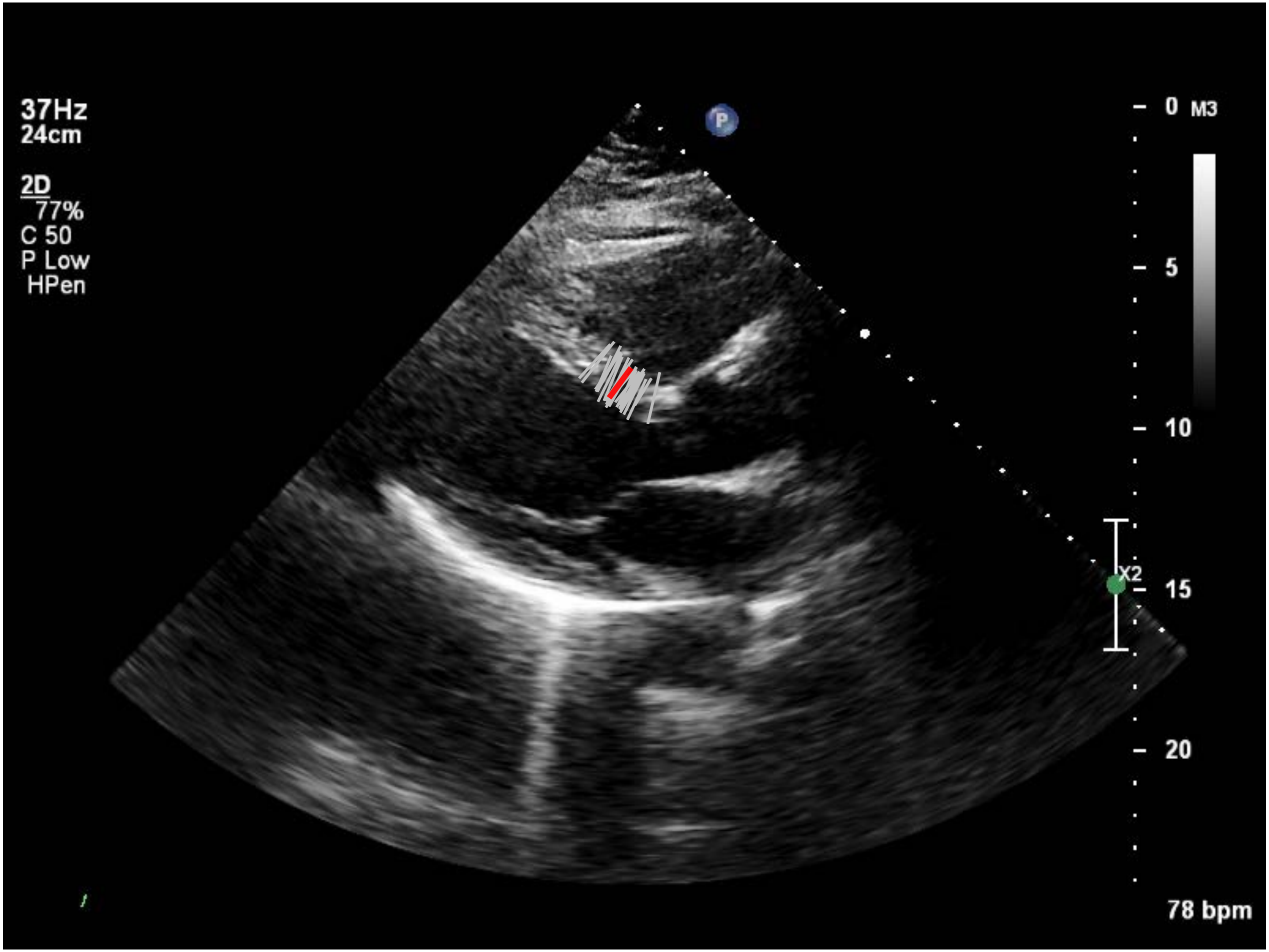

Pt 23 Diastole

Absolute difference between AI and expert consensus: 1.6 mm

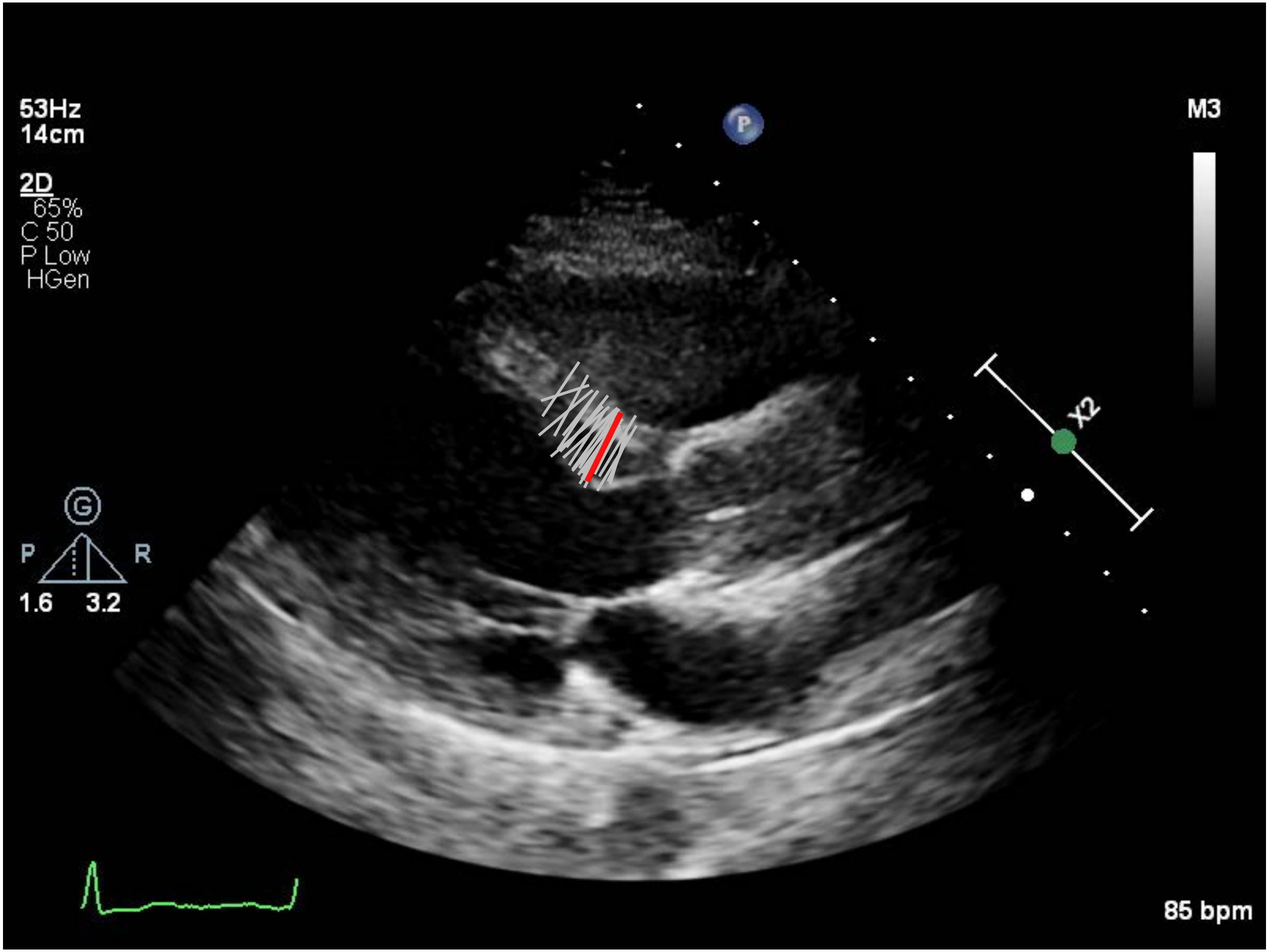

Pt 74 Diastole

Absolute difference between AI and expert consensus: 1.6 mm

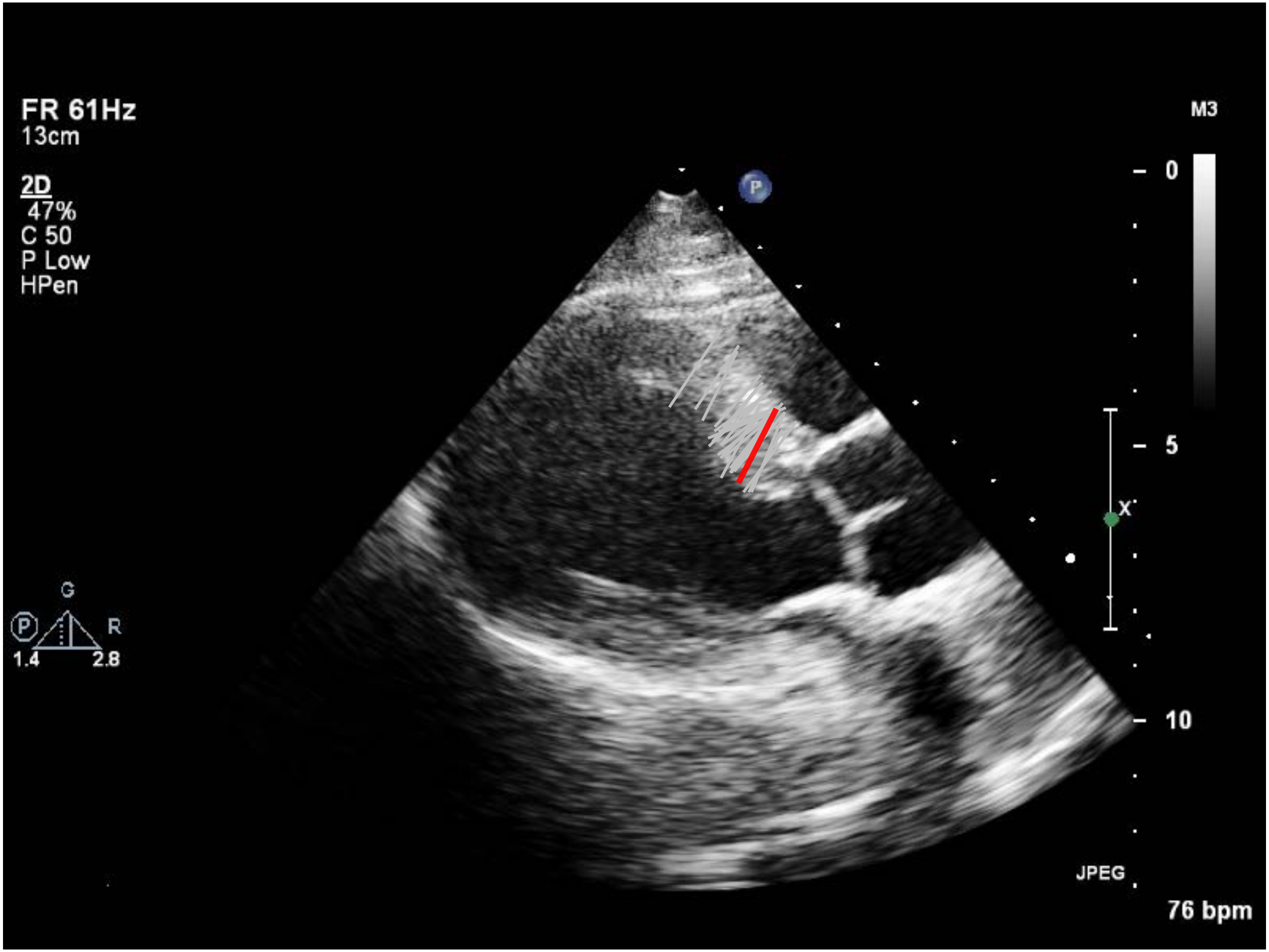

Pt 41 Diastole

Absolute difference between AI and expert consensus: 1.6 mm

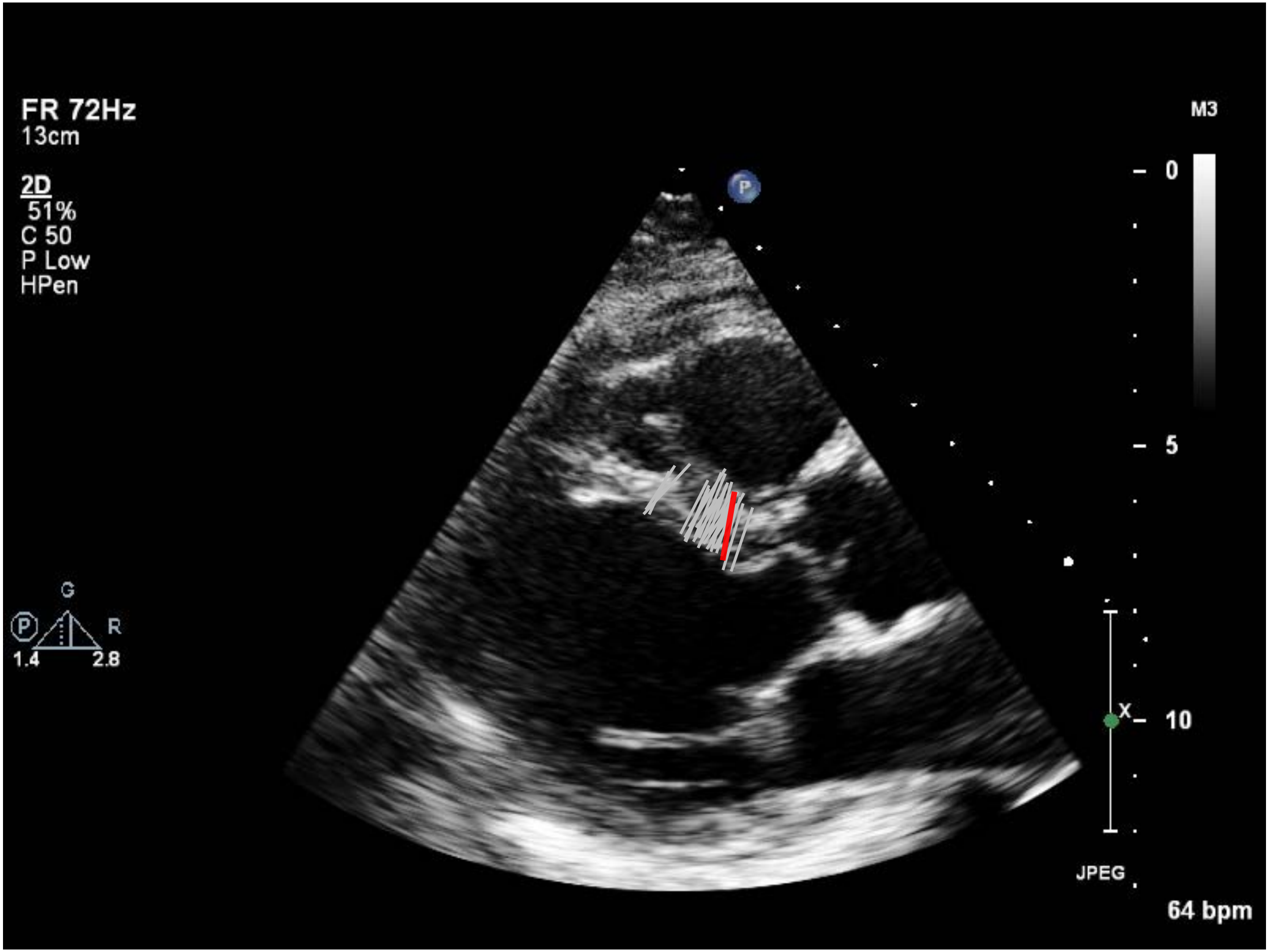

Pt 48 Diastole

Absolute difference between AI and expert consensus: 1.7 mm

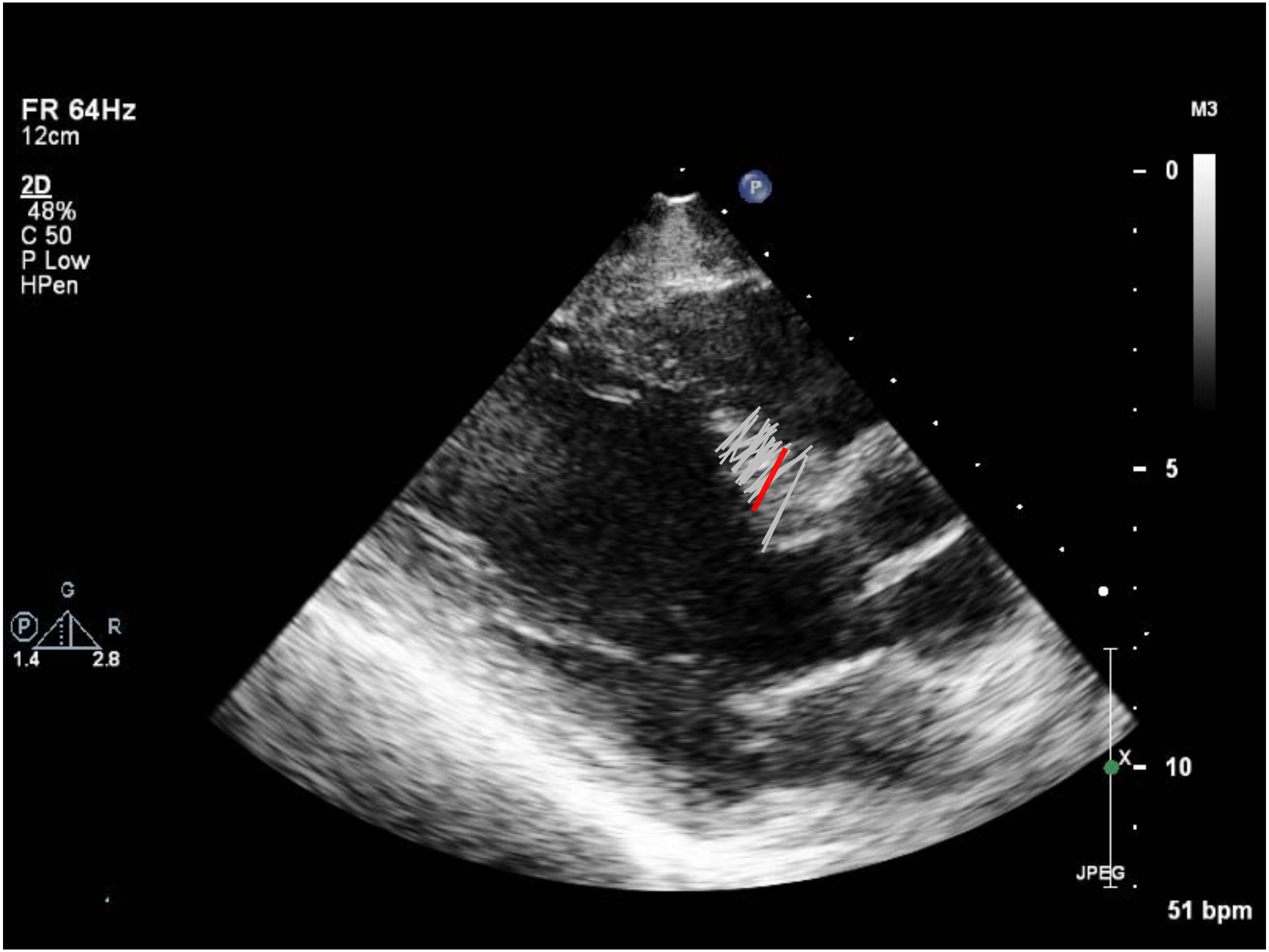

Pt 36 Diastole

Absolute difference between AI and expert consensus: 1.8 mm

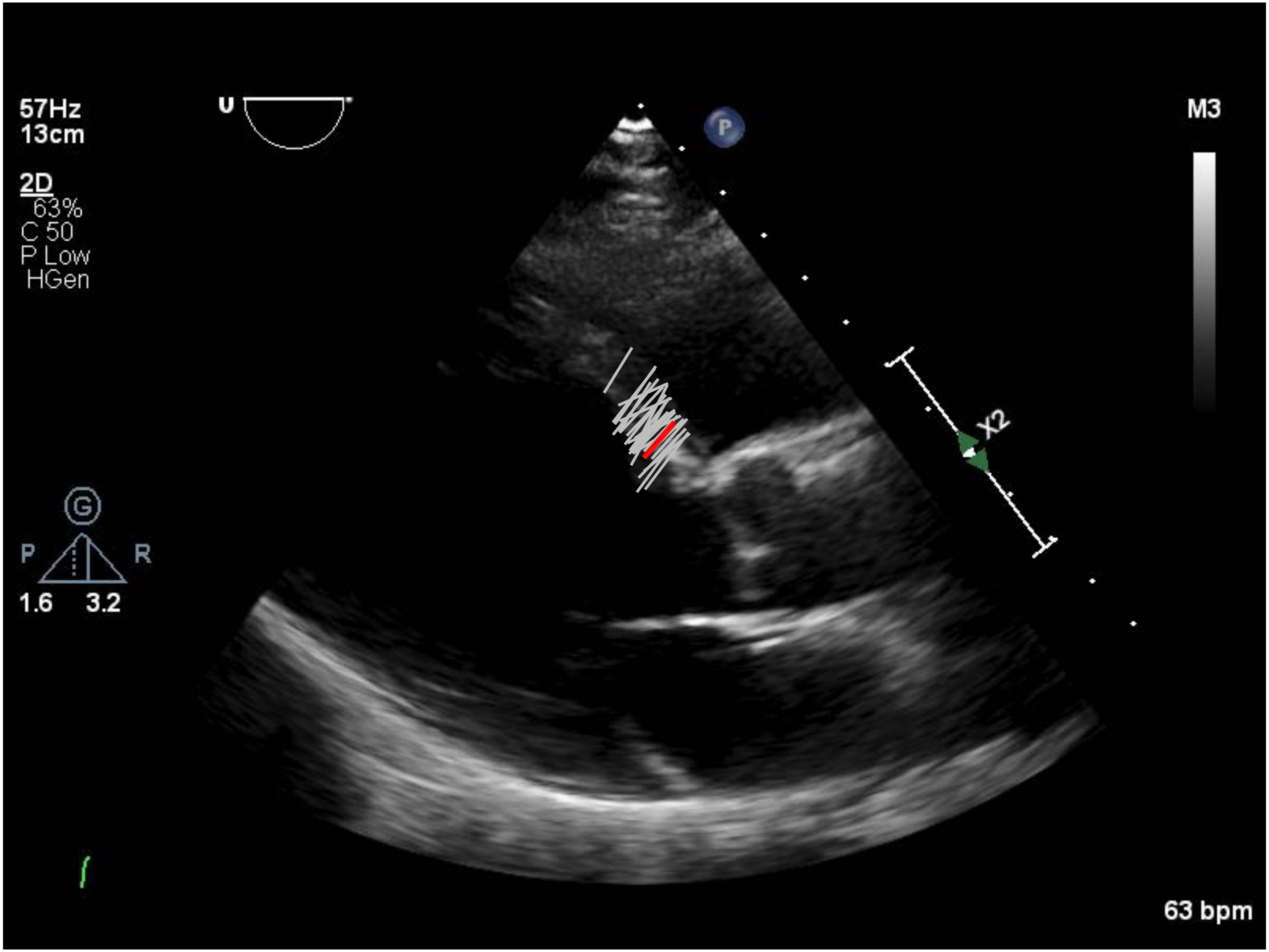

Pt 53 Diastole

Absolute difference between AI and expert consensus: 1.8 mm

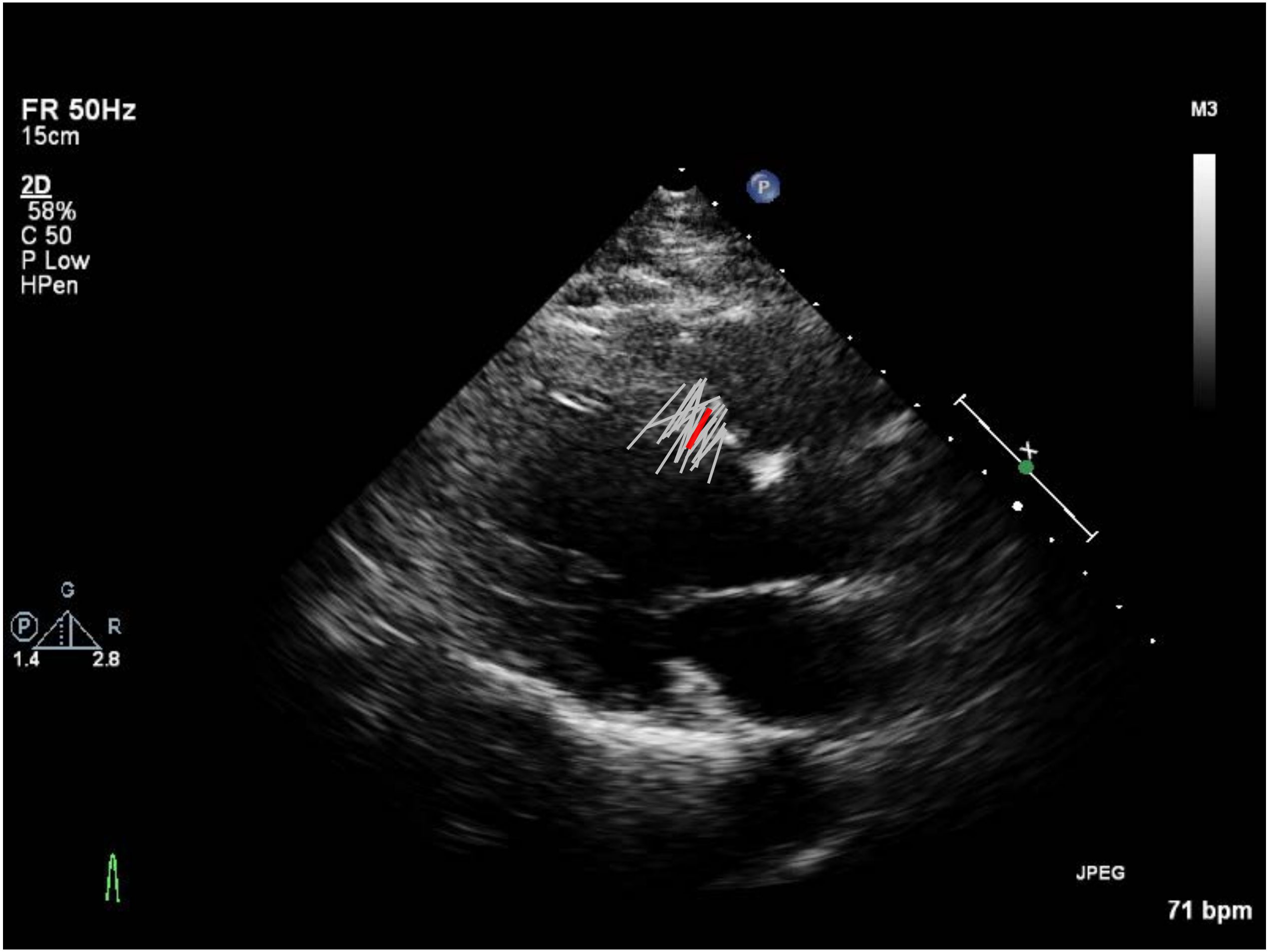

Pt 77 Diastole

Absolute difference between AI and expert consensus: 1.8 mm

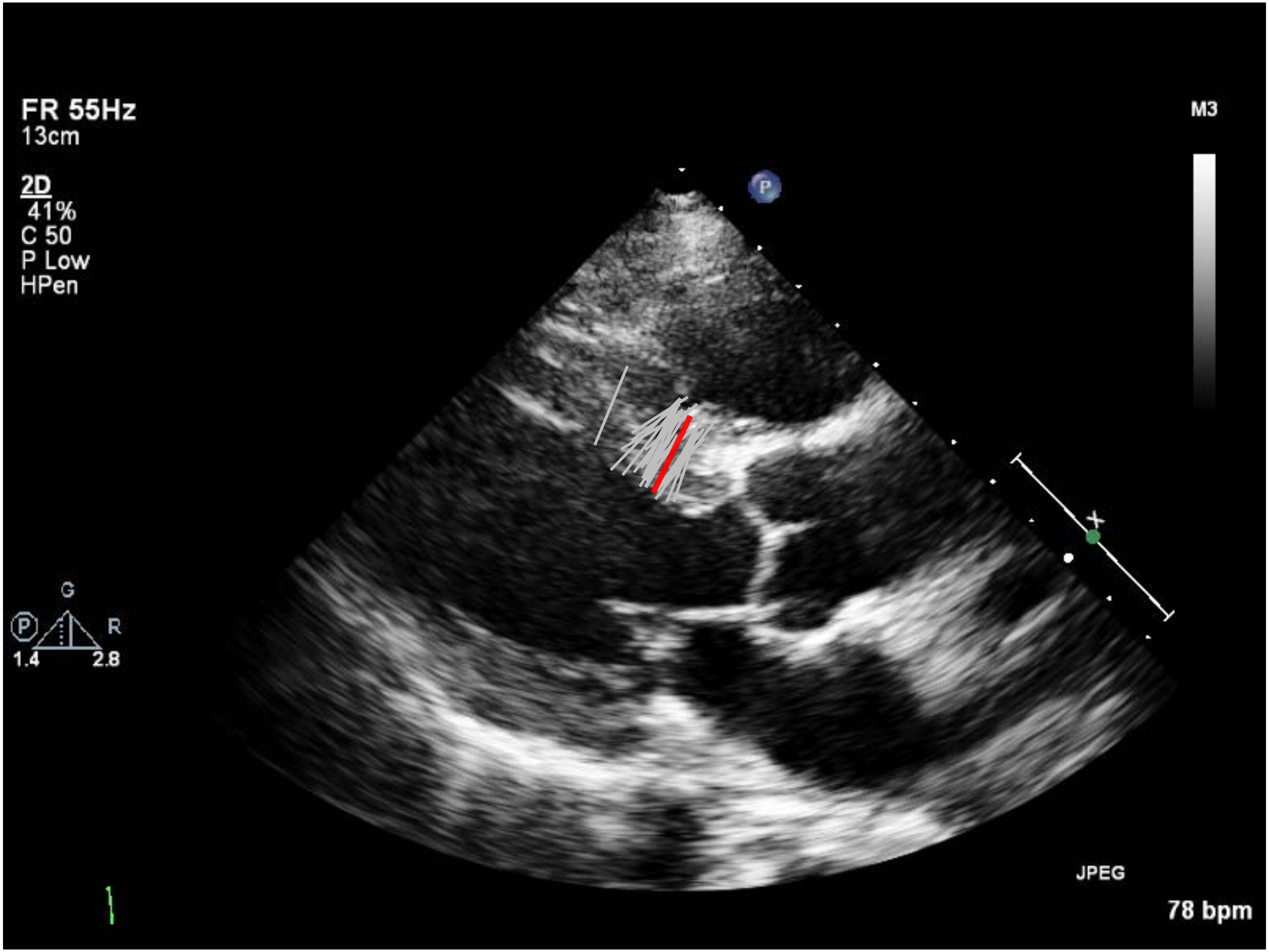

Pt 31 Diastole

Absolute difference between AI and expert consensus: 1.8 mm

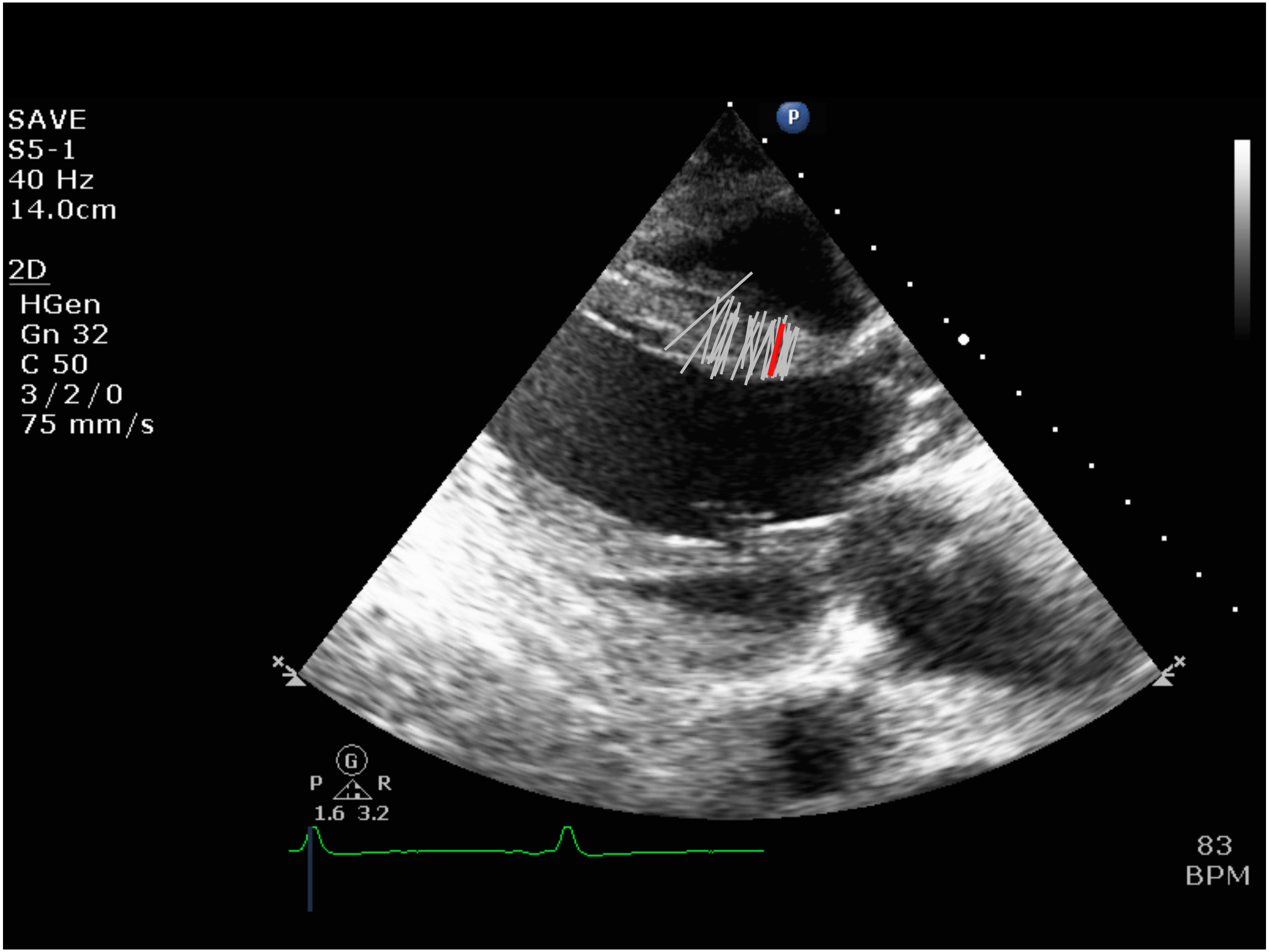

Pt 84 Diastole

Absolute difference between AI and expert consensus: 1.9 mm

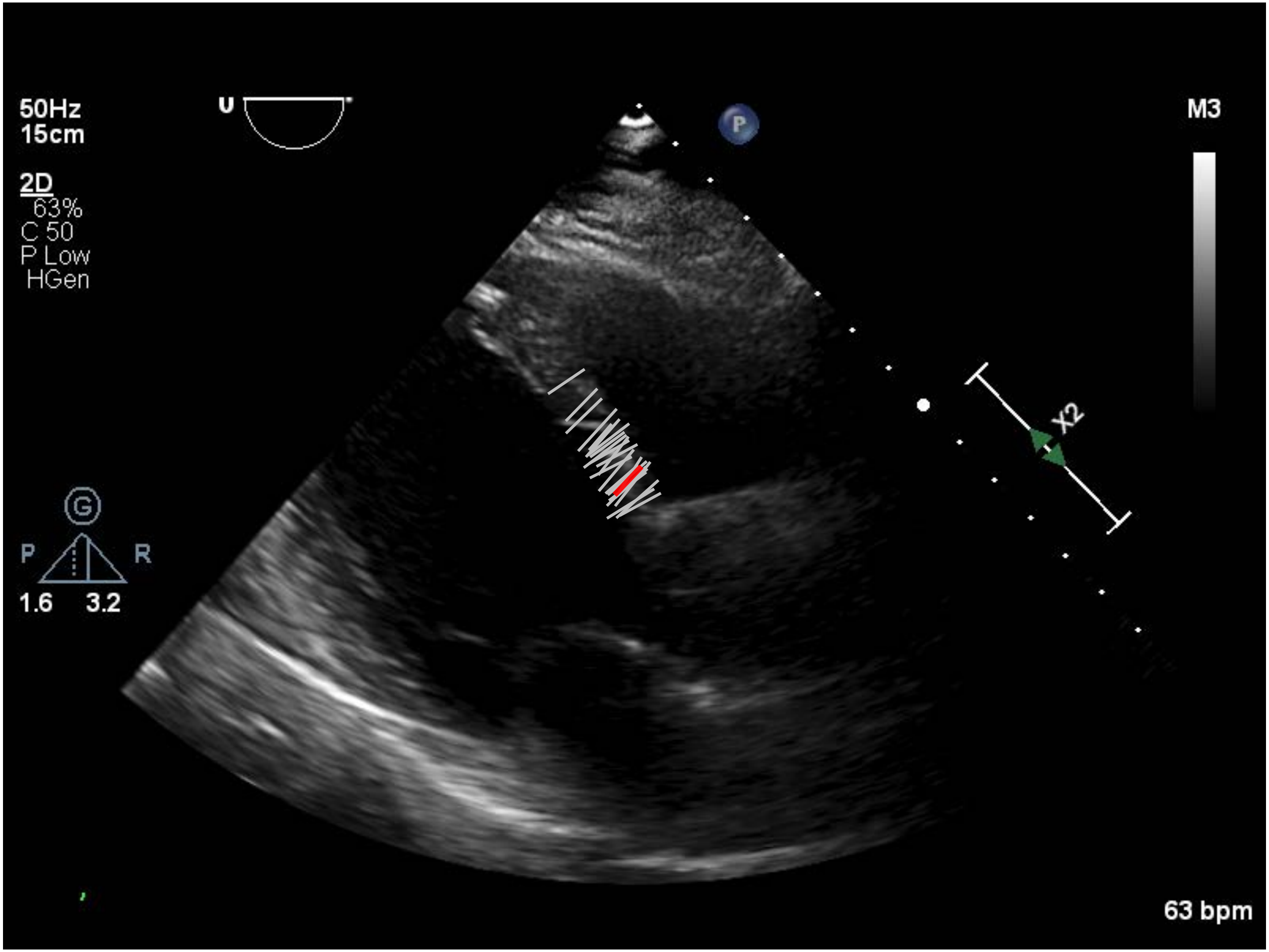

Pt 95 Diastole

Absolute difference between AI and expert consensus: 2 mm

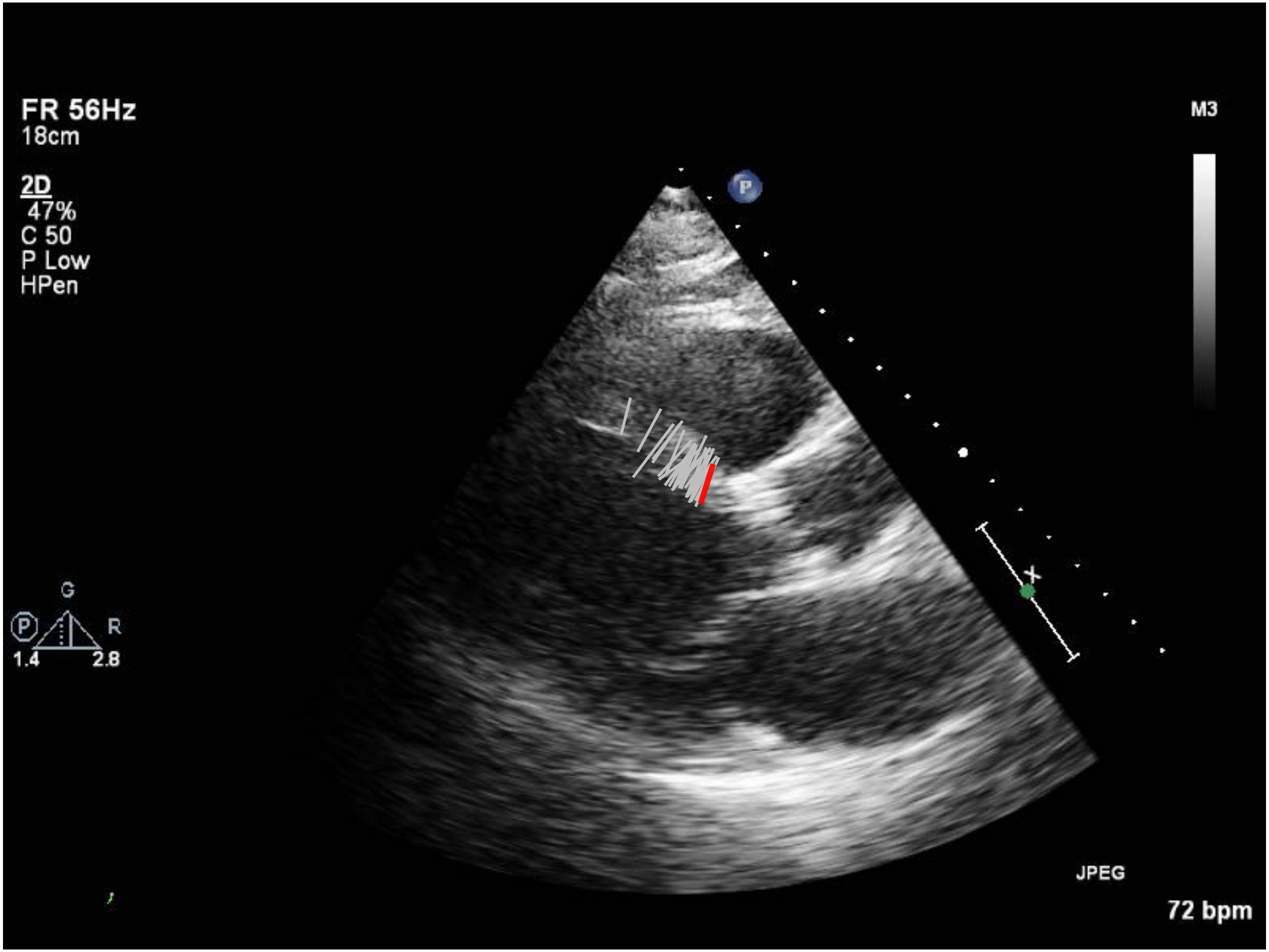

Pt 19 Diastole

Absolute difference between AI and expert consensus: 2.1 mm

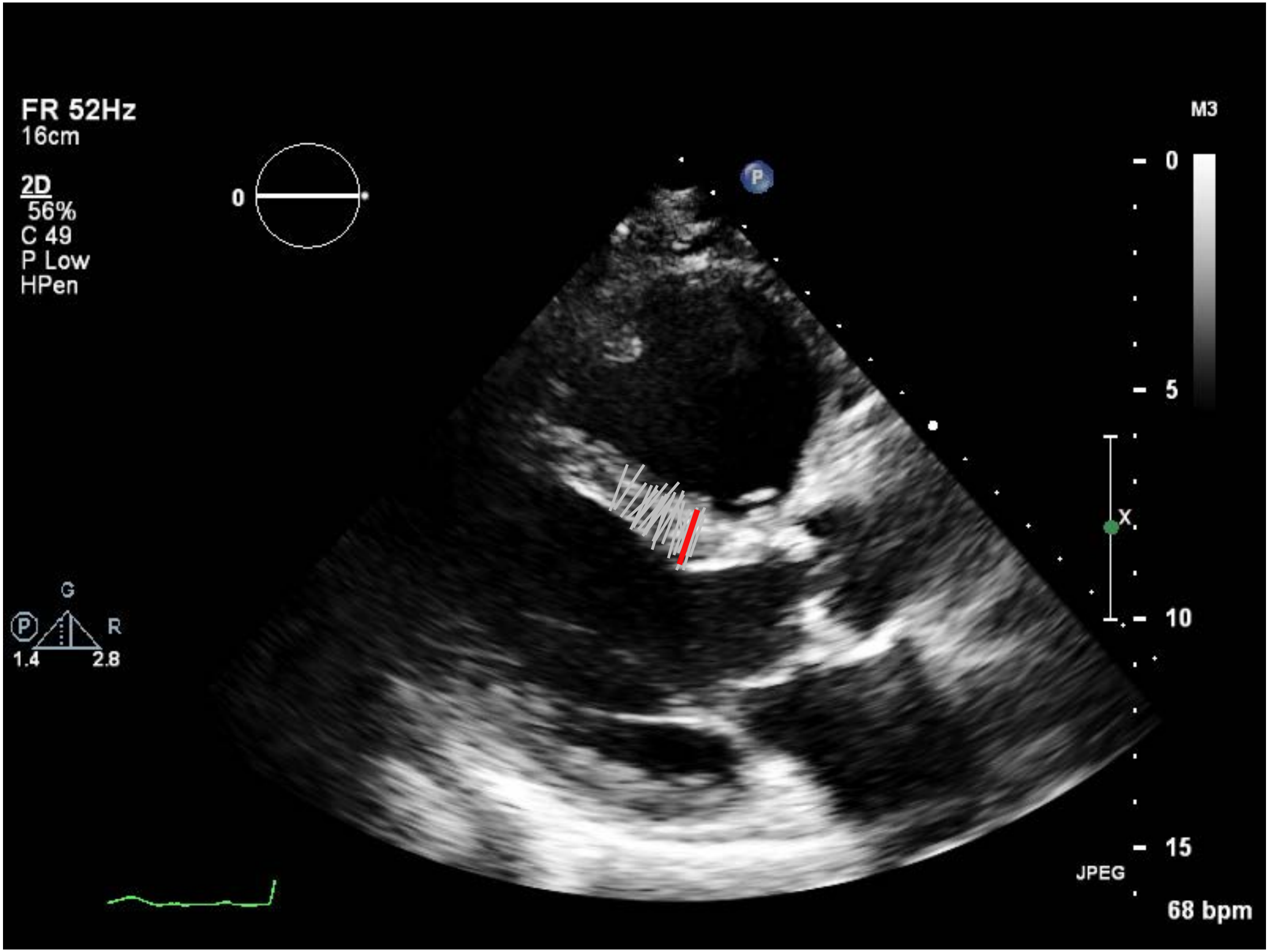

Pt 37 Diastole

Absolute difference between AI and expert consensus: 2.1 mm

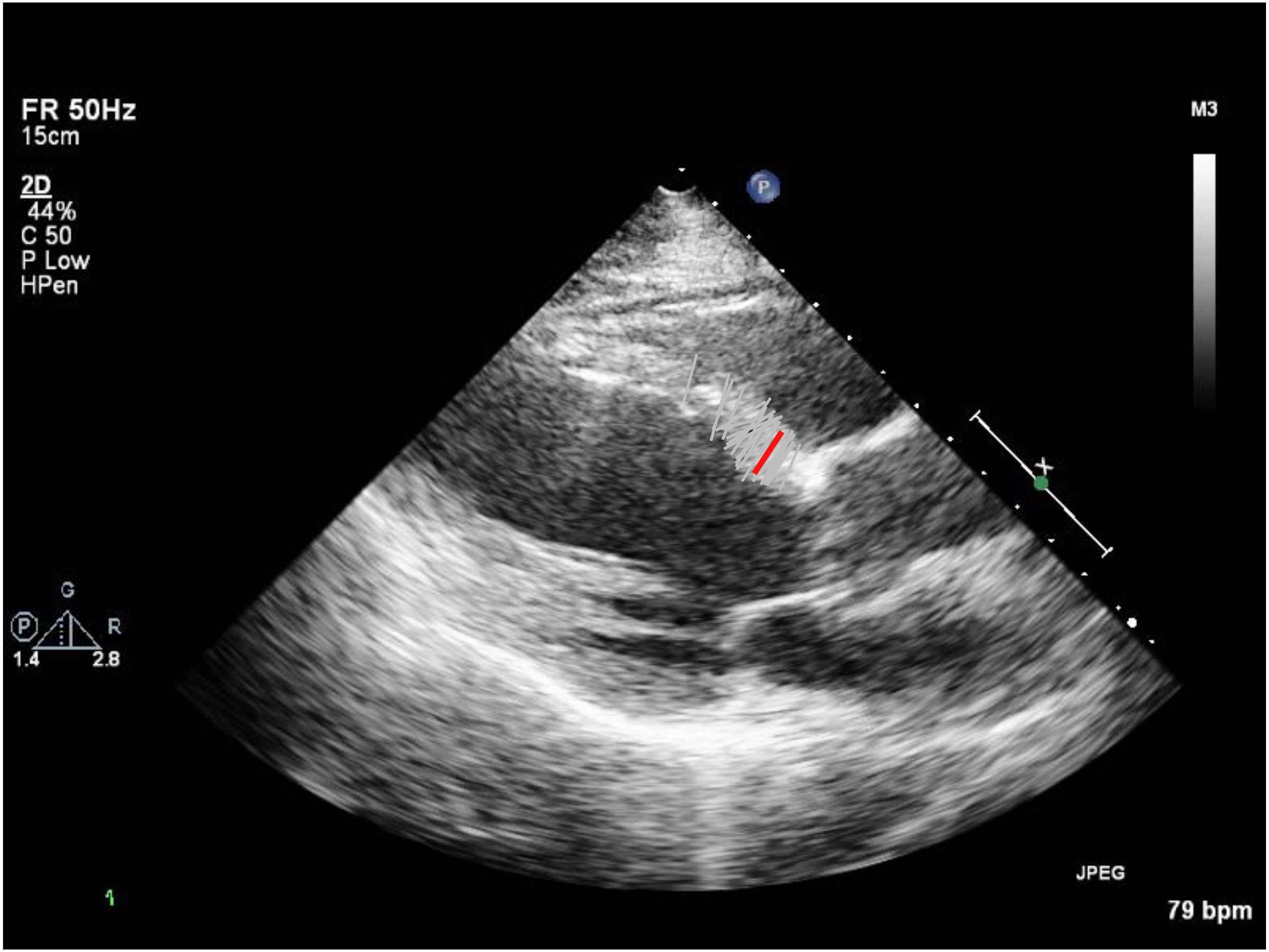

Pt 12 Diastole

Absolute difference between AI and expert consensus: 2.1 mm

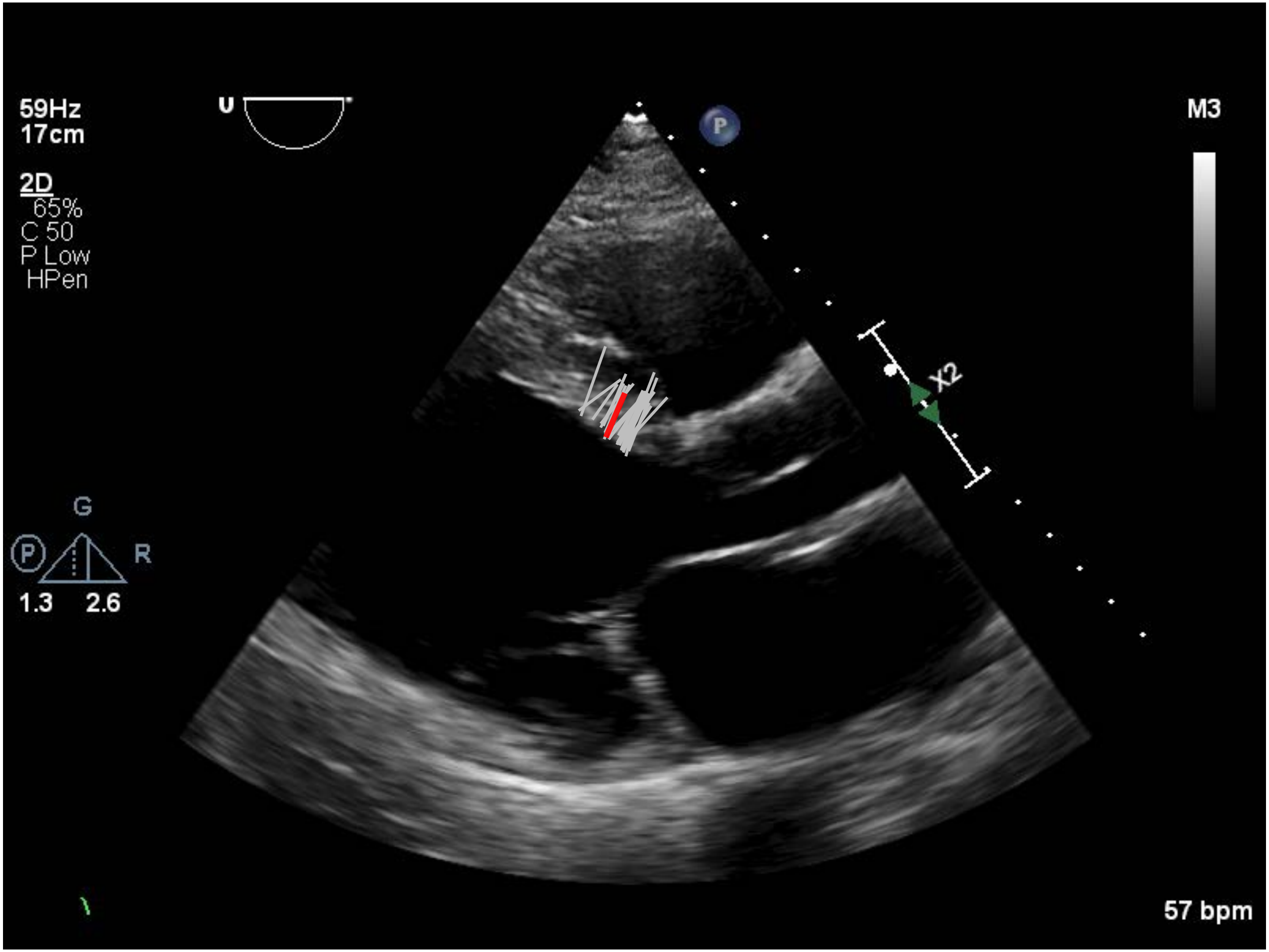

Pt 79 Diastole

Absolute difference between AI and expert consensus: 2.2 mm

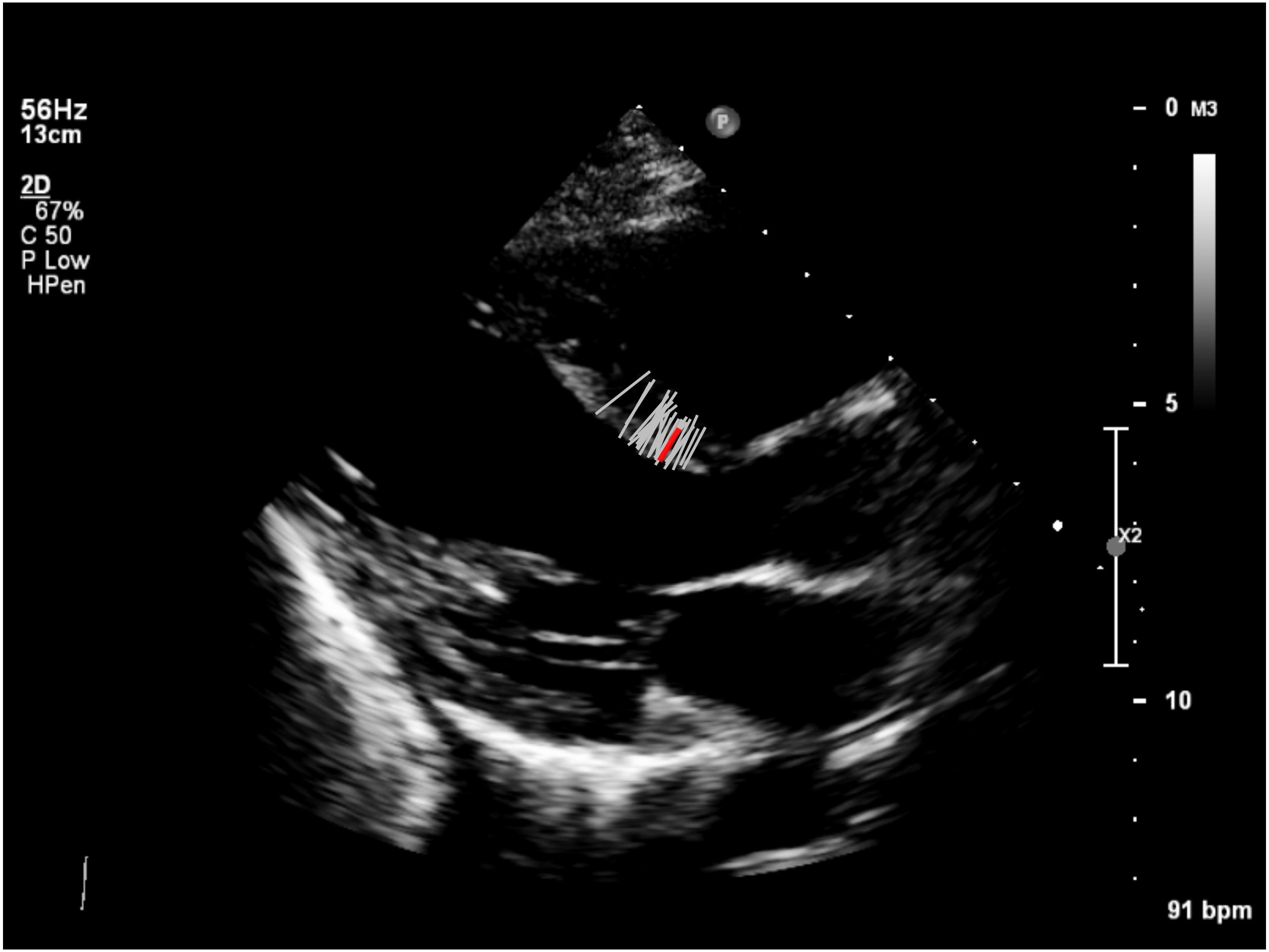

Pt 54 Diastole

Absolute difference between AI and expert consensus: 2.5 mm

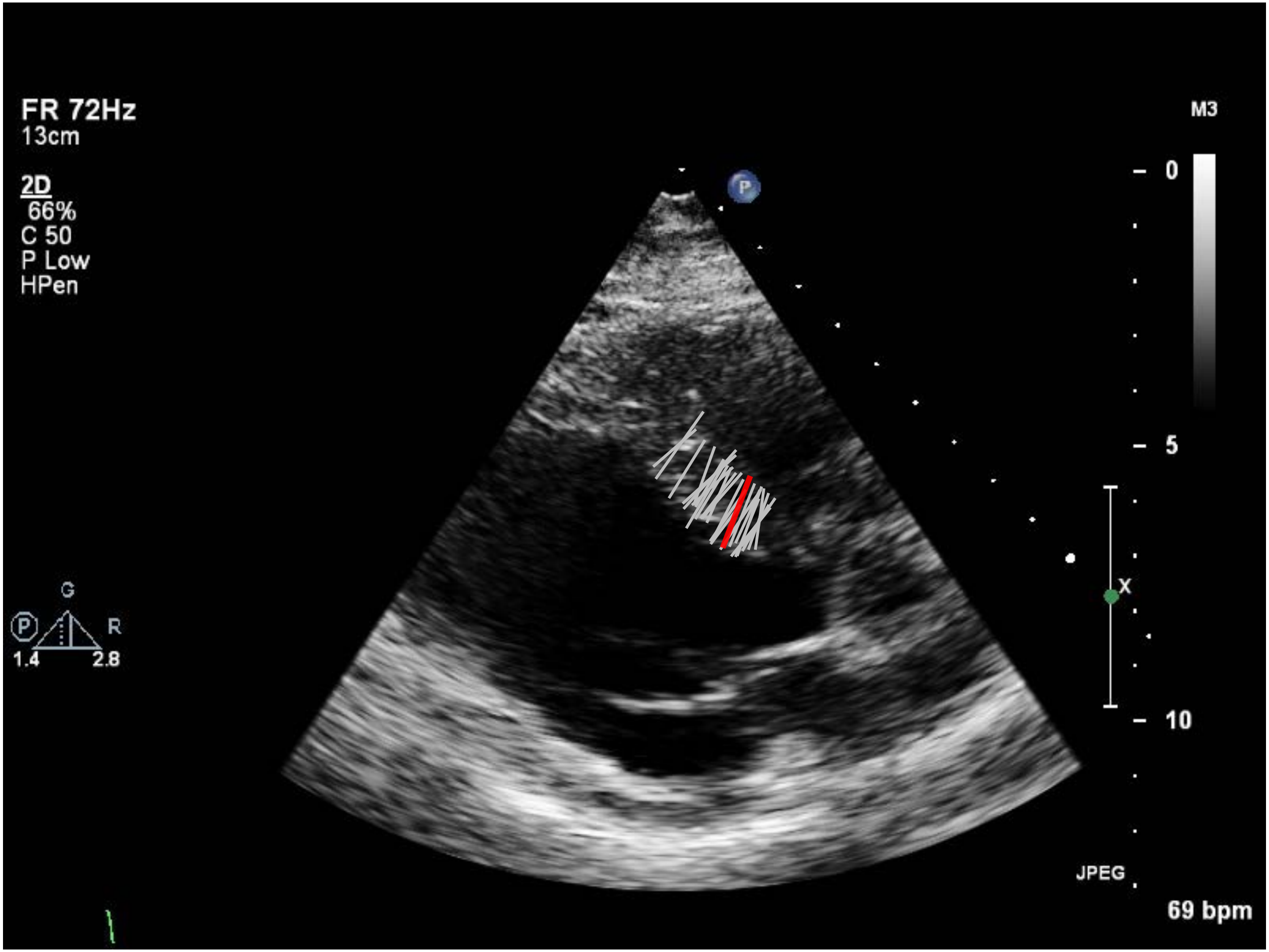

Pt 67 Diastole

Absolute difference between AI and expert consensus: 2.6 mm

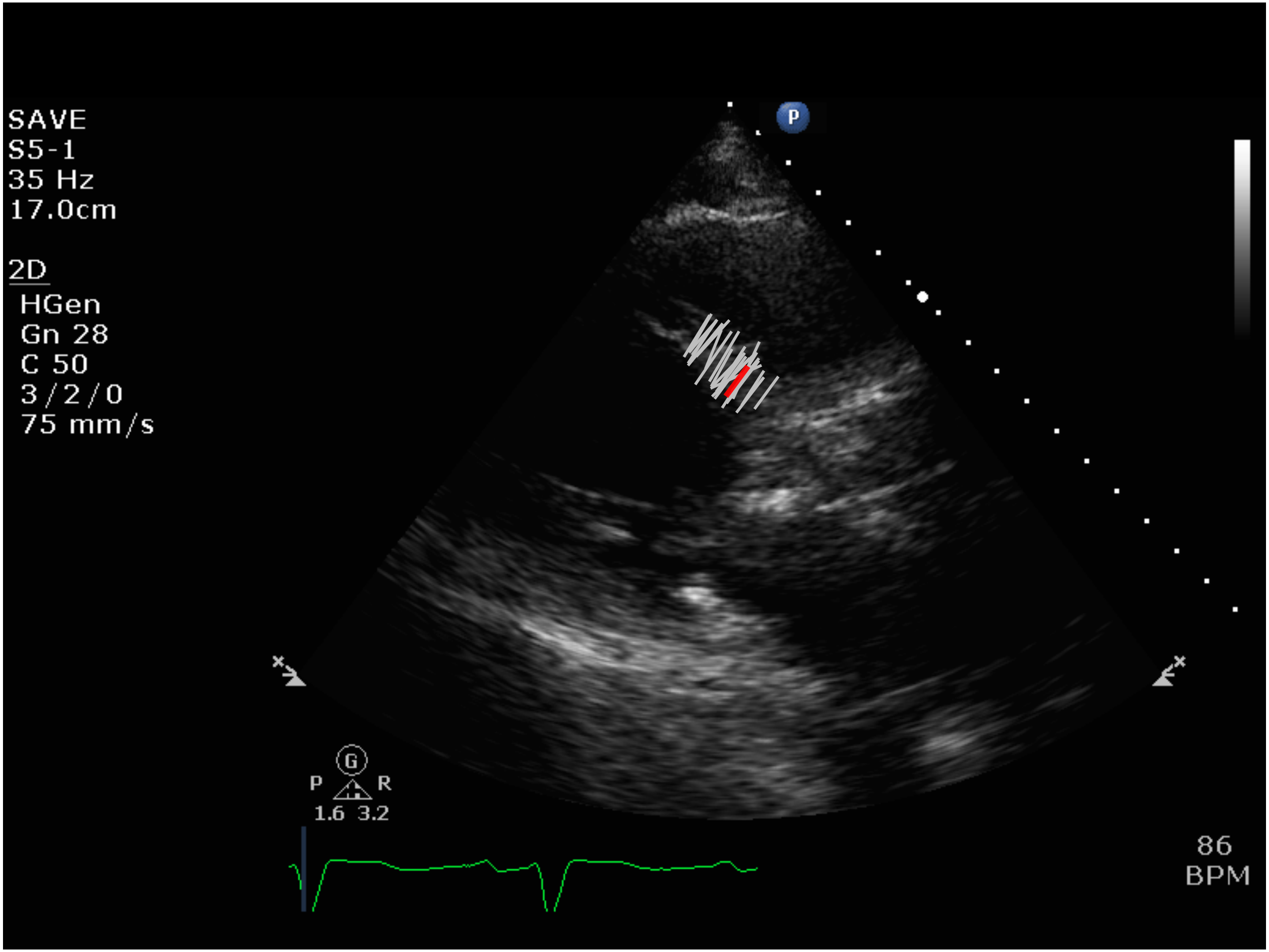

Pt 18 Diastole

Absolute difference between AI and expert consensus: 2.7 mm

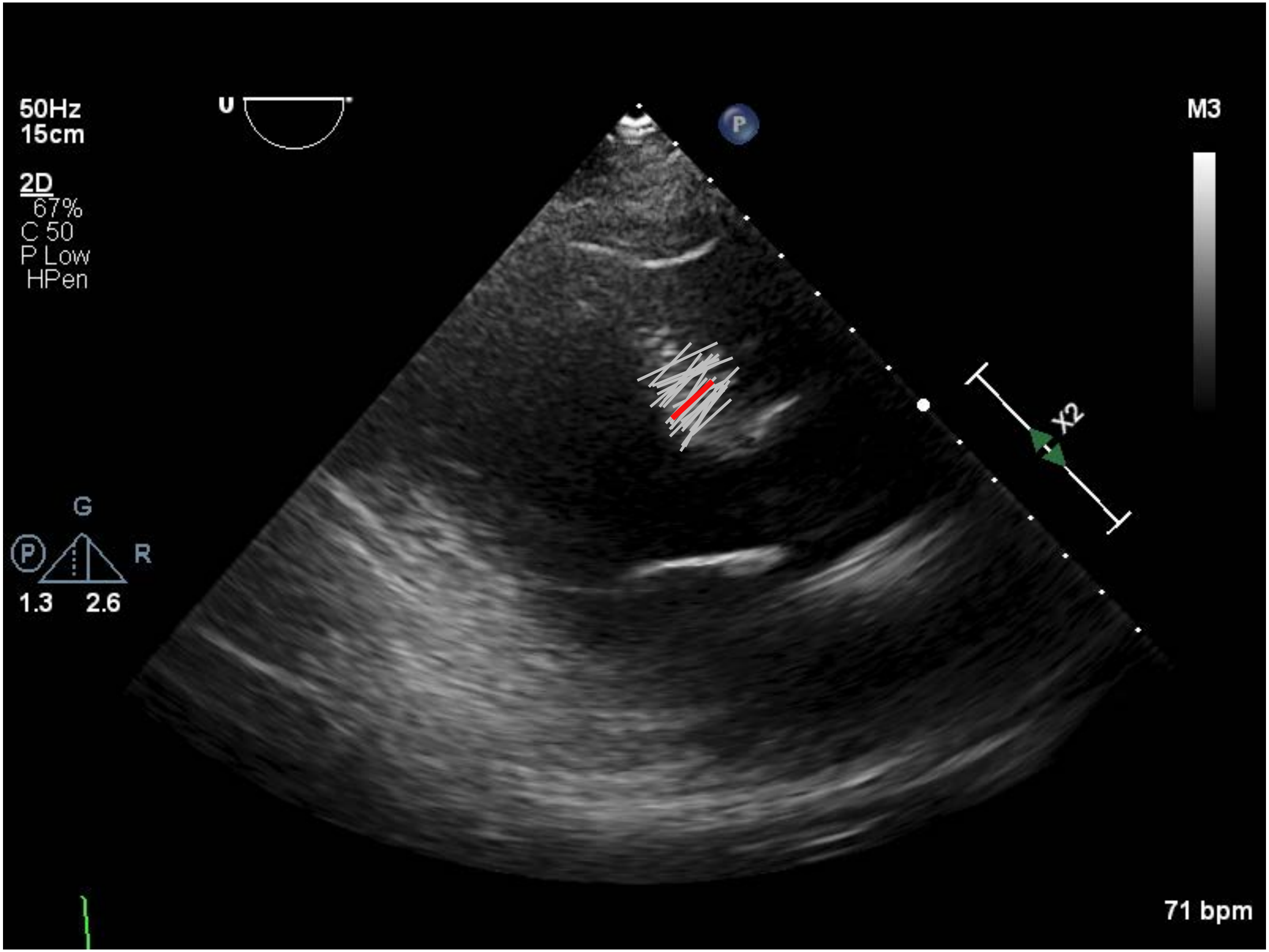

Pt 97 Diastole

Absolute difference between AI and expert consensus: 2.8 mm

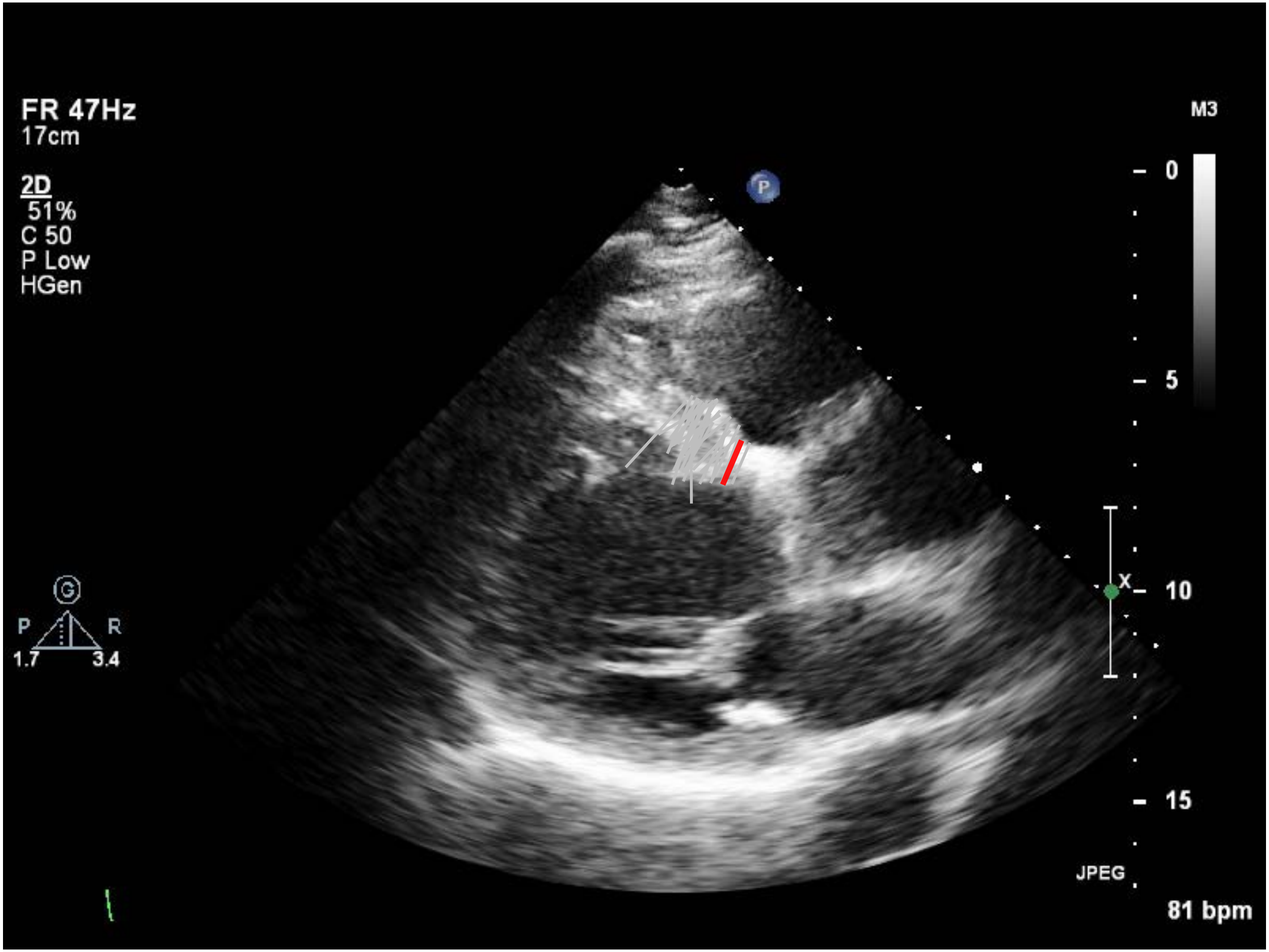

Pt 32 Diastole

Absolute difference between AI and expert consensus: 2.9 mm

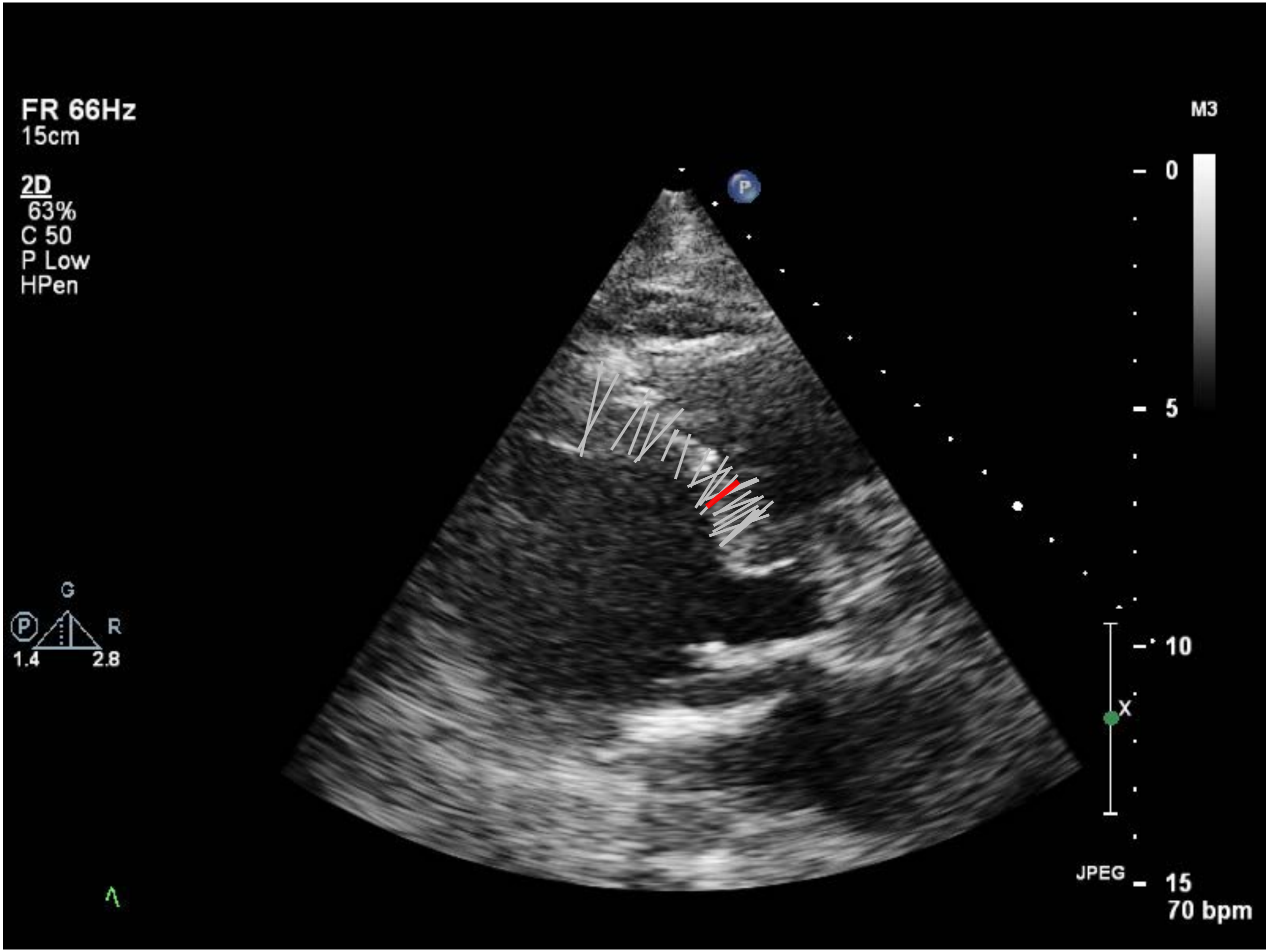

Pt 62 Diastole

Absolute difference between AI and expert consensus: 3 mm

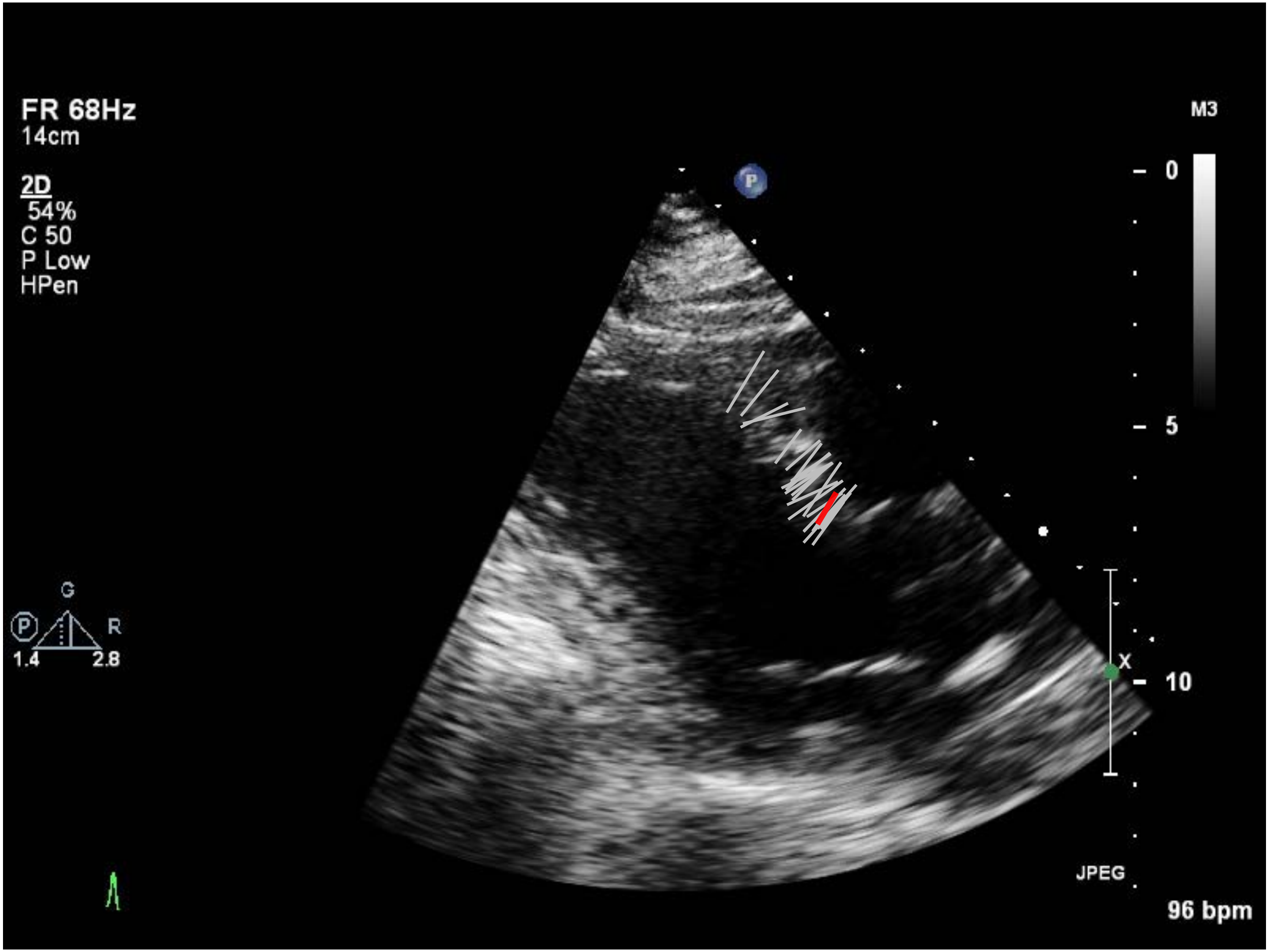

Pt 49 Diastole

Absolute difference between AI and expert consensus: 3.2 mm

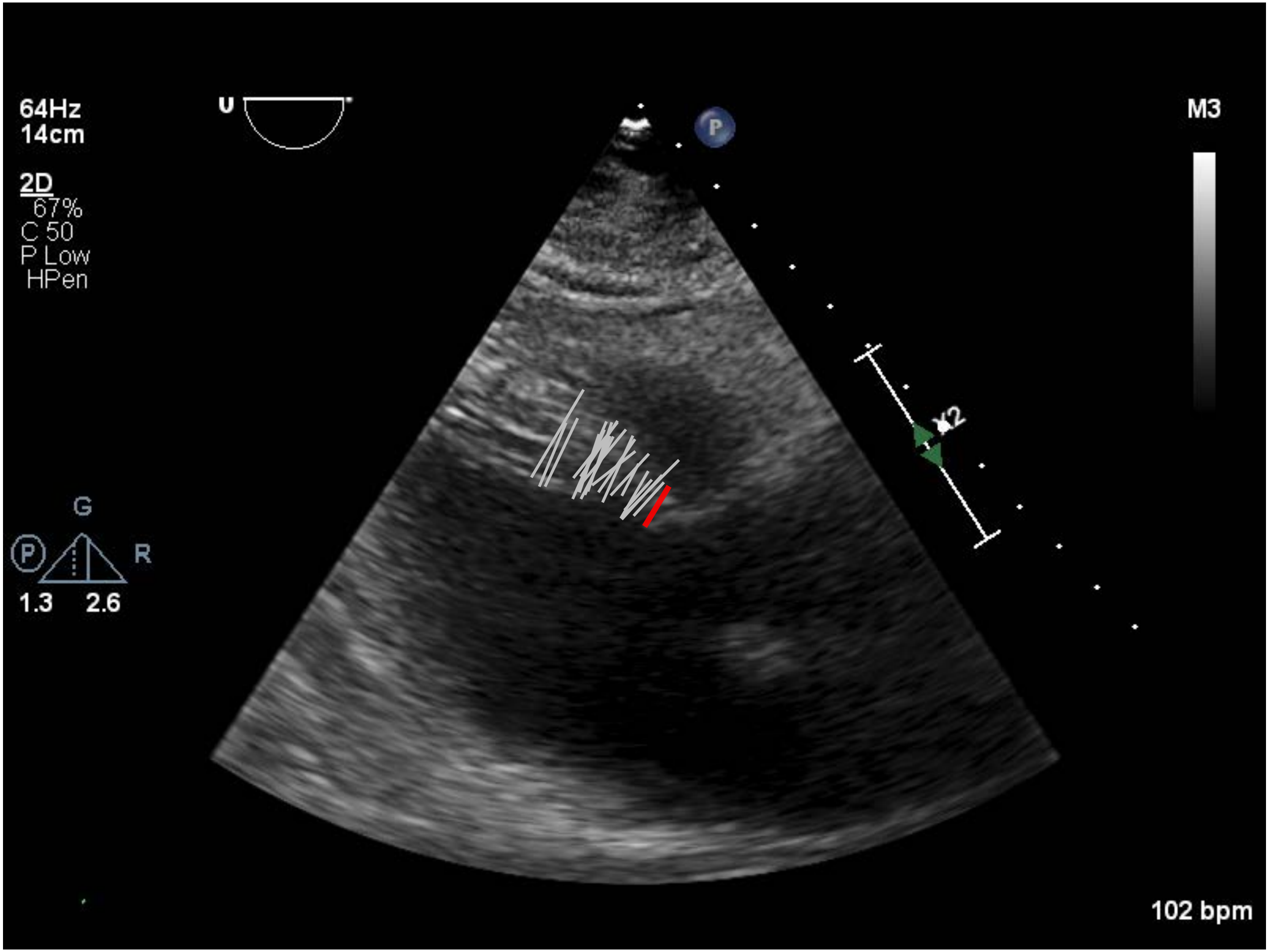

Pt 99 Diastole

Absolute difference between AI and expert consensus: 3.5 mm

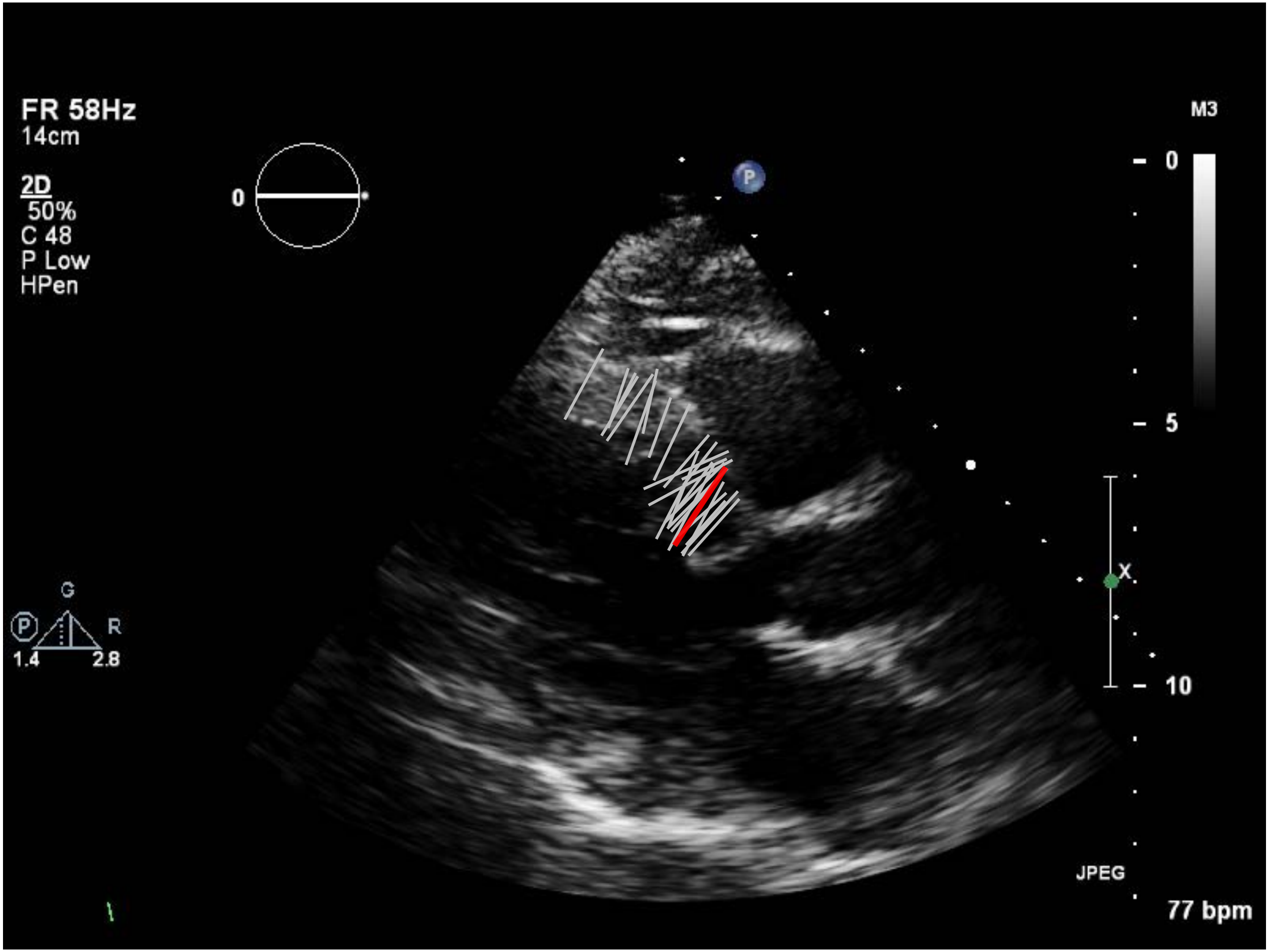

Pt 3 Diastole

Absolute difference between AI and expert consensus: 3.6 mm

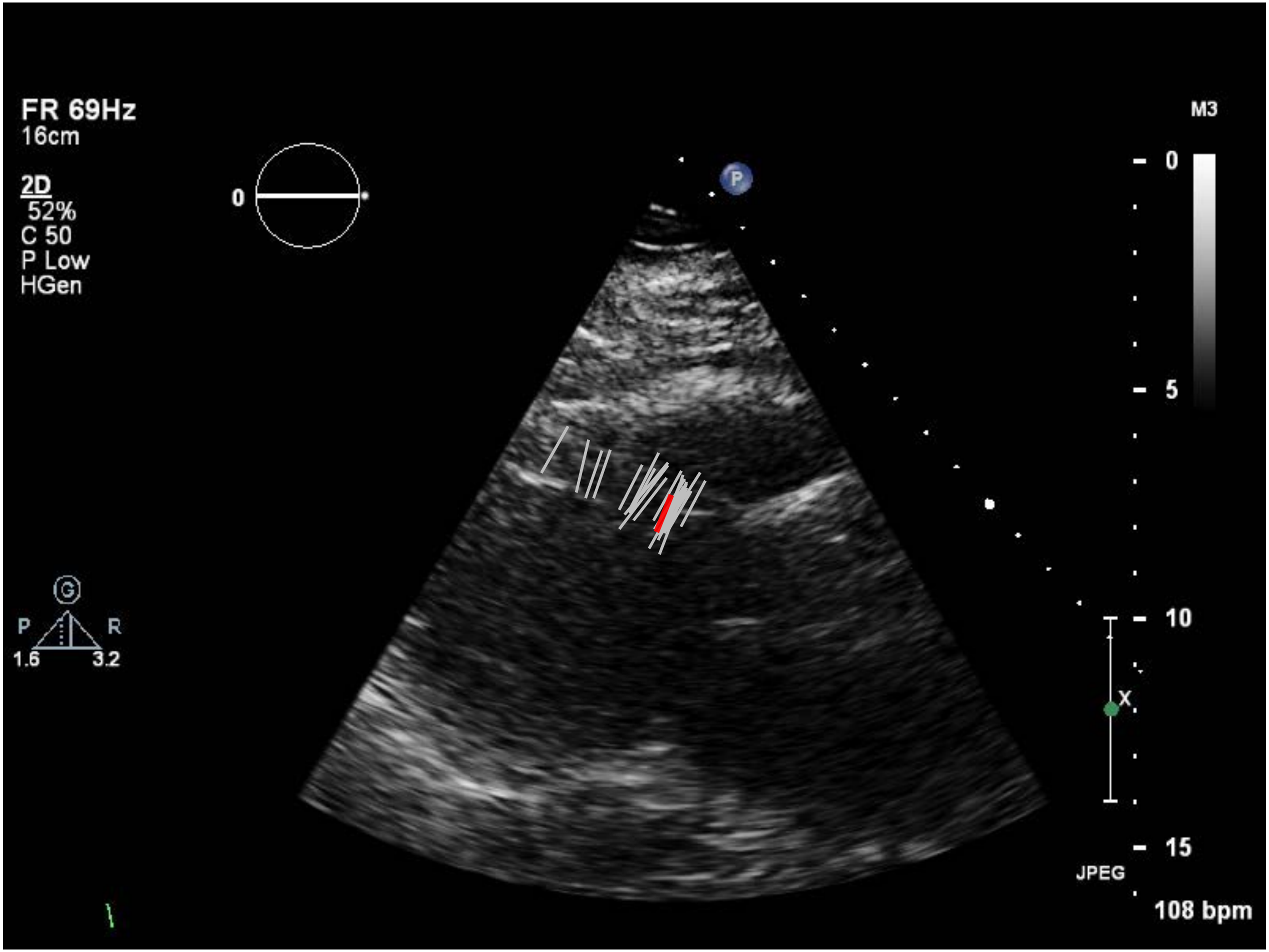

Pt 66 Diastole

Absolute difference between AI and expert consensus: 3.8 mm

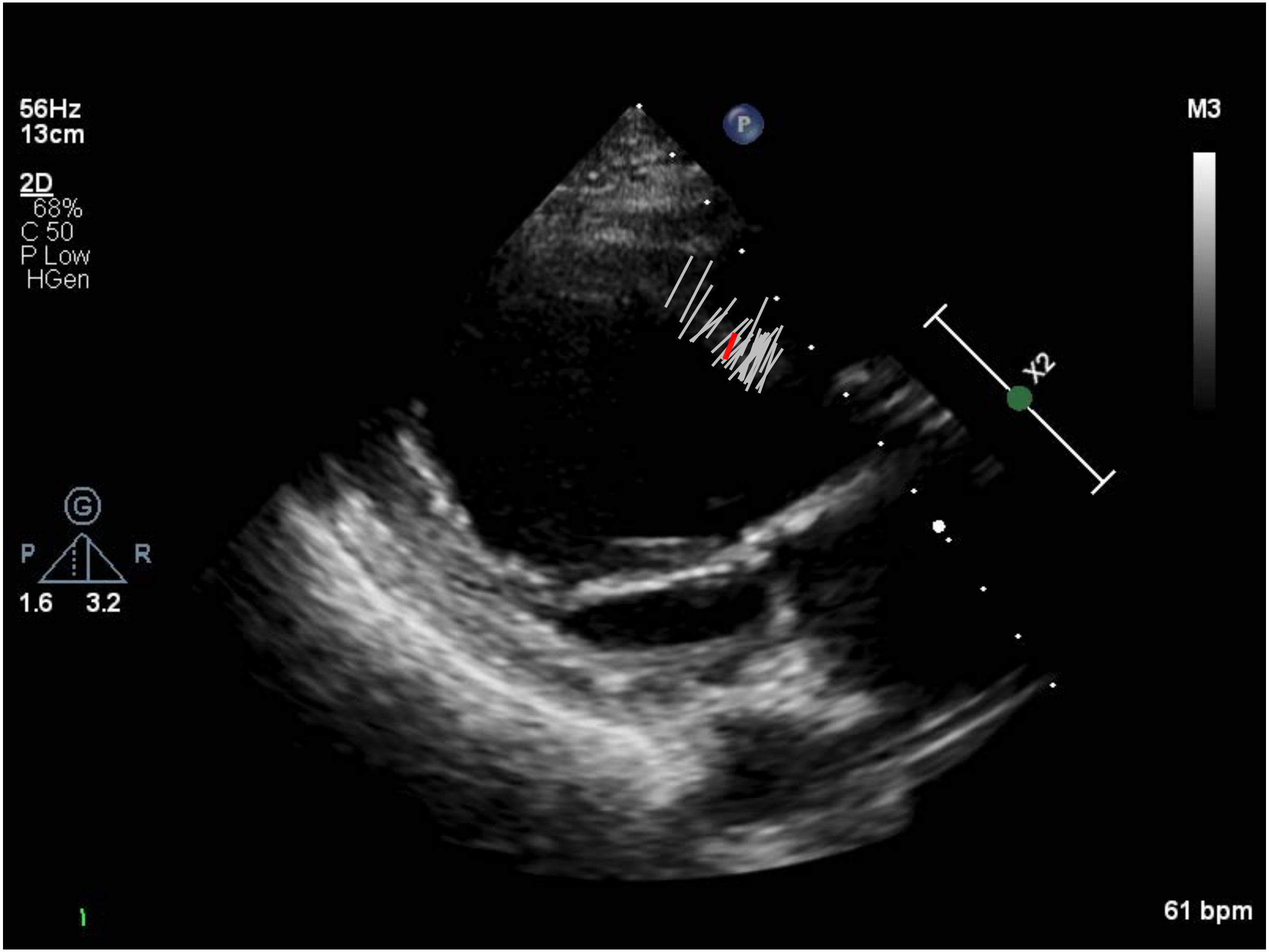

Pt 44 Diastole

Absolute difference between AI and expert consensus: 4 mm

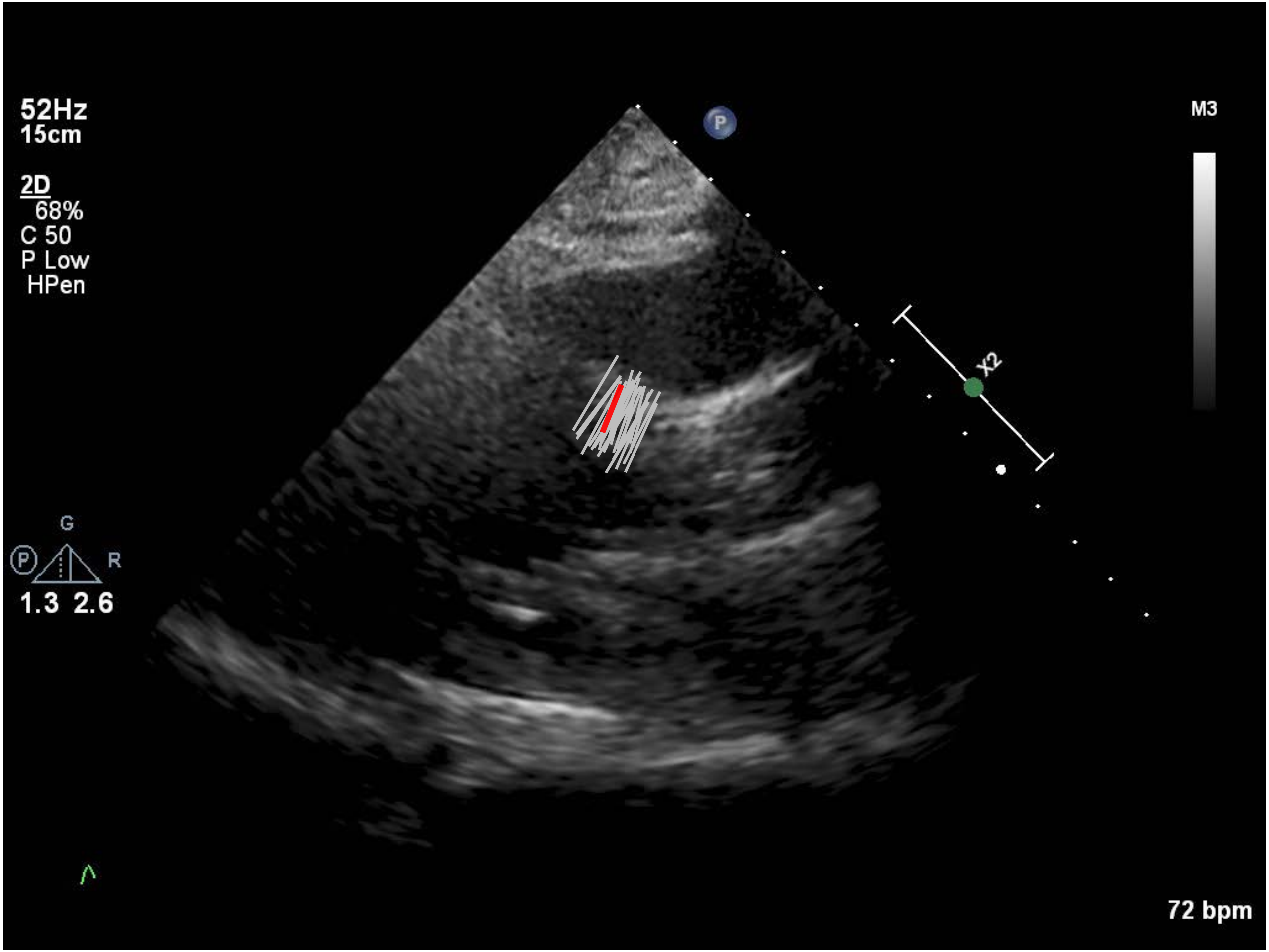

Pt 6 Diastole

Absolute difference between AI and expert consensus: 4.5 mm

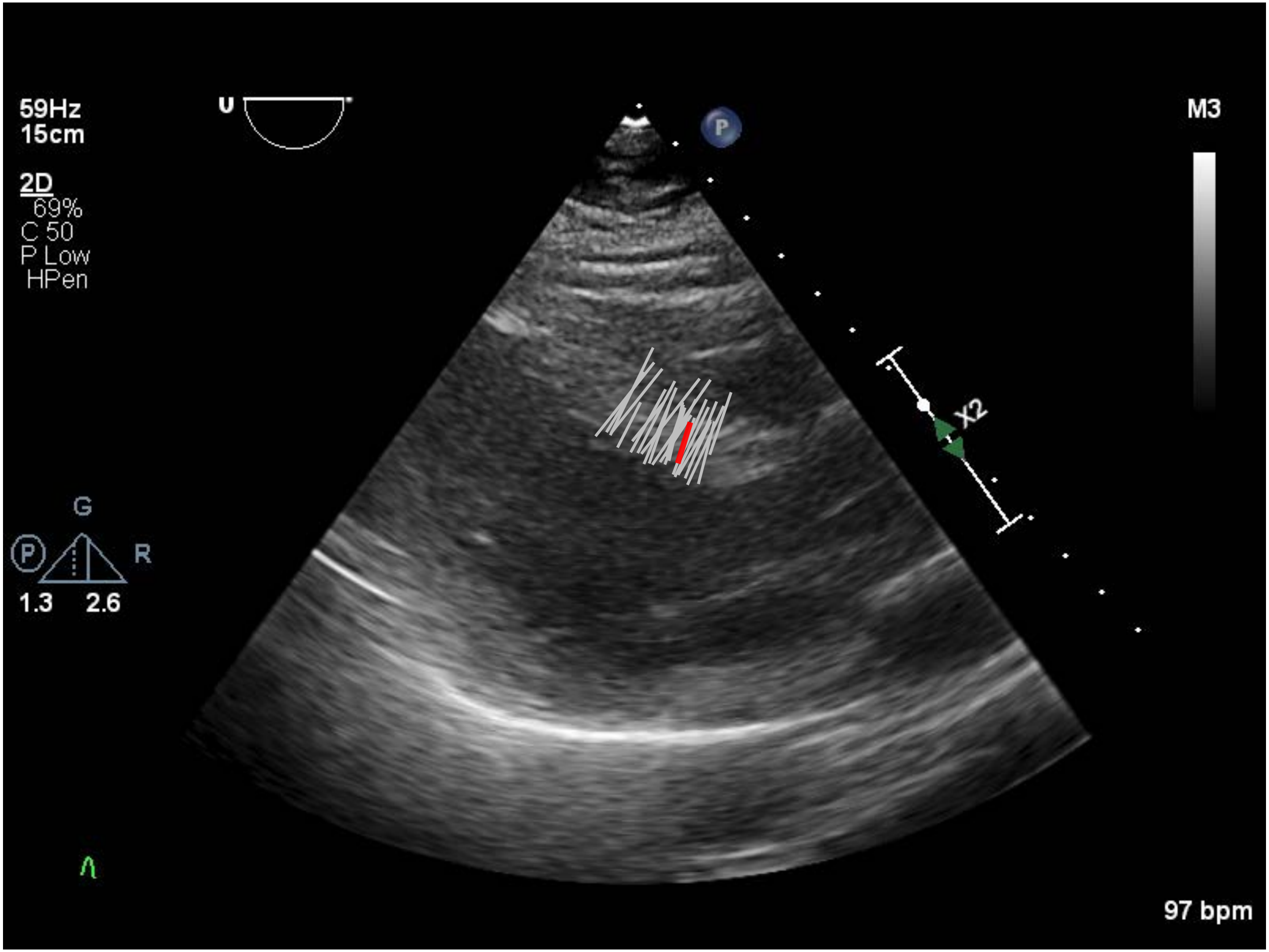

Pt 39 Diastole

Absolute difference between AI and expert consensus: 5.1 mm

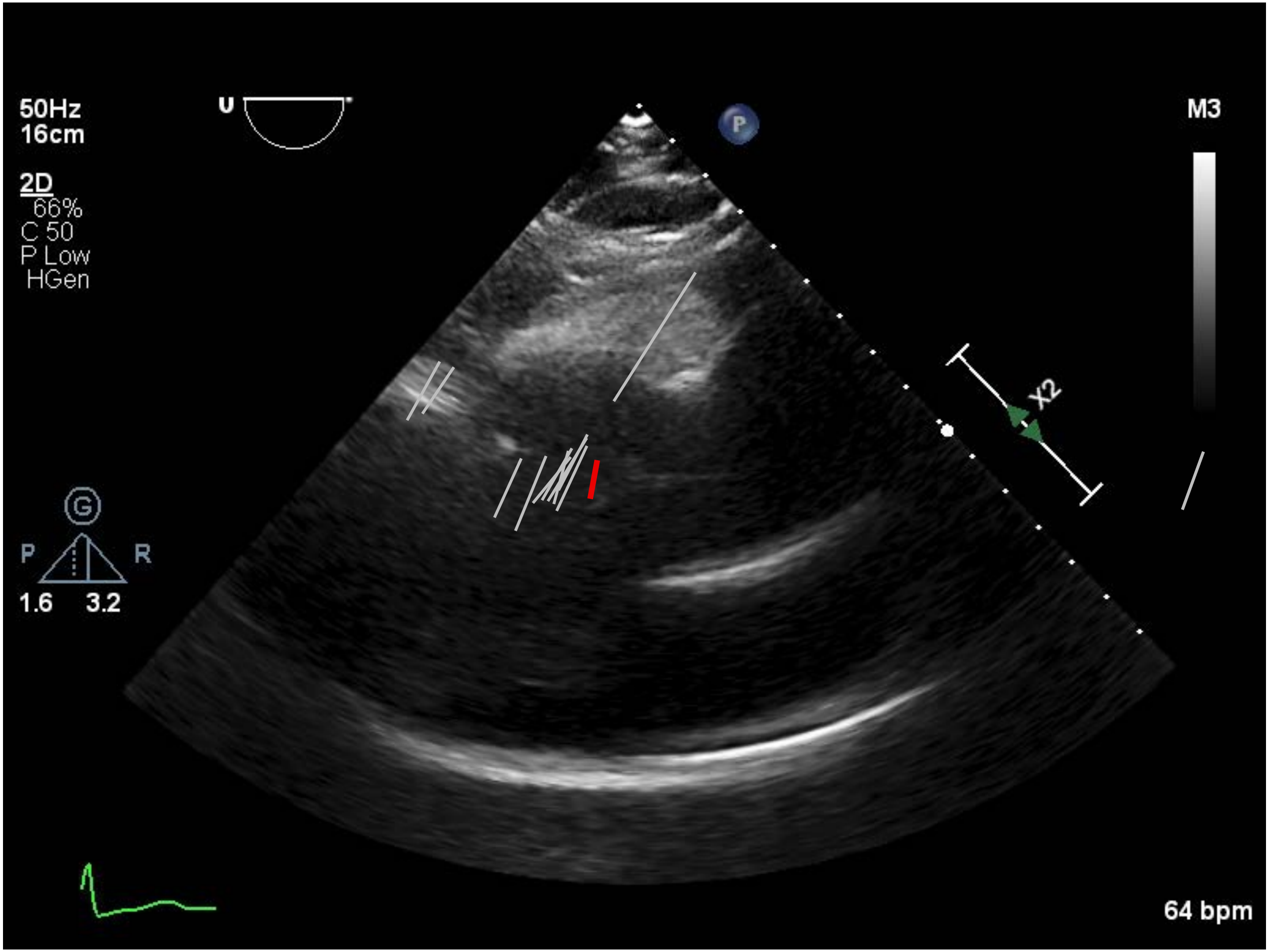

Pt 20 Diastole

Absolute difference between AI and expert consensus: 5.2 mm

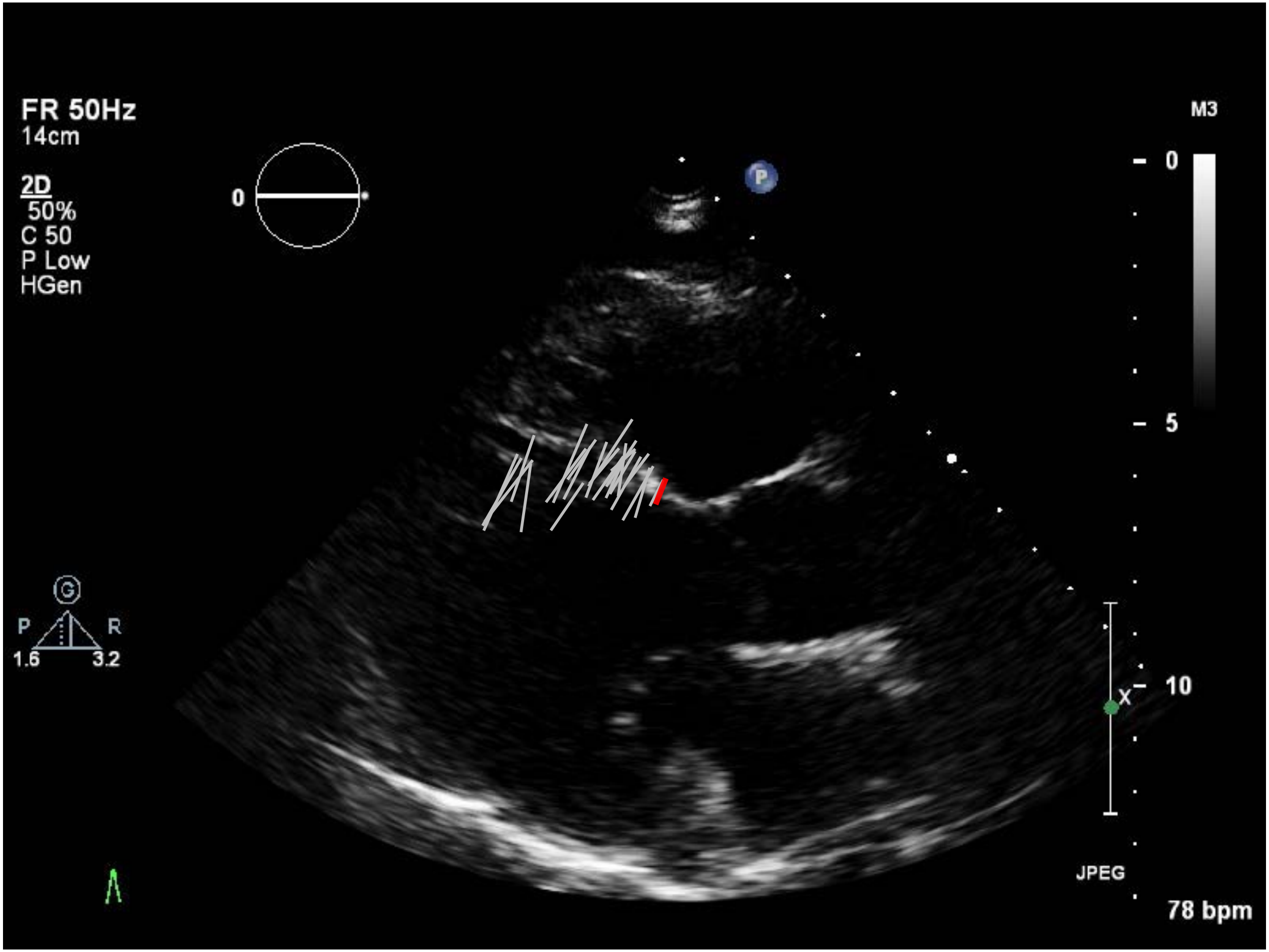

Pt 50 Diastole

Absolute difference between AI and expert consensus: 5.4 mm

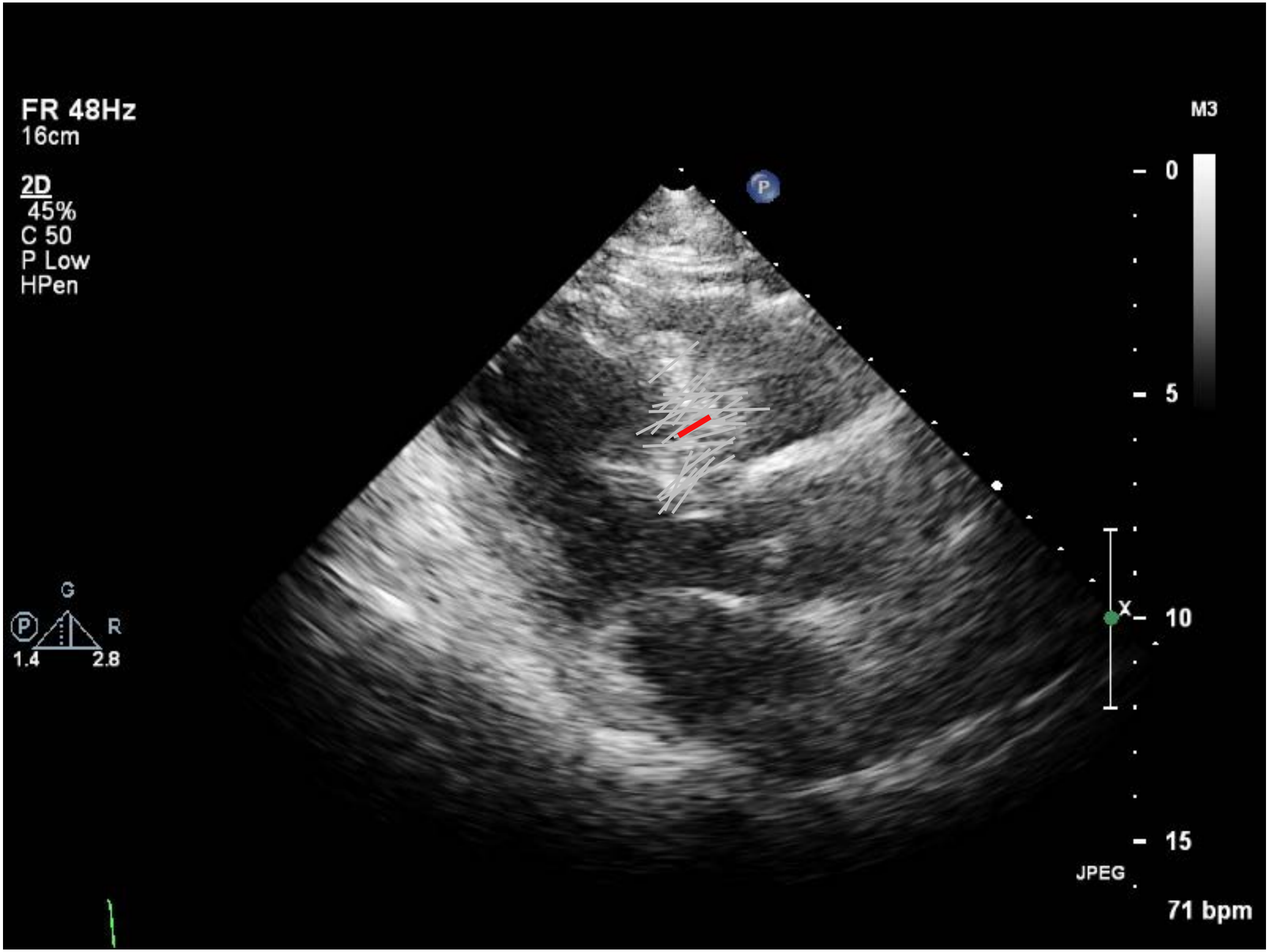

Pt 71 Diastole

Absolute difference between AI and expert consensus: 7.6 mm

Supplemental Figure VIII: Full disclosure PWd measurement dataset

The following 200 images show the AI (red) and expert (grey) posterior wall distance (PWd) measurements on the end-diastolic and end-systolic frames of the 100 validation images. The images are ordered by the absolute difference between AI and expert consensus LVID measurement.

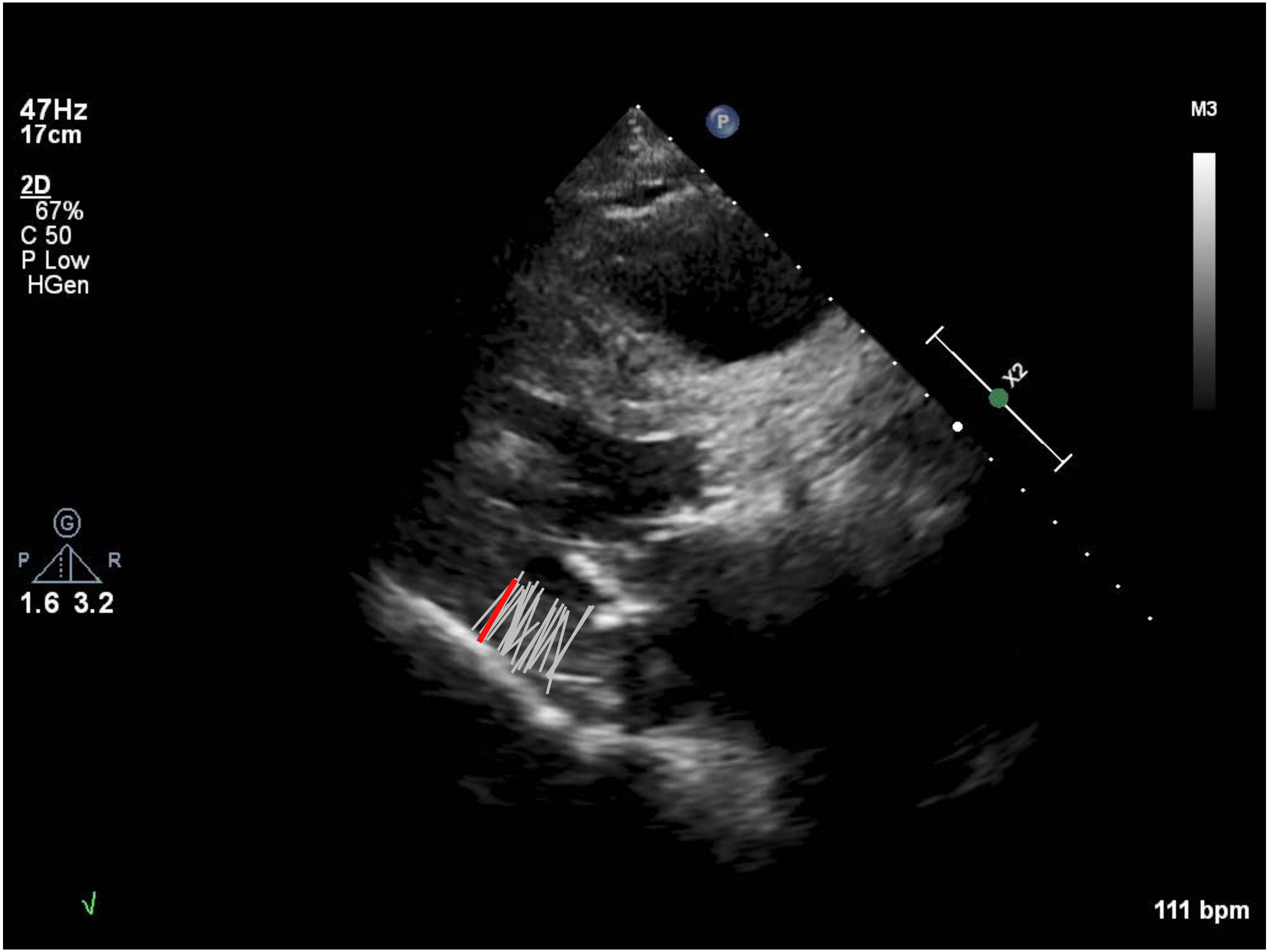

Pt 65 Diastole
Absolute difference between AI and expert consensus: 0.00064 mm

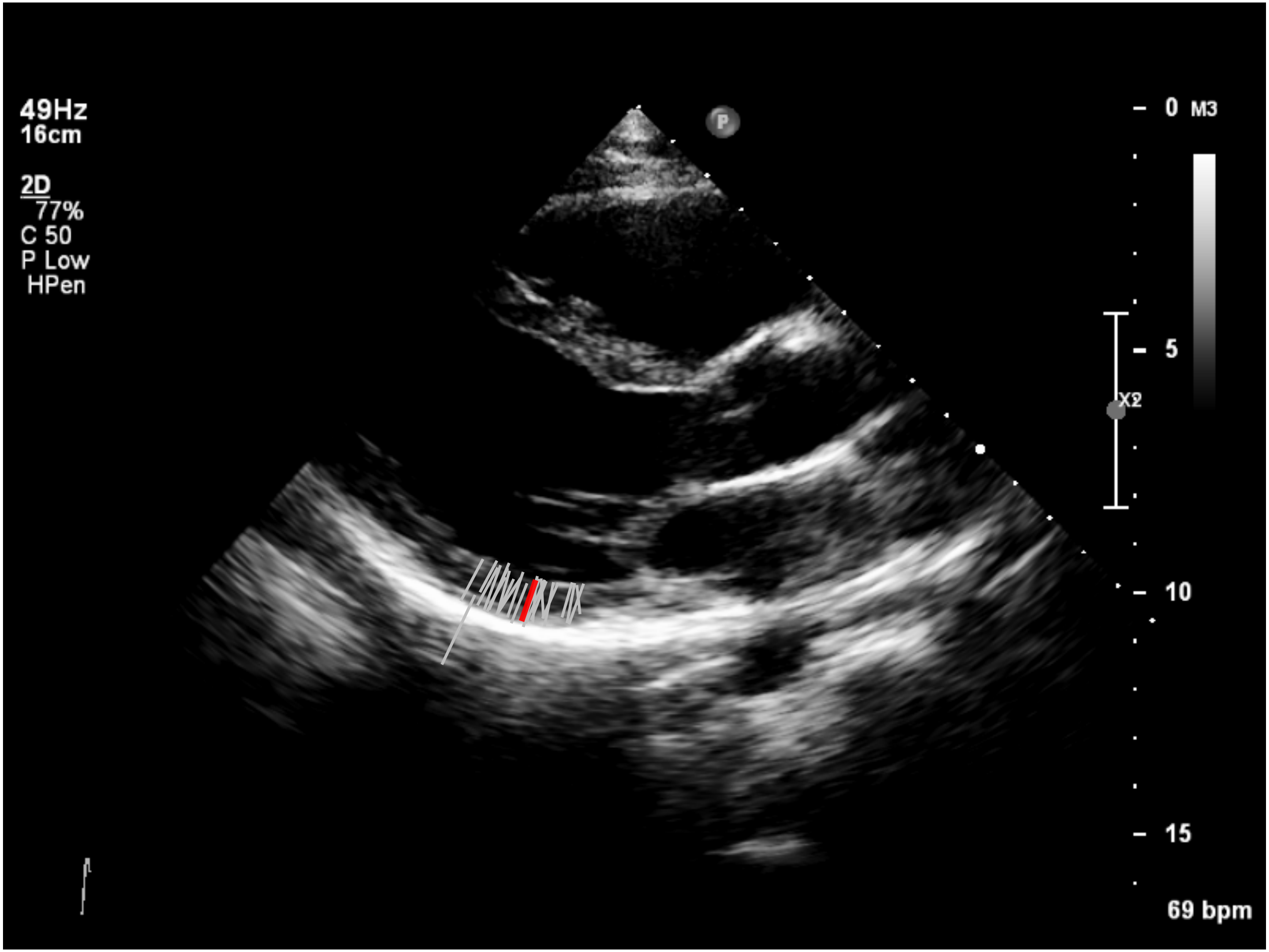

Pt 38 Diastole

Absolute difference between AI and expert consensus: 0.031 mm

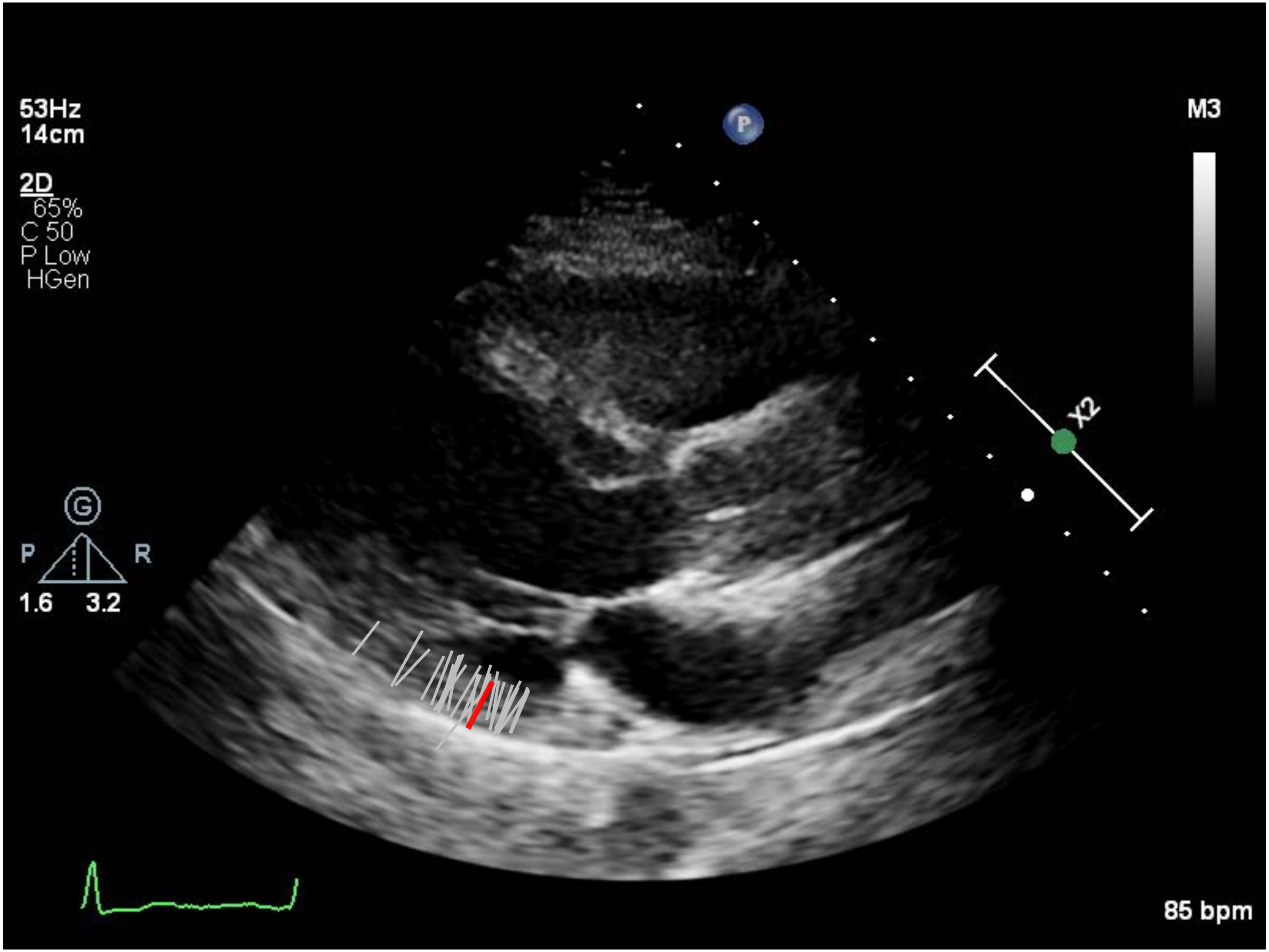

Pt 74 Diastole

Absolute difference between AI and expert consensus: 0.052 mm

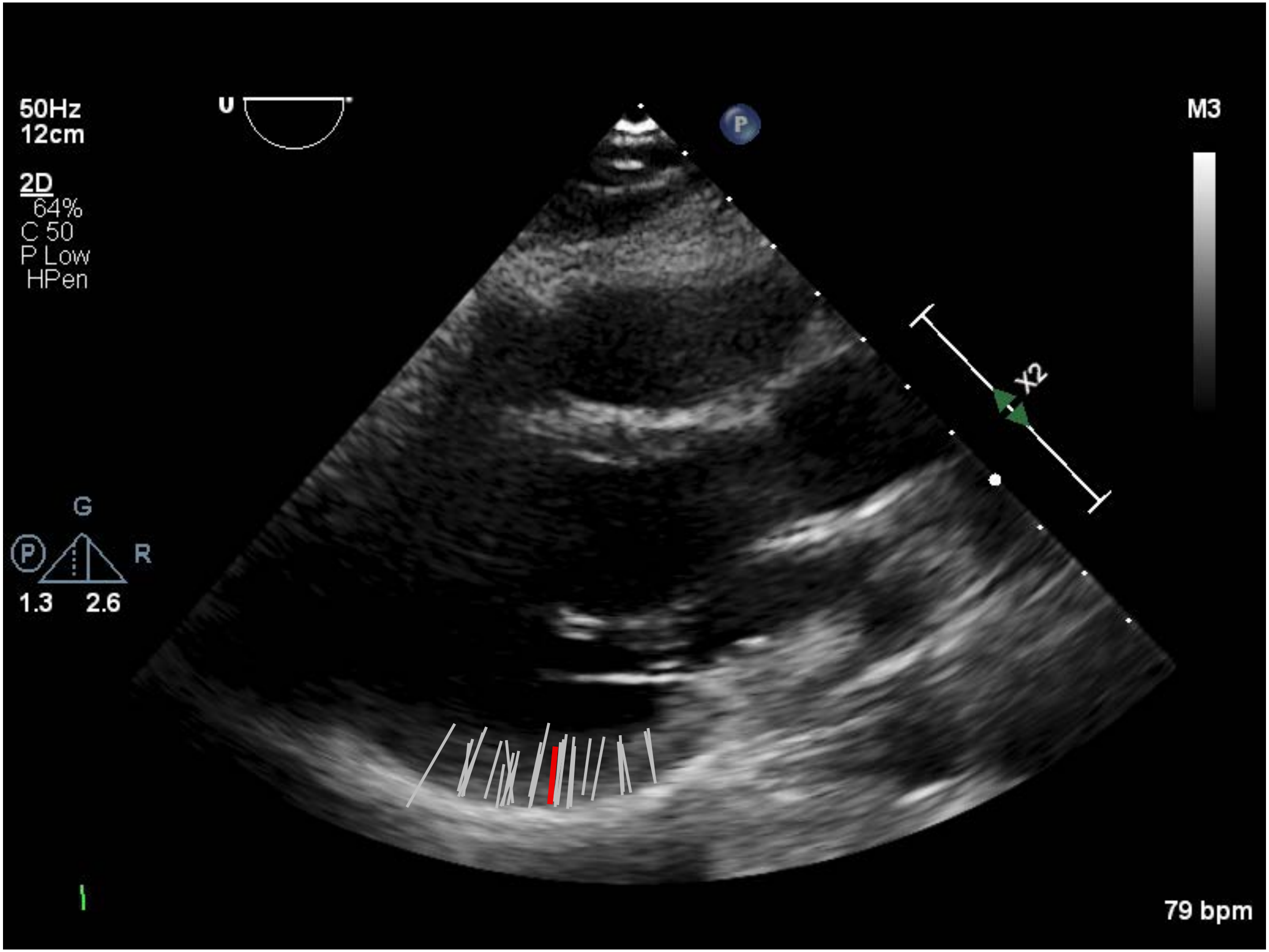

Pt 55 Diastole

Absolute difference between AI and expert consensus: 0.056 mm

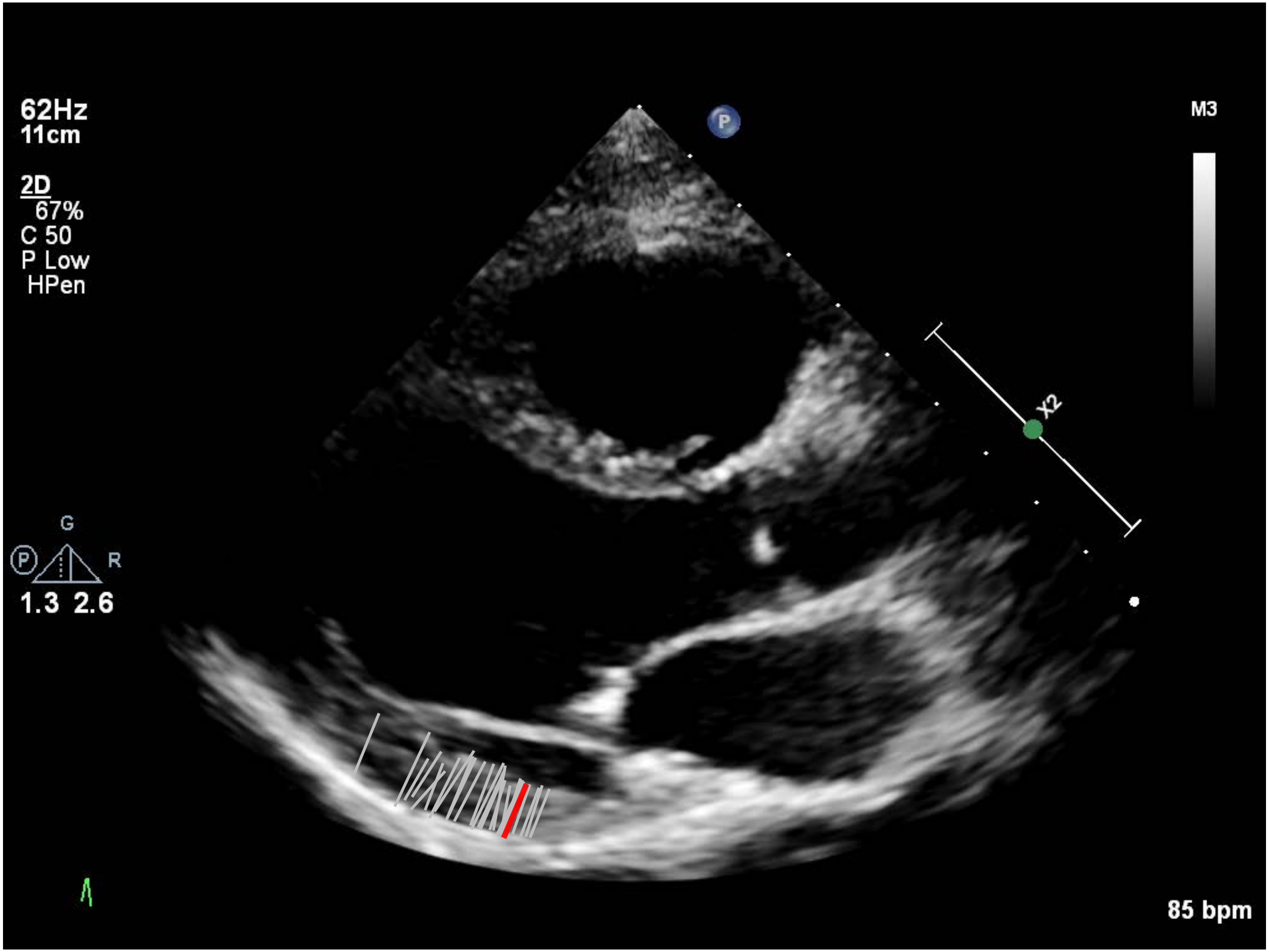

Pt 2 Diastole

Absolute difference between AI and expert consensus: 0.075 mm

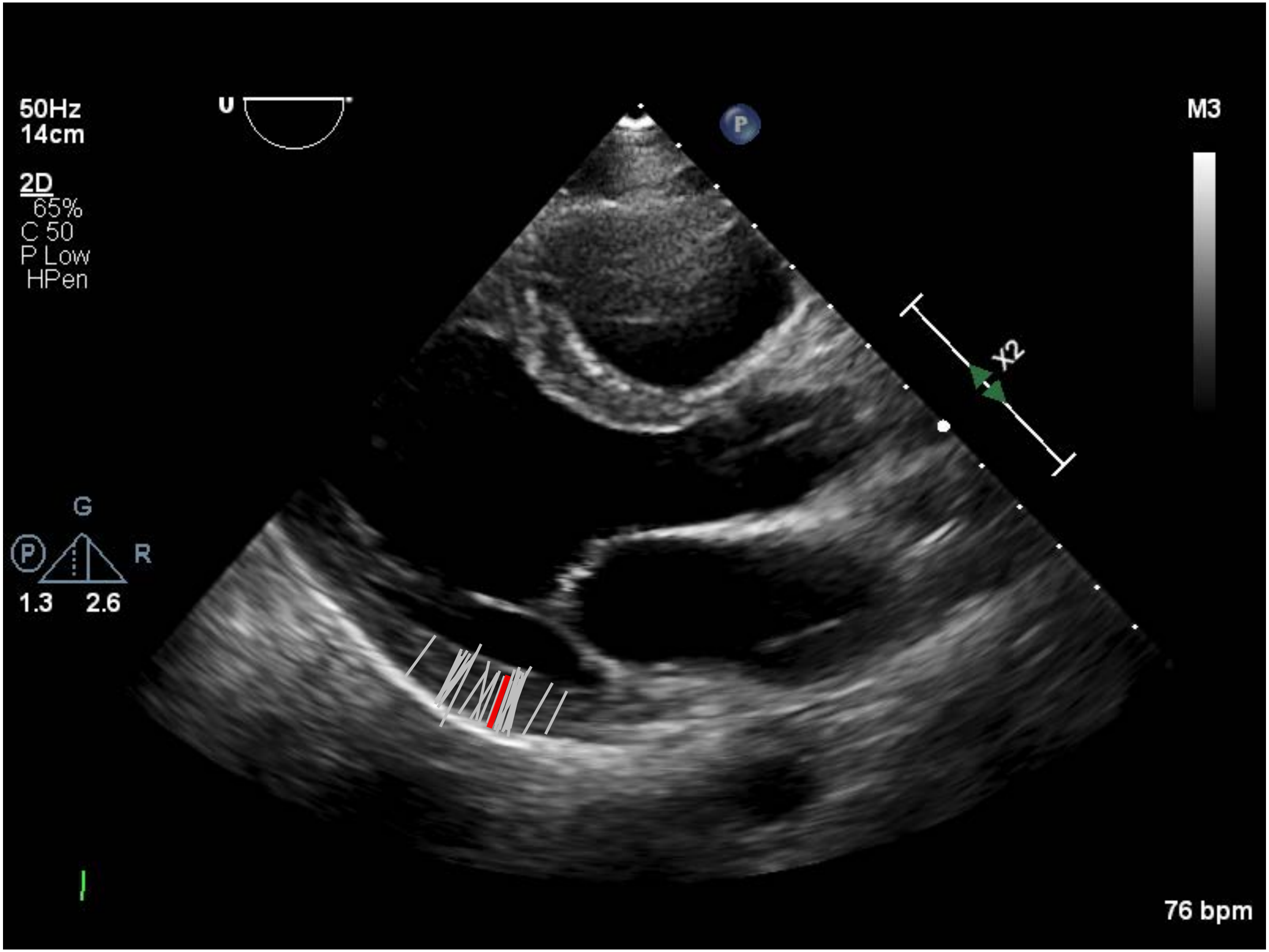

Pt 25 Diastole

Absolute difference between AI and expert consensus: 0.082 mm

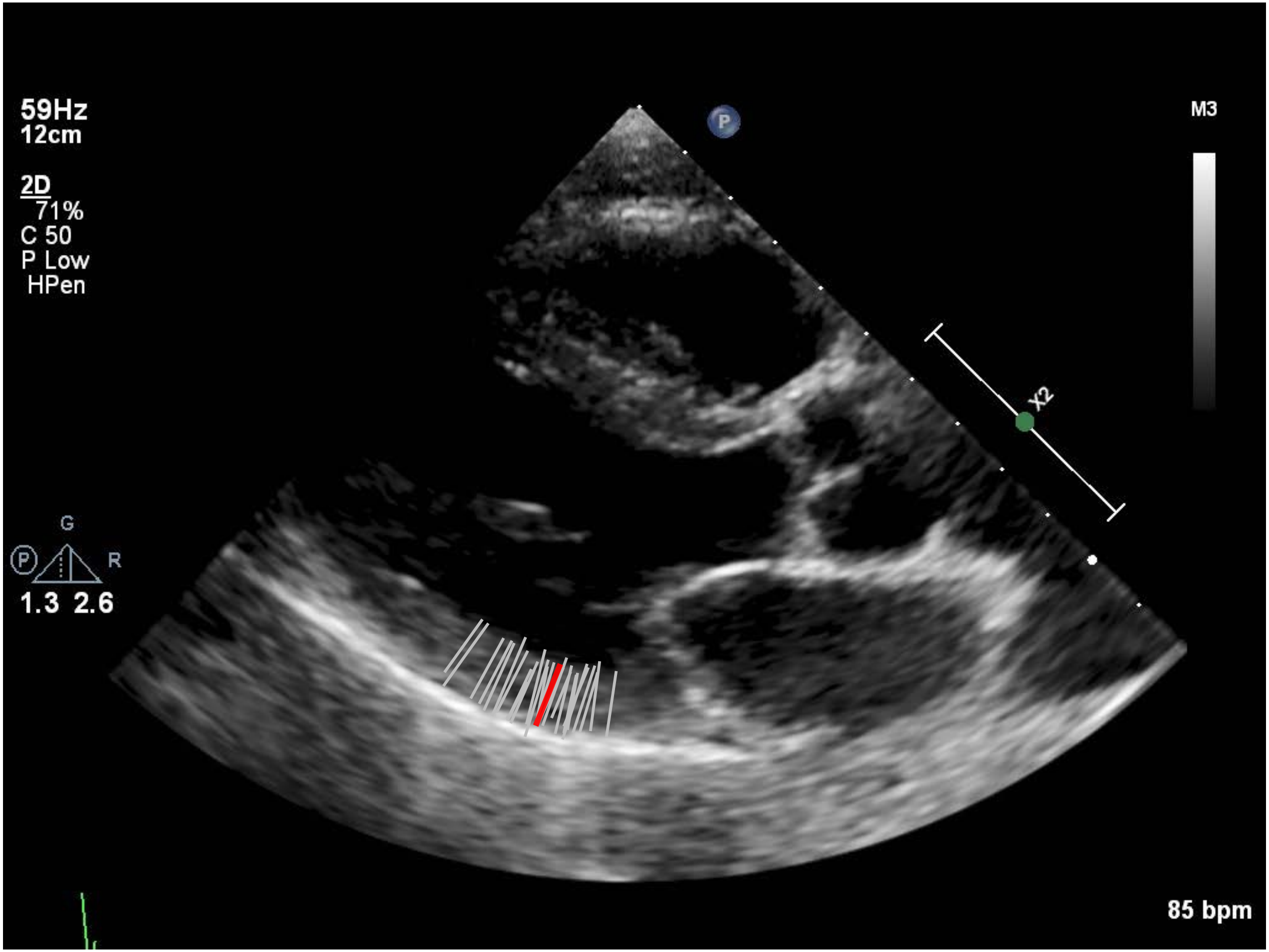

Pt 75 Diastole

Absolute difference between AI and expert consensus: 0.086 mm

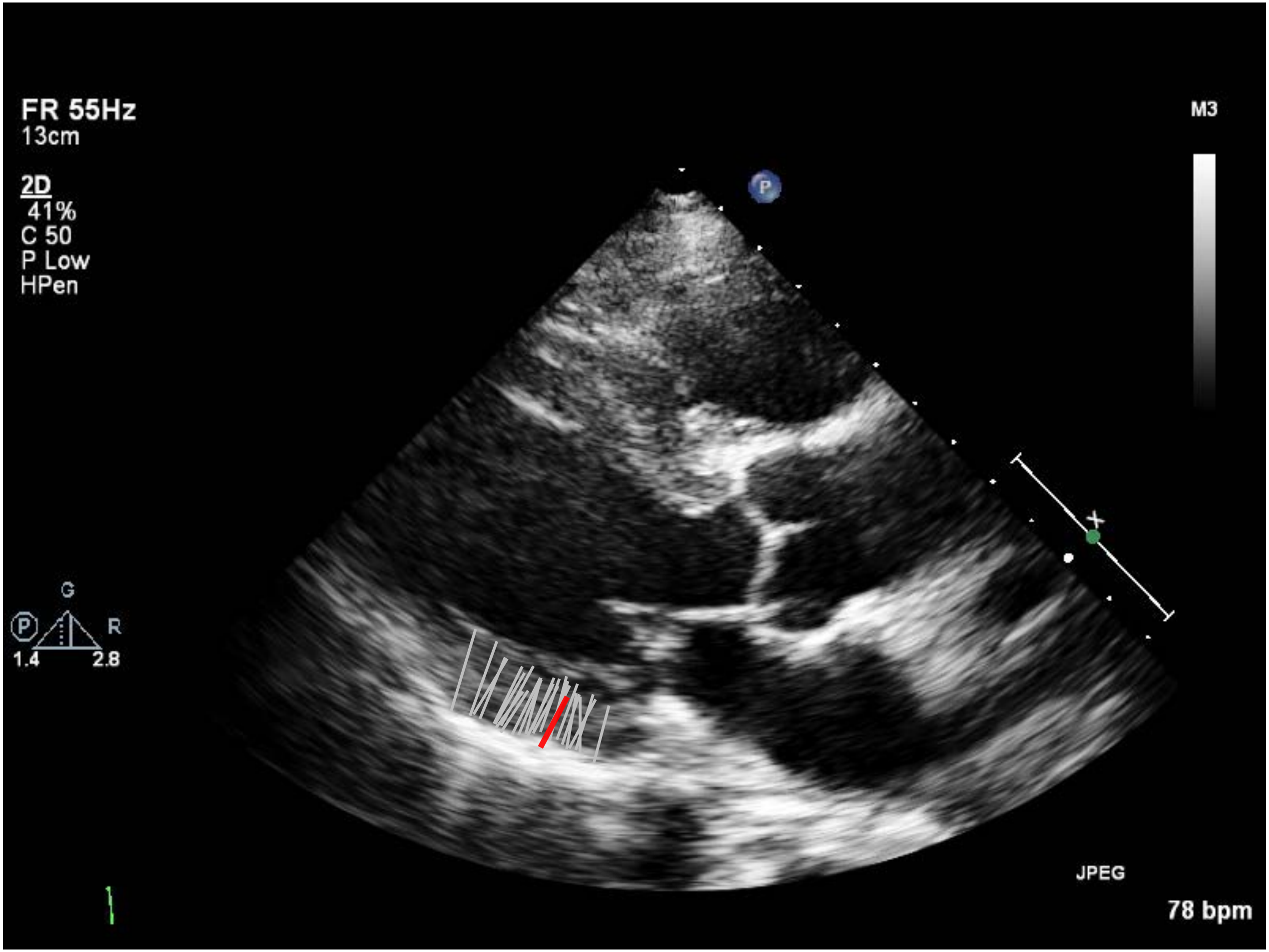

Pt 31 Diastole

Absolute difference between AI and expert consensus: 0.11 mm

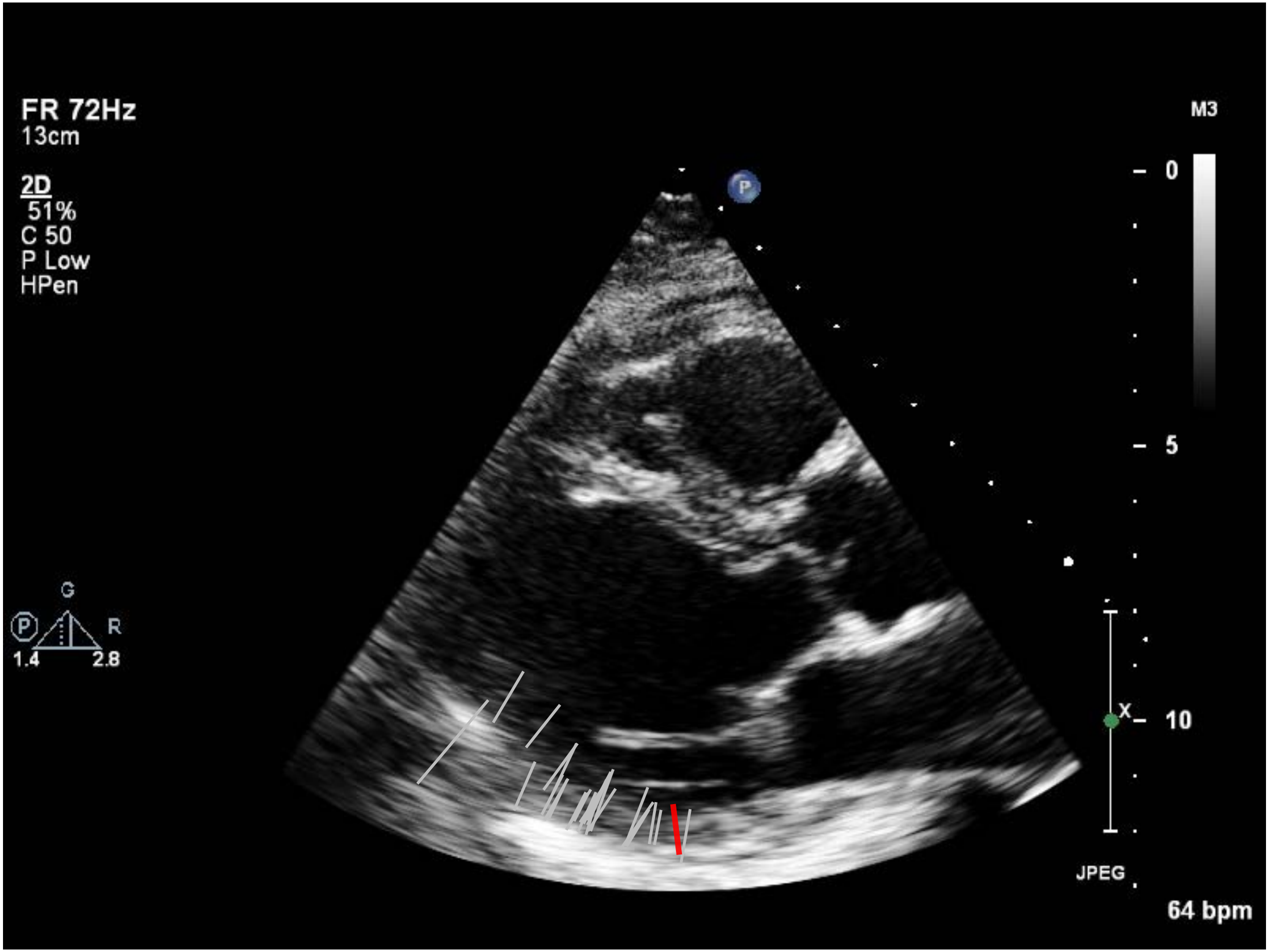

Pt 48 Diastole

Absolute difference between AI and expert consensus: 0.14 mm

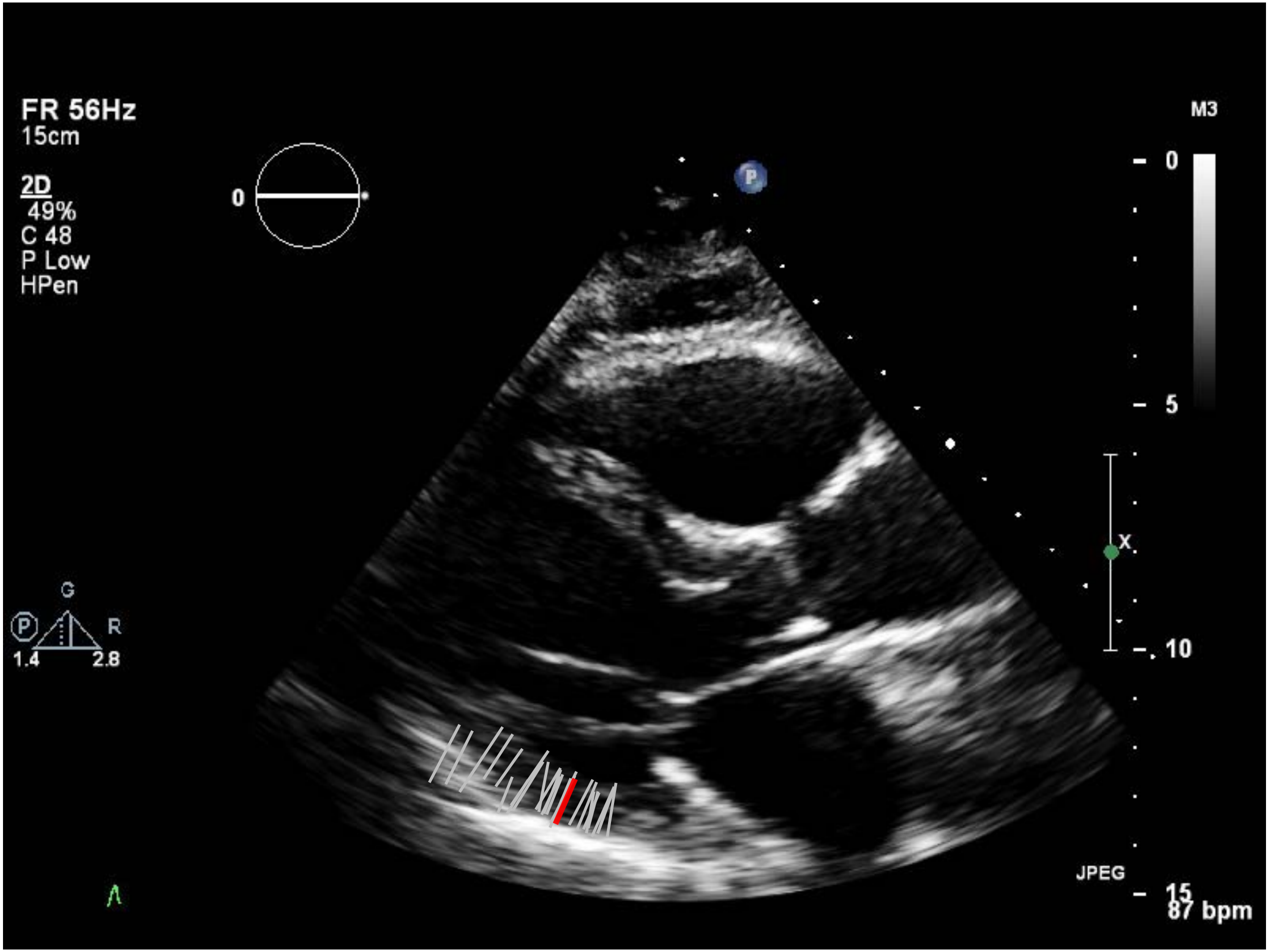

Pt 21 Diastole

Absolute difference between AI and expert consensus: 0.15 mm

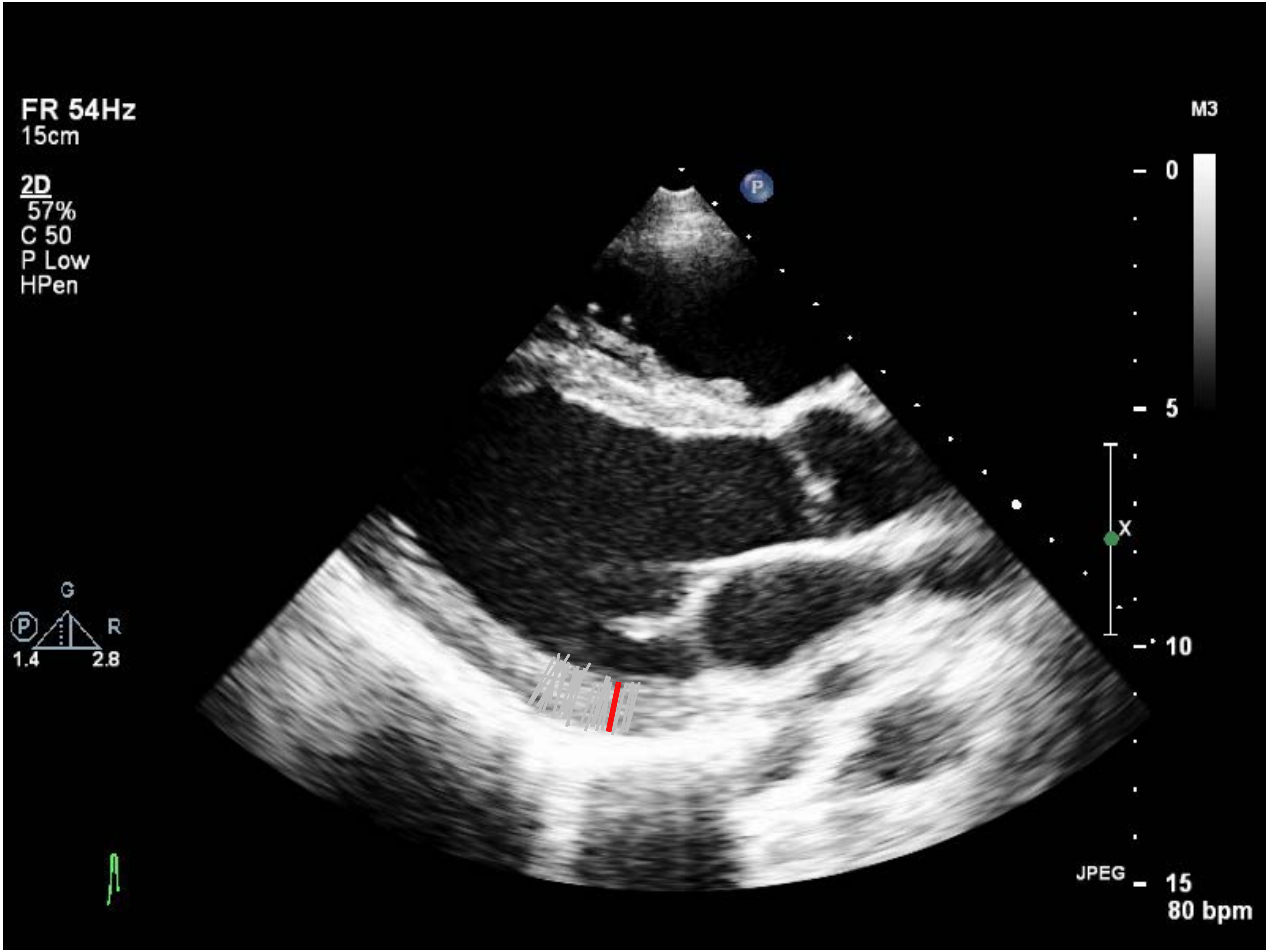

Pt 82 Diastole

Absolute difference between AI and expert consensus: 0.16 mm

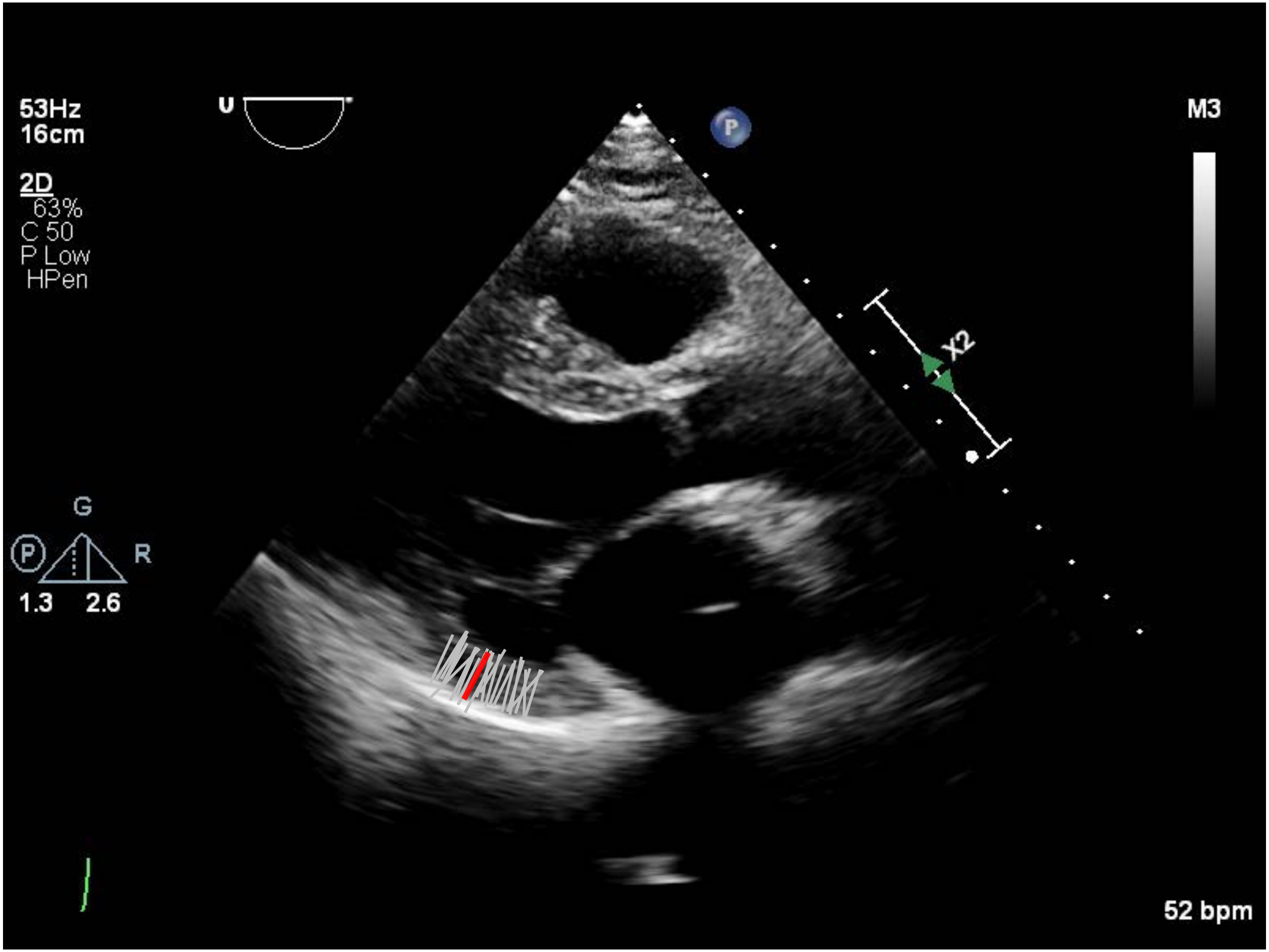

Pt 8 Diastole

Absolute difference between AI and expert consensus: 0.16 mm

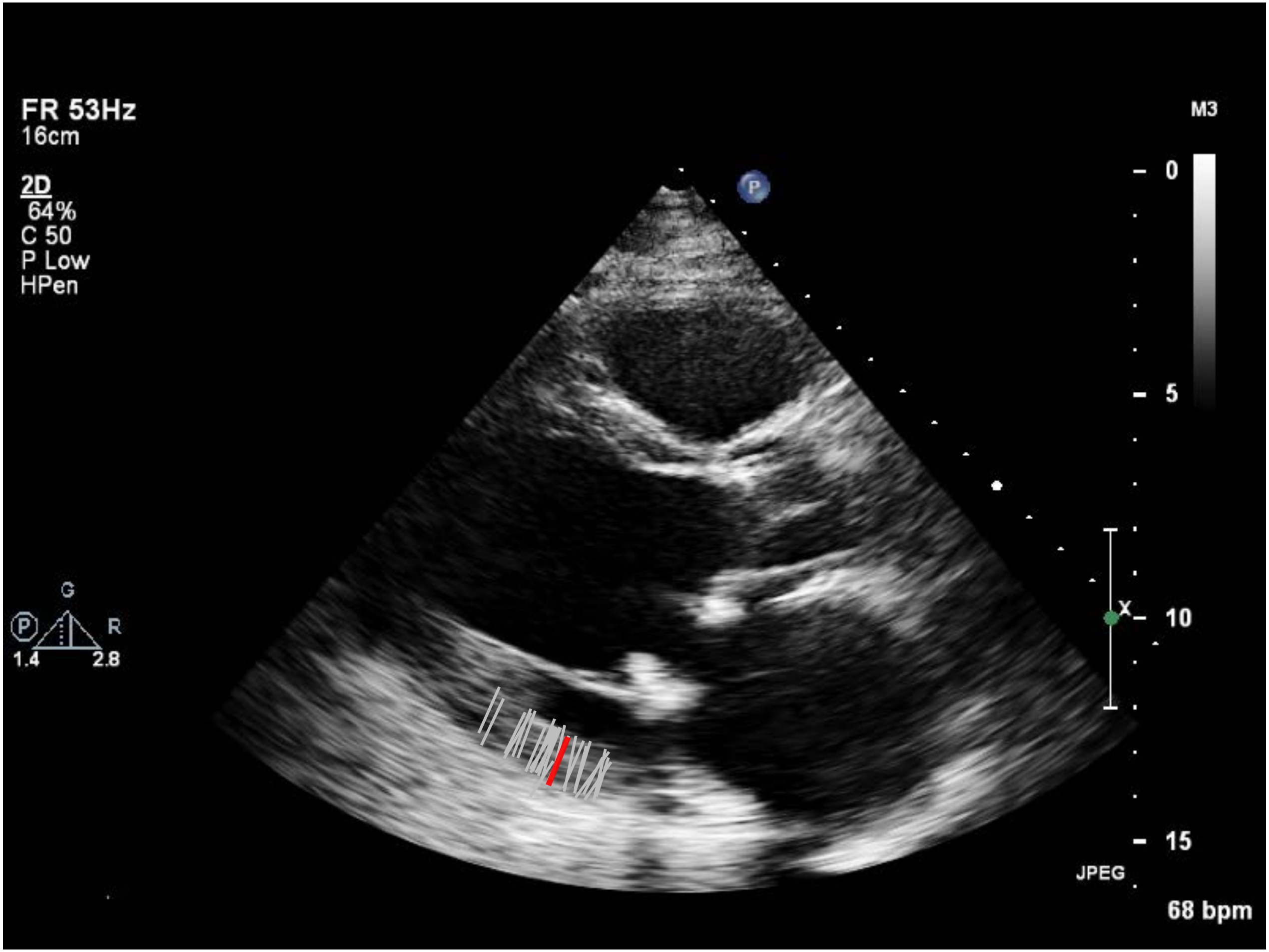

Pt 56 Diastole

Absolute difference between AI and expert consensus: 0.22 mm

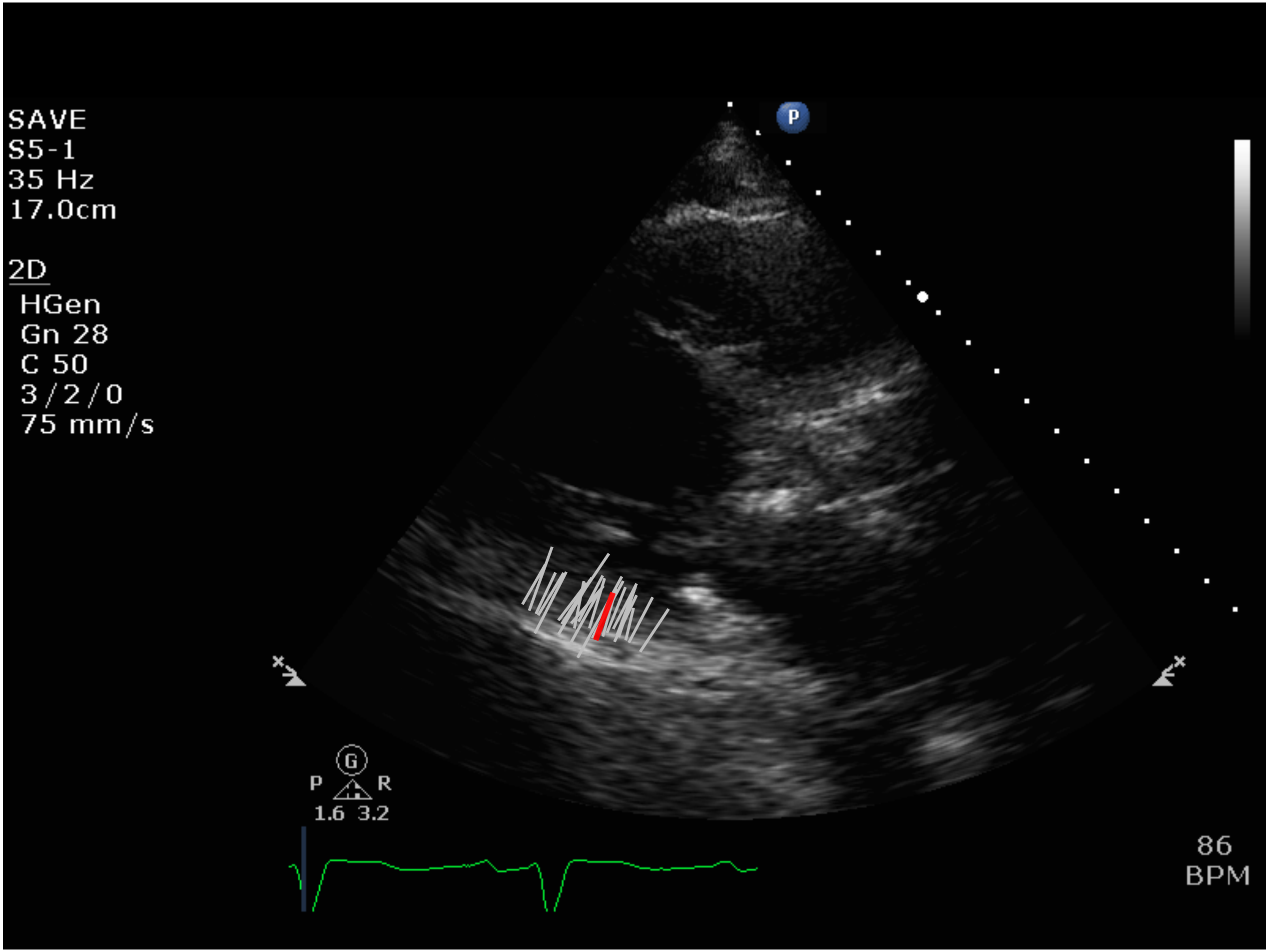

Pt 18 Diastole

Absolute difference between AI and expert consensus: 0.22 mm

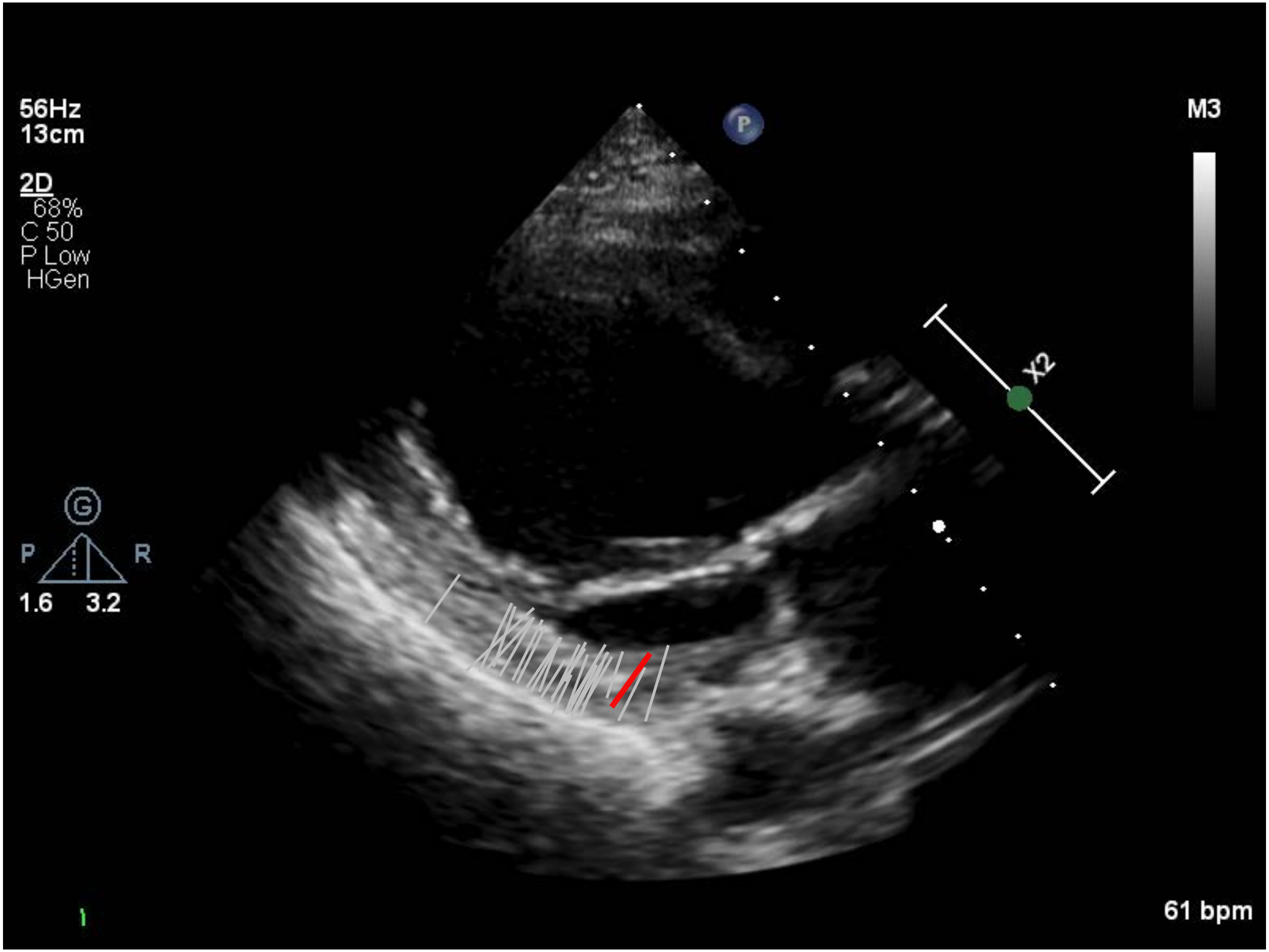

Pt 44 Diastole

Absolute difference between AI and expert consensus: 0.26 mm

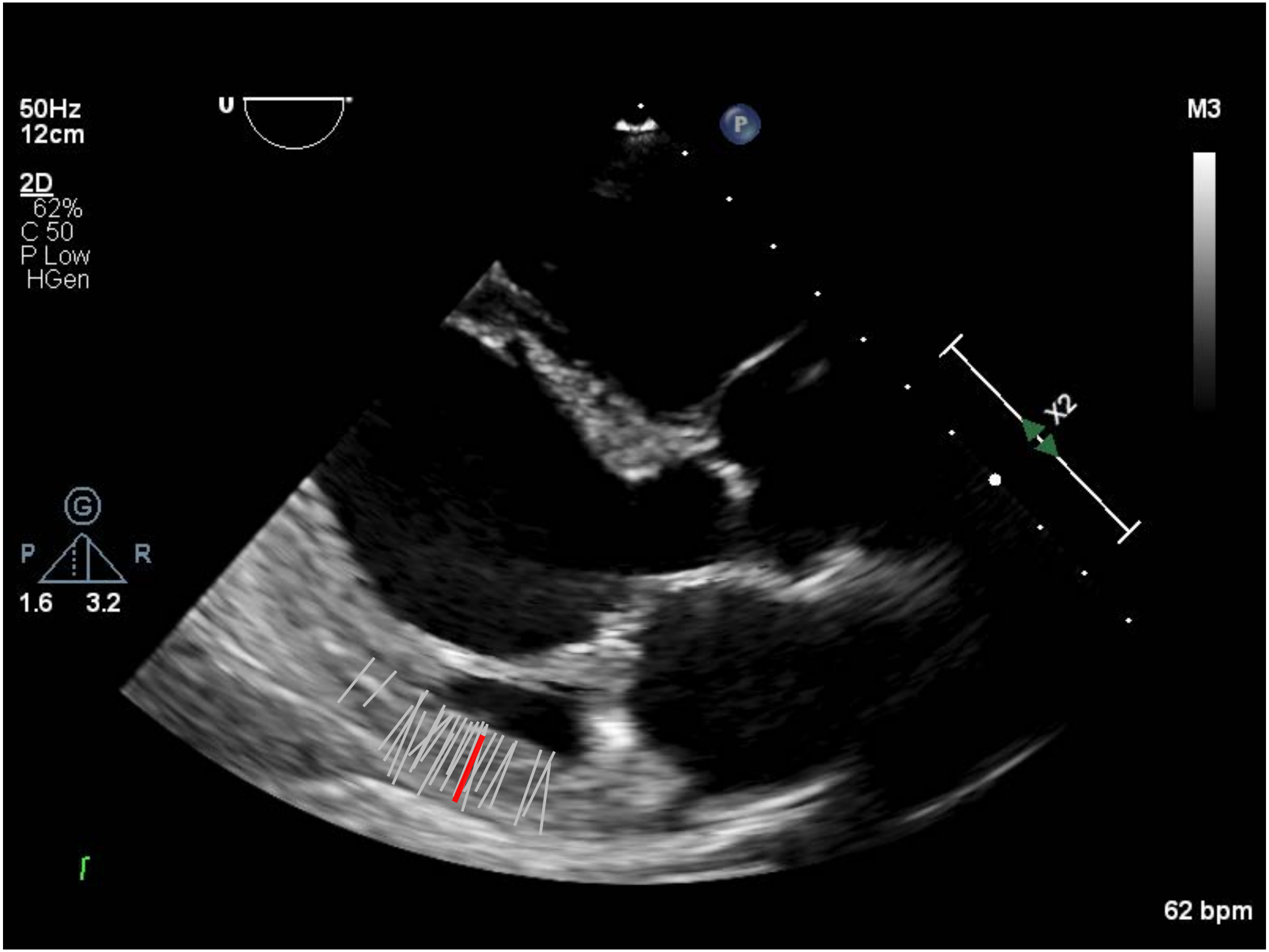

Pt 9 Diastole

Absolute difference between AI and expert consensus: 0.28 mm

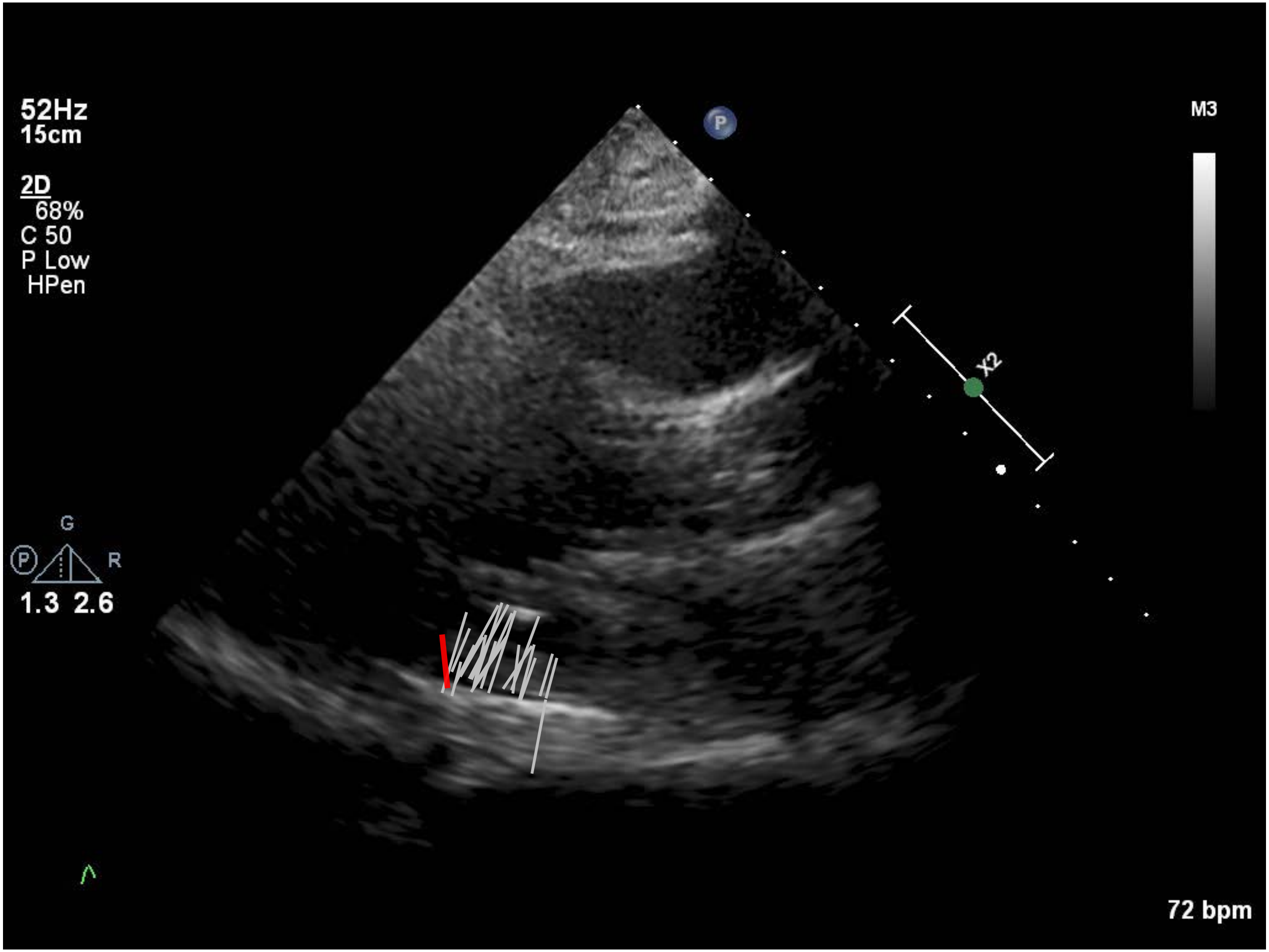

Pt 6 Diastole
Absolute difference between AI and expert consensus: 0.29 mm

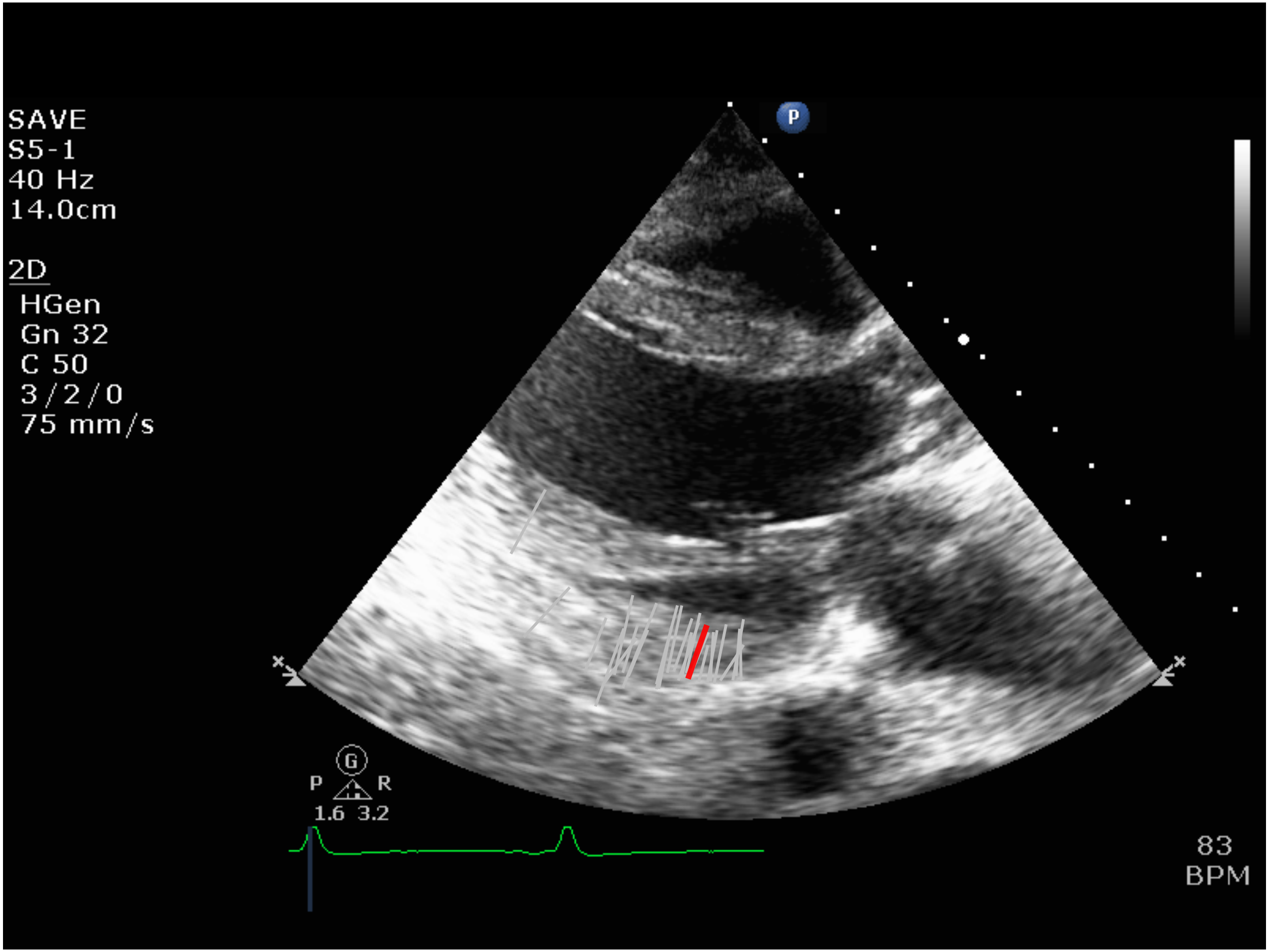

Pt 84 Diastole

Absolute difference between AI and expert consensus: 0.32 mm

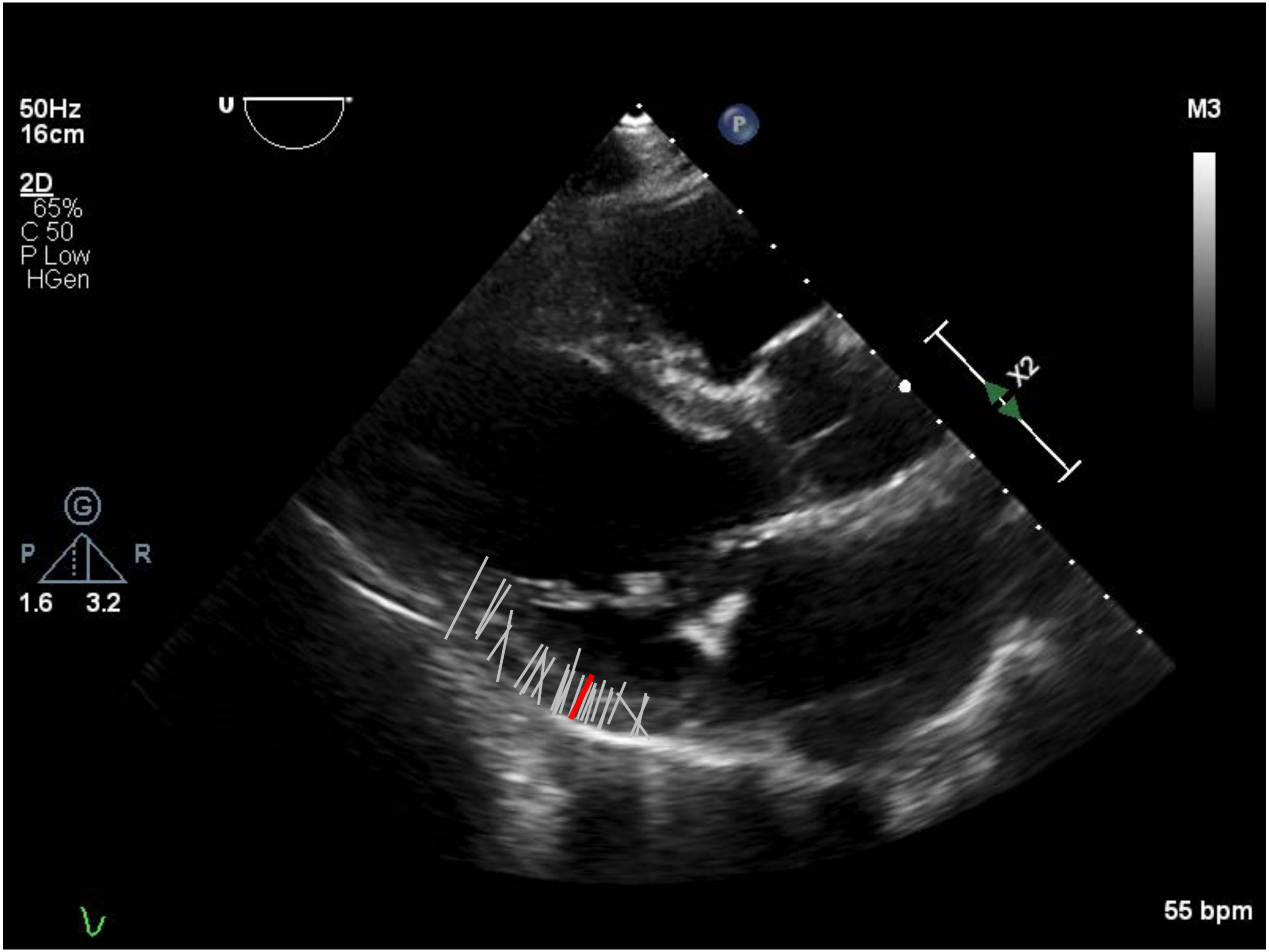

Pt 78 Diastole

Absolute difference between AI and expert consensus: 0.37 mm

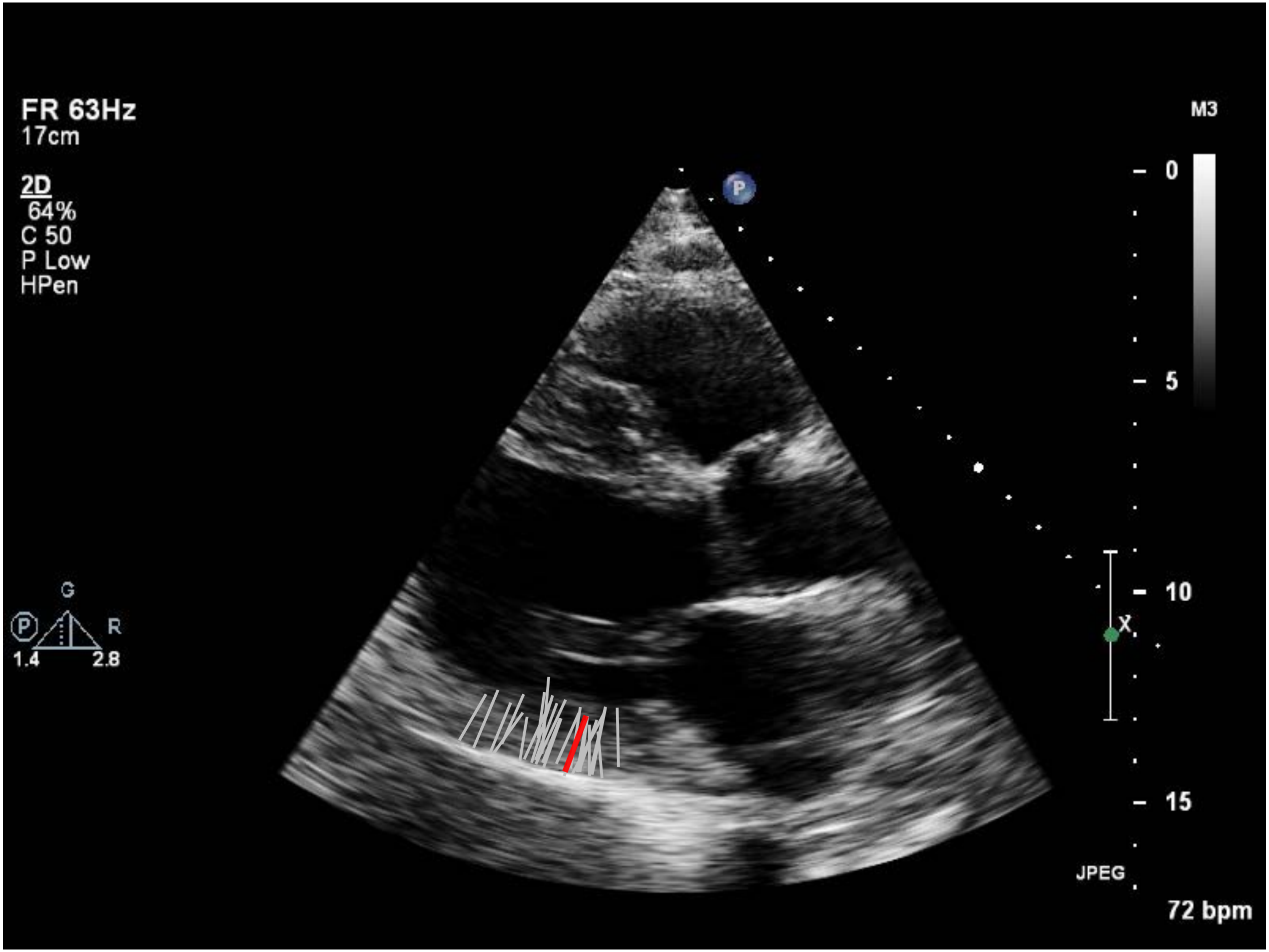

Pt 64 Diastole

Absolute difference between AI and expert consensus: 0.4 mm

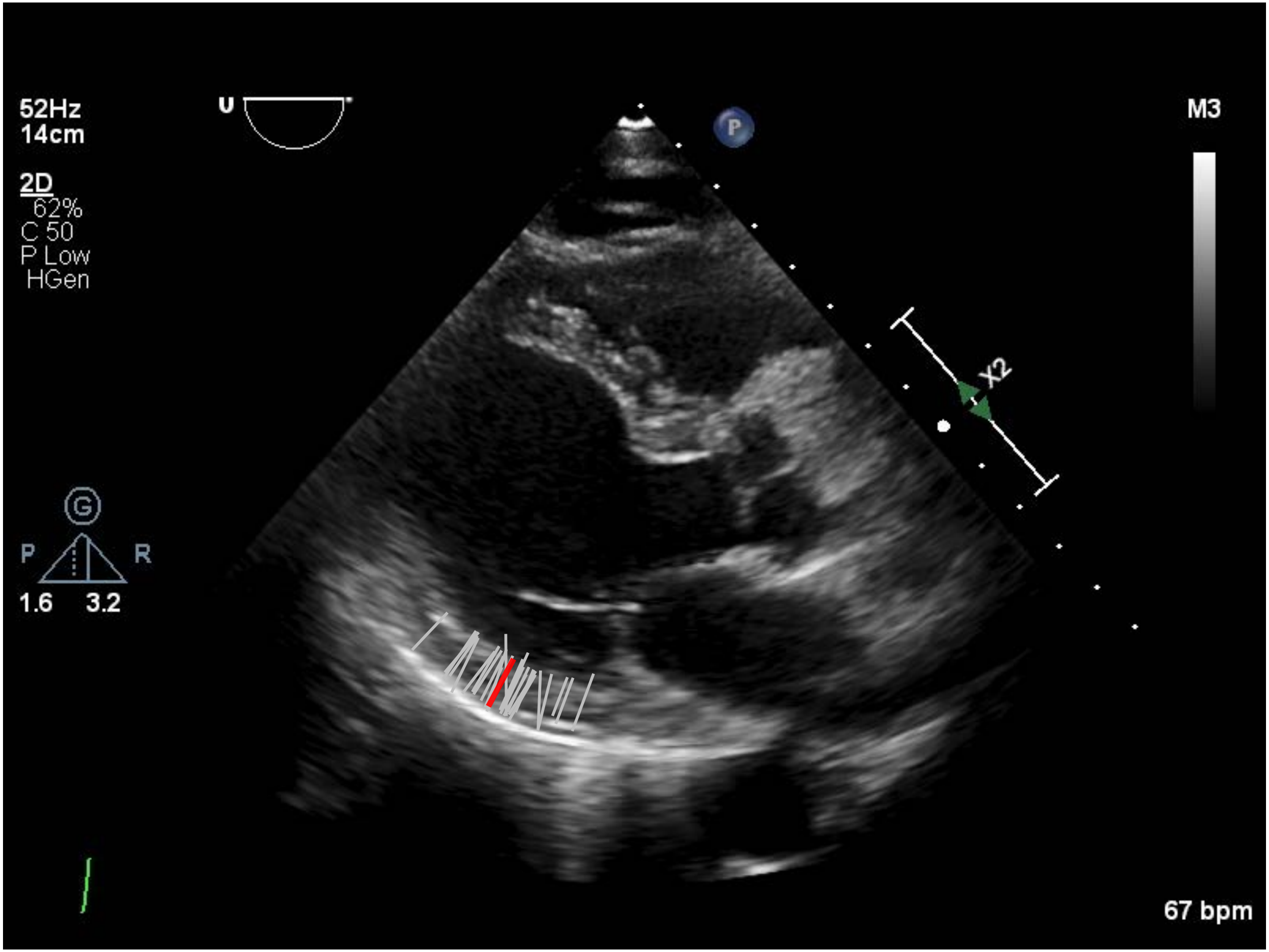

Pt 80 Diastole

Absolute difference between AI and expert consensus: 0.43 mm

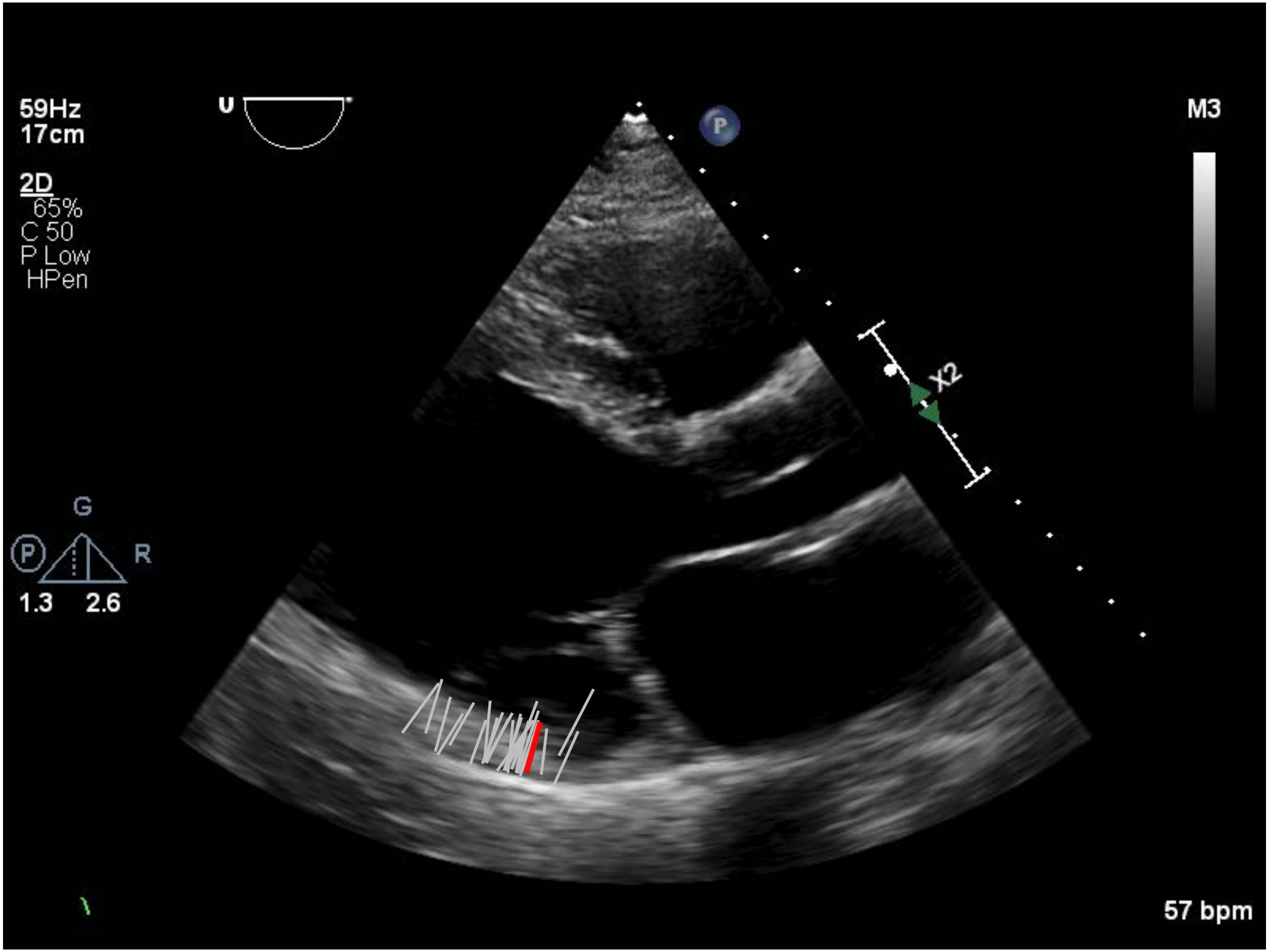

Pt 79 Diastole

Absolute difference between AI and expert consensus: 0.43 mm

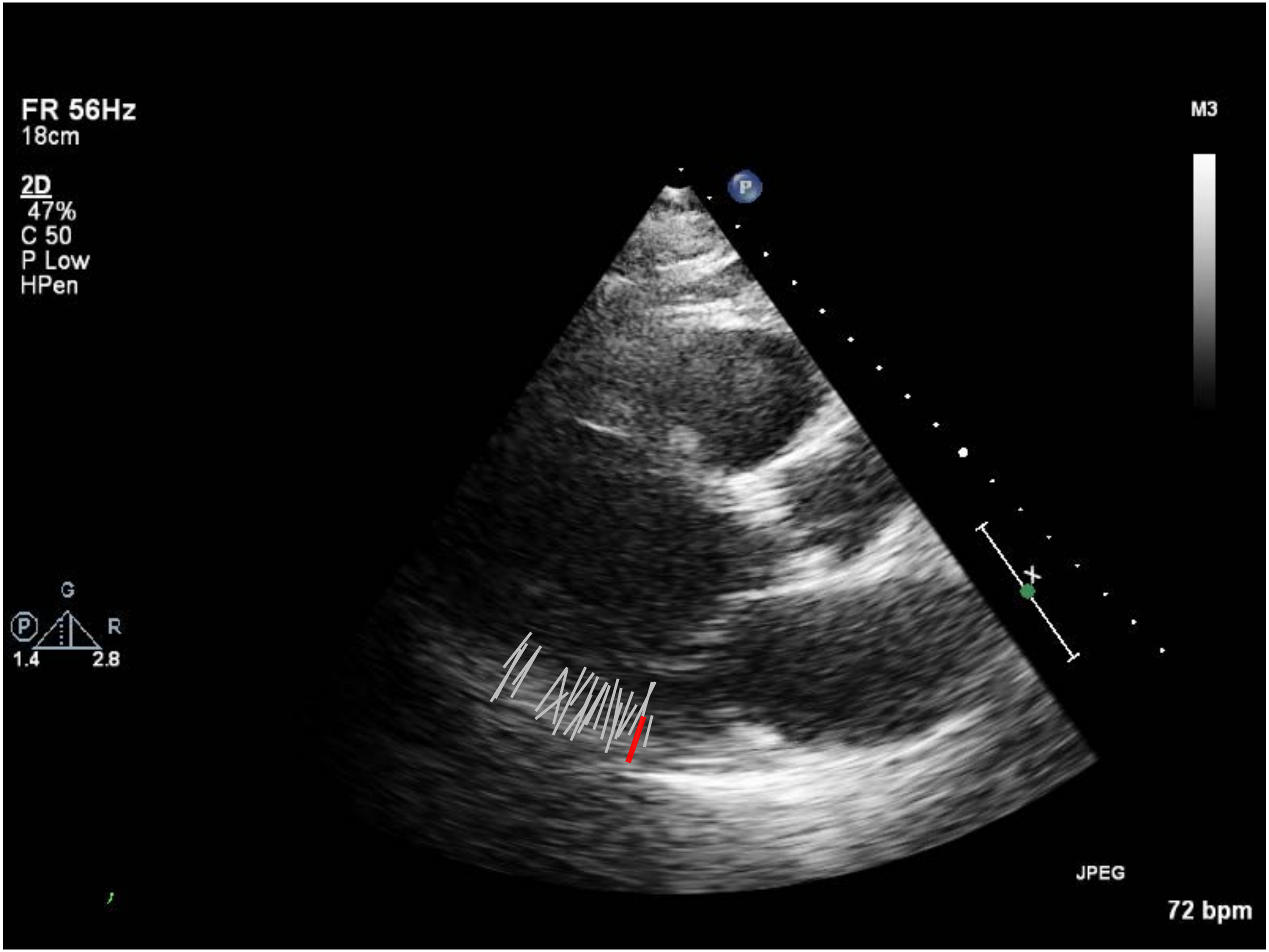

Pt 19 Diastole

Absolute difference between AI and expert consensus: 0.44 mm

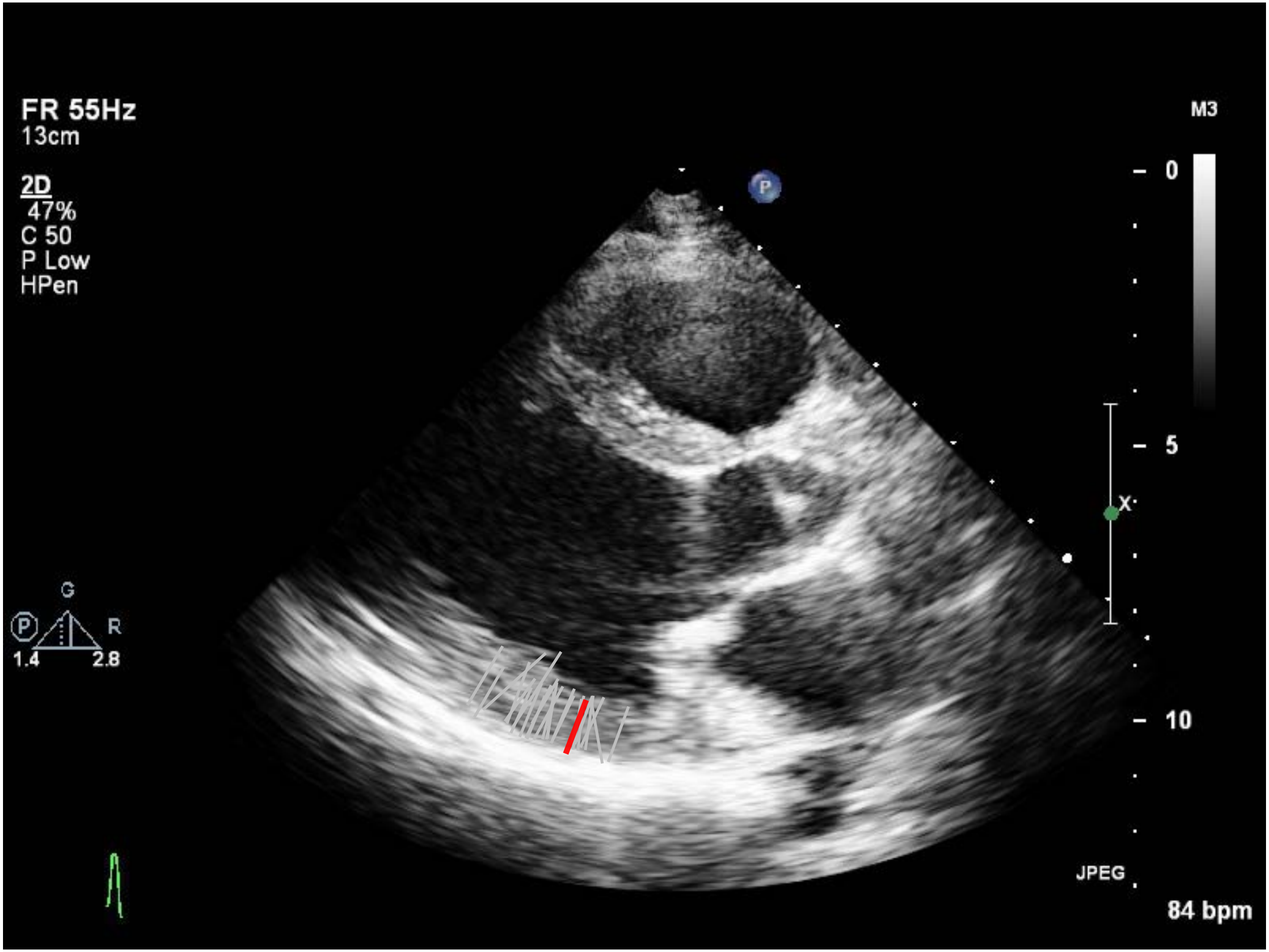

Pt 46 Diastole

Absolute difference between AI and expert consensus: 0.47 mm

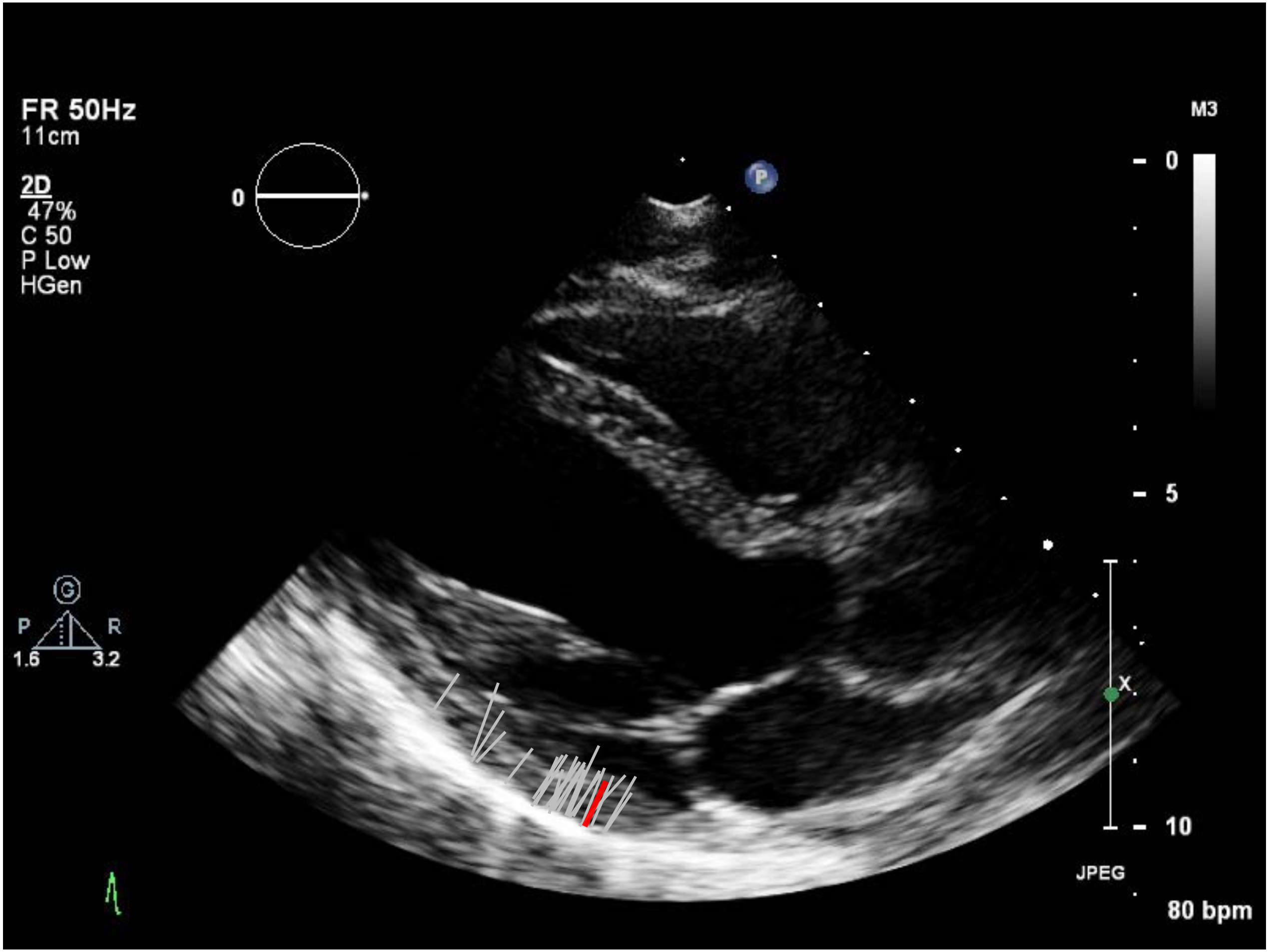

Pt 17 Diastole

Absolute difference between AI and expert consensus: 0.52 mm

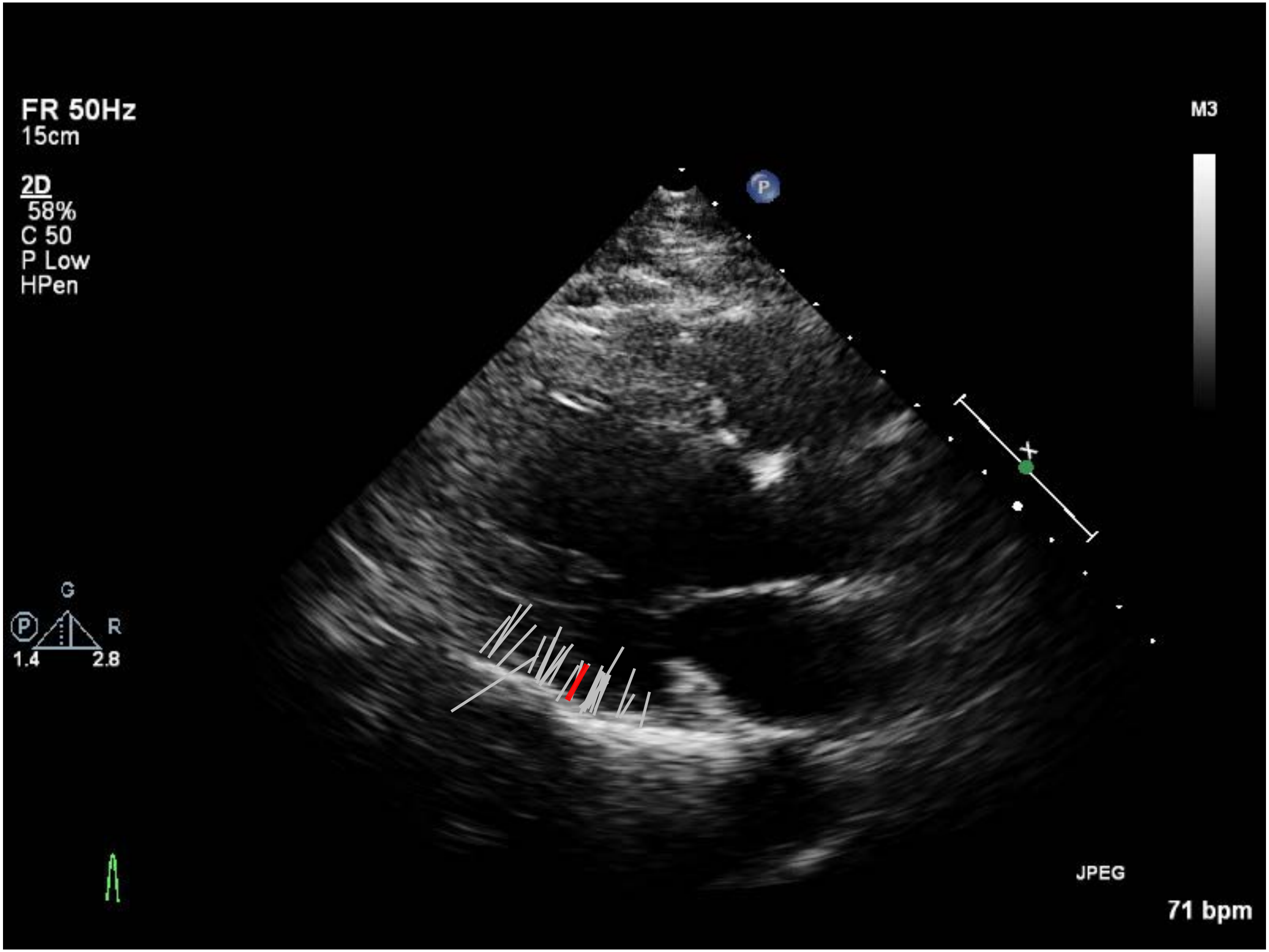

Pt 77 Diastole

Absolute difference between AI and expert consensus: 0.54 mm

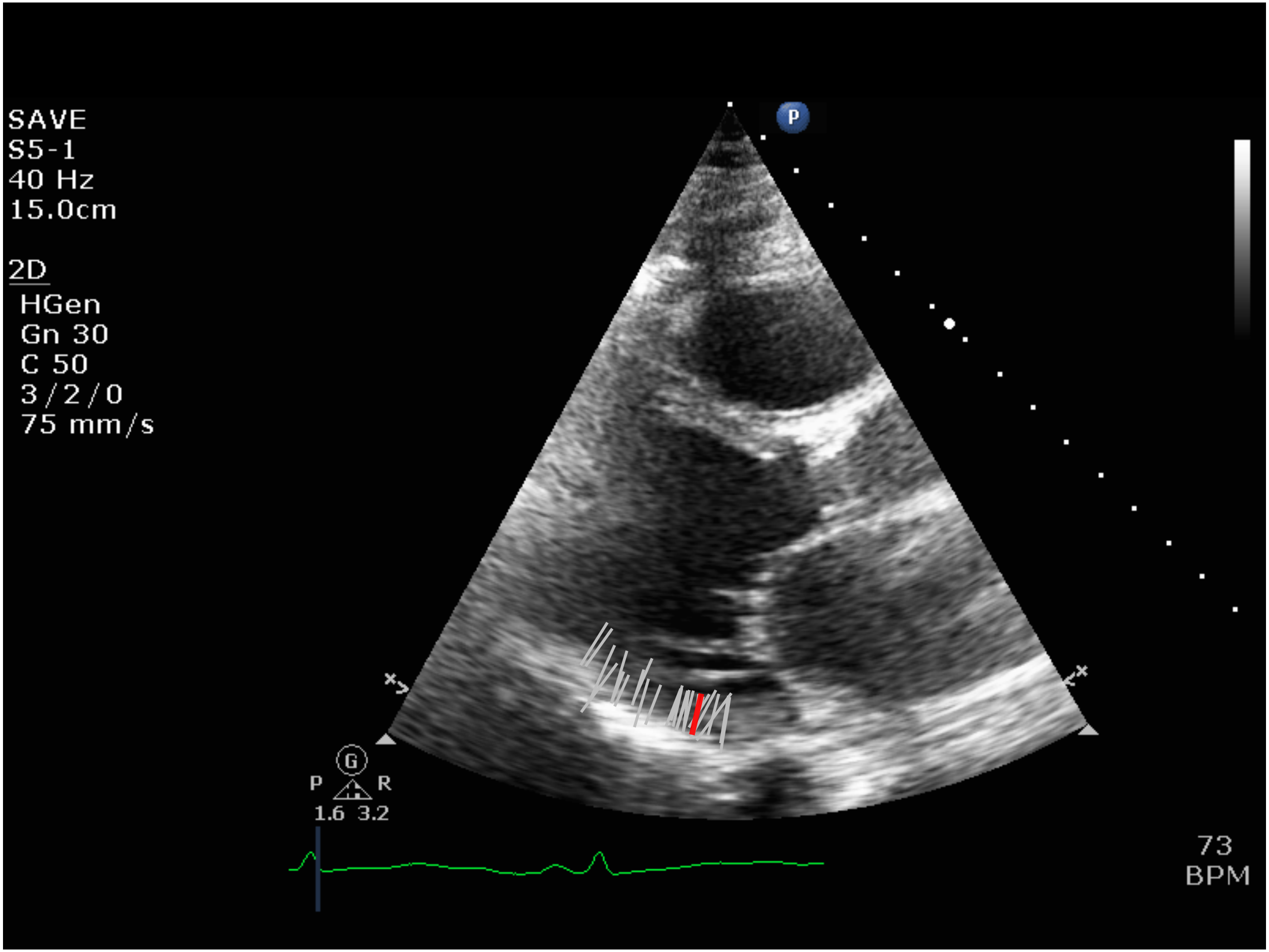

Pt 14 Diastole

Absolute difference between AI and expert consensus: 0.54 mm

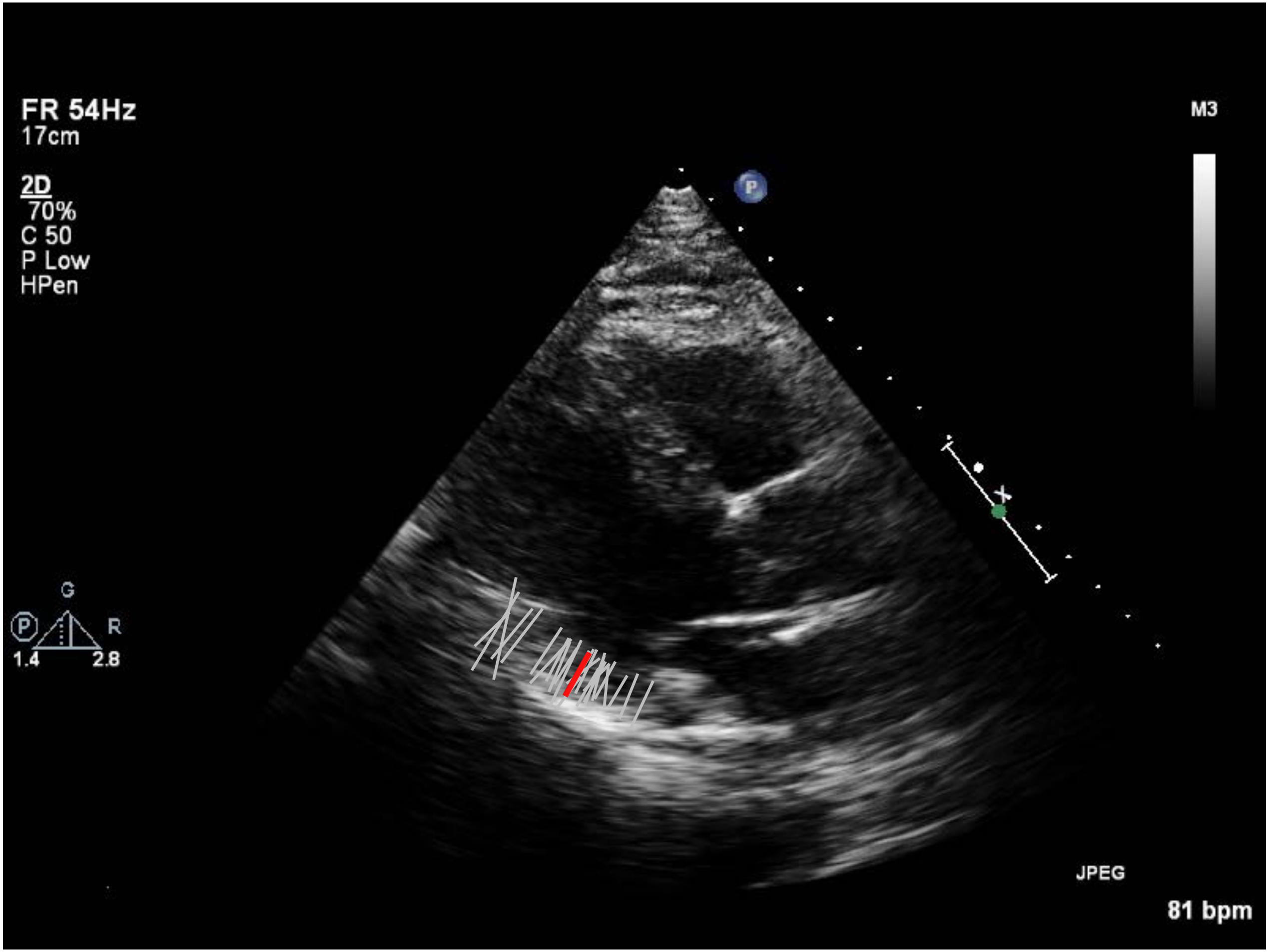

Pt 90 Diastole

Absolute difference between AI and expert consensus: 0.55 mm

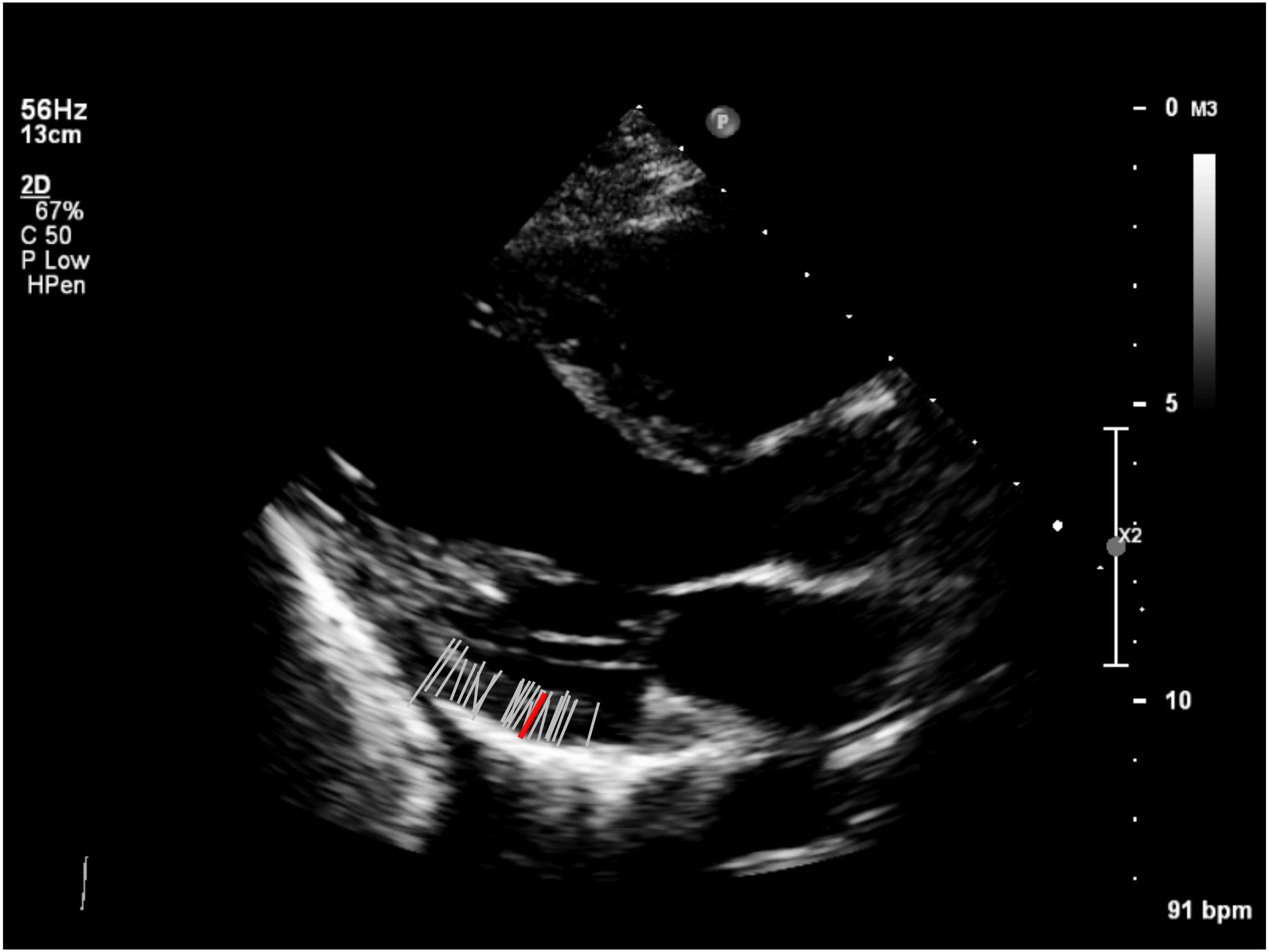

Pt 54 Diastole

Absolute difference between AI and expert consensus: 0.55 mm

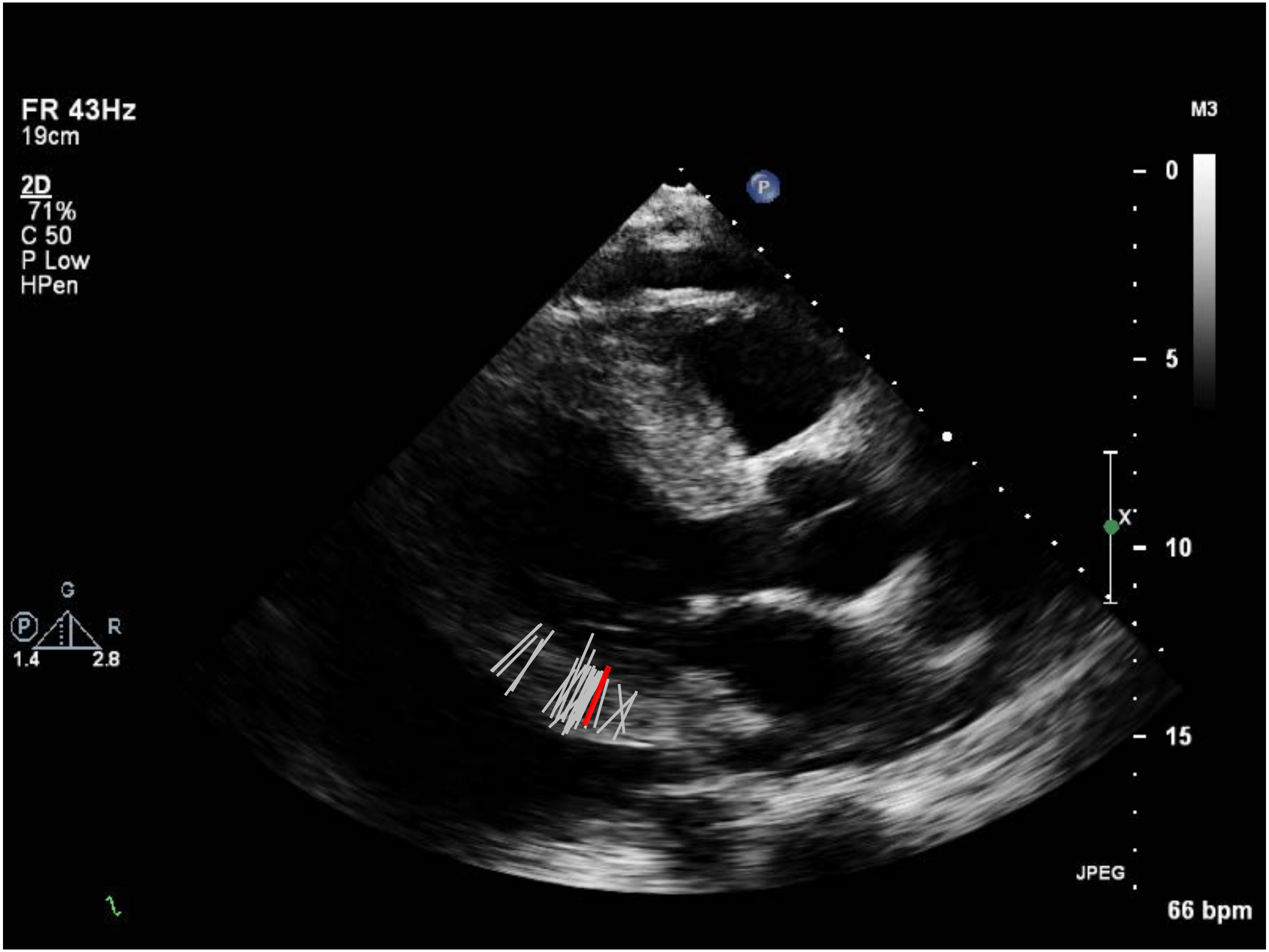

Pt 100 Diastole

Absolute difference between AI and expert consensus: 0.55 mm

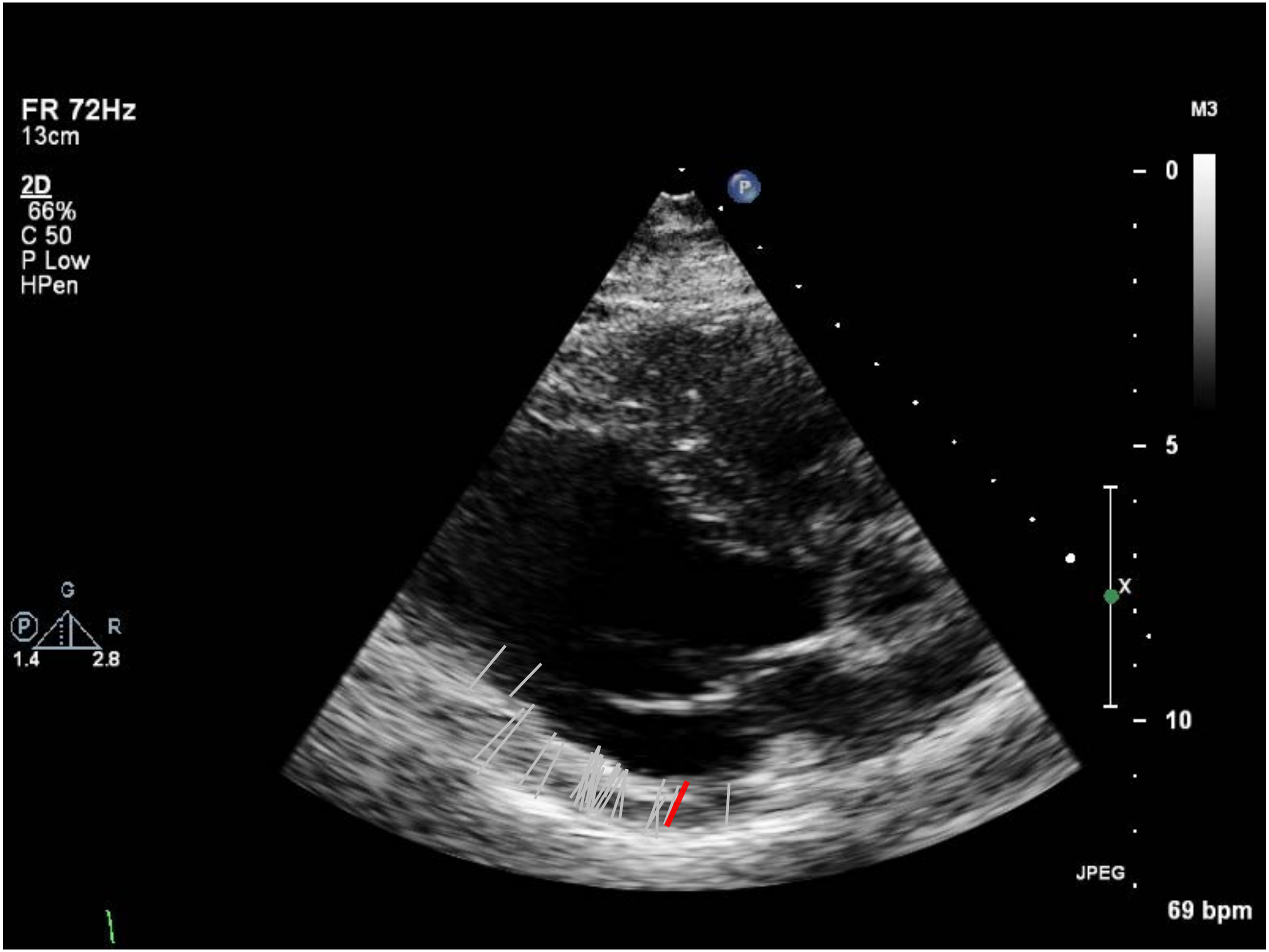

Pt 67 Diastole

Absolute difference between AI and expert consensus: 0.56 mm

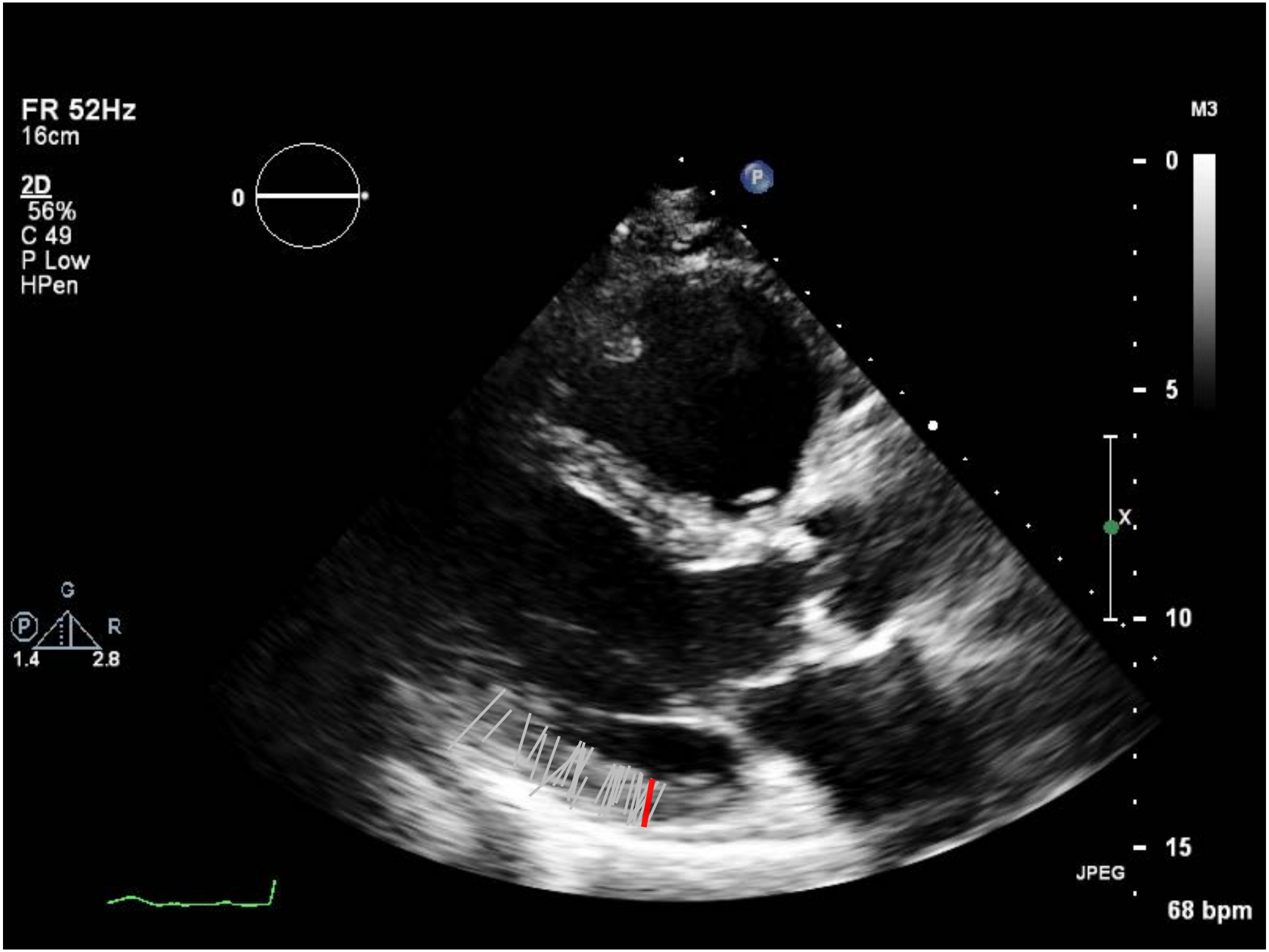

Pt 37 Diastole

Absolute difference between AI and expert consensus: 0.57 mm

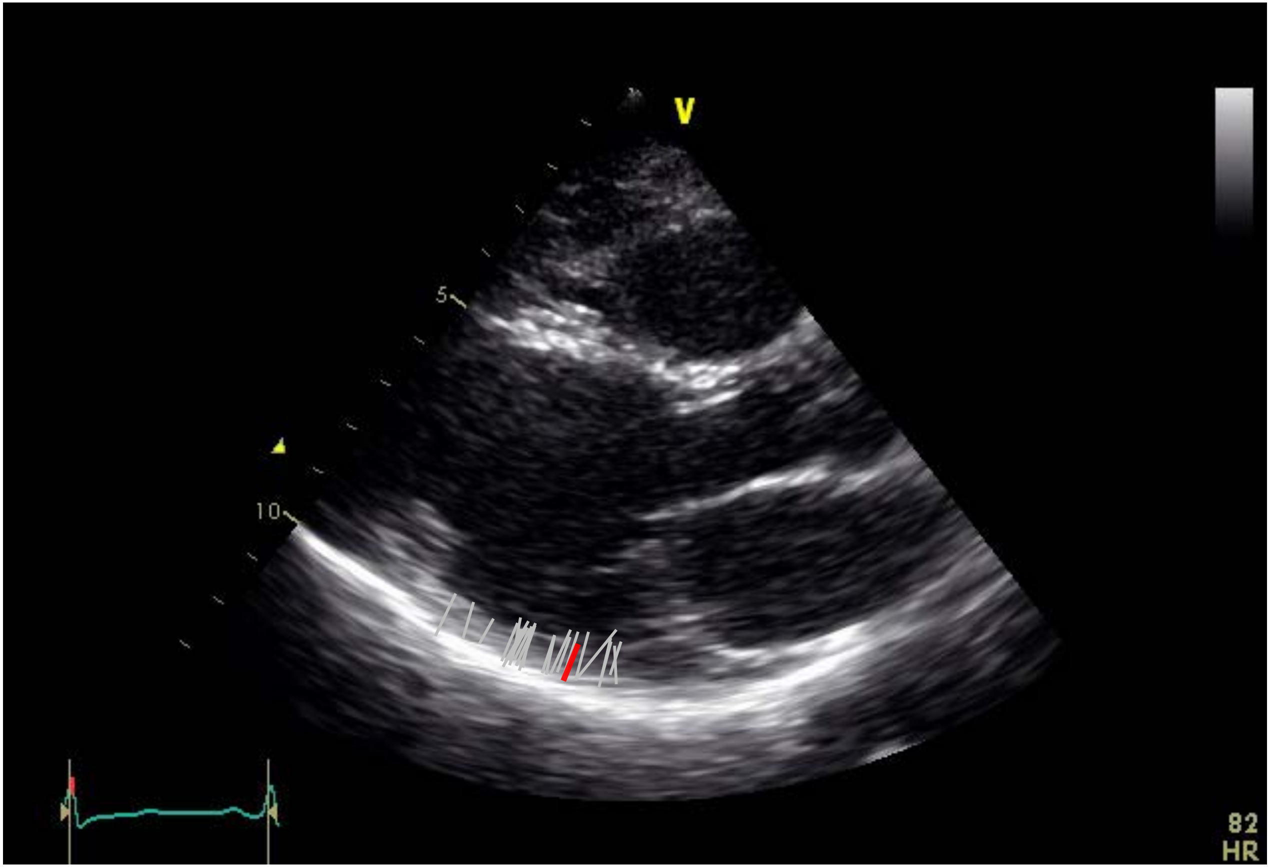

Pt 35 Diastole

Absolute difference between AI and expert consensus: 0.58 mm

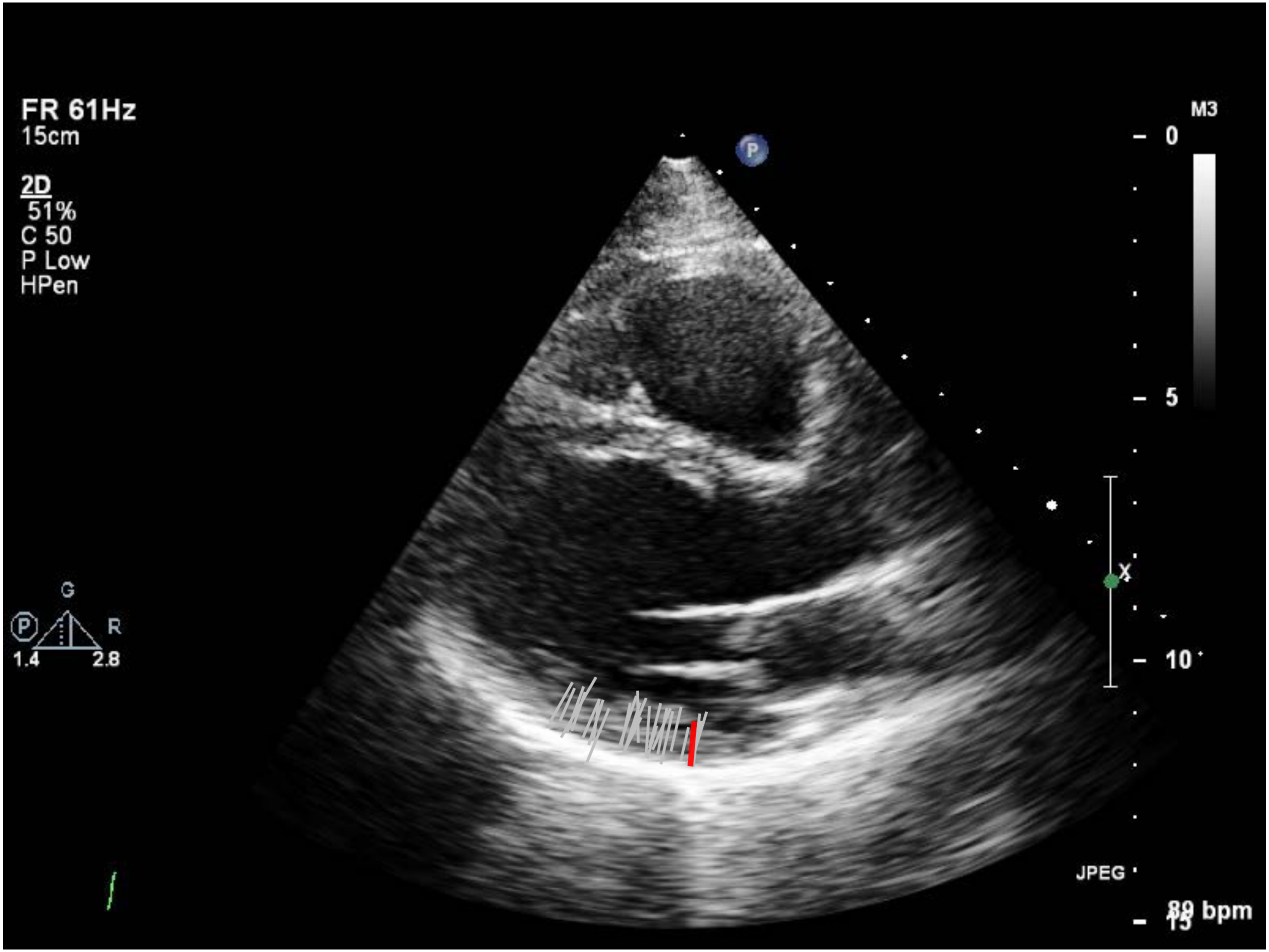

Pt 58 Diastole

Absolute difference between AI and expert consensus: 0.62 mm

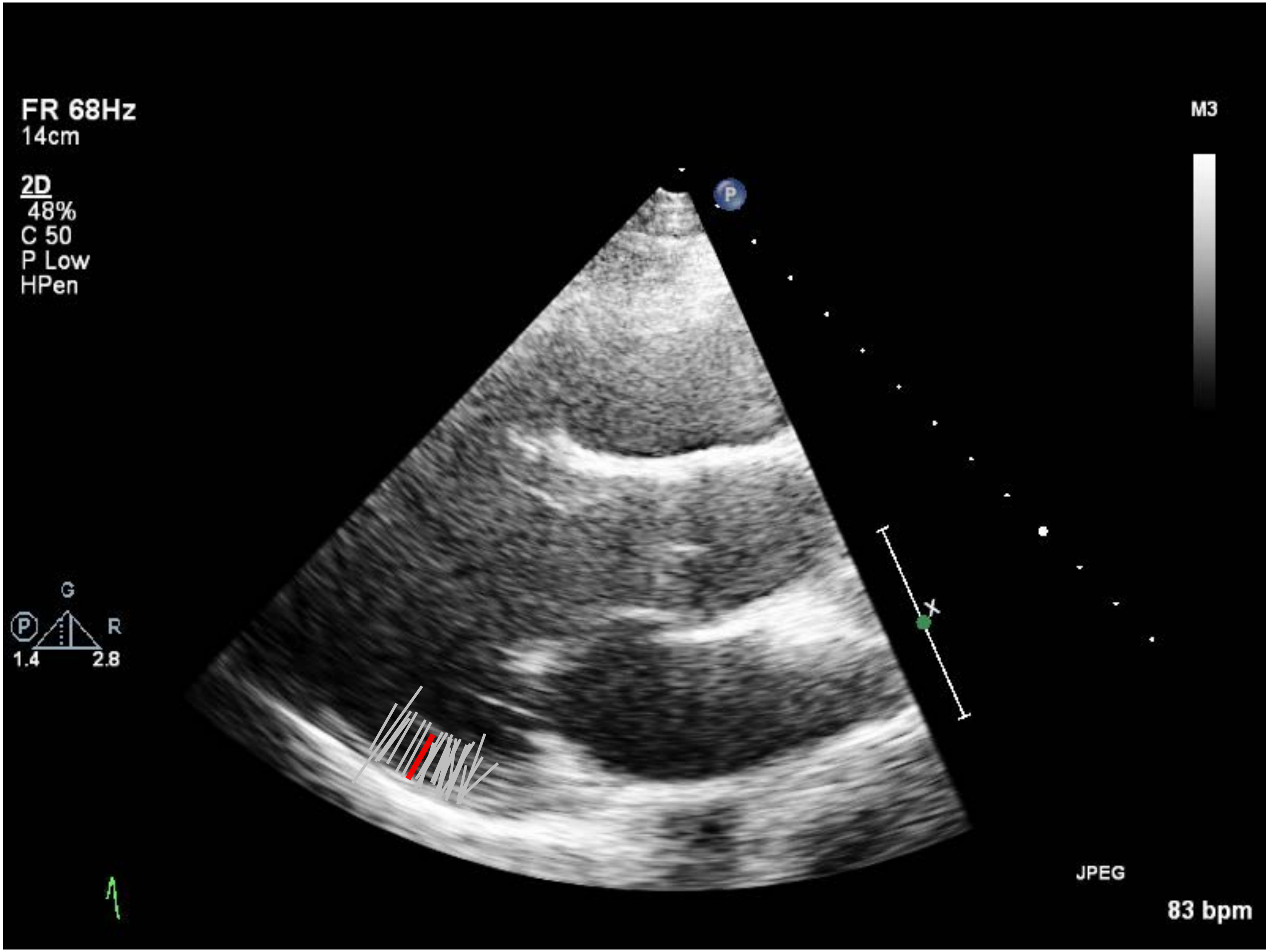

Pt 86 Diastole

Absolute difference between AI and expert consensus: 0.64 mm

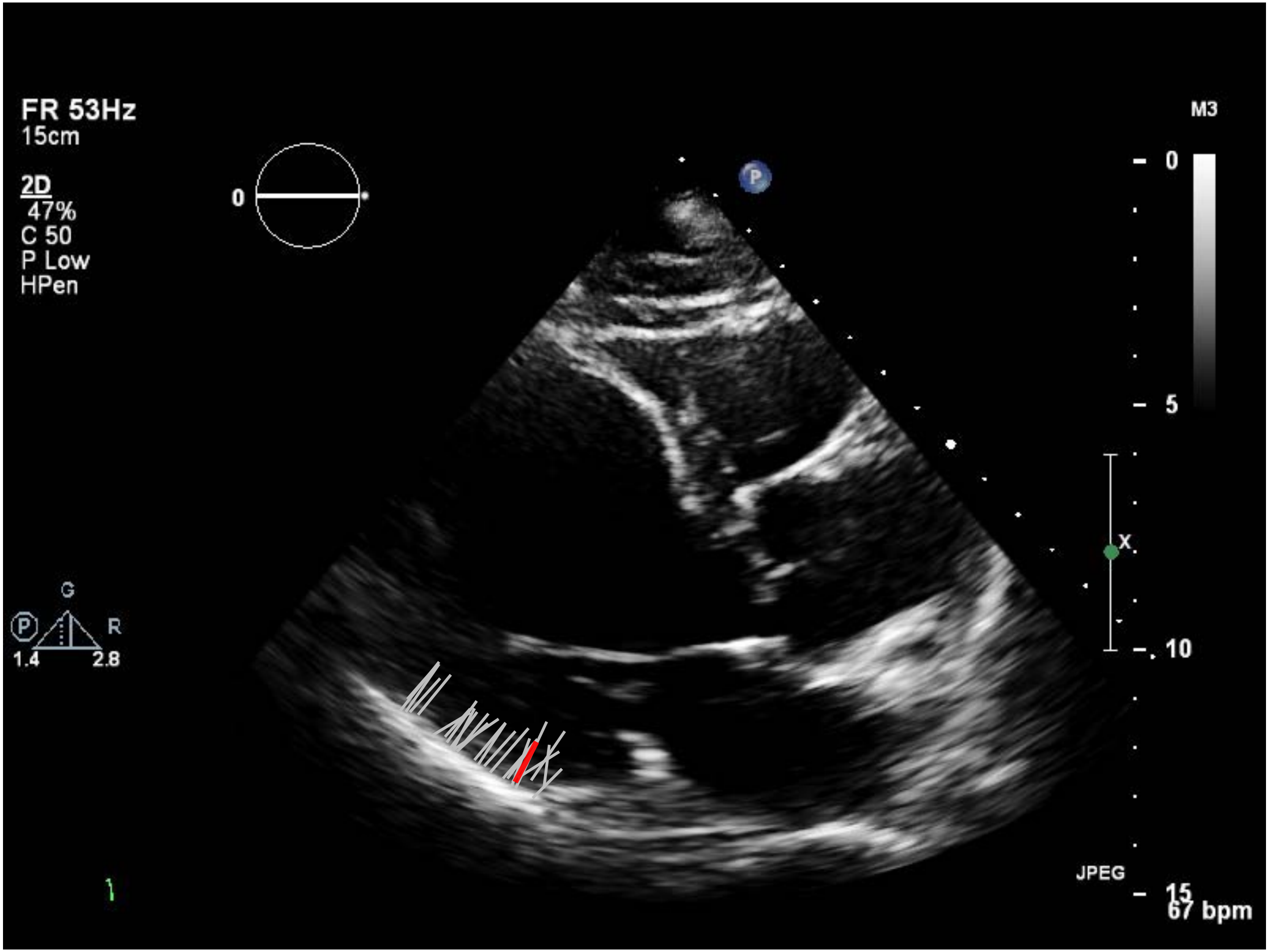

Pt 43 Diastole

Absolute difference between AI and expert consensus: 0.65 mm

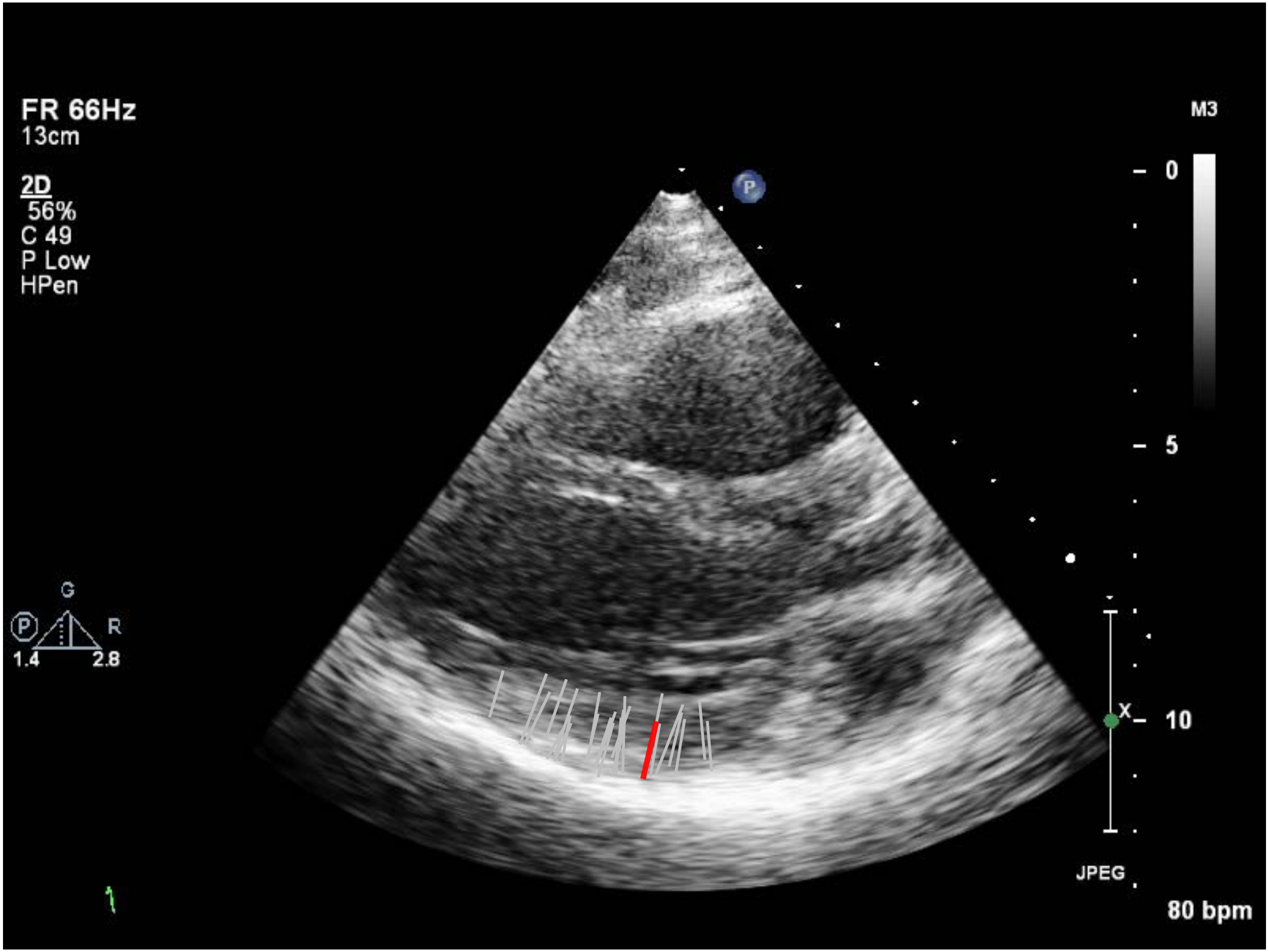

Pt 91 Diastole

Absolute difference between AI and expert consensus: 0.67 mm

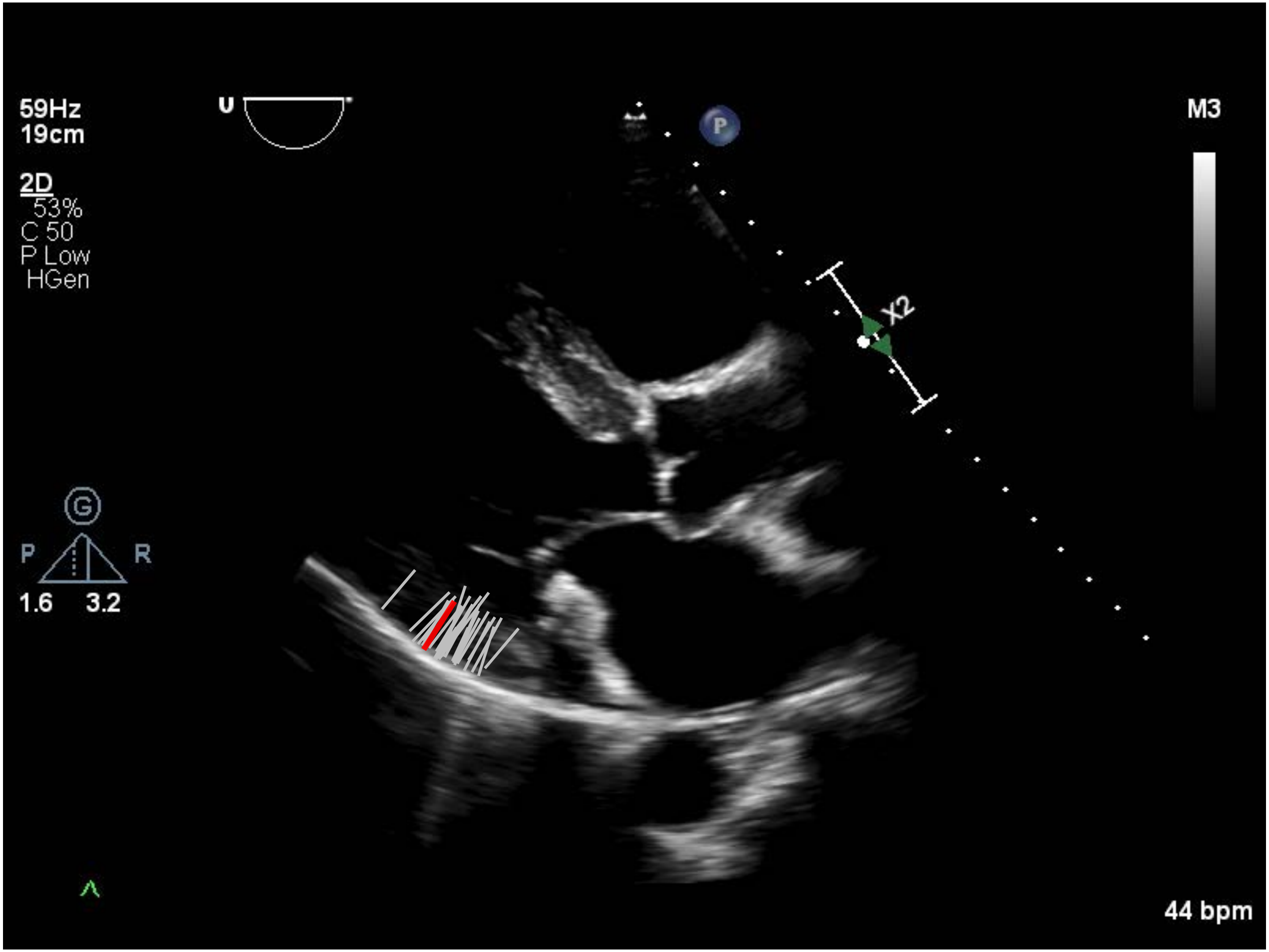

Pt 13 Diastole

Absolute difference between AI and expert consensus: 0.68 mm

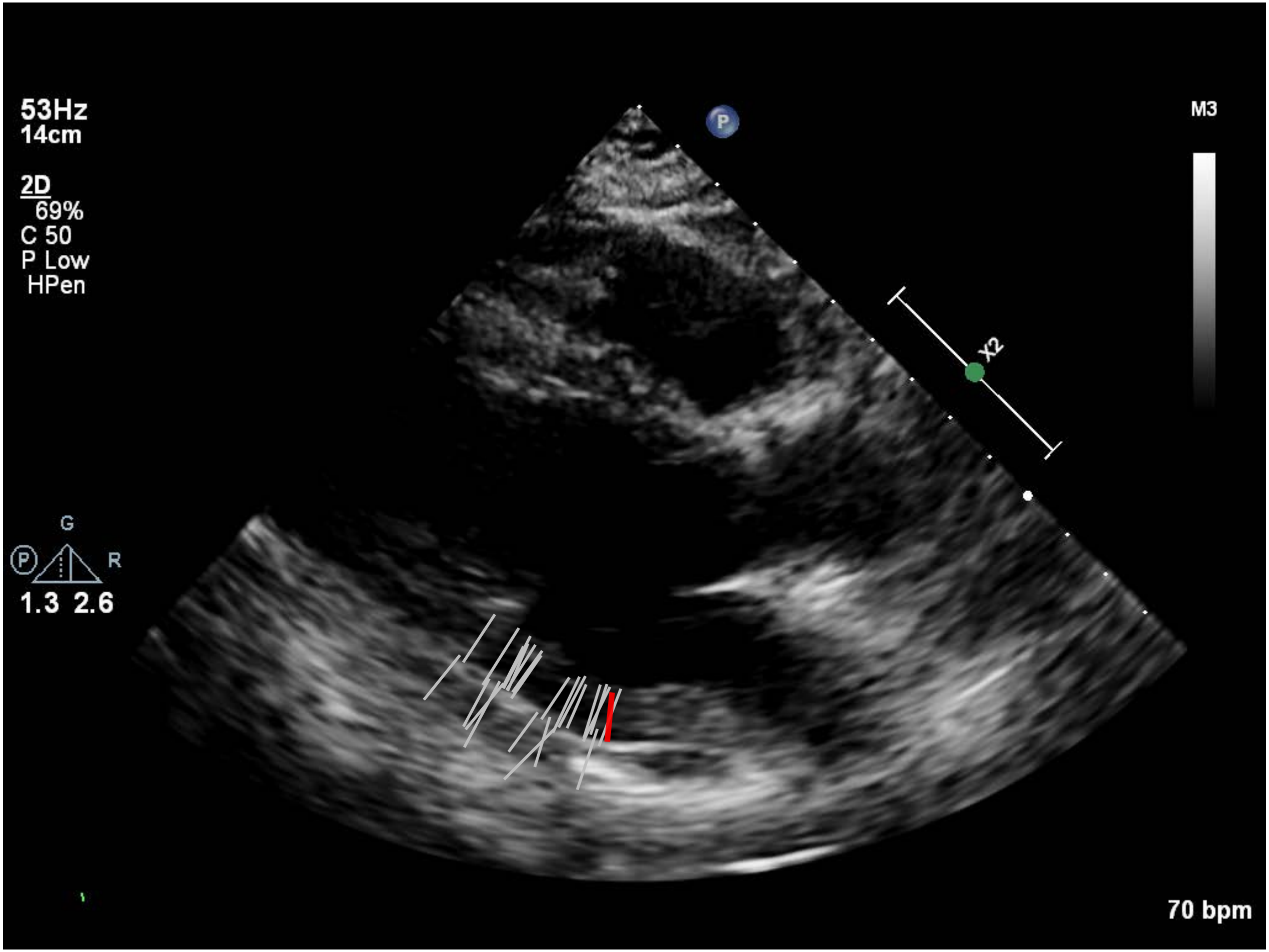

Pt 83 Diastole

Absolute difference between AI and expert consensus: 0.72 mm

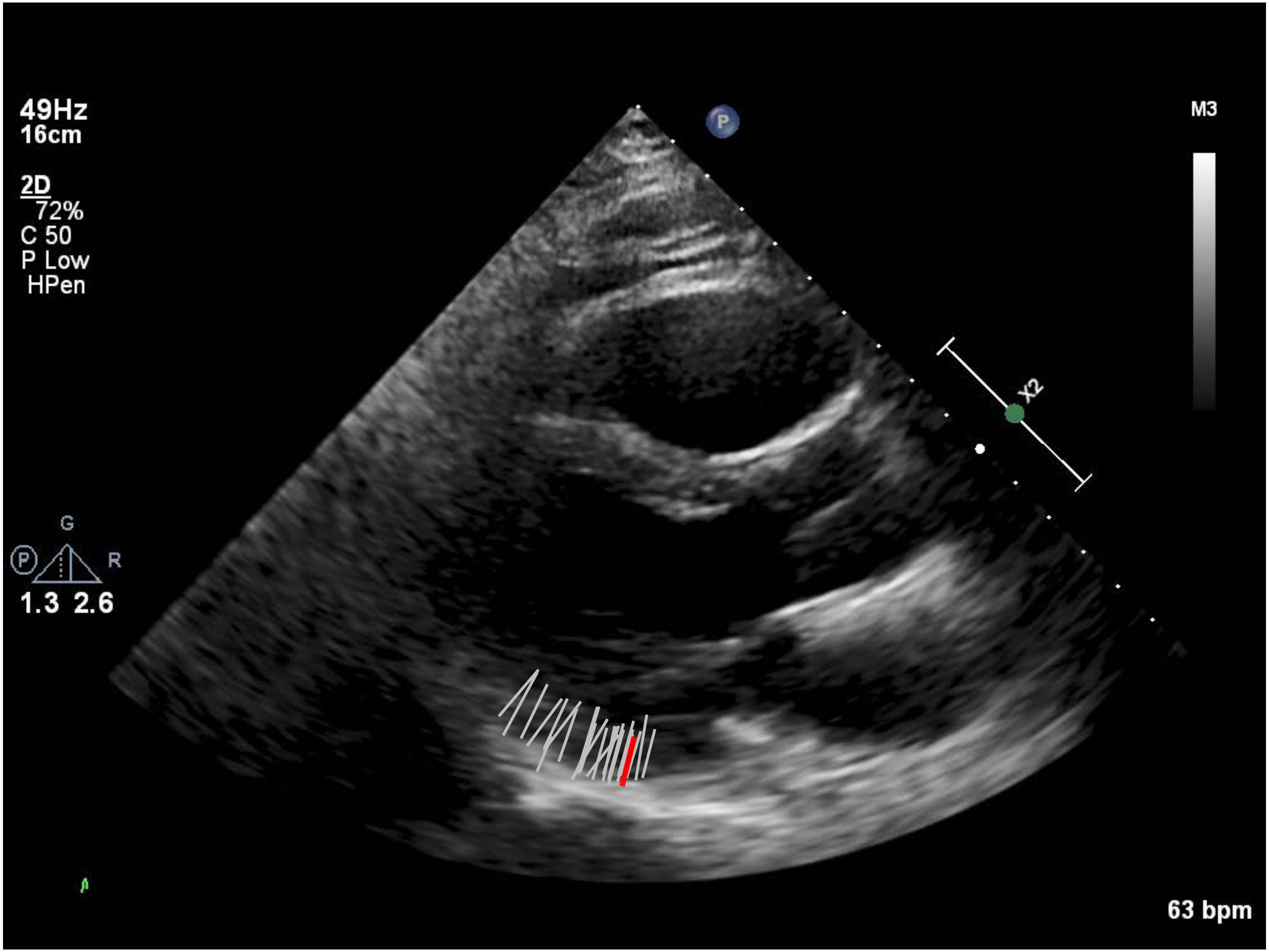

Pt 24 Diastole

Absolute difference between AI and expert consensus: 0.74 mm

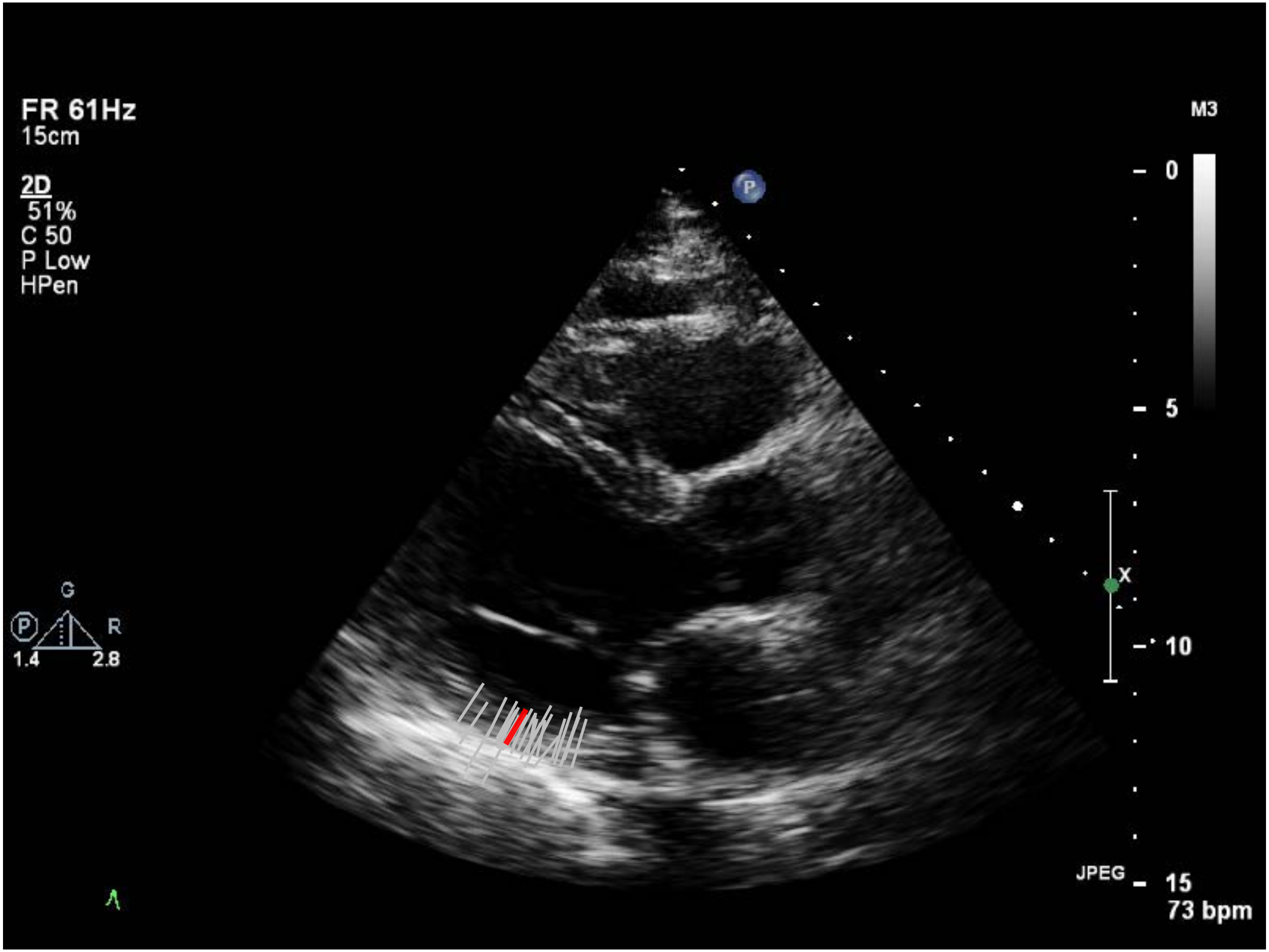

Pt 93 Diastole

Absolute difference between AI and expert consensus: 0.74 mm

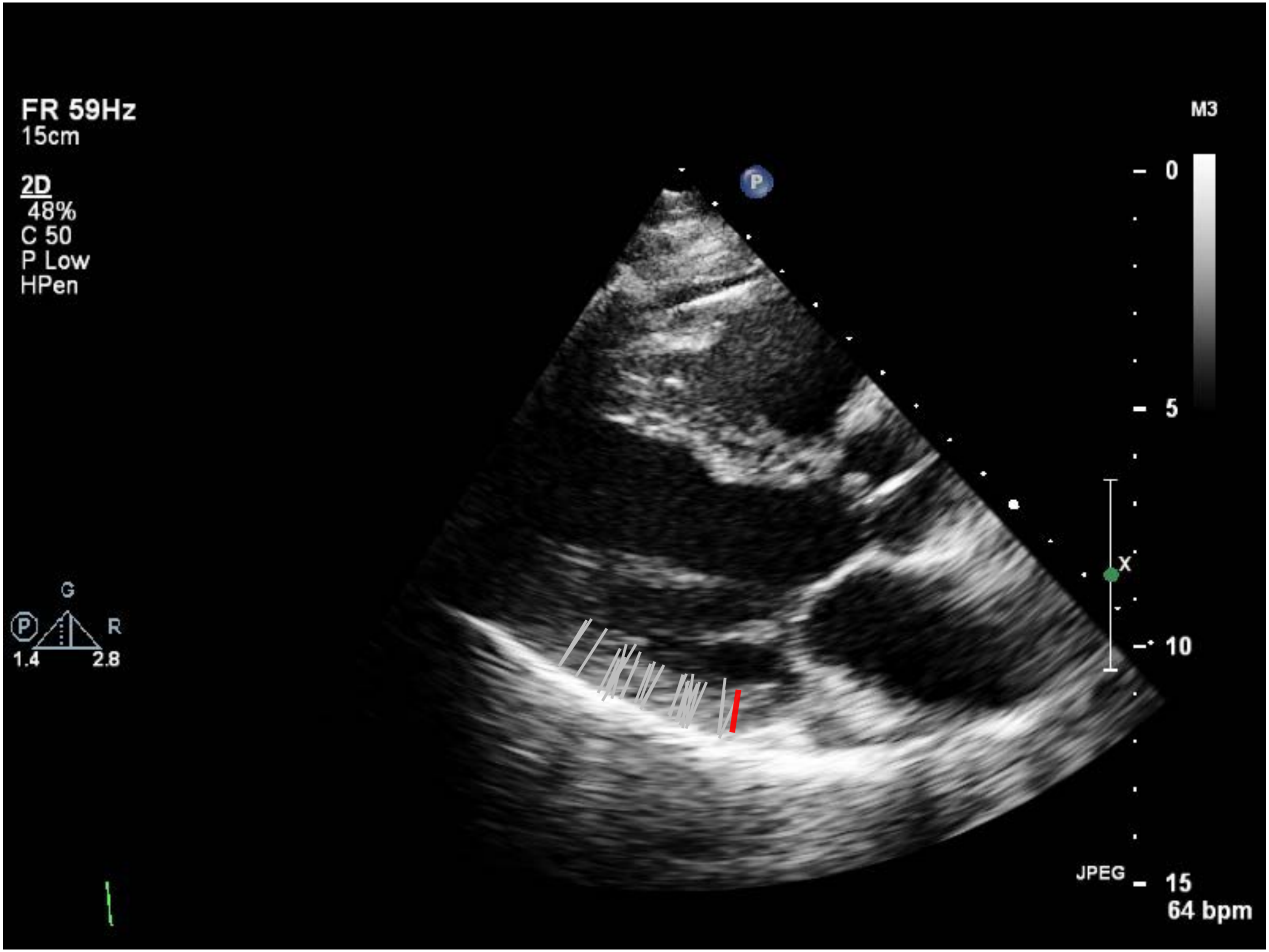

Pt 27 Diastole

Absolute difference between AI and expert consensus: 0.75 mm

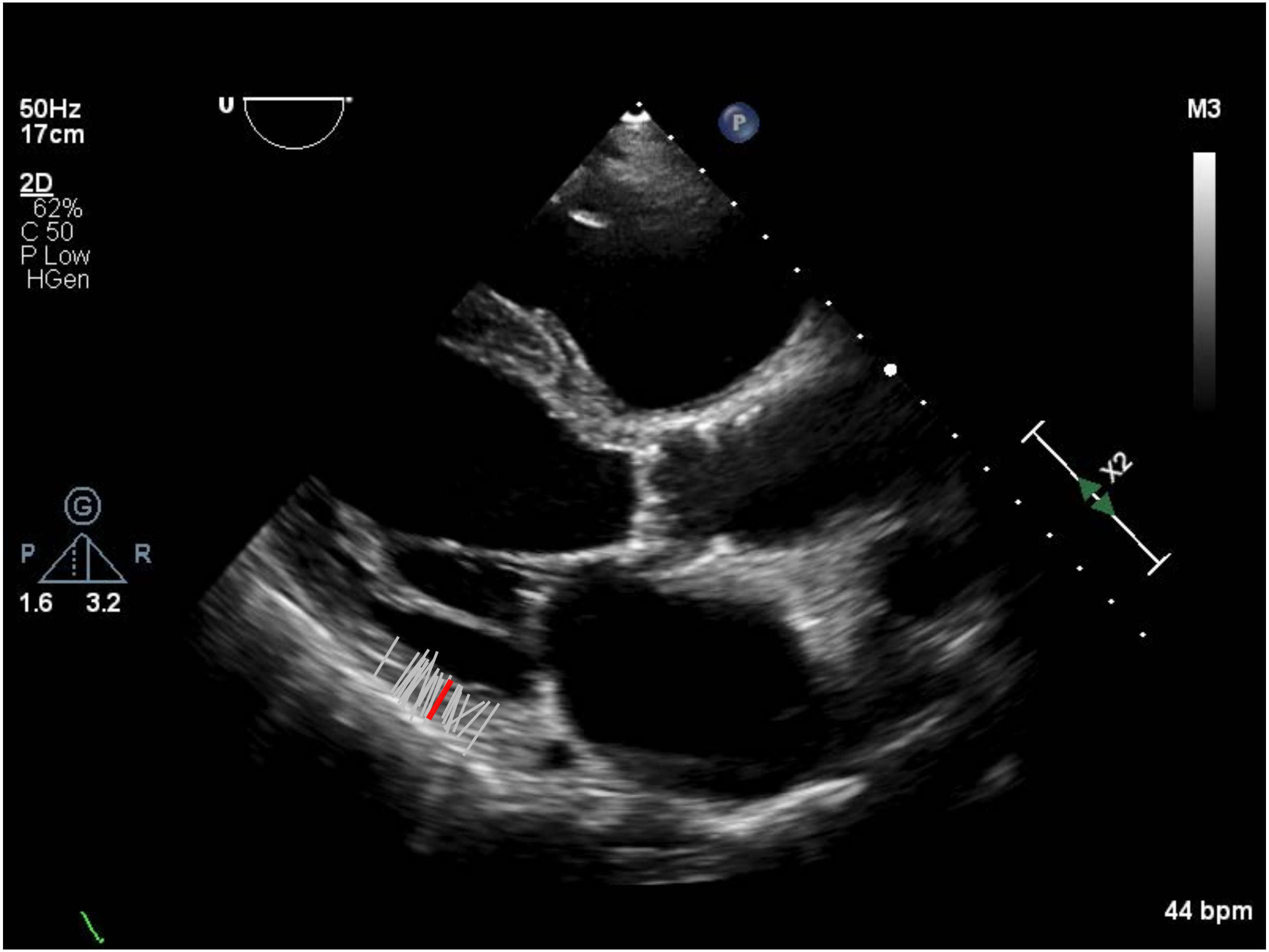

Pt 52 Diastole

Absolute difference between AI and expert consensus: 0.75 mm

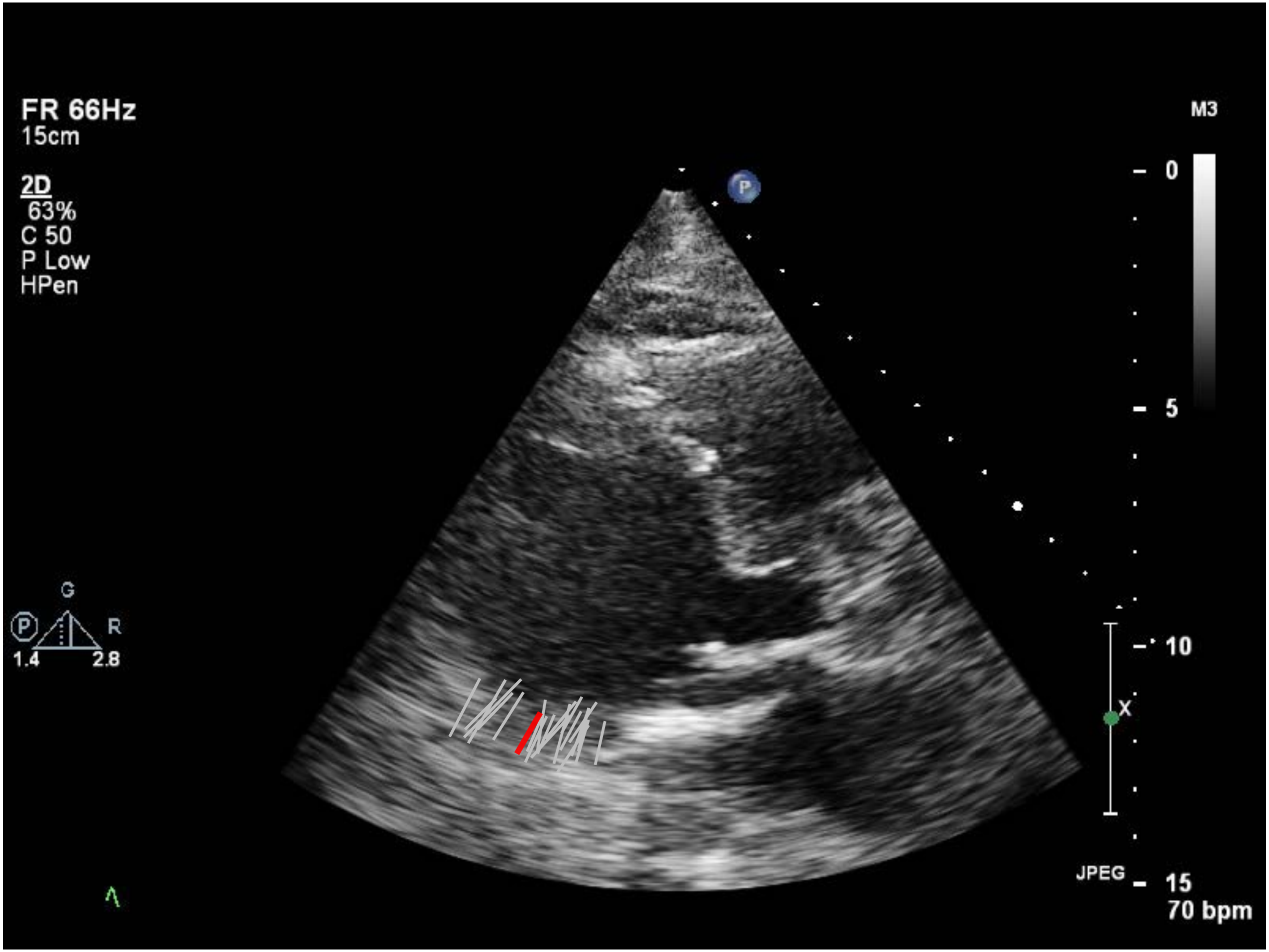

Pt 62 Diastole

Absolute difference between AI and expert consensus: 0.78 mm

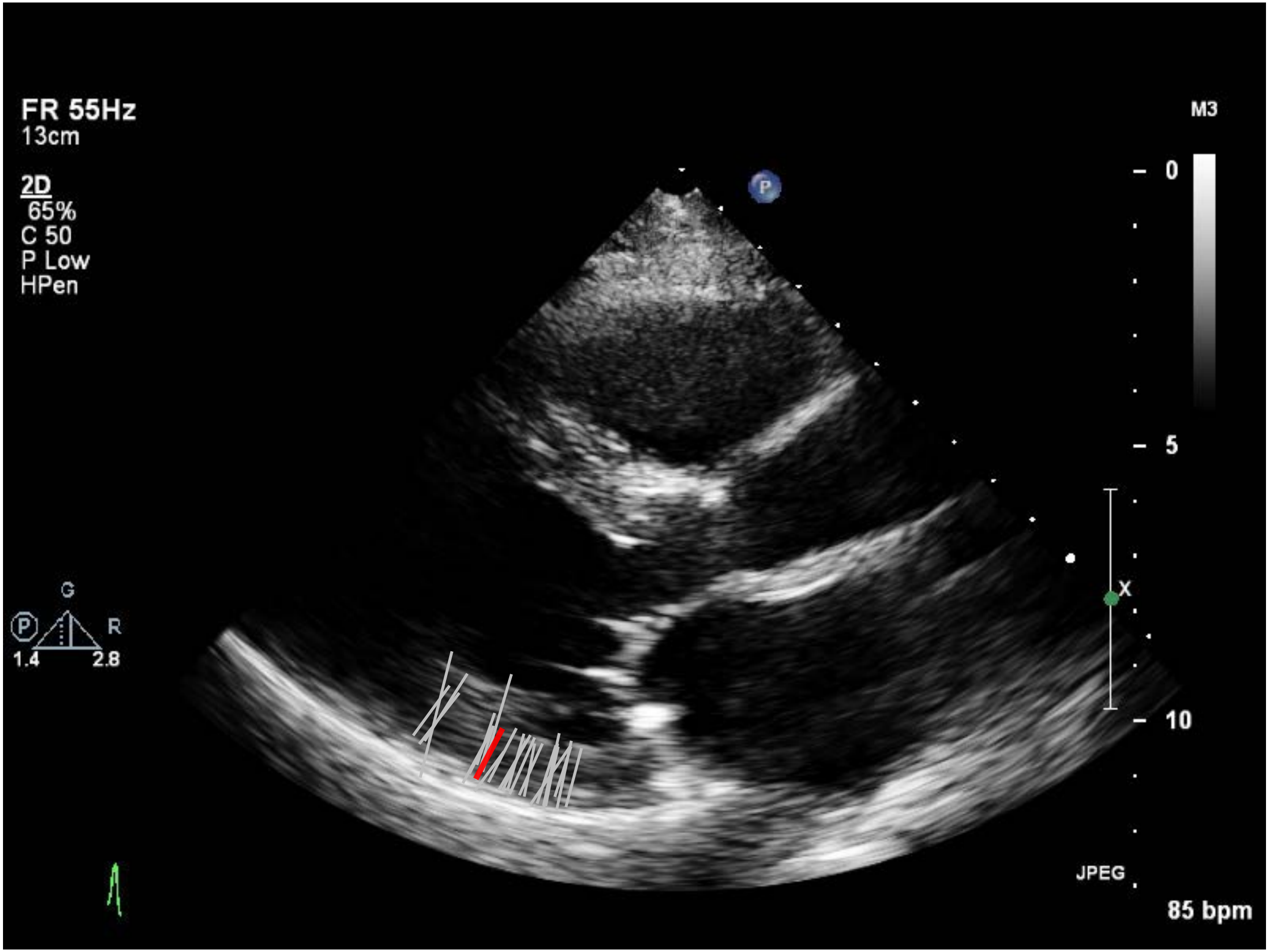

Pt 73 Diastole

Absolute difference between AI and expert consensus: 0.81 mm

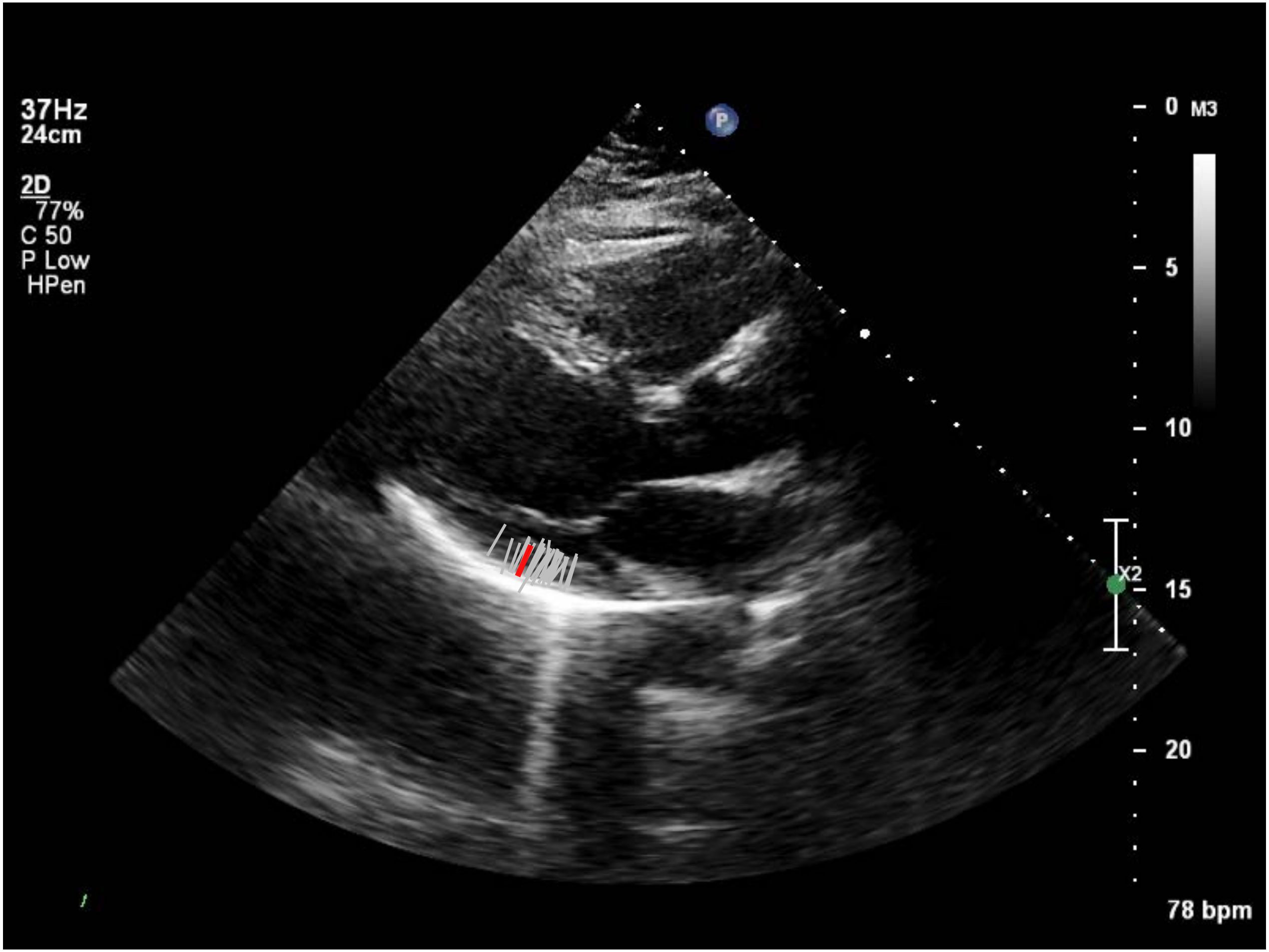

Pt 23 Diastole

Absolute difference between AI and expert consensus: 0.82 mm

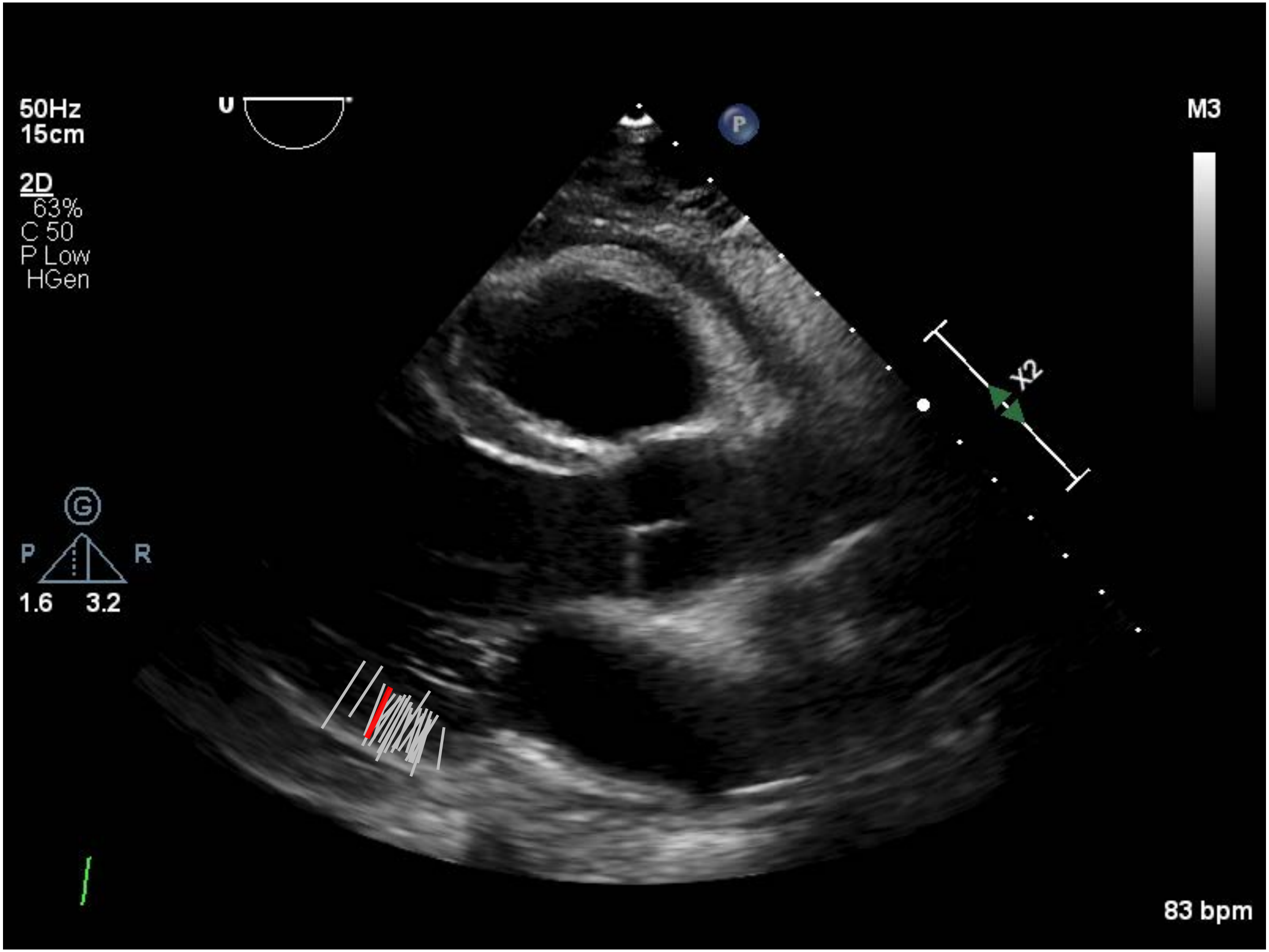

Pt 4 Diastole

Absolute difference between AI and expert consensus: 0.86 mm

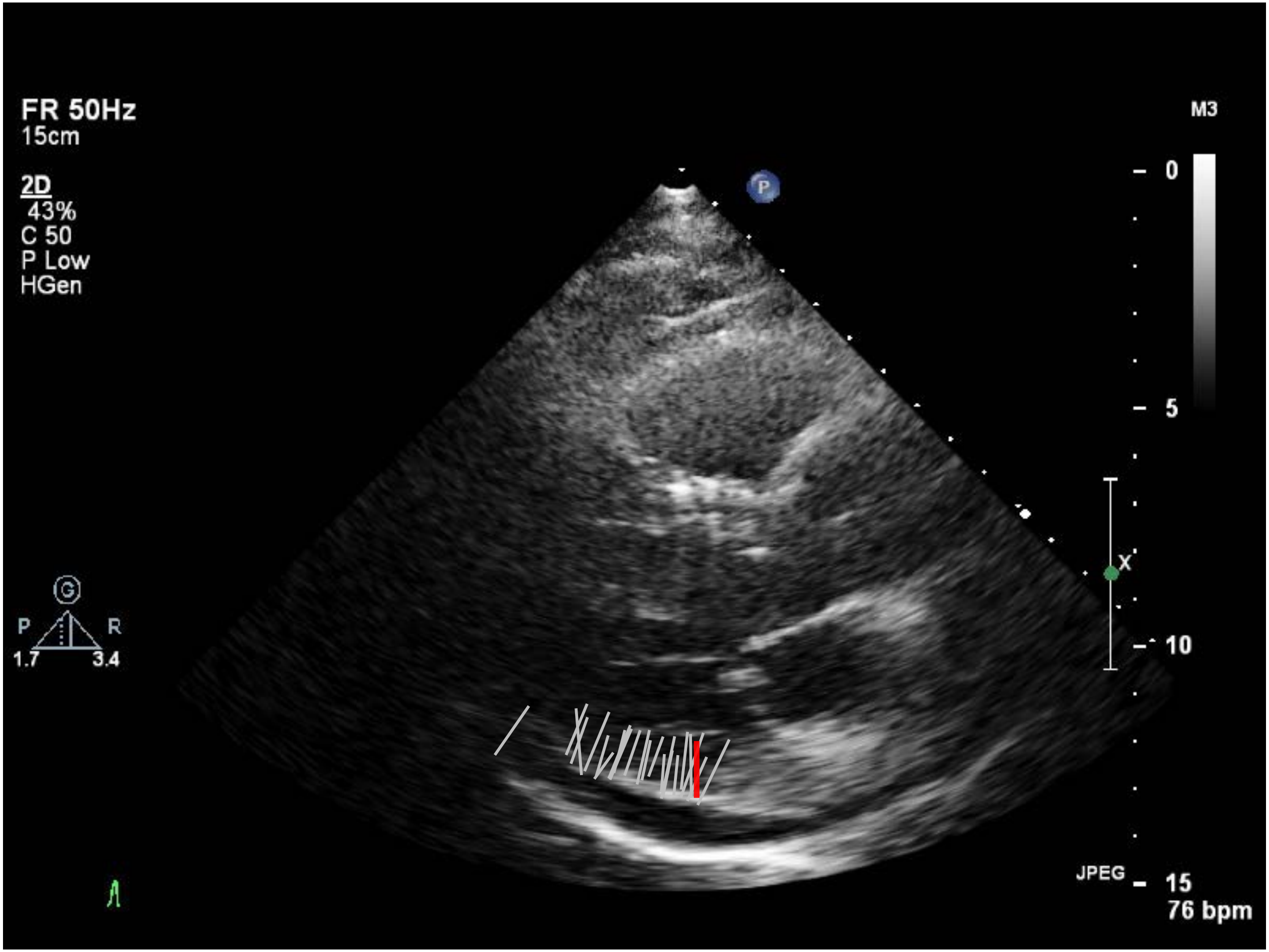

Pt 1 Diastole

Absolute difference between AI and expert consensus: 0.87 mm

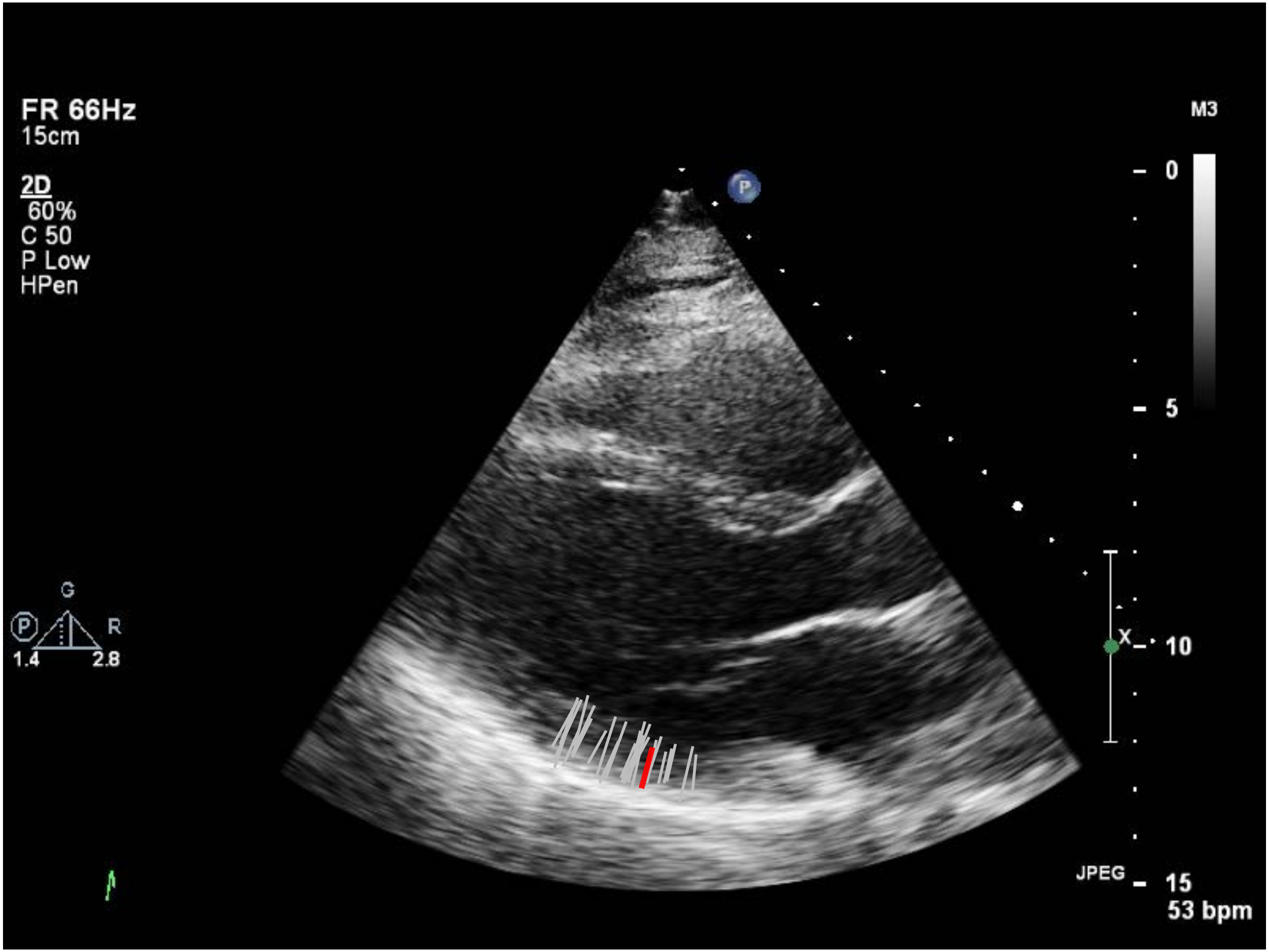

Pt 51 Diastole

Absolute difference between AI and expert consensus: 0.91 mm

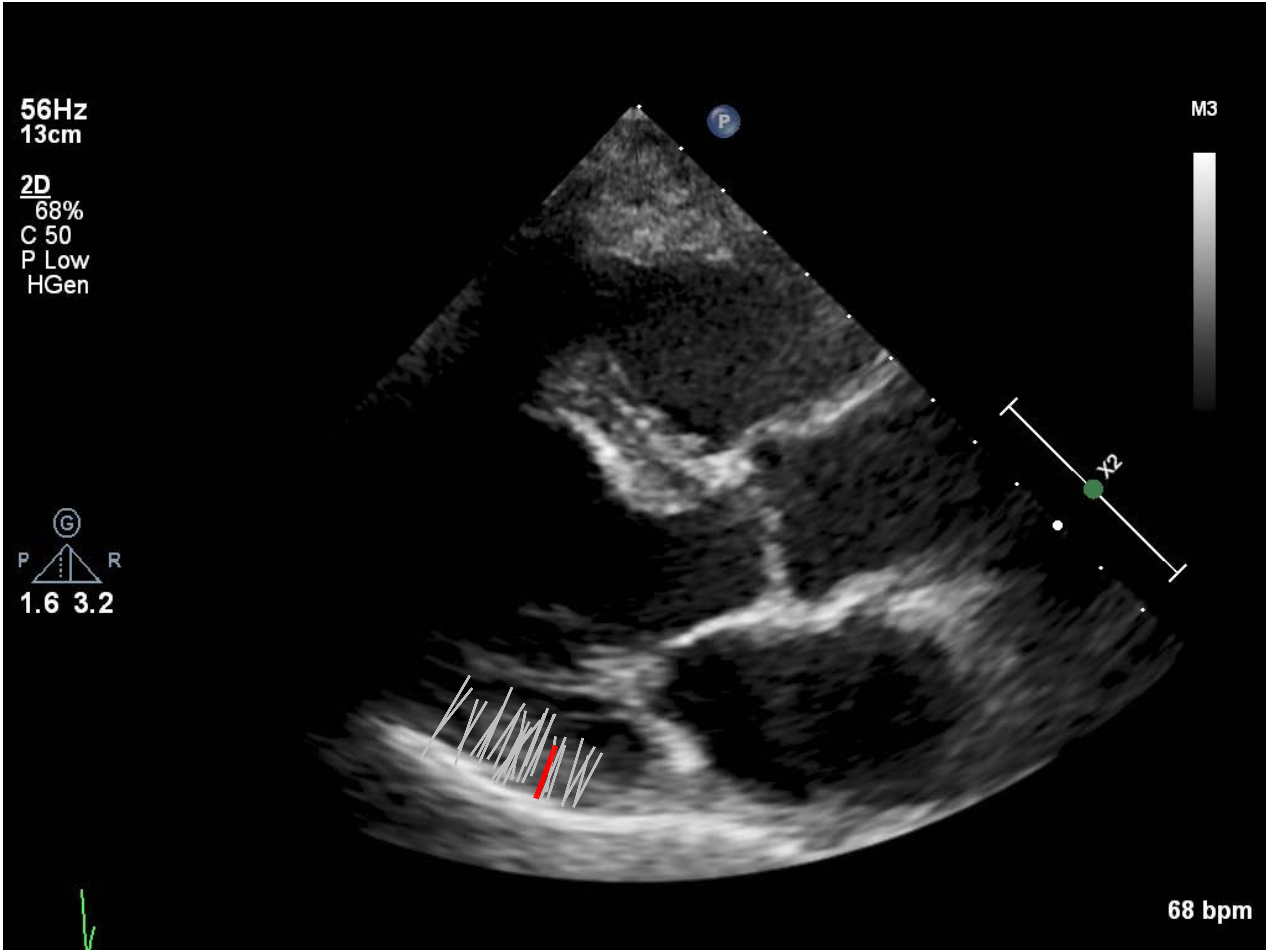

Pt 57 Diastole
Absolute difference between AI and expert consensus: 0.91 mm

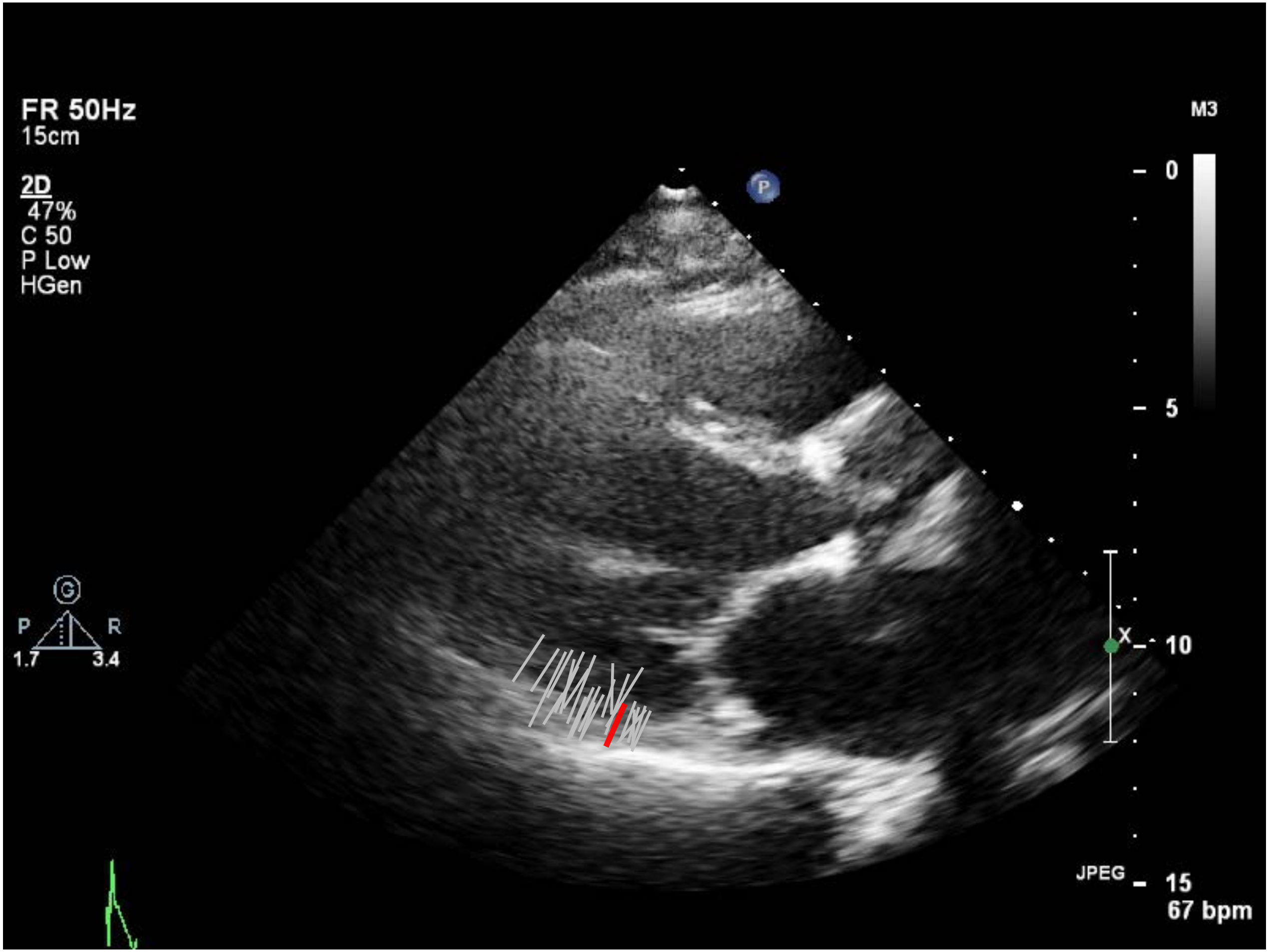

Pt 72 Diastole

Absolute difference between AI and expert consensus: 0.92 mm

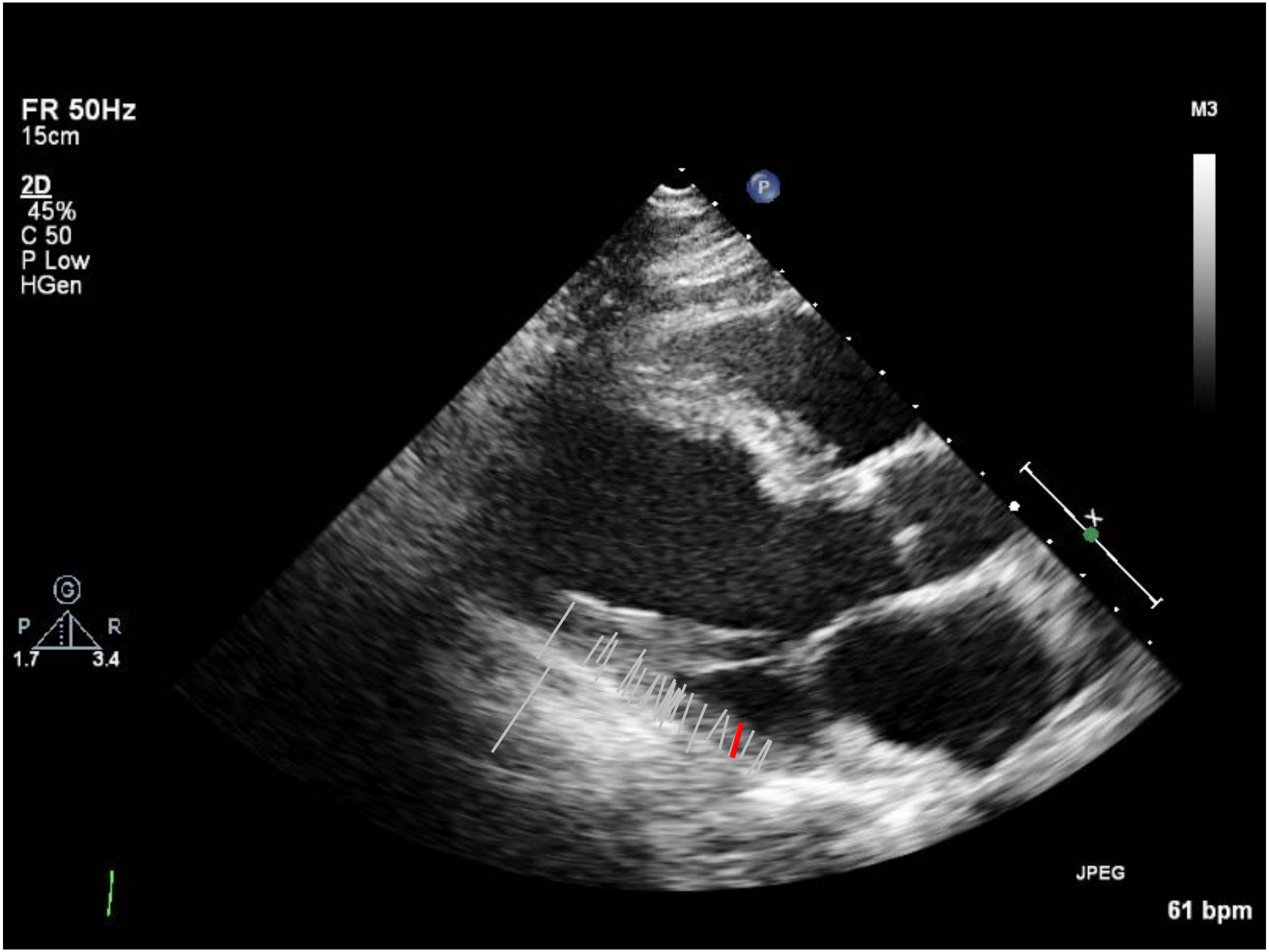

Pt 85 Diastole

Absolute difference between AI and expert consensus: 0.93 mm

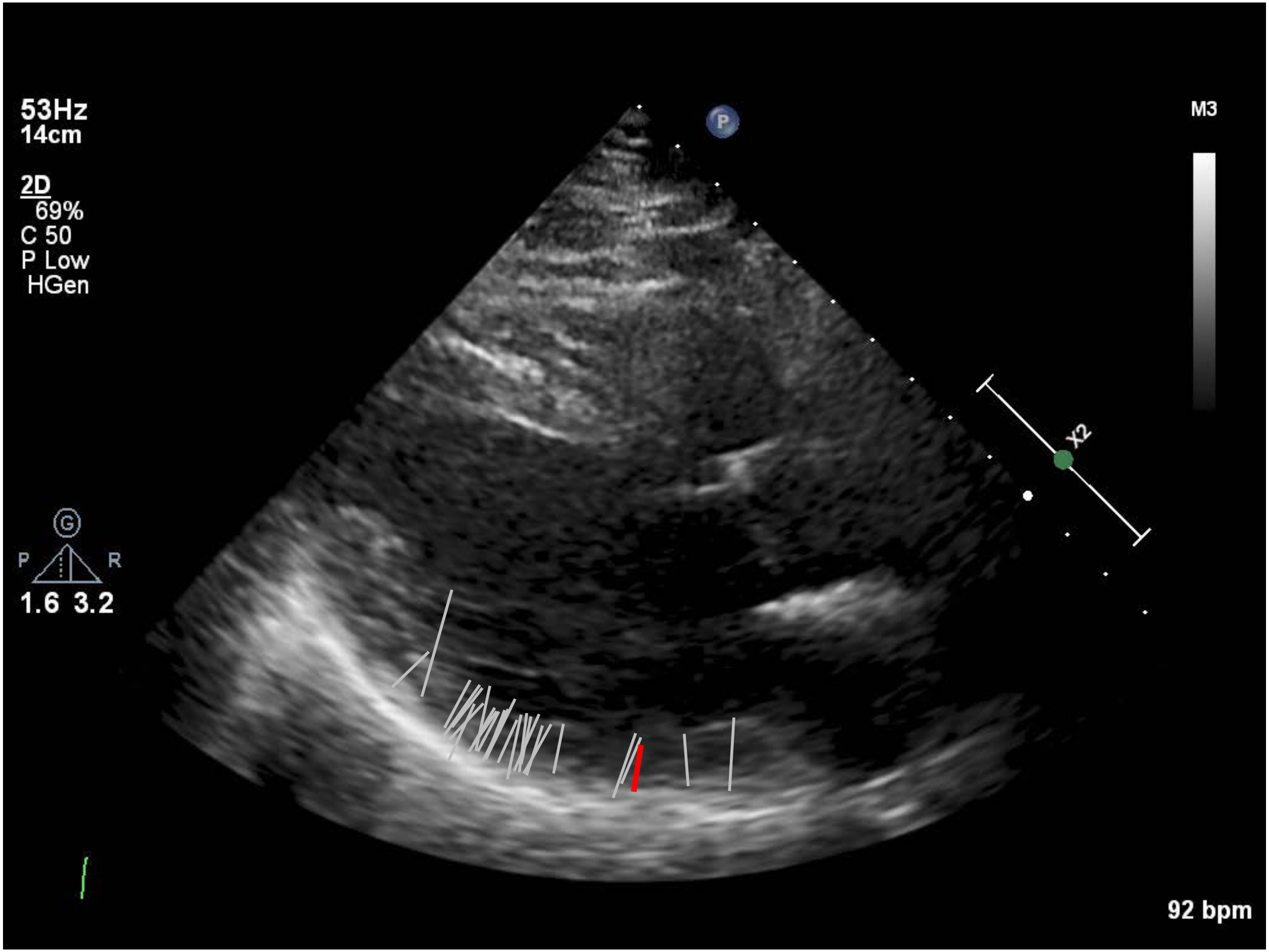

Pt 70 Diastole
Absolute difference between AI and expert consensus: 0.93 mm

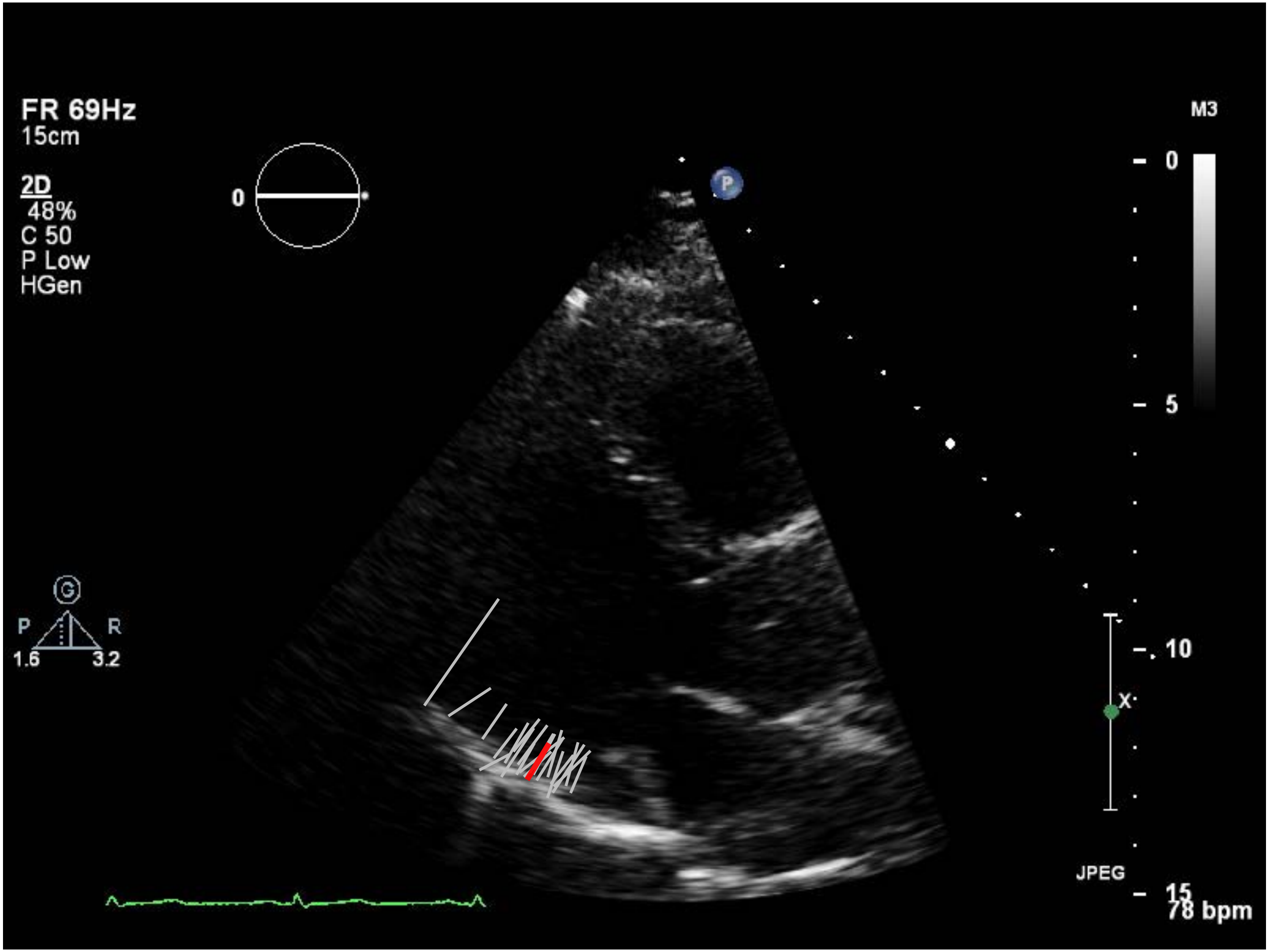

Pt 59 Diastole

Absolute difference between AI and expert consensus: 0.99 mm

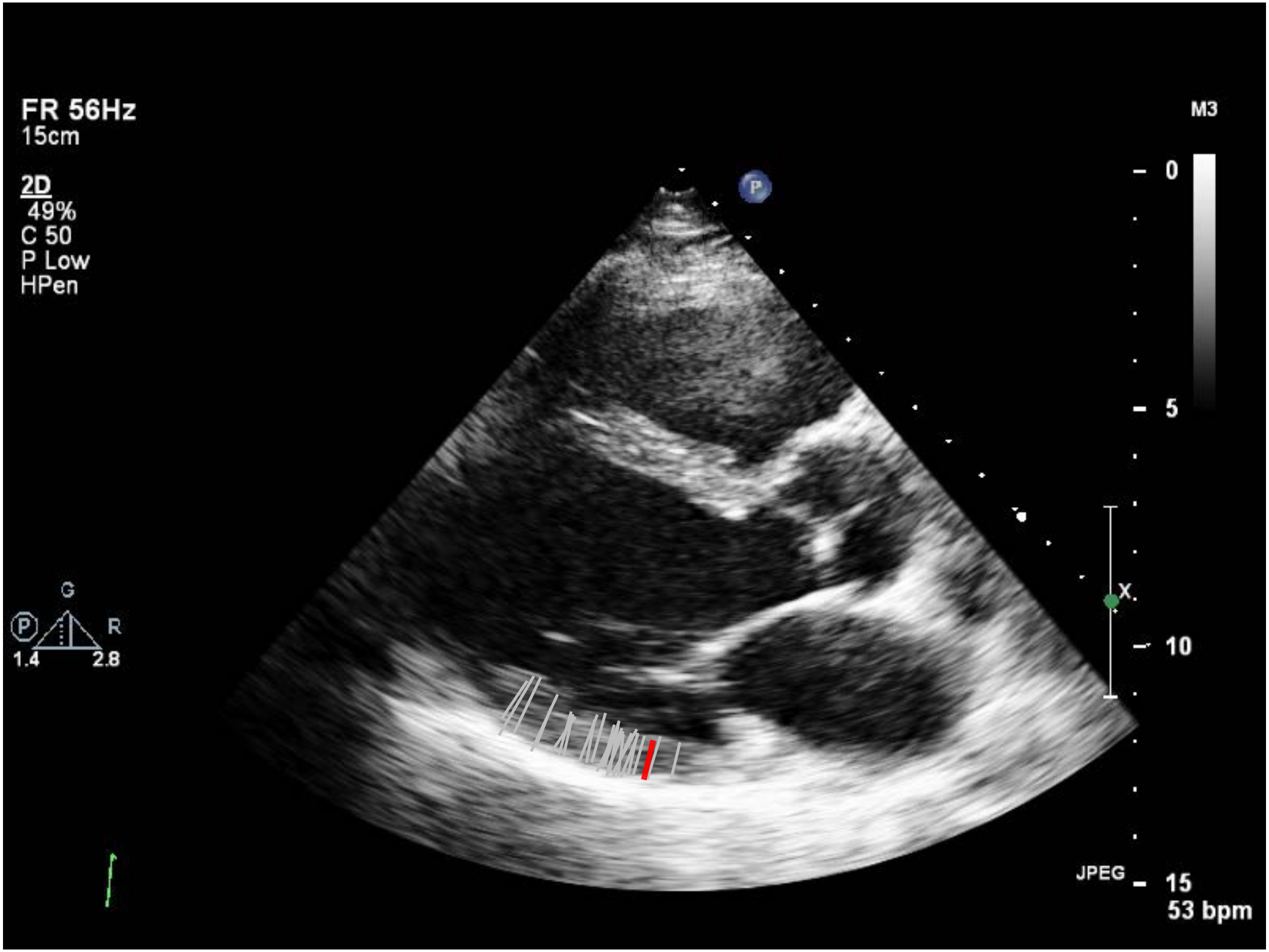

Pt 29 Diastole

Absolute difference between AI and expert consensus: 0.99 mm

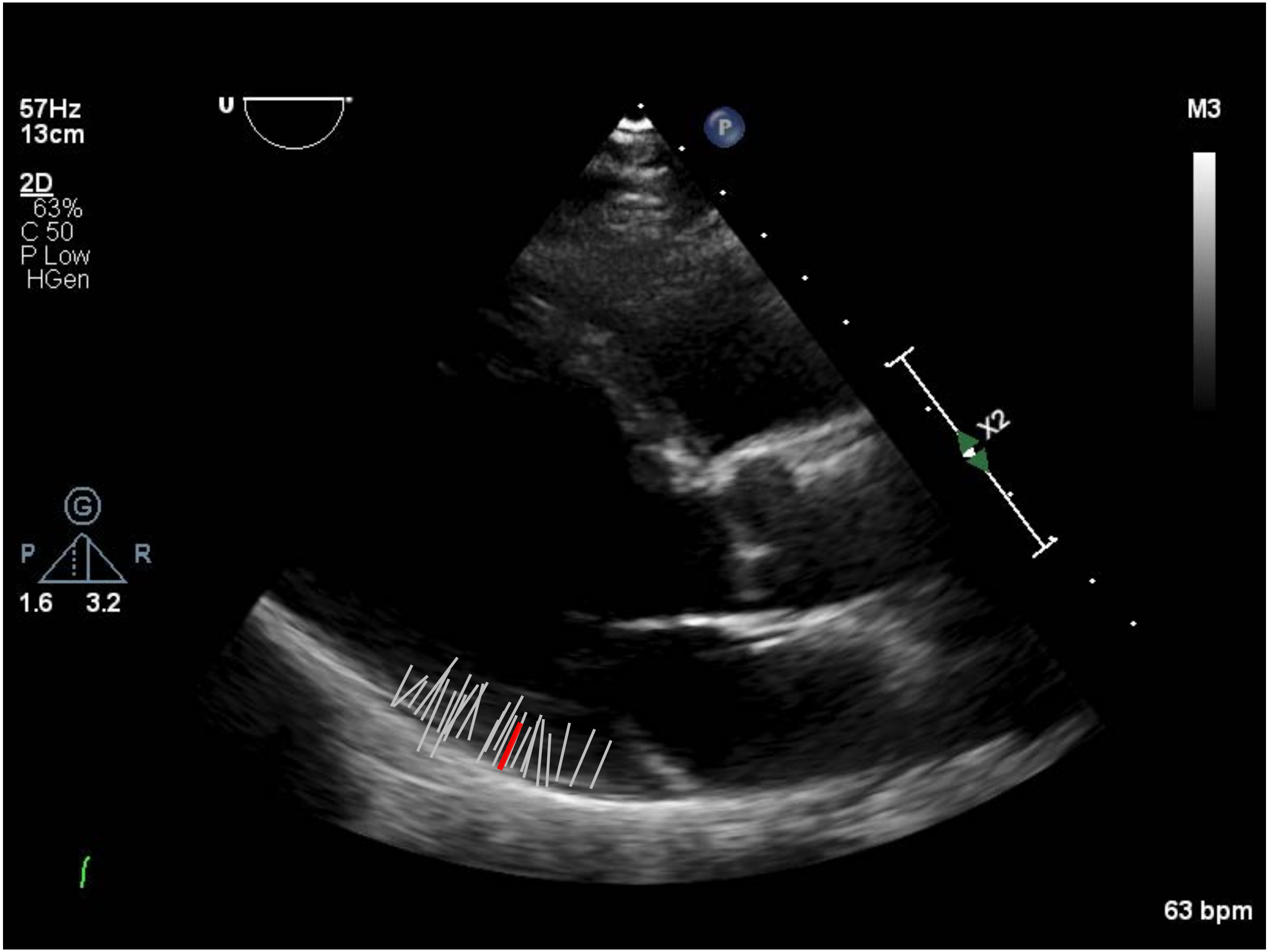

Pt 53 Diastole

Absolute difference between AI and expert consensus: 1 mm

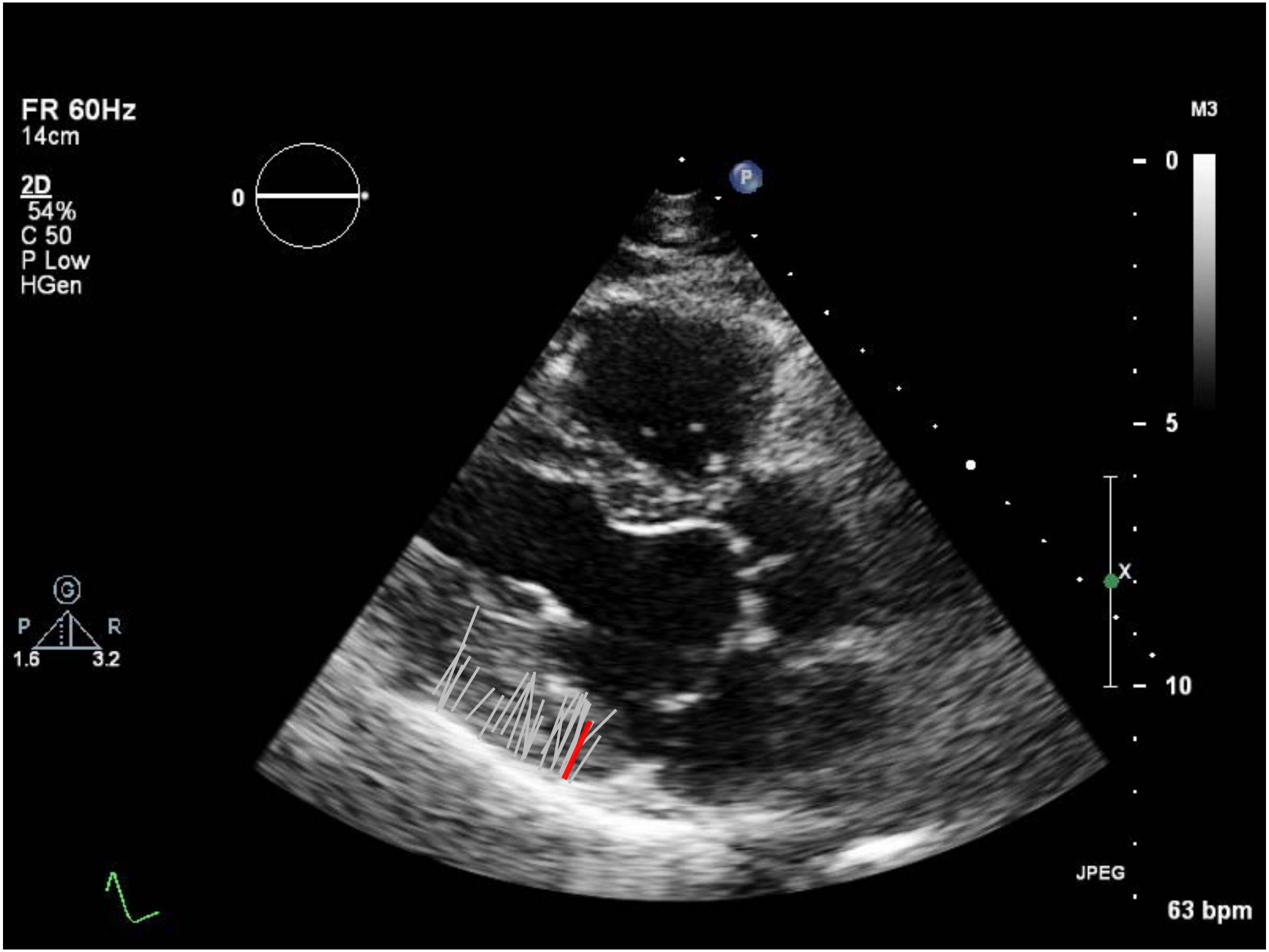

Pt 10 Diastole

Absolute difference between AI and expert consensus: 1.1 mm

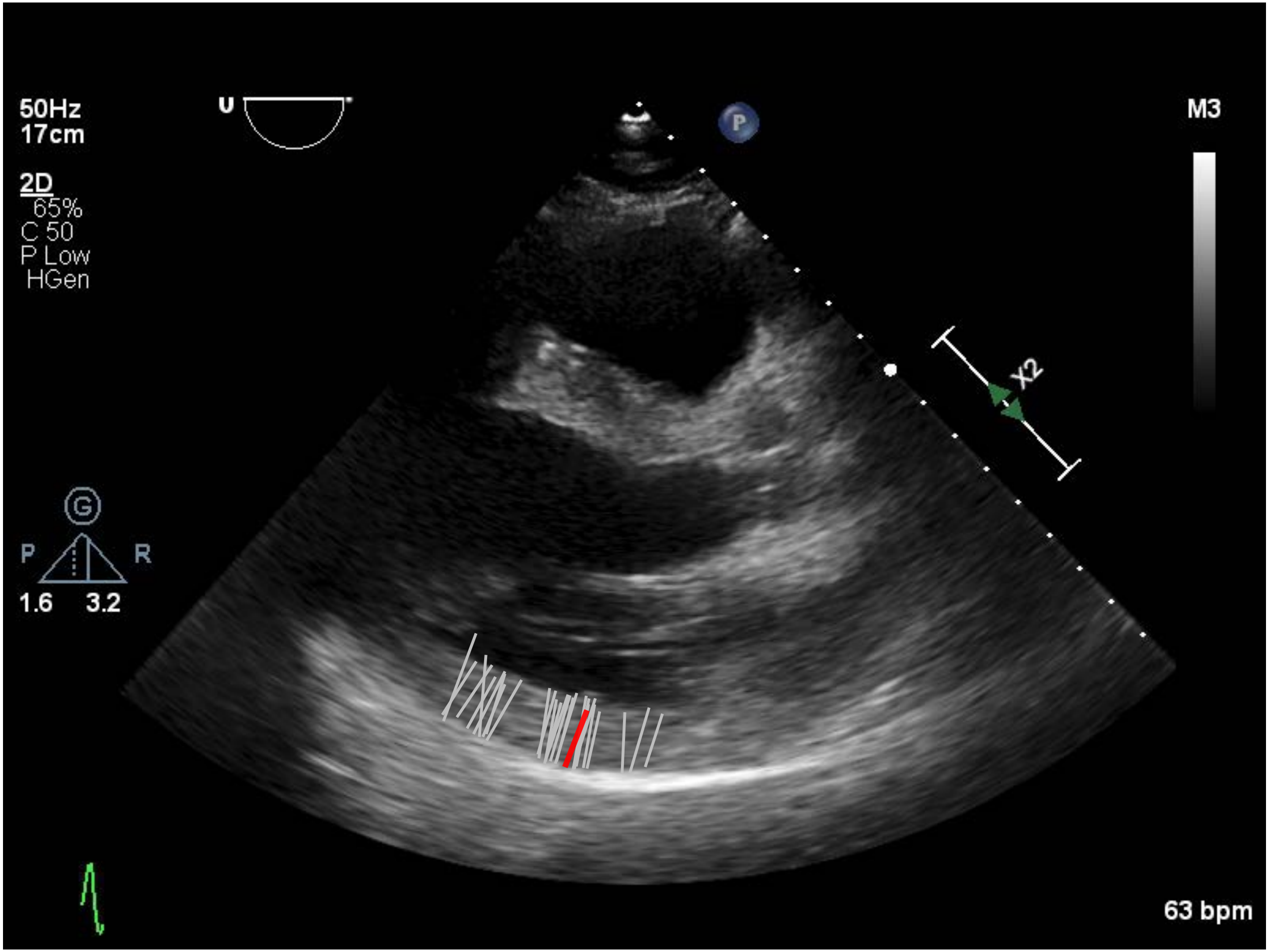

Pt 68 Diastole

Absolute difference between AI and expert consensus: 1.1 mm

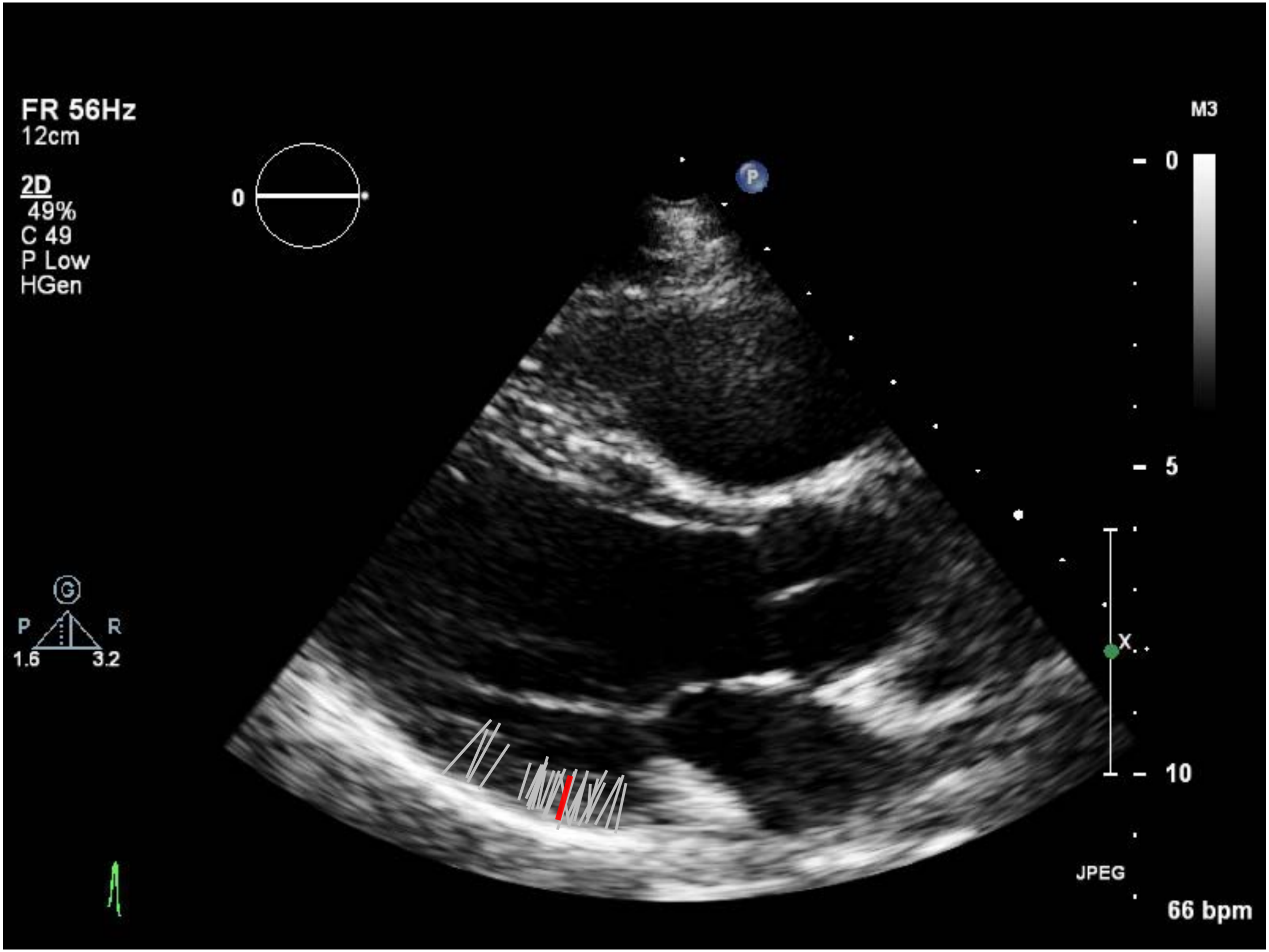

Pt 40 Diastole

Absolute difference between AI and expert consensus: 1.1 mm

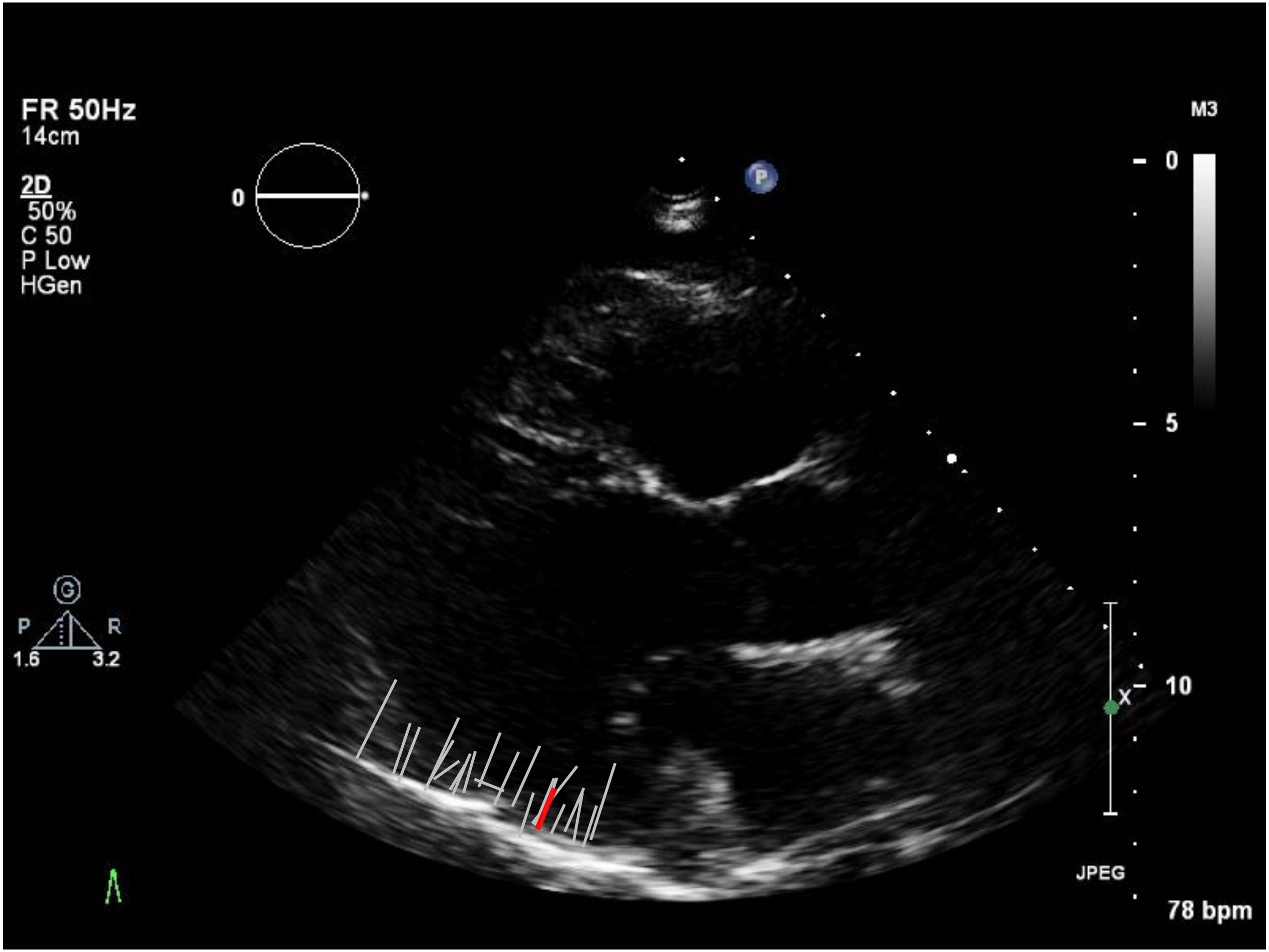

Pt 50 Diastole

Absolute difference between AI and expert consensus: 1.1 mm

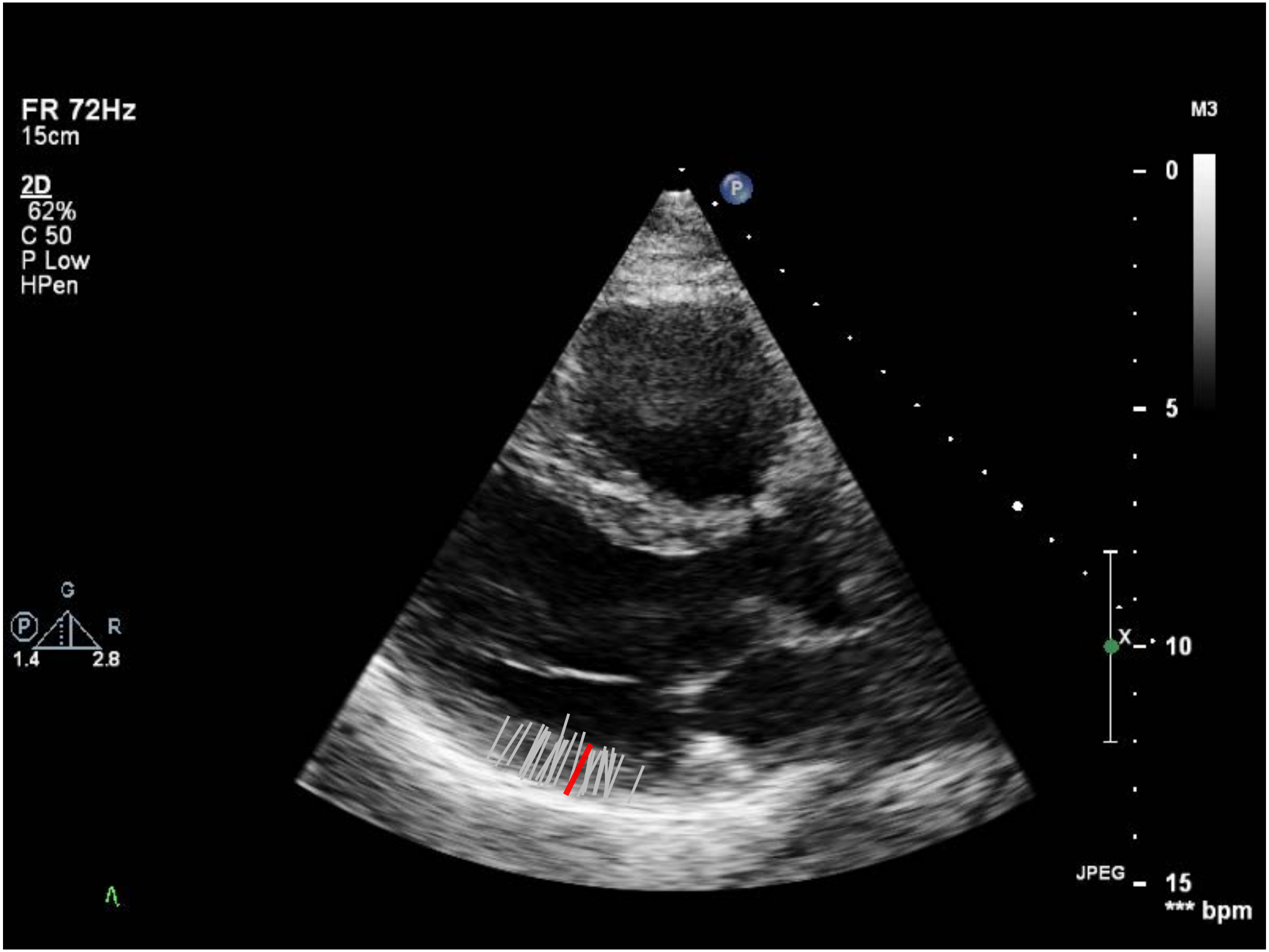

Pt 94 Diastole

Absolute difference between AI and expert consensus: 1.2 mm

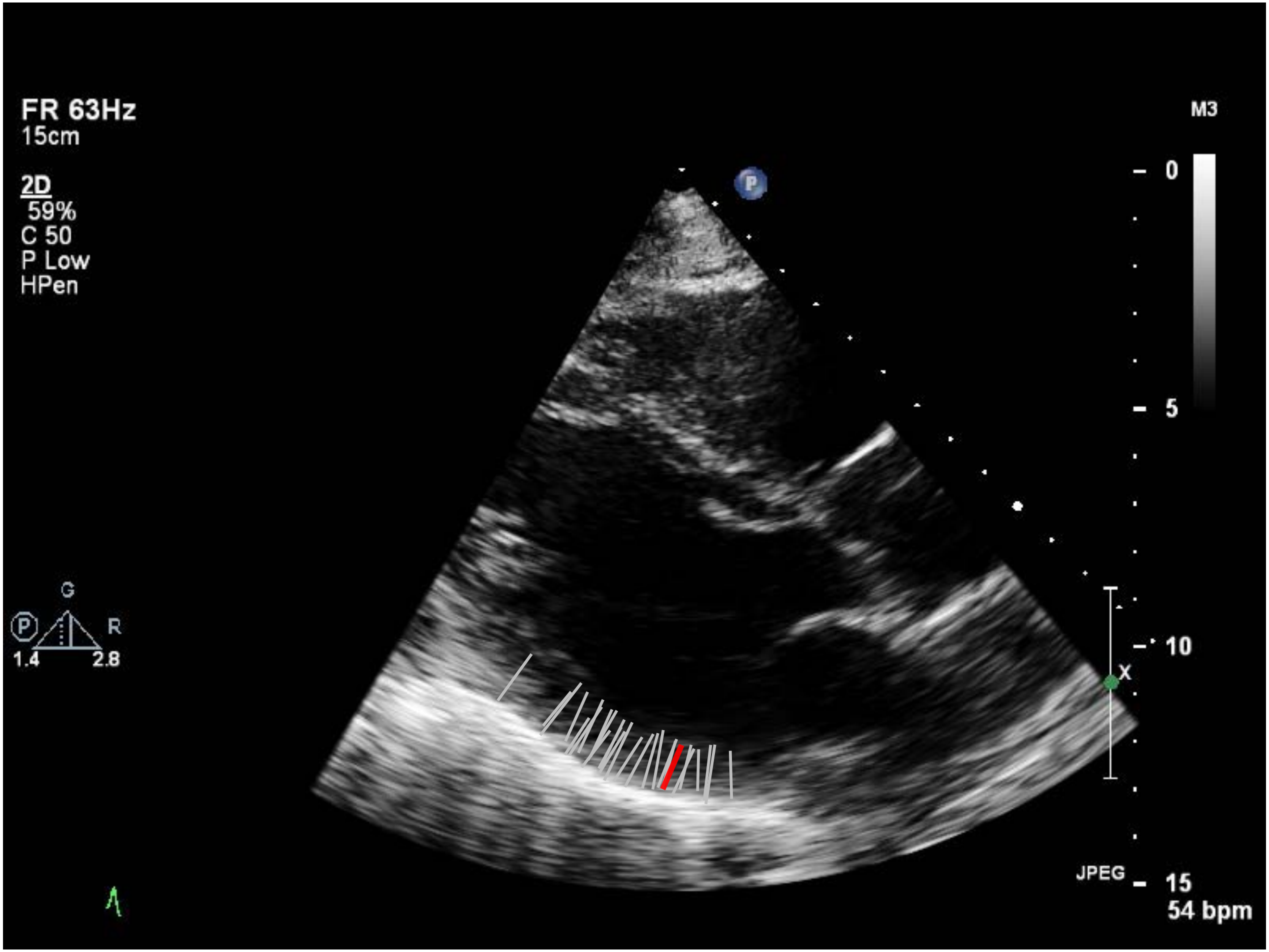

Pt 69 Diastole

Absolute difference between AI and expert consensus: 1.3 mm

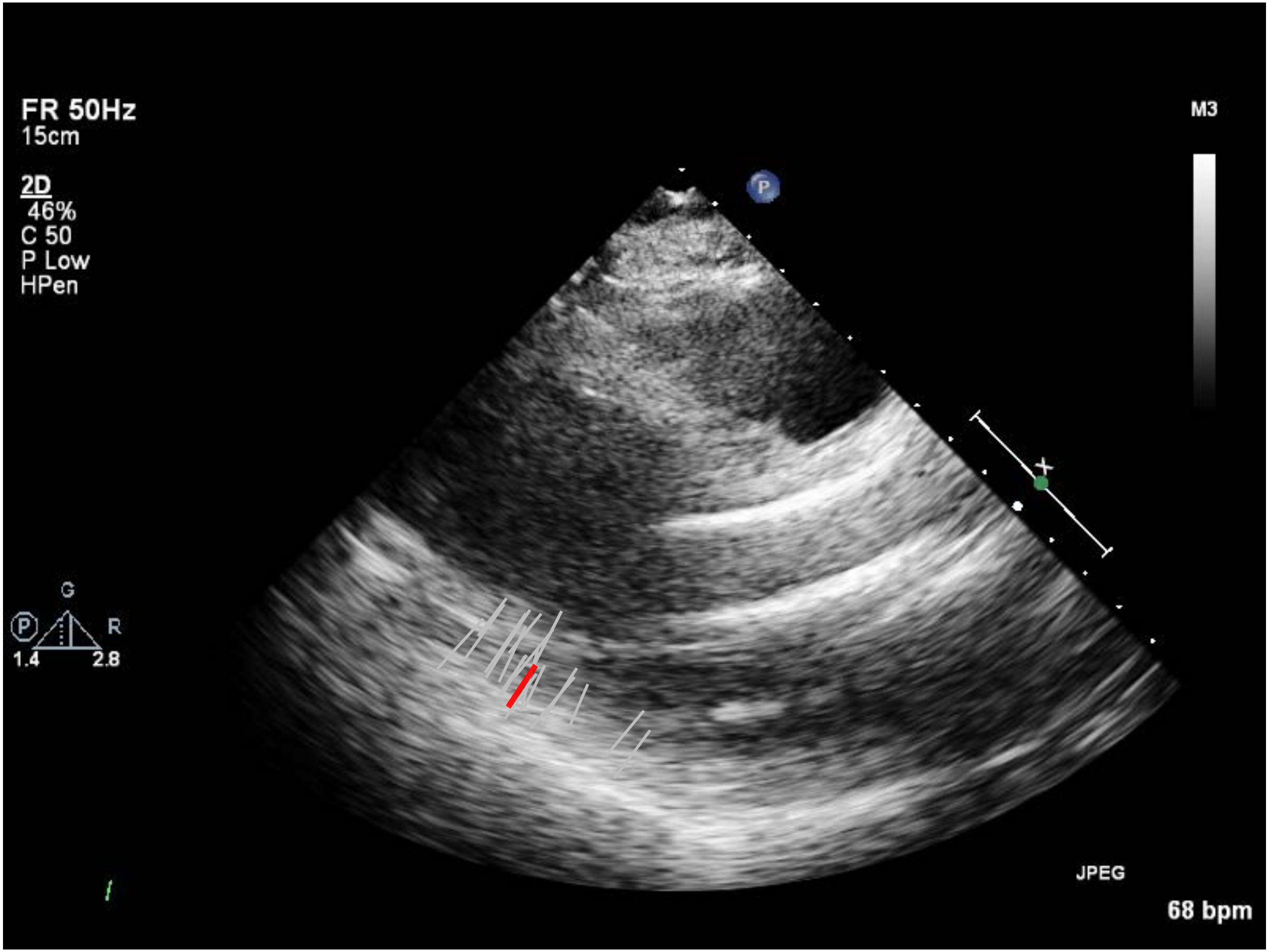

Pt 81 Diastole

Absolute difference between AI and expert consensus: 1.3 mm

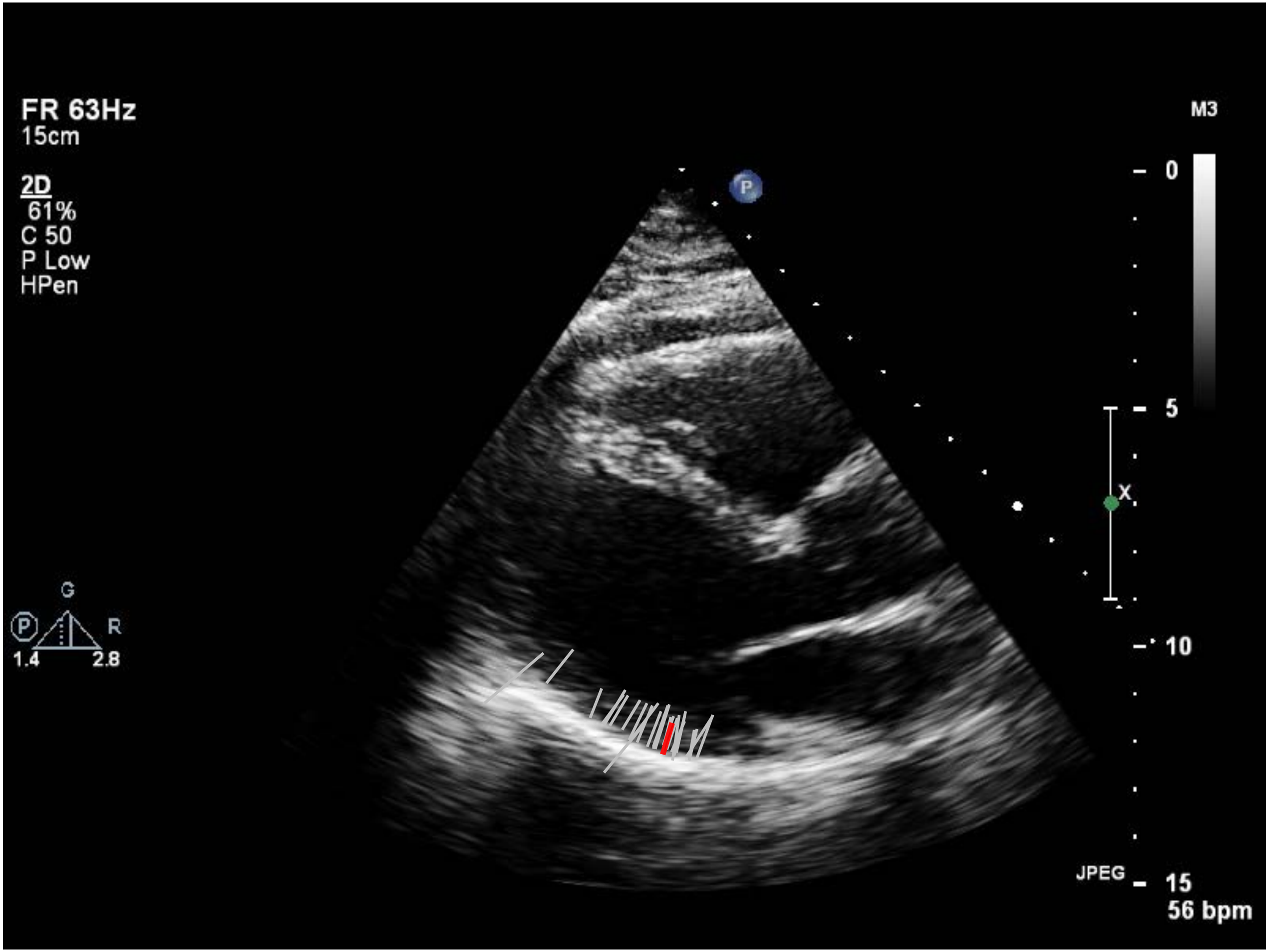

Pt 98 Diastole

Absolute difference between AI and expert consensus: 1.3 mm

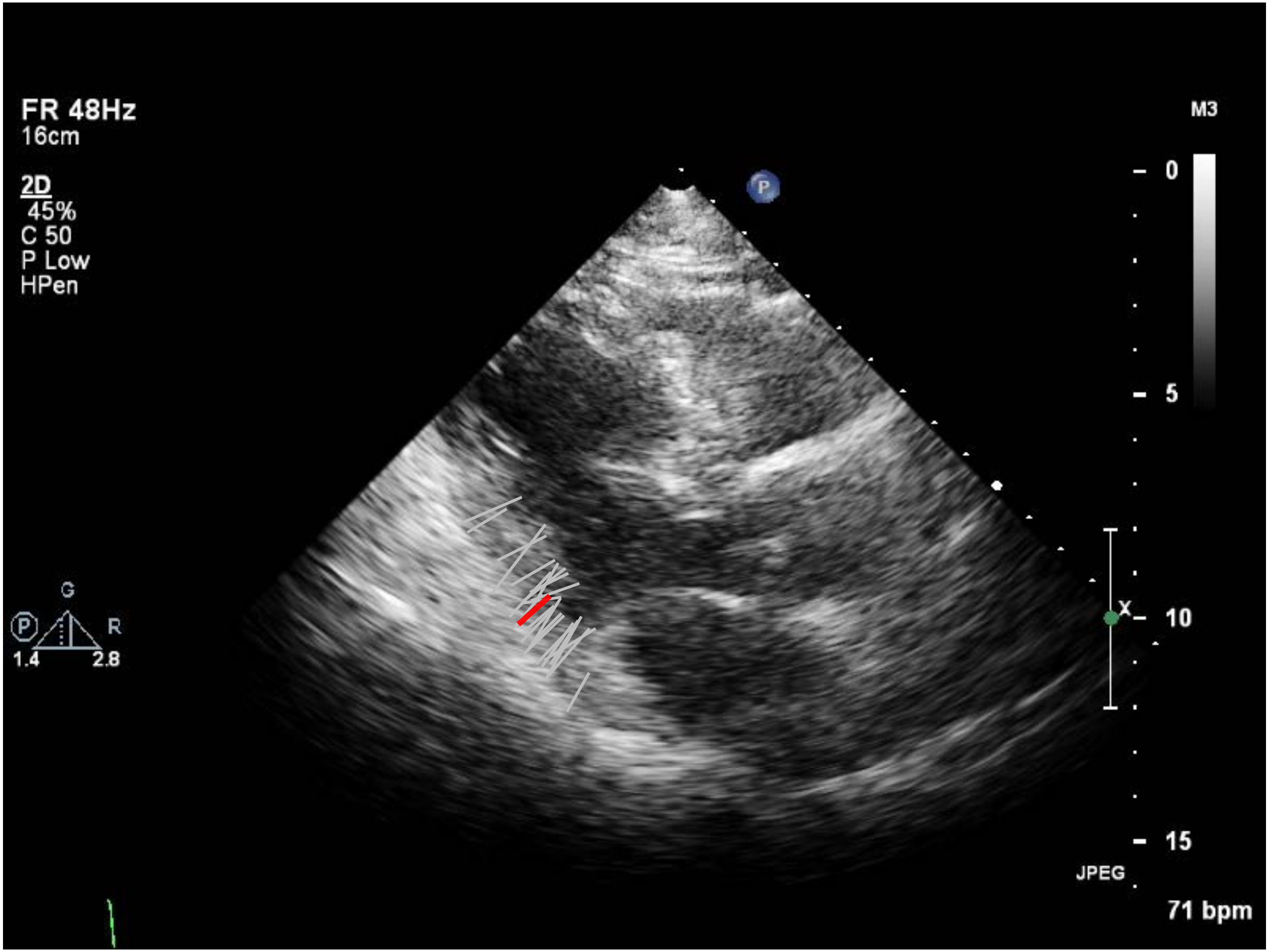

Pt 71 Diastole

Absolute difference between AI and expert consensus: 1.3 mm

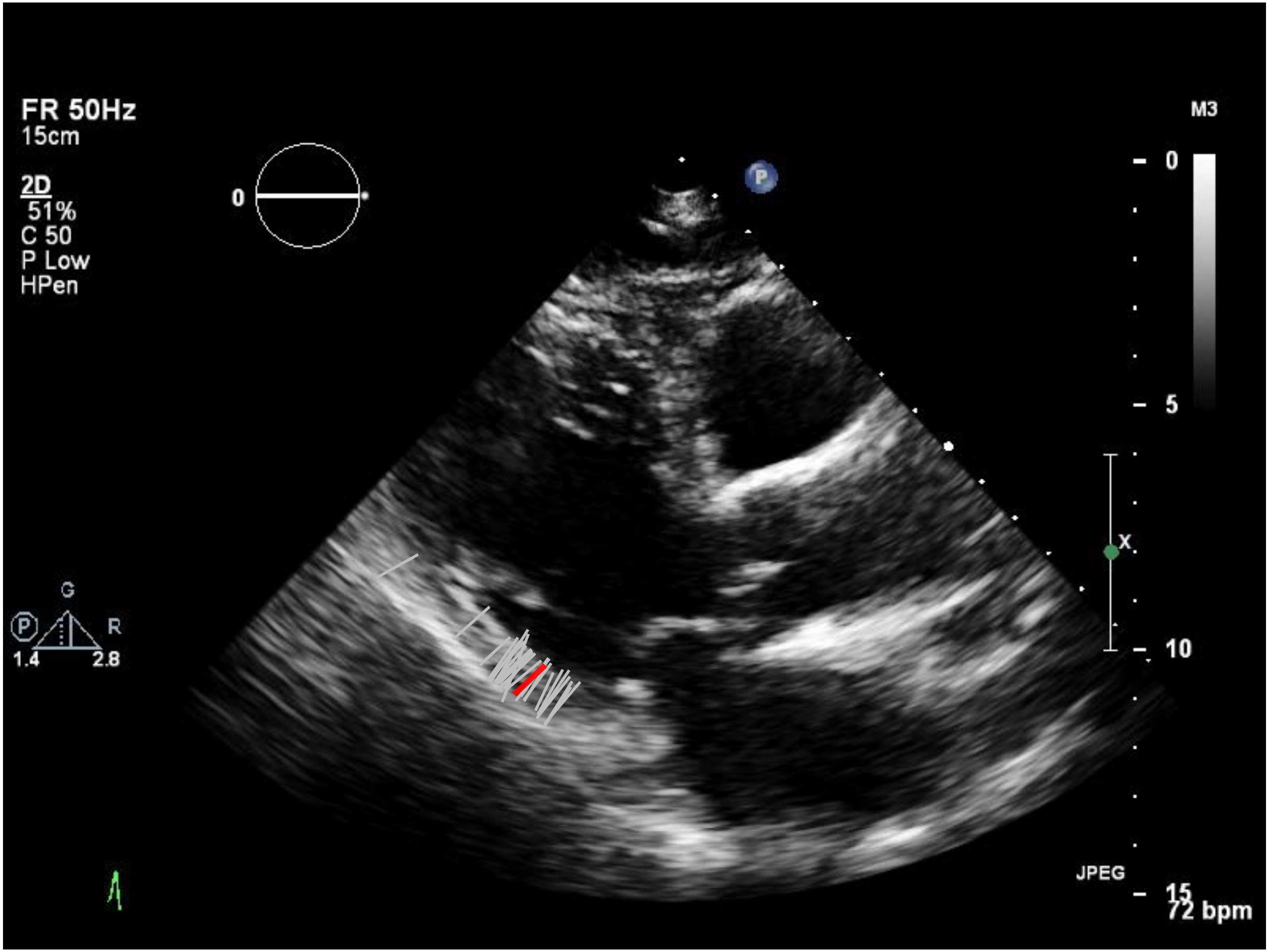

Pt 30 Diastole

Absolute difference between AI and expert consensus: 1.4 mm

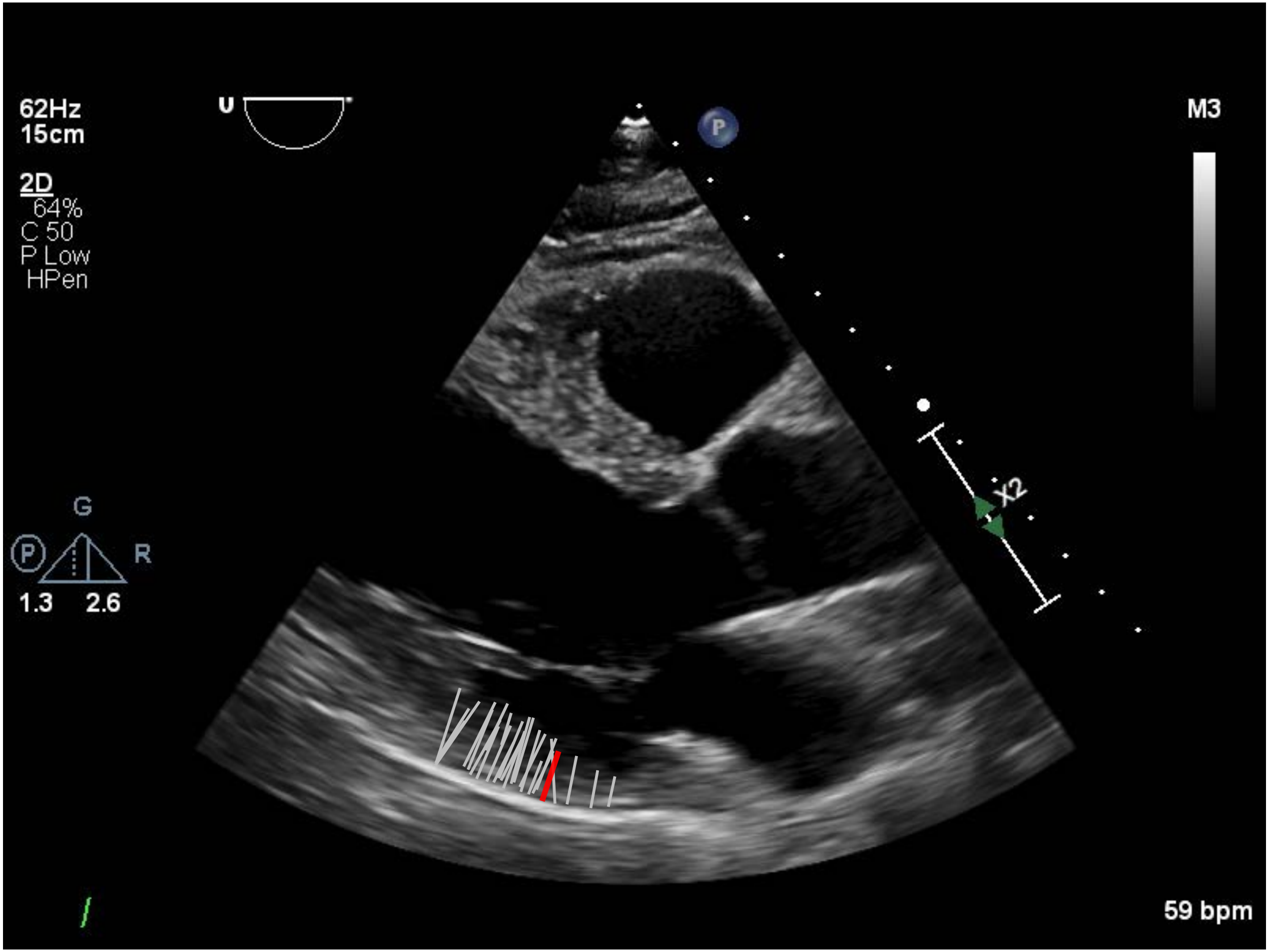

Pt 60 Diastole

Absolute difference between AI and expert consensus: 1.4 mm

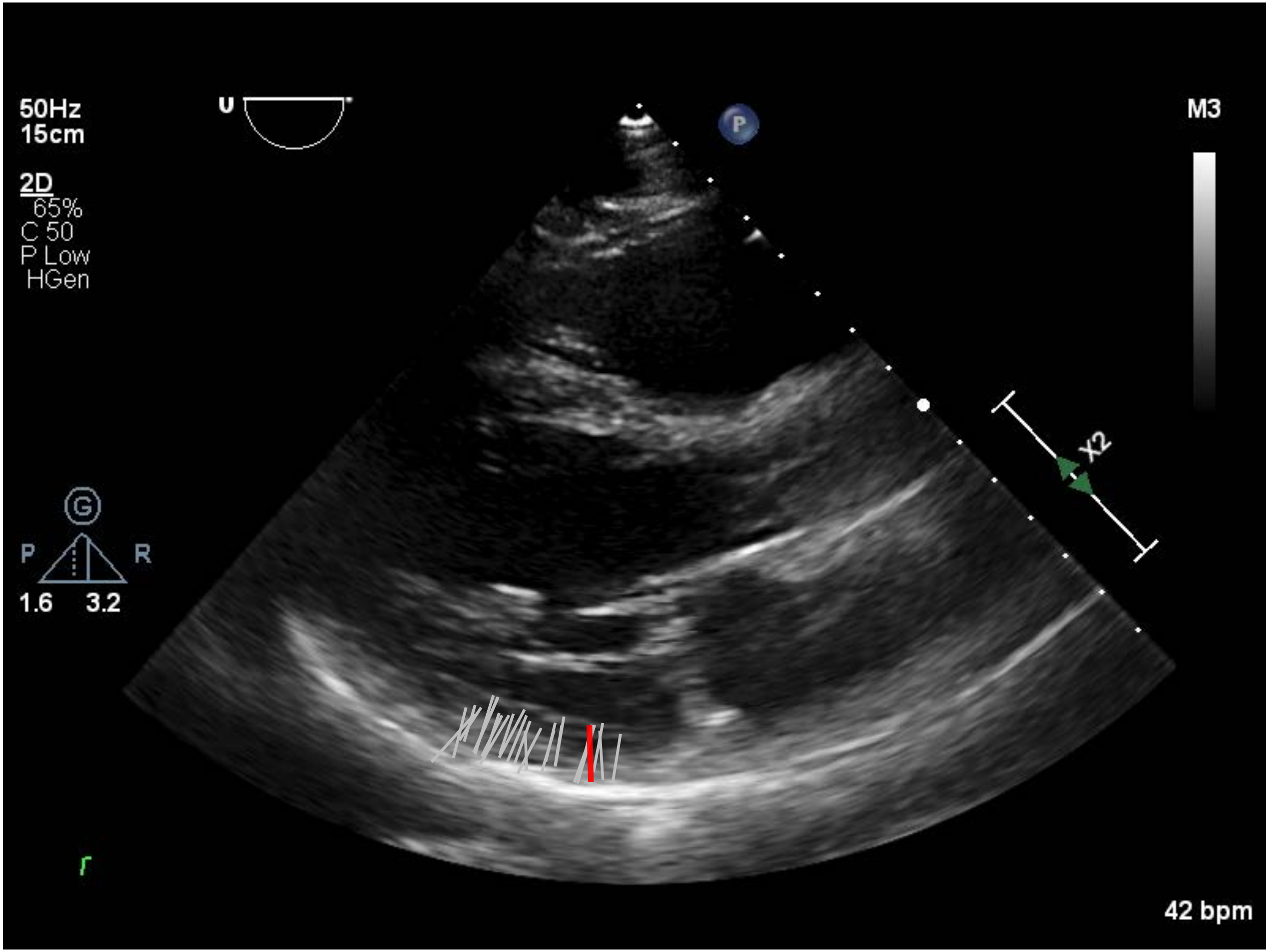

Pt 22 Diastole

Absolute difference between AI and expert consensus: 1.4 mm

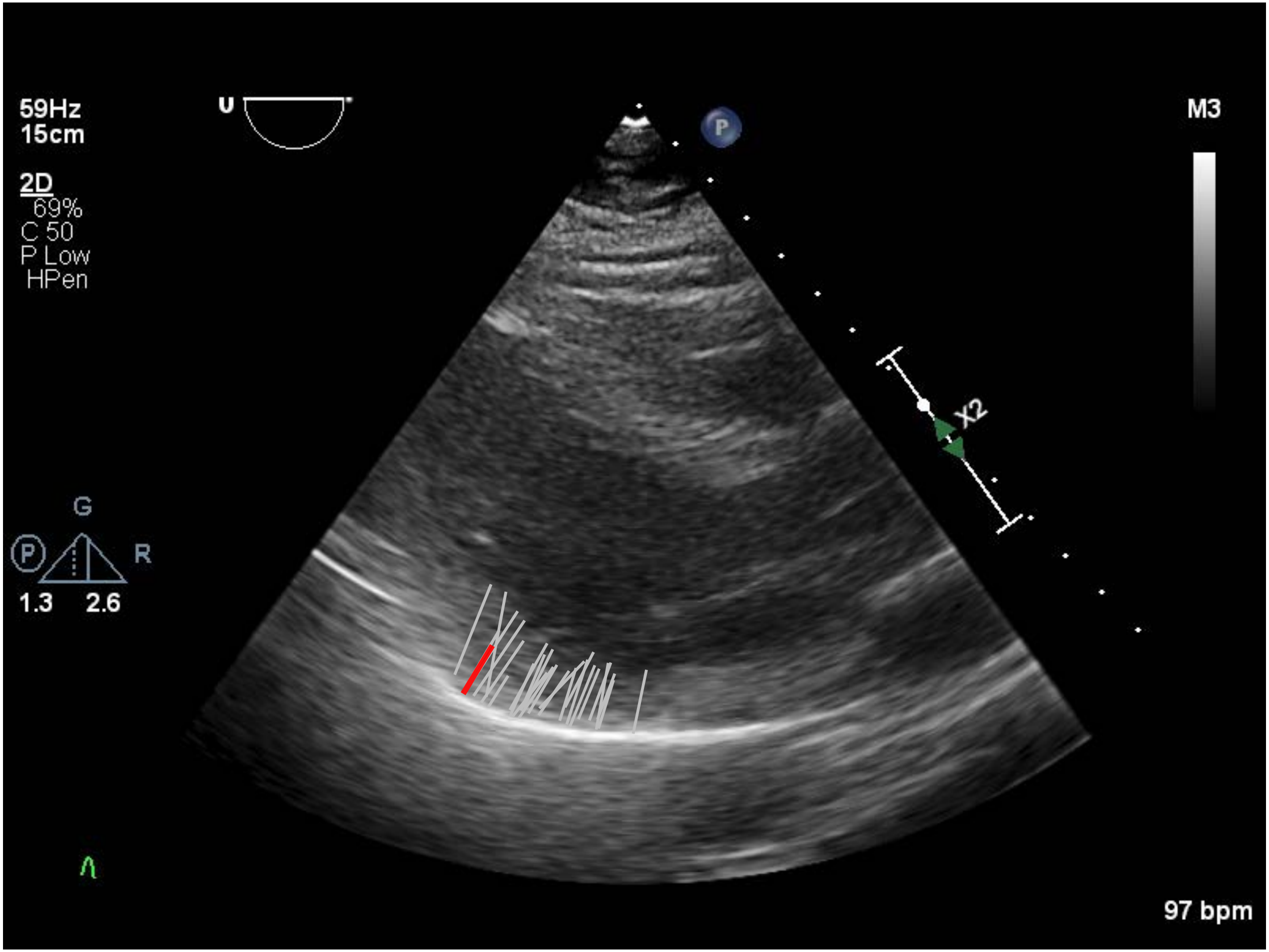

Pt 39 Diastole

Absolute difference between AI and expert consensus: 1.4 mm

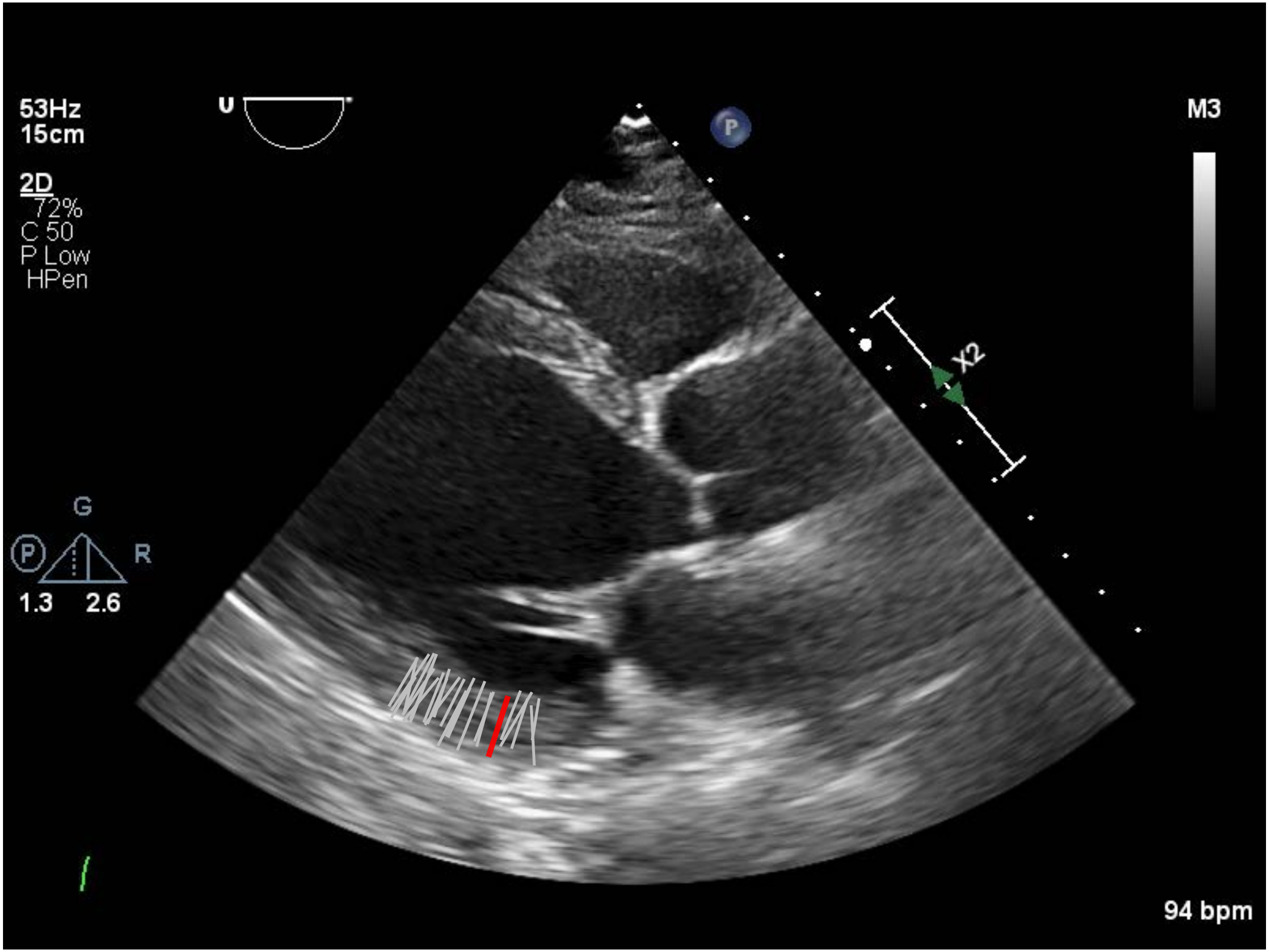

Pt 61 Diastole

Absolute difference between AI and expert consensus: 1.4 mm

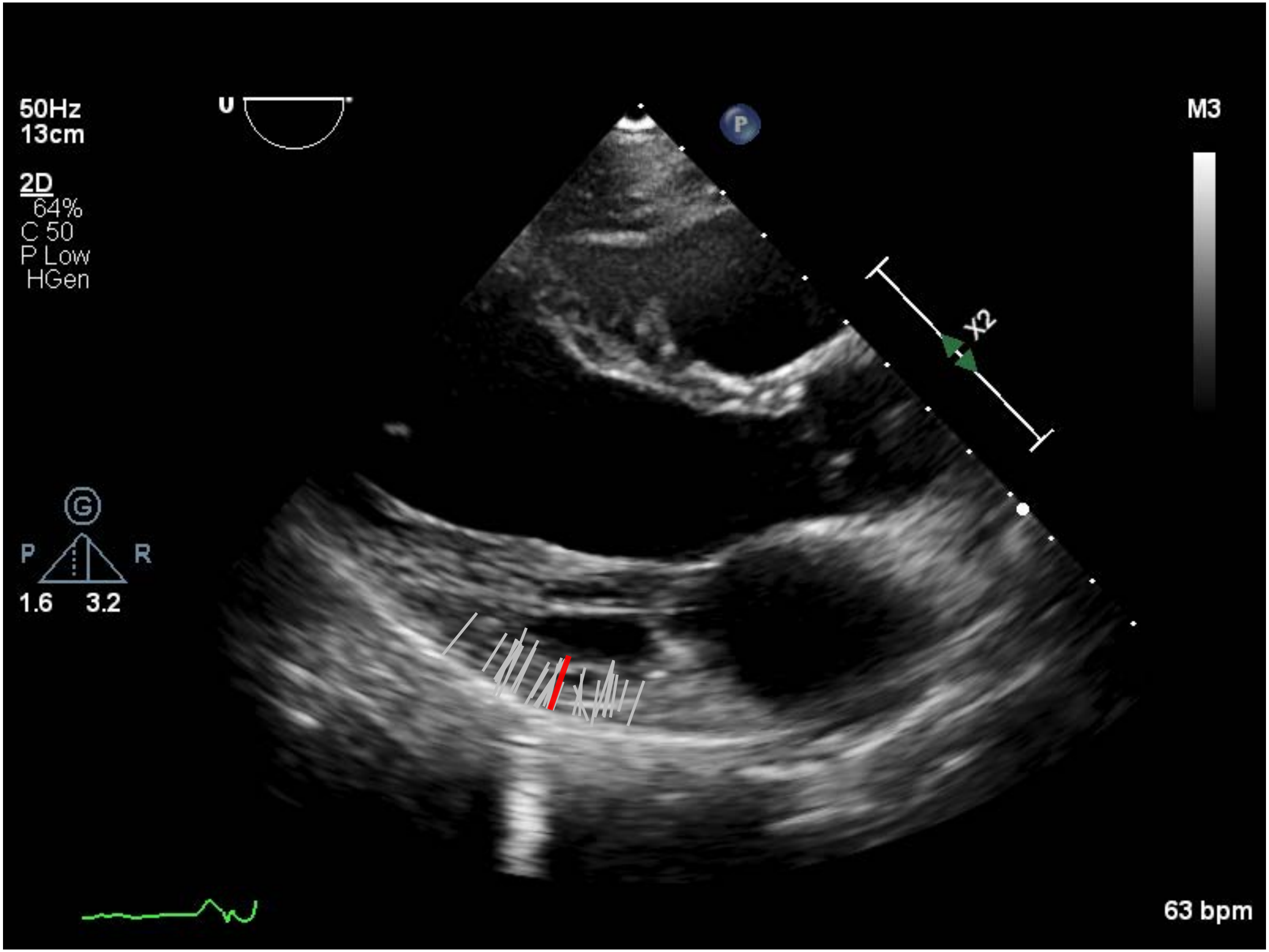

Pt 16 Diastole

Absolute difference between AI and expert consensus: 1.4 mm

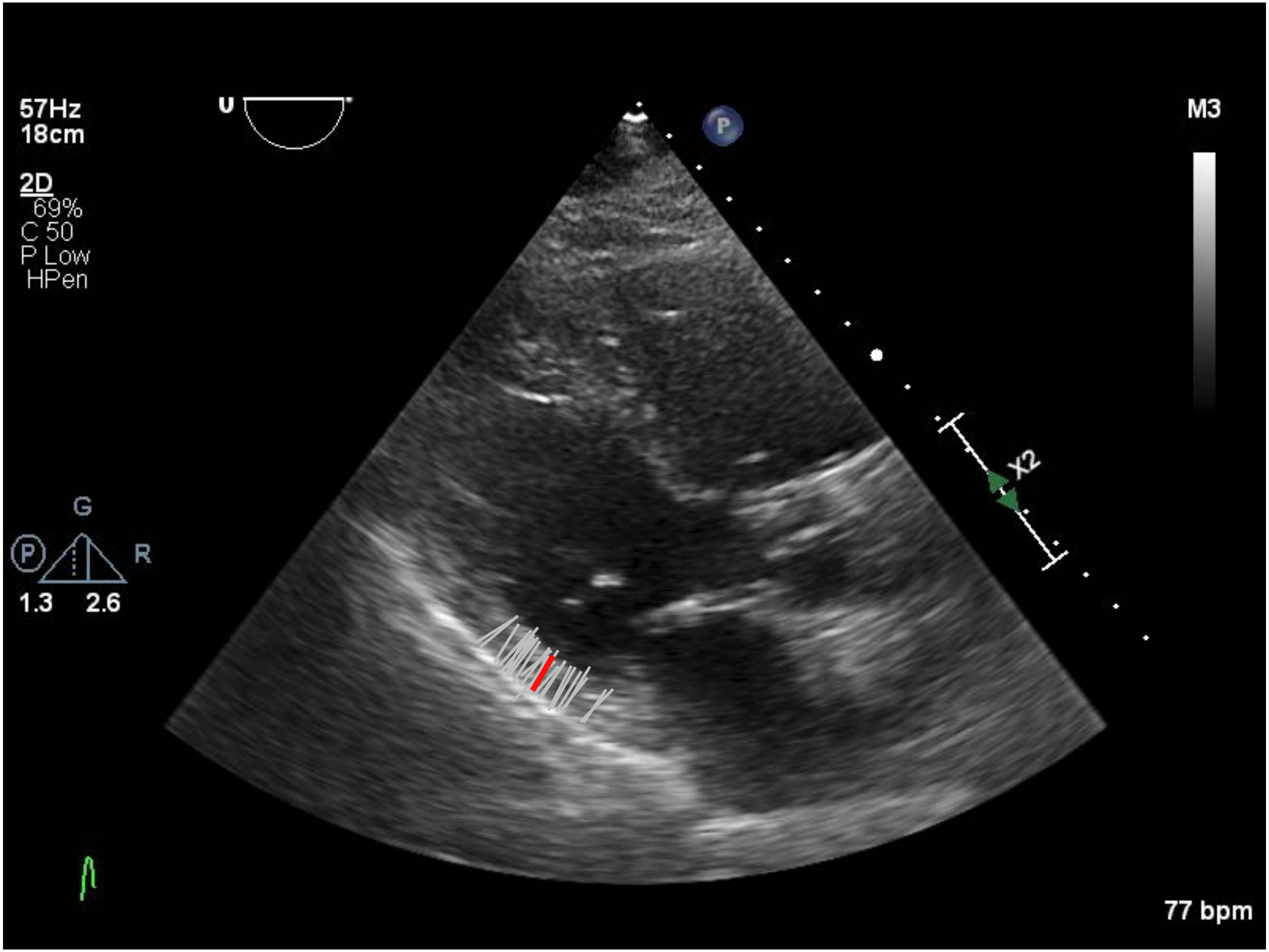

Pt 15 Diastole

Absolute difference between AI and expert consensus: 1.4 mm

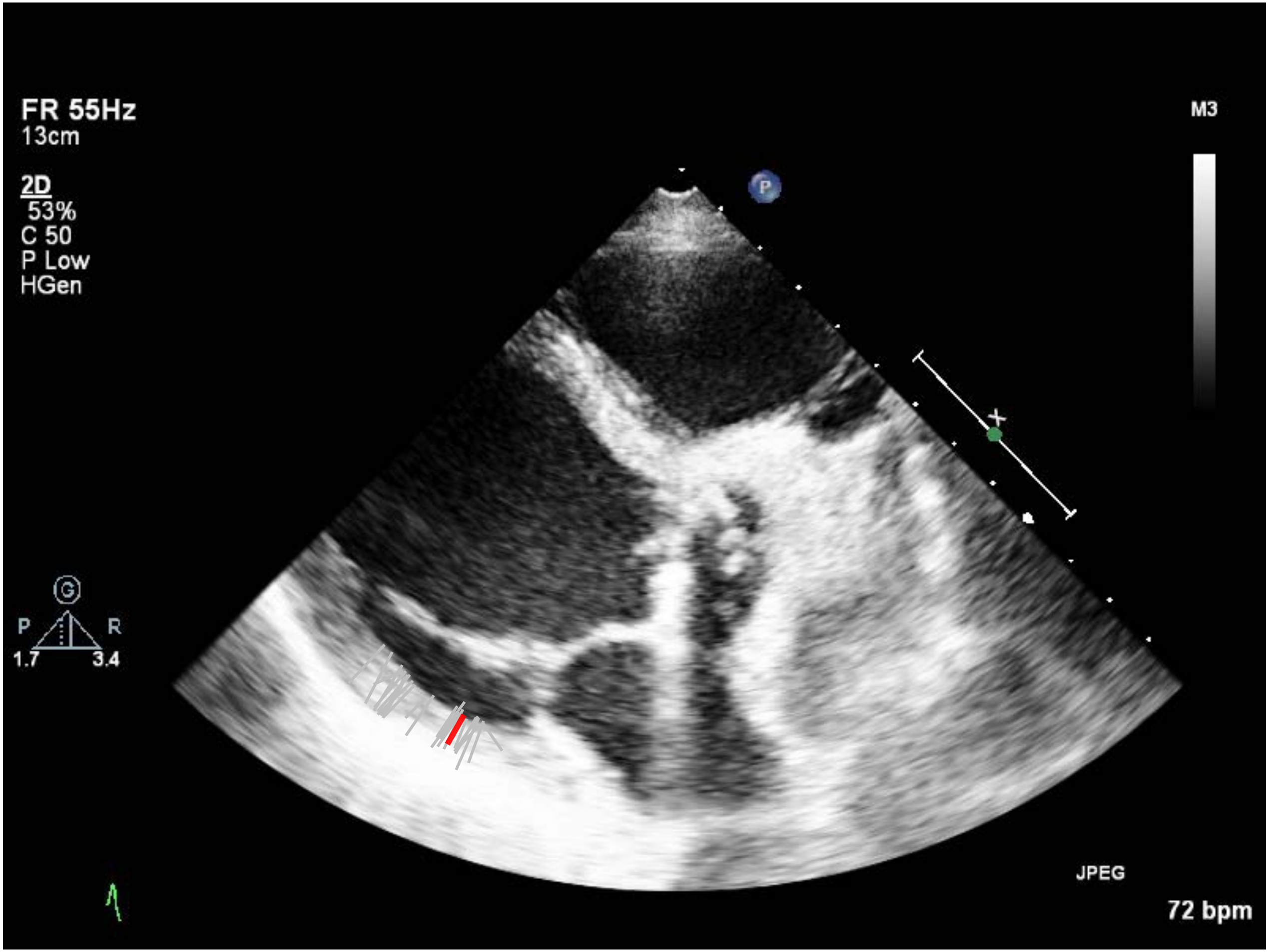

Pt 28 Diastole

Absolute difference between AI and expert consensus: 1.5 mm

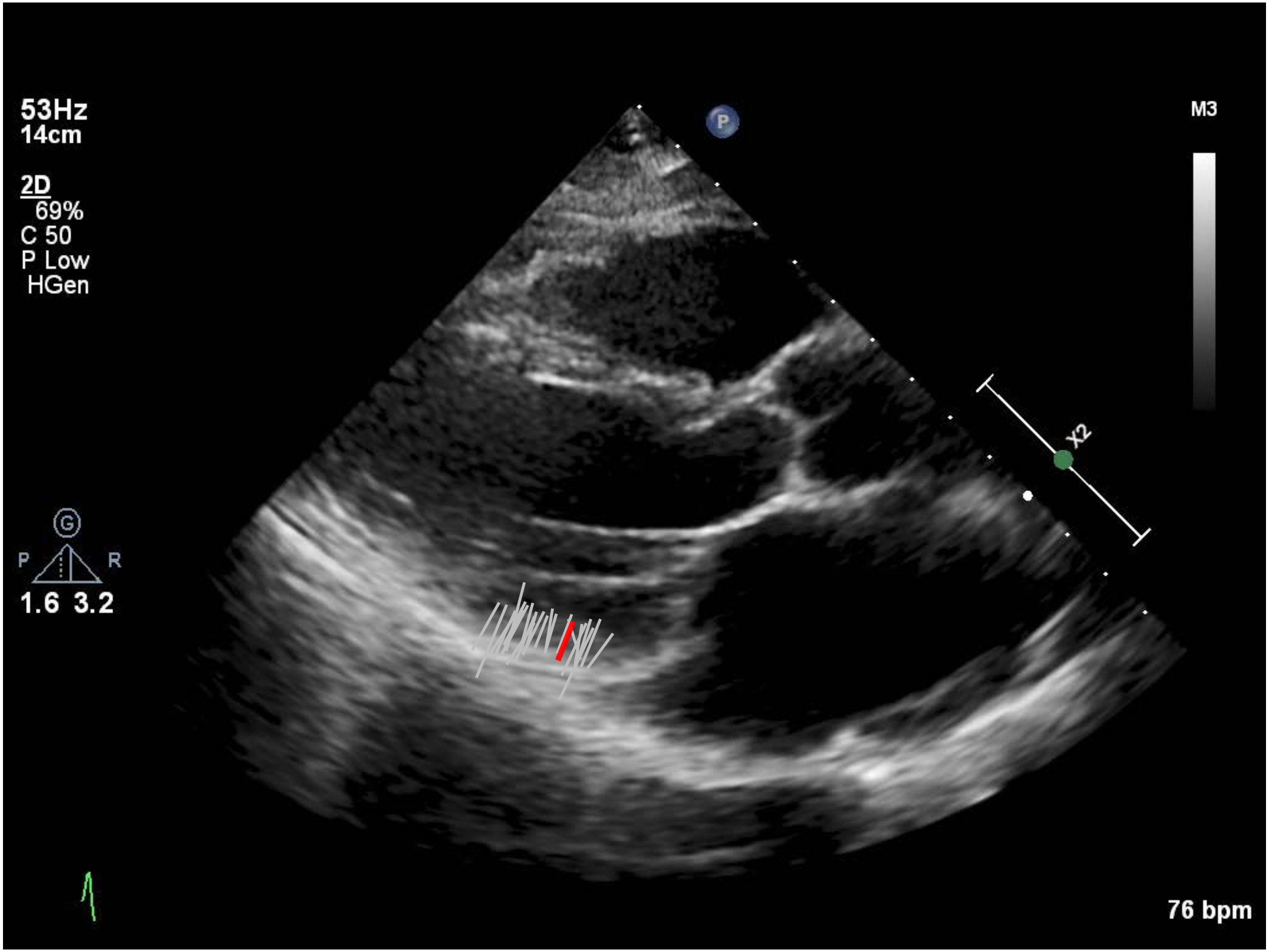

Pt 87 Diastole
Absolute difference between AI and expert consensus: 1.5 mm

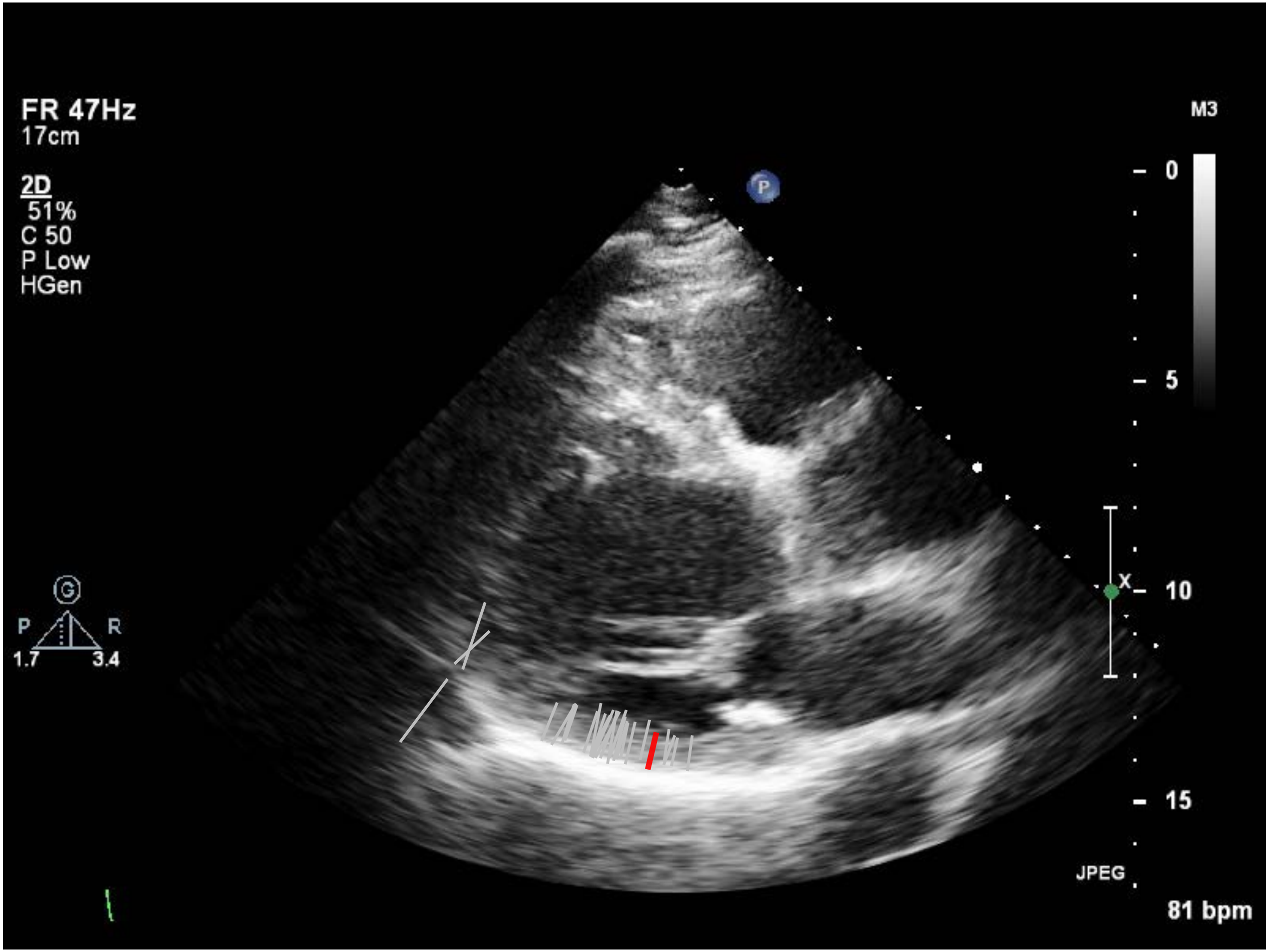

Pt 32 Diastole

Absolute difference between AI and expert consensus: 1.5 mm

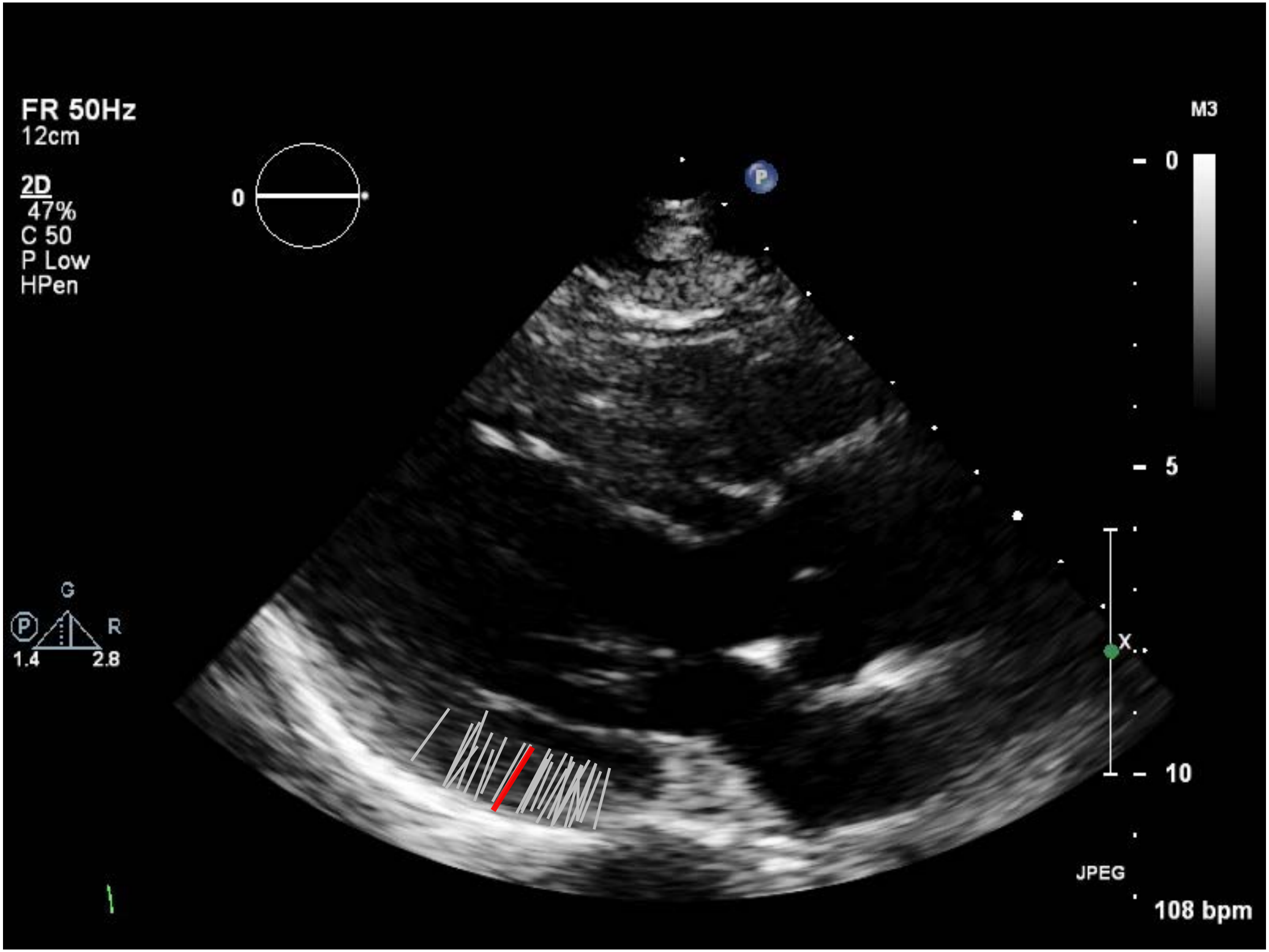

Pt 89 Diastole

Absolute difference between AI and expert consensus: 1.6 mm

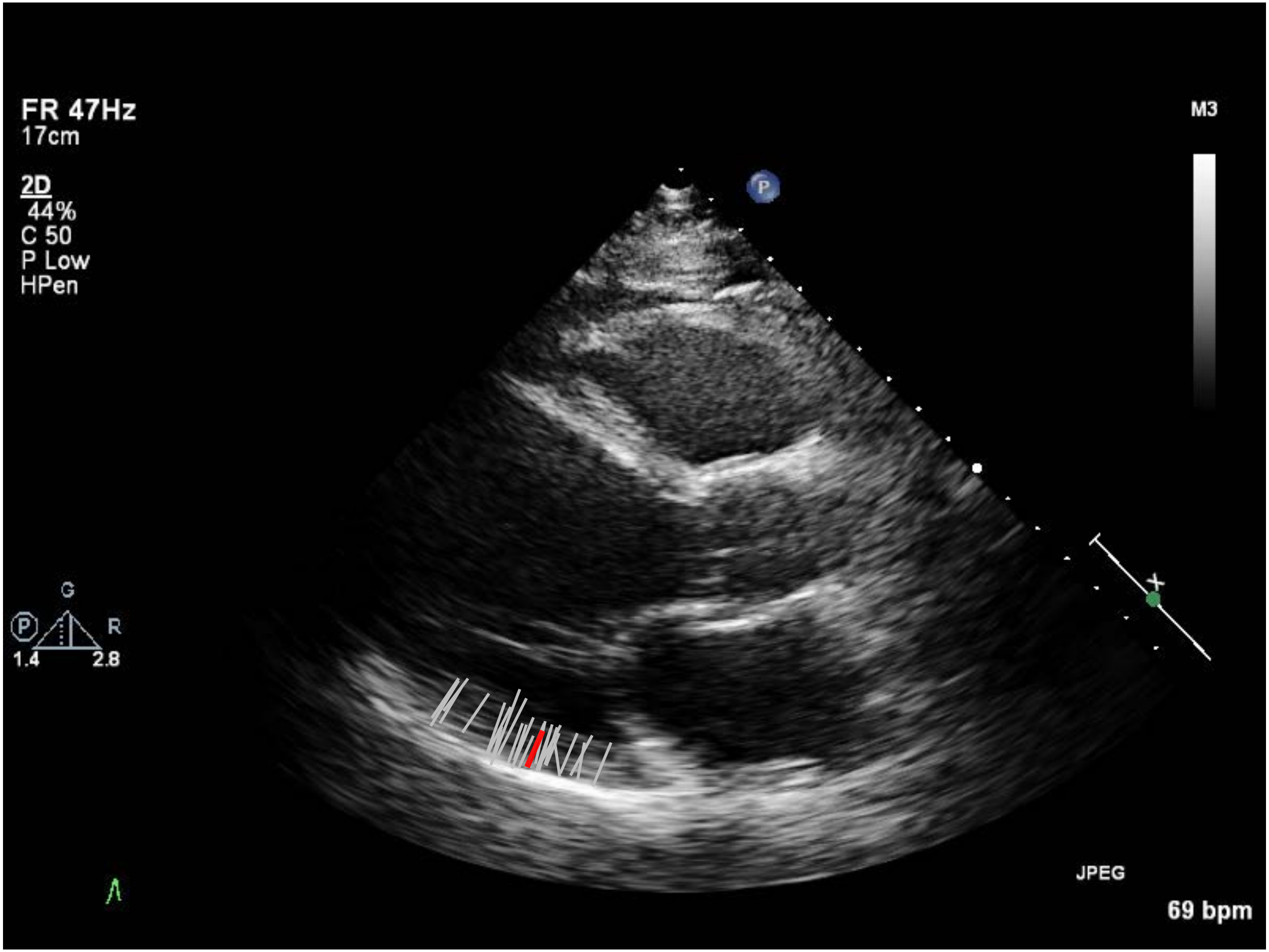

Pt 34 Diastole

Absolute difference between AI and expert consensus: 1.7 mm

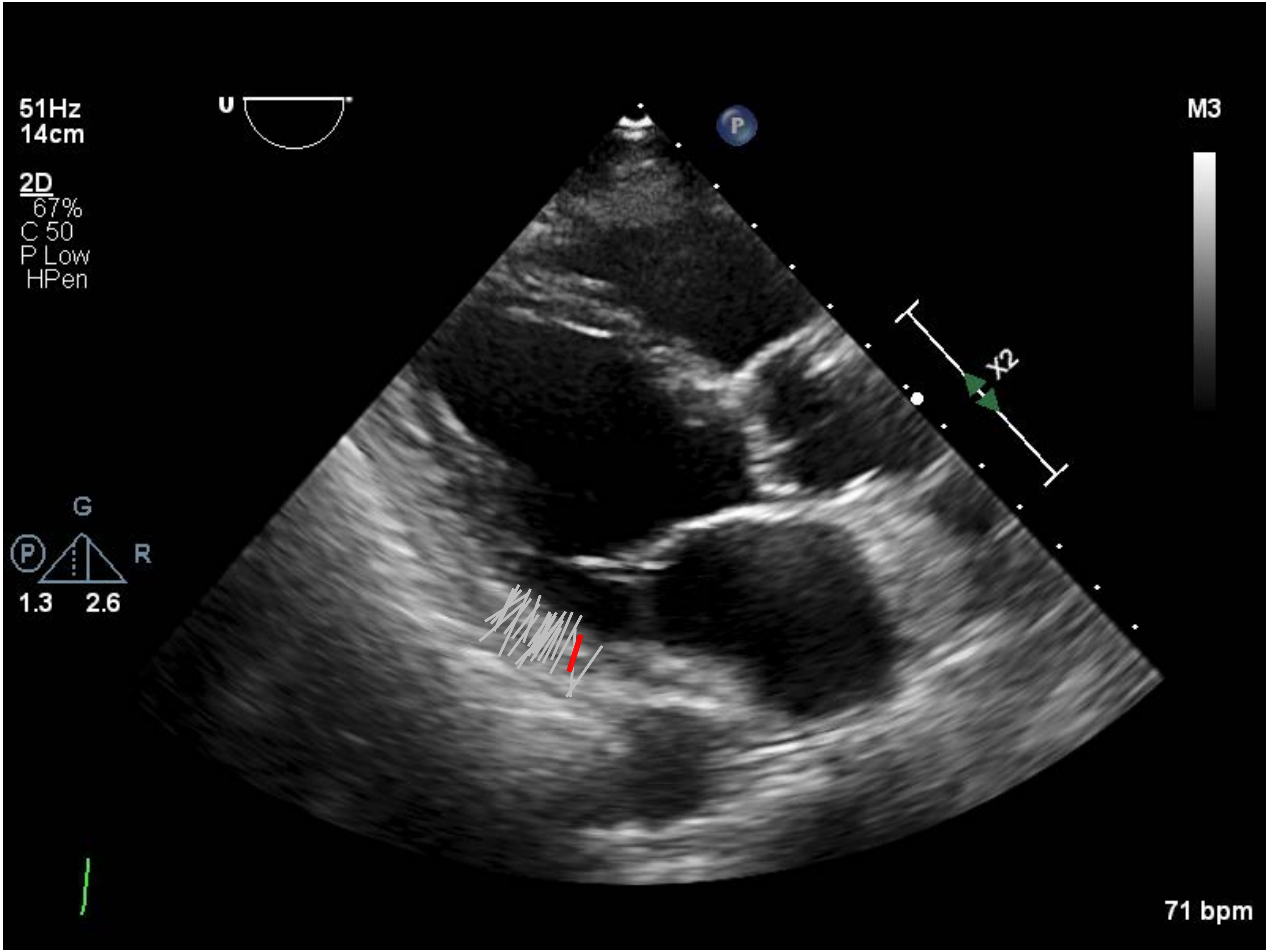

Pt 88 Diastole

Absolute difference between AI and expert consensus: 1.7 mm

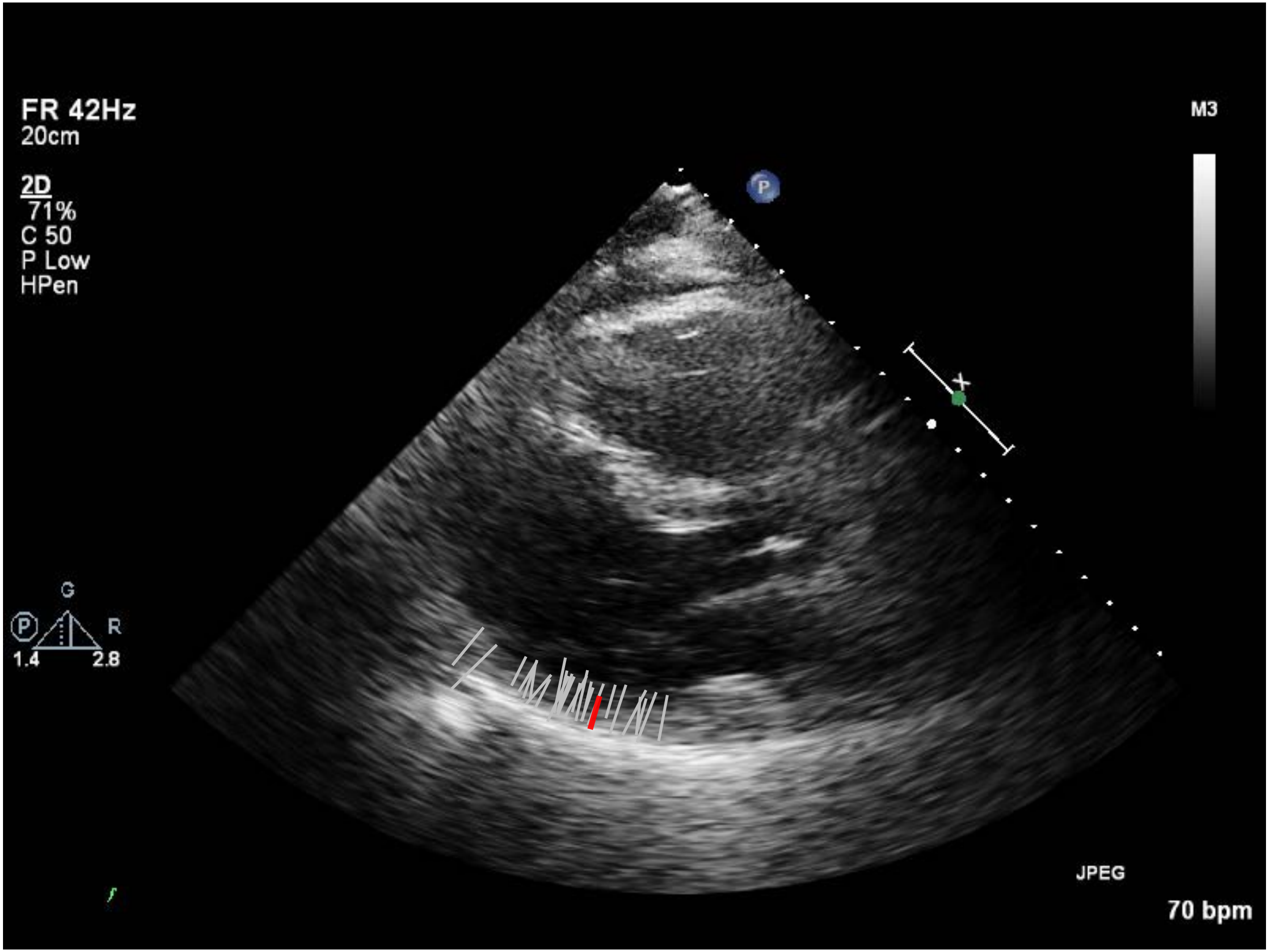

Pt 96 Diastole

Absolute difference between AI and expert consensus: 1.8 mm

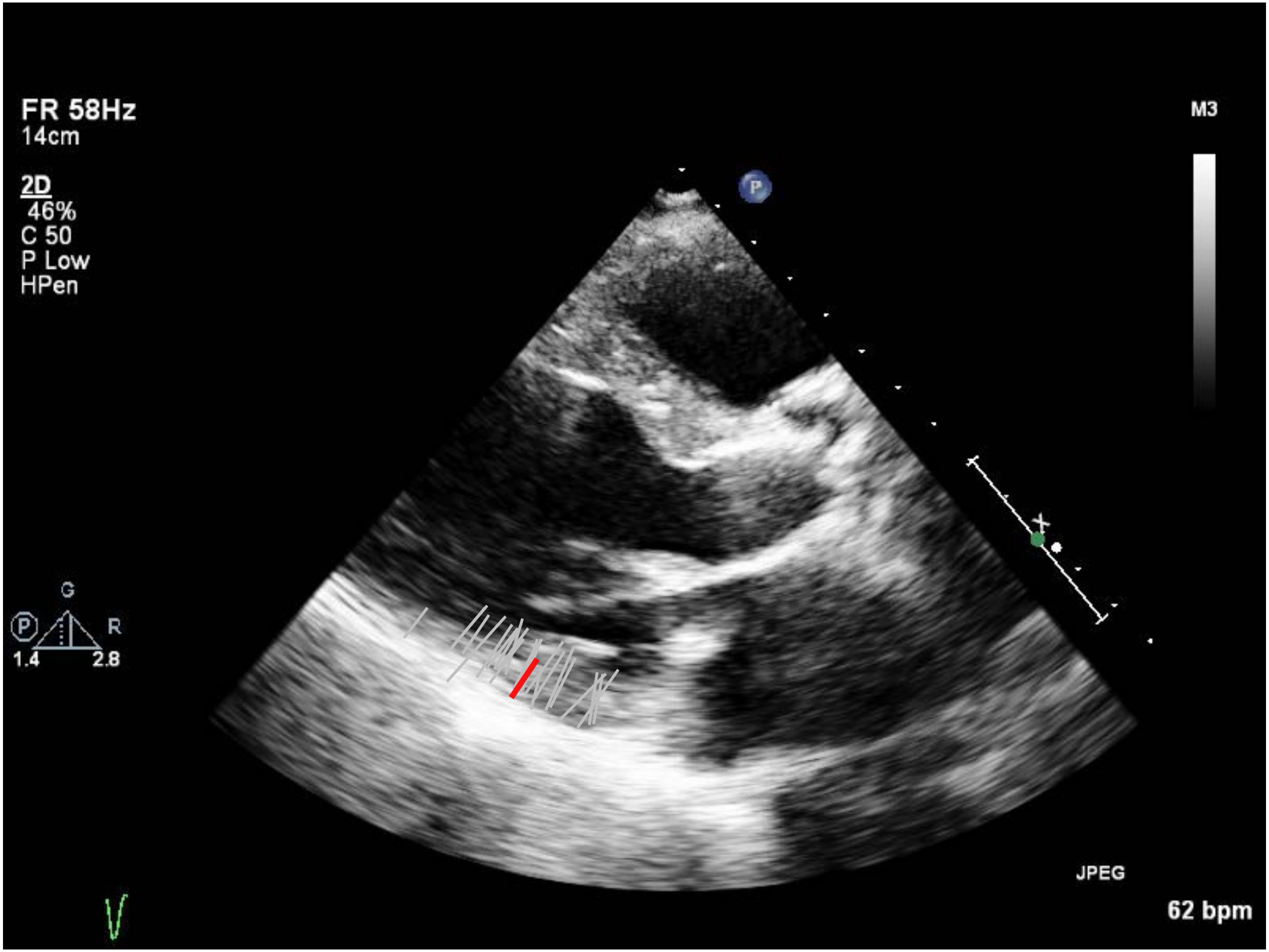

Pt 92 Diastole

Absolute difference between AI and expert consensus: 1.9 mm

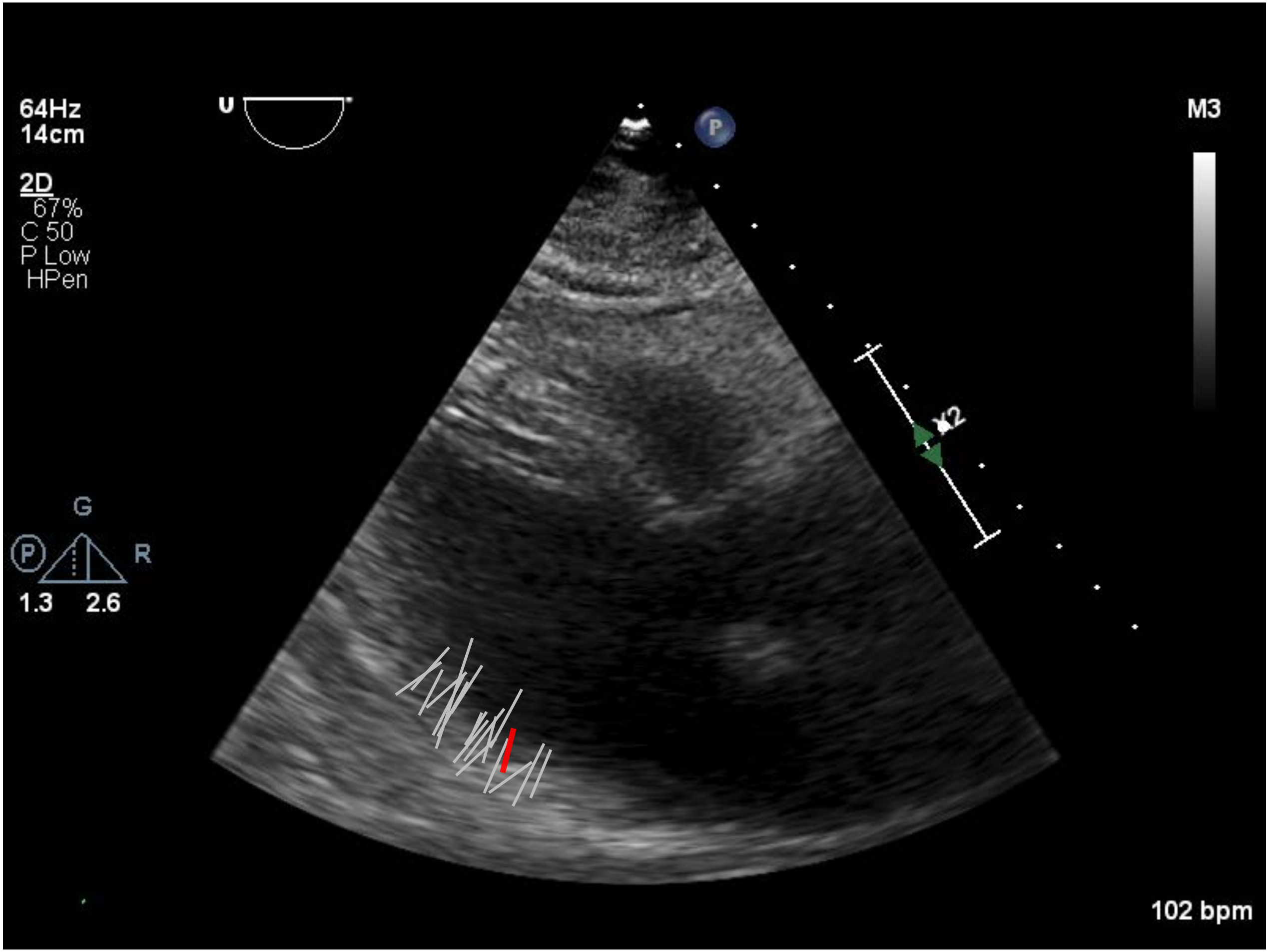

Pt 99 Diastole

Absolute difference between AI and expert consensus: 1.9 mm

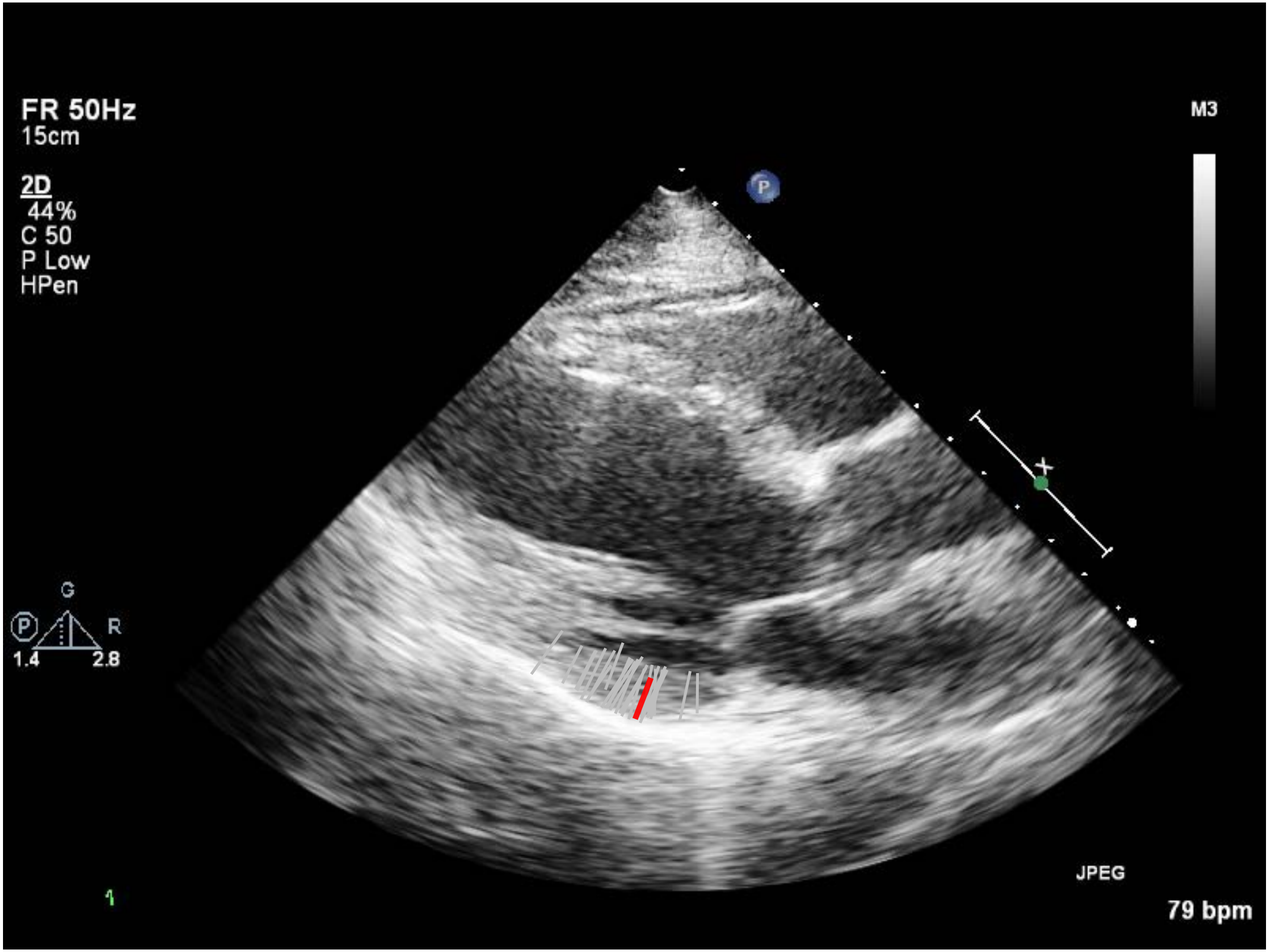

Pt 12 Diastole

Absolute difference between AI and expert consensus: 1.9 mm

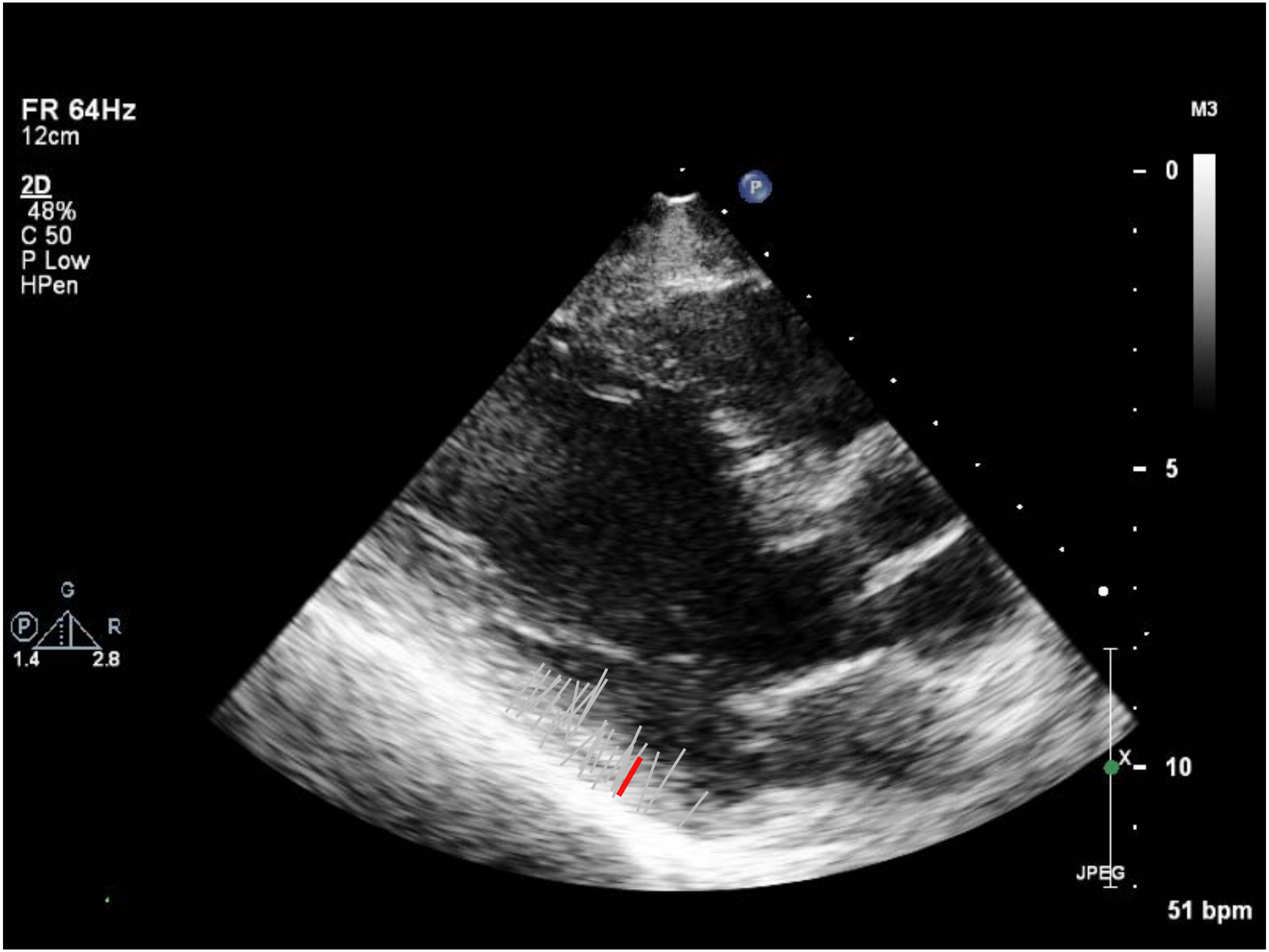

Pt 36 Diastole

Absolute difference between AI and expert consensus: 1.9 mm

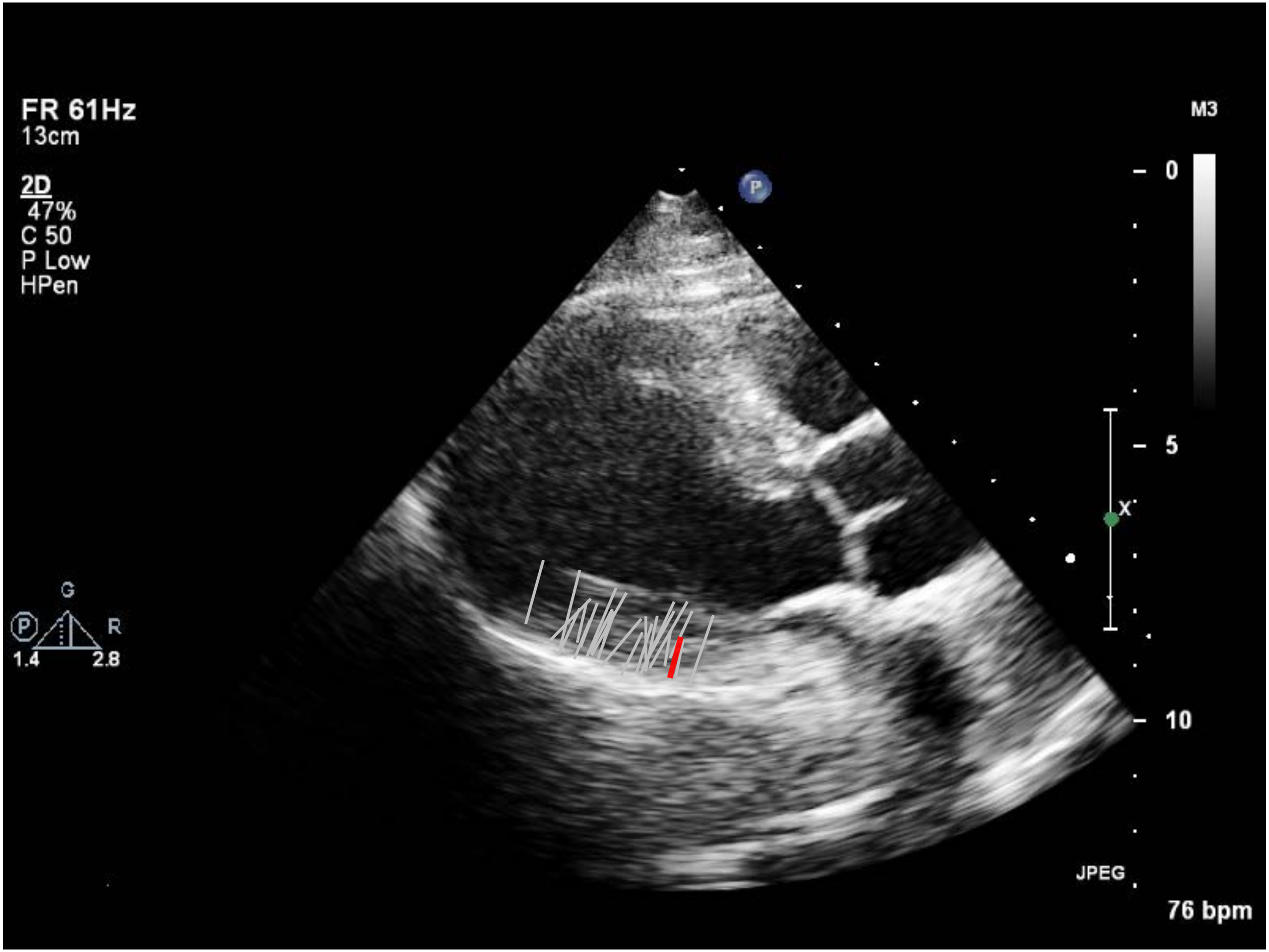

Pt 41 Diastole

Absolute difference between AI and expert consensus: 2 mm

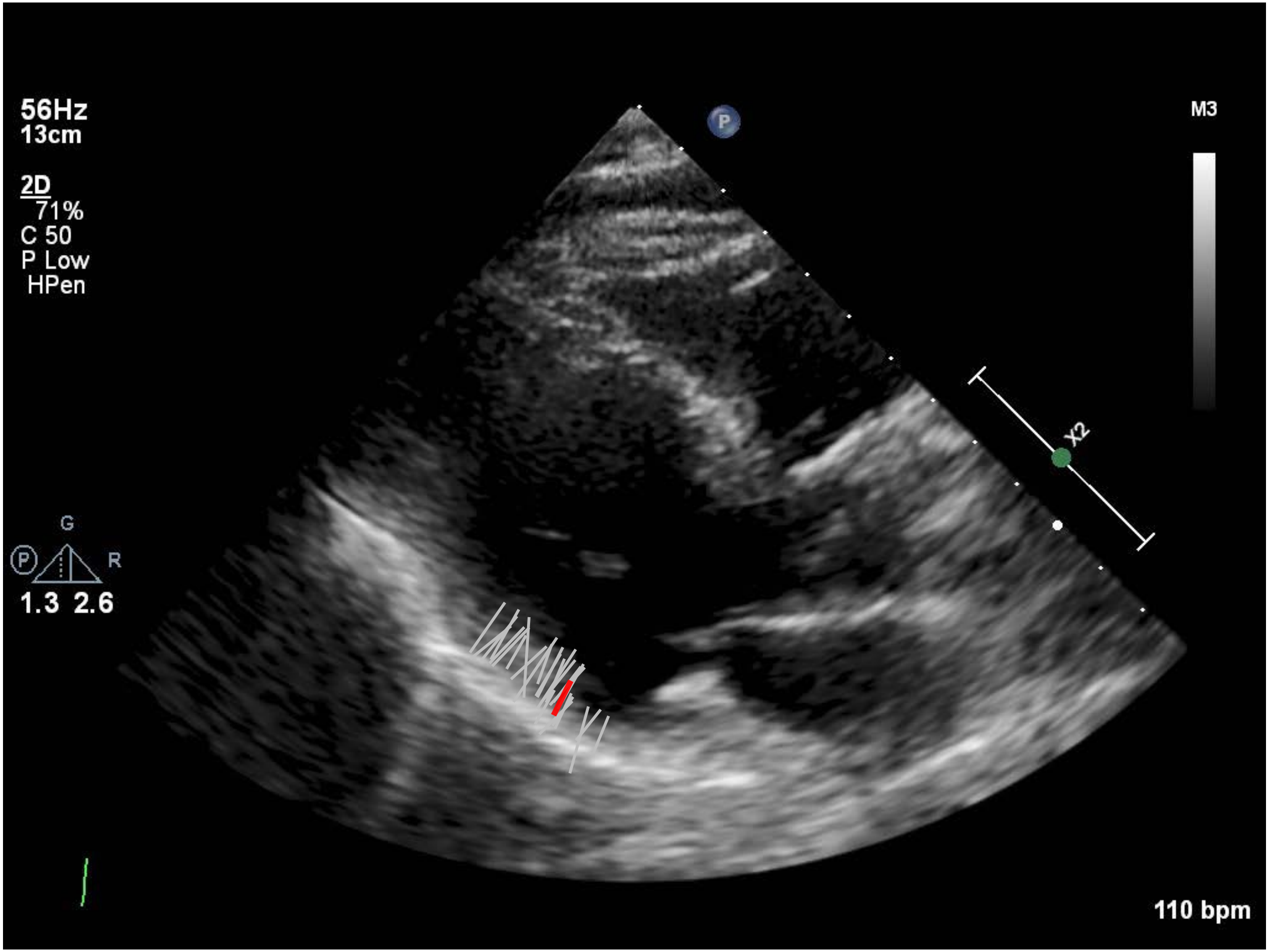

Pt 26 Diastole
Absolute difference between AI and expert consensus: 2.1 mm

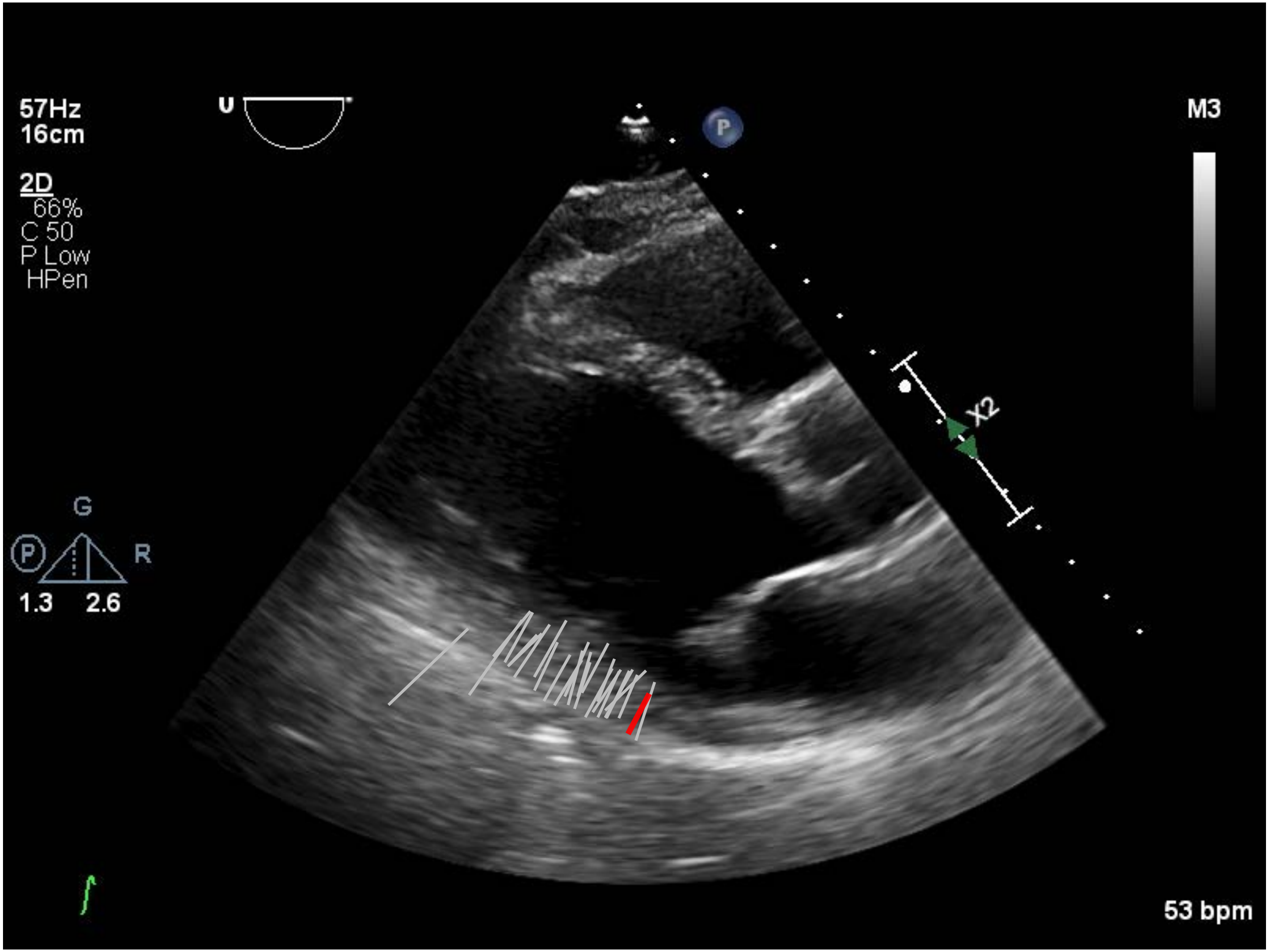

Pt 7 Diastole

Absolute difference between AI and expert consensus: 2.2 mm

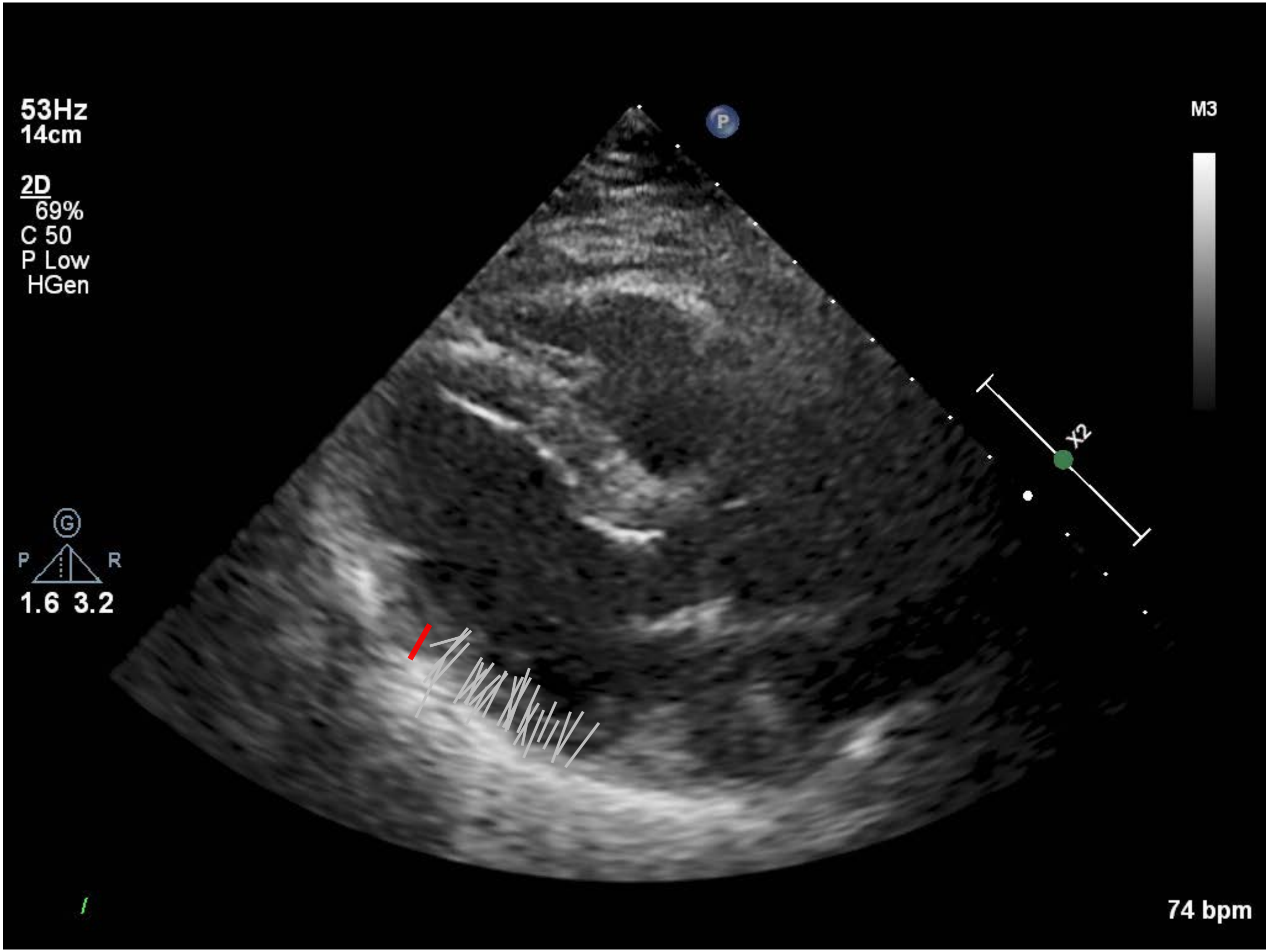

Pt 42 Diastole

Absolute difference between AI and expert consensus: 2.3 mm

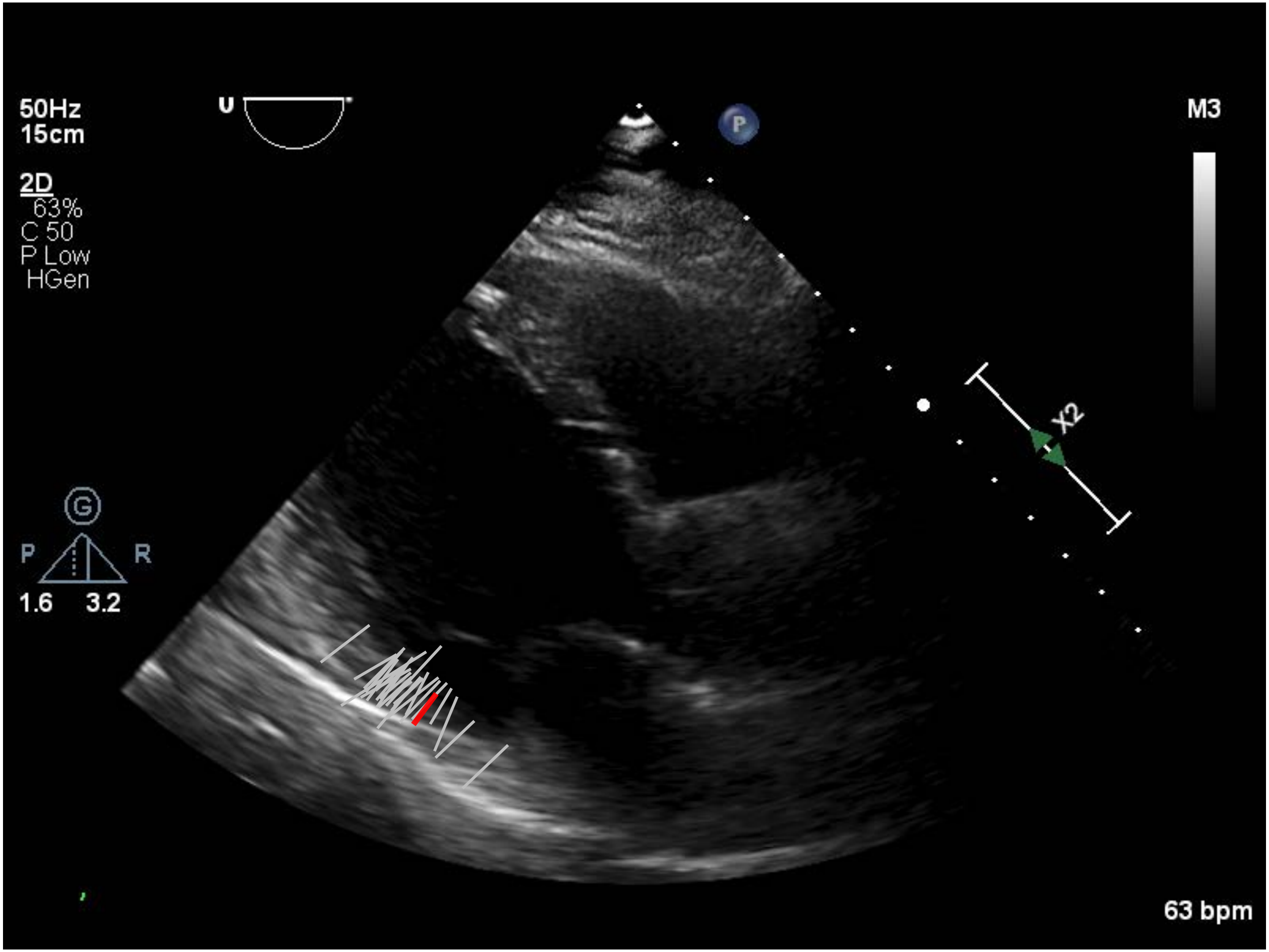

Pt 95 Diastole

Absolute difference between AI and expert consensus: 2.3 mm

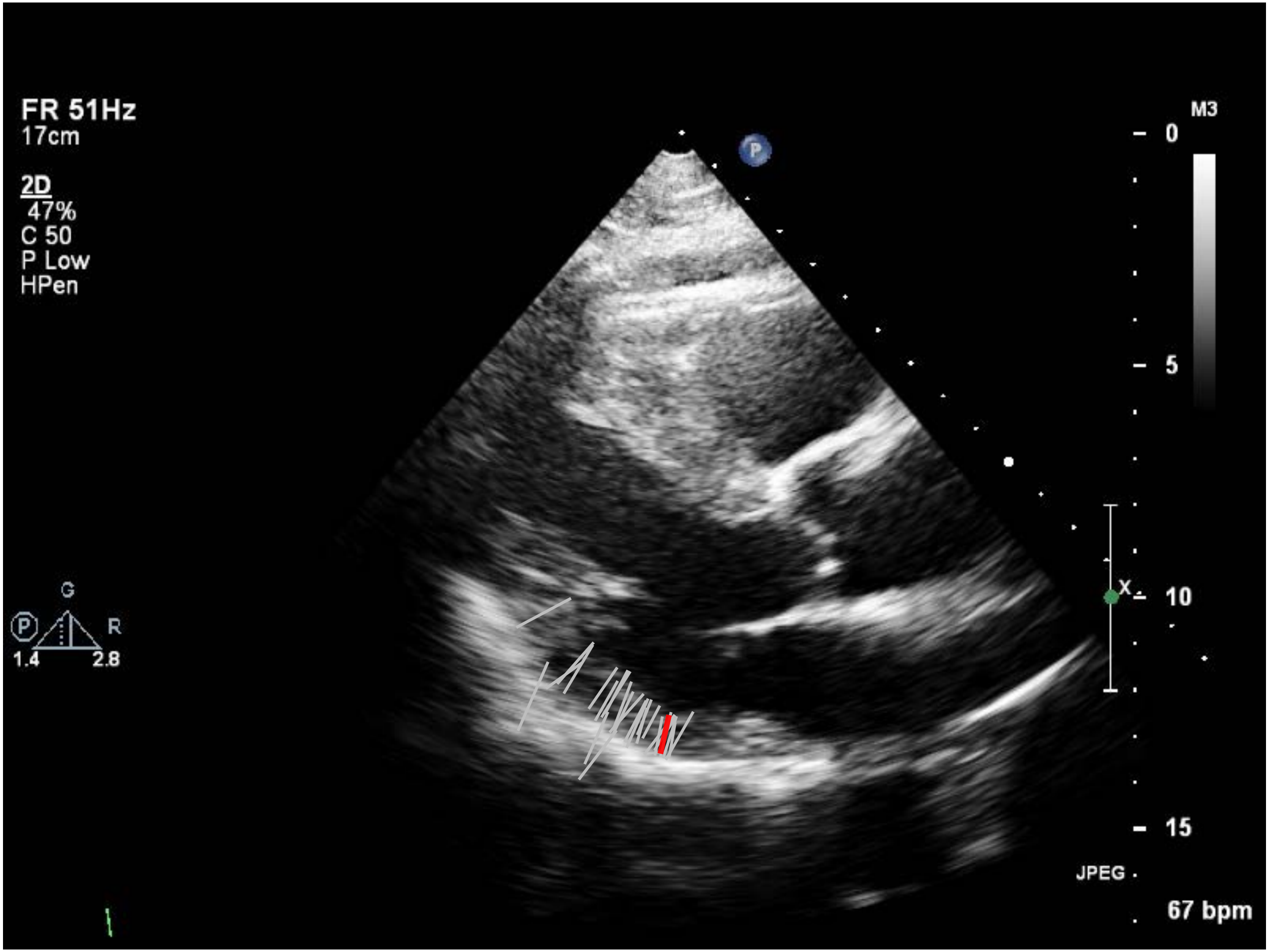

Pt 5 Diastole

Absolute difference between AI and expert consensus: 2.3 mm

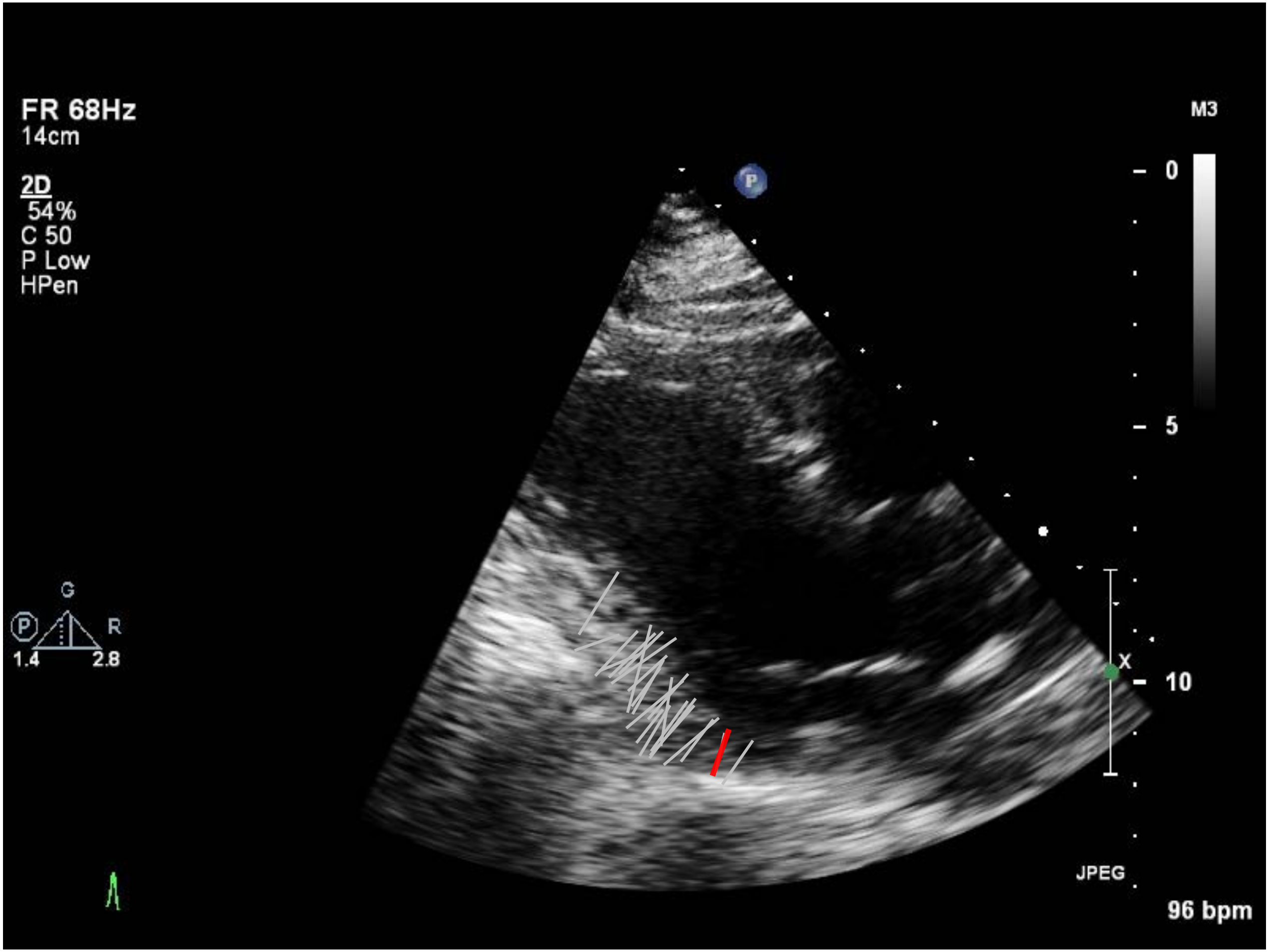

Pt 49 Diastole

Absolute difference between AI and expert consensus: 2.4 mm

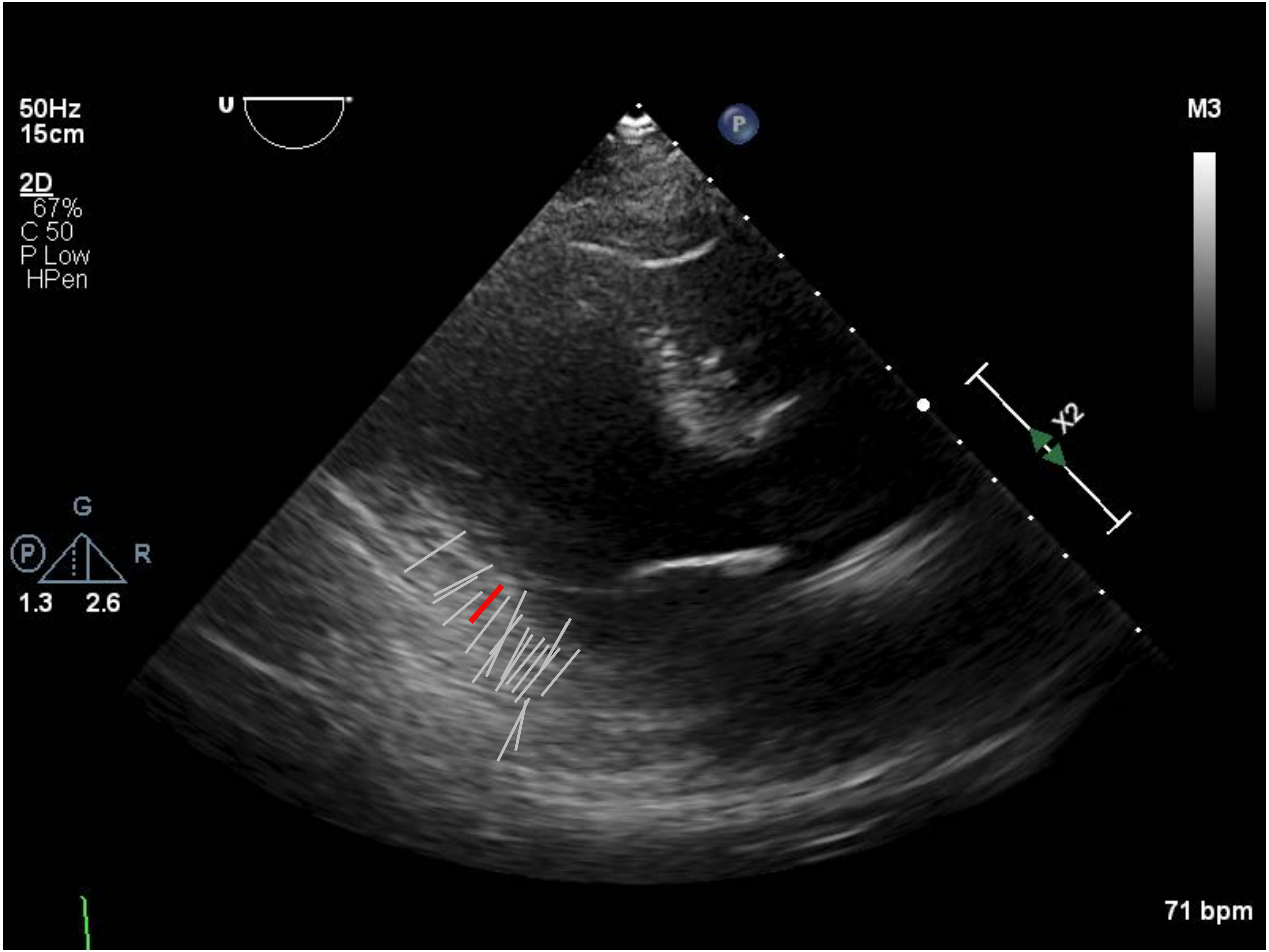

Pt 97 Diastole

Absolute difference between AI and expert consensus: 2.4 mm

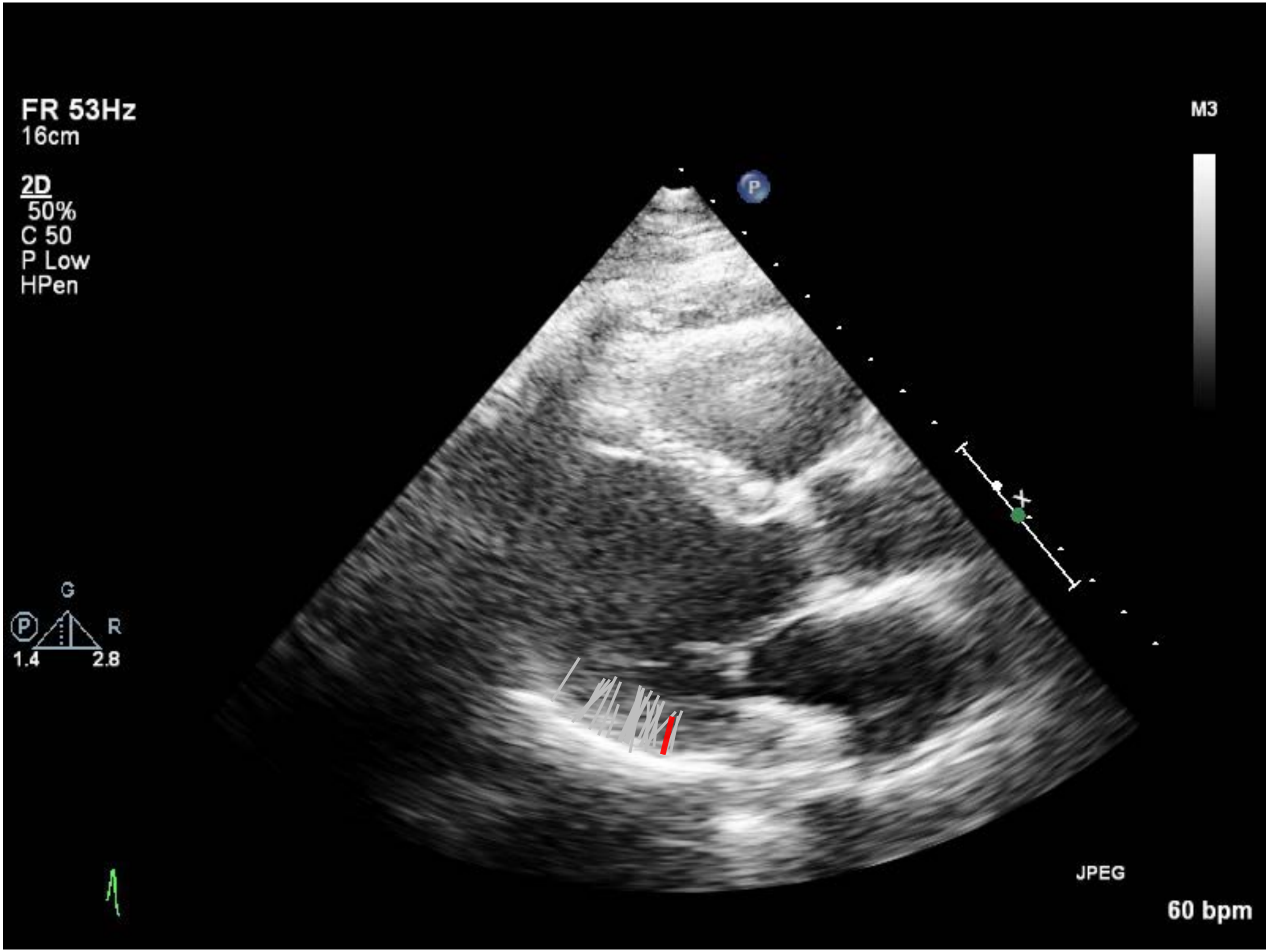

Pt 47 Diastole

Absolute difference between AI and expert consensus: 2.4 mm

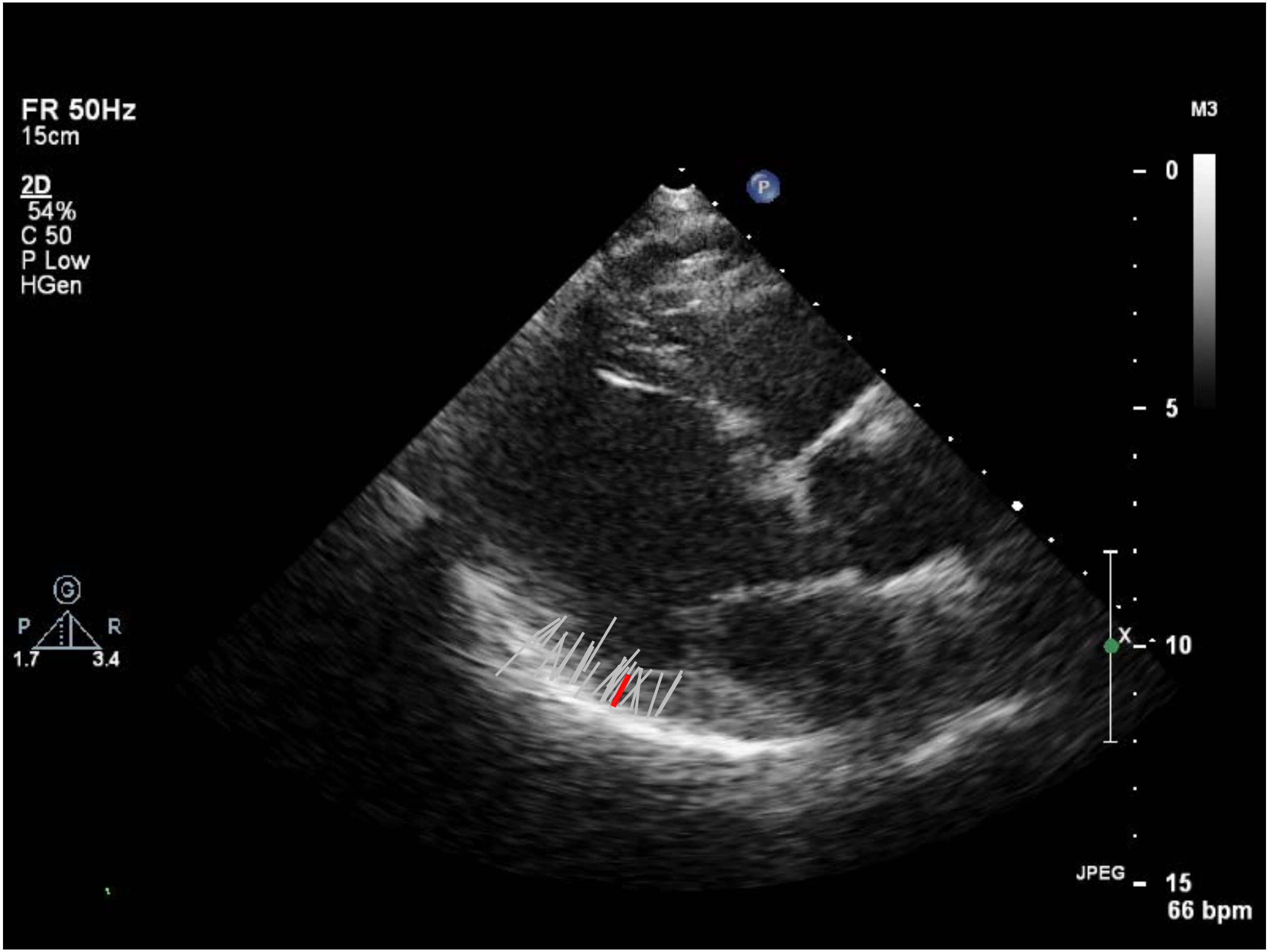

Pt 33 Diastole

Absolute difference between AI and expert consensus: 2.7 mm

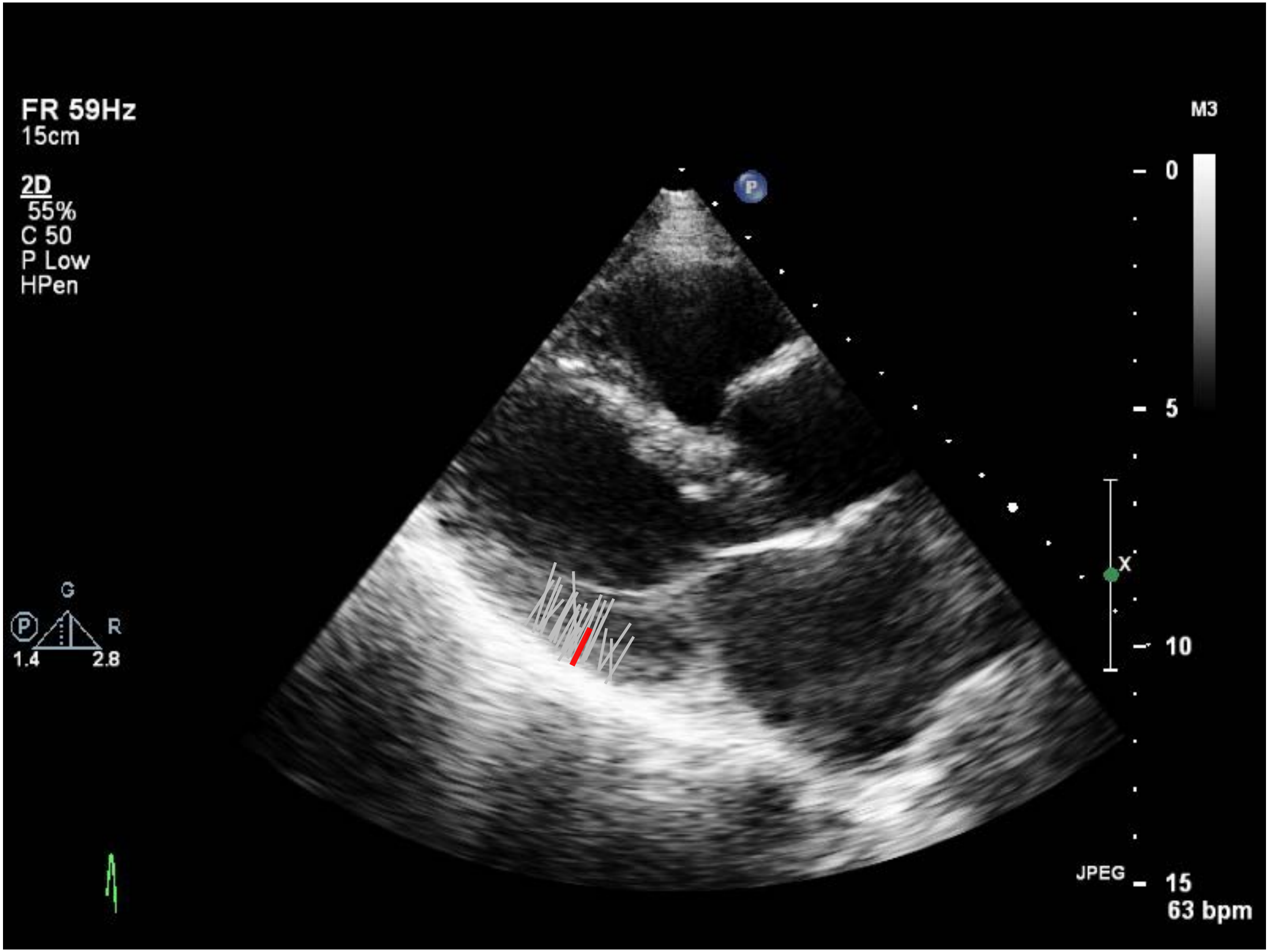

Pt 45 Diastole

Absolute difference between AI and expert consensus: 2.8 mm

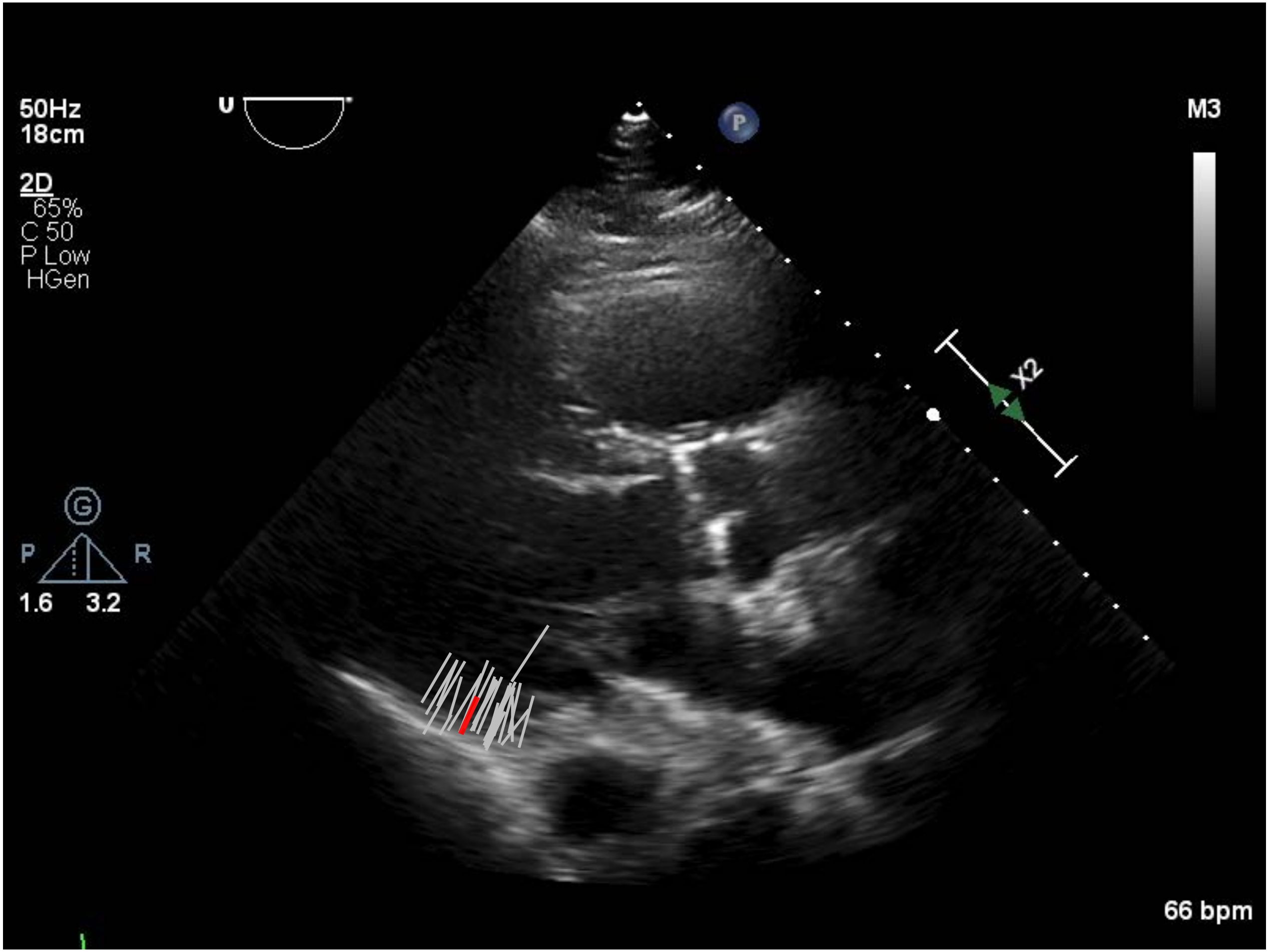

Pt 11 Diastole

Absolute difference between AI and expert consensus: 3.4 mm

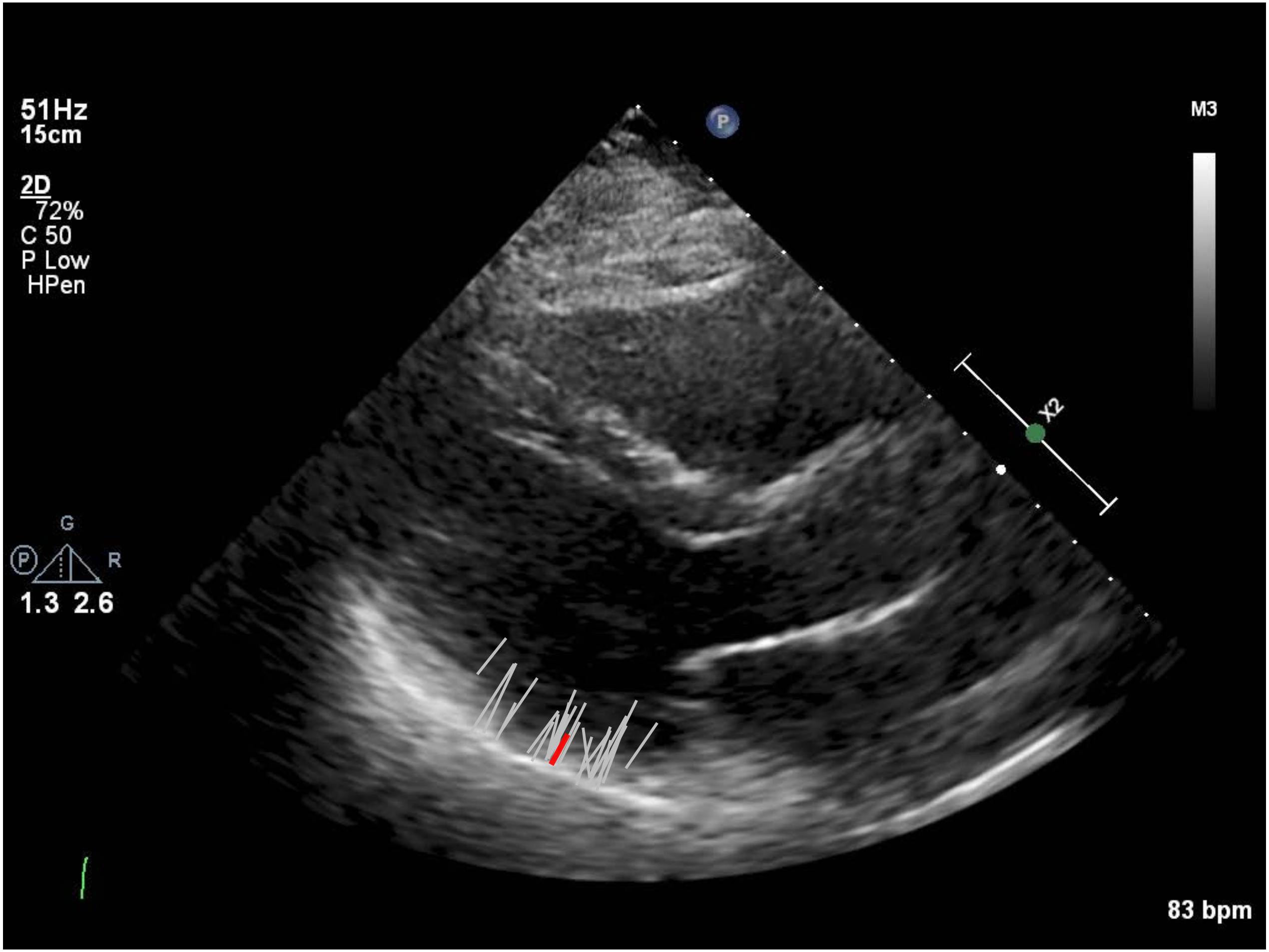

Pt 63 Diastole
Absolute difference between AI and expert consensus: 4 mm

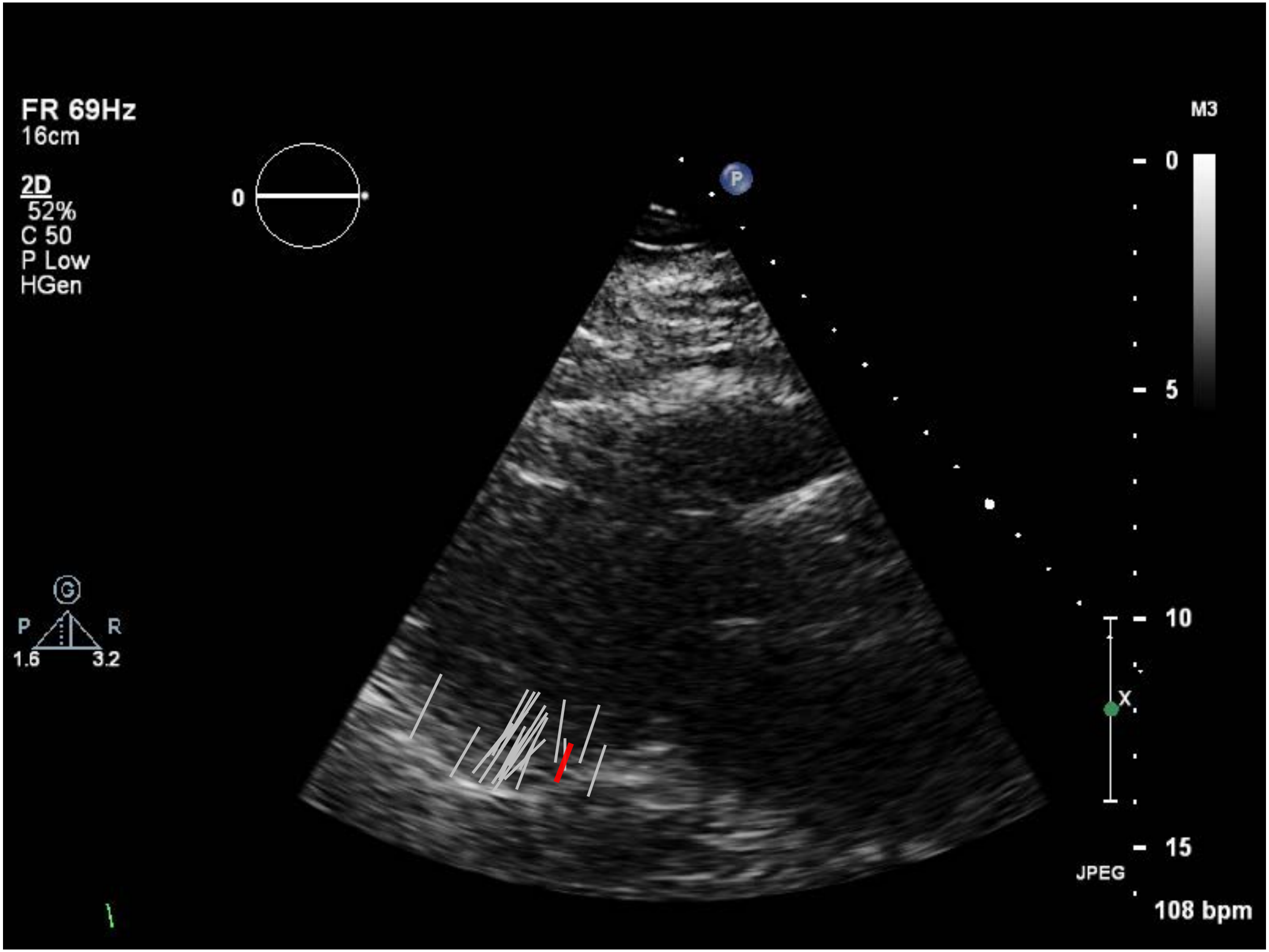

Pt 66 Diastole

Absolute difference between AI and expert consensus: 4.1 mm

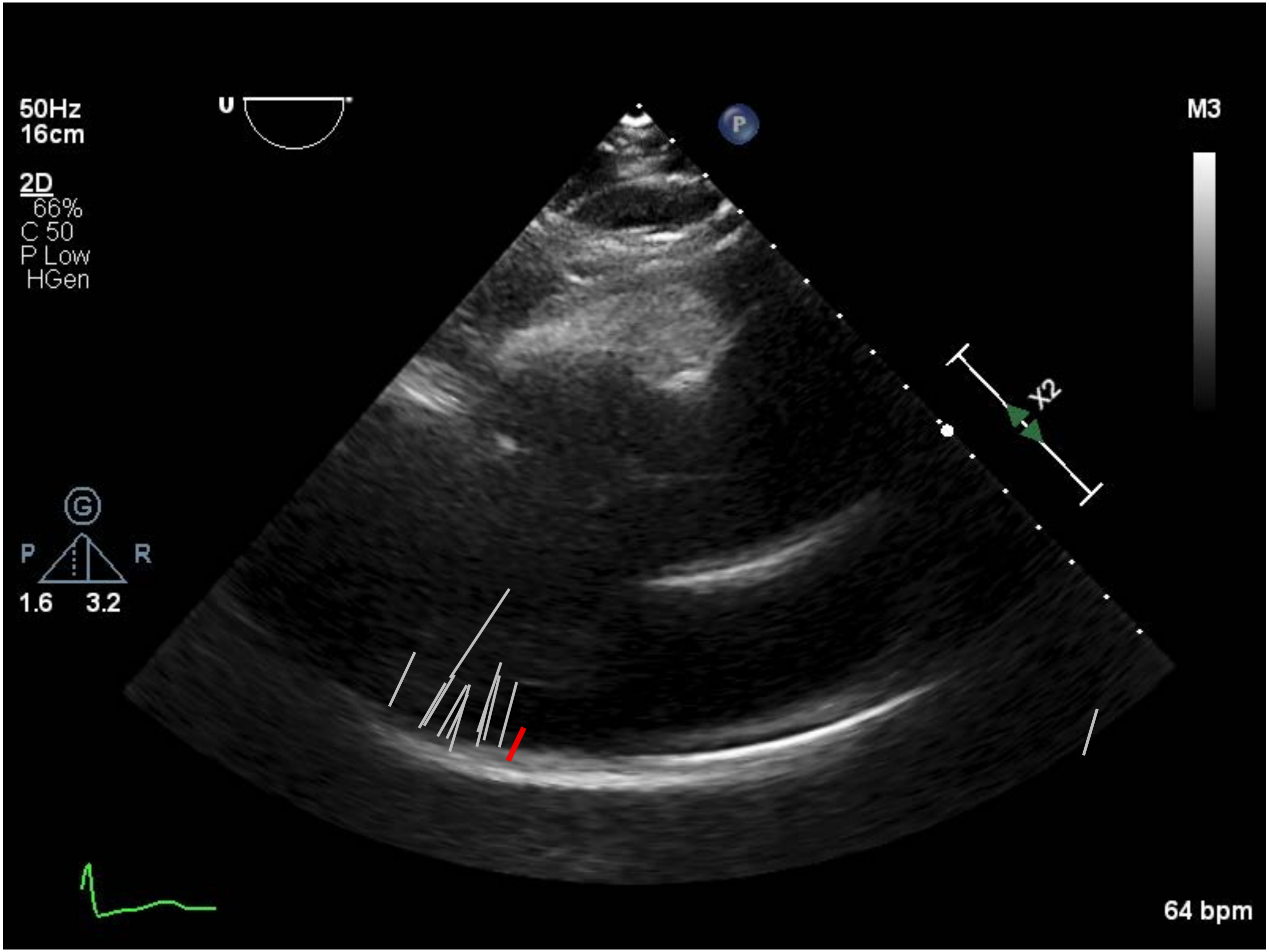

Pt 20 Diastole

Absolute difference between AI and expert consensus: 4.8 mm

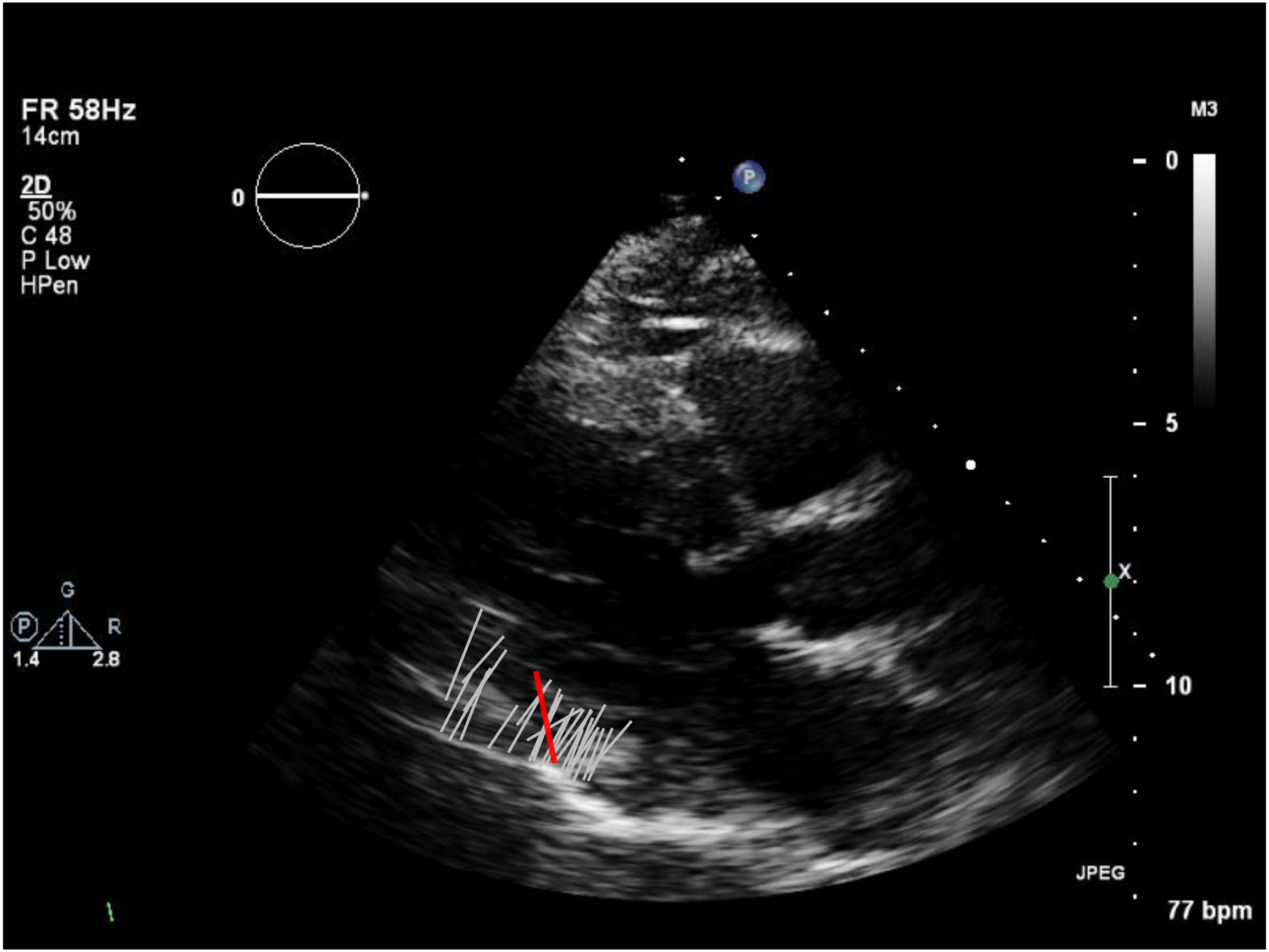

Pt 3 Diastole

Absolute difference between AI and expert consensus: 5.4 mm

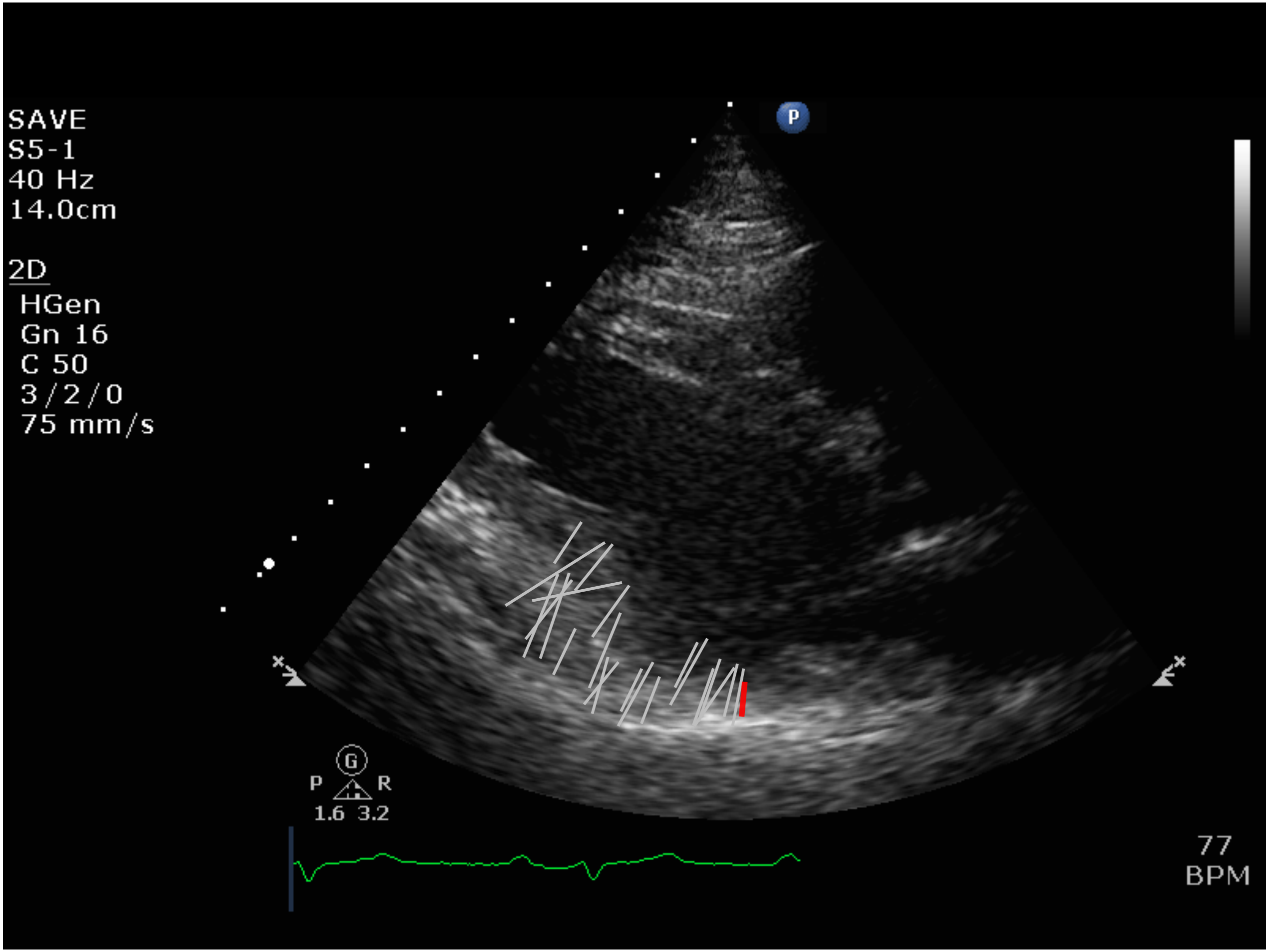

Pt 76 Diastole

Absolute difference between AI and expert consensus: 5.7 mm
